# Supplementary material for: 2,2-difluorovinyl benzoates for diverse synthesis of gem-difluoroenol ethers by Ni-catalyzed cross-coupling reactions
Source: Nat Commun. 2021 Jan 18;12:412. doi: 10.1038/s41467-020-20725-9 (PMC7814061; doi:10.1038/s41467-020-20725-9)
Supplement: Supplementary file 1 — Supplementary Information [file 41467_2020_20725_MOESM1_ESM.pdf]

Supplementary Information

2,2-Difluorovinyl Benzoates for Diverse Synthesis of *gem*-Difluoroenol Ethers by Ni-Catalyzed Cross Coupling Reactions

Bingnan Du[1], Chun-Ming Chan[1,2], Pui-Yiu Lee[1,2], Leong-Hung Cheung[1], Xin Xu[3], Zhenyang Lin[3]* and Wing-Yiu Yu[1]*

[1]State Key Laboratory of Chemical Biology and Drug Discovery and Department of Applied Biology and Chemical Technology, The Hong Kong Polytechnic University, Hung Hom, Kowloon, Hong Kong. [2]These authors contributed equally. [3]Department of Chemistry, The Hong Kong University of Science and Technology, Clear Water Bay, New Territories, Hong Kong.

*e-mail: wing-yiu.yu@polyu.edu.hk

*e-mail: chzlin@ust.hk

Table of Contents

1. General Information 2

2. Preparation and physical characterization data of substrates 3

3. Exploration of the coupling partners with the BzO-DF building blocks 35

4. Preparation and physical characterization data 38

5. Mechanistic studies 58

6. X-ray crystallographic data for 5c, 4s and 9a 80

7. NMR Spectra 109

8. Supplementary References 326

1. General Information

All the reagents were obtained from commercial sources and used without purification unless otherwise specified. All the reagents were obtained from commercial sources and used without purification unless stated otherwise. All solvents used were distilled before use. All glasswares were dried overnight at 150 °C prior to use. Thin layer chromatography (TLC) was performed on silica gel plates. Visualization on TLC was achieved using UV light (254 nm).

Zinc powder was obtained from commercial source and activated with 1 M HCl. The activated zinc was first washed with acetone, and then dried by vacuum for 1 h and stored in a glovebox.

All experiments were performed under a nitrogen atmosphere. Flash column chromatography was performed on silica gel columns (Merck, 230–400 mesh). NMR analysis was recorded on a Brüker DPX-400 MHz spectrometer. HRMS were performed with Agilent 6540 QTOF-MS (ESIMS) and Waters GCT Premier (EIMS). X-ray crystallographic study was performed by a SuperNova, Dual, Cu at zero, AtlasS2 diffractometer. The crystal samples were kept at 294 K (**4s**), 294 K (**5c**) and 100 K (**9a**) during data collection.

2. Preparation and physical characterization data of substrates

Scope of 2-bromo-2,2-difluoroacetates and 2-bromo-2,2-difluoroketones (1)

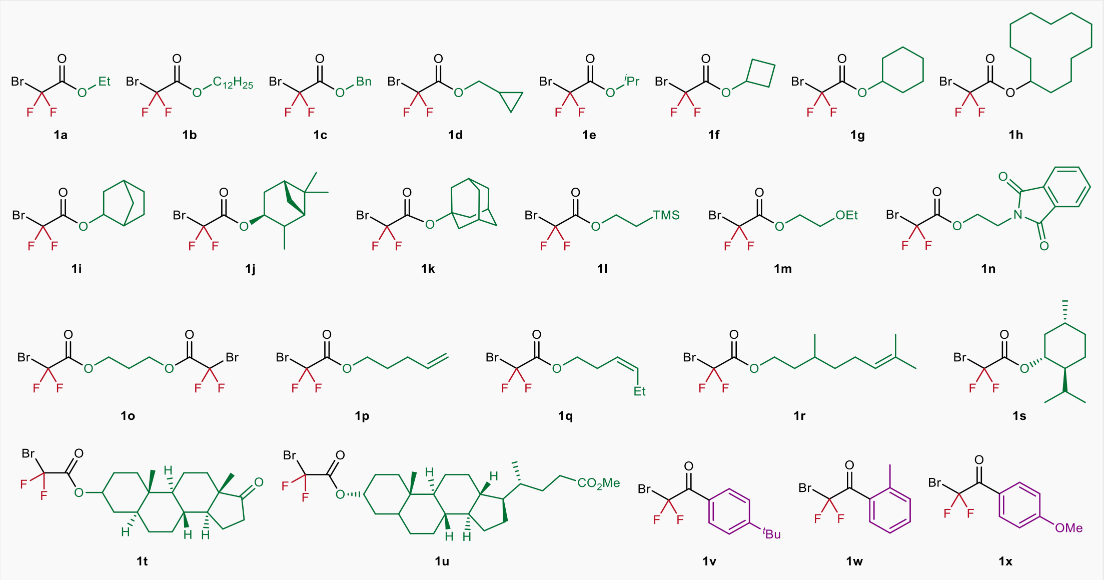

General procedure for preparation of 2-bromo-2,2-difluoroacetates (1)[1]

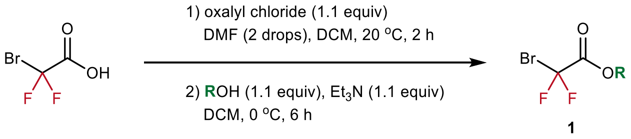

In a glovebox, 2-bromo-2,2-difluoroacetic acid (5.0 mmol, 1.0 equiv, 875 mg) were mixed with dry DCM (15 mL) in a Schlenk flask. The flask was then sealed with a screw cap before taken out from the glovebox. Oxalyl chloride (1.1 equiv, 0.46 mL) was then added slowly into reaction system over 5 min with stirring at 20 °C. Two drops of DMF were then added, and the reaction mixture was stirred at 20 °C for further 2 h before cooling to 0 °C. Then a mixture of alcohol (1.1 equiv) and Et_3N (1.1 equiv, 0.76 mL) dissolved in dry DCM (15 mL) was added dropwise to the reaction mixture.

After stirring for 6 h, water (80 mL) was added to the reaction mixture and the crude was extracted with DCM (30 mL x 3). The combined organic extracts were then dried with anhydrous $MgSO_4$ and concentrated under vacuum. The residue was purified

by flash column chromatography on silica gel to give the corresponding products **1b, 1c, 1f–1x** (59–86% yields).

For 1d*, *1e and 1f

In a glovebox, 2-bromo-2,2-difluoroacetic acid (0.5 mmol, 1.0 equiv) and dry DCM (2 mL) were added to an 8 mL vial. The vial was sealed with a screw cap before taken out from the glovebox. Oxalyl chloride (0.55 mmol, 1.1 equiv) was added slowly into reaction system over 5 min and allowed to stir at 20 ^{o}C. One drop of DMF was then added, and the reaction mixture was stirred at 20 ^{o}C for further 2 h before cooling to 0 ^{o}C. A solution of alcohol (0.55 mmol, 1.1 equiv) and Et_3N (0.55 mmol, 1.1 equiv) dissolved in dry DCM (2 mL) was then added dropwise to the reaction mixture. After stirring for 6 h, water (20 mL) was added to the reaction mixture and the crude was extracted with Et_2O (10 mL x 3). The combined organic extracts were dried with anhydrous $MgSO_4$ and concentrated carefully under vacuum at 10 ^{o}C. Then the crude products **1d**, **1e** and **1f** were used directly for subsequent synthesis to give the corresponding products **4c**, **4d** and **4e**.

General procedure for preparation of bromodifluoroketones (1v–1x)[2]

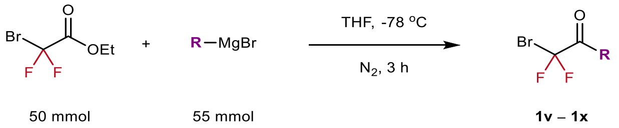

Ethyl 2-bromo-2,2-difluoroacetate (50 mmol, 1.0 equiv, 10.2 g) and dry THF (50 mL) were added to a Schlenk flask in a glovebox. The flask was then sealed with a screw cap before taken out from the glovebox, arylmagnesium bromide solution (1.0 M in THF, 55 mmol, 1.1 equiv, 55 mL) was added slowly into the reaction system over 20 min with vigorous stirring at -78 ^{o}C. The mixture was stirred at -78 ^{o}C for further 3 h before being quenched with 3 N HCl. Water (80 mL) was then added and the organic layer was extracted with diethyl ether (50 mL x 3). The combined organic extracts were dried with anhydrous $MgSO_4$ and concentrated under vacuum. The residue was purified

by flash column chromatography on silica gel to give the corresponding product **1v–1x** (62–77% yields).

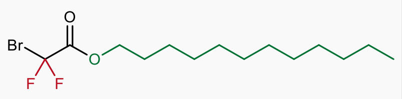

1b

Dodecyl 2-bromo-2,2-difluoroacetate (1b): colorless oil (80%). **^{1}H NMR** (400 MHz, Chloroform-*d*) δ= 4.35 (t, *J* = 6.64 Hz, 2H), 1.75 (m, 2H), 1.43-1.26 (m, 18H), 0.88 (m, 3H) ppm. **^{13}C NMR** (101 MHz, Chloroform-*d*) δ= 159.7 (t, *J* = 31.3 Hz), 108.8 (t, *J* = 315.4 Hz), 68.5, 31.9, 29.6, 29.5, 29.4, 29.3, 29.0, 28.1, 25.5, 22.7, 14.1 ppm. **^{19}F NMR** (376 MHz, Chloroform-*d*) δ= -60.68 (s, 2F) ppm. **HRMS** (ESI): calcd. for $C_{14}H_{26}BrF_2O^+$: 343.1079. found: 343.1087.

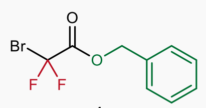

1c

Benzyl 2-bromo-2,2-difluoroacetate (1c): colorless oil (85%). **^{1}H NMR** (400 MHz, Chloroform-*d*) δ= 7.39 (s, 5H), 5.35 (s, 2H) ppm. **^{13}C NMR** (101 MHz, Chloroform-*d*) δ= 159.5 (t, *J* = 31.7 Hz), 129.2, 128.9, 128.5, 127.0, 108.8 (t, *J* = 314.0 Hz), 69.8 ppm. **^{19}F NMR** (376 MHz, Chloroform-*d*) δ= -60.70 (s, 2F) ppm. **HRMS** (EI): calcd. for $C_9H_7BrF_2O_2$: 263.9597. found: 263.9600.

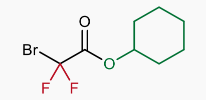

1g

Cyclohexyl 2-bromo-2,2-difluoroacetate (1g): colorless oil (78%). **^{1}H NMR** (400 MHz, Chloroform-*d*) δ= 4.98 (m, 1H), 1.92-1.89 (m, 2H), 1.80-1.76 (m, 2H), 1.65-1.53 (m, 3H), 1.47-1.35 (m, 3H) ppm. **^{13}C NMR** (101 MHz, Chloroform-*d*) δ= 159.1 (t, *J* = 30.9 Hz), 109.1 (t, *J* = 315.9 Hz), 77.7, 30.8, 25.0, 23.2 ppm. **^{19}F NMR** (376 MHz, Chloroform-*d*) δ= -60.87 ppm. **HRMS** (EI): calcd. for $C_8H_{11}BrF_2O_2$: 255.9910. found: 255.9897.

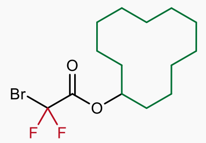

1h

Cyclododecyl 2-bromo-2,2-difluoroacetate (1h): colorless oil (72%). **^{1}H NMR** (400 MHz, Chloroform-*d*) δ= 5.20 (m, 1H), 1.90-1.83 (m, 2H), 1.67-1.61 (m, 2H), 1.45-1.38 (m, 19H) ppm. **^{13}C NMR** (101 MHz, Chloroform-*d*) δ= 159.3 (t, *J* = 30.8 Hz), 109.0 (t, *J* = 316.0 Hz), 78.0, 28.6, 24.0, 23.9, 23.3, 23.1, 20.6 ppm. **^{19}F NMR** (376 MHz, Chloroform-*d*) δ= -60.82 (s, 2F) ppm. **HRMS** (EI): calcd. for $C_{14}H_{23}BrF_2O_2$: 340.0849 found: 340.0872.

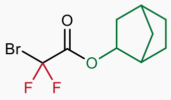

(1S,4R)-bicyclo[2.2.1]heptan-2-yl 2-bromo-2,2-difluoroacetate (1i): colorless oil (64%). **^{1}H NMR** (400 MHz, Chloroform-*d*) δ= 4.81 (d, *J* = 6.88 Hz, 1H), 2.44 (d, *J* = 4.76 Hz, 1H), 2.37 (m, 1H), 1.82-1.79 (m, 1H), 1.65-1.46 (m, 4H), 1.25 (d, *J* = 9.36 Hz, 1H), 1.20-1.10 (m, 2H) ppm. **^{13}C NMR** (101 MHz, Chloroform-*d*) δ= 159.1 (t, *J* = 31.1 Hz), 109.1 (t, *J* = 315.9 Hz), 82.2, 41.2, 38.9, 35.3, 35.2, 27.9, 23.9 ppm. **^{19}F NMR** (376 MHz, Chloroform-*d*) δ= -60.98 ppm. **HRMS** (EI): calcd. for $C_9H_{11}BrF_2O_2$: 267.9910. found: 267.9905.

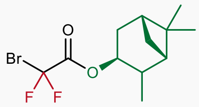

(1S,3S,5R)-2,6,6-trimethylbicyclo[3.1.1]heptan-3-yl 2-bromo-2,2-difluoroacetate (1j): colorless oil (59%). **^{1}H NMR** (400 MHz, Chloroform-*d*) δ= 4.51 (s, 1H), 1.80-1.74 (m, 4H), 1.64-1.61 (m, 1H), 1.54-1.47 (m, 1H), 1.27 (m, 1H), 1.14 (s, 3H), 1.10 (s, 3H), 0.87 (s, 3H) ppm. **^{13}C NMR** (101 MHz, Chloroform-*d*) δ= 160.0 (t, *J* = 31.1 Hz), 108.9 (t, *J* = 315.7 Hz), 90.8, 48.7, 48.2, 41.2, 40.0, 29.6, 26.3, 25.7, 20.0, 19.1 ppm. **^{19}F NMR** (376 MHz, Chloroform-*d*) δ= -60.15 (d, *J* = 16.28 Hz, 2F) ppm. **HRMS** (EI): calcd. for $C_{12}H_{17}BrF_2O_2$: 310.0380. found: 310.0378.

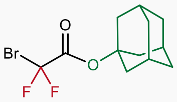

(3S,5S,7S)-adamantan-1-yl 2-bromo-2,2-difluoroacetate (1k): colorless oil (67%). **^{1}H NMR** (400 MHz, Chloroform-*d*) δ= 2.25 (s, 3H), 2.19 (s, 6H), 1.70 (s, 6H) ppm. **^{13}C NMR** (101 MHz, Chloroform-*d*) δ= 157.7 (t, *J* = 30.4 Hz), 109.2 (t, *J* = 317.2 Hz), 86.6, 40.7, 35.8, 31.0 ppm. **^{19}F NMR** (376 MHz, Chloroform-*d*) δ= -60.88 ppm. **HRMS** (EI): calcd. for $C_{12}H_{15}BrF_2O_2$: 308.0223. found: 308.0223.

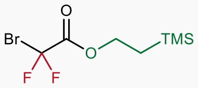

2-(Trimethylsilyl)ethyl 2-bromo-2,2-difluoroacetate (1l): colorless oil (86%). **^{1}H NMR** (400 MHz, Chloroform-*d*) δ= 4.44 (m, 2H), 1.13 (m, 2H), 0.08 (s, 9H) ppm.**^{13}C NMR** (101 MHz, Chloroform-*d*) δ= 159.7 (t, *J* = 31.1 Hz), 109.0 (t, *J* = 315.5 Hz), 67.4, 17.1, -1.6 ppm. **^{19}F NMR** (376 MHz, Chloroform-*d*) δ= -60.71 ppm. **HRMS** (EI): calcd. for $CBrF_2$: 128.9151. found: 128.9156.

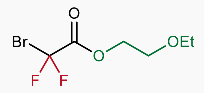

1m

2-Ethoxyethyl 2-bromo-2,2-difluoroacetate (1m): colorless oil (71%). **^{1}H NMR** (400 MHz, Chloroform-*d*) δ= 4.48 (m, 2H), 3.72 (m, 2H), 3.55 (q, *J* = 6.96 Hz, 2H), 1.21 (t, *J* = 7.04 Hz, 3H) ppm. **^{13}C NMR** (101 MHz, Chloroform-*d*) δ= 159.6 (t, *J* = 31.6 Hz), 108.6 (t, *J* = 315.3 Hz), 67.4, 67.2, 66.9, 15.0 ppm. **^{19}F NMR** (376 MHz, Chloroform-*d*) δ= -60.75 ppm. **HRMS** (EI): calcd. for $C_6H_9BrF_2O_3$: 245.9703. found: 245.9704.

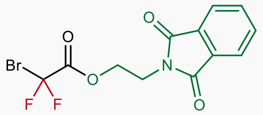

1n

2-(1,3-Dioxoisoindolin-2-yl)ethyl 2-bromo-2,2-difluoroacetate (1n): colorless oil (76%), white solid, melting point: 51 ^{o}C. **^{1}H NMR** (400 MHz, Chloroform-*d*) δ= 7.88 (m, 2H), 7.76 (m, 2H), 4.61 (t, *J* = 5.36 Hz, 2H), 4.09 (t, *J* = 5.40 Hz, 2H) ppm. **^{13}C NMR** (101 MHz, Chloroform-*d*) δ= 167.8, 159.4 (t, *J* = 31.9 Hz), 134.3, 131.8, 123.6, 108.3 (t, *J* = 315.5 Hz), 65.0, 36.2 ppm. **^{19}F NMR** (376 MHz, Chloroform-*d*) δ= -60.76 (s, 2F) ppm. **HRMS** (ESI): calcd. for $C_{12}H_8BrF_2NO_4Na^+$: 369.9497. found: 369.9491.

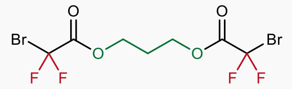

1o

Propane-1,3-diyl bis(2-bromo-2,2-difluoroacetate) (1o): colorless oil (72%). **^{1}H NMR** (400 MHz, Chloroform-*d*) δ= 4.49 (t, *J* = 6.08 Hz, 4H), 2.25 (m, 2H) ppm. **^{13}C NMR** (101 MHz, Chloroform-*d*) δ= 159.4 (t, *J* = 31.7 Hz), 108.4 (t, *J* = 315.5 Hz), 64.0, 27.2 ppm. **^{19}F NMR** (376 MHz, Chloroform-*d*) δ= -60.94 (s, 4F) ppm. **HRMS** (EI): calcd. for $C_5H_6BrF_2O_2$: 214.9519. found: 214.9520.

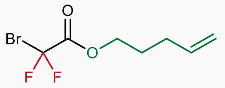

1p

Pent-4-en-1-yl 2-bromo-2,2-difluoroacetate (1p): colorless oil (76%). **^{1}H NMR** (400 MHz, Chloroform-*d*) δ= 5.85-5.75 (m, 1H), 5.10-5.03 (m, 2H), 4.37 (t, *J* = 6.52 Hz, 2H), 2.19 (q, *J* = 7.0 Hz, 2H), 1.86 (m, 2H) ppm. **^{13}C NMR** (101 MHz, Chloroform-*d*) δ= 159.6 (t, *J* = 31.4 Hz), 136.6, 116.0, 108.7 (t, *J* = 315.4 Hz), 67.6, 29.6, 27.2 ppm. **^{19}F NMR** (376 MHz, Chloroform-*d*) δ= -60.76 ppm. **HRMS** (EI): calcd. for $C_7H_9BrF_2O_2$: 241.9754. found: 241.9760.

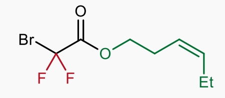

1q

(*Z*)-hex-3-en-1-yl 2-bromo-2,2-difluoroacetate (1q): colorless oil (61%). **^{1}H NMR** (400 MHz, Chloroform-*d*) δ= 5.57 (m, 1H), 5.31 (m, 1H), 4.34 (t, *J* = 6.84 Hz, 2H), 2.50 (q, *J* = 6.88 Hz, 2H), 2.07 (m, 2H), 0.98 (t, *J* = 7.56 Hz, 3H) ppm. **^{13}C NMR** (101 MHz, Chloroform-*d*) δ= 159.6 (t, *J* = 31.3 Hz), 135.8, 122.1, 108.7 (t, *J* = 315.5 Hz), 67.6, 26.3, 20.6, 14.1 ppm. **^{19}F NMR** (376 MHz, Chloroform-*d*) δ= -60.68 ppm. **HRMS** (EI): calcd. for $C_8H_{11}BrF_2O_2$: 255.9910. found: 255.9912.

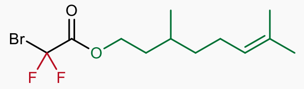

1r

3,7-Dimethyloct-6-en-1-yl 2-bromo-2,2-difluoroacetate (1r): colorless oil (65%). **^{1}H NMR** (400 MHz, Chloroform-*d*) δ= 5.08 (t, *J* = 7.06 Hz, 1H), 4.41-4.37 (m, 2H), 2.06-1.94 (m, 2H), 1.82-1.76 (m, 1H), 1.68 (s, 3H), 1.61-1.53 (m, 5H), 1.41-1.33 (m, 1H), 1.26-1.19 (m, 1H), 0.95 (d, *J* = 6.40 Hz, 3H) ppm. **^{13}C NMR** (101 MHz, Chloroform-*d*) δ= 159.6 (t, *J* = 31.3 Hz), 131.6, 124.2, 108.8 (t, *J* = 315.6 Hz), 66.9, 36.8, 34.9, 29.2, 25.7, 25.3, 19.3, 17.6 ppm. **^{19}F NMR** (376 MHz, Chloroform-*d*) δ= -60.72 ppm. **HRMS** (EI): calcd. for 312.0536. found: 312.0538.

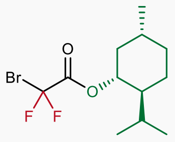

1s

(1S,2R,5S)-2-isopropyl-5-methylcyclohexyl 2-bromo-2,2-difluoroacetate (1s): colorless oil (86%). **^{1}H NMR** (400 MHz, Chloroform-*d*) δ= 4.85 (td, *J* = 10.92, 4.44 Hz, 1H), 2.07 (m, 1H), 1.93 (m, 1H), 1.74 (m, 2H), 1.55 (m, 2H), 1.13 (m, 2H), 0.94 (m, 7H), 0.79 (d, *J* = 7.00 Hz, 3H) ppm. **^{13}C NMR** (101 MHz, Chloroform-*d*) δ= 159.2 (t, *J* = 31.0 Hz), 108.9 (t, *J* = 316.0 Hz), 79.5, 46.8, 39.9, 33.9, 31.4, 26.2, 23.3, 21.8, 20.6, 16.1 ppm. **^{19}F NMR** (376 MHz, Chloroform-*d*) δ= -60.85 (d, *J* = 8.54 Hz, 2F) ppm. **HRMS** (EI): calcd. for $CBrF_2$: 128.9151. found: 128.9157.

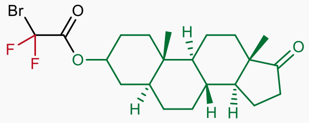

1t

(5S,8R,9S,10S,13S,14S)-10,13-dimethyl-17-oxohexadecahydro-1H-cyclopenta[a]phenanthren-3-yl 2-bromo-2,2-difluoroacetate (1t): white solid, melting point:108 ^{o}C (74%). **^{1}H NMR** (400 MHz, Chloroform-*d*) δ= 5.22 (m, 1H), 2.48-2.41 (m, 1H), 2.12-2.03 (m, 1H), 1.98-1.92 (m, 1H), 1.91-1.66 (m,

6H), 1.62-1.48 (m, 6H), 1.35-1.22 (m, 6H), 1.10-1.00 (m, 1H), 0.87 (s, 3H), 0.85 (s, 3H) ppm. **^{13}C NMR** (101 MHz, Chloroform-*d*) δ= 159.0 (t, *J* = 30.9 Hz), 109.1 (t, *J* = 315.1 Hz), 75.7, 54.1, 51.4, 47.8, 39.9, 35.9, 35.8, 35.0, 32.6, 32.3, 31.5, 30.6, 27.9, 25.6, 21.7, 20.1, 13.8, 11.3 ppm. **^{19}F NMR** (376 MHz, Chloroform-*d*) δ= -60.75 ppm. **HRMS** (EI): calcd. for $C_{21}H_{29}BrF_2O_2$: 430.1319. found: 430.1322.

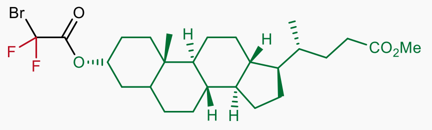

1u

Methyl(4R)-4-((8R,9S,10S,13R,14S,17R)-3-(2-bromo-2,2-difluoroacetoxy)-10,13-dimethylhexadecahydro-1H-cyclopenta[a]phenanthren-17-yl)pentanoate (1u): white solid, melting point: 84 °C (71%). **^{1}H NMR** (400 MHz, Chloroform-*d*) δ= 4.96-4.88 (m, 1H), 3.66 (s, 3H), 2.39-2.32 (m, 1H), 2.26-2.18 (m, 1H), 2.00-1.18 (m, 6H), 1.67-1.02 (m, 19H), 0.95 (s, 3H), 0.91 (d, *J* = 6.4 Hz, 3H), 0.65 (s, 3H) ppm. **^{13}C NMR** (101 MHz, Chloroform-*d*) δ= 174.7, 159.1 (t, *J* = 30.9 Hz), 109.1 (t, *J* = 316.1 Hz), 79.5, 56.4, 56.0, 51.5, 42.7, 41.9, 40.4, 40.0, 35.8, 35.3, 34.8, 34.6, 31.5, 31.0, 30.9, 28.2, 26.9, 26.2, 26.0, 24.2, 23.2, 20.9, 18.3, 12.0 ppm. **^{19}F NMR** (376 MHz, Chloroform-*d*) δ= -60.90 ppm. **HRMS** (ESI): calcd. for $C_{27}H_{41}BrF_2NaO_4^+$: 569.2049. found: 569.2045.

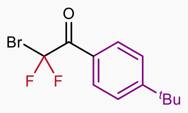

1v

2-bromo-1-(4-(tert-butyl)phenyl)-2,2-difluoroethan-1-one (1v): colorless oil (77%). **^{1}H NMR** (400 MHz, Chloroform-*d*) δ= 7.94 (d, *J* = 7.70 Hz, 1H), 7.50 (m, 1H), 7.36-7.30 (m, 2H), 2.54 (s, 3H) ppm. **^{13}C NMR** (101 MHz, Chloroform-*d*) δ= 183.9 (t, *J* = 25.4 Hz), 141.6, 133.4, 132.4, 130.0 (t, *J* = 4.55 Hz), 129.4, 125.6, 114.2 (t, *J* = 320.1 Hz), 21.4 ppm. **^{19}F NMR** (376 MHz, Chloroform-*d*) δ= -57.83 (s, 2F) ppm. **HRMS** (ESI): calcd. for $C_{12}H_{14}BrF_2O^+$: 291.0191. found: 291.0200.

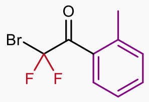

1w

2-bromo-2,2-difluoro-1-(o-tolyl)ethan-1-one (1w): colorless oil (70%). **^{1}H NMR** (400 MHz, Chloroform-*d*) δ= 7.94 (d, *J* = 7.70 Hz, 1H), 7.50 (m, 1H), 7.36-7.30 (m, 2H), 2.54 (s, 3H) ppm. **^{13}C NMR** (101 MHz, Chloroform-*d*) δ= 183.9 (t, *J* = 25.4 Hz), 141.6, 133.4, 132.4, 130.0 (t, *J* = 4.55 Hz), 129.4, 125.6, 114.2 (t, *J* = 320.1 Hz),

21.4 ppm. ^{19}**F NMR** (376 MHz, Chloroform-*d*) δ= -57.83 (s, 2F) ppm. **HRMS** (EI): calcd. for C_8H_7O: 119.0497. found: 119.0498.

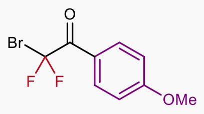

1x

2-Bromo-2,2-difluoro-1-(4-methoxyphenyl)ethan-1-one (1x): colorless oil (62%). ^{1}**H NMR** (400 MHz, Chloroform-*d*) δ= 8.13 (d, *J* = 9.04 Hz, 2H), 6.99 (d, *J* = 9.04 Hz, 2H), 3.91 (s, 3H) ppm. ^{13}**C NMR** (101 MHz, Chloroform-*d*) δ= 180.0 (t, *J* = 25.7 Hz), 165.1, 133.3 (t, *J* = 2.8 Hz), 121.7, 114.3, 113.9 (t, *J* = 319.9 Hz), 55.7 ppm. ^{19}**F NMR** (376 MHz, Chloroform-*d*) δ= -57.00 (s, 2F) ppm. **HRMS** (EI): calcd. for $C_9H_7BrF_2O_2$: 263.9597. found: 263.9600.

Preparation of BzO-DF – Initial trial

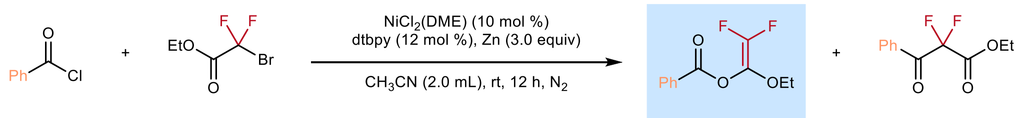

(0.2 mmol) | **1a** (0.4 mmol) | **3a** | **3a'**

	3a	3a'
(a) with Ethyl acetate (0.2 mL)	<5%	44%
(b) without Zn	n.d	n.d
**(c) without [Ni] and dtbpy**	**67%**	**trace**

Preliminarily, benzoyl chloride (0.2 mmol) was mixed with **1a** (0.4 mmol), $NiCl_2$(DME) (10 mol%), dtbpy (12 mol%) and Zn powder (3.0 equiv). Control experiments indicated that only Zn powder was effective for the preparation of **3a**.

Supplementary Table 1. Optimization studies for preparation of BzO-DF (3a)

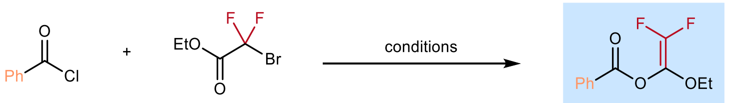

(0.5 mmol) | **1a** (1.0 mmol) | **3a**

entry	Metal (2.0 equiv)	Temp. (ºC)	solvent A (mL)	solvent B (mL)	yield (%)
1	Zn	30	-	dioxane (2.0 + 0.5)	68
2	Zn	30	CH_3CN (2.0 + 0.5)	-	62
3	Zn	30	CH_3CN (0.25)	dioxane (1.75 + 0.5)	73
**4**	**Zn**	**30**	**CH_3CN (0.5)**	**dioxane (1.5 + 0.5)**	**77(75)**
5	Zn	30	CH_3CN (1.0)	dioxane (1.0 + 0.5)	70
6	Zn	30	CH_3CN (2.0)	dioxane (0.5)	66
7	Zn	20	CH_3CN (0.25)	dioxane (1.75 + 0.5)	69
8	Zn	25	CH_3CN (0.25)	dioxane (1.75 + 0.5)	74
9	Zn	35	CH_3CN (0.25)	dioxane (1.75 + 0.5)	73
10	Mn	30	CH_3CN (0.25)	dioxane (1.75 + 0.5)	n.d
11	Fe	30	CH_3CN (0.25)	dioxane (1.75 + 0.5)	n.d
12	-	30	CH_3CN (0.25)	dioxane (1.75 + 0.5)	n.d

Yields were determined by ^{19}F NMR with trifluorotoluene as internal standard. Isolated yield in parentheses.

Scope of BzO-DFs (3–5)

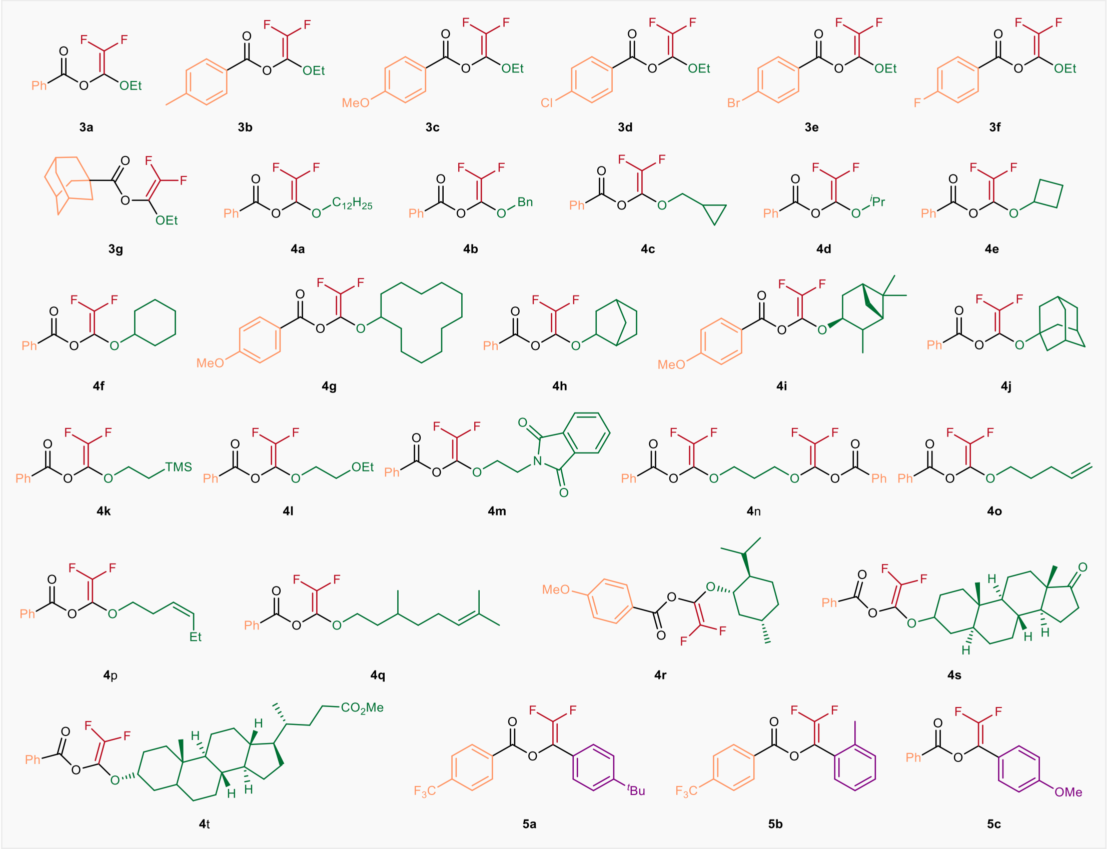

General procedure for preparation of BzO-DFs (3–4)

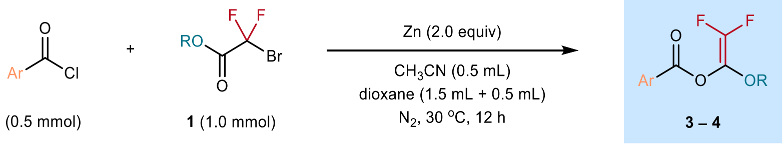

To a 8 mL vial equipped with a magnetic stir bar, acyl chloride (1.0 equiv, 0.5 mmol) and Zn powder (2.0 equiv, 1.0 mmol), CH_3CN (0.5 mL) and dioxane (1.5 mL) were added. The reaction mixture was sealed with a rubber septum and inside glovebox. **1** (2.0 equiv, 1.0 mmol) was dissolved in dioxane (0.5 mL) and added to the “acyl chloride + Zn powder” mixture with a syringe pump over 20 min. Then the reaction mixture was then stirred for 12 hours at 30 ^{o}C. Upon completion, the crude mixture was filtered, and the filtrate was concentrated in *vacuo*. The residue was purified by flash column chromatography (gradient elution: *n*-hexane:ethyl acetate = 200:1~50:1) to give the desired 2,2-difluorovinyl benzoate **3–4** (36–84% yields).

General procedure for preparation of 5a and 5b

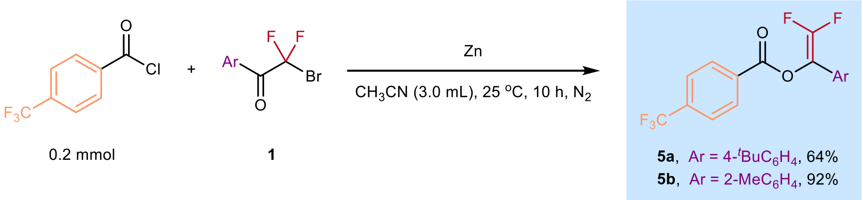

*For the synthesis of **5a**:* To a 8 mL vial equipped with a magnetic stir bar, 4-(trifluoromethyl)benzoyl chloride (1.0 equiv, 0.2 mmol) and Zn powder (3.0 equiv, 0.6 mmol) were mixed in CH_3CN (0.5 mL) in a glovebox. The reaction mixture was then sealed with a rubber septum inside the glovebox. To the mixture, **1v** (3.0 equiv, 0.6 mmol) was added with a syringe pump over 8 h. The reaction was then stirred for 2 hours at room temperature. Upon completion, the reaction mixture was filtered, and the filtrate was concentrated in *vacuo*. The residue was then purified by flash column chromatography (*n*-hexane:ethyl acetate) to give the desired **5a** (64% yield).

*For the synthesis of **5b**:* To a 8 mL vial equipped with a magnetic stir bar, 4-(trifluoromethyl)benzoyl chloride (1.0 equiv, 0.2 mmol) and Zn powder (2.0 equiv, 0.4 mmol) were mixed in CH_3CN (0.5 mL) in a glovebox. The reaction mixture was then sealed with a rubber septum inside the glovebox. To the mixture, **1w** (1.5 equiv, 0.3 mmol) was added with a syringe pump over 8 h. The reaction was then stirred for 2 hours at room temperature. Upon completion, the reaction mixture was filtered, and the filtrate was concentrated in *vacuo*. The residue was purified by flash column chromatography (*n*-hexane:ethyl acetate) to give the desired **5b** (92% yield).

General procedure for preparation of 5c

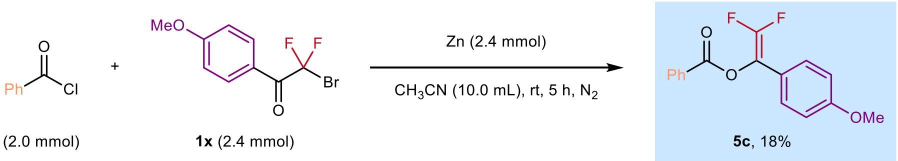

To a 25 mL round-bottom flask equipped with a magnetic stir bar, benzoyl chloride (1.0 equiv, 2.0 mmol), Zn powder (1.2 equiv, 2.4 mmol) were add to CH_3CN (10.0 mL) in a glovebox. The reaction mixture was then sealed with a rubber septum inside the glovebox. To the mixture, **1x** (1.2 equiv, 2.4 mmol) was added with a syringe pump over 10 minutes. Then the reaction mixture was then stirred for 5 hours at room temperature. Upon completion, the reaction mixture was filtered, and the filtrate was concentrated in *vacuo*. The residue was then purified by flash column chromatography (*n*-hexane:ethyl acetate) to give the desired 2,2-difluorovinyl benzoate **5c** (18% yield).

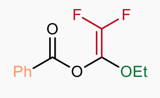

3a

1-Ethoxy-2,2-difluorovinyl benzoate (3a): colorless oil (75%). **^{1}H NMR** (400 MHz, Chloroform-*d*) δ= 8.12 (d, *J* = 7.28 Hz, 2H), 7.65 (t, *J* = 7.44 Hz, 1H), 7.50 (t, *J* = 7.80 Hz, 2H), 4.04 (q, *J* = 7.08 Hz, 2H), 1.34 (t, *J* = 7.08 Hz, 3H) ppm. **^{13}C NMR** (101 MHz, Chloroform-*d*) δ= 163.4, 150.1 (t, *J* = 280.7 Hz), 134.2, 130.4, 128.7, 127.9, 124.0 (t, *J* = 40.0 Hz), 69.0 (m), 14.8 ppm. **^{19}F NMR** (376 MHz, Chloroform-*d*) δ= -116.13 (d, *J* = 81.63 Hz, 1F), -117.15 (d, *J* = 81.59 Hz, 1F) ppm. **HRMS** (ESI): calcd. For $C_{11}H_{10}F_2NaO_3^+$: 251.0488. found: 251.0490.

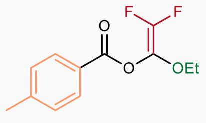

3b

1-Ethoxy-2,2-difluorovinyl 4-methylbenzoate (3b): colorless oil (82%). **^{1}H NMR** (400 MHz, Chloroform-*d*) δ= 8.00 (d, *J* = 8.16 Hz, 2H), 7.29 (d, *J* = 8.04 Hz, 2H), 4.03 (q, *J* = 7.08 Hz, 2H), 2.44 (s, 3H), 1.33 (t, *J* = 7.04 Hz, 3H) ppm. **^{13}C NMR** (101 MHz, Chloroform-*d*) δ= 163.4 (m), 150.1 (t, *J* = 280.6 Hz), 145.3, 130.5, 129.4, 125.1, 124.0 (t, *J* = 40.0 Hz), 68.9, 21.8, 14.8 ppm. **^{19}F NMR** (376 MHz, Chloroform-*d*) δ= -116.40 (d, *J* = 81.86 Hz, 1F), -117.33 (d, *J* = 81.78 Hz, 1F) ppm. **HRMS** (ESI): calcd. for $C_{12}H_{12}F_2NaO_3^+$: 265.0647. found: 265.0650.

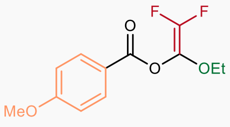

3c

1-Ethoxy-2,2-difluorovinyl 4-methoxybenzoate (3c): colorless oil (72%). **^{1}H NMR** (400 MHz, Chloroform-*d*) δ= 8.07 (d, *J* = 8.76 Hz, 2H), 6.96 (d, *J* = 8.80 Hz, 2H), 4.03 (q, *J* = 7.08 Hz, 2H), 3.89 (s, 3H), 1.33 (t, *J* = 7.04 Hz, 3H) ppm. **^{13}C NMR** (101 MHz, Chloroform-*d*) δ= 164.5, 163.0, 150.2 (t, *J* = 279.7 Hz), 132.5, 123.9 (t, *J* = 40.4 Hz), 119.9, 114.0, 68.7, 55.3, 14.6 ppm. **^{19}F NMR** (376 MHz, Chloroform-*d*) δ= -116.54 (d, *J* = 82.23 Hz, 1F), -117.38 (d, *J* = 82.04 Hz, 1F) ppm. **HRMS** (ESI): calcd. for $C_{12}H_{12}F_2NaO_4^+$: 281.0596. found: 281.0596.

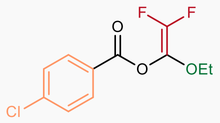

3d

1-Ethoxy-2,2-difluorovinyl 4-chlorobenzoate (3d): colorless oil (64%). **^{1}H NMR** (400 MHz, Chloroform-*d*) δ= 8.05 (d, *J* = 8.52 Hz, 2H), 7.48 (d, *J* = 8.56 Hz, 2H), 4.04 (q, *J* = 7.08 Hz, 2H), 1.33 (t, *J* = 7.08 Hz, 3H) ppm. **^{13}C NMR** (101 MHz, Chloroform-*d*) δ= 162.6 (m), 150.0 (t, *J* = 280.2 Hz), 140.9, 131.8, 129.1, 126.3, 124.0 (t, *J* = 39.8 Hz), 69.2 (t, *J* = 3.0 Hz), 14.8 ppm. **^{19}F NMR** (376 MHz,

Chloroform-*d*) δ= -115.85 (d, *J* = 80.73 Hz, 1F), -116.93 (d, *J* = 81.18 Hz, 1F) ppm. **HRMS** (ESI): calcd. for $C_{11}H_9ClF_2NaO_3^+$: 285.0100. found: 285.0103.

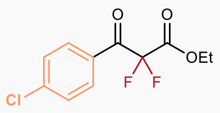

3d'

Ethyl 3-(4-chlorophenyl)-2,2-difluoro-3-oxopropanoate (3d′): colorless oil (3%). **^{1}H NMR** (400 MHz, Chloroform-*d*) δ= 8.05 (d, *J* = 8.52 Hz, 2H), 7.48 (d, *J* = 8.56 Hz, 2H), 4.04 (q, *J* = 7.08 Hz, 2H), 1.33 (t, *J* = 7.08 Hz, 3H) ppm. **^{13}C NMR** (101 MHz, Chloroform-*d*) δ= 162.6 (m), 150.0 (t, *J* = 280.2 Hz), 140.9, 131.8, 129.1, 126.3, 124.0 (t, *J* = 39.8 Hz), 69.2 (t, *J* = 3.0 Hz), 14.8 ppm. **^{19}F NMR** (376 MHz, Chloroform-*d*) δ= -115.85 (d, *J* = 80.73 Hz, 1F), -116.93 (d, *J* = 81.18 Hz, 1F) ppm. **HRMS** (EI): calcd. for $C_{11}H_9ClF_2O_3$: 262.0208. found: 262.0211.

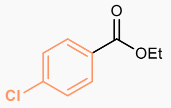

3d''

Ethyl 4-chlorobenzoate (3d″): colorless oil (15%). **^{1}H NMR** (400 MHz, Chloroform-*d*) δ= 7.96 (d, *J* = 8.23 Hz, 2H), 7.40 (d, *J* = 8.29 Hz, 2H), 4.36 (q, *J* = 7.26 Hz, 2H), 1.38 (t, *J* = 7.31 Hz, 3H) ppm. **^{13}C NMR** (101 MHz, Chloroform-*d*) δ= 165.7, 139.2, 130.9, 128.9, 128.6, 61.2, 14.3 ppm. **HRMS** (ESI): calcd. for $C_9H_{10}ClO_2^+$: 185.0364. found: 185.0368.

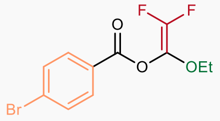

3e

1-Ethoxy-2,2-difluorovinyl 4-bromobenzoate (3e): colorless oil (45%). **^{1}H NMR** (400 MHz, Chloroform-*d*) δ= 7.97 (d, *J* = 8.48 Hz, 2H), 7.64 (d, *J* = 8.48 Hz, 2H), 4.04 (q, *J* = 7.12 Hz, 2H), 1.33 (t, *J* = 7.04 Hz, 3H) ppm. **^{13}C NMR** (101 MHz, Chloroform-*d*) δ= 162.8, 150.0 (t, *J* = 280.9 Hz), 132.1, 131.8, 129.7, 126.8, 124.0 (t, *J* = 41.1 Hz), 69.2, 14.8 ppm. **^{19}F NMR** (376 MHz, Chloroform-*d*) δ= -115.79 (d, *J* = 81.14 Hz, 1F), -116.88 (d, *J* = 81.37 Hz, 1F) ppm. **HRMS** (ESI): calcd. for $C_{11}H_9BrF_2NaO_3^+$: 328.9595. found: 328.9595.

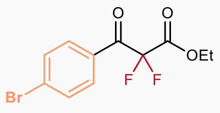

3e'

Ethyl 3-(4-bromophenyl)-2,2-difluoro-3-oxopropanoate (3e′): colorless oil (4%). **^{1}H NMR** (400 MHz, Chloroform-*d*) δ= 7.93 (d, *J* = 8.78 Hz, 2H), 7.58 (d, *J* = 8.52 Hz, 2H), 4.41 (q, *J* = 7.15 Hz, 2H), 1.36 (t, *J* = 7.18 Hz, 3H) ppm.

^{13}**C NMR** (101 MHz, Chloroform-*d*) δ= 161.6, 159.5, 159.1, 132.3, 131.8, 130.5, 125.7, 113.5 (t, *J* = 270.0 Hz), 64.0, 13.7 ppm. ^{19}**F NMR** (376 MHz, Chloroform-*d*) δ= -82.6 ppm. **HRMS** (EI): calcd. for $C_{11}H_9BrF_2O_3$: 305.9703. found: 305.9691.

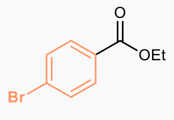

3e''

Ethyl 4-bromobenzoate (3e''): colorless oil (18%). ^{1}**H NMR** (400 MHz, Chloroform-*d*) δ= 7.89 (d, *J* = 8.45 Hz, 2H), 7.56 (d, *J* = 8.45 Hz, 2H), 4.36 (q, *J* = 7.06 Hz, 2H), 1.38 (t, *J* = 7.15 Hz, 3H) ppm. ^{13}**C NMR** (101 MHz, Chloroform-*d*) δ= 165.9, 131.6, 131.1, 129.4, 127.9, 61.2, 14.3 ppm. **HRMS** (ESI): calcd. for $C_9H_{10}BrO_2^+$: 228.9859. found: 228.9857.

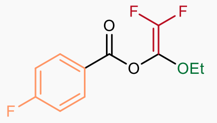

3f

1-Ethoxy-2,2-difluorovinyl 4-fluorobenzoate (3f): colorless oil (66%). ^{1}**H NMR** (400 MHz, Chloroform-*d*) δ= 8.14 (m, 2H), 7.17 (t, *J* = 8.56 Hz, 2H), 4.04 (q, *J* = 7.04 Hz, 2H), 1.33 (t, *J* = 7.08 Hz, 3H) ppm. ^{13}**C NMR** (101 MHz, Chloroform-*d*) δ= 167.8, 165.2, 162.4 (m), 150.1 (t, *J* = 280.1 Hz), 133.1 (d, *J* = 9.7 Hz), 124.1 (d, *J* = 2.9 Hz), 124.0 (t, *J* = 40.4 Hz), 116.0 (d, *J* = 22.2 Hz), 69.1 (t, *J* = 3.0 Hz), 14.7 ppm. ^{19}**F NMR** (376 MHz, Chloroform-*d*) δ= -103.05 (s, 1F), -116.08 (d, *J* = 81.40 Hz, 1F), -117.12 (d, *J* = 81.25 Hz, 1F) ppm. **HRMS** (ESI): calcd. for $C_{11}H_{13}F_3NO_3^+$: 264.0842. found: 264.0849.

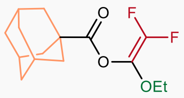

3g

1-Ethoxy-2,2-difluorovinyl (3r,5r,7r)-adamantane-1-carboxylate (3g): colorless oil (78%). ^{1}**H NMR** (400 MHz, Chloroform-*d*) δ= 3.93 (q, *J* = 7.12 Hz, 2H), 2.06 (s, 3H), 1.97 (m, 6H), 1.74 (m, 6H), 1.29 (t, *J* = 7.12 Hz, 3H) ppm. ^{13}**C NMR** (101 MHz, Chloroform-*d*) δ= 174.3, 149.8 (t, *J* = 279.6 Hz), 123.8 (t, *J* = 39.6 Hz), 68.5, 40.9, 38.4, 36.3, 27.7, 14.8 ppm. ^{19}**F NMR** (376 MHz, Chloroform-*d*) δ= -117.36 (d, *J* = 83.70 Hz, 1F), -118.02 (d, *J* = 83.28 Hz, 1F) ppm. **HRMS** (ESI): calcd. for $C_{15}H_{20}F_2NaO_3^+$: 309.1273. found: 309.1265.

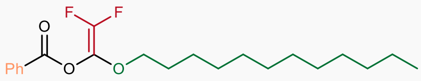

4a

1-(Dodecyloxy)-2,2-difluorovinyl benzoate (4a): colorless oil (84%). ^{1}**H NMR** (400 MHz, Chloroform-*d*) δ= 8.12 (d, *J* = 7.48 Hz, 2H), 7.64 (t, *J* = 7.44 Hz, 1H), 7.49 (t,

J = 7.76 Hz, 2H), 3.97 (t, J = 6.60 Hz, 2H), 1.68 (m, 2H), 1.38-1.24 (m, 21H) ppm. **^{13}C NMR** (101 MHz, Chloroform-*d*) δ= 163.4 (m), 149.9 (t, J = 279.9 Hz), 134.2, 130.4, 128.7, 127.9, 124.4 (t, J = 40.3 Hz), 73.5 (m), 31.9, 29.6, 29.5, 29.5, 29.4, 29.3, 29.2, 25.6, 22.7, 14.1 ppm. **^{19}F NMR** (376 MHz, Chloroform-*d*) δ= -116.42 (d, J = 81.86 Hz, 1F), -117.21 (d, J = 81.82 Hz, 1F) ppm. **HRMS** (EI): calcd. for $C_{21}H_{30}F_2O_3$: 368.2163. found: 368.2180.

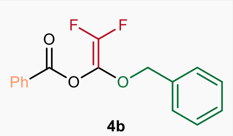

4b

1-(Benzyloxy)-2,2-difluorovinyl benzoate (4b): colorless oil (71%). **^{1}H NMR** (400 MHz, Chloroform-*d*) δ= 8.07 (d, J = 7.28 Hz, 2H), 7.62 (t, J = 7.40 Hz, 1H), 7.47 (t, J = 7.80 Hz, 2H), 7.39-7.30 (m, 5H), 4.99 (s, 2H) ppm. **^{13}C NMR** (101 MHz, Chloroform-*d*) δ= 163.4, 150.2 (t, J = 280.9 Hz), 135.4, 134.3, 130.5, 128.73, 128.68, 128.6, 128.5, 127.8, 124.2 (t, J = 40.4 Hz), 75.0 ppm. **^{19}F NMR** (376 MHz, Chloroform-*d*) δ= -115.59 (d, J = 79.25 Hz, 1F), -116.03 (d, J = 79.94 Hz, 1F) ppm. **HRMS** (ESI): calcd. for $C_{16}H_{12}F_2O_3Na^+$: 313.0647. found: 313.0649.

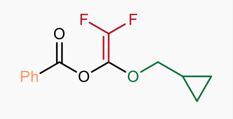

4c

1-(Cyclopropylmethoxy)-2,2-difluorovinyl benzoate (4c): colorless oil (52%). **^{1}H NMR** (400 MHz, Chloroform-*d*) δ= 8.11 (d, J = 7.20 Hz, 2H), 7.65 (t, J = 7.48 Hz, 1H), 7.50 (t, J = 7.88 Hz, 2H), 3.81 (d, J = 7.32 Hz, 2H), 1.20 (m, 1H), 0.61 (m, 2H), 0.31 (m, 2H) ppm. **^{13}C NMR** (101 MHz, Chloroform-*d*) δ= 163.4, 150.0 (t, J = 280.5 Hz), 134.2, 130.4, 128.7, 127.9, 124.2 (t, J = 40.4 Hz), 78.2 (m), 10.2, 3.2 ppm. **^{19}F NMR** (376 MHz, Chloroform-*d*) δ= -116.08 (d, J = 80.46 Hz, 1F), -116.93 (d, J = 81.59 Hz, 1F) ppm. **HRMS** (ESI): calcd. for $C_{13}H_{13}F_2O_3^+$: 255.0827. found: 255.0829.

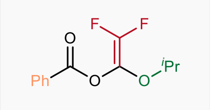

4d

2,2-Difluoro-1-isopropoxyvinyl benzoate (4d): colorless oil (45%). **^{1}H NMR** (400 MHz, Chloroform-*d*) δ= 8.18 (d, J = 7.24 Hz, 2H), 7.64 (t, J = 7.46 Hz, 1H), 7.49 (t, J = 7.76 Hz, 2H), 4.31-4.22 (m, 1H), 1.32 (d, J = 6.24 Hz, 6H) ppm. **^{13}C NMR** (101 MHz, Chloroform-*d*) δ= 163.2, 150.6 (t, J = 280.2 Hz), 134.2, 130.4, 128.7, 128.0, 122.6 (t, J = 40.3 Hz), 75.9 (t, J = 2.6 Hz), 22.0 ppm. **^{19}F NMR** (376 MHz, Chloroform-*d*) δ= -115.66 (d, J =

85.2 Hz, 1F), -117.30 (d, *J* = 85.3 Hz, 1F) ppm. **HRMS** (ESI): calcd. for $C_{12}H_{13}F_2O_3^+$: 243.0827. found: 243.0828.

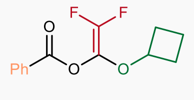

4e

1-Cyclobutoxy-2,2-difluorovinyl benzoate (4e): colorless oil (62%). **^{1}H NMR** (400 MHz, Chloroform-*d*) δ= 8.11 (d, *J* = 7.16 Hz, 2H), 7.64 (t, *J* = 7.40 Hz, 1H), 7.49 (t, *J* = 7.80 Hz, 2H), 4.50 (m, 1H), 2.32-2.24 (m, 2H), 2.22-2.12 (m, 2H), 1.79-1.71 (m, 1H), 1.57-1.45 (m, 1H) ppm. **^{13}C NMR** (101 MHz, Chloroform-*d*) δ= 163.2 (m), 150.3 (t, *J* = 279.9 Hz), 134.2, 130.4, 128.7, 127.9, 122.9 (t, *J* = 40.0 Hz), 76.4 (m), 30.4, 12.3 ppm. **^{19}F NMR** (376 MHz, Chloroform-*d*) δ= -116.22 (d, *J* = 80.88 Hz, 1F), -117.22 (d, *J* = 81.48 Hz, 1F) ppm. **HRMS** (EI): calcd. for $C_{13}H_{12}F_2O_3$: 254.0755. found: 254.0765.

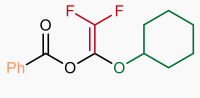

4f

1-(Cyclohexyloxy)-2,2-difluorovinyl benzoate (4f): colorless oil (83%). **^{1}H NMR** (400 MHz, Chloroform-*d*) δ= 8.12 (d, *J* = 7.2 Hz, 2H), 7.64 (t, *J* = 7.48 Hz, 1H), 7.50 (m, 2H), 4.00-3.94 (m, 1H), 1.96-1.94 (m, 2H), 1.77-1.75 (m, 2H), 1.57-1.49 (m, 3H), 1.34-1.24 (m, 3H) ppm. **^{13}C NMR** (101 MHz, Chloroform-*d*) δ= 163.2, 150.5 (t, *J* = 280.0 Hz), 134.2, 130.4, 128.7, 128.0, 122.5 (t, *J* = 40.4 Hz), 80.9, 31.8, 25.3, 23.5 ppm. **^{19}F NMR** (376 MHz, Chloroform-*d*) δ= -115.63 (d, *J* = 80.24 Hz, 1F), -117.00 (d, *J* = 80.28 Hz, 1F) ppm. **HRMS** (ESI): calcd. for $C_{15}H_{16}F_2NaO_3^+$: 305.0960. found: 305.0962.

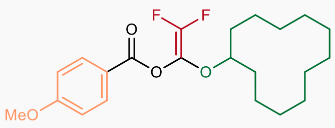

4g

1-(Cyclododecyloxy)-2,2-difluorovinyl 4-methoxybenzoate (4g): colorless oil (78%). **^{1}H NMR** (400 MHz, Chloroform-*d*) δ= 7.37 (d, *J* = 8.12 Hz, 2H), 6.92 (d, *J* = 8.72 Hz, 2H), 3.83 (s, 3H), 3.75 (m, 1H), 1.58-1.50 (m, 2H), 1.46-1.18 (m, 20H) ppm. **^{13}C NMR** (101 MHz, Chloroform-*d*) δ= 1594, 154.9 (t, *J* = 289.5 Hz), 128.5 (m), 123.1 (d, *J* = 5.8 Hz), 116.0 (dd, *J* = 66.0, 17.7 Hz), 113.9, 77.6, 55.3, 28.9, 24.5, 24.0, 23.3, 23.0, 20.6 ppm. **^{19}F NMR** (376 MHz, Chloroform-*d*) δ= -116.41 (d, *J* = 81.74 Hz, 1F), -117.57 (d, *J* = 81.67 Hz, 1F) ppm. **HRMS** (EI): calcd. for $C_{22}H_{30}F_2O_4$: 396.2112. found: 396.2107.

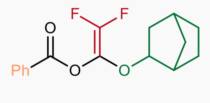

4h

1-(((1S,4R)-bicyclo[2.2.1]heptan-2-yl)oxy)-2,2-difluorovinyl benzoate (4h): colorless oil (70%). **^{1}H NMR** (400 MHz, Chloroform-*d*) δ= 8.13 (d, *J* = 7.24 Hz, 2H), 7.65 (t, *J* = 7.44 Hz, 1H), 7.50 (t, *J* = 7.84 Hz, 2H), 4.02 (m, 1H), 2.49 (d, *J* = 4.84 Hz, 1H), 2.30 (s, 1H), 1.62-1.51 (m, 4H), 1.47-1.40 (m, 1H), 1.15 (d, *J* = 9.84 Hz, 1H), 1.08-0.96 (m, 2H) ppm. **^{13}C NMR** (101 MHz, Chloroform-*d*) δ= 163.2, 155.6 (t, *J* = 279.86 Hz), 134.2, 130.4, 128.7, 128.0, 122.8 (t, *J* = 40.1 Hz), 85.3, 41.2, 38.3, 35.4, 34.7, 28.2, 23.9 ppm. **^{19}F NMR** (376 MHz, Chloroform-*d*) δ= -116.11 (d, *J* = 80.54 Hz, 1F), -117.41 (d, *J* = 80.95 Hz, 1F) ppm. **HRMS** (ESI): calcd. for $C_{16}H_{16}F_2NaO_3^+$: 317.0960. found: 317.0960.

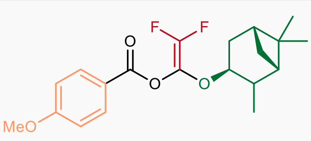

4i

2,2-Difluoro-1-(((1S,3S,5R)-2,6,6-trimethylbicyclo[3.1.1]heptan-3-yl)oxy)vinyl 4-methoxybenzoate (4i): colorless oil (74%). **^{1}H NMR** (400 MHz, Chloroform-*d*) δ= 8.08 (d, *J* = 8.80 Hz, 2H), 6.97 (d, *J* = 8.80 Hz, 2H), 3.88 (s, 3H), 3.52 (s, 1H), 1.84-1.77 (m, 1H), 1.70 (m, 1H), 1.47-1.37 (m, 2H), 1.10 (m, 6H), 0.96 (s, 3H), 0.88 (s, 3H) ppm. **^{13}C NMR** (101 MHz, Chloroform-*d*) δ= 164.4, 162.6, 149.4 (t, *J* = 279.2 Hz), 132.6, 124.9 (t, *J* = 40.1 Hz), 120.2, 114.1, 96.7, 55.5, 49.2, 48.1, 41.2, 39.5, 30.3, 25.9, 25.6, 20.8, 19.3 ppm. **^{19}F NMR** (376 MHz, Chloroform-*d*) δ= -116.37 (d, *J* = 89.64 Hz, 1F), -117.73 (d, *J* = 82.19 Hz, 1F) ppm. **HRMS** (EI): calcd. for $C_{20}H_{24}F_2O_4$: 366.1643. found: 366.1652.

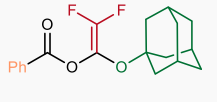

4j

1-(((3s,5s,7s)-adamantan-1-yl)oxy)-2,2-difluorovinyl benzoate (4j): colorless oil (62%). **^{1}H NMR** (400 MHz, Chloroform-*d*) δ= 8.10 (d, *J* = 7.20 Hz, 2H), 7.64 (t, *J* = 7.46 Hz, 1H), 7.49 (t, *J* = 7.72 Hz, 2H), 2.20 (s, 3H), 1.92 (s, 6H), 1.63 (s, 6H) ppm. **^{13}C NMR** (101 MHz, Chloroform-*d*) δ= 163.0 (m), 151.7 (m), 134.0, 130.3, 128.7, 128.4, 120.0 (t, *J* = 40.4 Hz), 82.7 (m), 42.2, 35.9, 31.0 ppm. **^{19}F NMR** (376 MHz, Chloroform-*d*) δ= -113.23 (d, *J* = 75.35 Hz, 1F), -114.79 (d, *J* = 75.31 Hz, 1F) ppm. **HRMS** (ESI): calcd. for $C_{19}H_{20}F_2NaO_3^+$: 357.1273. found: 357.1274.

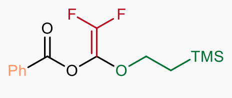

4k

2,2-Difluoro-1-(2-(trimethylsilyl)ethoxy)vinyl benzoate (4k): colorless oil (72%). **^{1}H NMR** (400 MHz, Chloroform-*d*) δ= 8.12 (d, *J* = 7.24 Hz, 2H), 7.65 (t, *J* = 7.48 Hz, 1H), 7.50 (t, *J* = 7.80 Hz, 2H), 4.06 (m, 2H), 1.10 (m, 2H), 0.03 (s, 9H) ppm. **^{13}C NMR** (101 MHz, Chloroform-*d*) δ= 163.4, 150.2 (t, *J* = 279.4 Hz), 134.2, 130.4, 128.7, 128.0, 123.8 (t, *J* = 40.5 Hz), 71.3 (t, *J* = 2.9 Hz), 18.0, -1.5 ppm. **^{19}F NMR** (376 MHz, Chloroform-*d*) δ= -116.11 (d, *J* = 81.93 Hz, 1F), -117.27 (d, *J* = 81.70 Hz, 1F) ppm. **HRMS** (ESI): calcd. for $C_{14}H_{19}F_2O_3Si^+$: 301.1066. found: 301.1063.

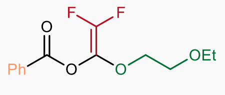

4l

1-(2-Ethoxyethoxy)-2,2-difluorovinyl benzoate (4l): colorless oil (81%). **^{1}H NMR** (400 MHz, Chloroform-*d*) δ= 8.11 (d, *J* = 7.40 Hz, 2H), 7.63 (t, *J* = 7.48 Hz, 1H), 7.48 (t, *J* = 7.84 Hz, 2H), 4.14 (m, 2H), 3.66 (t, *J* = 4.72 Hz, 2H), 3.51 (q, *J* = 6.96 Hz, 2H), 1.15 (t, *J* = 7.00 Hz, 3H) ppm. **^{13}C NMR** (101 MHz, Chloroform-*d*) δ= 163.3, 149.9 (t, *J* = 280.5 Hz), 134.3, 130.4, 128.7, 127.8, 124.4 (t, *J* = 40.4 Hz), 72.5 (m), 68.7, 66.7, 14.9 ppm. **^{19}F NMR** (376 MHz, Chloroform-*d*) δ= -116.19 (d, *J* = 81.14 Hz, 1F), -116.58 (d, *J* = 80.54 Hz, 1F) ppm. **HRMS** (ESI): calcd. for $C_{13}H_{14}F_2NaO_4^+$: 295.0752. found: 295.0756.

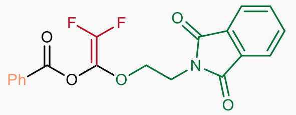

4m

1-(2-(1,3-Dioxoisoindolin-2-yl)ethoxy)-2,2-difluorovinyl benzoate (4m): colorless oil (36%). **^{1}H NMR** (400 MHz, Chloroform-*d*) δ= 7.93 (d, *J* = 7.20 Hz, 2H), 7.76 (dd, *J* = 5.40, 3.04 Hz, 2H), 7.66 (dd, *J* = 5.40, 3.04 Hz, 2H), 7.60 (t, *J* = 7.52 Hz, 1H), 7.41 (t, *J* = 7.92 Hz, 2H), 4.30 (t, *J* = 5.48 Hz, 2H), 4.00 (t, *J* = 5.48 Hz, 2H) ppm. **^{13}C NMR** (101 MHz, Chloroform-*d*) δ= 168.0, 163.3, 149.7 (t, *J* = 281.0 Hz), 134.2, 133.9, 131.9, 130.4, 128.6, 127.4, 124.6 (t, *J* = 40.5 Hz), 123.3, 70.4, 37.5 ppm. **^{19}F NMR** (376 MHz, Chloroform-*d*) δ= -115.73 (d, *J* = 10.75 Hz, 2F) ppm. **HRMS** (ESI): calcd. for $C_{19}H_{13}F_2NNaO_5^+$: 396.0659 found: 396.0650.

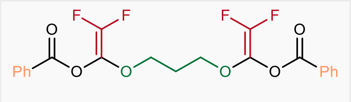

(Propane-1,3-diylbis(oxy))bis(2,2-difluoroethene-1,1-diyl) dibenzoate (4n): colorless oil (54%). **^{1}H NMR** (400 MHz, Chloroform-*d*) δ= 8.09 (d, *J* = 7.40 Hz, 4H), 7.64 (t, *J* = 7.40 Hz, 2H), 7.49 (t, *J* = 7.76 Hz, 4H), 4.11 (t, *J* = 6.08 Hz, 4H), 2.07 (m, 2H) ppm. **^{13}C NMR** (101 MHz, Chloroform-*d*) δ= 163.3, 149.9 (t, *J* = 281.0 Hz), 134.3, 130.5, 128.7, 127.7, 124.2 (t, *J* = 40.5 Hz), 69.2, 29.2 ppm. **^{19}F NMR** (376 MHz, Chloroform-*d*) δ= -115.86 (d, *J* = 81.18 Hz, 2F), -116.63 (d, *J* = 81.10 Hz, 2F) ppm. **HRMS** (ESI): calcd. for $C_{21}H_{16}F_4O_6Na^+$: 463.0775. found: 463.0778.

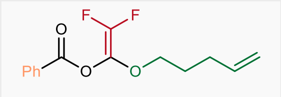

2,2-Difluoro-1-(pent-4-en-1-yloxy)vinyl benzoate (4o): colorless oil (68%). **^{1}H NMR** (400 MHz, Chloroform-*d*) δ= 8.12 (d, *J* = 7.44 Hz, 2H), 7.64 (t, *J* = 7.42 Hz, 1H), 7.49 (t, *J* = 7.74 Hz, 2H), 5.83-5.72 (m, 1H), 5.04-4.99 (m, 1H), 4.97 (d, *J* = 10.2 Hz, 1H), 3.98 (t, *J* = 6.4 Hz, 2H), 2.16 (dd, *J* = 14.28, 7.00 Hz, 2H), 1.78 (m, 2H) ppm. **^{13}C NMR** (101 MHz, Chloroform-*d*) δ= 163.4 (t, *J* = 2.5 Hz), 150.0 (t, *J* = 280.3 Hz), 137.3, 134.3, 130.4, 128.7, 127.8, 124.3 (t, *J* = 40.2 Hz), 115.4, 72.5, 29.6, 28.4 ppm. **^{19}F NMR** (376 MHz, Chloroform-*d*) δ= -116.31 (d, *J* = 81.78 Hz, 1F), -117.10 (d, *J* = 81.78 Hz, 1F) ppm. **HRMS** (ESI): calcd. for $C_{14}H_{15}F_2O_3^+$: 269.0984. found: 269.0987.

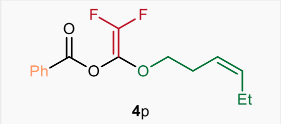

(Z)-2,2-difluoro-1-(hex-3-en-1-yloxy)vinyl benzoate (4p): colorless oil (75%). **^{1}H NMR** (400 MHz, Chloroform-*d*) δ= 8.12 (d, *J* = 7.32 Hz, 2H), 7.65 (m, 1H), 7.50 (t, *J* = 7.78 Hz, 2H), 5.52-5.45 (m, 1H), 5.35-5.29 (m ,1H), 3.96 (t, *J* = 7.04 Hz, 2H), 2.45 (q, *J* = 6.96 Hz, 2H), 2.04 (m, 2H), 0.95 (t, *J* = 7.52 Hz, 3H) ppm. **^{13}C NMR** (101 MHz, Chloroform-*d*) δ= 163.4 (t, *J* = 2.5 Hz), 149.9 (t, *J* = 280.4 Hz), 134.8, 134.3, 130.5, 128.7, 127.8, 124.3 (t, *J* = 40.4 Hz), 123.1, 72.8, 27.4, 20.6, 14.1 ppm. **^{19}F NMR** (376 MHz, Chloroform-*d*) δ= -116.20 (d, *J* = 81.48 Hz, 1F), -116.84 (d, *J* = 81.40 Hz, 1F) ppm. **HRMS** (ESI): calcd. for $C_{15}H_{16}F_2NaO_3^+$: 305.0960. found: 305.0964.

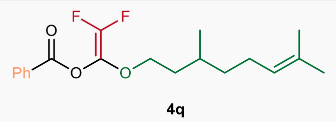

4q

1-((3,7-Dimethyloct-6-en-1-yl)oxy)-2,2-difluorovinyl benzoate (4q): colorless oil (71%). **^{1}H NMR** (400 MHz, Chloroform-*d*) δ= 8.12 (d, *J* = 7.60 Hz, 2H), 7.65 (t, *J* = 7.36 Hz, 1H), 7.50 (t, *J* = 7.72 Hz, 2H), 5.06 (t, *J* = 6.88 Hz, 1H), 4.01 (m, 2H), 2.05-1.88 (m, 2H), 1.79-1.70 (m, 1H), 1.67 (s, 3H), 1.62 (m, 1H), 1.58 (s, 3H), 1.53-1.45 (m, 1H), 1.38-1.30 (m, 1H), 1.21-1.12 (m, 1H), 0.90 (d, *J* = 6.52 Hz, 3H) ppm. **^{13}C NMR** (101 MHz, Chloroform-*d*) δ= 163.4, 149.9 (t, *J* = 280.5 Hz), 134.2, 131.3, 130.4, 128.7, 127.9, 124.5, 124.3 (t, *J* = 40.4 Hz), 71.8 (m), 37.0, 36.2, 29.1, 25.7, 25.4, 19.3, 17.6 ppm. **^{19}F NMR** (376 MHz, Chloroform-*d*) δ= -116.34 (d, *J* = 81.89 Hz, 1F), -117.10 (d, *J* = 81.78 Hz, 1F) ppm. **HRMS** (ESI): calcd. for $C_{19}H_{24}F_2NaO_3^+$: 361.1586. found: 361.1584.

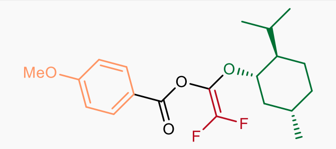

4r

2,2-Difluoro-1-(((1S,2R,5S)-2-isopropyl-5-methylcyclohexyl)oxy)vinyl 4-methoxybenzoate (4r): colorless oil (57%). **^{1}H NMR** (400 MHz, Chloroform-*d*) δ= 7.37 (d, *J* = 8.28 Hz, 2H), 6.91 (d, *J* = 7.40 Hz, 2H), 3.82 (s, 3H), 3.38 (m, 1H), 2.44 (m, 1H), 1.97 (d, *J* = 11.68 Hz, 1H), 1.65-1.58 (m, 2H), 1.42 (m, 1H), 1.23 (m, 2H), 0.96-0.88 (m, 8H), 0.72 (*J* = 6.64 Hz, 3H) ppm. **^{13}C NMR** (101 MHz, Chloroform-*d*) δ= 159.5, 155.0 (t, *J* = 288.1 Hz), 128.8 (m), 123.1 (m), 115.3 (dd, *J* = 35.3, 17.7 Hz), 113.8, 78.3, 55.3, 48.2, 39.9, 34.3, 31.2, 25.1, 22.8, 22.2, 21.2, 15.9 ppm. **^{19}F NMR** (376 MHz, Chloroform-*d*) δ= -100.71 (d, *J* = 64.11 Hz, 1F), -110.94 (d, *J* = 64.15 Hz, 1F) ppm. **HRMS** (EI): calcd. for $C_{20}H_{26}F_2O_4$: 368.1799. found: 368.1814.

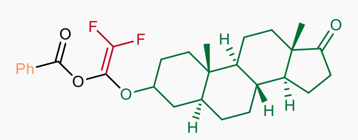

4s

1-(((5S,8R,9S,10S,13S,14S)-10,13-dimethyl-17-oxohexadecahydro-1H-cyclopenta[a]phenanthren-3-yl)oxy)-2,2-difluorovinyl benzoate (4s): white solid, melting point: 77 ºC (55%). **^{1}H NMR** (400 MHz, Chloroform-*d*) δ= 8.11 (d, *J* = 7.30 Hz, 2H), 7.65 (t, *J* = 7.56 Hz, 1H), 7.50 (t, *J* = 7.81 Hz, 2H), 4.24 (s, 1H), 2.43 (m, 1H), 2.06 (m, 1H), 1.92 (m, 2H), 1.78 (m, 2H), 1.67-1.58 (m, 4H), 1.49 (m, 4H), 1.31-1.18 (m, 6H), 1.04-0.93 (m, 1H), 0.85 (s, 3H), 0.83 (m, 1H), 0.81 (s, 3H) ppm. **^{13}C NMR** (101 MHz, Chloroform-*d*) δ= 163.1, 150.5 (t, *J* =

281.6 Hz), 134.2, 130.4, 128.7, 127.9, 122.5 (t, *J* = 40.6 Hz), 77.7, 54.2, 51.4, 47.8, 39.3, 35.9, 35.8, 35.0, 32.8, 32.3, 31.5, 30.7, 28.0, 25.8, 21.7, 20.0, 13.8, 11.3 ppm. **^{19}F NMR** (376 MHz, Chloroform-*d*) δ= -115.45 (d, *J* = 80.61 Hz, 1F), -117.30 (d, *J* = 80.58 Hz, 1F) ppm. **HRMS** (ESI): calcd. for $C_{28}H_{34}F_2NaO_4^+$: 495.2317. found: 495.2323.

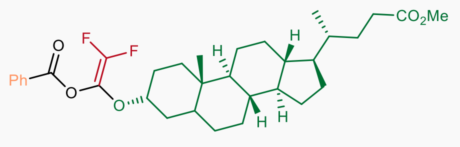

4t

2,2-Difluoro-1-(((8R,9S,10S,13R,14S,17R)-17-((R)-5-methoxy-5-oxopentan-2-yl)-10,13-di-methylhexadecahydro-1H-cyclo-penta[a]phenanthren-3-yl)oxy)vinyl benzoate (4t): colorless oil (73%). **^{1}H NMR** (400 MHz, Chloroform-*d*) δ= 8.12 (d, *J* = 7.24 Hz, 2H), 7.65 (t, *J* = 7.44 Hz, 1H), 7.50 (m, 2H), 3.96-3.91 (m, 1H), 3.66 (s, 3H), 2.39-2.31 (m, 1H), 2.26-2.18 (m, 1H), 1.96-1.82 (m, 6H), 1.68-1.48 (m, 4H), 1.39-1.05 (m, 15H), 0.91 (m, 6H), 0.63 (s, 3H) ppm. **^{13}C NMR** (101 MHz, Chloroform-*d*) δ= 174.8, 163.2, 150.5 (t, *J* = 279.8 Hz), 134.2, 130.4, 128.7, 128.0, 122.6 (t, *J* = 40.4 Hz), 82.9, 56.3, 55.9, 51.5, 42.7, 42.1, 40.4, 40.1, 35.8, 35.4, 35.0, 34.6, 32.8, 31.1, 31.0, 28.2, 27.1, 26.3, 24.2, 23.2, 20.8, 18.3, 12.0 ppm. **^{19}F NMR** (376 MHz, Chloroform-*d*) δ= -115.49 (d, *J* = 79.98 Hz, 1F), -116.86 (d, *J* = 79.9 Hz, 1F) ppm. **HRMS** (ESI): calcd. for $C_{34}H_{46}F_2O_5Na^+$: 595.3206. found: 595.3202.

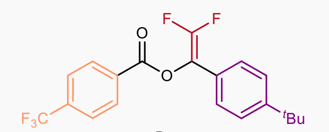

5a

1-(4-(tert-butyl)phenyl)-2,2-difluorovinyl 4-(trifluoromethyl)benzoate (5a): white solid, melting point: 38 °C (64%). **^{1}H NMR** (400 MHz, Chloroform-*d*) δ= 8.31 (d, *J* = 8.20 Hz, 2H), 7.79 (d, *J* = 8.20 Hz, 2H), 7.41 (m, 4H), 1.31 (s, 9H) ppm. **^{13}C NMR** (101 MHz, Chloroform-*d*) δ= 162.8, 154.2 (t, *J* = 290.5 Hz), 151.9, 135.5 (q, *J* = 32.9 Hz), 131.7, 130.7, 125.8, 125.7, 125.3 (dd, *J* = 6.1, 3.6 Hz), 122.1, 112.1 (dd, *J* = 39.1, 19.6 Hz), 34.7, 31.1 ppm. **^{19}F NMR** (376 MHz, Chloroform-*d*) δ= -63.27 (s, 3F), -92.61 (d, *J* = 49.52 Hz, 1F), -103.30 (d, *J* = 48.64 Hz, 1F) ppm. **HRMS** (ESI): calcd. for $C_{20}H_{17}F_5O_2Na^+$: 407.1041. found: 407.1044.

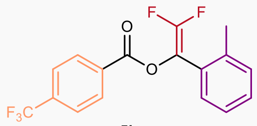

5b

2,2-difluoro-1-(o-tolyl)vinyl 4-(trifluoromethyl)benzoate (5b): colorless oil (92%). **^{1}H NMR** (400 MHz, Chloroform-*d*) δ= 8.22 (d, *J* = 8.02 Hz, 2H), 7.75 (d, *J* = 8.55 Hz, 2H), 7.49 (d, *J* = 7.48 Hz, 1H), 7.32 (m, 1H), 7.25 (m, 2H), 2.48 (s, 3H) ppm. **^{13}C NMR** (101 MHz, Chloroform-*d*) δ= 162.7, 153.6 (t, *J* = 284.2 Hz), 153.6, 138.0, 135.4, 135.1, 131.8, 130.6, 130.3 (m), 130.0, 128.0, 125.9, 125.7 (q, *J* = 3.6 Hz), 124.8, 122.1, 111.0 (dd, *J* = 46.3, 18.9 Hz), 19.6 (m) ppm. **^{19}F NMR** (376 MHz, Chloroform-*d*) δ= -63.29 (s, 3F), -94.92 (d, *J* = 49.29 Hz, 1F), -104.57 (d, *J* = 50.78 Hz, 1F) ppm. **HRMS** (ESI): calcd. for $C_{17}H_{11}F_5O_2Na^+$: 365.0571. found: 365.0565.

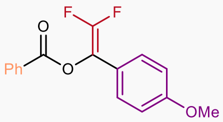

5c

2,2-Difluoro-1-(4-methoxyphenyl)vinyl benzoate (5c): white solid, melting point: 35 °C (18%). **^{1}H NMR** (400 MHz, Chloroform-*d*) δ= 8.18 (d, *J* = 7.32 Hz, 2H), 7.64 (t, *J* = 7.44 Hz, 1H), 7.51 (t, *J* = 7.64 Hz, 2H), 7.40 (d, *J* = 8.56 Hz, 2H), 6.90 (d, *J* = 8.88 Hz, 2H), 3.79 (s, 3H) ppm. **^{13}C NMR** (101 MHz, Chloroform-*d*) δ= 163.9, 159.7, 154.0 (t, *J* = 290.3 Hz), 134.0, 130.3, 128.7, 128.5, 127.1 (dd, *J* = 6.0, 3.6 Hz), 121.6 (d, *J* = 6.3 Hz), 114.2, 111.8 (dd, *J* = 39.7, 19.9 Hz), 55.3 ppm. **^{19}F NMR** (376 MHz, Chloroform-*d*) δ= -94.32 (d, *J* = 52.94 Hz, 1F), -104.94 (d, *J* = 52.83 Hz, 1F) ppm. **HRMS** (EI): calcd. for $C_{16}H_{12}F_2O_3$: 290.0755. found: 290.0774.

Preparation of boronic acid derived from vitamin E[3,4]

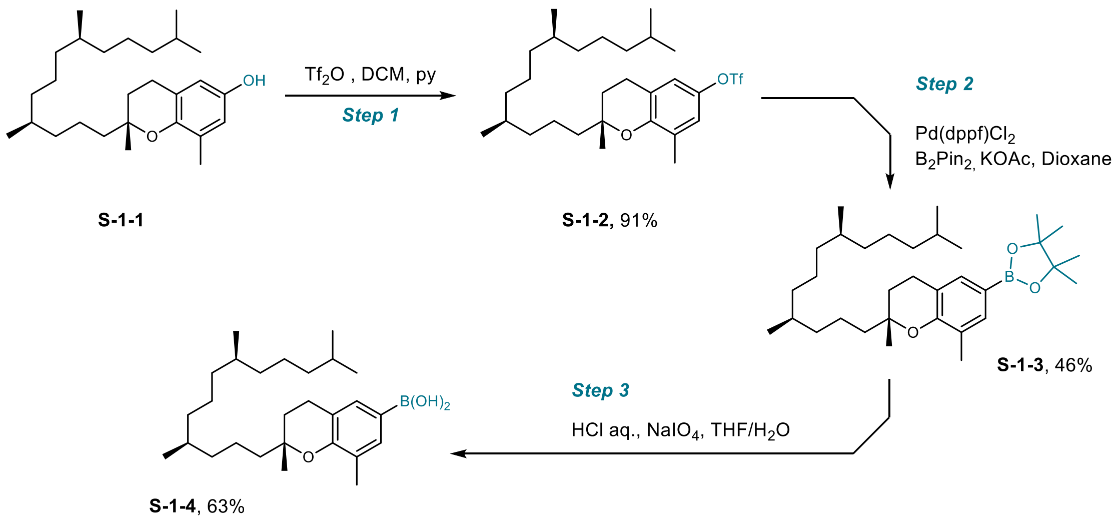

S-1-1, **S-1-2**, 91%, **S-1-3**, 46%, **S-1-4**, 63%

***Step 1*:** To a 100 mL round-bottom flask equipped with a stir bar, D-δ-tocopherol **S-1-1** (12.0 mmol, 1.0 equiv, 4.83 g), dry DCM (60 mL) and pyridine (18.0 mmol, 1.5 equiv, 1.45 mL) were added in a glovebox. The flask was sealed with a septum inside the glovebox. The mixture was then stirred at 0 °C for 5 min and Tf_2O (1.44 mmol, 1.2 equiv, 2.42 mL) was added dropwise to the mixture over 5 min. The reaction was stirred at 0 °C for 2 h, and water (100 mL) was then added to quench the reaction. The reaction mixture was then extracted with DCM (30 mL x 3). The combined organic extracts was dried with anhydrous $MgSO_4$ and concentrated under vacuum. The crude product was then purified with silica gel chromatography to afford the corresponding trifluoromethanesulfonic ester **S-1-2** (91% yield).

***Step 2*:** To a 100 mL seal tube equipped with a stir bar, **S-1-2** (5.0 mmol, 1.0 equiv, 2.67 g), B_2pin_2 (10.0 mmol, 2.0 equiv, 2.7 g), $Pd(dppf)Cl_2$ (0.5 mmol, 10 mol%, 0.367 g), KOAc (1.5 g, 15 mmol, 3.0 equiv) were mixed with dry 1,4-dioxane (20 mL) in a glovebox. The tube was then sealed and inside glovebox. The reaction mixture was left in a 120 °C oil bath with stirring for 8 h. Upon completion, the reaction was cooled to room temperature and water (100 mL) was added. The mixture was then extracted with DCM (30 mL x 3) and the combined organic extracts were dried with anhydrous

$MgSO_4$ and concentrated under vacuum. The product **S-1-3** (46% yield) was obtained through silica gel chromatography.

Step3**:** To a 50 mL round-bottom flask equipped with a stir bar, **S-1-3** (2.0 mmol, 1.0 equiv, 1.03 g) and $NaIO_4$ (6.0 mmol, 3.0 equiv, 1.28 g) was added in a glovebox. The flask was sealed with a septum inside the glovebox. Then, THF (15 mL), water (5 mL) and 1M HCl (1.2 mmol, 0.6 equiv, 1.2 mL) were added separately to the flask. The mixture was then stirred at room temperature for 4 h. The resulting mixture was extracted with Et_2O (30 mL x 3) and the combined organic extracts were dried with anhydrous $MgSO_4$ and concentrated under vacuum. The crude mixture was purified by silica gel chromatography to afford **S-1-4** (63% yield).

Preparation of boronic acid derived from 4-hydroxyflavanone[5]

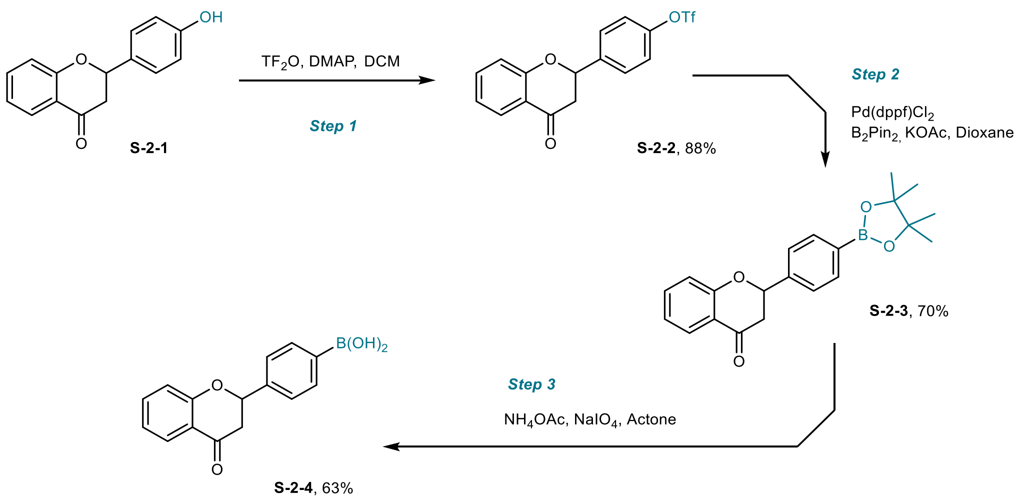

***Step 1*:** To a round-bottom flask equipped with a stir bar, 4'-hydroxyflavanone **S-2-1** (10 mmol, 1.0 equiv, 2.40 g), and DMAP (12 mmol, 1.2 equiv, 1.46 g) were mixed in DCM (100 mL) in a glovebox. The flask was then capped with a septum inside the glovebox. The reaction mixture was stirred at 0 °C, and Tf_2O (12 mmol, 2.02 mL) was added dropwise to the mixture over 5 min. The reaction was allowed to warm to room temperature and stirred for 1 h. Water (25 mL) was then added to quench the reaction, and the reaction mixture was extracted with DCM (30 mL x 3). The combined organic extracts were dried with anhydrous $MgSO_4$ and concentrated under vacuum. The crude was then purified by flash column chromatography on silica gel to give the corresponding trifluoromethanesulfonic ester **S-2-2** (82% yield).

***Step 2*:** To a 100 mL seal flask equipped with a stir bar, **S-2-2** (5.0 mmol, 1.0 equiv, 1.86 g), B_2pin_2 (10.0 mmol, 2.0 equiv, 2.7 g), Pd(dppf)Cl_2 (0.5 mmol, 10 mol%, 0.367 g), and KOAc (15 mmol, 3.0 equiv, 1.5 g) were mixed with dry 1,4-dioxane (20 mL) in a glovebox. The tube was then sealed inside the glovebox. After stirring at 120 °C for 8 h, the reaction was cooled to room temperature and filtered through a pad of Celite®. Water (30 mL) was added to the filtrate and the filtrate was extracted with DCM (30 mL x 3). The combined organic extracts were dried with anhydrous $MgSO_4$

and concentrated under vacuum. The product **S-2-3** (70% yield) was obtained through silica gel chromatography.

Step 3**:** To a 100 mL round-bottom flask equipped with a stir bar, **S-2-3** (2.0 mmol, 1.0 equiv, 0.70 g), NH_4OAc (12.0 mmol, 6.0 equiv, 0.92 g) and $NaIO_4$ (12.0 mmol, 6.0 equiv, 2.57 g) were added in a glovebox. The flask was sealed with a rubber cap inside the glovebox. Acetone (50 mL) and water (20 mL) were added separately to the flask and the mixture was stirred at room temperature for 48 h. The resulting mixture was filtered through a pad of Celite® and the filtrate was extracted with Et_2O (30 mL x 3). The combined organic extracts were dried with anhydrous $MgSO_4$ and concentrated under vacuum. The corresponding boronic acid **S-2-4** was obtained (63% yield) without further purification.

Preparation of boronic acid of estrone[6]

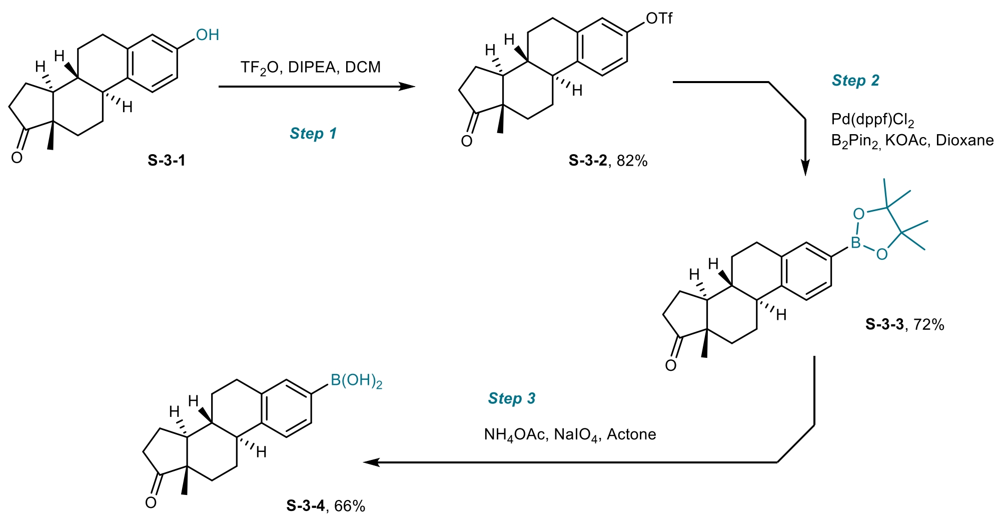

Step 1**:** To a 250 mL flask equipped with a stir bar, estrone **S-3-1** (20 mmol, 1.0 equiv, 5.41 g), and DIPEA (22 mmol, 1.1 equiv, 3.77 mL) were mixed with DCM (80 mL) in a glovebox. The flask was then sealed with a septum inside the glovebox. The reaction mixture was stirred at 0 °C, and Tf_2O (22 mmol, 1.1 equiv, 3.70 mL) was dropwise added into reaction system over 5 min. The reaction mixture was then allowed to warm to room temperature and stirred for 30 min. Upon completion, water (100 mL) was added to quench the reaction. The reaction mixture was then extracted with DCM (30 mL x 3). The combined organic extracts were dried with anhydrous $MgSO_4$ and concentrated under vacuum. The crude product was purified by flash column chromatography on silica gel to afford the corresponding trifluoromethanesulfonic ester **S-3-2** (82% yield).

Step 2**:** To a 100 mL seal tube equipped with a stir bar, **S-3-2** (5.0 mmol, 1.0 equiv), B_2pin_2 (10.0 mmol, 2.0 equiv, 2.7 g), Pd(dppf)Cl_2 (0.5 mmol, 10 mol%, 0.367 g), and KOAc (15 mmol, 3.0 equiv, 1.5 g) were mixed with dry 1,4-dioxane (20 mL) in a glovebox. The tube was then sealed inside the glovebox. The reaction mixture was stirred at 120 °C for 8 h. Upon completion, the reaction was cooled to room temperature and water (100 mL) was added. The mixture was extracted with DCM (30 mL x 3) and the combined organic extracts were dried with anhydrous $MgSO_4$ and concentrated

under vacuum. The product **S-3-3** (72% yield) was obtained through silica gel chromatography.

Step 3**:** To a 100 mL round-bottom flask equipped with a stir bar, **S-3-3** (2.0 mmol, 1.0 equiv, 0.76 g), NH_4OAc (12.0 mmol, 6.0 equiv, 0.92 g) and $NaIO_4$ (12.0 mmol, 6.0 equiv, 2.57 g) were weighed in a glovebox. The flask was sealed with a septum inside the glovebox. Acetone (50 mL) and water (20 mL) were added separately to the flask and the reaction mixture was stirred at room temperature for 48 h. Upon completion, the resulting mixture was filtered through a pad of Celite®. The filtrate was extracted with Et_2O (30 mL x 3). The combined organic layer was dried with anhydrous $MgSO_4$ and concentrated under vacuum. The corresponding boronic acid **S-3-4** was obtained (66% yield) without further purification.

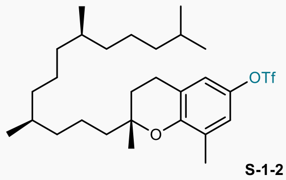

S-1-2

(S)-2,8-dimethyl-2-((4S,8R)-4,8,12-trimethyl-tridecyl)chroman-6-yl trifluoromethanesulfonate (S-1-2): colorless oil (91%). **^{1}H NMR** (400 MHz, Chloroform-*d*) δ= 6.84 (s, 1H), 6.80 (s, 1H), 2.74 (m, 2H), 2.16 (s, 3H), 1.85-1.70 (m, 2h), 1.59-1.49 (m, 3H), 1.45-1.38 (m, 6H), 1.30-1.27 (m, 10H), 1.17-1.06 (m, 5H), 0.86 (m, 12H) ppm. **^{13}C NMR** (101 MHz, Chloroform-*d*) δ= 151.7, 141.5, 128.4, 121.7, 120.8, 119.1, 76.8, 40.2, 39.4, 37.5, 37.4, 37.3, 32.8, 32.7, 30.7, 28.0, 24.8, 24.5, 24.0, 22.7, 22.6, 22.4, 20.9, 19.7, 19.6, 16.1 ppm. **^{19}F NMR** (376 MHz, Chloroform-*d*) δ= -73.11 (s, 3F) ppm. **HRMS** (EI): calcd. for $C_{28}H_{45}F_{3}O_{4}S$: 534.2991. found: 534.3002.

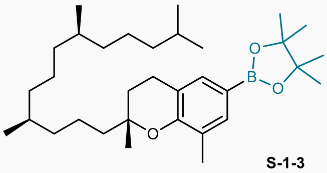

S-1-3

2-((S)-2,8-dimethyl-2-((4S,8R)-4,8,12-trimethyltridecyl)chroman-6-yl)-4,4,5,5-tetramethyl-1,3,2-dioxaborolane (S-1-3): colorless oil (46%). **^{1}H NMR** (400 MHz, Chloroform-*d*) δ= 7.42 (s, 1H), 7.40 (s, 1H), 2.75 (t, *J* = 6.68 H, 2H), 2.16 (m, 3H), 1.85-1.70 (m, 2H), 1.59-1.49 (m, 3H), 1.45-1.36 (m, 4H), 1.32 (s, 12H), 1.28-1.07 (m, 17H), 0.87 (s, 3H), 0.85 (m, 6H), 0.83 (m, 3H) ppm. **^{13}C NMR** (101 MHz, Chloroform-*d*) δ= 155.2, 134.9, 134.1, 125.7, 119.9, 83.4, 76.5, 40.2, 39.4, 37.4, 37.3, 32.8, 32.7, 31.2, 28.0, 24.8, 24.5, 24.4, 22.7, 22.6, 22.1, 21.0, 19.8, 19.7, 15.8 ppm. **HRMS** (EI): calcd. for $C_{33}H_{57}BO_{3}$: 512.4401. found: 512.4421.

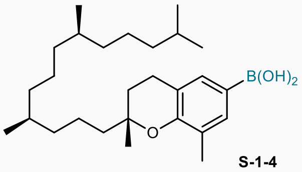

S-1-4

((S)-2,8-dimethyl-2-((4S,8R)-4,8,12-trimethyltridecyl)chroman-6-yl)boronic acid (S-1-4): white solid, melting point: 158 °C (63%). **^{1}H NMR** (400 MHz, Chloroform-*d*) δ= 7.80 (s, 1H), 7.79 (s, 1H), 2.87 (t, *J* = 6.28 Hz, 2H), 2.27 (s, 3H), 1.91-1.77 (m, 2H), 1.64-1.54 (m, 3H), 1.52-1.38 (m, 5H), 1.32-1.24 (m, 13H), 1.16-1.08 (m, 5H), 0.87 (m, 12H) ppm. **^{13}C NMR** (101 MHz, Chloroform-*d*) δ= 156.2, 135.8, 135.3, 125.8, 120.5, 120.0, 76.7, 40.3, 39.4, 37.5, 37.4, 37.3, 32.9, 32.7, 31.3, 28.0, 24.9, 24.5, 24.4, 22.8, 22.7, 22.3, 21.1, 19.8, 19.7, 16.2 ppm. **HRMS** (EI): calcd. for $C_{27}H_{47}BO_{3}$: 430.3618. found: 430.3630.

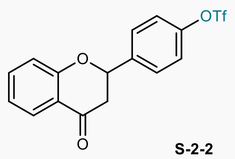
S-2-2

4-(4-Oxochroman-2-yl)phenyl trifluoromethanesulfonate (S-2-2): white solid, melting point: 77 °C (88%). **^{1}H NMR** (400 MHz, Chloroform-*d*) δ= 7.95 (dd, *J* = 7.84, 1.56 Hz, 1H), 7.60 (d, *J* = 8.68 Hz, 2H), 7.54 (m, 1H), 7.36 (d, *J* = 8.68 Hz, 2H), 7.11-7.06 (m, 2H), 5.53 (dd, *J* = 13.08, 3.04 Hz, 1H), 3.04 (dd, *J* = 16.84, 13.12 Hz, 1H), 2.92 (dd, *J* = 16.80, 3.12 Hz, 1H) ppm. **^{13}C NMR** (101 MHz, Chloroform-*d*) δ= 191.1, 161.1, 149.4, 139.4, 136.4, 128.0, 127.2, 122.0, 121.9, 120.9, 118.1, 78.5, 44.7 ppm. **^{19}F NMR** (376 MHz, Chloroform-*d*) δ= -72.76 ppm. **HRMS** (ESI): calcd. for $C_{16}H_{11}F_2NaO_5S^+$: 395.0171. found: 395.0168.

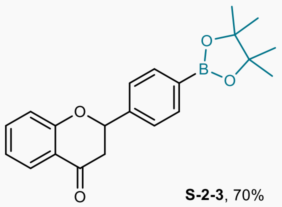
S-2-3, 70%

2-(4-(4,4,5,5-Tetramethyl-1,3,2-dioxaborolan-2-yl)phenyl)chroman-4-one (S-2-3): white solid, melting point: 155 °C (70%). **^{1}H NMR** (400 MHz, Chloroform-*d*) δ= 7.90 (m, 3H), 7.48 (m, 3H), 7.04 (m, 2H), 5.47 (dd, *J* = 13.20, 2.80 Hz, 1H), 3.03 (dd, *J* = 16.84, 13.24 Hz, 1H), 2.87 (dd, *J* = 16.88, 2.92 Hz, 1H), 1.34 (s, 12H) ppm. **^{13}C NMR** (101 MHz, Chloroform-*d*) δ= 191.7, 161.5, 141.7, 136.2, 135.3, 127.0, 125.3, 121.6, 120.9, 118.2, 83.9, 79.5, 44.7, 24.9 ppm.

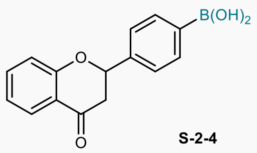
S-2-4

(4-(4-Oxochroman-2-yl)phenyl)boronic acid (S-2-4): white solid, melting point: 155 °C (63%). **^{1}H NMR** (400 MHz, DMSO-*d*) δ= 8.09 (s, 2H), 7.81 (m, 3H), 7.58 (t, *J* = 7.52 Hz, 1H), 7.49 (d, *J* = 7.68 Hz, 2H), 7.10 (m, 2H), 5.67 (d, *J* = 10.60 Hz, 1H), 3.23 (m, 1H), 2.83 (m, 1H) ppm. **^{13}C NMR** (101 MHz, Chloroform-*d*) δ= 192.0, 161.5, 141.0, 136.8, 134.8, 126.8, 126.0, 121.9, 121.2, 118.5, 79.3, 44.9 ppm.

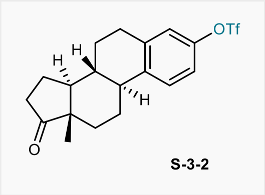

(8R,9S,13S,14S)-13-methyl-17-oxo-7,8,9,11,12,13,14,15,16,17-decahydro-6H-cyclopenta[a]phenanthren-3-yl trifluoromethanesulfonate (S-3-2): white solid, melting point: 91 °C (82%). **^{1}H NMR** (400 MHz, Chloroform-*d*) δ= 7.35 (d, *J* = 8.64 Hz, 1H), 7.04 (m, 1H), 7.00 (s, 1H), 2.95 (m, 2H), 2.52 (m, 1H), 2.43-2.39 (m, 1H), 2.33-2.27 (m, 1H), 2.20-1.96 (m, 4H), 1.68-1.44 (m, 6H), 0.92 (s, 3H) ppm. **^{13}C NMR** (101 MHz, Chloroform-*d*) δ= 220.4, 147.6, 140.3, 139.3, 127.2, 121.2, 118.9 (q, *J* = 322.0 Hz), 50.4, 47.9, 44.1, 37.7, 35.8, 31.5, 29.4, 26.1, 25.7, 21.6, 13.8 ppm. **^{19}F NMR** (376 MHz, Chloroform-*d*) δ= -72.99 ppm. **HRMS** (ESI): calcd. for $C_{19}H_{21}F_3NaO_4S^+$: 425.1005. found: 425.1007.

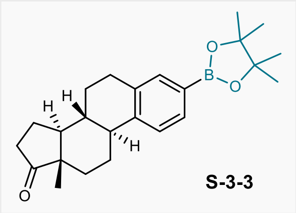

(8R,9S,13S,14S)-13-methyl-3-(4,4,5,5-tetramethyl-1,3,2-dioxaborolan-2-yl)-6,7,8,9,11,12,13,14,15,16-decahydro-17H-cyclopenta[a]phenanthren-17-one (S-3-3): white solid, melting point: 193 °C (72%). **^{1}H NMR** (400 MHz, Chloroform-*d*) δ= 7.60 (d, *J* = 7.84 Hz, 1H), 7.57 (s, 1H), 7.32 (d, *J* = 7.76 Hz, 1H), 2.94 (m, 2H), 2.54-2.44 (m, 2H), 2.33 (m, 1H), 2.20-1.96 (m, 4H), 1.66-1.43 (m, 6H), 1.34 (s, 12H), 0.91 (s, 3H) ppm. **^{13}C NMR** (101 MHz, Chloroform-*d*) δ= 143.1, 135.8, 135.6, 132.2, 124.8, 83.7, 50.6, 48.0, 44.7, 38.0, 35.9, 31.6, 29.1, 26.5, 25.6, 24.9, 24.8, 21.6, 13.8 ppm.

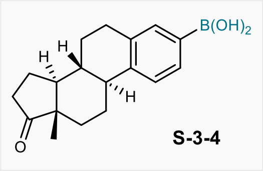

((8R,9S,13S,14S)-13-methyl-17-oxo-7,8,9,11,12,13,14,15,16,17-decahydro-6H-cyclopenta[a]phenanthren-3-yl)boronic acid (S-3-4): white solid, melting point: 196 °C (66%). **^{1}H NMR** (400 MHz, Chloroform-*d*) δ= 7.84 (s, 2H), 7.53 (d, *J* = 7.67 Hz, 1H), 7.49 (s, 1H), 7.20 (d, *J* = 7.98 Hz, 1H), 3.37 (s, 1H), 2.80 (m, 2H), 2.43-2.33 (m, 2H), 2.19 (m, 1H), 2.05-1.98 (m, 1H), 1.94-1.90 (m, 2H), 1.74 (m, 1H), 1.52-1.41 (m, 2H), 1.39-1.32 (m, 3H), 0.79 (s, 3H) ppm. **^{13}C NMR** (101 MHz, Chloroform-*d*) δ= 141.9, 135.4, 135.3, 132.0, 131.4, 124.6, 50.1, 47.7, 44.5, 38.1, 35.8, 31.8, 29.3, 26.5, 25.7, 21.6, 14.0 ppm.

3. Exploration of the coupling partners with the BzO-DF building blocks

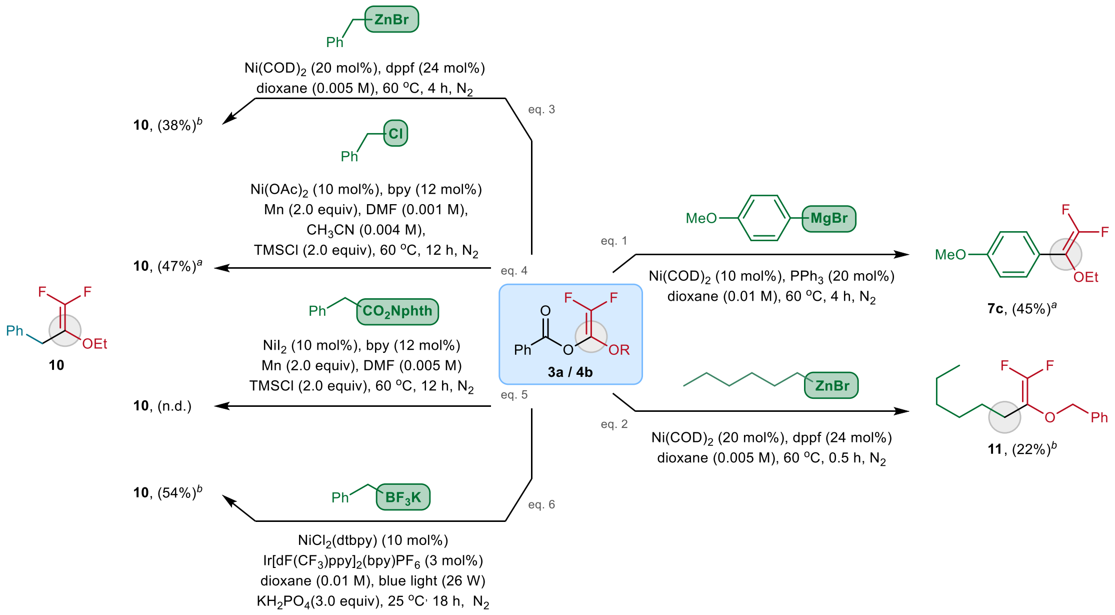

[a]Yields determined by ^{19}F NMR with $PhCF_3$ as an internal standard. [b]Isolated yield.

Procedures for Ni-catalyzed cross-coupling of 3a and Grignard reagent (eq. 1)

To a seal tube equipped with a stirrer, $Ni(COD)_2$ (10 mol%), and PPh_3 (20 mol%) were mixed with dioxane (2.0 mL) in a glovebox. After stirring for 10 min, the mixture was transferred to another nitrogen-filled seal tube containing **3a** (0.2 mmol). The reaction mixture was then stirred at 60 °C and Grignard reagent (0.4 mmol, 1M in dioxane) was added dropwise to the mixture over 1 h. The reaction was then stirred at 60 °C for another 3 h. The crude reaction mixture was subjected to ^{19}F NMR analysis with $PhCF_3$ as an internal standard.

Procedures for Ni-catalyzed cross-coupling of 4b and *n*-Hexyl-ZnBr (eq. 2)

To a seal tube equipped with a stirrer, $Ni(COD)_2$ (20 mol%) and dppf (24 mol%) were mixed with dioxane (2.0 mL) in a glovebox. After stirring for 10 min, the mixture

was transferred to another seal tube with **4b** (0.2 mmol). The tube was then sealed with a rubber cap before taken out from the glovebox. The reaction mixture was stirred at 60 ^{o}C and *n*-hexyl-ZnBr (0.4 mmol, 1M in THF) was added dropwise into mixture over 5 min. The reaction was then stirred at 60 ^{o}C for 25 min. The resulting mixture was filtered through a pad of Celite® and concentrated in vacuum. The crude product was purified by silica gel chromatography to afford **11** in 22% yield.

Procedures for Ni-catalyzed cross-coupling of 3a and benzylzinc-Br (eq. 3)

To a seal tube equipped with a stirrer, $Ni(COD)_2$ (20 mol%) and dppf (24 mol%) were mixed with dioxane (4.0 mL) in a glovebox. After stirring for 10 min, the mixture was transferred to another seal tube containing **3a** (0.2 mmol). The tube was then sealed with a rubber cap and taken out from the glovebox. After stirring at 60 ^{o}C and organozinc reagent (0.4 mmol, 1M in THF) was added dropwise into the reaction system over 30 min. The reaction was then stirred at 60 ^{o}C for 3 h and the resulting mixture was filtered through a pad of Celite®. The filtrate was concentrated in vacuum and the crude product was purified by silica gel chromatography to afford **10** in 38% yield.

Procedures for Ni-catalyzed cross-coupling of 3a and benzyl chloride (eq. 4)

To a seal tube equipped with a stirrer, $Ni(OAc)_2$ (10 mol%) and bpy (12 mol%), were mixed with CH_3CN (0.8 mL) and DMF (0.2 mL) in a glovebox. After stirring for 10 min, the mixture was transferred to another seal tube containing **3a** (0.2 mmol), benzyl chloride (0.4 mmol), Mn powder (0.4 mmol) and TMSCl (0.4 mmol). The tube was then sealed with a rubber cap and taken out from the glovebox. The reaction mixture was stirred at 60 ^{o}C for 12 h. The reaction crude was subjected to ^{19}F NMR analysis with $PhCF_3$ as an internal standard.

Procedure for Ni-catalyzed cross-coupling of 3a and potassium benzyltrifluoroborate (eq. 6)

To a seal tube equipped with a stirrer, **3a** (0.2 mmol), potassium benzyltrifluoroborate (0.8 mmol), $NiCl_2$(dtbpy) (10 mol%), Ir[dF(CF_3)ppy]$_2$(bpy)PF_6 (3 mol%), and KH_2PO_4 (0.6 mmol) were mixed with dioxane (2.0 mL) in the glovebox. The tube was then sealed with a rubber cap and taken out from the glovebox. The reaction mixture was irradiated under a 26 W blue light with stirring at 25 °C for 18 h. The resulting mixture was filtered through a pad of Celite® and concentrated in vacuum. The crude product was purified by silica gel chromatography to afford **10** in 54% yield.

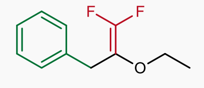

10

(2-ethoxy-3,3-difluoroallyl)benzene (10): colorless oil (38–54%). **^{1}H NMR** (400 MHz, Chloroform-*d*) δ= 7.30 (m, 2H), 7.24 (m, 3H), 3.68 (q, *J* = 6.80 Hz, 2H), 3.42 (dd, *J* = 3.84, 2.00 Hz, 2H), 1.14 (t, *J* = 7.08 Hz, 3H) ppm. **^{13}C NMR** (101 MHz, Chloroform-*d*) δ= 154.4 (t, *J* = 281.0 Hz), 154.3, 137.3, 128.6, 128.5, 126.7, 116.6 (dd, *J* = 39.2, 13.0 Hz), 67.2 (m), 32.8, 15.1 ppm. **^{19}F NMR** (376 MHz, Chloroform-*d*) δ= -103.27 (d, *J* = 76.06 Hz, 1F), -115.40 (d, *J* = 76.10 Hz, 1F) ppm. **HRMS** (EI): calcd. for $C_{11}H_{12}F_2O$: 198.0856. found: 198.0853.

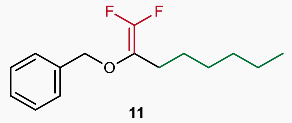

11

(((1,1-Difluorooct-1-en-2-yl)oxy)methyl)benzene (11): colorless oil (22%). **^{1}H NMR** (400 MHz, Chloroform-*d*) δ= 7.37-7.33 (m, 5H), 4.74 (s, 2H), 2.13-2.09 (m, 2H), 1.51-1.45 (m, 2H), 1.30-1.26 (m, 6H), 0.88 (t, *J* = 6.98 Hz, 3H) ppm. **^{13}C NMR** (101 MHz, Chloroform-*d*) δ= 154.4 (t, *J* = 281.0 Hz), 151.5, 137.0, 128.4, 128.1, 128.0, 117.4, 117.0, 73.0, 31.5, 28.6, 26.1, 26.0, 22.6, 14.0 ppm. **^{19}F NMR** (376 MHz, Chloroform-*d*) δ= -102.55 (d, *J* = 77.84 Hz, 1F), -115.63 (d, *J* = 78.39 Hz, 1F) ppm. **HRMS** (ESI): calcd. for $C_{15}H_{21}F_2O^+$: 255.1555. found: 255.1552.

4. Preparation and physical characterization data

Optimization studies for the Ni-catalyzed cross-coupling reaction between BzO-DFs (3a) and boronic acids (6c)

Supplementary Table 2. Optimization of reaction conditions[a]

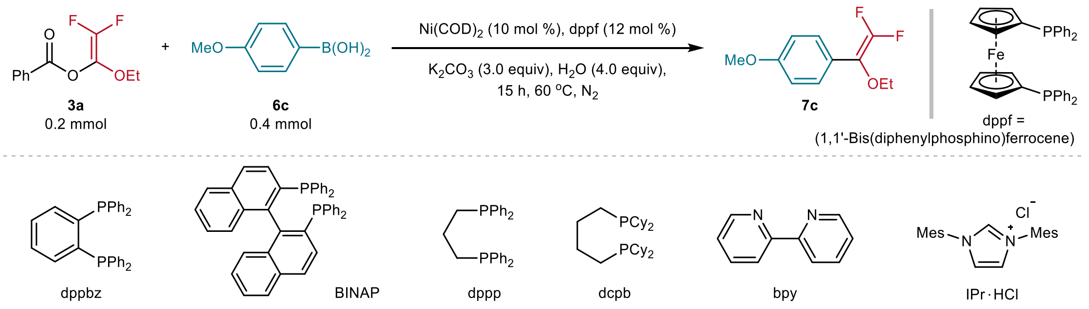

entry	deviations from standard conditions	yield[b] (%)
**1**	**None**	**87 (84)** [c]
2	PPh_3	<5
3	dppbz	<5
4	BINAP	<5
5	dppp	<5
6	dcpb	71
7	bpy	n.d.
8	IPr○HCl	n.d.
9	KOH	n.d.
10	KO^{t}Bu	n.d.
11	K_3PO_4	53
12	Na_2CO_3	62
13	Cs_2CO_3	<5
14	DABCO	n.d.
15	without H_2O	31
16	H_2O (2.0 equiv)	84
17	H_2O (4.0 equiv)	87
18	H_2O (6.0 equiv)	85
19	H_2O (8.0 equiv)	48
20	THF	43
21	ethyl acetate	36

22	trifluorotoluene	10
23	DCM	7
24	CH_3CN	<5
25	acetone	<5
26	DMF	n.d.
27	DMSO	n.d.
28	$Ni(PPh_3)_4$	40
29	$NiCl_2$	n.d.
30	$Ni(OAc)_2$	n.d.
31	without Ni catalyst	n.d.
32	without ligand	n.d.

[a]Reaction conditions: **3a** (0.2 mmol), **6c** (0.4 mmol), $Ni(COD)_2$ (10 mol%), ligand (12 mol%), K_2CO_3 (3.0 equiv) and water (4.0 equiv) in dioxane (2.0 mL) at 60 °C for 15 h under nitrogen atmosphere. [b]Yields determined by ^{19}F NMR with $PhCF_3$ as an internal standard. [c]Isolated yield in parentheses.

Scope of *gem*-difluoroalkenes and *gem*-difluoroenol ethers

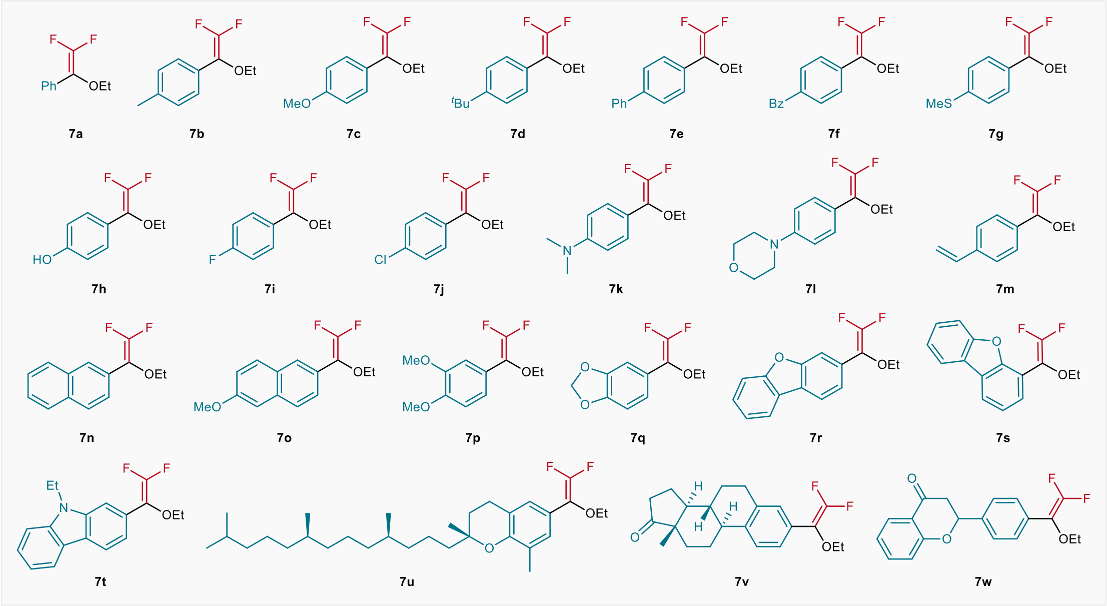

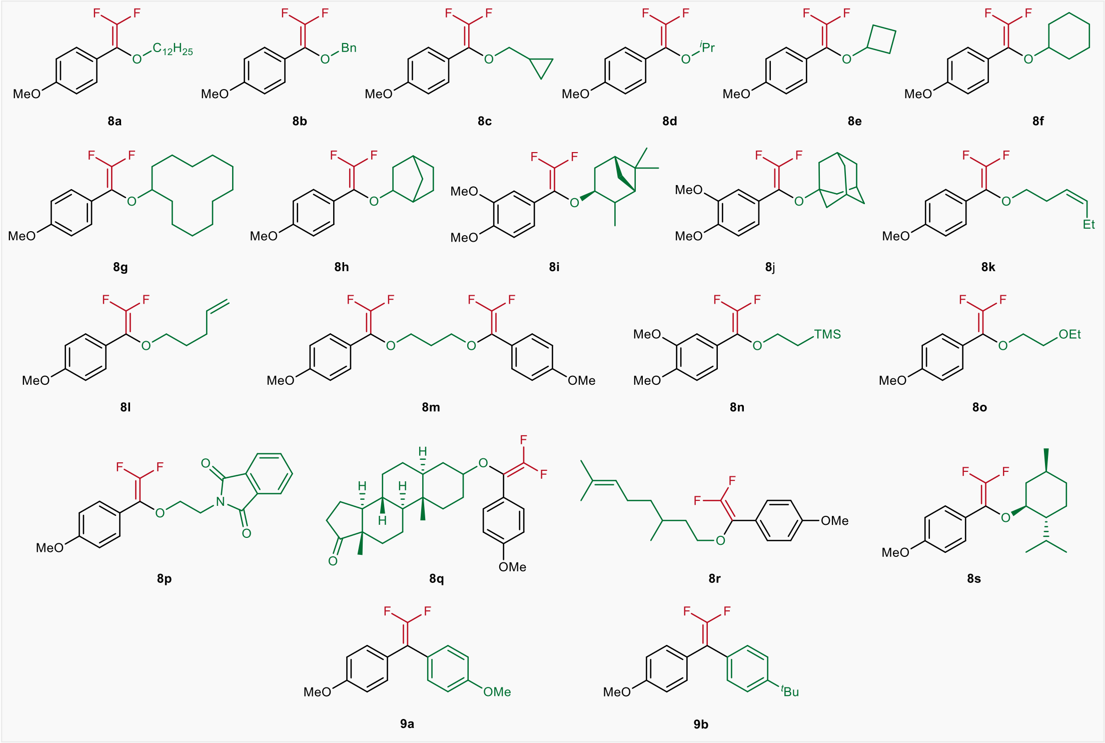

General Procedure for Ni-catalyzed cross-coupling reaction between BzO-DFs and arylboronic acids

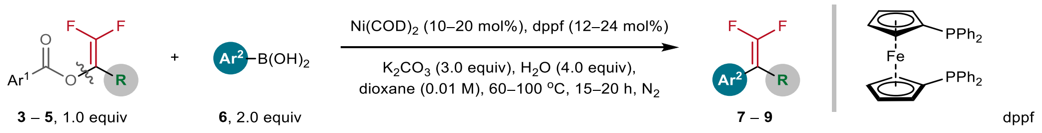

To a 8 mL vial equipped with a magnetic stir, 2,2-difluorovinyl benzoate **3–5** (1.0 equiv, 0.2 mmol), boronic acid **6** (2.0 equiv, 0.4 mmol), and K_2CO_3 (3.0 equiv, 0.6 mmol) were mixed with deionized water (4.0 equiv, 0.8 mmol). Then a premixed solution of $Ni(COD)_2$ (10 mol%, 0.02 mmol), and dppf (12 mol%, 0.024 mmol) in dioxane (0.01 M, 2.0 mL) was transferred to the 8 mL vial. The vial was then capped inside the glovebox. The reaction mixture was stirred for 15 hours at 60 °C. Upon completion, the reaction mixture was cooled to room temperature and diluted with ethyl acetate. The solution was then filtered through a pad of Celite® and the filtrate was dried over $MgSO_4$ and concentrated in *vacuo*. The residue was then purified by flash column chromatography (gradient elution: *n*-hexane:ethyl acetate = 200:1~50:1) to give the desired products.

For **(7a – 7f, 7i – 7o and 7q – 7t)** the coupling was performed in the presence of $Ni(COD)_2$ (10 mol%), dppf (12 mol%), and K_2CO_3 (3.0 equiv) in dioxane (0.01 M, 2.0 mL) at 80 °C for 20 h.

For **(7g, 7h, 7p and 7u – 7w)** the coupling was performed in the presence of $Ni(COD)_2$ (20 mol%), dppf (24 mol%), and K_2CO_3 (3.0 equiv) in dioxane (0.01 M, 2.0 mL) at 80 °C for 20 h.

For **9a** and **9b**, the coupling was performed in the presence of $Ni(COD)_2$ (20 mol%), dppf (24 mol%), K_2CO_3 (3.0 equiv), and water (4.0 equiv) in dioxane (2.0 mL) at 100 °C for 20 h.

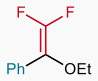

7a

(1-Ethoxy-2,2-difluorovinyl)benzene (7a): colorless oil (69%). **^{1}H NMR** (400 MHz, Chloroform-*d*) δ= 7.47 (d, *J* = 8.20 Hz, 2H), 7.38 (t, *J* = 7.40 Hz, 2H), 7.29 (t, *J* = 7.28 Hz, 1H), 3.78 (q, *J* = 7.00 Hz, 2H), 1.30 (t, *J* = 7.00 Hz, 3H) ppm. **^{13}C NMR** (101 MHz, Chloroform-*d*) δ= 155.6 (t, *J* = 291.7 Hz), 130.5 (d, *J* = 6.9 Hz), 128.5, 128.0, 126.4 (m), 117.8 (dd, *J* = 33.1, 18.2 Hz), 67.7, 15.1 ppm. **^{19}F NMR** (376 MHz, Chloroform-*d*) δ= -98.13 (d, *J* = 57.19 Hz, 1F), -107.62 (d, *J* = 57.23 Hz, 1F) ppm. **HRMS** (EI): calcd. for $C_{10}H_{10}F_2O$: 184.0700. found: 184.0702.

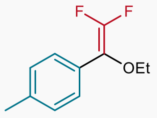

7b

1-(1-Ethoxy-2,2-difluorovinyl)-4-methylbenzene (7b): colorless oil (75%). **^{1}H NMR** (400 MHz, Chloroform-*d*) δ= 7.36 (d, *J* = 7.24 Hz, 2H), 7.20 (d, *J* = 8.08 Hz, 2H), 3.77 (q, *J* = 7.00 Hz, 2H), 2.36 (s, 3H), 1.29 (t, *J* = 7.08 Hz, 3H) ppm. **^{13}C NMR** (101 MHz, Chloroform-*d*) δ= 155.4 (t, *J* = 290.7 Hz), 138.0, 129.2, 127.5 (d, *J* = 5.4 Hz), 126.4 (dd, *J* = 5.9, 3.6 Hz), 117.8 (dd, *J* = 33.7, 18.3 Hz), 67.6, 21.2, 15.1 ppm. **^{19}F NMR** (376 MHz, Chloroform-*d*) δ= -99.02 (d, *J* = 59.18 Hz, 1F), -108.36 (d, *J* = 58.99 Hz, 1F) ppm. **HRMS** (EI): calcd. for $C_{11}H_{12}F_2O$: 198.0856. found: 198.0850.

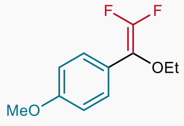

7c

1-(1-Ethoxy-2,2-difluorovinyl)-4-methoxybenzene (7c): colorless oil (84%). **^{1}H NMR** (400 MHz, Chloroform-*d*) δ= 7.39 (d, *J* = 8.24 Hz, 2H), 6.92 (d, *J* = 8.80 Hz, 2H), 3.82 (s, 3H), 3.76 (q, *J* = 7.04 Hz, 2H), 1.29 (t, *J* = 7.00 Hz, 3H) ppm. **^{13}C NMR** (101 MHz, Chloroform-*d*) δ= 159.4, 155.2 (t, *J* = 289.6 Hz), 127.9 (dd, *J* = 5.8, 3.6 Hz), 122.7 (d, *J* = 6.6 Hz), 117.5 (dd, *J* = 33.7, 18.1 Hz), 114.0, 67.5 (m), 55.3, 15.1 ppm. **^{19}F NMR** (376 MHz, Chloroform-*d*) δ= -100.11 (d, *J* = 62.53 Hz, 1F), -109.62 (d, *J* = 62.45 Hz, 1F) ppm. **HRMS** (ESI): calcd. for $C_{11}H_{13}F_2O_2^+$: 215.0878. found: 215.0883.

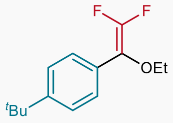

7d

1-(Tert-butyl)-4-(1-ethoxy-2,2-difluorovinyl)benzene (7d): colorless oil (79%). **^{1}H NMR** (400 MHz, Chloroform-*d*) δ= 7.40 (s, 4H), 3.78 (q, *J* = 7.04 Hz, 2H), 1.33 (s, 9H), 1.29 (m, 3H) ppm. **^{13}C NMR** (101 MHz, Chloroform-*d*) δ= 155.5 (t, *J* = 291.1 Hz), 151.1, 127.5 (d, *J* = 7.5 Hz), 126.1 (dd, *J* = 6.0, 3.5 Hz), 125.5,

117.7 (dd, *J* = 33.2, 18.1 Hz), 67.6 (t, *J* = 2.5 Hz), 34.6, 31.2, 15.1 ppm. **^{19}F NMR** (376 MHz, Chloroform-*d*) δ= -98.80 (d, *J* = 59.14 Hz, 1F), -108.18 (d, *J* = 58.77 Hz, 1F) ppm. **HRMS** (EI): calcd. for $C_{14}H_{18}F_2O$: 240.1326. found: 240.1330.

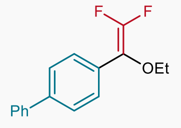

7e

4-(1-Ethoxy-2,2-difluorovinyl)-1,1'-biphenyl (7e): white solid, melting point: 34 °C (83%). **^{1}H NMR** (400 MHz, Chloroform-*d*) δ= 7.61 (m, 4H), 7.55 (m, 2H), 7.44 (m, 2H), 7.35 (m, 1H), 3.82 (q, *J* = 7.04 Hz, 2H), 1.33 (t, *J* = 7.04 Hz, 3H) ppm. **^{13}C NMR** (101 MHz, Chloroform-*d*) δ= 155.7 (t, *J* = 292.9 Hz), 140.8, 140.4, 129.5 (d, *J* = 5.6 Hz), 128.9, 127.6, 127.2, 127.0, 126.7 (dd, *J* = 6.2, 3.6 Hz), 117.7 (dd, *J* = 32.8, 18.0 Hz), 67.9, 15.1 ppm. **^{19}F NMR** (376 MHz, Chloroform-*d*) δ= -97.55 (d, *J* = 56.6 Hz), -106.91 (d, *J* = 56.1 Hz) ppm. **HRMS** (EI): calcd. for $C_{16}H_{14}F_2O$: 260.1013. found: 260.1018.

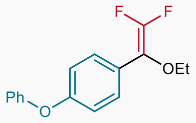

7f

1-(1-Ethoxy-2,2-difluorovinyl)-4-phenoxybenzene (7f): colorless oil (72%). **^{1}H NMR** (400 MHz, Chloroform-*d*) δ= 7.43 (d, *J* = 8.24 Hz, 2H), 7.36 (t, *J* = 7.80 Hz, 2H), 7.13 (t, *J* = 7.40 Hz, 1H), 7.02 (m, 4H), 3.79 (q, *J* = 7.04 Hz, 2H), 1.30 (t, *J* = 7.04 Hz, 3H) ppm. **^{13}C NMR** (101 MHz, Chloroform-*d*) δ= 157.3, 156.6, 155.4 (t, *J* = 290.9 Hz), 129.9, 127.9 (dd, *J* = 6.2, 3.6 Hz), 125.1 (d, *J* = 6.5 Hz), 123.7, 119.3, 118.5, 117.4 (dd, *J* = 33.6, 18.3 Hz), 67.7 (m), 15.1 ppm. **^{19}F NMR** (376 MHz, Chloroform-*d*) δ= -98.97 (d, *J* = 59.93 Hz, 1F), -108.46 (d, *J* = 59.93 Hz, 1F) ppm. **HRMS** (EI): calcd. for $C_{16}H_{14}F_2O_2$: 276.0962. found: 276.0965.

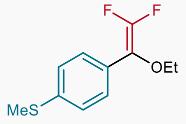

7g

(4-(1-Ethoxy-2,2-difluorovinyl)phenyl)(methyl)sulfane (7g): colorless oil (30%). **^{1}H NMR** (400 MHz, Chloroform-*d*) δ= 7.39 (d, *J* = 7.76 Hz, 2H), 7.26 (,d *J* = 8.44 Hz, 2H), 3.77 (q, *J* = 7.04 Hz, 2H), 2.49 (s, 3H), 1.30 (t, *J* = 7.02 Hz, 3H) ppm. **^{13}C NMR** (101 MHz, Chloroform-*d*) δ= 155.5 (t, *J* = 291.3 Hz), 138.7, 127.1 (d, *J* = 6.4 Hz), 126.7 (dd, *J* = 6.6, 3.6 Hz), 126.3, 117.5 (dd, *J* = 33.0, 18.4 Hz), 67.8, 15.6, 15.1

ppm. ^{19}**F NMR** (376 MHz, Chloroform-*d*) δ= -98.18 (d, *J* = 57.64 Hz), 107.47 (d, *J* = 58.00 Hz) ppm. **HRMS** (EI): calcd. for $C_{11}H_{12}F_2OS$: 230.0577. found: 230.0587.

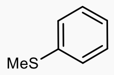

7g'

Methyl(phenyl)sulfane (7g′): colorless oil (43). ^{1}**H NMR** (400 MHz, Chloroform-*d*) δ= 7.33-7.26 (m, 4H), 7.18-7.14 (m, 1H), 2.50 (s, 3H) ppm. ^{13}**C NMR** (101 MHz, Chloroform-*d*) δ= 138.5, 128.8, 126.7, 125.1, 15.9 ppm. **HRMS** (EI): calcd. for C_7H_8S: 124.0347. found: 124.0343.

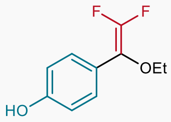

7h

4-(1-Ethoxy-2,2-difluorovinyl)phenol (7h): colorless oil (34%). ^{1}**H NMR** (400 MHz, Chloroform-*d*) δ= 7.35 (d, *J* = 7.88 Hz, 2H), 6.86 (d, *J* = 8.72 Hz, 2H), 5.34 (br s, 1H), 3.77 (q, *J* = 7.04 Hz, 2H), 1.29 (t, *J* = 7.04 Hz, 3H) ppm. ^{13}**C NMR** (101 MHz, Chloroform-*d*) δ= 155.4, 150.2 (t, *J* = 289.8 Hz), 128.1 (dd, *J* = 5.6, 3.4 Hz), 122.8 (d, *J* = 6.4 Hz), 117.4 (dd, *J* = 34.2, 18.5 Hz), 115.5, 67.6 (m), 15.0 ppm. ^{19}**F NMR** (376 MHz, Chloroform-*d*) δ= -99.89 (d, *J* = 61.63 Hz, 1F), -109.34 (d, *J* = 62.34 Hz, 1F) ppm. **HRMS** (EI): calcd. for $C_{10}H_{10}F_2O_2$: 200.0649. found: 200.0643.

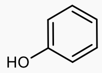

3h'

Phenol (3h′): white solid, melting point: 42 °C (45%). ^{1}**H NMR** (400 MHz, Chloroform-*d*) δ= 7.26 (t, *J* = 7.75 Hz, 2H), 6.95 (t, *J* = 7.75 Hz, 1H), 6.85 (t, *J* = 7.98 Hz, 2H), 4.97 (br s, 1H) ppm. ^{13}**C NMR** (101 MHz, Chloroform-*d*) δ= 155.4, 129.7, 120.9, 115.3 ppm. **HRMS** (EI): calcd. for C_6H_6O: 94.0419. found: 94.0424.

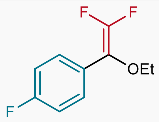

7i

1-(1-Ethoxy-2,2-difluorovinyl)-4-fluorobenzene (7i): colorless oil (61%). ^{1}**H NMR** (400 MHz, Chloroform-*d*) δ= 7.45 (dd, *J* = 8.08, 5.56 Hz, 2H), 7.08 (t, *J* = 8.68 Hz, 2H), 3.77 (q, *J* = 7.00 Hz, 2H), 1.30 (t, *J* = 7.04 Hz, 3H) ppm. ^{13}**C NMR** (101 MHz, Chloroform-*d*) δ= 13C NMR (101 MHz, Chloroform-*d*) δ= 162.3 (d, *J* = 248.9 Hz), 155.4 (t, *J* = 291.2 Hz), 128.2, 126.5, 117.1 (dd, *J* = 33.6, 18.6 Hz), 115.6 (d,

J = 21.9 Hz), 67.7, 15.1 ppm. **^{19}F NMR** (376 MHz, Chloroform-*d*) δ= -98.49 (d, J = 58.62 Hz, 1F), -108.13 (d, J = 58.66 Hz, 1F), -113.25 (s, 1F) ppm. **HRMS** (EI): calcd. for $C_{10}H_9F_3O$: 202.0605. found: 202.0610.

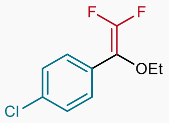

7j

1-Chloro-4-(1-ethoxy-2,2-difluorovinyl)benzene (7j): colorless oil (42%). **^{1}H NMR** (400 MHz, Chloroform-*d*) δ= 7.39 (m, 4H), 3.78 (q, J = 6.96 Hz, 2H), 1.31 (t, J = 6.96 Hz, 3H) ppm. **^{13}C NMR** (101 MHz, Chloroform-*d*) δ= 155.6 (t, J = 292.4 Hz), 133.8, 129.1 (d, J = 5.4 Hz), 128.8, 127.6 (dd, J = 6.6, 3.6 Hz), 117.2 (dd, J = 33.0, 18.7 Hz), 68.0 (m), 15.1 ppm. **^{19}F NMR** (376 MHz, Chloroform-*d*) δ= -97.09 (d, J = 55.65 Hz, 1F), -106.45 (d, J = 55.61 Hz, 1F) ppm. **HRMS** (EI): calcd. for $C_{10}H_9ClF_2O$: 218.0310. found: 218.0319.

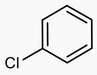

3j'

Chlorobenzene (3j′): colorless oil (51%). **^{1}H NMR** (400 MHz, Chloroform-*d*) δ= 7.36-7.25 (m, 5H) ppm. **^{13}C NMR** (101 MHz, Chloroform-*d*) δ= 134.3, 129.7, 128.6, 126.4 ppm. **HRMS** (EI): calcd. for C_6H_5Cl: 112.0080. found: 112.0073.

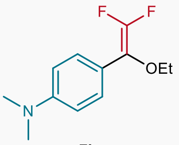

7k

4-(1-Ethoxy-2,2-difluorovinyl)-N,N-dimethylaniline (7k): colorless oil (52%). **^{1}H NMR** (400 MHz, Chloroform-*d*) δ= 7.33 (d, J = 8.16 Hz, 2H), 6.73 (d, J = 8.84 Hz, 2H), 3.76 (q, J = 7.00 Hz, 2H), 2.98 (s, 6H), 1.28 (t , J = 7.00 Hz, 3H) ppm. **^{13}C NMR** (101 MHz, Chloroform-*d*) δ= 155.0 (t, J = 288.6 Hz), 150.1, 127.5 (d, J = 9.14 Hz), 127.5 (d, J = 2.07 Hz), 117.8 (m), 112.1, 67.2, 40.3, 15.1 ppm. **^{19}F NMR** (376 MHz, Chloroform-*d*) δ= -101.6 (d, J = 65.54 Hz), -111.0 (d, J = 65.57 Hz) ppm. **HRMS** (ESI): calcd. for $C_{12}H_{16}F_2NO^+$: 228.1194. found: 228.1197.

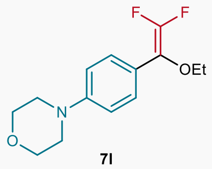

7l

4-(4-(1-Ethoxy-2,2-difluorovinyl)phenyl)morpholine (7l): colorless oil (50%). **^{1}H NMR** (400 MHz, Chloroform-*d*) δ= 7.37 (d, *J* = 8.2 Hz, 2H), 6.91 (d, *J* = 8.56 Hz, 2H), 3.86 (m, 4H), 3.76 (dd, *J* = 13.88, 6.88 Hz, 2H), 3.19 (m, 4H), 1.29 (t, *J* = 6.98 Hz, 3H) ppm. **^{13}C NMR** (101 MHz, Chloroform-*d*) δ= 155.2 (t, *J* = 289.8 Hz), 150.8, 127.5 (dd, *J* = 5.8, 3.6 Hz), 121.4 (d, *J* = 6.7 Hz), 117.6 (dd, *J* = 33.8, 18.0 Hz), 115.1, 67.4, 66.8, 48.8, 15.1 ppm. **^{19}F NMR** (376 MHz, Chloroform-*d*) δ= -100.21 (d, *J* = 62.68 Hz, 1F), -109.67 (d, *J* = 62.64 Hz, 1F) ppm. **HRMS** (ESI): calcd. for $C_{14}H_{18}F_2NO_2^+$: 270.1300. found: 270.1302.

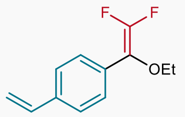

7m

1-(1-Ethoxy-2,2-difluorovinyl)-4-vinylbenzene (7m): colorless oil (40%). **^{1}H NMR** (400 MHz, Chloroform-*d*) δ= 7.43 (s, 4H)m 6.72 (dd, *J* = 17.60, 10.84 Hz, 1H), 5.78 (d, *J* = 17.56 Hz, 1H), 5.28 (d, *J* = 10.88 Hz, 1H), 3.79 (q, *J* = 7.04 Hz, 2H), 1.31 (t, *J* = 7.04 Hz, 3H) ppm. **^{13}C NMR** (101 MHz, Chloroform-*d*) δ= 155.6 (t, *J*= 292.3 Hz), 137.3, 126.2, 129.9, 126.5 (dd, *J* = 6.5, 3.6 Hz), 126.3, 114.5, 67.8, 15.1 ppm. **^{19}F NMR** (376 MHz, Chloroform-*d*) δ= -97.65 (d, *J* = 55.87 Hz, 1F), -106.85 (d, *J* = 56.14 Hz, 1F) ppm. **HRMS** (EI): calcd. for $C_{12}H_{12}F_2O$: 210.0856. found: 210.0855.

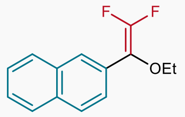

7n

2-(1-Ethoxy-2,2-difluorovinyl)naphthalene (7n): colorless oil (76%). **^{1}H NMR** (400 MHz, Chloroform-*d*) δ= 7.94 (s, 1H), 7.85-7.81 (m, 3H), 7.58 (d, *J* = 8.64 Hz, 1H), 7.48 (m, 2H), 3.83 (q, *J* = 7.00 Hz, 2H), 1.34 (t, *J* = 7.04 Hz, 3H) ppm. **^{13}C NMR** (101 MHz, Chloroform-*d*) δ= 155.8 (t, *J* = 291.0 Hz), 133.2, 132.9, 128.3, 128.2, 127.9 (d, *J* = 5.5 Hz), 127.6, 126.5, 126.4, 125.8 (t, *J* = 4.8 Hz), 123.9 (dd, *J* = 6.6, 2.2 Hz), 118.0 (dd, *J* = 32.5, 18.2 Hz), 67.9, 15.1 ppm. **^{19}F NMR** (376 MHz, Chloroform-*d*) δ= -97.41 (d, *J* = 56.63 Hz, 1F), -107.23 (d, *J* = 56.48 Hz, 1F) ppm. **HRMS** (EI): calcd. for $C_{14}H_{12}F_2O$: 234.0856. found: 234.0856.

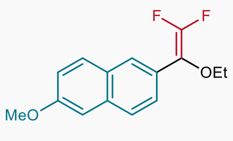

7o

2-(1-Ethoxy-2,2-difluorovinyl)-6-methoxynaphthalene (7o): colorless oil (82%). **^{1}H NMR** (400 MHz, Chloroform-*d*) δ= 7.85 (s, 1H), 7.73 (d, *J* = 8.68 Hz, 2H), 7.54 (d, *J* = 8.64 Hz, 1H), 7.16 (m, 1H), 7.12 (m, 1H), 3.92 (s, 3H), 3.82 (q, *J* = 7.08 Hz, 2H), 1.33 (t, *J* = 7.04 Hz, 3H) ppm. **^{13}C NMR** (101 MHz, Chloroform-*d*) δ= 158.2, 155.6 (t, *J* = 291.4 Hz), 134.1, 129.7, 128.6, 127.1, 125.6 (m), 124.5 (dd, *J* = 6..5, 2.5 Hz), 119.3, 118.0 (dd, *J* = 33.1, 18.2 Hz), 105.7, 67.8, 55.3, 15.1 ppm. **^{19}F NMR** (376 MHz, Chloroform-*d*) δ= -98.35 (d, *J* = 58.81 Hz, 1F), -108.15 (d, *J* = 58.69 Hz, 1F) ppm. **HRMS** (EI): calcd. for $C_{15}H_{14}F_2O_2$: 264.0962. found: 264.0977.

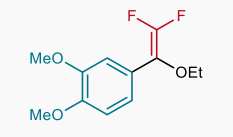

7p

4-(1-Ethoxy-2,2-difluorovinyl)-1,2-dimethoxybenzene (7p): colorless oil (78%). **^{1}H NMR** (400 MHz, Chloroform-*d*) δ= 7.04 (d, *J* = 8.36 Hz, 1H), 7.00 (s, 1H), 6.89 (d, *J* = 8.36 Hz, 1H), 3.90 (s, 6H), 3.78 (q, *J* = 7.04 Hz, 2H), 1.30 (t, *J* = 7.04 Hz, 3H) ppm. **^{13}C NMR** (101 MHz, Chloroform-*d*) δ= 155.3 (t, *J* = 290.1 Hz), 152.4, 148.9, 123.0 (d, *J* = 6.6 Hz), 119.4 (dd, *J* = 6.3, 3.7 Hz), 117.6 (dd, *J* = 33.7, 18.4 Hz), 111.1, 109.5 (dd, *J* = 5.8, 3.3 Hz), 67.6, 55.9, 15.1 ppm. **^{19}F NMR** (376 MHz, Chloroform-*d*) δ= -99.69 (d, *J* = 61.81 Hz, 1F), -109.02 (d, *J* = 61.40 Hz, 1F) ppm. **HRMS** (EI): calcd. for $C_{12}H_{14}F_2O_3$: 244.0911. found: 244.0902.

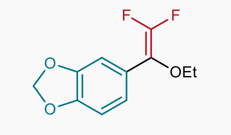

7q

5-(1-Ethoxy-2,2-difluorovinyl)benzo[d][1,3]dioxole (7q): colorless oil (74%). **^{1}H NMR** (400 MHz, Chloroform-*d*) δ= 6.96 (m, 2H), 6.83 (d, *J* = 8.04 Hz, 1H), 5.98 (s, 2H), 3.76 (q, *J* = 7.04 Hz, 2H), 1.29 (t, *J* = 7.04 Hz, 3H) ppm. **^{13}C NMR** (101 MHz, Chloroform-*d*) δ= 155.3 (t, *J* = 290.2 Hz), 147.9, 147.4, 124.3 (d, *J* = 6.6 Hz), 120.5 (dd, *J* = 6.2, 3.9 Hz), 117.6 (dd, *J* = 33.4, 18.7 Hz), 108.4, 106.9 (dd, *J* = 6.5, 3.5 Hz), 101.3, 67.6 (m), 15.0 ppm. **^{19}F NMR** (376 MHz, Chloroform-*d*) δ= -99.46 (d, *J* = 60.31 Hz, 1F), -108.62 (d, *J* = 60.99 Hz, 1F) ppm. **HRMS** (EI): calcd. for $C_{11}H_{10}F_2O_3$: 228.0598. found: 228.0590.

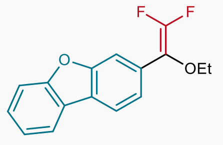

7r

3-(1-Ethoxy-2,2-difluorovinyl)dibenzo[b,d]furan (7r): colorless oil (74%). **^{1}H NMR** (400 MHz, Chloroform-*d*) δ= 8.03 (s, 1H), 7.95 (d, *J* = 7.6 Hz, 1H), 7.56 (m, 3H), 7.46 (t, *J* = 7.32 Hz, 1H), 7.34 (t, *J* = 7.4 Hz, 1H), 3.82 (q, *J* = 7.04 Hz, 2H), 1.33 (t, *J* = 7.04 Hz, 3H) ppm. **^{13}C NMR** (101 MHz, Chloroform-*d*) δ= 156.6, 155.8, 155.4 (t, *J* = 289.0 Hz), 127.6, 125.8 (dd, *J* = 5.9, 3.0 Hz), 125.2 (d, *J* = 4.9 Hz), 124.6, 123.9, 122.9, 120.8, 119.0 (dd, *J* = 5.8, 3.5 Hz), 117.8 (dd, *J* = 33.8, 18.2 Hz), 111.8 (d, *J* = 0.8 Hz), 67.6, 15.1 ppm. **^{19}F NMR** (376 MHz, Chloroform-*d*) δ= -99.29 (d, *J* = 60.99 Hz, 1F), -109.00 (d, *J* = 60.87 Hz, 1F) ppm. **HRMS** (EI): calcd. for $C_{16}H_{12}F_2O_2$: 274.0805. found: 274.0810.

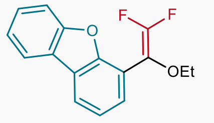

7s

4-(1-Ethoxy-2,2-difluorovinyl)dibenzo[b,d]furan (7s): colorless oil (56%). **^{1}H NMR** (400 MHz, Chloroform-*d*) δ= 7.97 (d, *J* = 7.60 Hz, 2H), 7.64 (d, *J* = 8.20 Hz, 1H), 7.52 (m, 2H), 7.39 (m, 2H), 3.79 (q, *J* = 7.04 Hz, 2H), 1.31 (t, *J* = 6.96 Hz, 3H) ppm. **^{13}C NMR** (101 MHz, Chloroform-*d*) δ= 157.7, 156.2, 153.3 (m), 127.5, 126.9 (t, *J* = 3.2 Hz), 125.0, 123.8, 123.0, 122.8, 121.2, 120.7, 114.9 (m), 13.5 (dd, *J* = 39.0, 20.5 Hz), 111.9, 67.2, 15.0 ppm. **^{19}F NMR** (376 MHz, Chloroform-*d*) δ= -98.79 (d, *J* = 54.63 Hz, 1F), -106.48 (d, *J* = 55.20 Hz, 1F) ppm. **HRMS** (EI): calcd. for $C_{16}H_{12}F_2O_2$: 274.0805. found: 274.0802.

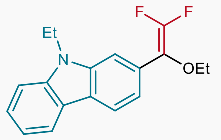

7t

2-(1-Ethoxy-2,2-difluorovinyl)-9-ethyl-9H-carbazole (7t): colorless oil (51%). **^{1}H NMR** (400 MHz, Chloroform-*d*) δ= 8.19 (s, 1H), 8.10 (d, *J* = 7.68 Hz, 1H), 7.56 (d, *J* = 8.48 Hz, 1H), 7.46 (m, 1H), 7.38 (d, *J* = 8.44 Hz, 2H), 7.23 (m, 1H), 4.32 (q, *J* = 7.16 Hz, 2H), 3.82 (q, *J* = 6.96 Hz, 2H), 1.40 (t, *J* = 7.16 Hz, 3H), 1.32 (t, *J* = 7.00 Hz, 3H) ppm. **^{13}C NMR** (101 MHz, Chloroform-*d*) δ= 156.3 (t, *J* = 288.9 Hz), 140.4, 139.7, 126.1, 124.5 (dd, *J* = 5.3, 3.1 Hz), 123.1, 122.8, 120.8 (d, *J* = 5.9 Hz), 120.6, 119.2, 119.1 (t, *J* = 4.1 Hz), 118.4 (dd, *J* = 34.1, 17.8 Hz), 108.7, 108.5, 67.4, 37.7, 15.2, 13.8 ppm. **^{19}F NMR** (376 MHz, Chloroform-*d*) δ= -100.77 (d, *J* = 64.15 Hz, 1F), -110.71 (d, *J* = 64.26 Hz, 1F) ppm. **HRMS** (ESI): calcd. for $C_{18}H_{18}F_2NO^+$: 302.1351. found: 302.1351.

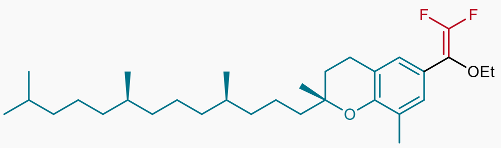

7u

(S)-6-(1-Ethoxy-2,2-difluorovinyl)-2,8-dimethyl-2-((4S,8S)-4,8,12-trimethyltridecyl)chromane (7u): colorless oil (26%). **^{1}H NMR** (400 MHz, Chloroform-*d*) δ= 7.05 (s, 1H), 6.99 (s, 1H), 3.76 (q, *J* = 7.00 Hz, 2H), 2.75 (m, 2H), 2.17 (s, 3H), 1.86-1.72 (m, 2H), 1.60-1.49 (m, 4H), 1.46-1.34 (m, 5H), 1.30-1.25 (m, 13H), 1.16-1.09 (m, 5H), 0.87-0.83 (m, 12H) ppm. **^{13}C NMR** (101 MHz, Chloroform-*d*) δ= 155.1 (t, *J* = 289.2 Hz), 152.2, 126.6 (dd, *J* = 5.5, 3.2 Hz), 126.4, 125.3 (dd, *J* = 5.3, 3.7 Hz), 120.5, 120.4, 117.8 (dd, *J* = 33.9, 17.7 Hz), 76.5, 67.3, 40.3, 39.4, 37.4 (m), 37.3, 32.8, 32.7, 31.1, 28.0, 24.8, 24.5, 24.3, 22.7, 22.6, 22.4, 21.0, 19.8, 19.7, 16.2, 15.1 ppm. **^{19}F NMR** (376 MHz, Chloroform-*d*) δ= -100.94 (d, *J* = 64.33 Hz, 1F), -110.27 (d, *J* = 64.07 Hz, 1F) ppm. **HRMS** (EI): calcd. for $C_{31}H_{50}F_2O_2$: 492.3779. found: 492.3781.

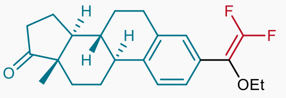

7v

(8R,9S,13S,14S)-3-(1-ethoxy-2,2-difluorovinyl)-13-methyl-6,7,8,9,11,12,13,14,15,16-decahydro-17H-cyclopenta[a]phenanthren-17-one (7v): colorless oil (60%). **^{1}H NMR** (400 MHz, Chloroform-*d*) δ= 7.32 (m, 1H), 7.27 (m, 1H), 7.20 (s, 1H), 3.78 (q, *J* = 7.04 Hz, 2H), 2.94 (m, 2H), 2.55-2.48 (m, 1H), 2.44 (m, 1H), 2.31 (m, 1H), 2.20-1.95 (m, 4H), 1.67-1.43 (m, 6H), 1.30 (t, *J* = 7.00 Hz, 3H) ppm. **^{13}C NMR** (101 MHz, Chloroform-*d*) δ= 155.5 (t, *J* = 291.1 Hz), 139.8, 137.7, 127.9 (d, *J* = 6.5 Hz), 126.9 (dd, *J* = 5.8, 3.6 Hz), 123.9 (dd, *J* = 6.2, 3.5 Hz), 117.7 (dd, *J* = 33.2, 17.9 Hz), 67.6 (t, *J* = 2.9 Hz), 50.5, 48.0, 44.4, 38.1, 35.8, 31.6, 29.5, 26.4, 25.6, 21.6, 15.1, 13.8 ppm. **^{19}F NMR** (376 MHz, Chloroform-*d*) δ= -98.53 (d, *J* = 58.51 Hz, 1F), -107.95 (d, *J* = 58.51 Hz, 1F) ppm. **HRMS** (EI): calcd. for $C_{22}H_{26}F_2O_2$: 360.1901. found: 360.1903.

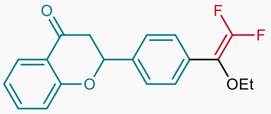

7w

2-(4-(1-Ethoxy-2,2-difluorovinyl)phenyl)chroman-4-one (7w): colorless oil (39%). **^{1}H NMR** (400 MHz, Chloroform-*d*) δ= 7.94 (d, *J* = 6.76 Hz, 1H), 7.54 (m, 5H), 7.07 (m, 2H), 5.51 (dd, *J* = 13.20, 2.72 Hz, 1H), 3.81 (q, *J* = 7.08 Hz, 2H), 3.09 (dd, *J* = 16.80, 13.28 Hz, 1H),

2.92 (dd, J = 16.84, 2.92 Hz, 1H), 1.33 (t, J = 7.00 Hz, 3H) ppm. **^{13}C NMR** (101 MHz, Chloroform-d) δ= 191.7, 161.5, 154.4 (t, J = 140.9 Hz), 138.5, 136.3, 131.2 (d, J = 7.5 Hz), 127.1, 126.7 (dd, J = 6.3, 3.7 Hz), 126.4, 121.8, 120.9, 118.1, 79.2, 67.9, 44.6, 15.1 ppm. **^{19}F NMR** (376 MHz, Chloroform-d) δ= -96.96 (d, J = 55.42 Hz, 1F), -106.51 (d, J = 54.75 Hz, 1F) ppm. **HRMS** (EI): calcd. for $C_{19}H_{16}F_2O_3$: 330.1068. found: 330.1033.

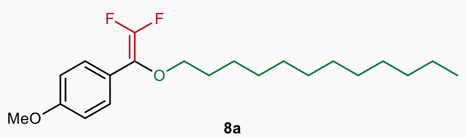

8a

1-(1-(Dodecyloxy)-2,2-difluorovinyl)-4-methoxybenzene (8a): colorless oil (89%). **^{1}H NMR** (400 MHz, Chloroform-d) δ= 7.40 (d, J = 8.68 Hz, 2H), 6.93 (d, J = 9.20 Hz, 2H), 3.83 (s, 3H), 3.68 (m, 2H), 1.67 (m, 2H), 1.40-1.28 (m, 21H) ppm. **^{13}C NMR** (101 MHz, Chloroform-d) δ= 159.4, 155.1 (t, J = 290.1 Hz), 127.9 (dd, J = 5.8, 3.6 Hz), 122.7 (d, J = 6.4 Hz), 117.9 (dd, J = 33.8, 18.0 Hz), 114.0, 72.2 (m), 55.2, 31.9, 29.7, 29.6, 29.5, 29.4, 25.8, 22.7, 14.1 ppm. **^{19}F NMR** (376 MHz, Chloroform-d) δ= -100.13 (d, J = 62.60 Hz, 1F), -109.83 (d, J = 62.60 Hz, 1F) ppm. **HRMS** (EI): calcd. for $C_{21}H_{32}F_2O_2$: 354.2370. found: 354.2375.

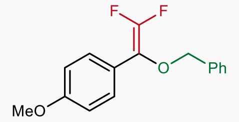

8b

1-(1-(Benzyloxy)-2,2-difluorovinyl)-4-methoxybenzene (8b): colorless oil (66%). **^{1}H NMR** (400 MHz, Chloroform-d) δ= 7.40 (d, J = 8.08 Hz, 2H), 7.35 (m, 5H), 6.94 (m, 2H), 4.71 (s, 2H), 3.83 (s, 3H) ppm. **^{13}C NMR** (101 MHz, Chloroform-d) δ= 159.5, 155.1 (t, J = 289.3 Hz), 136.6, 128.5, 128.3, 128.2, 128.1 (m), 122.3 (d, J = 6.5 Hz), 117.6 (dd, J = 34.8, 18.0 Hz), 114.1, 73.6 (m), 55.3 ppm. **^{19}F NMR** (376 MHz, Chloroform-d) δ= -99.10 (d, J = 61.14 Hz, 1F), -109.62 (d, J = 60.80 Hz, 1F) ppm. **HRMS** (EI): calcd. for $C_{16}H_{14}F_2O_2$: 276.0962. found: 276.0938.

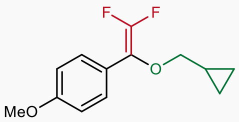

8c

1-(1-(Cyclopropylmethoxy)-2,2-difluorovinyl)-4-methoxybenzene (8c): colorless oil (84%). **^{1}H NMR** (400 MHz, Chloroform-d) δ= 7.40 (d, J = 8.16 Hz, 2H), 6.92 (d, J = 8.80 Hz, 2H), 3.82 (s, 3H), 3.53 (d, J = 7.12 Hz, 2H), 1.20-1.12 (m, 1H), 0.57 (m, 1H), 0.22 (m, 2H) ppm. **^{13}C NMR** (101 MHz, Chloroform-d) δ= 159.3,

155.2 (t, J = 289.8 Hz), 127.9 (dd, J = 5.5, 3.6 Hz), 122.7(d, J = 6.4 Hz), 117.5 (dd, J = 34.1, 18.2 Hz), 114.0, 55.3, 10.5, 3.0 ppm. **^{19}F NMR** (376 MHz, Chloroform-*d*) δ= -99.85 (d, J = 61.51 Hz, 1F), -109.61 (d, J = 62.15 Hz, 1F) ppm. **HRMS** (EI): calcd. for $C_{13}H_{14}F_2O_2$: 240.0962. found: 240.0971.

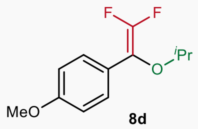

8d

1-(2,2-Bifluoro-1-isopropoxyvinyl)-4-methoxybenzene (8d): colorless oil (87%). **^{1}H NMR** (400 MHz, Chloroform-*d*) δ= 7.39 (d, J = 7.96 Hz, 2H), 6.92 (d, J = 8.80 Hz, 2H), 3.90 (m, 1H), 3.83 (s, 3H), 1.24 (d, J = 6.16 Hz, 6H) ppm. **^{13}C NMR** (101 MHz, Chloroform-*d*) δ= 159.4, 155.3 (t, J = 289.2 Hz), 128.2 (dd, J = 5.1, 3.5 Hz), 123.1 (d, J = 5.7 Hz), 115.9 (dd, J = 34.4, 17.9 Hz), 113.9, 72.6, 55.3, 21.9 ppm. **^{19}F NMR** (376 MHz, Chloroform-*d*) δ= -100.47 (d, J = 62.27 Hz, 1F), -110.42 (d, J = 62.53 Hz, 1F) ppm. **HRMS** (EI): calcd. for $C_{12}H_{14}F_2O_2$: 228.0962. found: 228.0970.

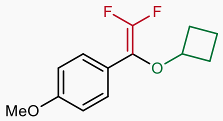

8e

1-(1-Cyclobutoxy-2,2-difluorovinyl)-4-methoxybenzene (8e): colorless oil (81%). **^{1}H NMR** (400 MHz, Chloroform-*d*) δ= 7.37 (d, J = 8.40 Hz, 2H), 6.91 (d, J = 8.76 Hz, 2H), 4.18 (m, 1H), 3.82 (s, 3H), 2.15 (m, 4H), 1.71 (m, 1H), 1.41 (m, 1H) ppm. **^{13}C NMR** (101 MHz, Chloroform-*d*) δ= 159.4, 155.3 (t, J = 289.4 Hz), 128.0 (dd, J = 3.6, 2.2 Hz), 123.0 (d, J = 6.6 Hz), 116.2 (dd, J = 34.3, 18.4 Hz), 113.9, 74.8 (m), 55.3, 30.6, 12.1 ppm. **^{19}F NMR** (376 MHz, Chloroform-*d*) δ= -99.96 (d, J = 62.98 Hz, 1F), -109.87 (d, J = 62.72 Hz, 1F) ppm. **HRMS** (EI): calcd. for $C_{13}H_{14}F_2O_2$: 240.0962. found: 240.0965.

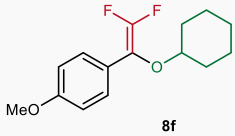

8f

1-(1-(Cyclohexyloxy)-2,2-difluorovinyl)-4-methoxybenzene (8f): colorless oil (71%). **^{1}H NMR** (400 MHz, Chloroform-*d*) δ= 7.39 (d, J = 7.64 Hz, 2H), 6.92 (d, J = 8.84 Hz, 2H), 3.82 (s, 3H), 360-3.53 (m, 1H), 1.89-1.86 (m, 2H), 1.77-1.73 (m, 2H), 1.50-1.42 (m, 3H), 1.26-1.18 (m, 3H) ppm. **^{13}C NMR** (101 MHz, Chloroform-*d*) δ= 159.3, 155.3 (t, J = 289.0 Hz), 128.2 (dd, J = 5.3, 3.3 Hz), 123.2 (d, J = 5.5 Hz), 115.7 (dd, J = 34.3, 18.2 Hz), 113.9, 78.0, 55.3, 32.0, 25.5, 23.8 ppm. **^{19}F NMR** (376 MHz, Chloroform-*d*) δ= -100.05 (d, J =

62.53 Hz), -110.29 (d, J = 62.34 Hz) ppm. **HRMS** (EI): calcd. for $C_{15}H_{18}F_2O_2$: 268.1275. found: 268.1270.

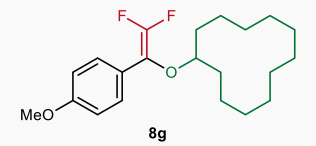

8g

((2,2-Difluoro-1-(4-methoxyphenyl)vinyl)oxy)cyclododecane (8g): white solid, melting point: 47 °C (73%). **^{1}H NMR** (400 MHz, Chloroform-*d*) δ= 8.07 (d, J = 8.84 Hz, 2H), 6.96 (d, J = 8.88 Hz, 2H), 4.11 (m, 1H), 388 (s, 3H), 1.77-1.70 (m, 2H), 1.63-1.57 (m, 2H), 1.47-1.42 (m, 2H), 1.30 (br s, 16H) ppm. **^{13}C NMR** (101 MHz, Chloroform-*d*) δ= 164.4, 162.8, 150.4 (t, J = 279.6 Hz), 132.6, 122.8 (t, J = 40.0 Hz), 120.2, 114.0, 80.9, 55.5, 29.3, 24.0, 23.7, 23.4, 23.2, 20.8 ppm. **^{19}F NMR** (376 MHz, Chloroform-*d*) δ= -116.31 (d, J = 81.52 Hz, 1F), -117.46 (d, J = 81.52 Hz, 1F) ppm. **HRMS** (ESI): calcd. for $C_{21}H_{30}F_2O_2Na^+$: 375.2106. found: 375.2108.

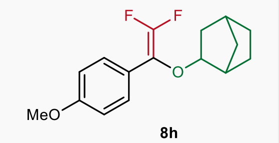

8h

(1S,4R)-2-((2,2-difluoro-1-(4-methoxy-phenyl)vinyl)oxy)bicyclo[2.2.1]heptane (8h): colorless oil (80%). **^{1}H NMR** (400 MHz, Chloroform-*d*) δ= 7.37 (d, J = 7.76 Hz, 2H), 6.92 (d, J = 8.84 Hz, 2H), 3.82 (s, 3H), 3.67 (d, J = 6.20 Hz, 1H), 2.31 (m, 2H), 1.69 (d, J = 9.72 Hz, 1H), 1.60-1.58 (m, 1H), 1.53-1.35 (m, 3H), 1.15 (d, J = 9.72 Hz, 1H), 1.00-0.95 (m. 1H), 0.87-0.82 (m, 1H) ppm. **^{13}C NMR** (101 MHz, Chloroform-*d*) δ= 159.3, 155.4 (t, J = 288.8 Hz), 128.3 (dd, J = 5.5, 3.4 Hz), 122.9 (d, J = 6.2 Hz), 116.2 (dd, J = 34.4, 18.5 Hz), 113.9, 83.1, 55.3, 40.9, 38.9, 35.4, 34.9, 28.4, 24.1 ppm. **^{19}F NMR** (376 MHz, Chloroform-*d*) δ= -100.13 (d, J = 62.79 Hz, 1F), -110.72 (d, J = 62.72 Hz, 1F) ppm. **HRMS** (EI): calcd. for $C_{16}H_{18}F_2O_2$: 280.1275. found: 280.1275.

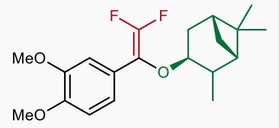

8i

(1S,3S,5R)-3-((1-(3,4-dimethoxyphenyl)-2,2-difluorovinyl)oxy)-2,6,6-trimethylbicyclo [3.1.1] heptane (8i): colorless oil (70%). **^{1}H NMR** (400 MHz, Chloroform-*d*) δ= 6.95 (m, 1H), 6.88 (m, 2H), 3.91 (s, 3H), 3.89 (s, 3H), 3.27 (s, 1H), 1.99 (m, 1H), 1.77 (m, 1H), 1.62 (m, 1H), 1.45 (m, 1H), 1.36 (d, J = 10.04 Hz, 1H), 1.08 (s, 3H), 1.05 (m, 1H), 1.02 (s, 3H), 0.86 (s, 3H) ppm. **^{13}C NMR** (101 MHz, Chloroform-*d*) δ=

153.5 (t, J = 286.0 Hz), 149.1, 148.8, 123.0, 121.1, 118.7 (dd, J = 36.7, 17.6 Hz), 110.8, 91.4, 55.9, 49.5, 48.1, 41.6, 39.8, 30.3, 26.1, 25.9, 21.0, 19.9 ppm. ^{19}**F NMR** (376 MHz, Chloroform-*d*) δ= -99.73 (d, J = 66.18 Hz, 1F), -113.71 (d, J = 65.80 Hz, 1F) ppm. **HRMS** (ESI): calcd. for $C_{20}H_{26}F_2O_3Na^+$: 375.1742. found: 375.1747.

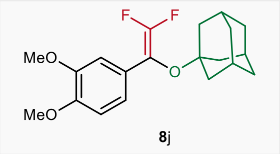

(3S,5S,7S)-1-((1-(3,4-dimethoxyphenyl)-2,2-difluorovinyl)oxy)adamantine (8j): white solid, melting point: 67 °C (76%). ^{1}**H NMR** (400 MHz, Chloroform-*d*) δ= 7.04 (m, 2H), 6.85 (m, 1H), 3.89 (s, 6H), 2.10 (s, 3H), 1.77 (s, 6H), 1.56 (m, 6H) ppm. ^{13}**C NMR** (101 MHz, Chloroform-*d*) δ= 156.1 (t, J = 290.2 Hz), 148.5, 126.9 (d, J = 6.5 Hz), 119.4 (dd, J = 5.9, 3.0 Hz), 112.5 (dd, J= 34.2, 18.6 Hz), 110.7, 110.1 (t, J = 3.9 Hz), 80.3, 55.9, 55.8, 42.7, 36.0, 30.9 ppm. ^{19}**F NMR** (376 MHz, Chloroform-*d*) δ= -96.55 (d, J = 56.48 Hz, 1F), -107.78 (d, J = 56.96 Hz, 1F) ppm. **HRMS** (ESI): calcd. for $C_{20}H_{24}F_2O_3Na^+$: 373.1586. found: 373.1582.

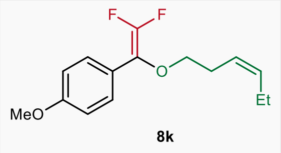

(Z)-1-(2,2-difluoro-1-(hex-3-en-1-yloxy)vinyl)-4-methoxybenzene (8k): colorless oil (70%). ^{1}**H NMR** (400 MHz, Chloroform-*d*) δ= 7.40 (d, J = 8.12 Hz, 2H), 6.92 (d, J = 8.76 Hz, 2H), 5.50 (m, 1H), 5.36 (m, 1H), 3.82 (s, 3H), 3.68 (t, J = 6.92 Hz, 2H), 2.42 (q, J = 6.88 Hz, 2H), 2.05 (m, 2H), 0.96 (t, J = 7.52 Hz, 3H) ppm. ^{13}**C NMR** (101 MHz, Chloroform-*d*) δ= 159.4, 155.1 (t, J = 289.9 Hz), 134.3, 127.9 (dd, J = 5.7, 3.5 Hz), 124.0, 122.6 (d, J = 6.6 Hz), 117.8 (dd, J = 34.0, 17.9 Hz), 114.0, 71.5 (t, J = 2.5 Hz), 55.3, 31.6, 27.7, 20.6 ppm. ^{19}**F NMR** (376 MHz, Chloroform-*d*) δ= -99.87 (d, J = 62.42 Hz, 1F), -109.68 (d, J = 62.38 Hz, 1F) ppm. **HRMS** (ESI): calcd. for $C_{15}H_{18}F_2NaO_2^+$: 291.1167. found: 291.1202.

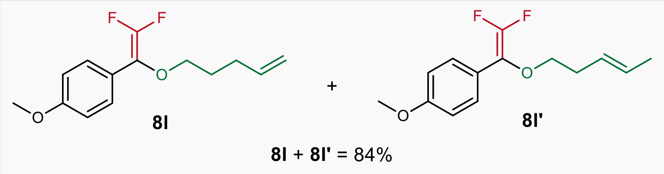

1-(2,2-Difluoro-1-(pent-4-en-1-yloxy)vinyl)-4-methoxybenzene (8l) and (E)-1-(2,2-difluoro-1-(pent-3-en-1-yloxy)vinyl)-4-

methoxybenzene (8l'): colorless oil. **^{1}H NMR** (400 MHz, Chloroform-*d*) δ= 7.04 (m, 1H), 6.98 (m, 1H), 6.89 (d, *J* = 8.40 Hz, 1H), 5.86-5.76 (m, 0.73H, from **8l**), 5.61-5.53 (m, 0.31H, from **8l'**), 5.47-5.43 (m, 0.31H, from **8l'**), 5.04 (d, *J* = 17.21 Hz, 0.75H, from **8l**), 4.98 (d, *J* = 10.07 Hz, 0.76H, from **8l'**), 3.90 (s, 3H), 3.89 (s, 3H), 3.71 (m, 2H), 2.45 (q, *J* = 6.34 Hz, 0.55H, from **8l'**), 2.20 (q, *J* = 7.39 Hz, 1.51H, from **8l**), 1.78 (m, 1.53H, from **8l**), 1.64 (d, *J* = 6.34 Hz, 0.94H, from **8l'**) ppm. **^{13}C NMR** (101 MHz, Chloroform-*d*) δ= 155.2 (t, *J* = 289.3 Hz), 149.0, 148.9, 137.7, 126.6, 125.7, 122.9 (d, *J* = 6.4 Hz), 120.8, 119.4 (dd, *J* = 5.8, 3.6 Hz), 117.9 (dd, *J* = 33.4, 18.4 Hz), 115.2, 111.1, 109.5 (m), 71.5, 55.9, 30.0, 28.9, 27.5, 12.9 ppm. **^{19}F NMR** (376 MHz, Chloroform-*d*) δ= -99.35 (d, *J* = 61.45 Hz, 0.28F), -99.53 (d, *J* = 61.45 Hz, 0.72F), -108.89 (d, *J* = 61.45 Hz, 0.37F), -109.09 (d, *J* = 0.91F) ppm. **HRMS** (ESI): calcd. for $C_{15}H_{18}F_2O_3Na^+$: 307.1116. found: 307.1110.

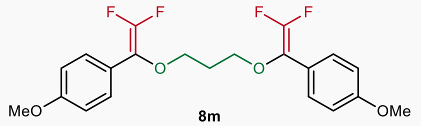

8m

1,3-Bis((2,2-difluoro-1-(4-methoxy-phenyl)vinyl)oxy)propane (8m): colorless oil (57%). **^{1}H NMR** (400 MHz, Chloroform-*d*) δ= 7.37 (d, *J* = 8.44 Hz, 4H), 6.92 (d, *J* = 8.80 Hz, 4H), 3.85 (m, 4H), 383 (s, 6H), 2.02 (m, 2H) ppm. **^{13}C NMR** (101 MHz, Chloroform-*d*) δ= 159.4, 155.0 (t, *J* = 290.0), 127.9 (dd, *J* = 5.8, 3.7 Hz), 122.4 (d, *J* = 6.3 Hz), 117.8 (dd, *J* = 34.2, 18.2 Hz), 114.0, 68.5, 55.3, 30.0 ppm. **^{19}F NMR** (376 MHz, Chloroform-*d*) δ= -99.81 (d, *J* = 62.04 Hz, 2F), -109.57 (d, *J* = 62.30 Hz, 2F) ppm. **HRMS** (EI): calcd. for $C_{21}H_{20}F_4O_4$: 412.1298. found: 412.1279.

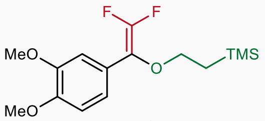

8n

(2-((1-(3,4-Dimethoxyphenyl)-2,2-difluorovi-nyl)oxy)ethyl)trimethylsilane (8n): colorless oil (68%). **^{1}H NMR** (400 MHz, Chloroform-*d*) δ= 7.03 (d, *J* = 8.44 Hz, 1H), 6.99 (s, 1H), 6.88 (d, *J* = 8.40 Hz, 1H), 3.89 (s, 3H), 3.88 (s, 3H), 3.77 (m, 2H), 1.07 (m, 2H), 0.00 (s, 9H) ppm. **^{13}C NMR** (101 MHz, Chloroform-*d*) δ= 155.4 (t, *J* = 290.2 Hz), 149.0, 148.8, 123.2 (d, *J* = 6.6 Hz), 119.4 (dd, *J* = 6.2, 3.6 Hz), 117.4 (dd, *J* = 33.2, 18.5 Hz), 111.1, 109.6 (dd, *J* = 5.8, 3.3 Hz), 69.7, 55.9, 18.4, -1.5 ppm.

^{19}**F NMR** (376 MHz, Chloroform-*d*) δ= -99.40 (d, *J* = 61.93 Hz, 1F), -108.85 (d, *J* = 61.40 Hz, 1F) ppm. **HRMS** (EI): calcd. for $C_{15}H_{22}F_2O_3Si$: 316.1306. found: 316.1301.

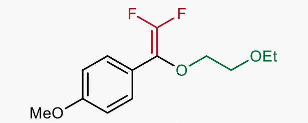

8o

1-(1-(2-Ethoxyethoxy)-2,2-difluorovinyl)-4-methoxybenzene (8o): colorless oil (77%). ^{1}**H NMR** (400 MHz, Chloroform-*d*) δ= 7.44 (d, *J* = 8.10 Hz, 2H), 6.92 (d, *J* = 8.77 Hz, 2H), 3.85 (m, 2H), 3.82 (s, 3H), 3.66 (m, 2H), 3.55 (q, *J* = 6.92 Hz, 2H), 1.24 (t, *J* = 6.92 Hz, 3H) ppm. ^{13}**C NMR** (101 MHz, Chloroform-*d*) δ= 159.4, 155.1 (t, *J* = 290.1 Hz), 127.9 (dd, *J* = 5.9, 3.7 Hz), 122.4 (d, *J* = 6.6 Hz), 118.0 (dd, *J* = 33.9, 17.7 Hz), 114.0, 71.2 (m), 69.2, 66.7, 55.3, 15.1 ppm. ^{19}**F NMR** (376 MHz, Chloroform-*d*) δ= -99.41 (d, *J* = 61..48 Hz, 1F), -109.32 (d, *J* = 61.93 Hz, 1F) ppm. **HRMS** (ESI): calcd. for $C_{13}H_{16}F_2O_3Na^+$: 281.0960. found: 281.0956.

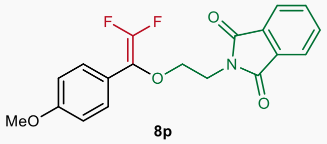

8p

2-(2-((2,2-Difluoro-1-(4-methoxy-phenyl)vinyl)oxy)ethyl)isoindoline-1,3-dione (8p): white solid, melting point: 40 °C (43%). ^{1}**H NMR** (400 MHz, Chloroform-*d*) δ= 7.86 (m, 2H), 7.73 (m, 2H), 7.29 (d, *J* = 8.44 Hz, 2H), 6.80 (d, *J* = 8.76 Hz, 2H), 3.98 (m, 4H), 3.78 (s, 3H) ppm. ^{13}**C NMR** (101 MHz, Chloroform-*d*) δ= 168.1, 159.5, 154.8 (t, *J* = 290.1 Hz), 134.0, 132.0, 127.9 (m), 123.3, 121.9 (d, *J* = 6.0 Hz), 118.0 (dd, *J* = 35.4, 18.3 Hz), 114.0, 68.7, 55.2, 37.7 ppm. ^{19}**F NMR** (376 MHz, Chloroform-*d*) δ= -99.10 (d, *J* = 61.40 Hz, 1F), -109.55 (d, *J* = 61.29 Hz, 1F) ppm. **HRMS** (ESI): calcd. for $C_{19}H_{15}F_2NNaO_4^+$: 382.0861. found: 382.0865.

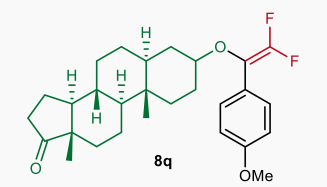

8q

(5S,8R,9S,10S,13S,14S)-3-((2,2-difluoro-1-(4-methoxyphenyl)vinyl)oxy)-10,13-dimethylhexadecahydro-17H-cyclopenta[a]phenanthren-17-one (8q): white solid, melting point: 81 °C (75%). ^{1}**H NMR** (400 MHz, Chloroform-*d*) δ= 7.34 (d, *J* = 8.12 Hz, 2H), 6.89 (d, *J* = 8.72 Hz, 2H), 3.79 (m, 4H), 2.45-2.38 (m, 1H), 2.10-2.01 (m, 1H), 1.96-1.89 (m, 1H), 1.80-1.75 (m, 3H), 1.68-1.67 (m, 2H), 1.56-1.37 (m, 8H), 1.33-1.24 (m, 6H), 1.21-1.17 (m, 1H),

1.09-1.02 (m, 1H), 0.84 (m, 6H), 0.77 (s, 3H) ppm. **^{13}C NMR** (101 MHz, Chloroform-*d*) δ= 159.4, 155.2 (t, *J* = 288.3 Hz), 128.4 (m), 122.8 (d, *J* = 5.9 Hz), 115.9 (dd, *J* = 34.8, 18.2 Hz), 113.9, 74.6, 55.3, 54.4, 51.4, 47.8, 39.6, 35.9, 35.8, 35.1, 33.0, 32.6, 31.6, 30.8, 28.2, 25.6, 21.8, 20.1, 13.8, 11.3 ppm. **^{19}F NMR** (376 MHz, Chloroform-*d*) δ= -100.47 (d, *J* = 63.09 Hz, 1F), -110.85 (d, *J* = 63.62 Hz, 1F) ppm. **HRMS** (ESI): calcd. for $C_{28}H_{40}NF_2O_3^+$: 476.2971. found: 476.2967.

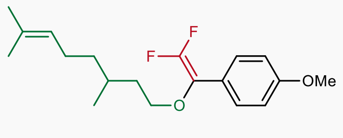

8r

1-(1-((3,7-Dimethyloct-6-en-1-yl)oxy)-2,2-difluorovinyl)-4-methoxybenzene (8r): colorless oil (79%). **^{1}H NMR** (400 MHz, Chloroform-*d*) δ= 7.39 (d, *J* = 8.00 Hz, 2H), 6.92 (d, *J* = 8.84 Hz, 2H), 5.09 (t, *J* = 7.08 Hz, 1H), 3.82 (s, 3H), 3.73-3.69 (m, 2H), 2.04-1.92 (m, 2H), 1.77-1.70 (m, 1H), 1.68 (s, 3H), 1.63 (m, 1H), 1.60 (s, 3H), 1.52-1.43 (m, 1H), 1.39-1.30 (m, 1H), 1.21-1.11 (m, 1H), 0.89 (d, *J* = 6.56 Hz, 3H) ppm. **^{13}C NMR** (101 MHz, Chloroform-*d*) δ= 159.4, 155.1 (t, *J* = 289.8 Hz), 131.3, 127.9 (dd, *J* = 5.8, 3.5 Hz), 124.7, 122.7 (d, *J* = 6.6 Hz), 117.9 (dd, *J* = 33.9, 18.0 Hz), 114.0, 70.4, 55.3, 37.1, 36.7, 29.2, 25.7, 25.4, 19.4, 17.6 ppm. **^{19}F NMR** (376 MHz, Chloroform-*d*) δ= -100.03 (d, *J* = 62.57 Hz, 1F), -109.82 (d, *J* = 62.57 Hz, 1F) ppm. **HRMS** (EI): calcd. for $C_{19}H_{26}F_2O_2$: 324.1901. found: 324.1914.

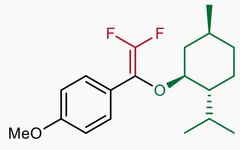

8s

1-(2,2-Difluoro-1-(((1S,2R,5S)-2-isopropyl-5-methylcyclohexyl)oxy)vinyl)-4-methoxybenzene (8s): colorless oil (80%). **^{1}H NMR** (400 MHz, Chloroform-*d*) δ= 7.37 (d, *J* = 8.28 Hz, 2H), 6.91 (d, *J* = 7.4 Hz, 2H), 3.82 (s, 3H), 3.38 (m, 1H), 2.44 (m, 1H), 1.97 (d, *J* = 11.68 Hz, 1H), 1.65-1.58 (m, 2H), 1.42 (m, 1H), 1.28-1.20 (m, 2H), 0.95 (m, 3H), 0.92 (m, 2H), 0.88 (m, 3H), 0.72 (m, 3H) ppm. **^{13}C NMR** (101 MHz, Chloroform-*d*) δ= 159.5, 155.0 (t, *J* = 288.1 Hz), 128.8 (t, *J* = 3.7 Hz), 123.0 (d, *J* = 5.8 Hz), 115.2 (dd, *J* = 35.3, 17.7 Hz), 113.8, 78.3, 55.3, 48.2, 39.9, 34.3, 31.2, 25.1, 22.8, 22.2, 21.2, 15.9 ppm. **^{19}F NMR** (376 MHz, Chloroform-*d*) δ= -100.71 (d, *J* = 64.11 Hz, 1F), -110.94 (d, *J* = 64.15 Hz, 1F) ppm. **HRMS** (EI): calcd. for $C_{19}H_{26}F_2O_2$: 324.1901. found: 324.1910.

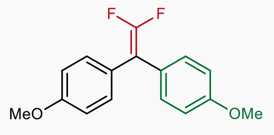

9a

4,4'-(2,2-Difluoroethene-1,1-diyl)bis(methoxybenzene) (9a): white solid, melting point: 36 ºC (65%). **^{1}H NMR** (400 MHz, Chloroform-*d*) δ= 7.18 (d, *J* = 8.36 Hz, 4H), 6.87 (d, *J* = 8.64 Hz, 4H), 3.80 (s, 6H) ppm. **^{13}C NMR** (101 MHz, Chloroform-*d*) δ= 158.9, 153.5 (t, *J* = 292.9 Hz), 130.7, 126.8, 113.8, 95.2 (t, *J* = 18.2 Hz), 55.3 ppm. **^{19}F NMR** (376 MHz, Chloroform-*d*) δ= -89.77 ppm. **HRMS** (EI): calcd. for $C_{16}H_{14}F_2O_2$: 276.0962. found: 276.0979.

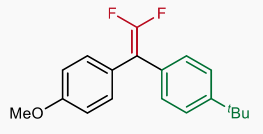

9b

1-(tert-butyl)-4-(2,2-difluoro-1-(4-methoxyphenyl)vinyl)benzene (9b): colorless oil (67%). **^{1}H NMR** (400 MHz, Chloroform-*d*) δ= 7.37 (d, *J* = 8.45 Hz, 2H), 7.21 (d, *J* = 7.74 Hz, 4H), 6.90 (d, *J* = 8.80 Hz, 2H), 3.83 (s, 3H), 1.34 (s, 9H) ppm. **^{13}C NMR** (101 MHz, Chloroform-*d*) δ= 158.9, 153.6 (t, *J* = 292.0 Hz), 150.4, 131.5 (m), 130.9 (m), 129.1 (m), 126.7 (m), 125.3, 113.8, 95.5 (t, *J* = 18.2 Hz), 55.3, 34.6, 31.3 ppm. **^{19}F NMR** (376 MHz, Chloroform-*d*) δ= -89.84 (d, *J* = 36.36 Hz, 1F), -89.13 (d, *J* = 35.38 Hz, 1F) ppm. **HRMS** (ESI): calcd. for $C_{19}H_{21}F_2O^+$: 303.1555. found: 303.1554.

5. Mechanistic studies

5.1 ^{19}F NMR spectra of 1a, 3a and 7c

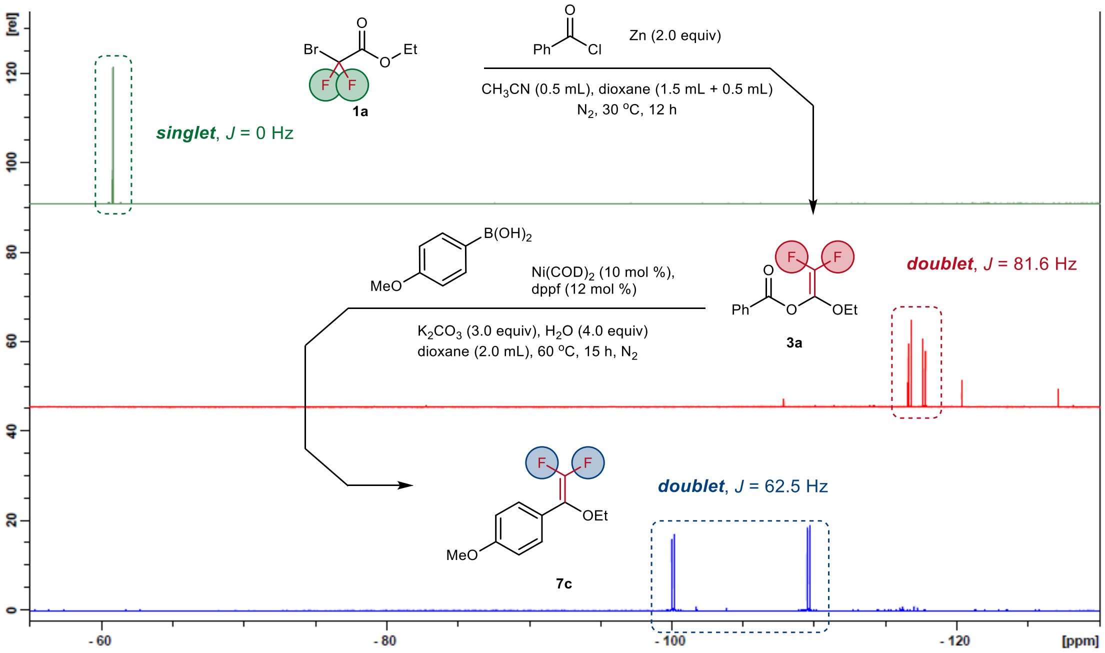

The transformations from **1a** → **3a** → **7c** can be traced easily by ^{19}F NMR analysis of their crude mixtures.

Bromodifluoroacetate **1a** displays a singlet signal at **δ= -60.12 (s, 2F) ppm**. Upon the addition of benzoyl chloride and zinc, the singlet signal disappeared with emergence of a new doublet signals at δ= -116.13 (d, J = 81.63 Hz, 1F), -117.15 (d, J = 81.59 Hz, 1F) ppm attributed by 1-ethoxy-2,2-difluorovinyl benzoate **3a**.

Further addition of Ni(COD)$_2$ catalyst and 4-methoxyphenylboronic acid resulted in disappearance of the doublets with the emergence of a new doublet signal at δ= -100.11 (d, J = 62.53 Hz, 1F), -109.62 (d, J = 62.45 Hz, 1F) ppm, which is attributed to (1-(1-ethoxy-2,2-difluorovinyl)-4-methoxybenzene, **7c**).

5.2 Effects of leaving group on the BzO-DFs 3 and 7c formation

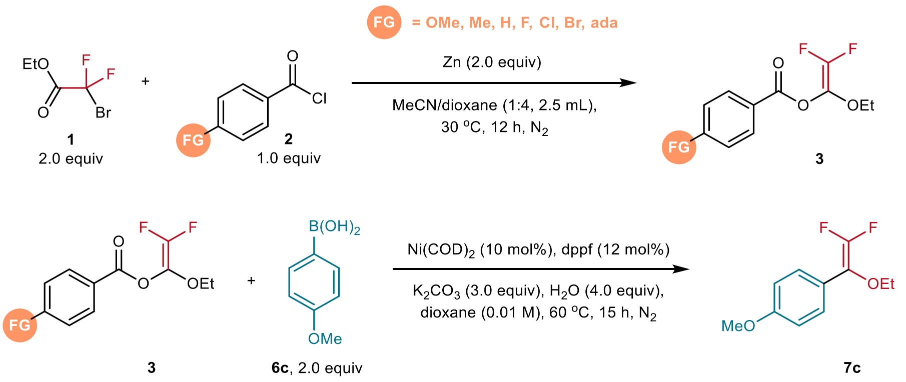

Next, we investigated the effects of p*K*a of the leaving groups on the construction of BzO-DFs and the corresponding coupling with boronic acids. The effects of p*K*a range for the synthesis of **3** and **7c** was found to be 3.97–4.57.

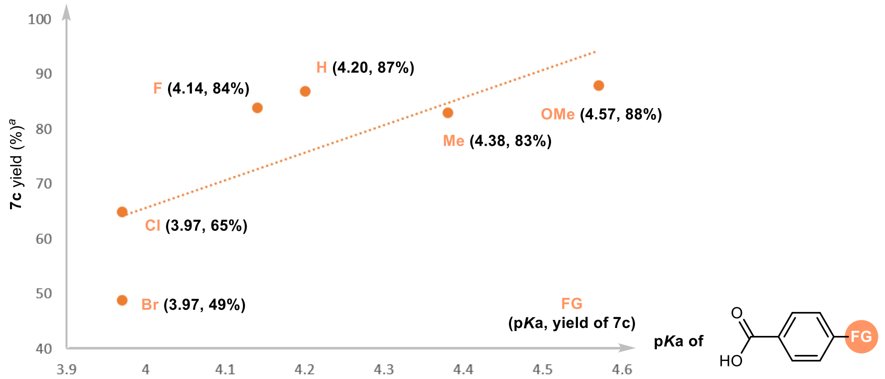

Supplementary Table 3. Effects of leaving group on the 3 and 7c formation

FG	p*K*a of FG-$C_6H_4CO_2H$	yield of **3** (%)	yield of **7c** (%)
OMe	4.57	72	88
Me	4.38	82	83
H	4.20	77	87
F	4.14	66	84
Cl	3.97	64	65
Br	3.97	45	49
ada	4.86	78	<5

Reactions were performed according to the general procedure mentioned above. Yields were determined by ^{19}F NMR with trifluorotoluene as internal standard.

5.3 Competitive experiments – Relative reactivity of the benzoate leaving group

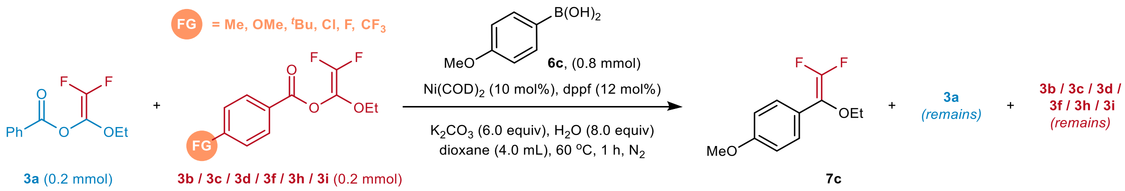

To a 8 mL vial equipped with a magnetic stir, **3a** (0.2 mmol) paired with an equimolar quantity of **3b / 3c / 3d / 3f / 3h / 3i** (0.2 mmol) was treated with **6c** (0.8 mmol), K_2CO_3 (6.0 equiv, 1.2 mmol) and de-ionized water (8.0 equiv, 1.6 mmol) was mixed in a glovebox. Then a dioxane solution (0.01 M, 4.0 mL) of $Ni(COD)_2$ (10 mol%, 0.02 mmol) and dppf (12 mol%, 0.024 mmol) was transferred to the vial containing the substrates and reagents inside the glovebox. The reaction mixture was then stirred for 1 hour at 60 °C. To work-up, the reaction mixture was filtered through a pad of Celite® and the filtrate was concentrated in *vacuo*. $PhCF_3$ (0.067 mmol, 8.2 μL. -63.72 ppm) was then added as internal standard for ^{19}F NMR analysis with $CDCl_3$ as solvent.

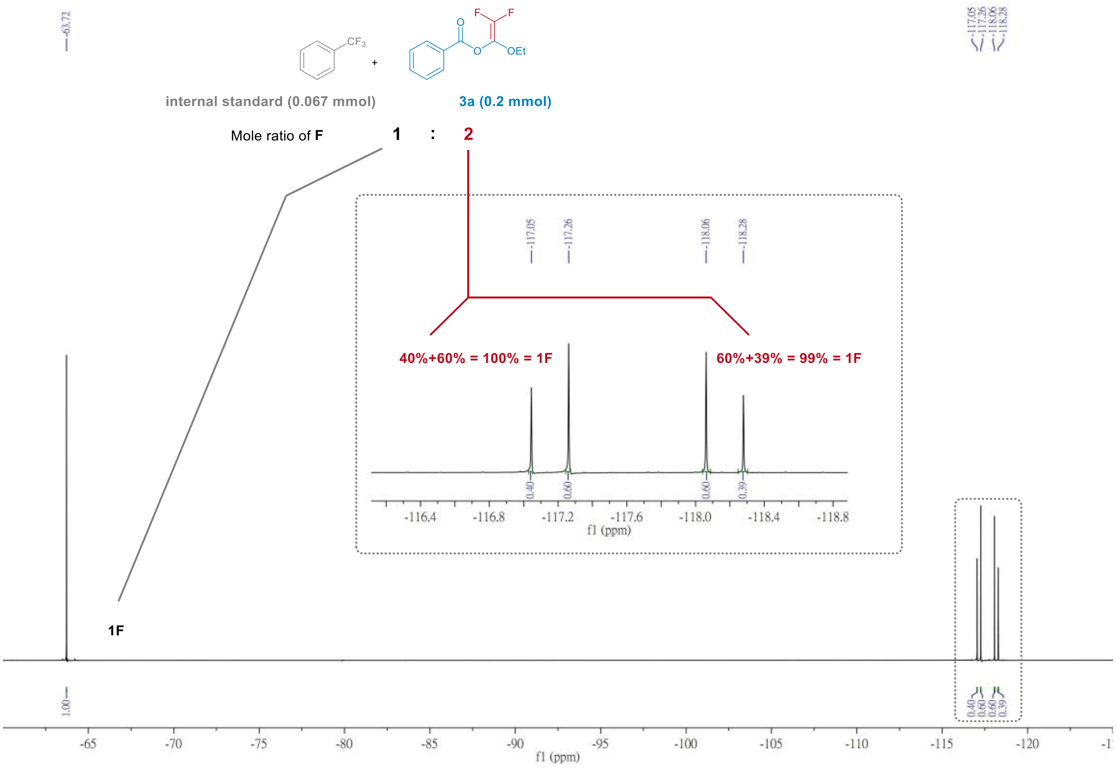

Four sets of competition reaction: [**3a**+**3b**], [**3a**+**3d**], [**3a**+**3f**], [**3a**+**3h**] were performed in triplicate, and the yields of the remaining substrates were determined by ^{19}F NMR with $PhCF_3$ (0.067 mmol, 8.2 μL) as an internal standard (-63.72 ppm).

Derivation of the equation used for calculating relative rate information:

For the reaction between *n* and *i* competing reactants s_1, s_2, …s_i, the rate equation related to a given species *i* is:

$$\frac{d_{s_i}}{d_t} = k_i s_i [n_{t=0} - (s_{1,t=0} - s_1) - (s_{2,t=0} - s_2) \ldots + (s_{i,t=0} - s_i)]$$

Partially integrated form:

$$\ln\left(\frac{s_i}{s_{i,t=0}}\right) = k_i \int [n_{t=0} - (s_{1,t=0} - x_1) - (s_{2,t=0} - s_2) \ldots + (s_{i,t=0} - s_i)] dt$$

Solving the equation for two competing species FG and H, the following expression can be obtained for the calculation of relative rate information based on the amount of starting material left:

$$\frac{k_{\mathrm{FG}}}{k_{\mathrm{H}}} = \frac{\ln\left({s^{\mathrm{FG}}}_{\mathrm{x}} / {s^{\mathrm{FG}}}_{\mathrm{x,t=0}}\right)}{\ln\left({s^{\mathrm{H}}}_{\mathrm{x}} / {s^{\mathrm{H}}}_{\mathrm{x,t=0}}\right)}$$

${s^{\mathrm{FG}}}_{\mathrm{x}}$ = % yield of **3b / 3d / 3f/ 3h** remaining after 1 h of the reaction

${s^{\mathrm{FG}}}_{\mathrm{x,t=0}}$ = % yield of **3b / 3d / 3f / 3h** initially = 100%

${s^{\mathrm{H}}}_{\mathrm{x}}$ = % yield of **3a** remaining after 1 h of the reaction

${s^{\mathrm{H}}}_{\mathrm{x,t=0}}$ = % yield of **3a** initially = 100%

To perform the free-energy study, the relative rate values were fitted into the following Hammett equation:

$$\log\left(\frac{k_{\mathrm{FG}}}{k_{\mathrm{H}}}\right) = \rho\sigma$$

ρ = reaction constant, σ = Hammett substituent constant (*para*)

- ^{19}F NMR spectra of pure **3a** and pure **3b**

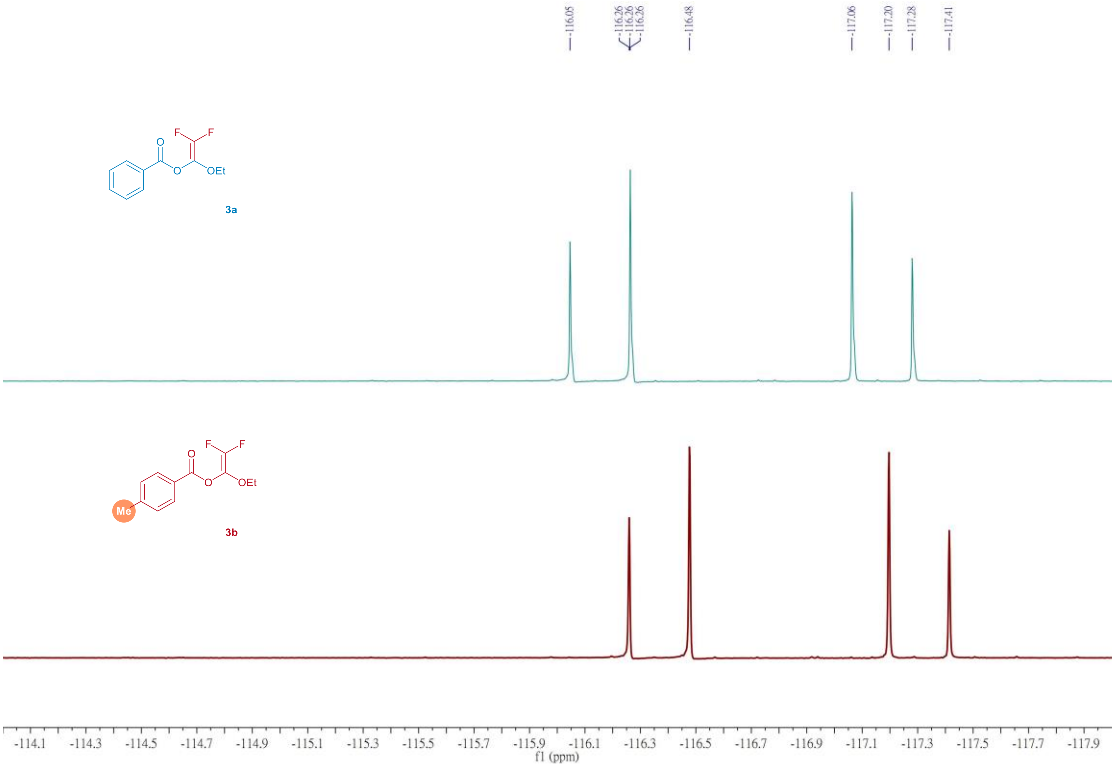

- ^{19}F NMR spectra of **(reaction mixture "3a + 3b" – trial 1)**

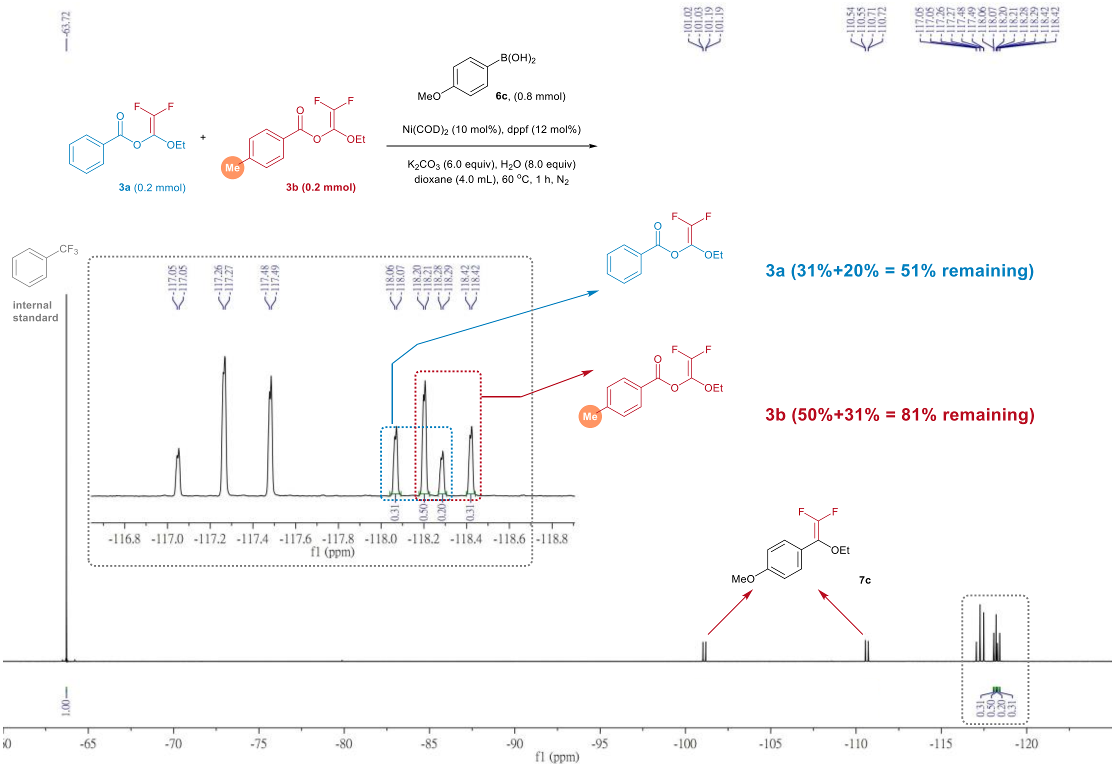

- ^{19}F NMR spectra of **(reaction mixture "3a + 3b" – trial 2)**

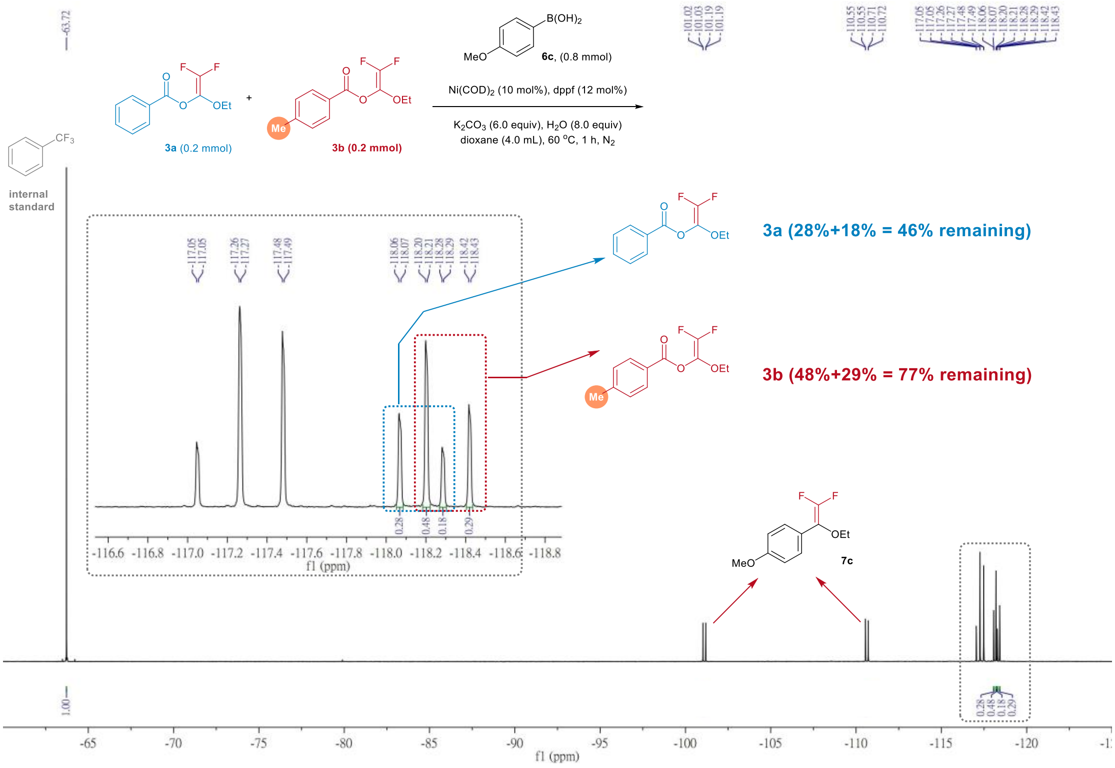

- ^{19}F NMR spectra of **(reaction mixture "3a + 3b" – trial 3)**

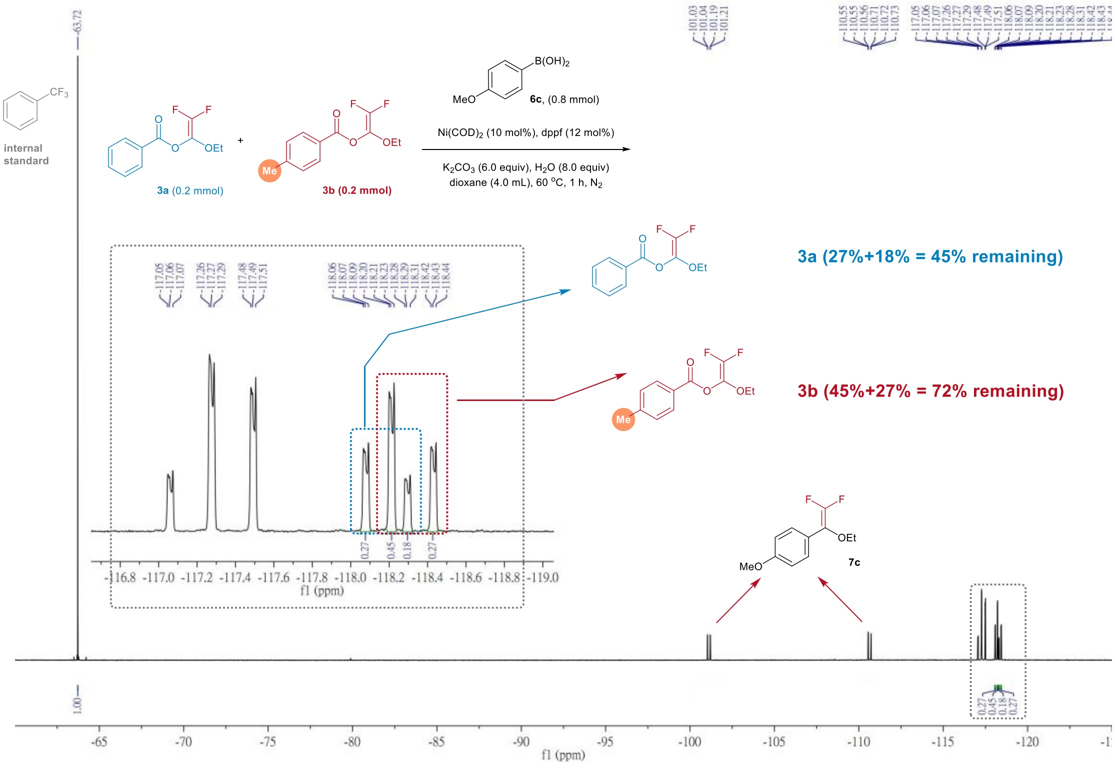

- ^{19}F NMR spectra of pure **3a** and pure **3c**

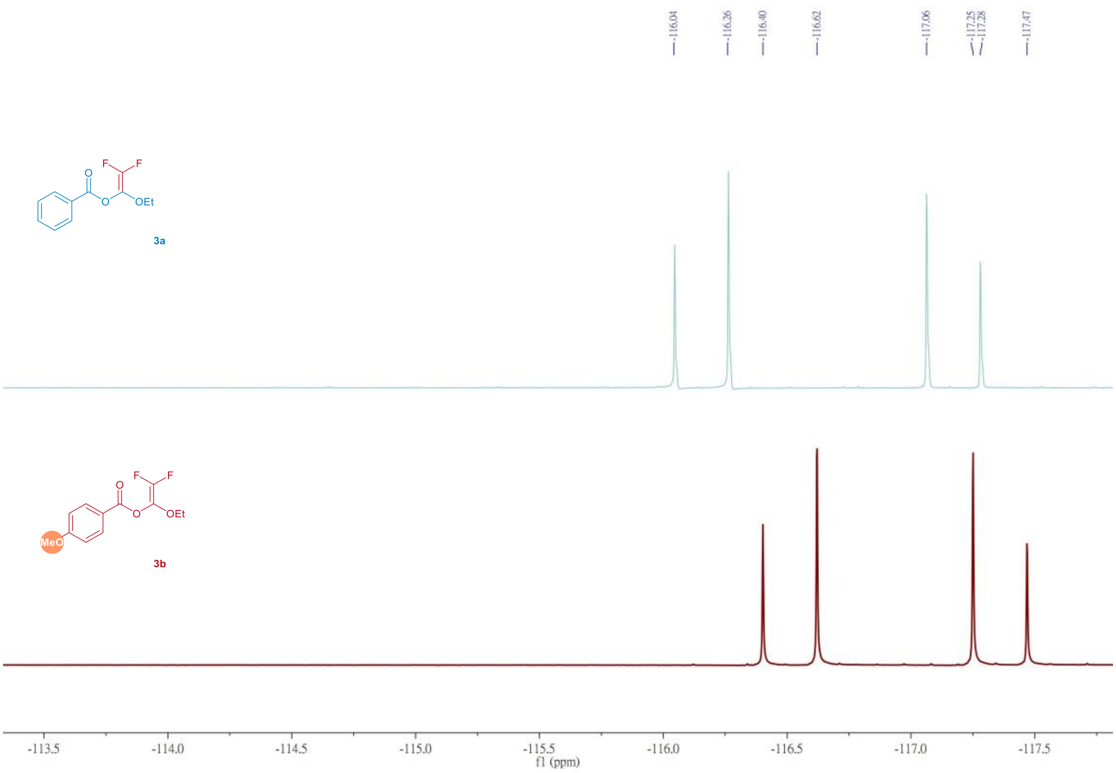

- ^{19}F NMR spectra of **(reaction mixture "3a + 3c" – trial 1)**

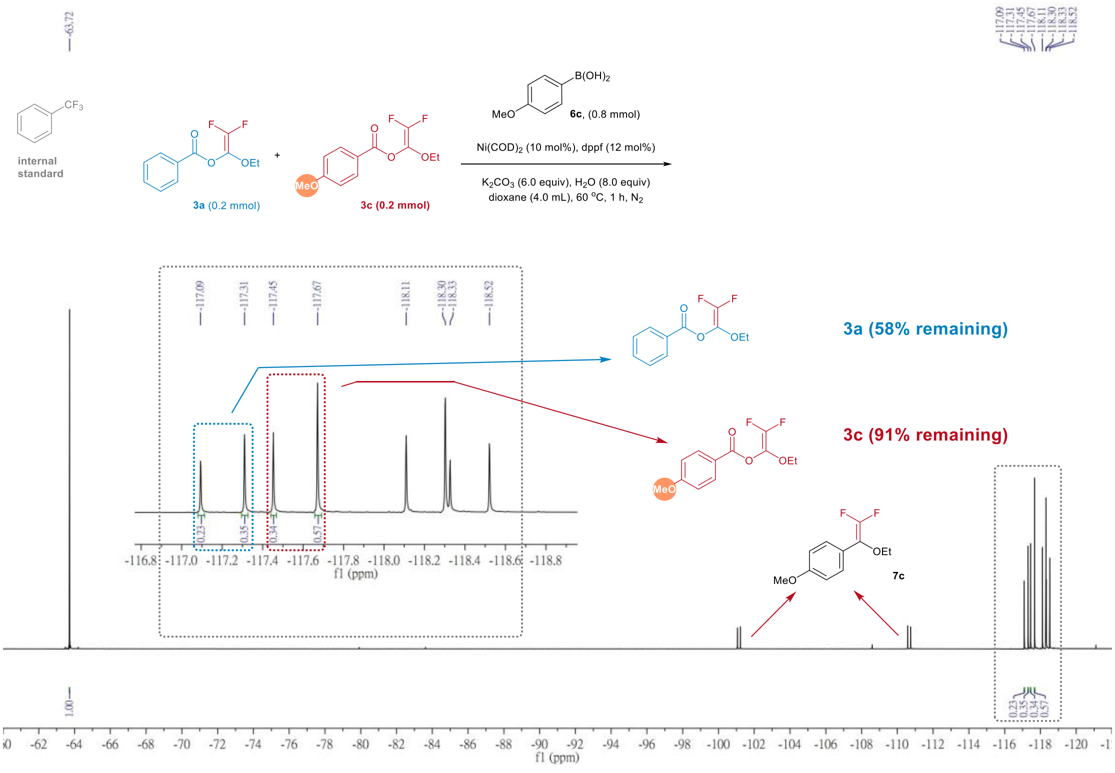

- ^{19}F NMR spectra of **(reaction mixture "3a + 3c" – trial 2)**

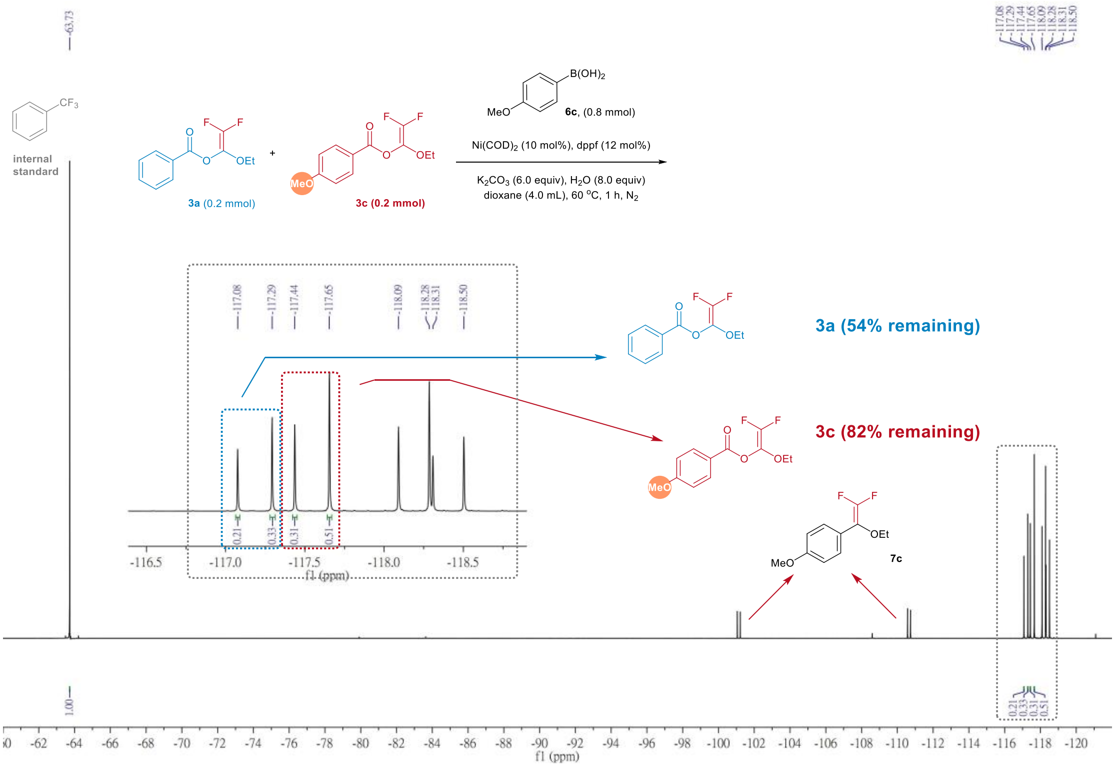

- ^{19}F NMR spectra of **(reaction mixture "3a + 3c" – trial 3)**

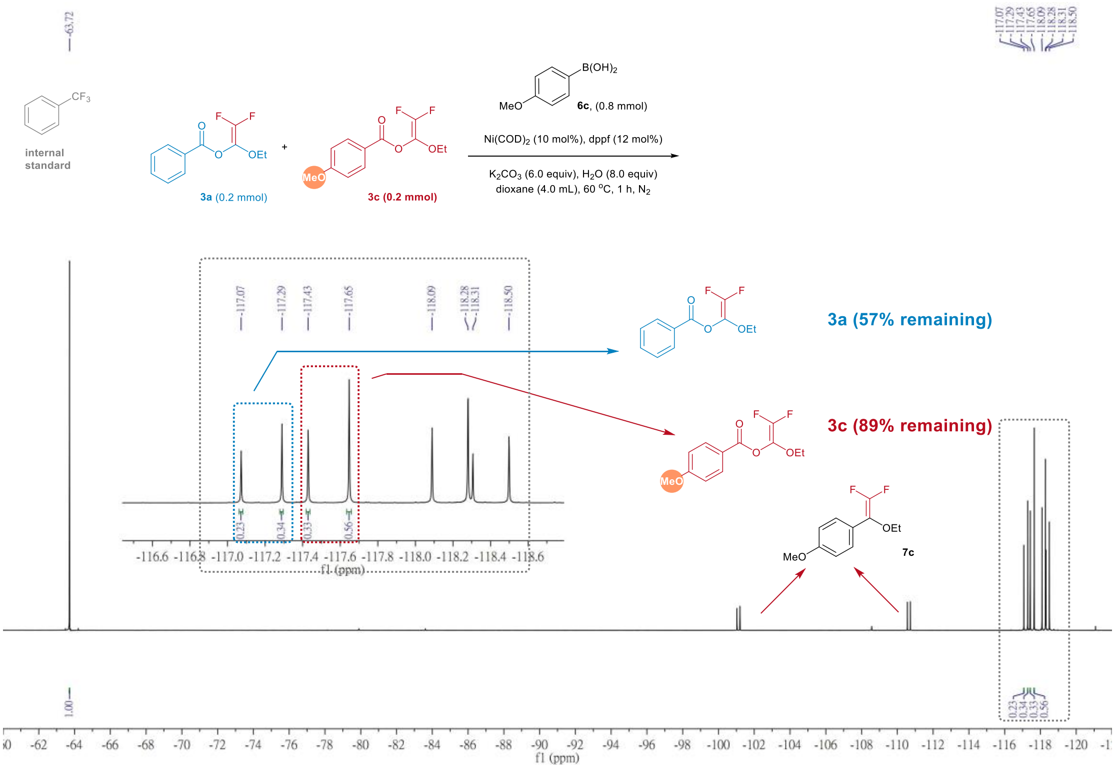

- ^{19}F NMR spectra of pure **3a** and pure **3d**

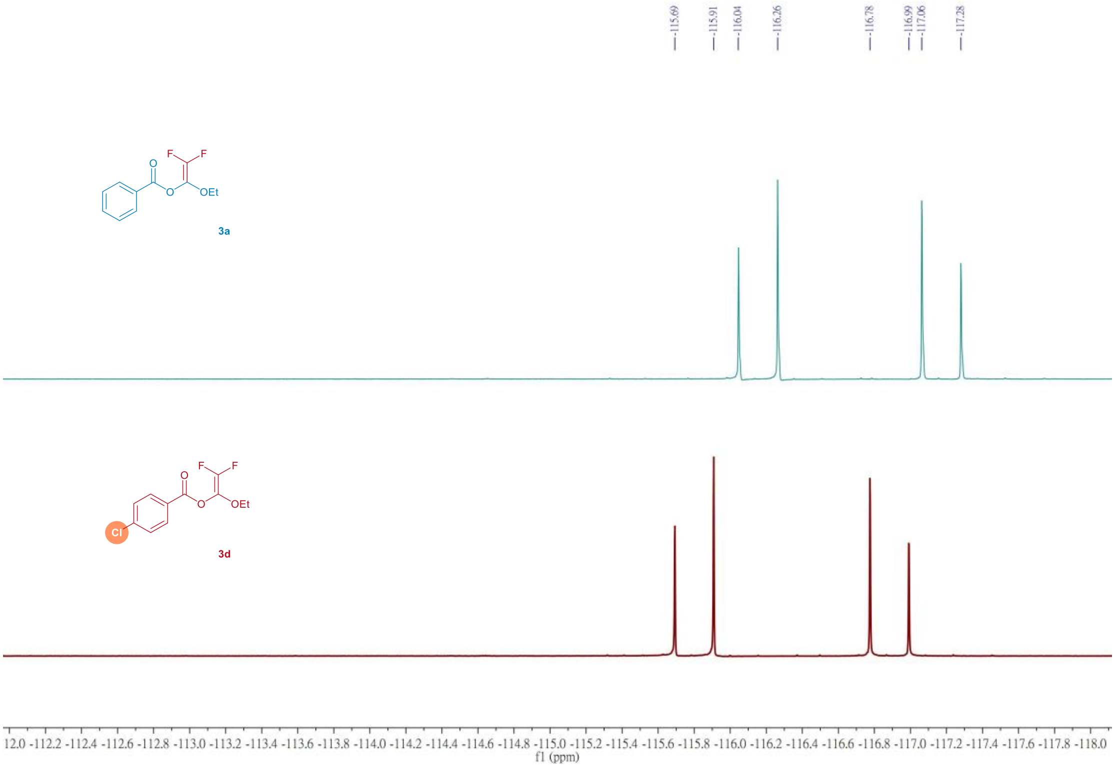

- ^{19}F NMR spectra of **(reaction mixture "3a + 3d" – trial 1)**

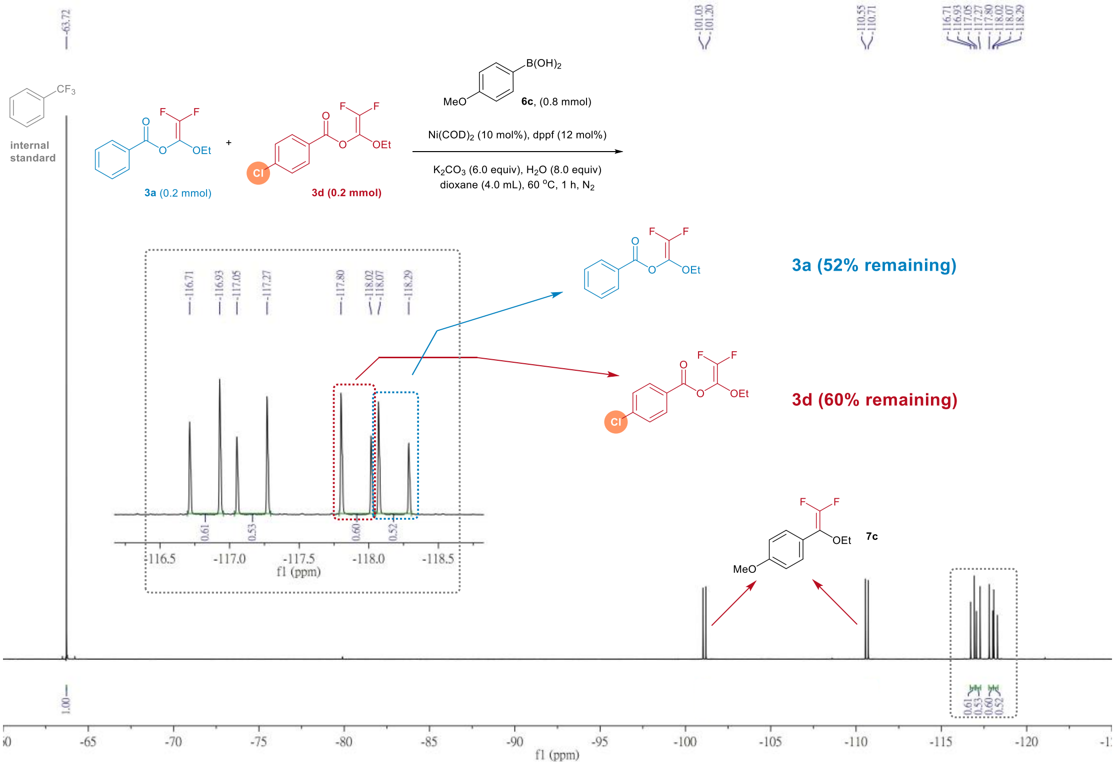

- ^{19}F NMR spectra of **(reaction mixture "3a + 3d" – trial 2)**

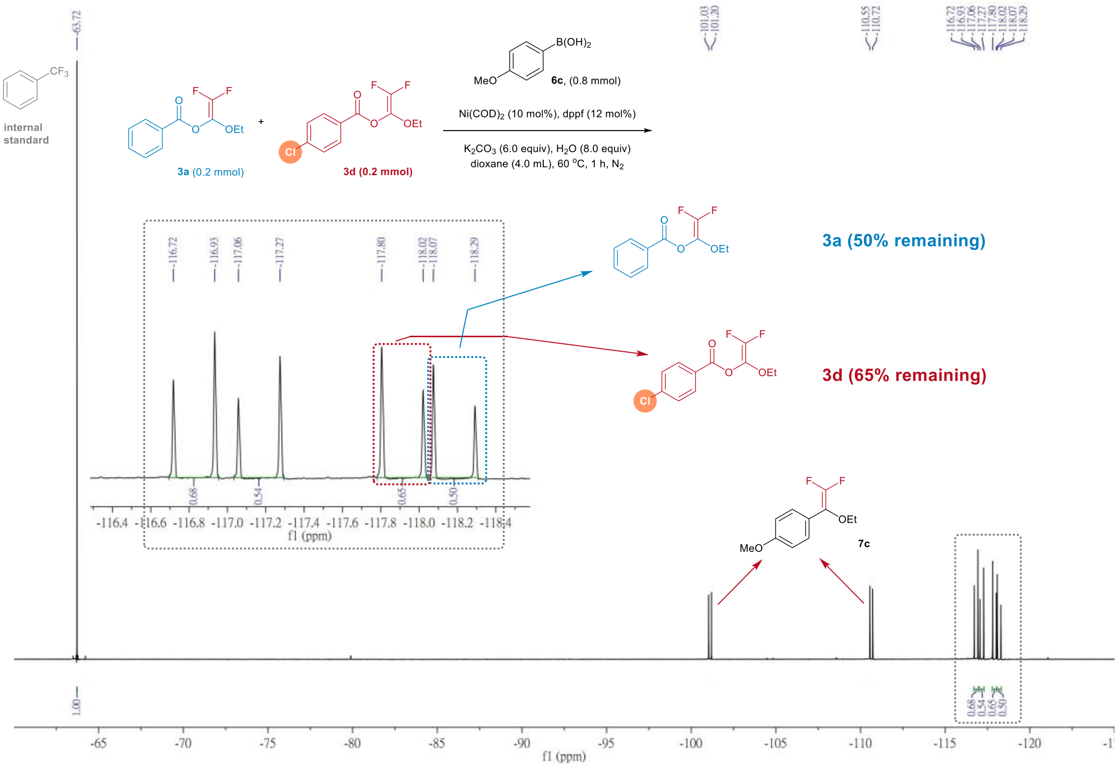

- ^{19}F NMR spectra of **(reaction mixture "3a + 3d" – trial 3)**

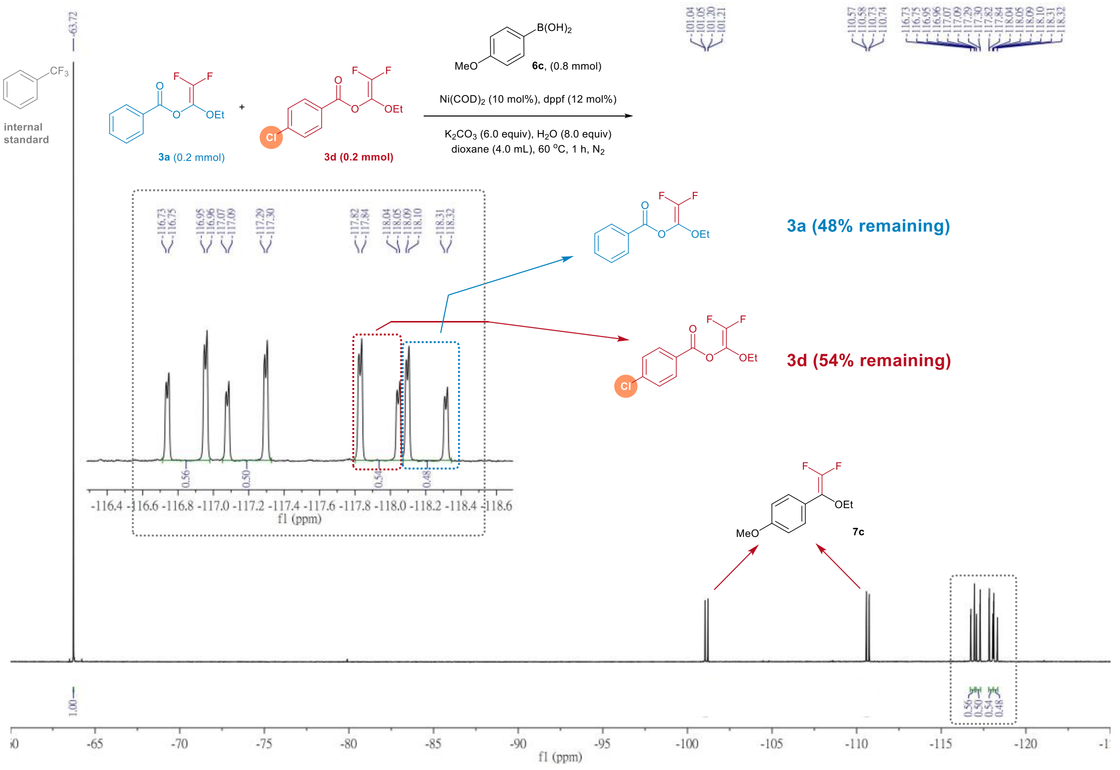

- ^{19}F NMR spectra of pure **3a** and pure **3f**

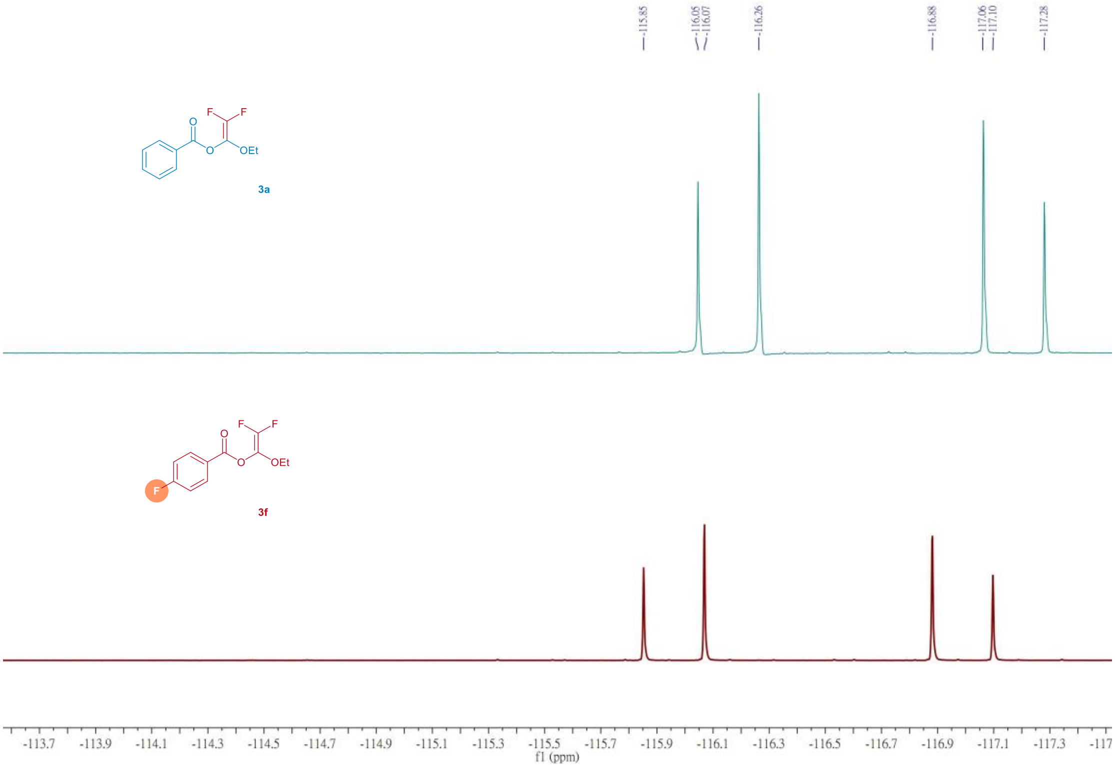

- ^{19}F NMR spectra of **(reaction mixture "3a + 3f" – trial 1)**

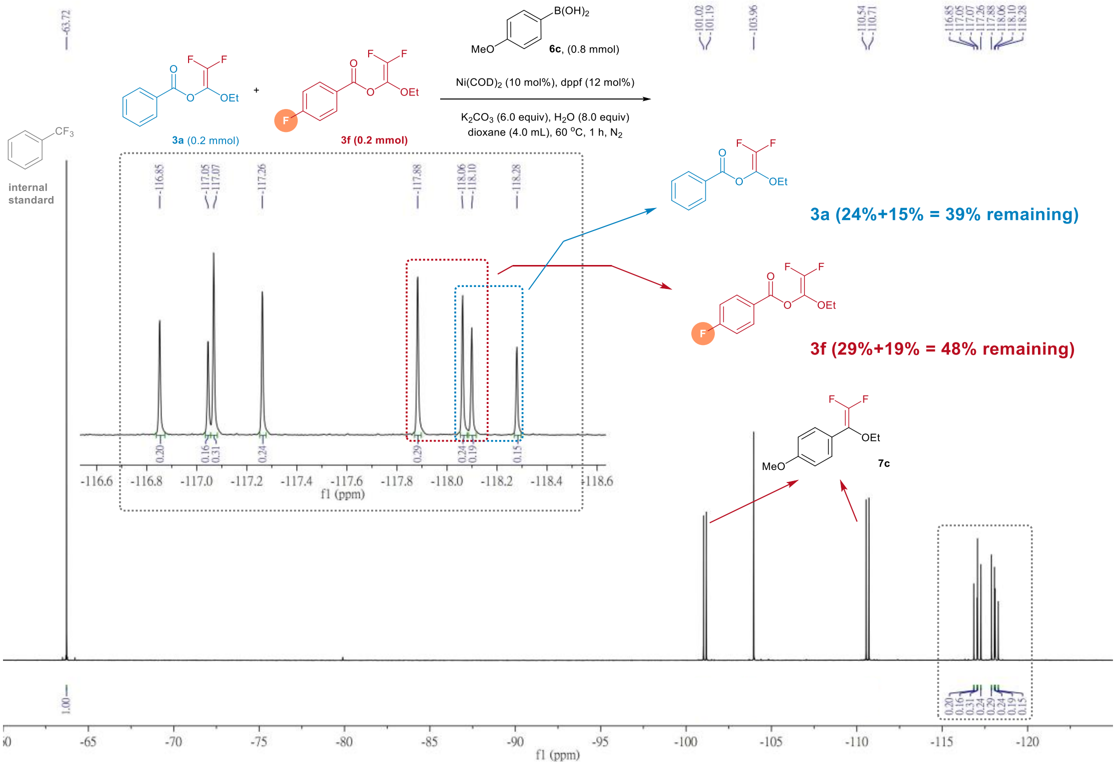

- ^{19}F NMR spectra of **(reaction mixture "3a + 3f" – trial 2)**

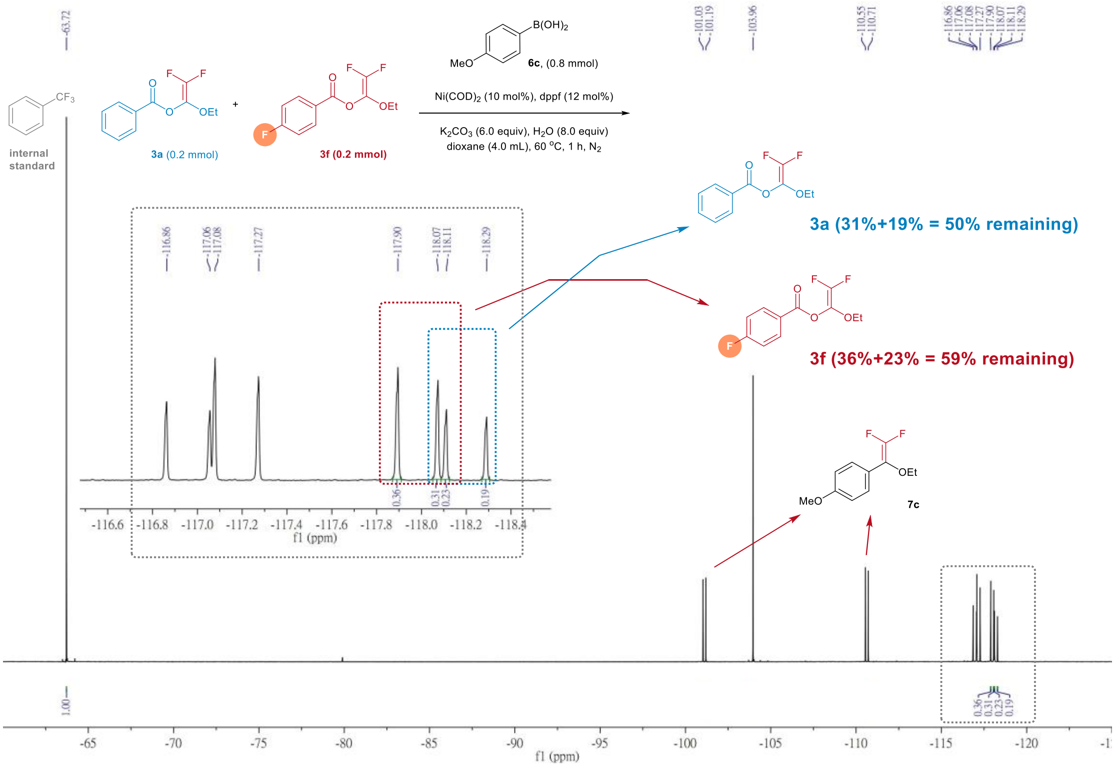

- ^{19}F NMR spectra of **(reaction mixture "3a + 3f" – trial 3)**

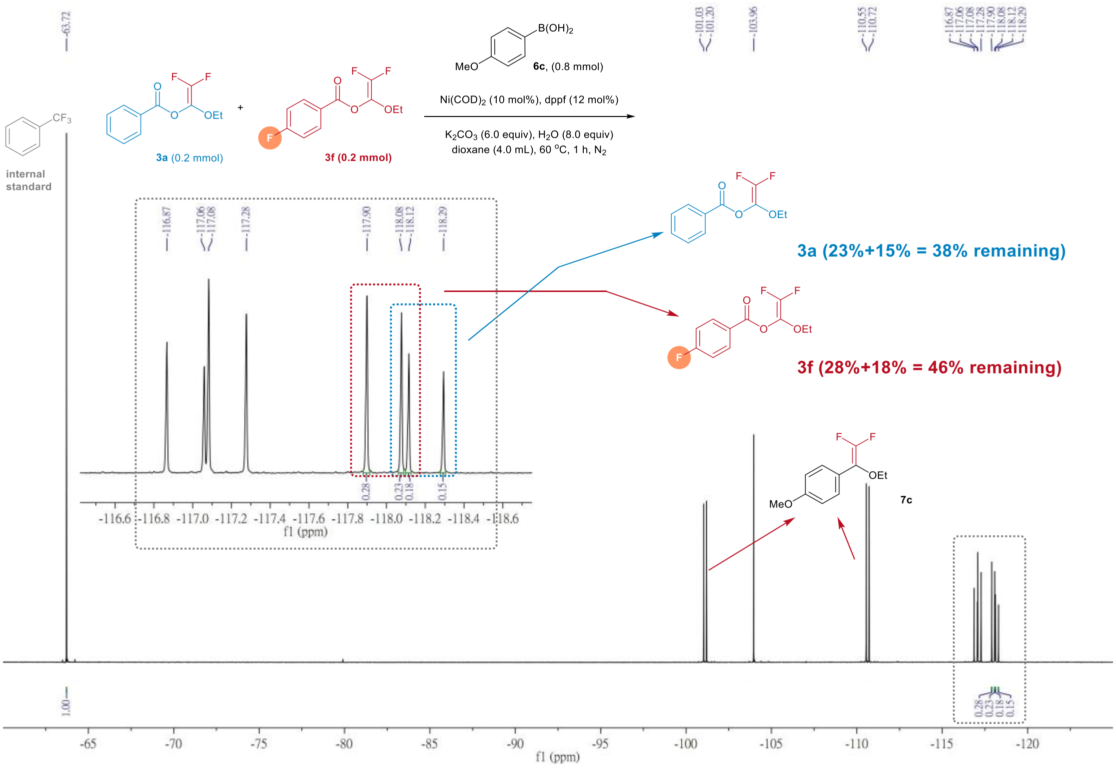

- ^{19}F NMR spectra of pure **3a** and pure **3h**

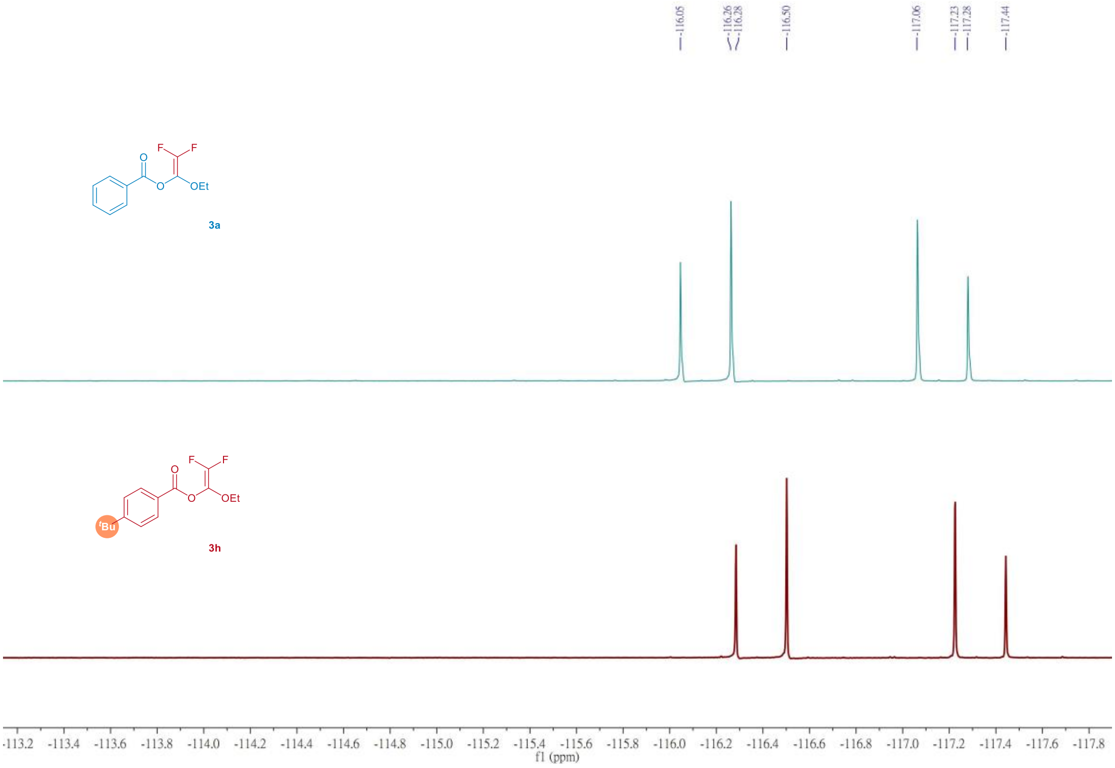

- ^{19}F NMR spectra of **(reaction mixture "3a + 3h" – trial 1)**

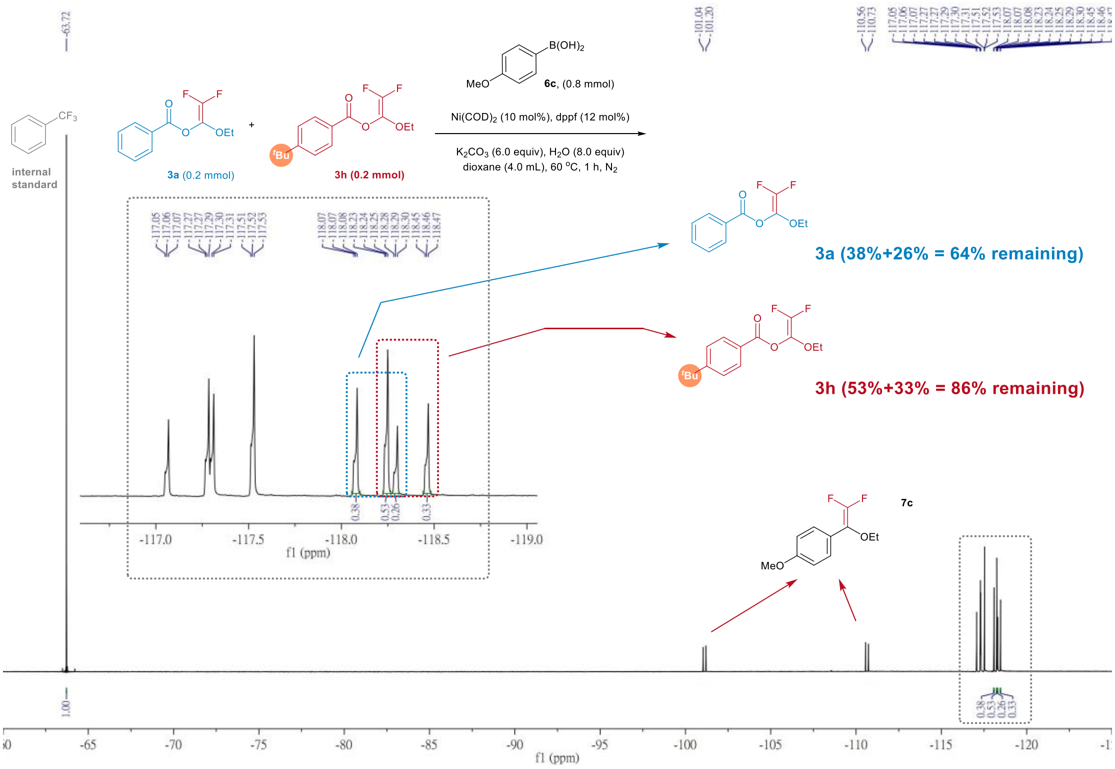

- ^{19}F NMR spectra of **(reaction mixture “3a + 3h” – trial 2)**

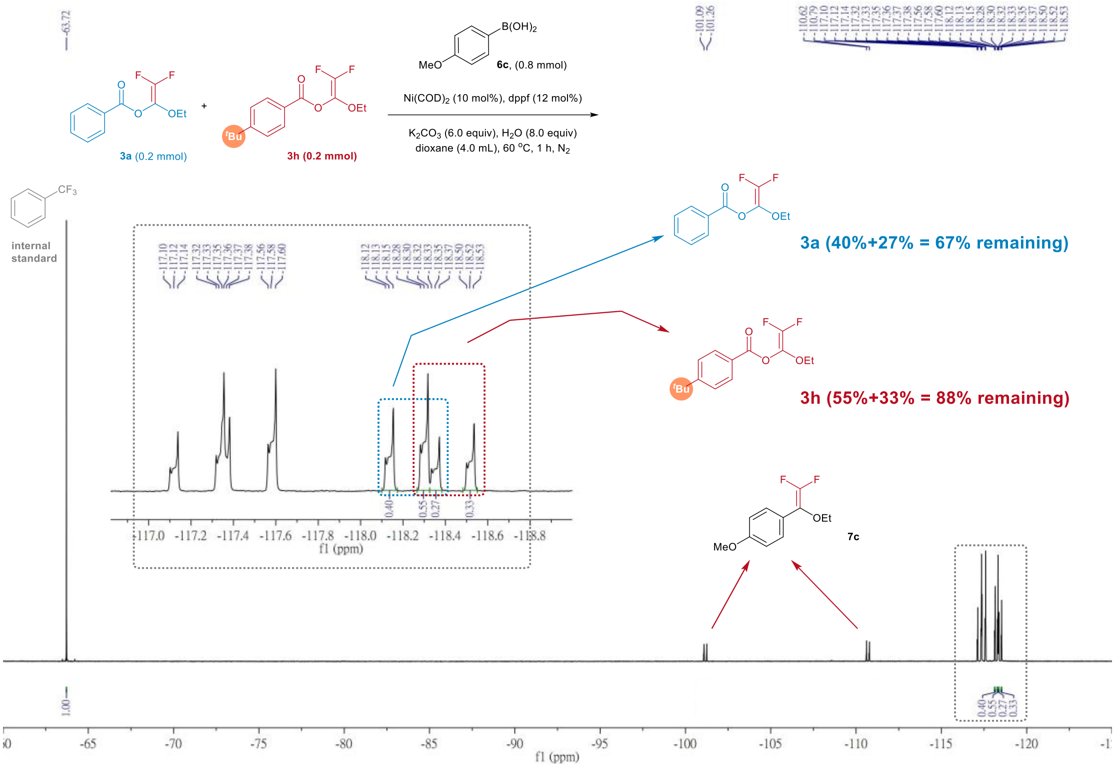

- ^{19}F NMR spectra of **(reaction mixture “3a + 3h” – trial 3)**

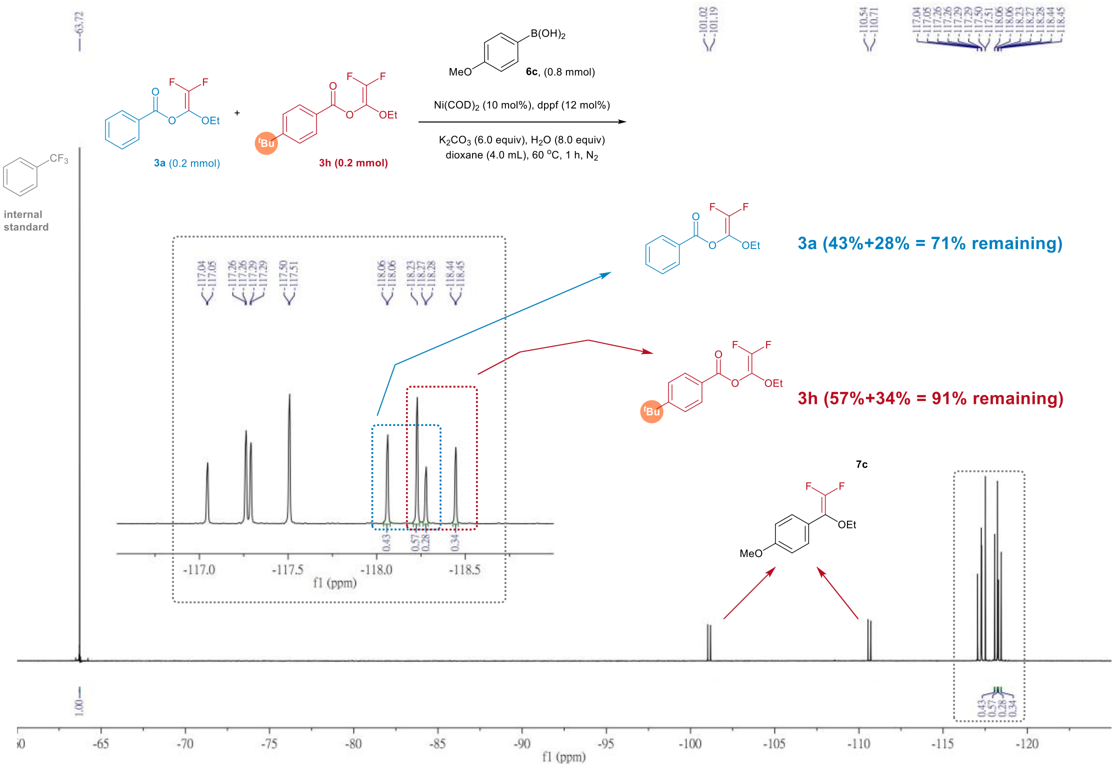

- ^{19}F NMR spectra of pure **3a** and pure **3i**

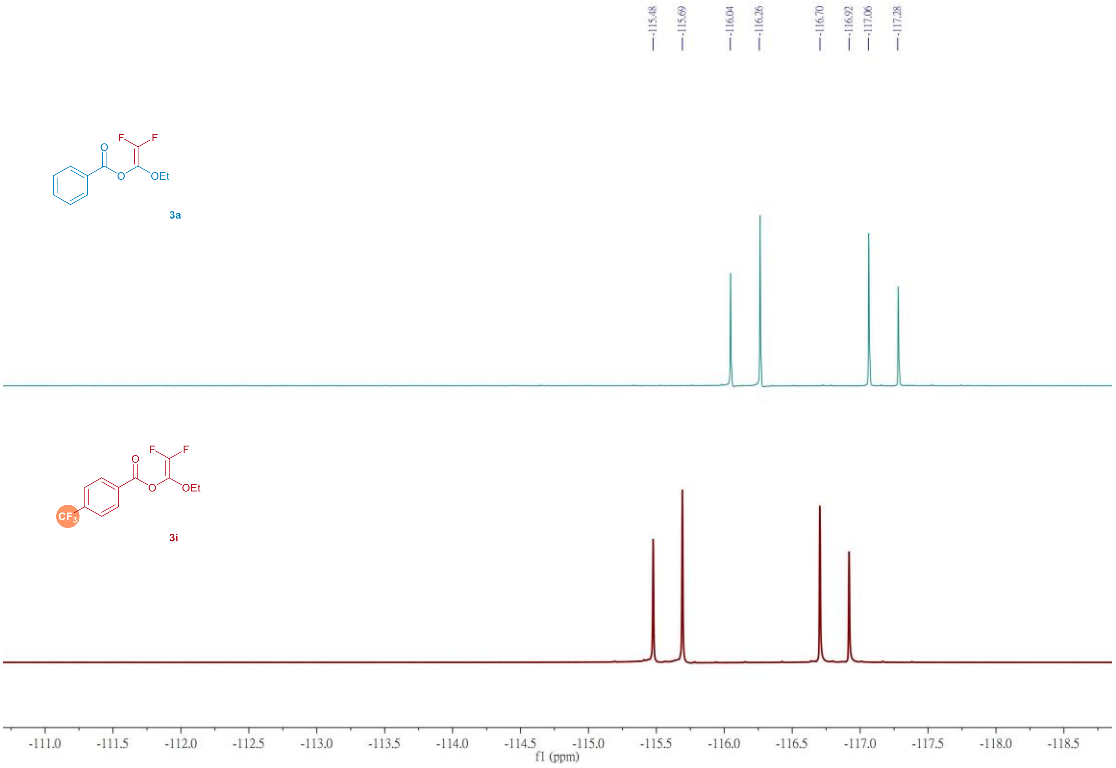

- ^{19}F NMR spectra of **(reaction mixture "3a + 3i" – trial 1)**

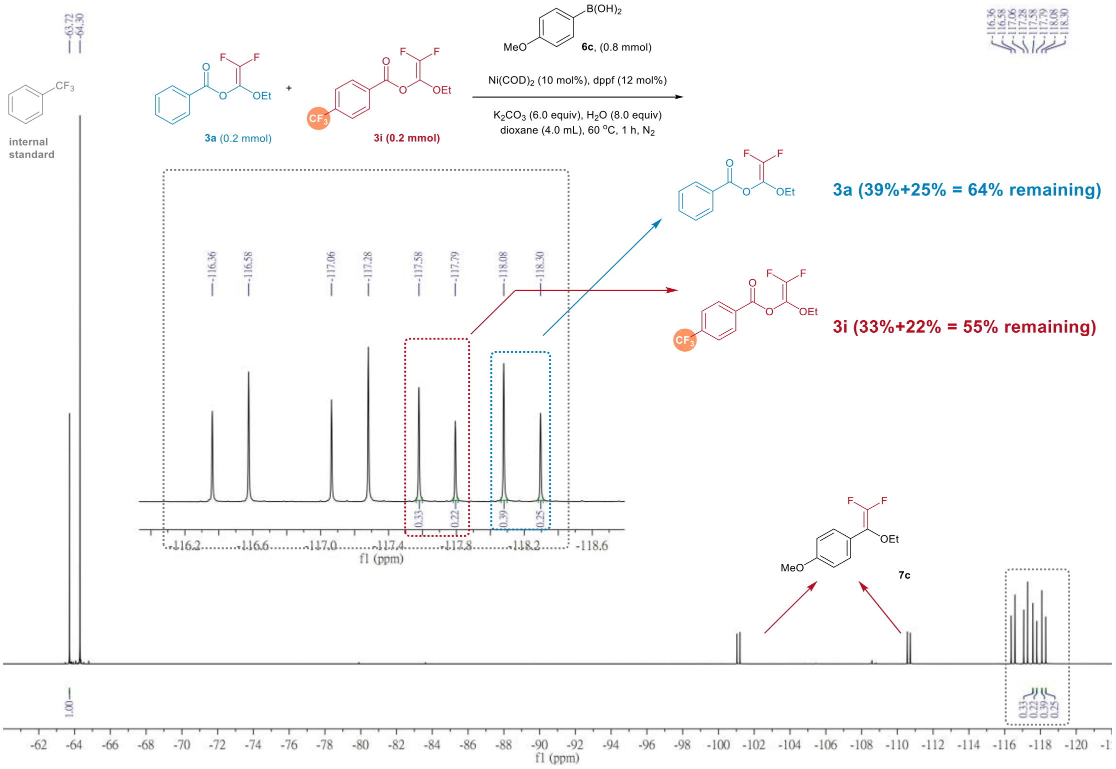

- ^{19}F NMR spectra of **(reaction mixture “3a + 3i” – trial 2)**

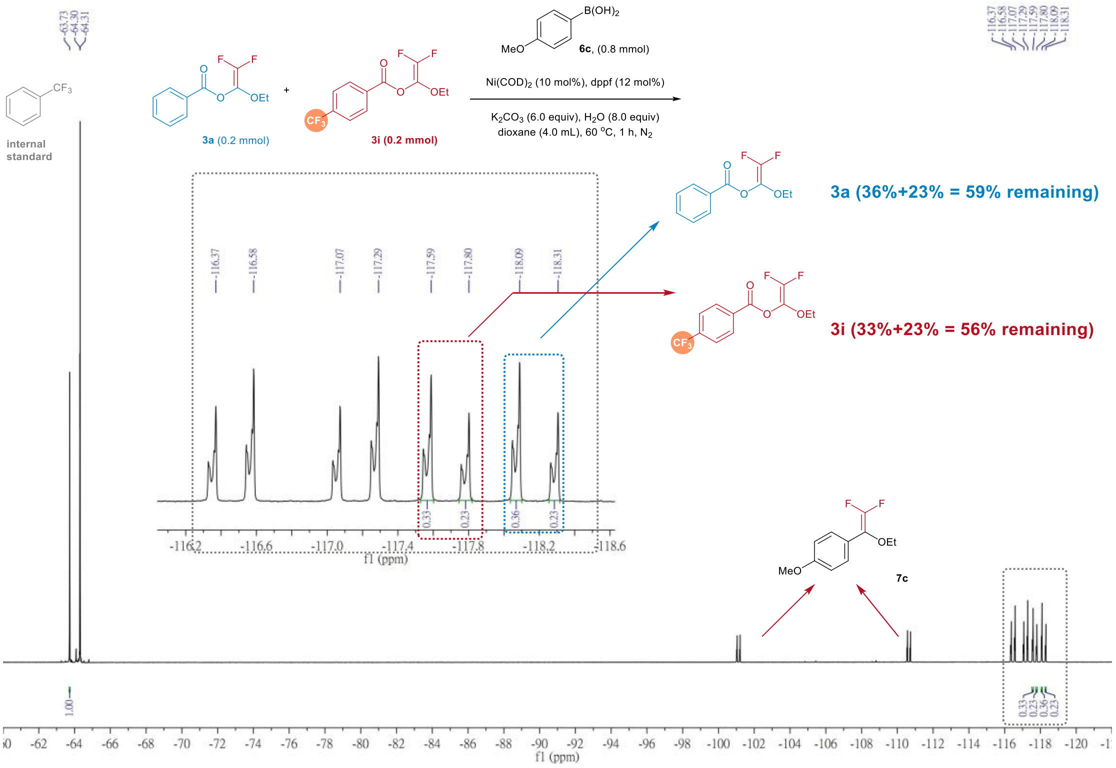

- ^{19}F NMR spectra of **(reaction mixture “3a + 3i” – trial 3)**

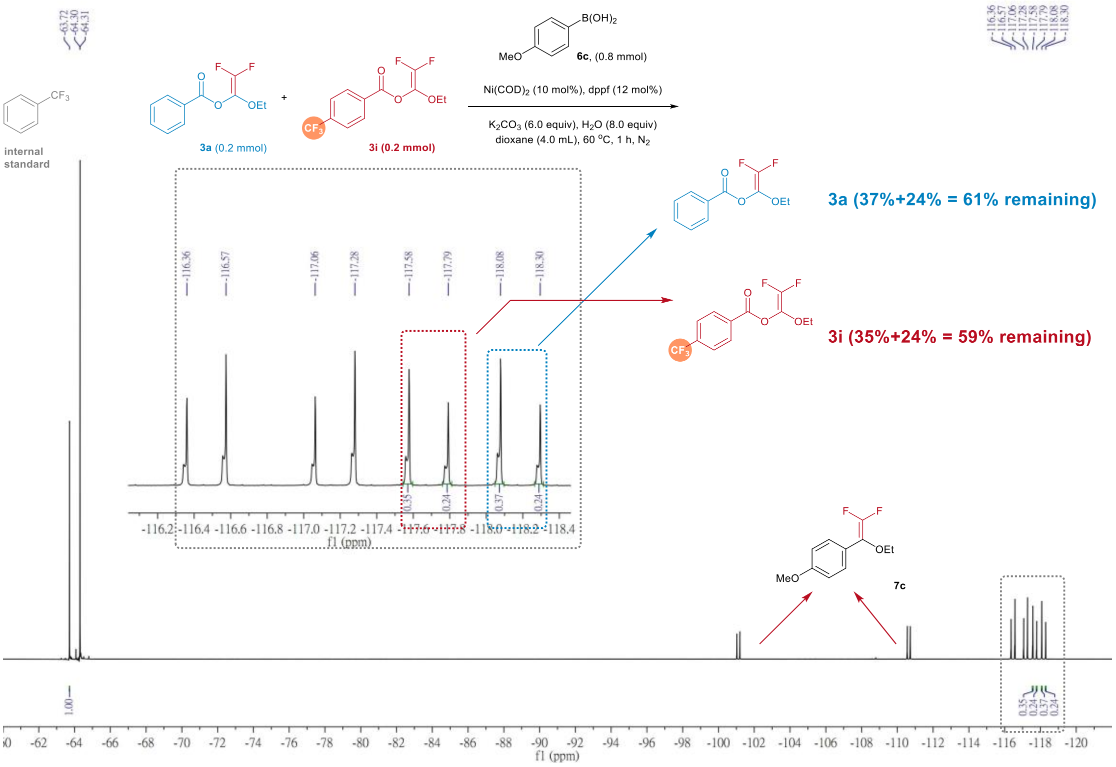

Result summary for [3a (H) + 3b (Me)]

**trial**	**3a** remaining	**3b** remaining	$\frac{k_{\mathrm{Me}}}{k_{\mathrm{H}}}$	$\log\left(\frac{k_{\mathrm{Me}}}{k_{\mathrm{H}}}\right)$	*Avg.* $\log\left(\frac{k_{\mathrm{Me}}}{k_{\mathrm{H}}}\right)$
1	51%	81%	0.31	-0.50	
2	46%	77%	0.34	-0.47	-0.45
3	45%	72%	0.41	-0.39	

Result summary for [3a (H) + 3c (OMe)]

**trial**	**3a** remaining	**3d** remaining	$\frac{k_{\mathrm{OMe}}}{k_{\mathrm{H}}}$	$\log\left(\frac{k_{\mathrm{OMe}}}{k_{\mathrm{H}}}\right)$	*Avg.* $\log\left(\frac{k_{\mathrm{OMe}}}{k_{\mathrm{H}}}\right)$
1	58%	91%	0.17	-0.76	
2	54%	82%	0.32	-0.49	-0.68
3	57%	89%	0.21	-0.68	

Result summary for [3a (H) + 3d (Cl)]

**trial**	**3a** remaining	**3d** remaining	$\frac{k_{\mathrm{Cl}}}{k_{\mathrm{H}}}$	$\log\left(\frac{k_{\mathrm{Cl}}}{k_{\mathrm{H}}}\right)$	*Avg.* $\log\left(\frac{k_{\mathrm{Cl}}}{k_{\mathrm{H}}}\right)$
1	40%	60%	0.59	-0.25	
2	50%	65%	0.62	-0.21	-0.18
3	48%	54%	0.84	-0.08	

Result summary for [3a (H) + 3f (F)]

**trial**	**3a** remaining	**3f** remaining	$\frac{k_{\mathrm{F}}}{k_{\mathrm{H}}}$	$\log\left(\frac{k_{\mathrm{F}}}{k_{\mathrm{H}}}\right)$	*Avg.* $\log\left(\frac{k_{\mathrm{F}}}{k_{\mathrm{H}}}\right)$
1	39%	48%	0.78	-0.11	
2	50%	59%	0.76	-0.12	-0.11
3	38%	46%	0.80	-0.10	

Result summary for [3a (H) + 3h (^{t}Bu)]

**trial**	**3a** remaining	**3h** remaining	$\frac{k_{tBu}}{k_H}$	$\log\left(\frac{k_{tBu}}{k_H}\right)$	*Avg.* $\log\left(\frac{k_{tBu}}{k_H}\right)$
1	64%	86%	0.34	-0.47	
2	67%	88%	0.32	-0.49	-0.51
3	71%	91%	0.28	-0.56	

Result summary for [3a (H) + 3i (CF_3)]

**trial**	**3a** remaining	**3i** remaining	$\frac{k_{CF_3}}{k_H}$	$\log\left(\frac{k_{CF_3}}{k_H}\right)$	*Avg.* $\log\left(\frac{k_{CF_3}}{k_H}\right)$
1	64%	55%	1.34	0.13	
2	59%	56%	1.10	0.41	0.27
3	61%	59%	1.07	0.28	

Hammett Correlation Plot

**FG**	p*K*a of FG-$C_6H_4CO_2H$	σ	*Avg.* $\log\left(\frac{k_{FG}}{k_H}\right)$
OMe	4.57	-0.27	-0.68
^{t}Bu	4.57	-0.20	-0.51
Me	4.38	-0.17	-0.45
H	4.20	0	0
F	4.14	0.06	-0.11
Cl	3.97	0.23	-0.17
CF_3	3.69	0.54	0.27

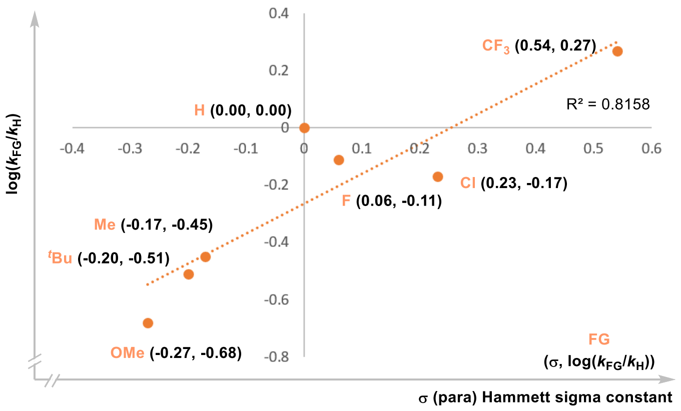

5.4 Side-products analysis preparation of BzO-DFs (3d and 3e)

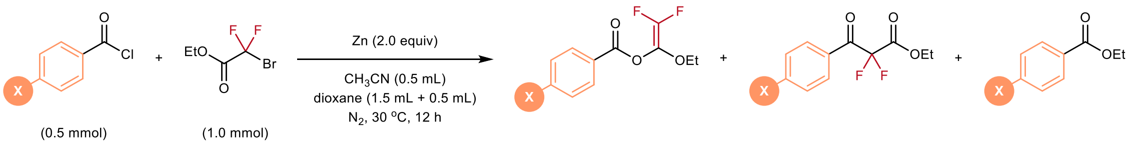

X = H	3a, 75%	3a', 3%	-
X = Cl	3d, 64%	3d', 3%	3d'', 15%
X = Br	3e, 45%	3e', 4%	3e'', 18%

For the synthesis of BzO-DFs bearing Cl- (**3d**), and Br- (**3e**) substituents, lower product yields (45–64%) were obtained. In these cases, side-products (**3d′**, **3d″**) and (**4d′**, **4d″**) were characterized.

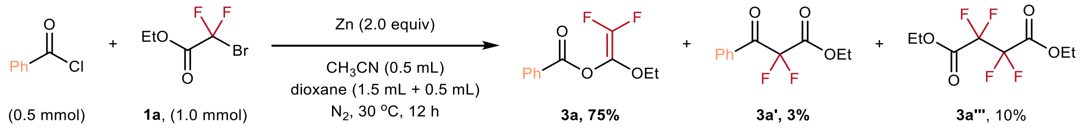

For reaction between benzoyl chloride and **1a** promoted by zinc powder, we found **3a** could be obtained in 75% yield. A reductive cross-electrophile coupling side-product ethyl 2,2-difluoro-3-oxo-3-phenylpropanoate **3a′** was also detected in the reaction system. 10% of Homocoupling product **3a‴** was also be detected.

To a 25 mL round bottom flask with a magnetic stir bar, benzoyl chloride (0.5 mmol), Zn powder (2.0 equiv, 1.0 mmol) were mixed in CH3CN (0.5 mL) and dioxane (1.5 mL) in a glovebox. The reaction mixture was then sealed with a rubber septum inside the glovebox. To the mixture, **1a** (0.4 mmol) was diluted with dioxane (0.5 mL) and added with a syringe pump over 20 min. Then the reaction mixture was stirred for further 12 hours at 30 °C. After the reaction was completed, the reaction mixture was filtered, and the filtrate was concentrated in *vacuo*. The residue was purified by flash column chromatography (n-hexane: ethyl acetate) to give **3a**, **3a′** and **3a‴**.

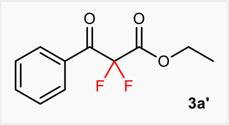

Ethyl 2,2-difluoro-3-oxo-3-phenylpropanoate[7] (3a′): colorless oil. **^{1}H NMR** (400 MHz, Chloroform-*d*) δ= 8.08 (d, *J* = 7.56 Hz, 2H), 7.68 (t, *J* = 7.40 Hz,

1H), 7.53 (t, J = 7.88 Hz, 2H), 4.39 (q, J = 7.08 Hz, 2H), 1.32 (t, J = 7.08 Hz, 3H) ppm. **^{13}C NMR** (101 MHz, Chloroform-*d*) δ= 185.5 (t, J = 27.6 Hz), 161.8 (t, J = 30.6 Hz), 135.1, 131.1, 129.9 (m), 129.0, 109.8 (t, J = 265.6 Hz), 63.8, 13.8 ppm. **^{19}F NMR** (376 MHz, Chloroform-*d*) δ= -107.61 (s, 2F) ppm.

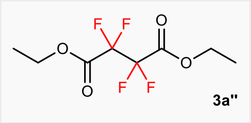

Diethyl 2,2,3,3-tetrafluorosuccinate (3a″): colorless oil. **^{1}H NMR** (400 MHz, Chloroform-*d*) δ= 4.41 (q, J = 6.85 Hz, 2H), 1.38 (t, J = 7.19 Hz, 3H) ppm. **^{13}C NMR** (101 MHz, Chloroform-*d*) δ= 159.4, 64.2, 31.6, 13.7 ppm. **^{19}F NMR** (376 MHz, Chloroform-*d*) δ= -120.12 (s, 4F) ppm. **HRMS** (EI): calcd. for $C_8H_{10}F_4O_4$: 246.0515. found: 246.0506.

5.5 Side-products analysis for the Ni-catalyzed cross-coupling reaction between BzO-DF (3a) and (4-chlorophenyl) / (4-(methylthio)phenyl) / (4-hydroxyphenyl) boronic acid

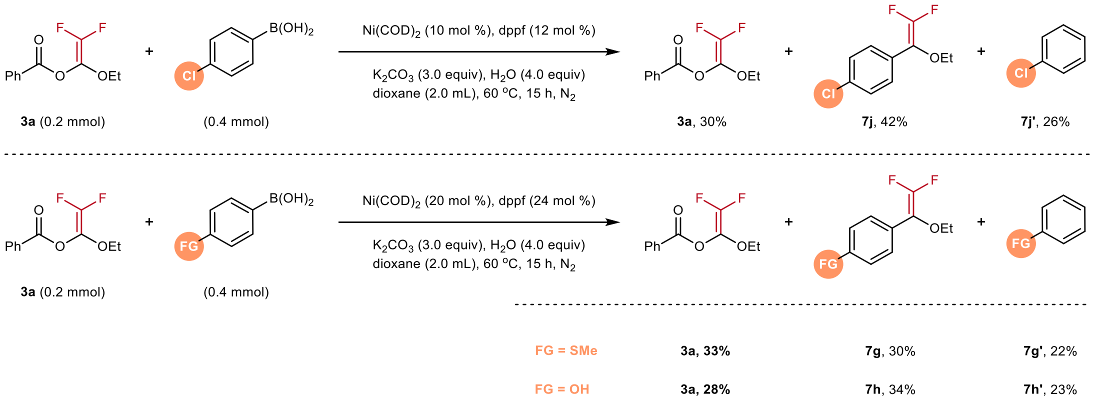

For the Ni-catalyzed cross-coupling reaction between **3a** and (4-chlorophenyl)- / (4-(methylthio)phenyl)- / (4-hydroxyphenyl)-boronic acid, we observed relatively lower yields when compared to other boronic acids. We therefore analyzed the reaction crudes of these reaction and found that around 28–33% **3a** remains after the optimized reaction time. The unreacted arylboronic acids were also found to be deboronated.

5.6 Acidic work-up for the Ni-catalyzed coupling reaction for recovery of the benzoic acids leaving group

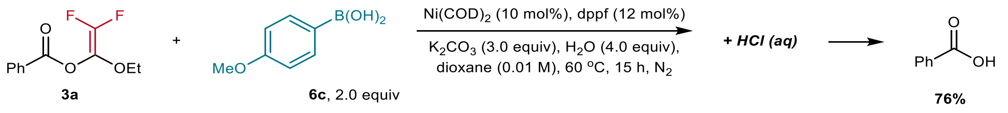

After the Ni-cat. cross coupling of **3a** with **6c**, DCM (20 mL) was added to the crude mixture and extracted with water (20 mL x 3). Next, 1M HCl (20 mL) was added to the aqueous layer and the resulting mixture was extracted with DCM (20 ml x 3). The combined organic layer was dried with anhydrous $MgSO_4$ and concentrated under vacuum. The benzoic acid was obtained (76% yield) through flash column chromatography.

5.7 Acidic work-up for the Ni-catalyzed coupling reaction for recovery of the benzoic acids leaving group

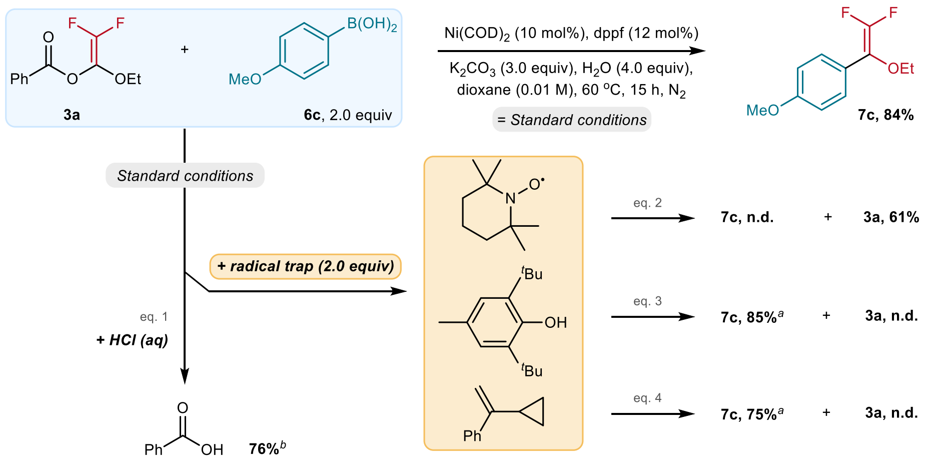

To a 8 mL vial equipped with a magnetic stir, 2,2-difluorovinyl benzoate **3a** (1.0 equiv, 0.2 mmol), boronic acid **6c** (2.0 equiv, 0.4 mmol), [radical scavenger (2.0 equiv)] and K_2CO_3 (3.0 equiv, 0.6 mmol) were mixed with deionized water (4.0 equiv, 0.8 mmol). Then a premixed solution of $Ni(COD)_2$ (10 mol%, 0.02 mmol), and dppf (12 mol%, 0.024 mmol) in dioxane (0.01 M, 2.0 mL) was transferred to the 8 mL vial. The vial was then capped inside the glovebox. The reaction mixture was stirred for 15 hours at 60 ^oC. Upon completion, the reaction mixture was cooled to room temperature and

diluted with ethyl acetate. The solution was then filtered through a pad of Celite® and the filtrate was dried over $MgSO_4$ and concentrated in *vacuo*.

Density Functional Theory (DFT) calculation results

All of the structures for reactants, intermediates, transition states, and products were calculated at the B3LYP[8] level of density functional theory (DFT) with the 6-31G* basis set for H, B, C, O, F, P and the LANL2DZ[9,10] basis set for K, Fe, Ni. Polarization functions were added for Fe (ζ_f = 2.462) and Ni (ζ_f = 3.130).[11] Frequency calculations for all of the optimized structures were performed to ensure that transition states only have one imaginary frequency and local minima have no imaginary frequency. Intrinsic Reaction Coordinates[12] calculations of each transition state were carried out to confirm the structure connecting two relevant minima. All calculations were performed using Gaussian 09 package.[13]

6. X-ray crystallographic data for 5c, 4s and 9a

Supplementary Figure 1. 2,2-difluoro-1-(4-methoxyphenyl)vinyl benzoate (5c) (CCDC 1921828)

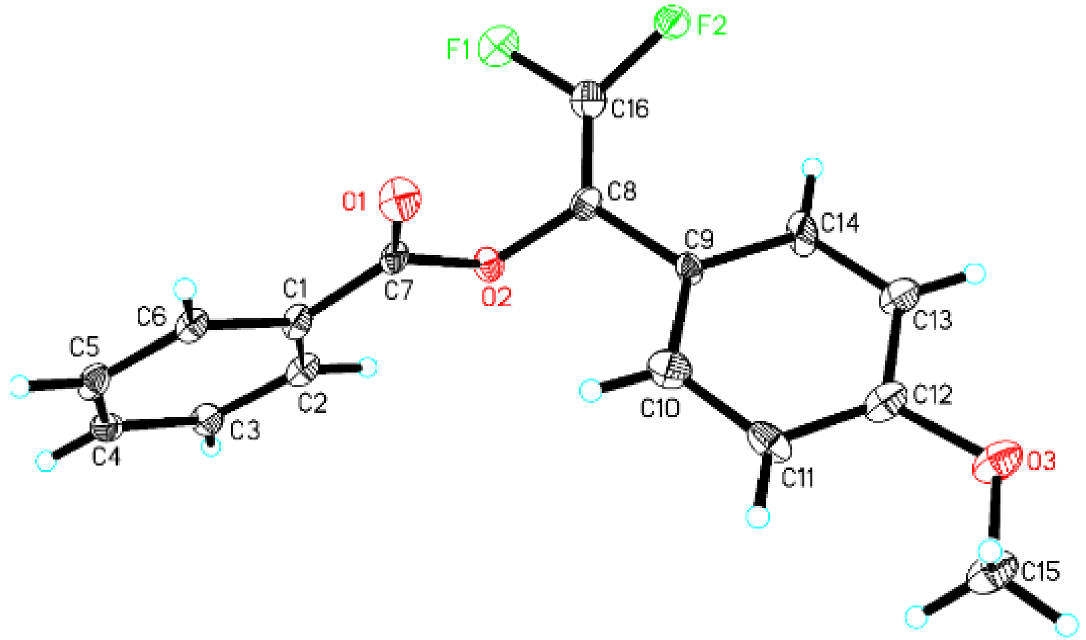

Crystal structure determination of 5c

Crystal Data for $C_{16}H_{12}F_2O_3$ (M =290.26 g/mol): orthorhombic, space group $Pca2_1$ (no. 29), a = 11.1992(5) Å, b = 31.9196(18) Å, c = 7.4845(5) Å, V = 2675.5(3) Å^3, Z = 4, T = 293.98(10) K, μ(CuKα) = 0.501 mm^{-1}, $Dcalc$ = 0.721 g/cm^3, 9868 reflections measured (5.538° ≤ 2Θ ≤ 147.176°), 3586 unique (R_{int} = 0.0751, R_{sigma} = 0.0580) which were used in all calculations. The final R_1 was 0.0425 (I > 2σ(I)) and wR_2 was 0.1382 (all data).

Supplementary Table 4 Crystal data and structure refinement for 5c

Empirical formula	$C_{16}H_{12}F_2O_3$
Formula weight	290.26
Temperature/K	293.98(10)
Crystal system	orthorhombic
Space group	$Pca2_1$
a/Å	11.1992(5)
b/Å	31.9196(18)
c/Å	7.4845(5)
α/°	90
β/°	90
γ/°	90
Volume/Å^3	2675.5(3)
Z	4
ρ_{calc}g/cm^3	0.721
μ/mm^{-1}	0.501
F(000)	600.0
Crystal size/mm^3	0.14 × 0.13 × 0.12
Radiation	CuKα (λ = 1.54184)
2Θ range for data collection/°	5.538 to 147.176
Index ranges	$-13 \leq h \leq 13$, $-39 \leq k \leq 36$, $-8 \leq l \leq 4$
Reflections collected	9868
Independent reflections	3586 [R_{int} = 0.0751, R_{sigma} = 0.0580]
Data/restraints/parameters	3586/22/185
Goodness-of-fit on F^2	1.058
Final R indexes [I>=2σ (I)]	R_1 = 0.0425, wR_2 = 0.1276
Final R indexes [all data]	R_1 = 0.0466, wR_2 = 0.1382
Largest diff. peak/hole / e Å^{-3}	0.24/-0.23
Flack parameter	0.20(16)

Supplementary Table 5 Fractional Atomic Coordinates ($\times10^4$) and Equivalent Isotropic Displacement Parameters ($Å^2\times10^3$) for 5c. U_{eq} is defined as 1/3 of of the trace of the orthogonalised U_{IJ}tensor

Atom	*x*	*y*	*z*	U(eq)
F2	6433.9(15)	8177.5(5)	9936(3)	29.9(4)
F1	6300.6(15)	8794.8(6)	11152(3)	34.1(5)
O2	5168.0(16)	9133.0(5)	8300(3)	23.0(5)
O1	3468.5(17)	8990.8(6)	9855(3)	31.5(5)
O3	4468(2)	7795.6(8)	1919(3)	39.1(5)
C7	4102(2)	9247.8(9)	9184(4)	23.0(6)
C3	4625(2)	10414.6(9)	8650(5)	25.4(6)
C6	2926(2)	9858.8(8)	9987(4)	22.2(5)
C1	3953(2)	9701.1(9)	9176(5)	23.0(6)
C4	3597(3)	10570.0(9)	9429(5)	25.1(6)
C5	2750(3)	10299.1(9)	10096(5)	27.0(6)
C16	6057(2)	8570.2(9)	9623(5)	28.5(7)
C2	4822(2)	9974.6(9)	8486(4)	23.4(6)
C8	5456(3)	8711.1(8)	8280(4)	23.0(6)
C9	5201(2)	8472.4(8)	6592(4)	20.5(6)
C11	3948(3)	8387.5(8)	3917(5)	31.8(8)
C12	4646(3)	8043.4(10)	3445(5)	31.2(6)
C10	4217(3)	8596.5(10)	5553(5)	33.8(8)
C14	5858(3)	8122.6(10)	6080(5)	33.7(8)
C13	5627(3)	7910.5(11)	4521(5)	33.1(7)
C15	3457(3)	7895.6(11)	860(5)	39.1(5)

Supplementary Table 6 Anisotropic Displacement Parameters (Å^2×10^3) for 5c. The Anisotropic displacement factor exponent takes the form: -2π^2[h^{2}a*^{2}U$_{11}$+2hka*b*U$_{12}$+…]

Atom	U_{11}	U_{22}	U_{33}	U_{23}	U_{13}	U_{12}
F2	27.8(9)	35.1(9)	26.8(10)	0.4(8)	-2.8(8)	7.9(7)
F1	22.7(8)	44.6(10)	35.1(12)	-2.8(9)	-0.1(8)	-7.2(7)
O2	17.2(9)	23.5(9)	28.2(12)	-1.2(9)	4.5(9)	1.8(7)
O1	17.3(9)	37.2(11)	40.0(15)	1.9(10)	10.1(10)	-7.0(8)
O3	26.3(8)	64.3(12)	26.9(10)	-2.4(9)	0.8(8)	0.6(8)
C7	17.7(12)	32.8(14)	18.7(16)	-1.5(12)	6.8(12)	6.3(11)
C3	15.8(13)	34.2(15)	26.3(18)	-0.3(13)	-0.1(13)	0.6(11)
C6	11.0(10)	35.0(13)	20.7(14)	-2.1(12)	1.9(11)	-2.4(9)
C1	9.2(11)	36.1(14)	23.8(17)	-7.3(13)	-0.4(11)	2.0(10)
C4	28.0(14)	25.0(13)	22.2(16)	1.7(12)	-3.0(14)	0.1(11)
C5	19.6(12)	39.3(13)	22.0(14)	-4.9(12)	2.4(12)	0.1(10)
C16	15.1(12)	36.7(16)	34(2)	0.4(14)	6.1(13)	3.7(11)
C2	9.5(13)	40.6(15)	20.0(15)	-3.1(14)	0.7(11)	-5.8(10)
C8	22.6(14)	26.0(13)	20.5(16)	-4.3(12)	-0.9(13)	-3.7(10)
C9	16.6(11)	19.7(12)	25.3(16)	-3.8(12)	-4.7(12)	4.8(9)
C11	18.3(13)	23.5(13)	54(2)	5.7(15)	-10.5(15)	-1.7(11)
C12	16.6(11)	52.1(15)	24.7(14)	-2.6(13)	2.6(12)	-4.8(11)
C10	22.9(15)	39.1(17)	40(2)	3.7(16)	-3.3(15)	0.8(12)
C14	17.6(13)	39.5(16)	44(2)	-10.0(16)	3.8(15)	16.7(12)
C13	23.7(14)	51.2(19)	24.4(18)	-1.8(15)	-0.2(13)	9.0(13)
C15	26.3(8)	64.3(12)	26.9(10)	-2.4(9)	0.8(8)	0.6(8)

Supplementary Table 7 Bond Lengths for 5c

Atom	Atom	Length/Å	Atom	Atom	Length/Å
F2	C16	1.343(3)	C6	C5	1.421(4)
F1	C16	1.377(4)	C1	C2	1.406(4)
O2	C7	1.414(3)	C4	C5	1.378(4)
O2	C8	1.385(3)	C16	C8	1.291(5)
O1	C7	1.195(4)	C8	C9	1.503(4)
O3	C12	1.404(4)	C9	C10	1.405(4)
O3	C15	1.419(4)	C9	C14	1.391(4)
C7	C1	1.456(4)	C11	C12	1.393(4)
C3	C4	1.382(4)	C11	C10	1.427(5)
C3	C2	1.427(4)	C12	C13	1.427(4)
C6	C1	1.395(4)	C14	C13	1.374(5)

Supplementary Table 8 Bond Angles for 5c

Atom	Atom	Atom	Angle/°	Atom	Atom	Atom	Angle/°
C8	O2	C7	117.0(2)	C1	C2	C3	118.2(3)
C12	O3	C15	116.2(3)	O2	C8	C9	117.3(3)
O2	C7	C1	110.6(2)	C16	C8	O2	116.8(3)
O1	C7	O2	121.3(2)	C16	C8	C9	125.3(3)
O1	C7	C1	128.0(3)	C10	C9	C8	118.1(3)
C4	C3	C2	121.2(3)	C14	C9	C8	122.5(3)
C1	C6	C5	119.8(3)	C14	C9	C10	119.3(3)
C6	C1	C7	116.8(3)	C12	C11	C10	117.9(3)
C6	C1	C2	120.4(3)	O3	C12	C13	113.6(3)
C2	C1	C7	122.6(2)	C11	C12	O3	124.8(3)
C3	C4	C5	120.1(3)	C11	C12	C13	121.6(3)
C4	C5	C6	120.3(3)	C9	C10	C11	120.5(3)
F2	C16	F1	106.2(3)	C13	C14	C9	122.0(3)
C8	C16	F2	128.7(3)	C14	C13	C12	118.6(3)
C8	C16	F1	124.7(3)				

Supplementary Table 9 Hydrogen Atom Coordinates (Å×10^4) and Isotropic Displacement Parameters (Å^2×10^3) for 5c

**Atom**	***x***	***y***	***z***	**U(eq)**
H3	5198.52	10600.21	8224.7	31
H6	2358.6	9676.62	10454.91	27
H4	3478.2	10857.82	9503.26	30
H5	2059.27	10404.68	10618.67	32
H2	5507.02	9871.63	7936.71	28
H11	3326.7	8477.98	3187.81	38
H10	3736.52	8816.69	5936.2	41
H14	6472.68	8029.53	6816.07	40
H13	6099.64	7685.22	4174.47	40
H15A	3569.68	7789.94	-329	59
H15B	3358.65	8194.25	817.39	59
H15C	2757.33	7770.44	1374.74	59

Supplementary Figure 2. 1-(((5S,8R,9S,10S,13S,14S)-10,13-dimethyl-17-oxohexadecahydro-1H-cyclopenta[a]phenanthren-3-yl)oxy)-2,2-difluorovinyl benzoate (4s) (CCDC 2008864)

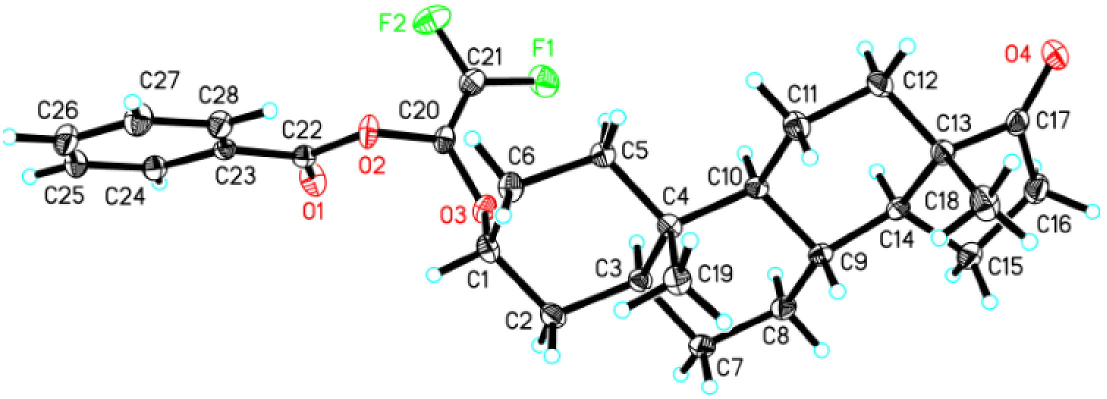

Crystal structure determination of 4s

Crystal Data for $C_{28}H_{34}F_2O_4$ (M =472.55 g/mol): orthorhombic, space group $P2_12_12_1$ (no. 19), a = 9.04950(10) Å, b = 10.22550(10) Å, c = 26.3128(3) Å, V = 2434.87(5) Å^3, Z = 4, T = 149.99(18) K, μ(Cu Kα) = 0.781 mm^{-1}, $Dcalc$ = 1.289 g/cm^3, 15581 reflections measured (6.718° ≤ 2Θ ≤ 147.234°), 4813 unique (R_{int} = 0.0268, R_{sigma} = 0.0203) which were used in all calculations. The final R_1 was 0.0334 (I > 2σ(I)) and wR_2 was 0.0858 (all data).

Supplementary Table 10 Crystal data and structure refinement for 4s

Identification code	3
Empirical formula	$C_{28}H_{34}F_2O_4$
Formula weight	472.55
Temperature/K	149.99(18)
Crystal system	orthorhombic
Space group	$P2_12_12_1$
a/Å	9.04950(10)
b/Å	10.22550(10)
c/Å	26.3128(3)
α/°	90
β/°	90
γ/°	90
Volume/Å^3	2434.87(5)
Z	4
ρ_{calc}g/cm^3	1.289
μ/mm^{-1}	0.781
F(000)	1008.0
Crystal size/mm^3	0.14 × 0.13 × 0.12
Radiation	Cu Kα (λ = 1.54184)
2Θ range for data collection/°	6.718 to 147.234
Index ranges	$-11 \leq h \leq 11$, $-12 \leq k \leq 10$, $-31 \leq l \leq 32$
Reflections collected	15581
Independent reflections	4813 [R_{int} = 0.0268, R_{sigma} = 0.0203]
Data/restraints/parameters	4813/0/309
Goodness-of-fit on F^2	1.064
Final R indexes [I>=2σ (I)]	R_1 = 0.0334, wR_2 = 0.0852
Final R indexes [all data]	R_1 = 0.0343, wR_2 = 0.0858
Largest diff. peak/hole / e Å^{-3}	0.15/-0.20
Flack/Hooft parameter	0.04(5)/0.05(4)

Supplementary Table 11 Fractional Atomic Coordinates (×10^4) and Equivalent Isotropic Displacement Parameters (Å2×10^3) for 4s. Ueq is defined as 1/3 of of the trace of the orthogonalised U_{IJ}tensor

Atom	*x*	*y*	*z*	U(eq)
F1	6198.7(14)	9557.5(15)	6761.9(5)	46.3(3)
O3	4980.0(13)	8433.6(12)	5901.8(5)	28.5(3)
F2	4210.1(16)	10730.7(13)	6875.7(5)	50.7(4)
O2	2745.1(13)	9439.4(13)	6111.5(5)	32.2(3)
O1	3311.8(15)	11192.9(15)	5633.0(6)	38.7(4)
O4	10532.9(18)	2698.1(16)	8064.7(5)	42.8(4)
C23	786.3(18)	10510.6(17)	5698.3(6)	22.3(3)
C5	4745.8(19)	5896.8(18)	6542.7(7)	25.4(4)
C14	9696.5(19)	3882.5(17)	6853.5(7)	23.9(4)
C20	4223.4(18)	9221.3(18)	6212.7(7)	26.4(4)
C9	8569.1(19)	4106.2(17)	6433.2(6)	23.9(4)
C22	2398.6(19)	10465.3(17)	5794.6(6)	23.5(3)
C10	7175.7(18)	4718.4(17)	6678.7(6)	22.2(3)
C24	232(2)	11527.5(18)	5406.1(7)	27.6(4)
C4	5958.3(19)	5068.7(17)	6285.1(6)	23.5(3)
C6	3603(2)	6449.3(19)	6171.2(7)	28.8(4)
C12	7784(2)	3599(2)	7524.6(7)	33.6(4)
C13	9134(2)	2985.5(18)	7279.1(7)	26.8(4)
C3	6676.5(19)	5964.4(18)	5875.9(7)	24.8(4)
C28	-158(2)	9546.7(19)	5884.0(7)	28.5(4)
C17	10504(2)	2872.8(18)	7608.9(8)	30.5(4)
C11	6600(2)	3870(2)	7120.6(7)	31.9(4)

C1	4293(2)	7205.4(18)	5735.7(7)	28.1(4)
C7	8007(2)	5328(2)	5618.7(7)	31.2(4)
C8	9183(2)	4977(2)	6011.1(7)	29.2(4)
C2	5547(2)	6463(2)	5487.4(7)	30.2(4)
C21	4841(2)	9800(2)	6601.8(8)	33.1(4)
C19	5274(2)	3823.1(19)	6055.2(8)	30.9(4)
C25	-1266(2)	11581(2)	5296.9(7)	33.1(4)
C26	-2201(2)	10627(2)	5479.1(8)	36.9(5)
C15	11247(2)	3338(2)	6735.6(7)	33.1(4)
C27	-1651(2)	9610(2)	5771.3(8)	36.2(5)
C16	11848(2)	3022(2)	7269.8(8)	36.3(5)
C18	8816(3)	1576(2)	7098.4(9)	39.6(5)

Supplementary Table 12 Anisotropic Displacement Parameters (Å^2×10^3) for 4s. The Anisotropic displacement factor exponent takes the form: -2π^2[h^{2}a*^{2}U$_{11}$+2hka*b*U$_{12}$+…]

Atom	U_{11}	U_{22}	U_{33}	U_{23}	U_{13}	U_{12}
F1	32.0(6)	60.5(8)	46.3(7)	-2.5(6)	-9.8(5)	-8.6(6)
O3	22.6(6)	24.3(6)	38.5(7)	-0.3(6)	1.2(5)	-0.9(5)
F2	56.9(8)	38.7(7)	56.5(8)	-13.0(6)	17.5(7)	-3.7(6)
O2	19.2(6)	27.7(7)	49.7(8)	16.0(6)	0.7(5)	2.7(5)
O1	29.8(7)	38.4(8)	47.9(8)	17.6(7)	-6.0(6)	-11.3(6)
O4	51.3(9)	47.2(9)	30.0(7)	1.2(7)	-9.7(7)	13.2(7)
C23	24.4(8)	21.6(8)	20.8(7)	-2.4(7)	1.1(6)	1.4(7)
C5	24.2(8)	25.7(9)	26.4(8)	1.8(7)	0.3(7)	-1.7(7)
C14	25.4(8)	20.2(8)	26.2(9)	-3.9(7)	1.2(7)	0.3(6)
C20	19.3(7)	24.0(9)	35.9(9)	5.5(7)	1.6(7)	-0.3(7)
C9	26.1(8)	21.6(8)	23.9(8)	-3.2(7)	1.0(7)	-0.3(6)
C22	27.0(8)	19.5(8)	23.9(8)	-0.1(7)	1.0(6)	-0.7(7)
C10	24.2(8)	21.9(8)	20.7(7)	-0.7(6)	0.3(6)	-1.6(7)
C24	31.3(9)	23.6(9)	27.9(9)	1.5(7)	1.3(7)	1.0(7)
C4	25.0(8)	22.6(8)	23.0(8)	1.1(7)	-1.5(6)	-2.6(7)
C6	23.7(8)	27.3(9)	35.5(10)	1.2(8)	-3.6(7)	-3.3(7)
C12	32.6(9)	43.9(11)	24.1(9)	8.3(8)	3.1(8)	5.8(8)
C13	31.6(9)	22.2(8)	26.7(9)	-0.9(7)	-3.1(7)	1.7(7)
C3	27.8(8)	24.0(9)	22.5(8)	1.1(7)	0.8(7)	0.0(7)
C28	28.2(8)	26.3(9)	30.9(9)	5.3(8)	2.6(7)	0.5(7)
C17	37.1(10)	22.0(9)	32.4(10)	-5.2(7)	-5.7(8)	6.9(7)
C11	25.8(9)	40.3(11)	29.8(9)	9.9(9)	2.4(7)	-1.2(8)

Supplementary Table 12 Anisotropic Displacement Parameters ($Å^2×10^3$) for 4s. The Anisotropic displacement factor exponent takes the form: $-2\pi^2[h^2a^{*2}U_{11}+2hka^*b^*U_{12}+…]$

Atom	U_{11}	U_{22}	U_{33}	U_{23}	U_{13}	U_{12}
C1	27.1(9)	27.4(10)	30.0(9)	3.1(7)	-8.4(7)	-1.2(7)
C7	36.1(10)	34.4(10)	23.1(8)	2.5(8)	5.2(8)	4.0(8)
C8	27.0(8)	32.5(10)	28.1(9)	1.7(8)	7.2(7)	3.0(7)
C2	37.6(10)	30.2(10)	22.8(8)	2.1(7)	-3.8(7)	0.6(8)
C21	29.7(9)	30.9(10)	38.6(10)	-1.0(8)	3.5(8)	-1.4(8)
C19	33.3(9)	24.9(9)	34.6(10)	-1.5(8)	-6.9(8)	-3.5(8)
C25	34.6(10)	32.7(10)	31.9(9)	1.8(8)	-3.7(8)	10.2(8)
C26	23.5(8)	44.3(12)	42.8(11)	-2.5(9)	-1.3(8)	5.1(8)
C15	27.9(9)	36.6(11)	35.0(10)	-2.3(8)	1.5(7)	6.1(8)
C27	26.8(9)	38.2(11)	43.5(11)	5.2(9)	5.0(8)	-3.7(8)
C16	31.4(9)	35.9(11)	41.6(11)	-6.3(9)	-6.9(8)	9.4(8)
C18	50.2(12)	24.4(10)	44.2(11)	3.6(9)	-12.0(9)	-5.4(9)

Supplementary Table 13 Bond Lengths for 4s

Atom	Atom	Length/Å	Atom	Atom	Length/Å
F1	C21	1.322(2)	C10	C4	1.554(2)
O3	C20	1.337(2)	C10	C11	1.541(2)
O3	C1	1.468(2)	C24	C25	1.387(3)
F2	C21	1.324(2)	C4	C3	1.556(2)
O2	C20	1.382(2)	C4	C19	1.540(2)
O2	C22	1.376(2)	C6	C1	1.517(3)
O1	C22	1.191(2)	C12	C13	1.518(3)
O4	C17	1.213(2)	C12	C11	1.534(3)
C23	C22	1.482(2)	C13	C17	1.517(3)
C23	C24	1.387(2)	C13	C18	1.545(3)
C23	C28	1.393(3)	C3	C7	1.527(2)
C5	C4	1.543(2)	C3	C2	1.533(2)
C5	C6	1.531(2)	C28	C27	1.385(3)
C14	C9	1.522(2)	C17	C16	1.516(3)
C14	C13	1.534(2)	C1	C2	1.514(3)
C14	C15	1.541(2)	C7	C8	1.526(3)
C20	C21	1.308(3)	C25	C26	1.377(3)
C9	C10	1.549(2)	C26	C27	1.386(3)
C9	C8	1.528(2)	C15	C16	1.541(3)

Supplementary Table 14 Bond Angles for 4s

Atom	Atom	Atom	Angle/°	Atom	Atom	Atom	Angle/°
C20	O3	C1	118.73(14)	C13	C12	C11	110.00(15)
C22	O2	C20	117.40(14)	C14	C13	C18	113.26(16)
C24	C23	C22	118.33(16)	C12	C13	C14	109.29(15)
C24	C23	C28	120.20(16)	C12	C13	C18	111.47(17)
C28	C23	C22	121.45(16)	C17	C13	C14	101.08(15)
C6	C5	C4	113.74(14)	C17	C13	C12	116.47(15)
C9	C14	C13	113.45(14)	C17	C13	C18	104.94(16)
C9	C14	C15	121.23(15)	C7	C3	C4	112.65(15)
C13	C14	C15	103.46(15)	C7	C3	C2	111.83(15)
O3	C20	O2	118.37(16)	C2	C3	C4	112.27(14)
C21	C20	O3	122.19(16)	C27	C28	C23	119.35(18)
C21	C20	O2	119.44(18)	O4	C17	C13	126.48(18)
C14	C9	C10	107.67(13)	O4	C17	C16	125.39(18)
C14	C9	C8	111.84(14)	C16	C17	C13	108.13(15)
C8	C9	C10	111.31(15)	C12	C11	C10	112.85(15)
O2	C22	C23	110.61(14)	O3	C1	C6	112.67(15)
O1	C22	O2	122.32(16)	O3	C1	C2	103.86(15)
O1	C22	C23	127.08(17)	C2	C1	C6	112.29(15)
C9	C10	C4	113.11(13)	C8	C7	C3	110.51(15)
C11	C10	C9	111.25(15)	C7	C8	C9	112.03(15)
C11	C10	C4	113.12(14)	C1	C2	C3	112.27(15)
C23	C24	C25	119.88(18)	F2	C21	F1	111.23(17)
C5	C4	C10	109.76(13)	C20	C21	F1	124.18(19)

C5	C4	C3	106.14(14)	C20	C21	F2	124.56(18)
C10	C4	C3	107.50(13)	C26	C25	C24	120.01(19)
C19	C4	C5	109.92(14)	C25	C26	C27	120.28(18)
C19	C4	C10	110.86(14)	C16	C15	C14	102.32(15)
C19	C4	C3	112.52(14)	C28	C27	C26	120.3(2)
C1	C6	C5	113.10(15)	C17	C16	C15	105.95(16)

Supplementary Table 15 Hydrogen Atom Coordinates (Å×10^4) and Isotropic Displacement Parameters (Å^2×10^3) for 4s

Atom	*x*	*y*	*z*	U(eq)
H5A	4240.71	5360.82	6792.32	30
H5B	5209.66	6617.07	6721.85	30
H14	9856.84	4738.64	7011.54	29
H9	8300.06	3258.04	6286.54	29
H10	7494.13	5547.59	6829.95	27
H24	863.48	12171.95	5283.6	33
H6A	3023.69	5733.88	6033.6	35
H6B	2935.84	7021.98	6354.89	35
H12A	8060.35	4410.77	7689.94	40
H12B	7389.31	3013.1	7780.79	40
H3	7051.06	6735.78	6055.52	30
H28	211.1	8867.23	6081.81	34
H11A	5769.97	4310.13	7279.36	38
H11B	6246.42	3044.46	6985.95	38
H1	3533.36	7396.53	5480.72	34
H7A	7692.77	4543.02	5441.85	37
H7B	8420.52	5925.07	5370.24	37
H8A	9572.73	5773.95	6159.52	35
H8B	9990.77	4527.67	5843.13	35
H2A	6042.01	7031.74	5246.23	36
H2B	5147.06	5725.97	5300.71	36
H19A	4740.33	3358.51	6313.68	46
H19B	6044.91	3274.82	5923.05	46

Supplementary Table 15 Hydrogen Atom Coordinates (Å×10⁴) and Isotropic Displacement Parameters (Å²×10³) for 4s

Atom	*x*	*y*	*z*	U(eq)
H19C	4611.18	4059.32	5785.65	46
H25	-1639.44	12261.55	5100.65	40
H26	-3205.2	10665.96	5405.81	44
H15A	11191.27	2558.19	6526.56	40
H15B	11856.03	3984.8	6565.68	40
H27	-2287.61	8967.1	5892.17	43
H16A	12418.19	2218.59	7264.04	44
H16B	12475.94	3724.43	7390.97	44
H18A	8112.05	1596.93	6825.42	59
H18B	8420.59	1075.2	7375.5	59
H18C	9715.73	1179.87	6981.93	59

Supplementary Figure 3. 4,4'-(2,2-difluoroethene-1,1-diyl)bis(methoxybenzene) (9a) (CCDC 1921829)

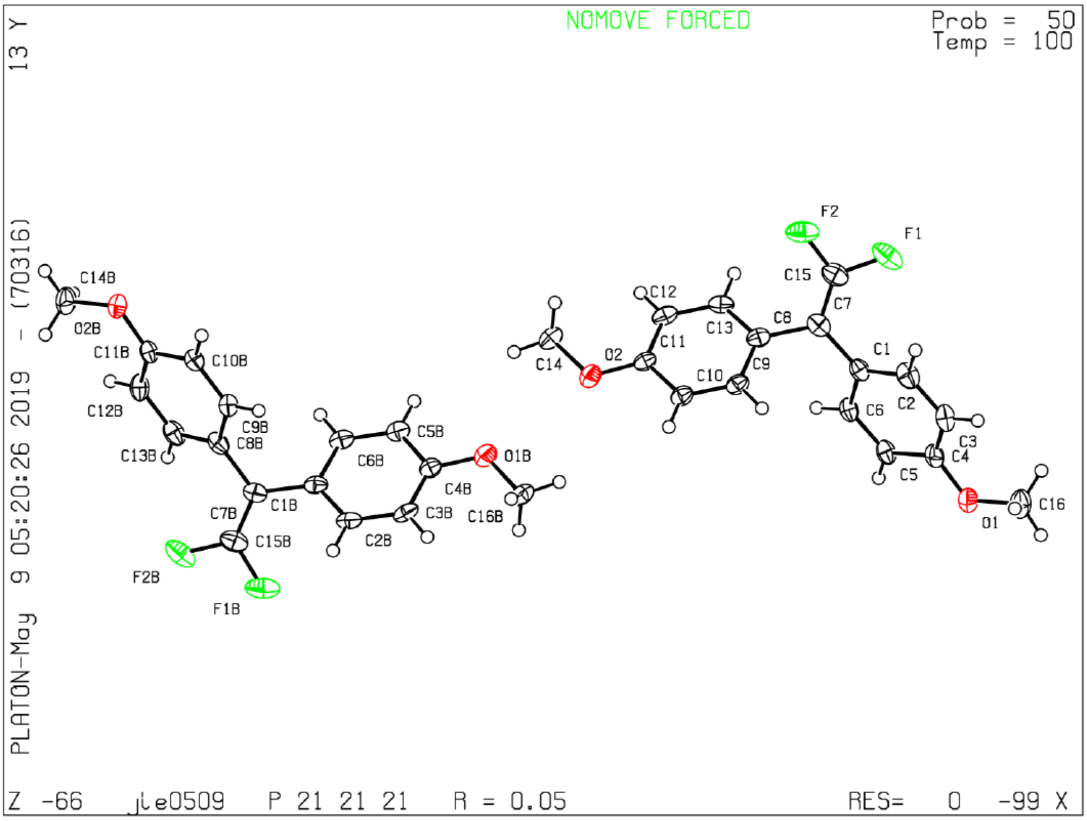

Crystal structure determination of 9a

Crystal Data for $C_{32}H_{28}F_4O_4$ (M =552.54 g/mol): orthorhombic, space group $P2_12_12_1$ (no.19), a = 7.3961(4) Å, b = 8.1539(4) Å, c = 44.723(2) Å, V = 2697.1(2) Å^3, Z = 4, T = 100.00(10) K, μ(MoKα) = 0.107 mm^{-1}, $Dcalc$ = 1.361 g/cm^3, 9889 reflections measured (5.078° ≤ 2Θ ≤ 49.984°), 4638 unique (R_{int} = 0.0358, R_{sigma} = 0.0547) which were used in all calculations. The final R_1 was 0.0472 (I > 2σ(I)) and wR_2 was 0.1098 (all data).

Supplementary Table 16 Crystal data and structure refinement for 9a

Empirical formula	$C_{32}H_{28}F_4O_4$
Formula weight	552.54
Temperature/K	100.00(10)
Crystal system	orthorhombic
Space group	$P2_12_12_1$
a/Å	7.3961(4)
b/Å	8.1539(4)
c/Å	44.723(2)
α/°	90
β/°	90
γ/°	90
Volume/Å^3	2697.1(2)
Z	4
ρ_{calc}g/cm^3	1.361
μ/mm^{-1}	0.107
F(000)	1152.0
Crystal size/mm^3	0.12 × 0.11 × 0.09
Radiation	MoKα (λ = 0.71073)
2Θ range for data collection/°	5.078 to 49.984
Index ranges	$-7 \le h \le 8, -8 \le k \le 9, -38 \le l \le 53$
Reflections collected	9889
Independent reflections	4638 [R_{int} = 0.0358, R_{sigma} = 0.0547]
Data/restraints/parameters	4638/0/365
Goodness-of-fit on F^2	1.065
Final R indexes [I>=2σ (I)]	R_1 = 0.0472, wR_2 = 0.1009
Final R indexes [all data]	R_1 = 0.0628, wR_2 = 0.1098
Largest diff. peak/hole / e Å^{-3}	0.20/-0.22
Flack parameter	-0.2(6)

Supplementary Table 17 Fractional Atomic Coordinates (×10^4) and Equivalent Isotropic Displacement Parameters (Å^2×10^3) for 9a. $U_{eq}$is defined as 1/3 of of the trace of the orthogonalised U_{IJ}tensor

Atom	*x*	*y*	*z*	U(eq)
F2	16168(3)	2591(3)	8397.9(5)	43.7(6)
F1	16450(3)	2538(3)	8877.9(5)	47.0(6)
F2B	-1535(3)	2694(3)	5983.5(5)	48.1(7)
F1B	-1530(3)	2918(3)	6464.5(5)	47.9(7)
O2B	5229(3)	2032(3)	5146.3(5)	28.4(6)
O1	9962(3)	2965(3)	9803.9(5)	29.4(6)
O1B	5427(3)	3189(3)	7284.6(5)	30.2(6)
O2	9095(3)	2775(3)	7646.7(5)	29.6(6)
C1B	2330(5)	2861(4)	6508.3(7)	24.2(8)
C8B	2318(5)	2451(4)	5940.3(7)	25.2(8)
C9	10904(5)	3727(4)	8375.9(7)	25.7(9)
C11	10197(5)	2640(4)	7891.9(7)	23.4(8)
C4	10915(5)	2944(4)	9539.5(7)	25.9(9)
C11B	4207(5)	2096(4)	5403.0(7)	23.7(8)
C8	12331(5)	2612(4)	8408.6(7)	24.9(8)
C6	11024(5)	1895(4)	9039.2(7)	26.3(9)
C4B	4347(5)	3141(4)	7037.0(7)	24.1(8)
C5	10184(5)	1971(4)	9315.6(7)	26.2(9)
C1	12620(5)	2767(4)	8981.3(7)	24.6(8)
C7B	1298(5)	2695(4)	6225.8(7)	25.3(9)
C13	12625(5)	1492(4)	8179.9(7)	26.4(8)
C13B	1788(5)	1301(4)	5728.6(7)	29.2(9)

C5B	4782(5)	1942(4)	6826.9(7)	26.2(8)
C10B	4797(5)	3218(4)	5617.7(7)	25.1(8)
C2	13296(6)	3749(5)	9208.5(7)	32.3(10)
C2B	1938(5)	4065(4)	6719.8(7)	27.5(9)
C12B	2732(6)	1118(4)	5460.5(7)	29.3(9)
C7	13483(5)	2645(4)	8682.4(8)	26.8(8)
C10	9864(5)	3754(4)	8121.7(7)	25.6(9)
C9B	3859(5)	3386(4)	5882.5(7)	25.1(9)
C15	15248(5)	2595(5)	8654.3(9)	33.7(9)
C15B	-465(6)	2758(5)	6224.2(8)	33.8(10)
C12	11572(5)	1495(4)	7922.5(7)	27.9(9)
C6B	3788(5)	1822(4)	6567.1(7)	27.0(9)
C3B	2928(5)	4213(4)	6981.9(7)	29.1(10)
C3	12467(6)	3845(4)	9486.8(7)	31.3(10)
C16B	4938(6)	4302(4)	7521.1(8)	33.7(9)
C14B	4513(6)	1089(4)	4903.1(7)	34.7(10)
C14	9523(5)	1766(4)	7393.3(7)	31.9(9)
C16	10722(6)	3855(4)	10050.5(7)	37.5(11)

Supplementary Table 18 Anisotropic Displacement Parameters (Å^2×10^3) for 9a. The Anisotropic displacement factor exponent takes the form: -2π^2[h^{2}a*^{2}U$_{11}$+2hka*b*U$_{12}$+…]

Atom	U11	U22	U33	U23	U13	U12
F2	29.1(13)	38.7(14)	63.3(14)	-6.8(12)	14.8(12)	-4.5(12)
F1	28.4(13)	40.4(15)	72.1(16)	-1.0(12)	-13.9(12)	0.6(13)
F2B	28.2(13)	46.0(16)	70.1(15)	-2.3(12)	-15.2(12)	1.0(13)
F1B	28.6(13)	46.4(15)	68.8(15)	-1.2(12)	15.8(12)	-1.2(13)
O2B	33.6(16)	29.4(13)	22.1(12)	-2.8(10)	-3.3(12)	-2.2(13)
O1	35.4(16)	27.3(13)	25.4(12)	-1.8(10)	-6.3(12)	1.6(13)
O1B	35.5(16)	28.9(13)	26.1(13)	-1.9(10)	3.3(12)	2.1(14)
O2	36.0(15)	28.4(14)	24.4(12)	-3.7(11)	1.2(12)	1.1(14)
C1B	22(2)	20.8(18)	29.4(18)	6.0(15)	7.6(17)	-0.5(19)
C8B	24(2)	20.7(19)	30.7(18)	9.7(15)	-5.0(16)	0.7(19)
C9	31(2)	19.9(18)	26.1(18)	-0.4(14)	5.2(17)	3.9(19)
C11	25(2)	21.1(18)	23.8(17)	2.0(14)	8.2(16)	-2.7(19)
C4	32(2)	20.3(18)	25.0(18)	5.8(14)	-7.2(17)	6(2)
C11B	26(2)	22.8(18)	22.3(17)	4.1(15)	-6.1(16)	0.5(19)
C8	24(2)	21.3(19)	29.1(18)	1.5(15)	7.4(16)	-4.8(19)
C6	27(2)	24.7(19)	26.7(18)	0.3(14)	-7.3(17)	-4.0(19)
C4B	25(2)	24.8(18)	22.7(17)	5.2(14)	6.4(16)	-4.3(19)
C5	27(2)	22.8(18)	28.9(18)	3.1(15)	-7.5(17)	-5.8(19)
C1	25(2)	20.5(19)	28.4(17)	2.8(15)	-5.5(17)	0.5(19)
C7B	22(2)	20.3(19)	34.0(19)	5.3(16)	2.5(16)	0.9(19)
C13	21(2)	23.1(19)	35.4(19)	2.3(16)	7.7(17)	0.4(19)
C13B	25(2)	27(2)	36(2)	5.2(16)	-7.4(18)	-8.6(19)

C5B	29(2)	21.2(17)	27.9(18)	4.3(15)	6.1(17)	2.9(19)
C10B	23(2)	25.5(18)	26.7(18)	1.8(14)	-3.6(16)	-1.4(19)
C2	31(2)	29(2)	37(2)	7.1(17)	-6.9(19)	-8(2)
C2B	24(2)	26(2)	33.2(19)	6.7(15)	10.6(18)	7.1(18)
C12B	38(3)	23.5(19)	26.2(18)	-0.2(15)	-8.0(18)	-7(2)
C7	27(2)	16.7(19)	36(2)	0.9(16)	-3.9(17)	-0.7(18)
C10	29(2)	24.5(18)	23.8(17)	2.8(14)	6.0(17)	0.4(19)
C9B	28(2)	24.9(19)	22.8(17)	0.4(15)	-3.3(17)	-2.1(19)
C15	26(2)	28(2)	47(2)	0.5(19)	-2(2)	-3(2)
C15B	26(2)	26(2)	49(2)	0.9(18)	1(2)	1(2)
C12	30(2)	22.9(19)	30.3(19)	-1.4(16)	11.7(18)	-2(2)
C6B	29(2)	21.2(19)	31.0(19)	2.1(14)	8.8(17)	1.4(19)
C3B	34(3)	28(2)	25.1(18)	1.7(15)	14.5(18)	4(2)
C3	38(3)	29(2)	27.8(19)	2.5(16)	-10.5(18)	-3(2)
C16B	37(2)	36(2)	27.8(18)	-8.9(16)	8.4(18)	0(2)
C14B	44(3)	31(2)	29.0(19)	-7.2(16)	-8.4(19)	0(2)
C14	39(2)	31(2)	26.4(18)	-5.9(15)	6.8(18)	1(2)
C16	50(3)	31(2)	32(2)	-8.9(16)	-10(2)	0(2)

Supplementary Table 19 Bond Lengths for 9a

Atom	Atom	Length/Å	Atom	Atom	Length/Å
F2	C15	1.333(4)	C11	C12	1.387(5)
F1	C15	1.339(4)	C4	C5	1.387(5)
F2B	C15B	1.337(4)	C4	C3	1.383(5)
F1B	C15B	1.339(4)	C11B	C10B	1.396(5)
O2B	C11B	1.375(4)	C11B	C12B	1.376(5)
O2B	C14B	1.433(4)	C8	C13	1.388(5)
O1	C4	1.377(4)	C8	C7	1.493(5)
O1	C16	1.435(4)	C6	C5	1.385(4)
O1B	C4B	1.366(4)	C6	C1	1.402(5)
O1B	C16B	1.440(4)	C4B	C5B	1.394(5)
O2	C11	1.371(4)	C4B	C3B	1.388(5)
O2	C14	1.436(4)	C1	C2	1.387(5)
C1B	C7B	1.483(5)	C1	C7	1.485(4)
C1B	C2B	1.394(5)	C7B	C15B	1.304(6)
C1B	C6B	1.396(5)	C13	C12	1.390(5)
C8B	C7B	1.497(4)	C13B	C12B	1.395(5)
C8B	C13B	1.389(5)	C5B	C6B	1.379(5)
C8B	C9B	1.395(5)	C10B	C9B	1.379(4)
C9	C8	1.401(5)	C2	C3	1.389(5)
C9	C10	1.373(4)	C2B	C3B	1.387(5)
C11	C10	1.393(4)	C7	C15	1.312(5)

Supplementary Table 20 Bond Angles for 9a

Atom	Atom	Atom	Angle/°	Atom	Atom	Atom	Angle/°
C11B	O2B	C14B	116.8(3)	C6	C1	C7	119.6(3)
C4	O1	C16	117.8(3)	C2	C1	C6	117.5(3)
C4B	O1B	C16B	117.8(3)	C2	C1	C7	122.9(3)
C11	O2	C14	117.0(3)	C1B	C7B	C8B	118.7(3)
C2B	C1B	C7B	122.4(3)	C15B	C7B	C1B	121.0(3)
C2B	C1B	C6B	117.4(3)	C15B	C7B	C8B	120.3(3)
C6B	C1B	C7B	120.2(3)	C8	C13	C12	121.4(3)
C13B	C8B	C7B	121.9(3)	C8B	C13B	C12B	121.1(4)
C13B	C8B	C9B	118.2(3)	C6B	C5B	C4B	119.6(3)
C9B	C8B	C7B	119.8(3)	C9B	C10B	C11B	119.9(3)
C10	C9	C8	121.3(3)	C1	C2	C3	122.0(4)
O2	C11	C10	115.7(3)	C3B	C2B	C1B	121.7(3)
O2	C11	C12	124.7(3)	C11B	C12B	C13B	119.7(3)
C12	C11	C10	119.7(3)	C1	C7	C8	119.6(3)
O1	C4	C5	115.4(3)	C15	C7	C8	119.3(3)
O1	C4	C3	124.4(3)	C15	C7	C1	121.1(3)
C3	C4	C5	120.3(3)	C9	C10	C11	120.1(3)
O2B	C11B	C10B	115.3(3)	C10B	C9B	C8B	121.0(3)
O2B	C11B	C12B	124.7(3)	F2	C15	F1	107.7(3)
C12B	C11B	C10B	119.9(3)	C7	C15	F2	126.2(4)
C9	C8	C7	120.3(3)	C7	C15	F1	126.2(4)
C13	C8	C9	117.9(3)	F2B	C15B	F1B	107.6(3)
C13	C8	C7	121.8(3)	C7B	C15B	F2B	126.5(4)

C5	C6	C1	121.3(3)	C7B	C15B	F1B	126.0(4)
O1B	C4B	C5B	115.6(3)	C11	C12	C13	119.6(3)
O1B	C4B	C3B	124.6(3)	C5B	C6B	C1B	121.9(3)
C3B	C4B	C5B	119.8(3)	C2B	C3B	C4B	119.6(3)
C6	C5	C4	119.7(3)	C4	C3	C2	119.3(3)

Supplementary Table 21 Hydrogen Atom Coordinates (Å×10^4) and Isotropic Displacement Parameters (Å^2×10^3) for 9a

Atom	*x*	*y*	*z*	U(eq)
H9	10655.9	4462.7	8529.35	31
H6	10519.98	1252.09	8889.01	32
H5	9135.29	1373.2	9350.88	31
H13	13546.61	722.68	8199.33	32
H13B	788.03	641	5766.13	35
H5B	5737.56	1225.66	6861.85	31
H10B	5820.59	3850.38	5582.28	30
H2	14334.97	4361.74	9173.57	39
H2B	988.17	4786.76	6684.43	33
H12B	2366.12	338.58	5321.34	35
H10	8935.88	4516.2	8102.87	31
H9B	4260.21	4134.48	6024.75	30
H12	11787.04	734.84	7771.73	33
H6B	4096.43	1026.84	6427.06	32
H3B	2642.46	5025.52	7119.88	35
H3	12951.36	4507.51	9635.97	38
H16D	4966.89	5407.19	7447.23	51
H16E	5779.39	4191.86	7683.28	51
H16F	3741.66	4048.94	7590.38	51
H14D	5228.96	1274.1	4727.29	52
H14E	3286.96	1416.82	4865.32	52
H14F	4542.09	-54.57	4953.76	52
H14A	8705.87	2009.49	7232.55	48

H14B	9409.48	631.54	7447.53	48
H14C	10741.16	1982.08	7330.3	48
H16A	10798.61	4997.43	10000.22	56
H16B	11910.38	3441.73	10093.05	56
H16C	9966.96	3721.02	10223.37	56

7. NMR Spectra

^{1}H NMR of **1b**

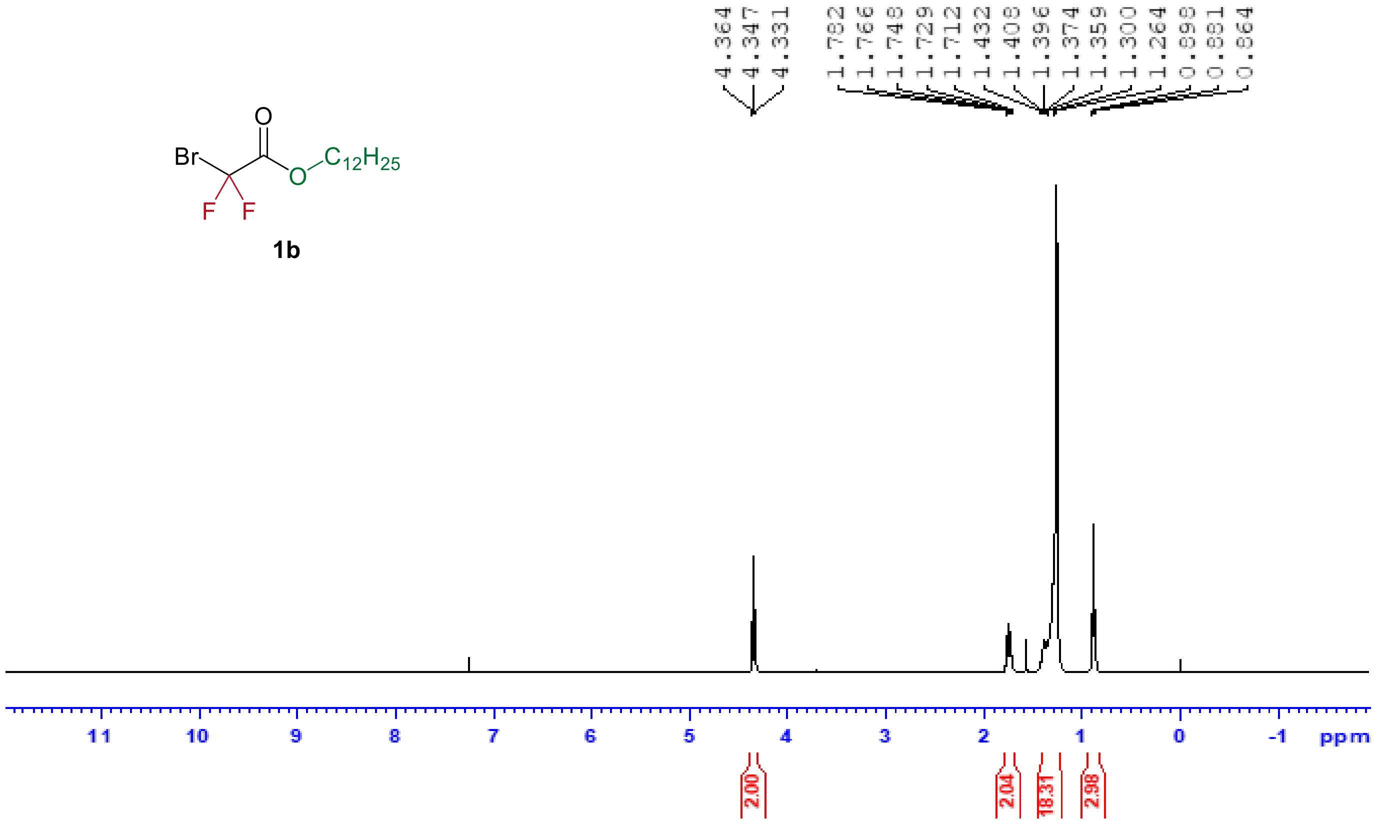

^{13}C NMR of **1b**

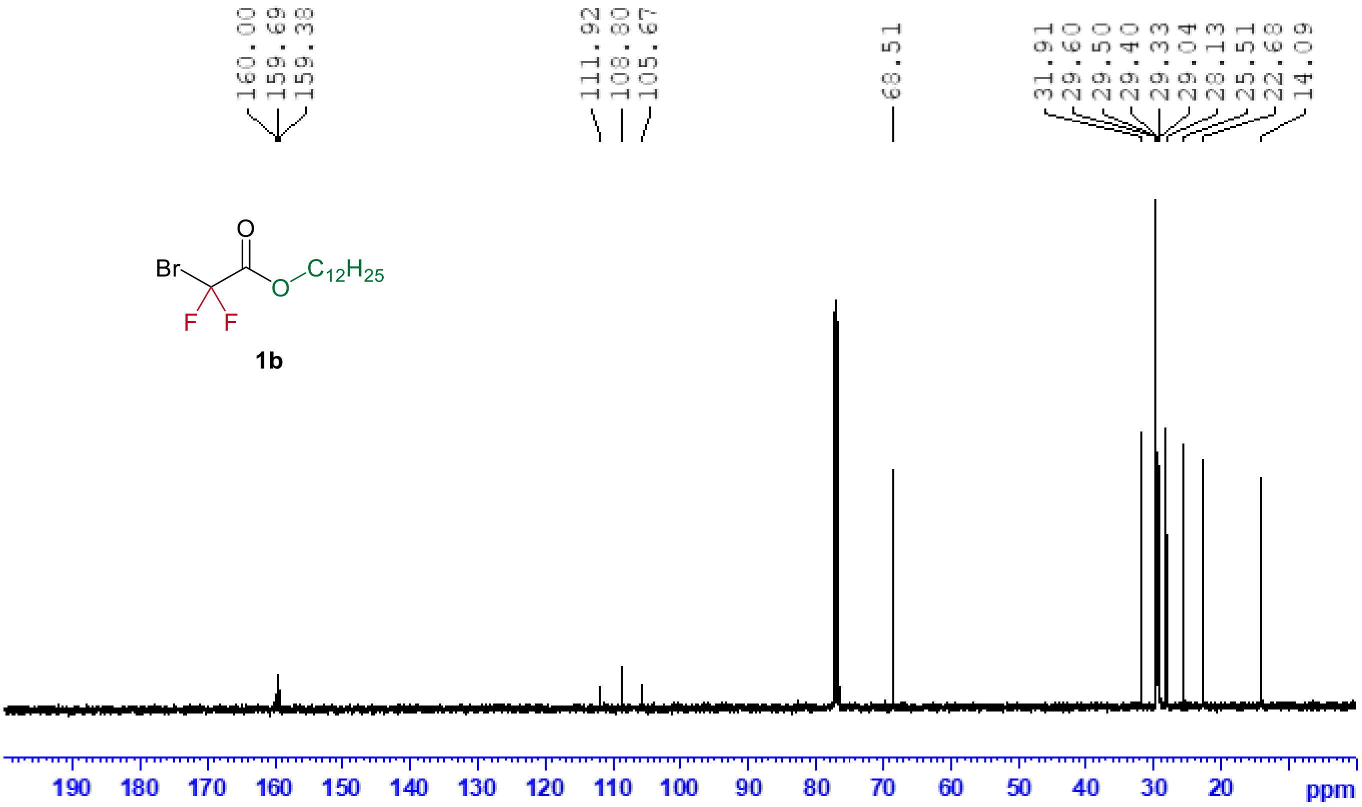

^{19}F NMR of **1b**

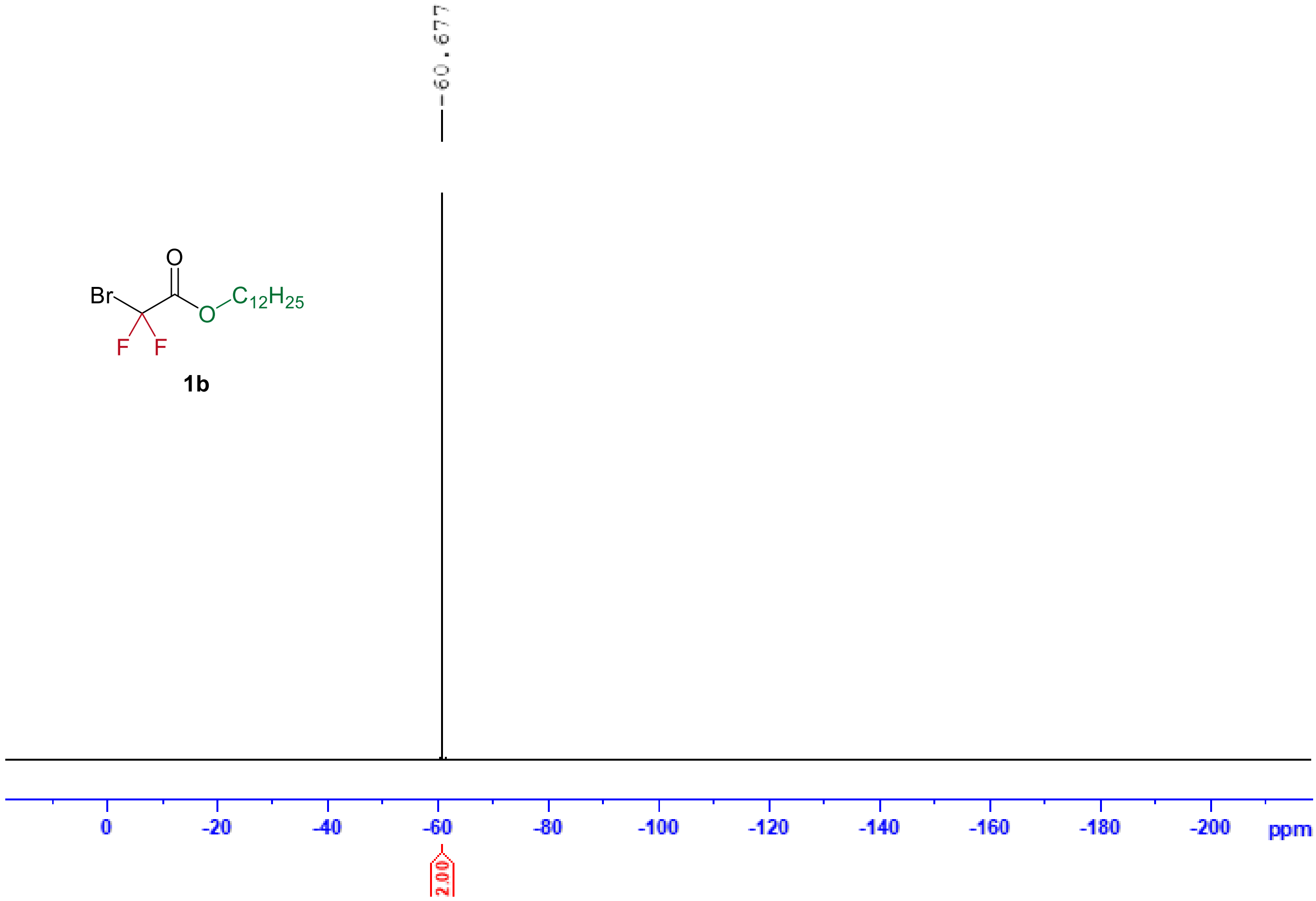

-60.677
Br
O
O
$C_{12}H_{25}$
F
F
1b
0
-20
-40
-60
-80
-100
-120
-140
-160
-180
-200
ppm
2.00

^{1}H NMR of **1c**

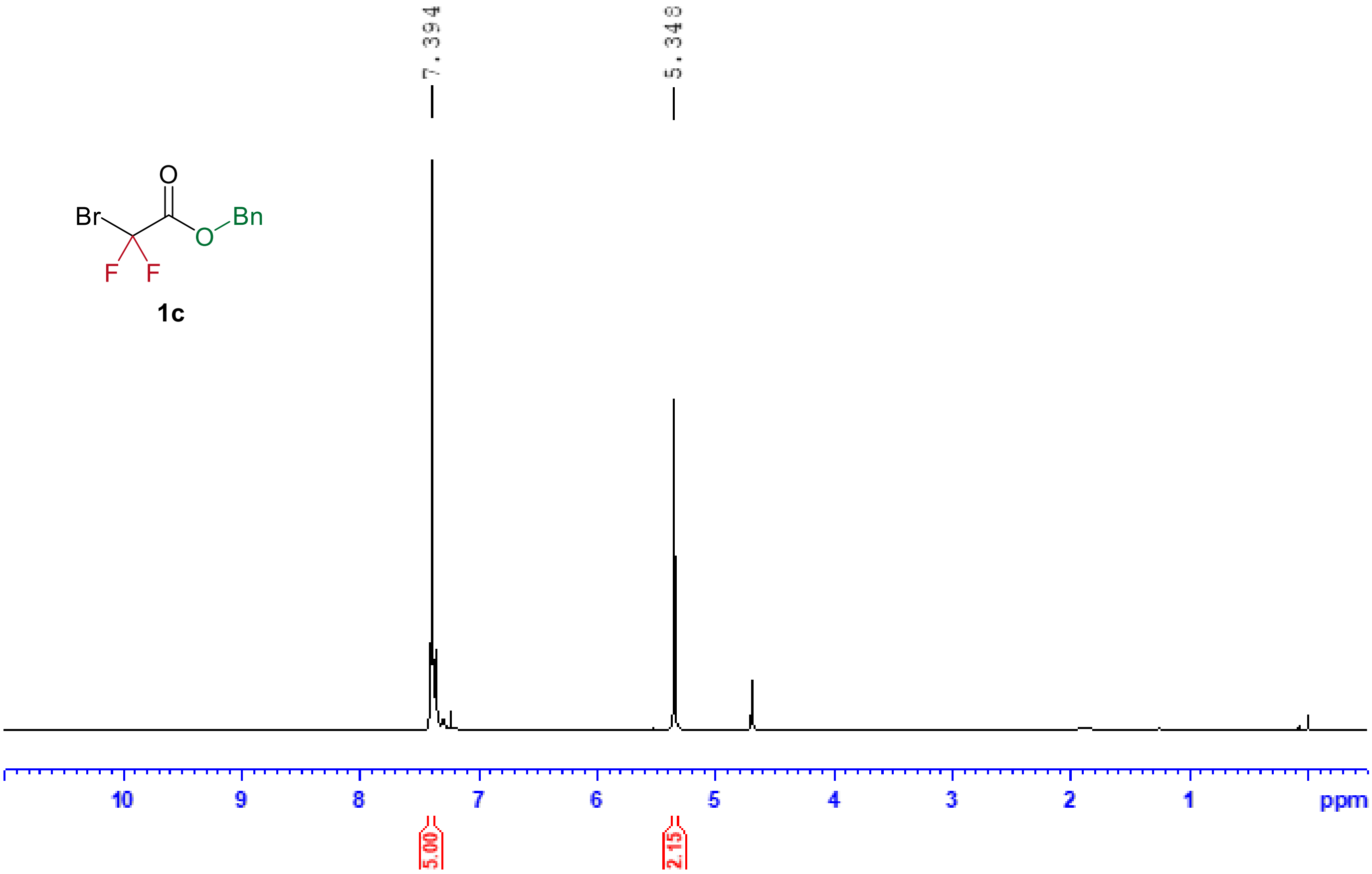

^{13}C NMR of **1c**

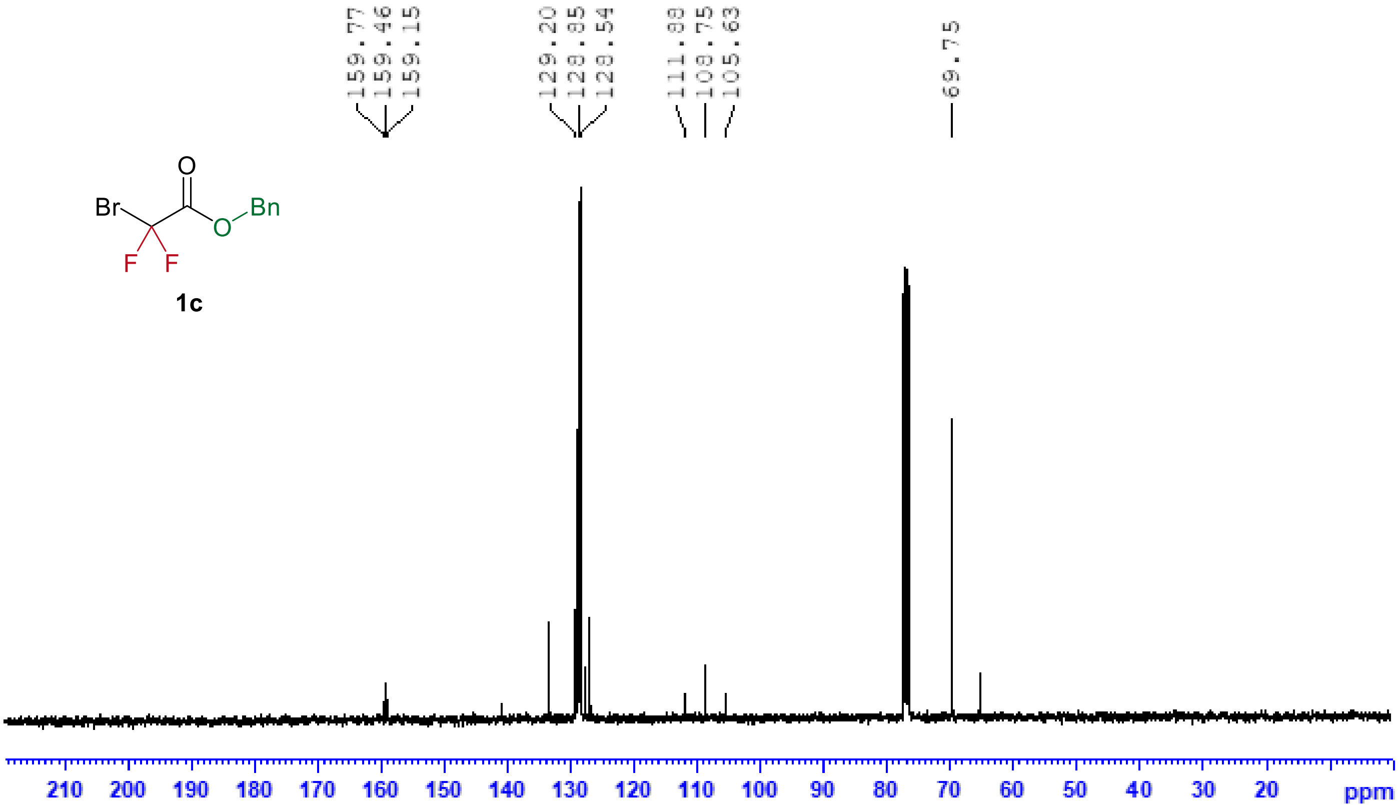

^{19}F NMR of **1c**

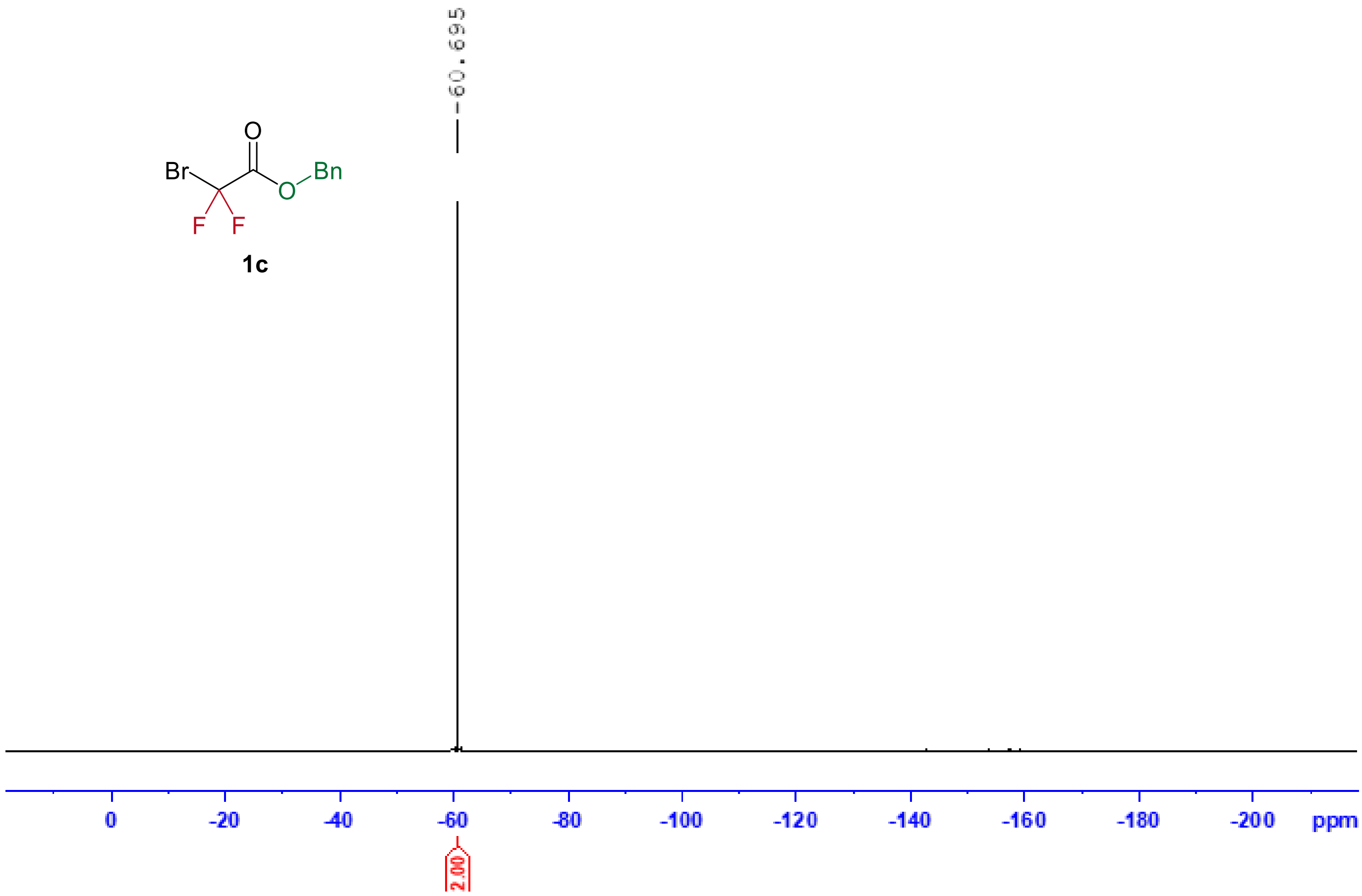

^{1}H NMR of **1g**

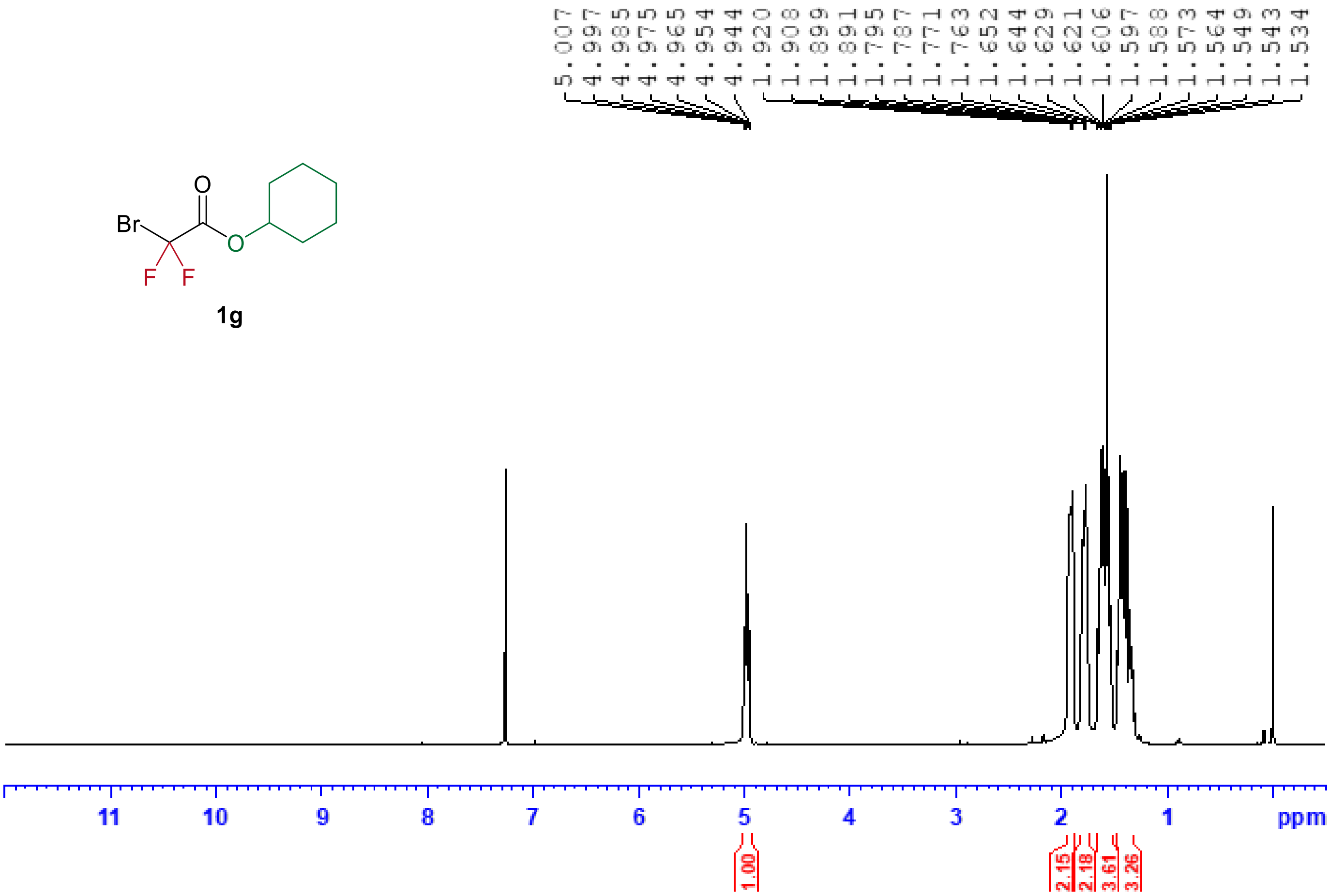

^{13}C NMR of **1g**

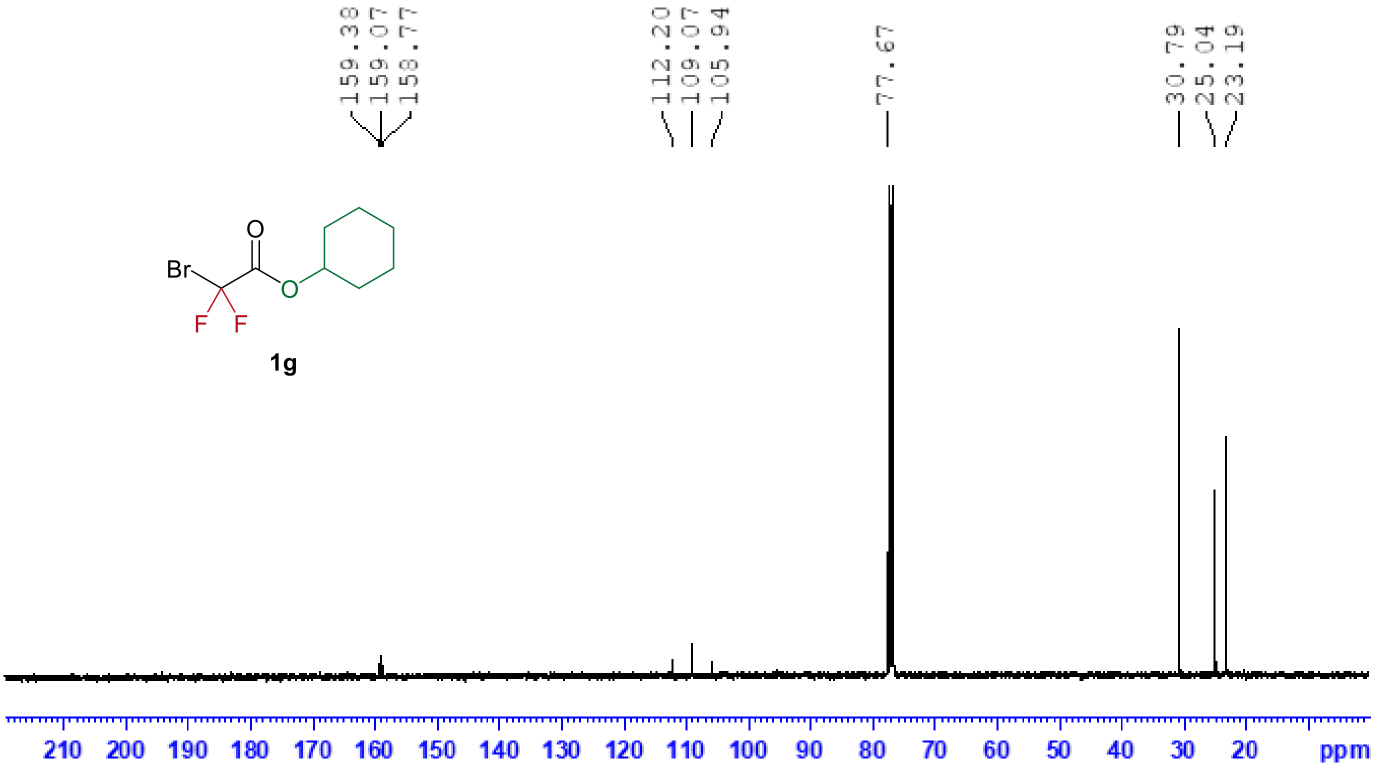

^{19}F NMR of **1g**

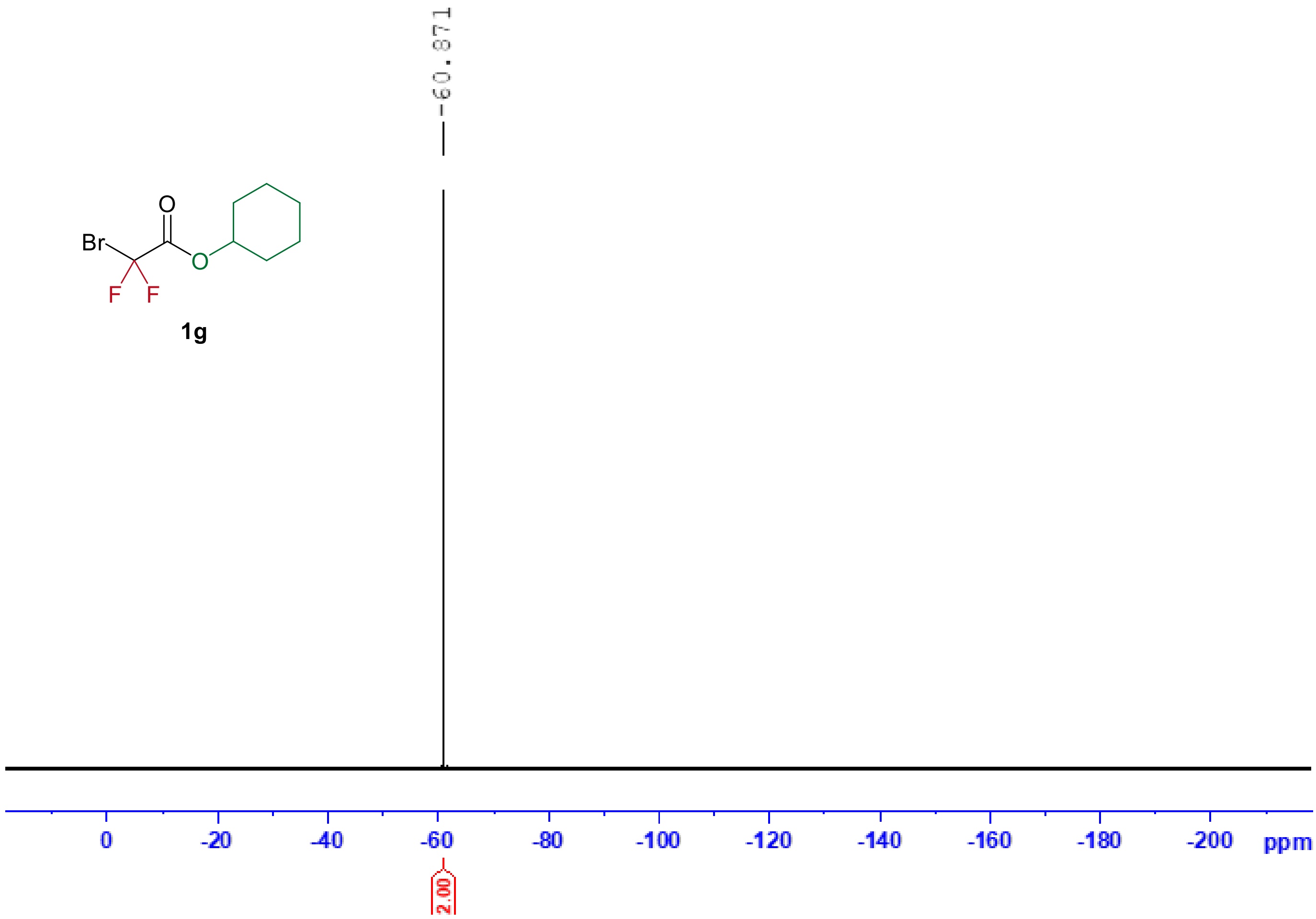

^{1}H NMR of **1h**

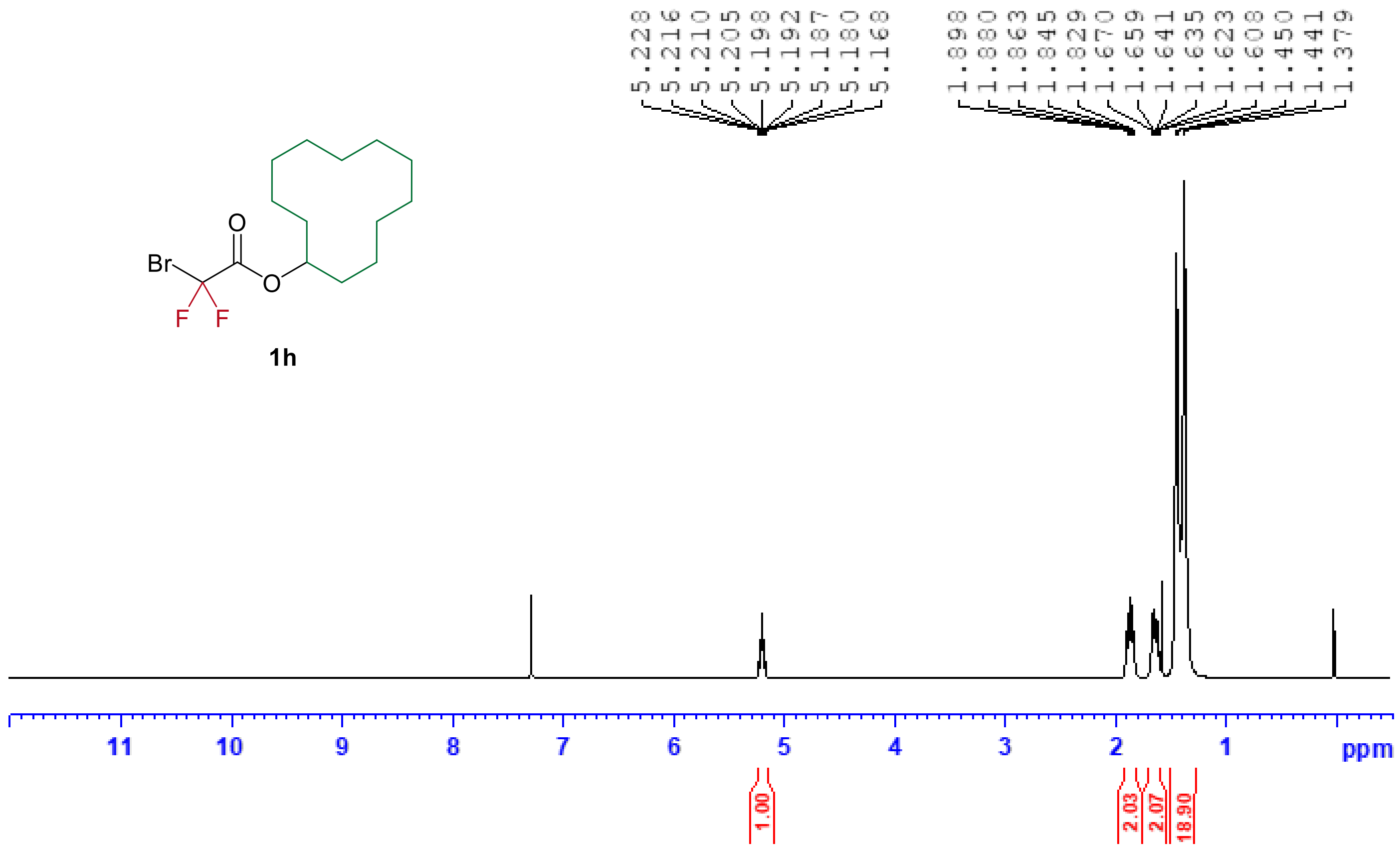

^{13}C NMR of **1h**

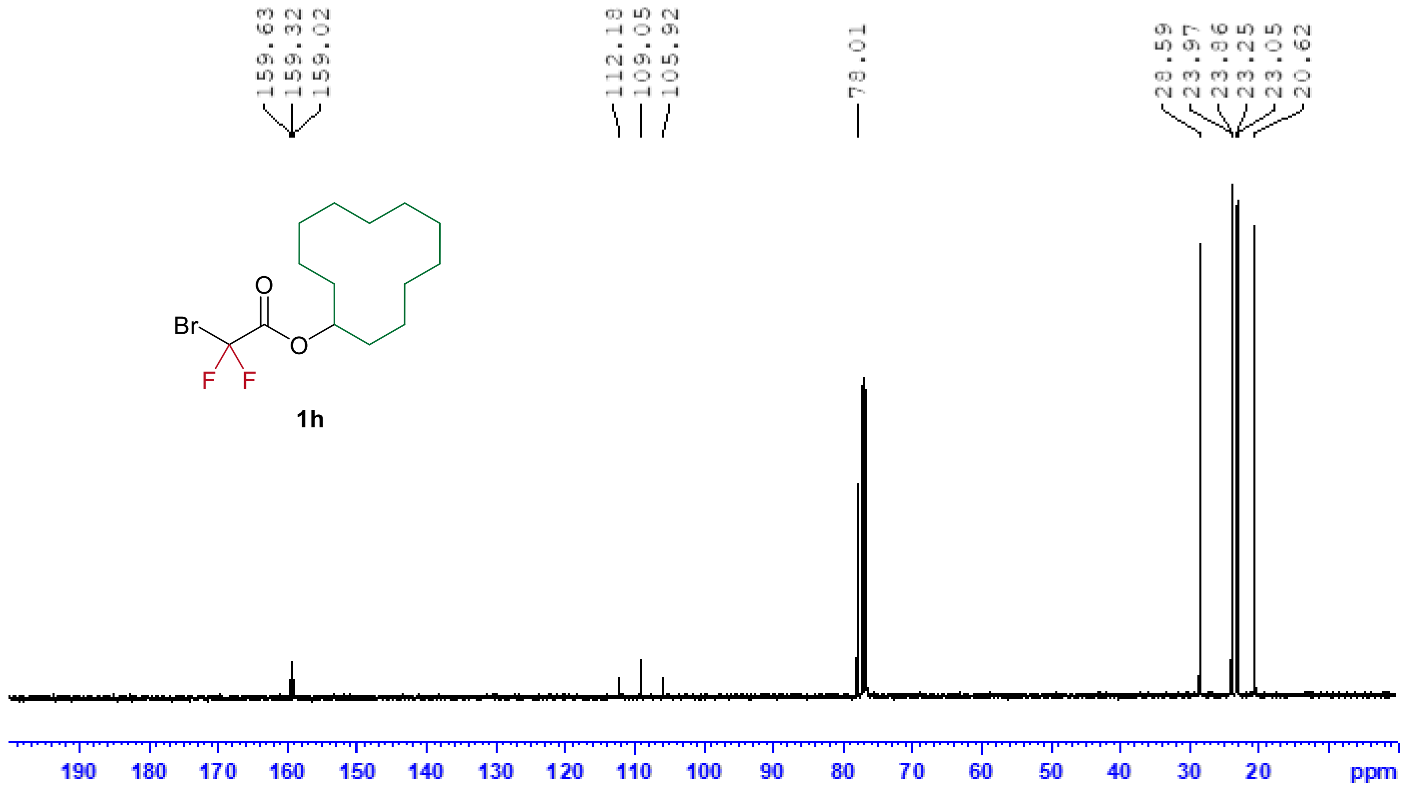

^{19}F NMR of **1h**

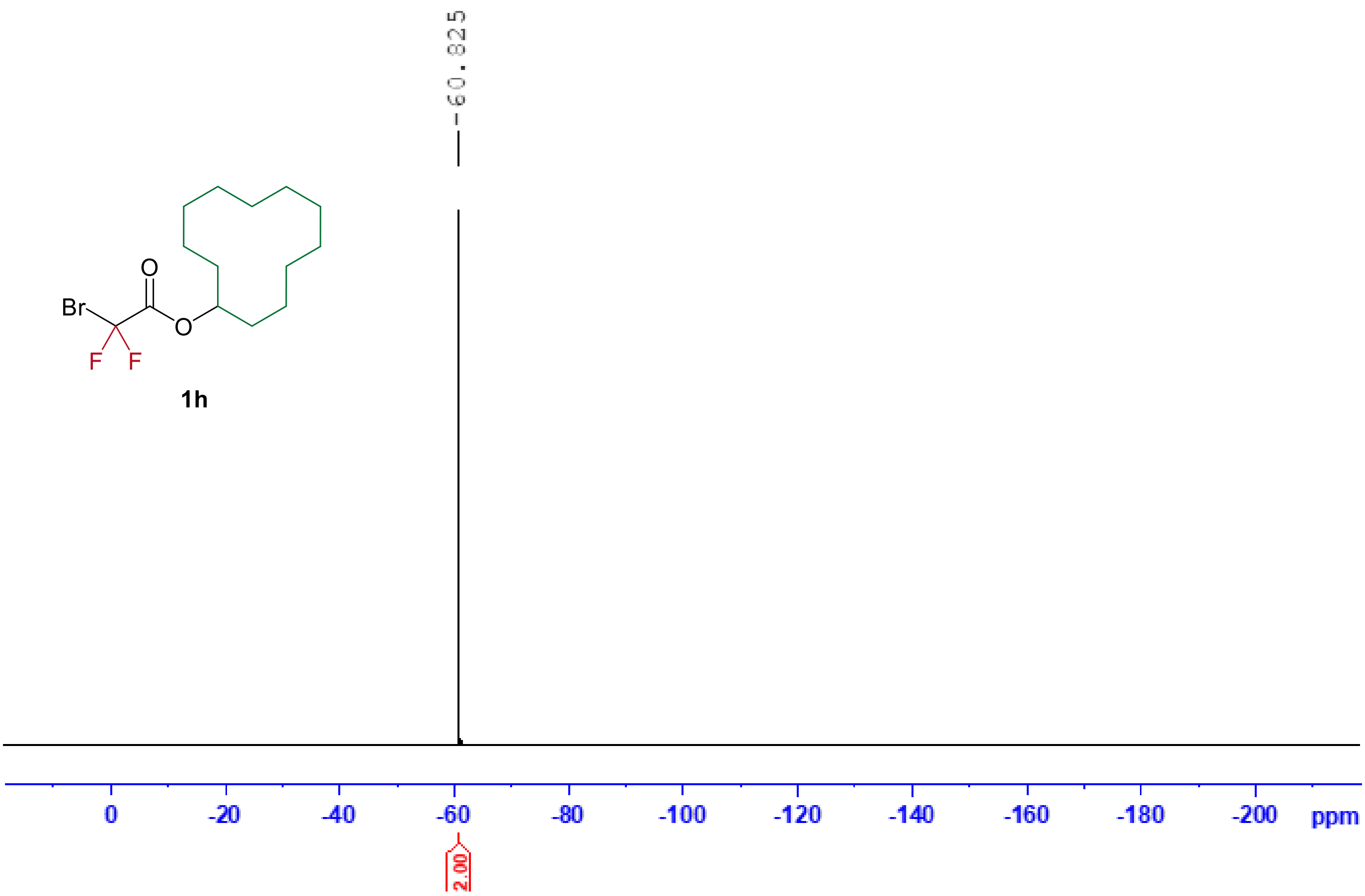

^{1}H NMR of **1i**

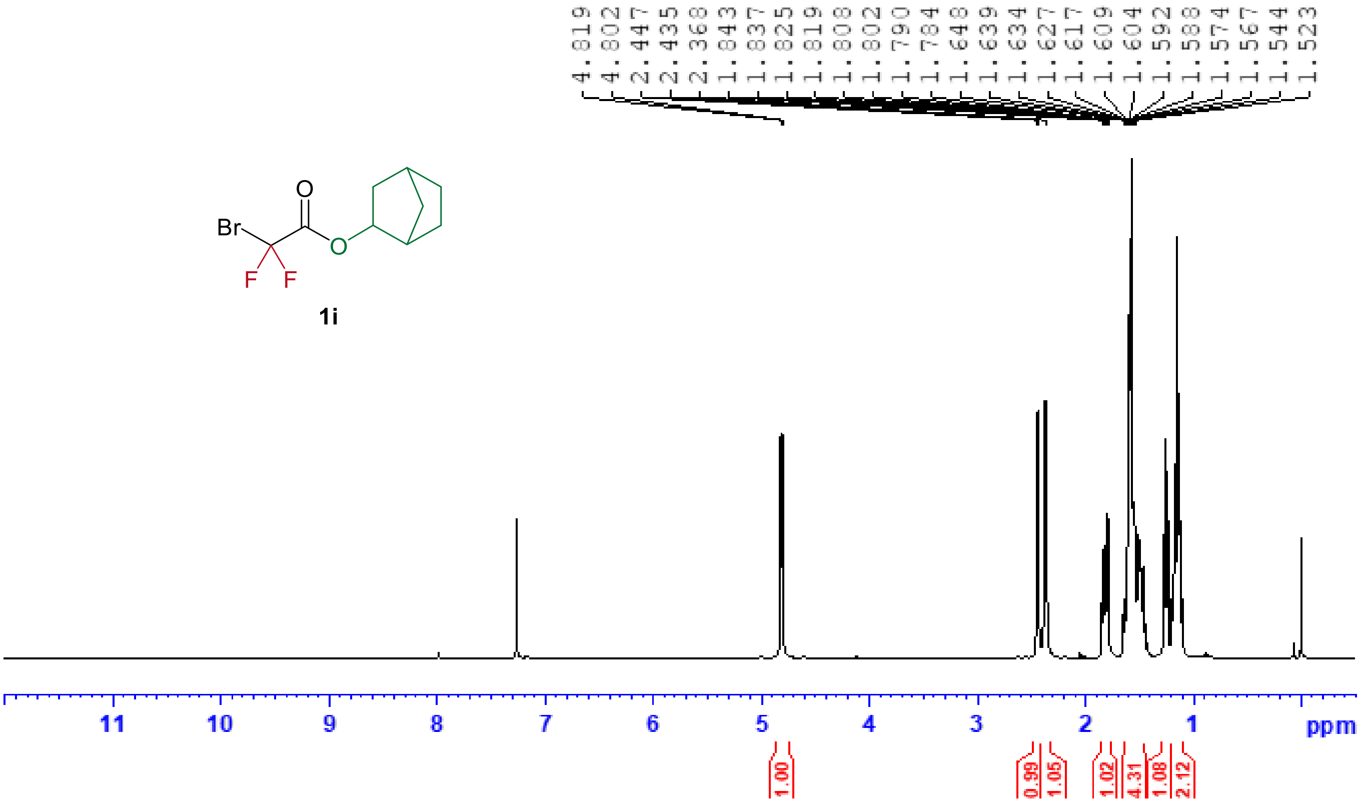

^{13}C NMR of **1i**

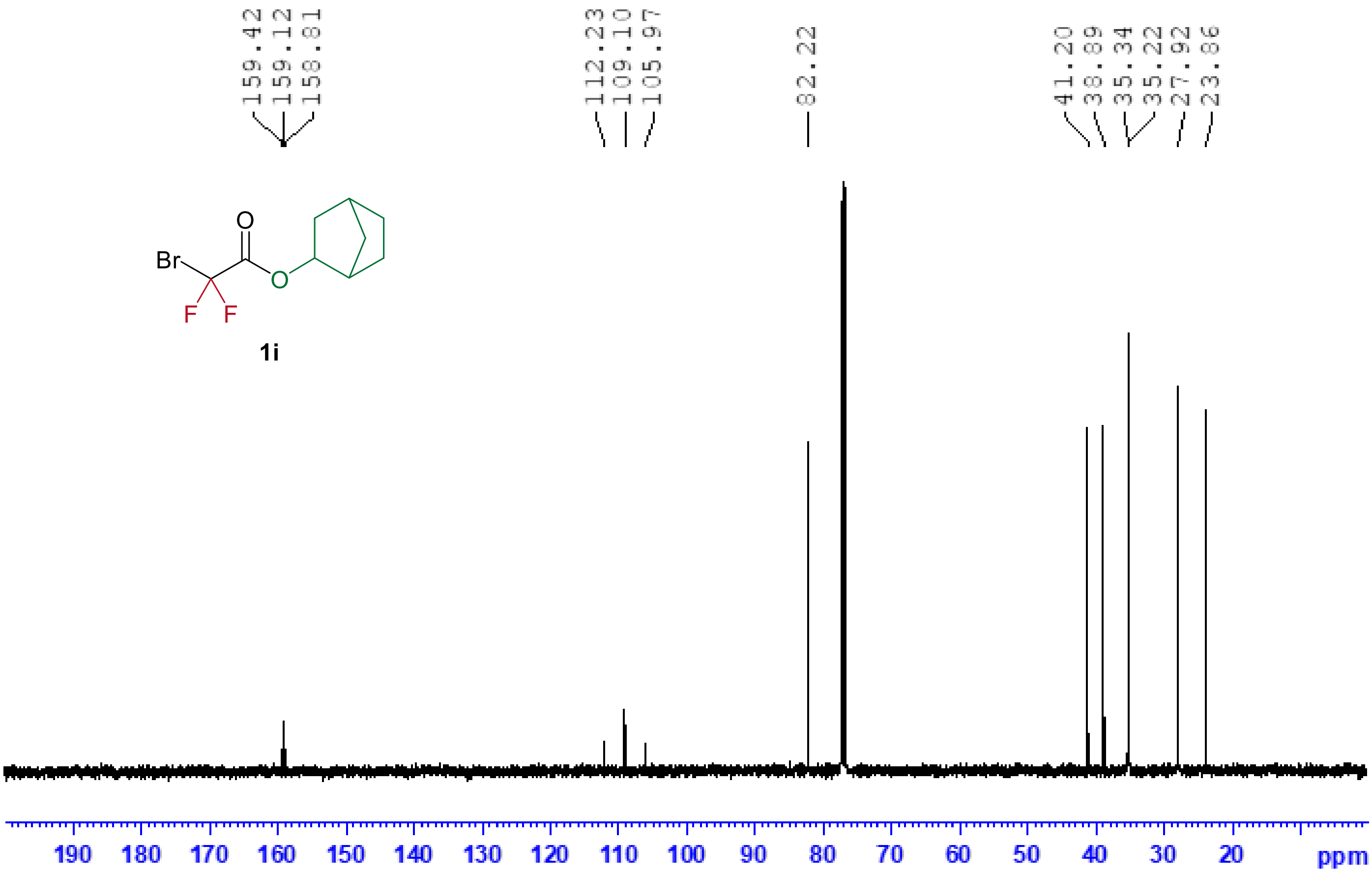

^{19}F NMR of **1i**

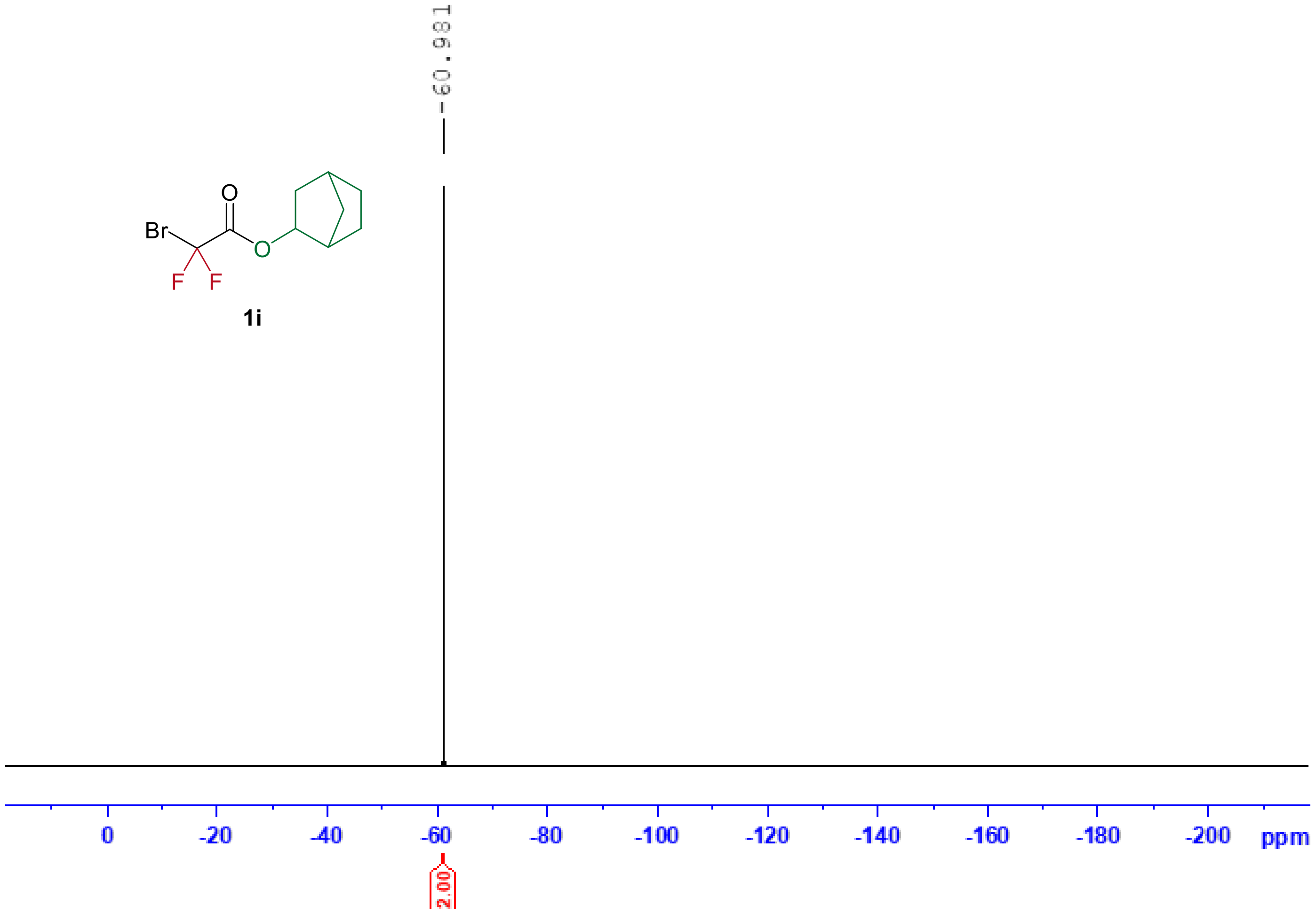

^{1}H NMR of **1j**

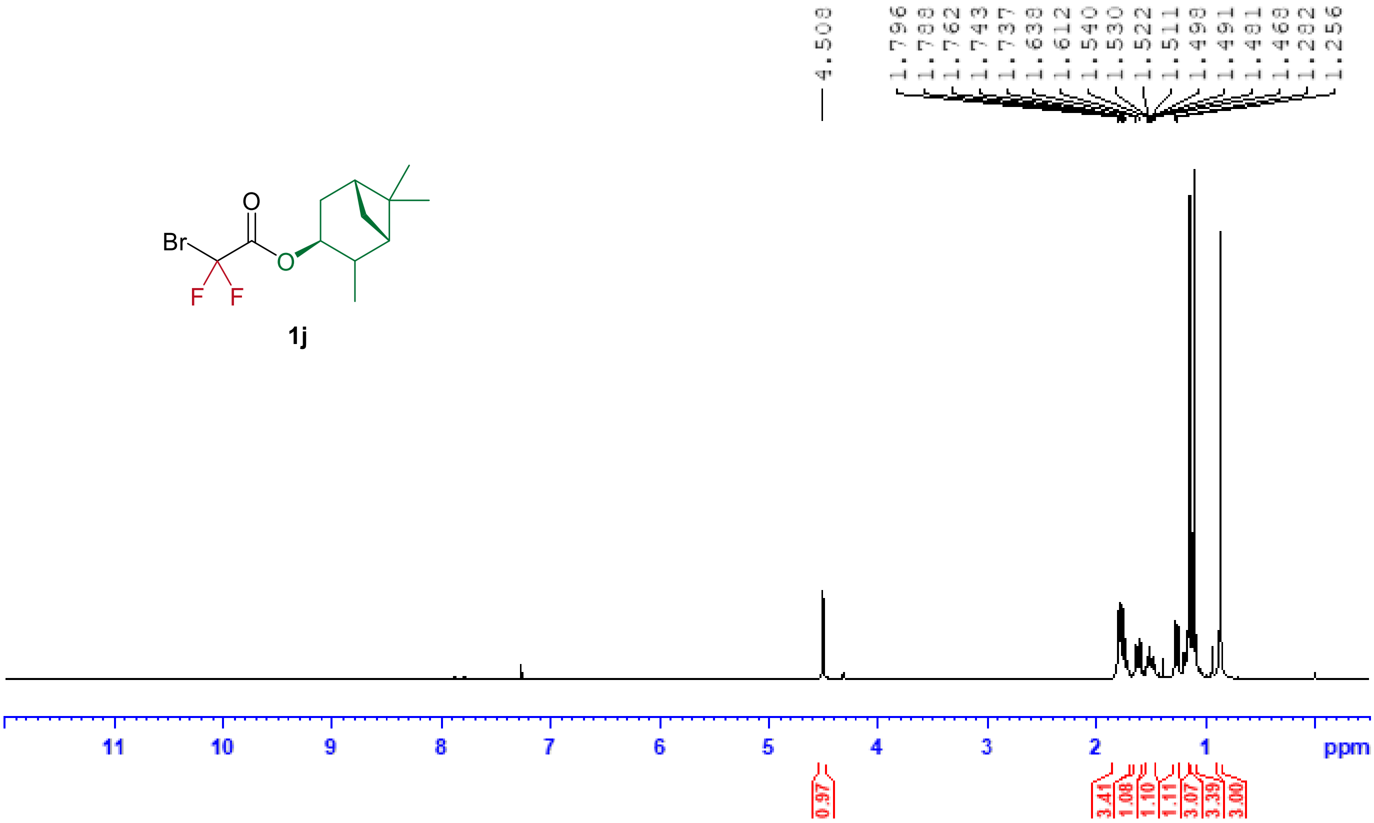

^{13}C NMR of **1j**

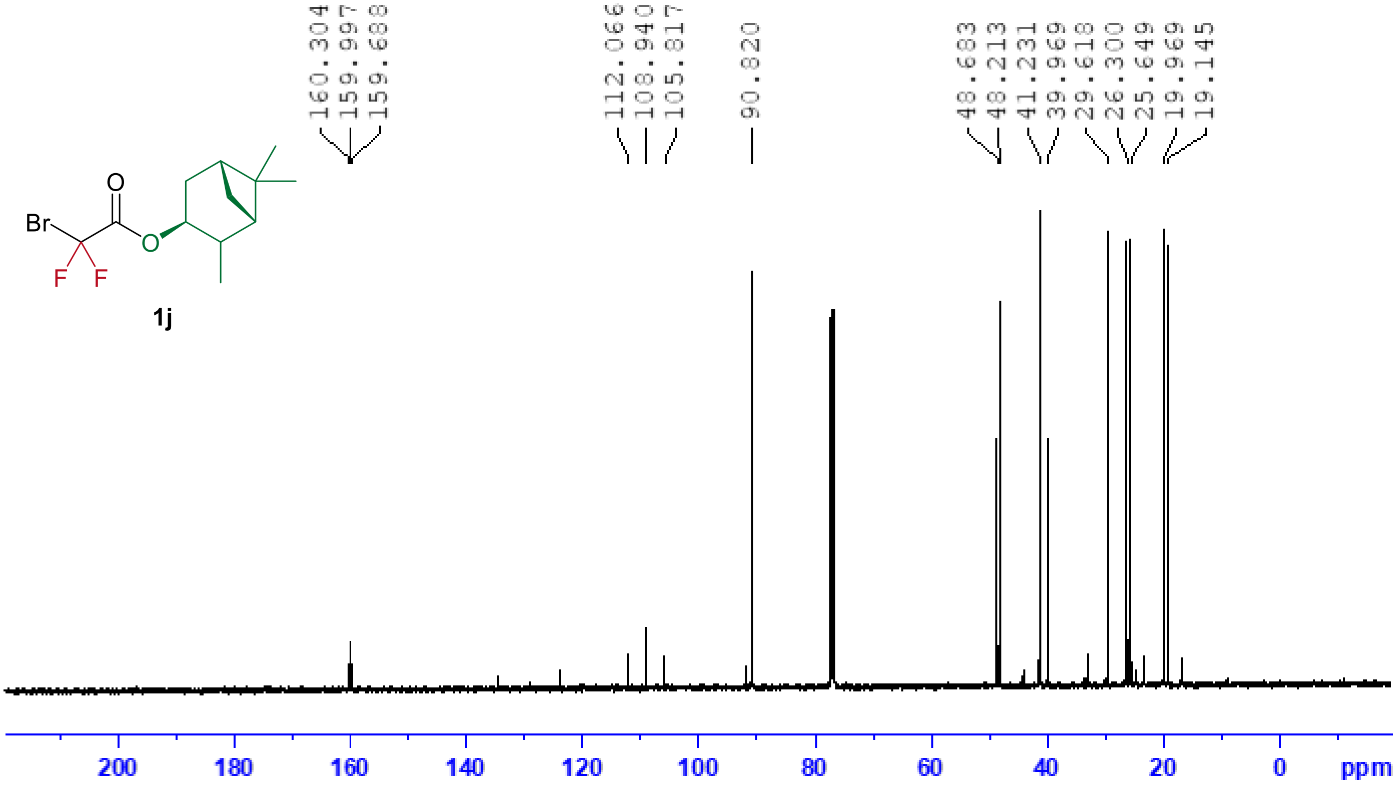

^{19}F NMR of **1j**

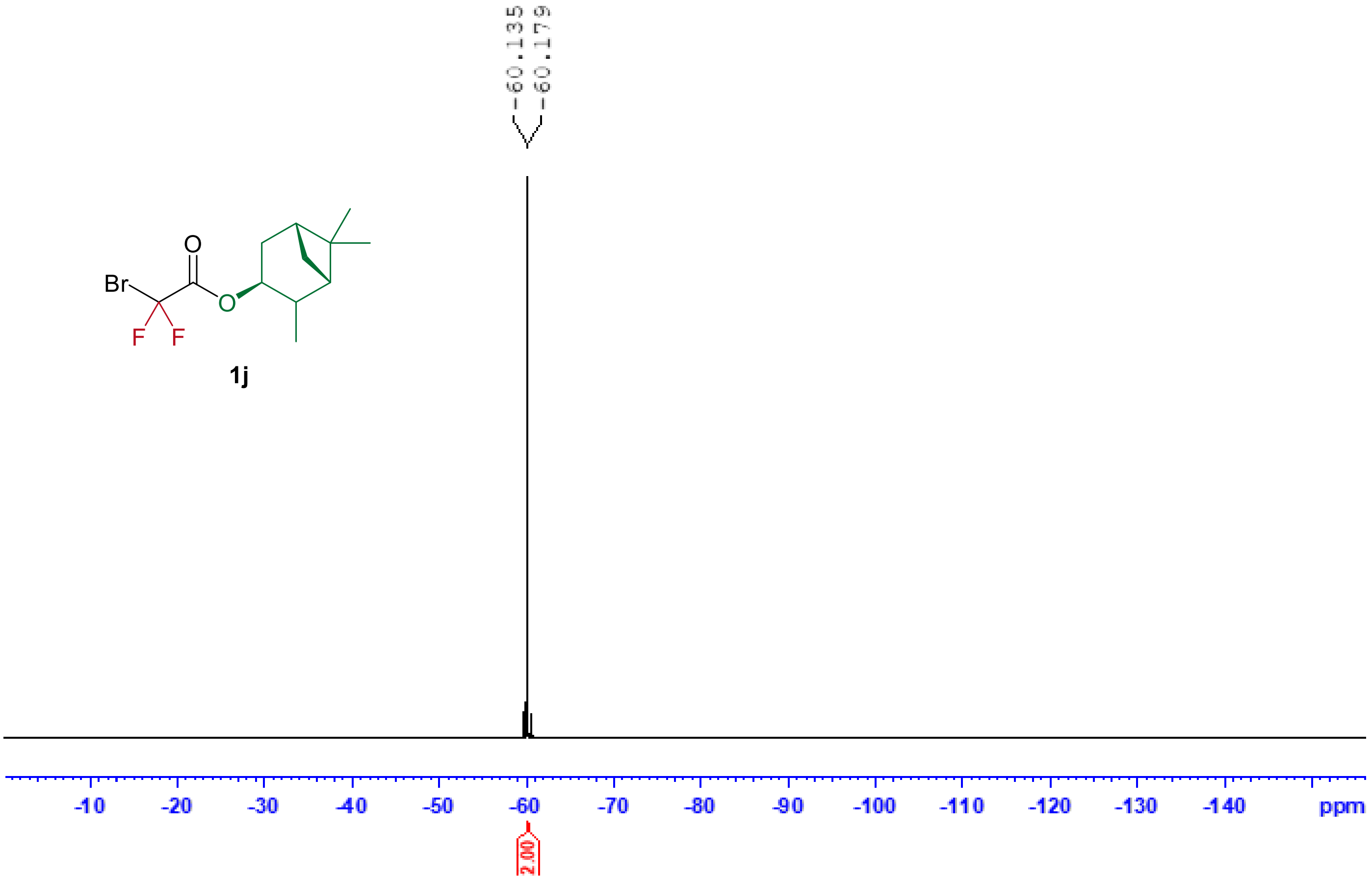
-60.135
-60.179
O
Br
O
F F
1j
-10 -20 -30 -40 -50 -60 -70 -80 -90 -100 -110 -120 -130 -140 ppm
2.00

^{1}H NMR of **1k**

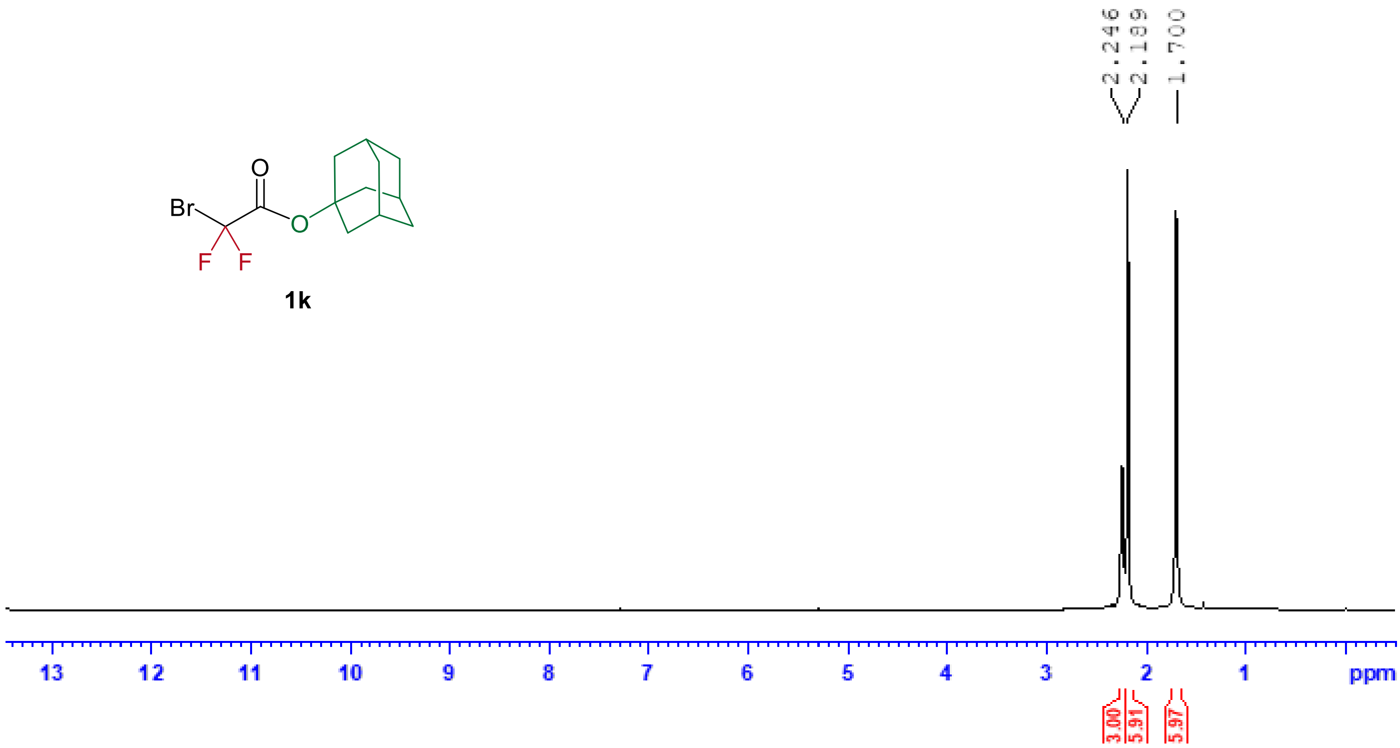

^{13}C NMR of **1k**

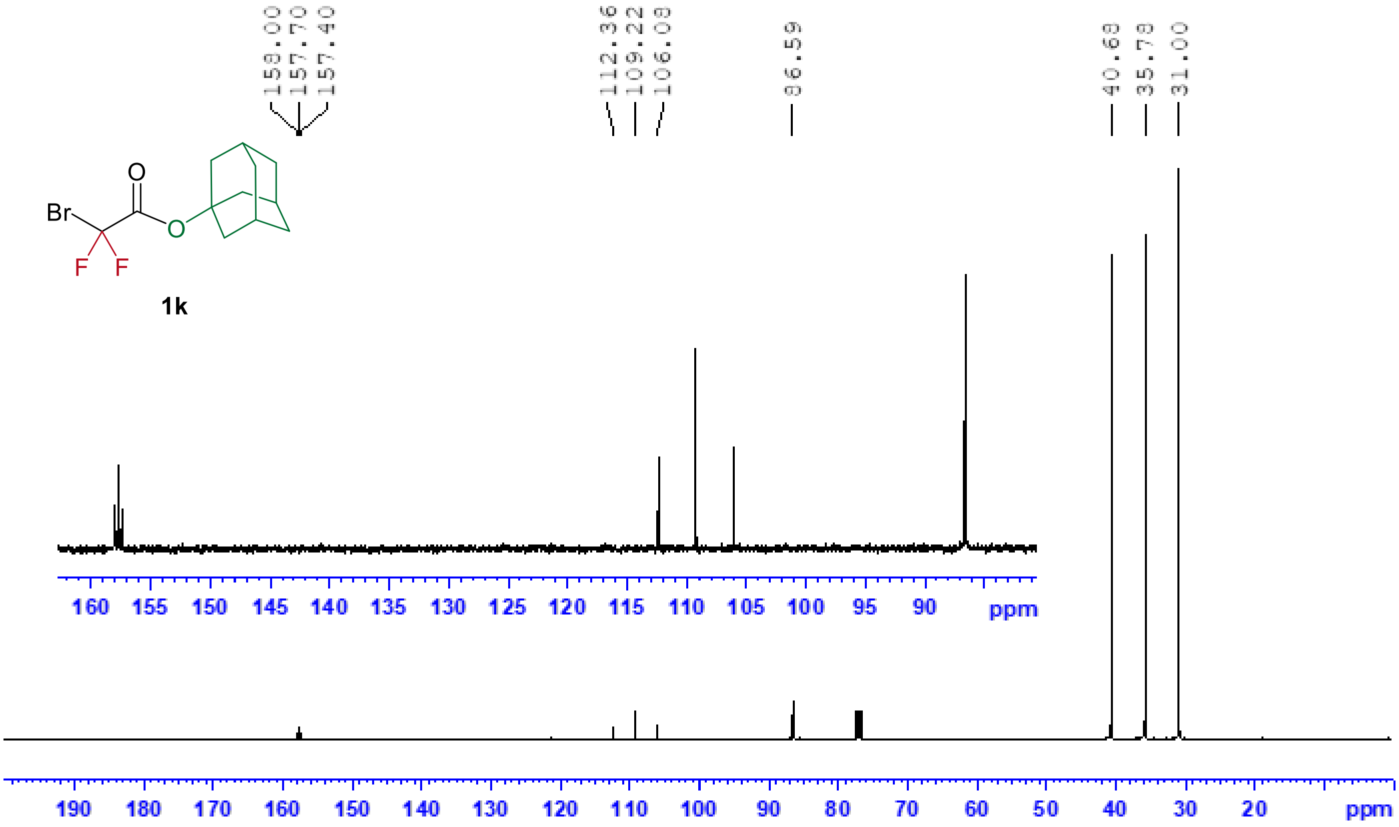

^{19}F NMR of **1k**

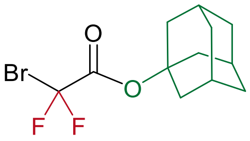

1k

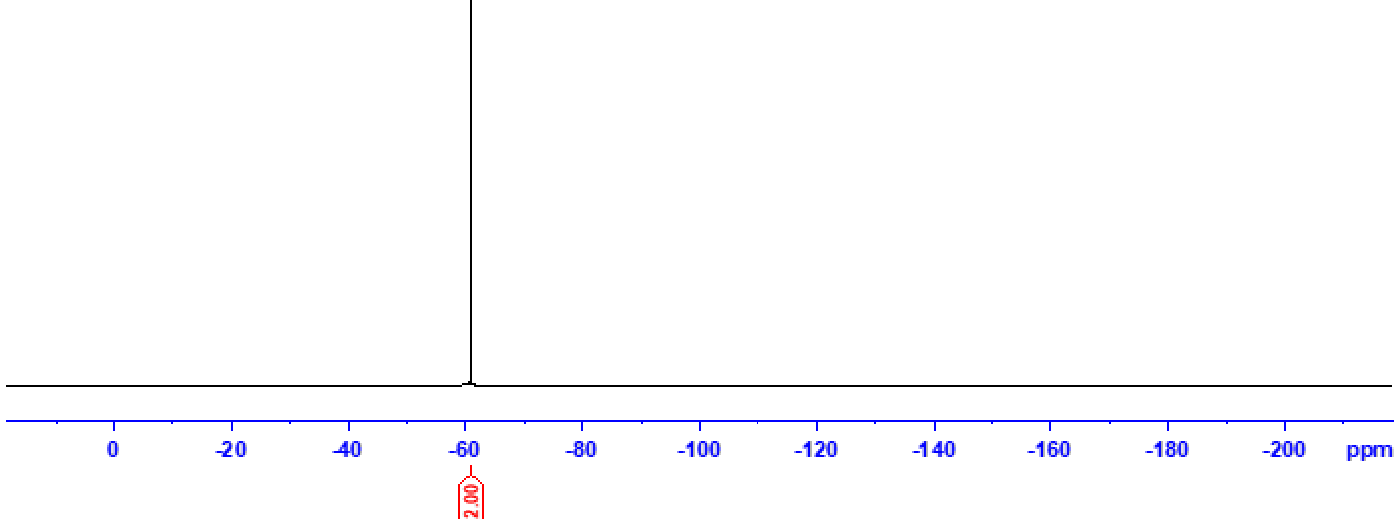

^{1}H NMR of **1l**

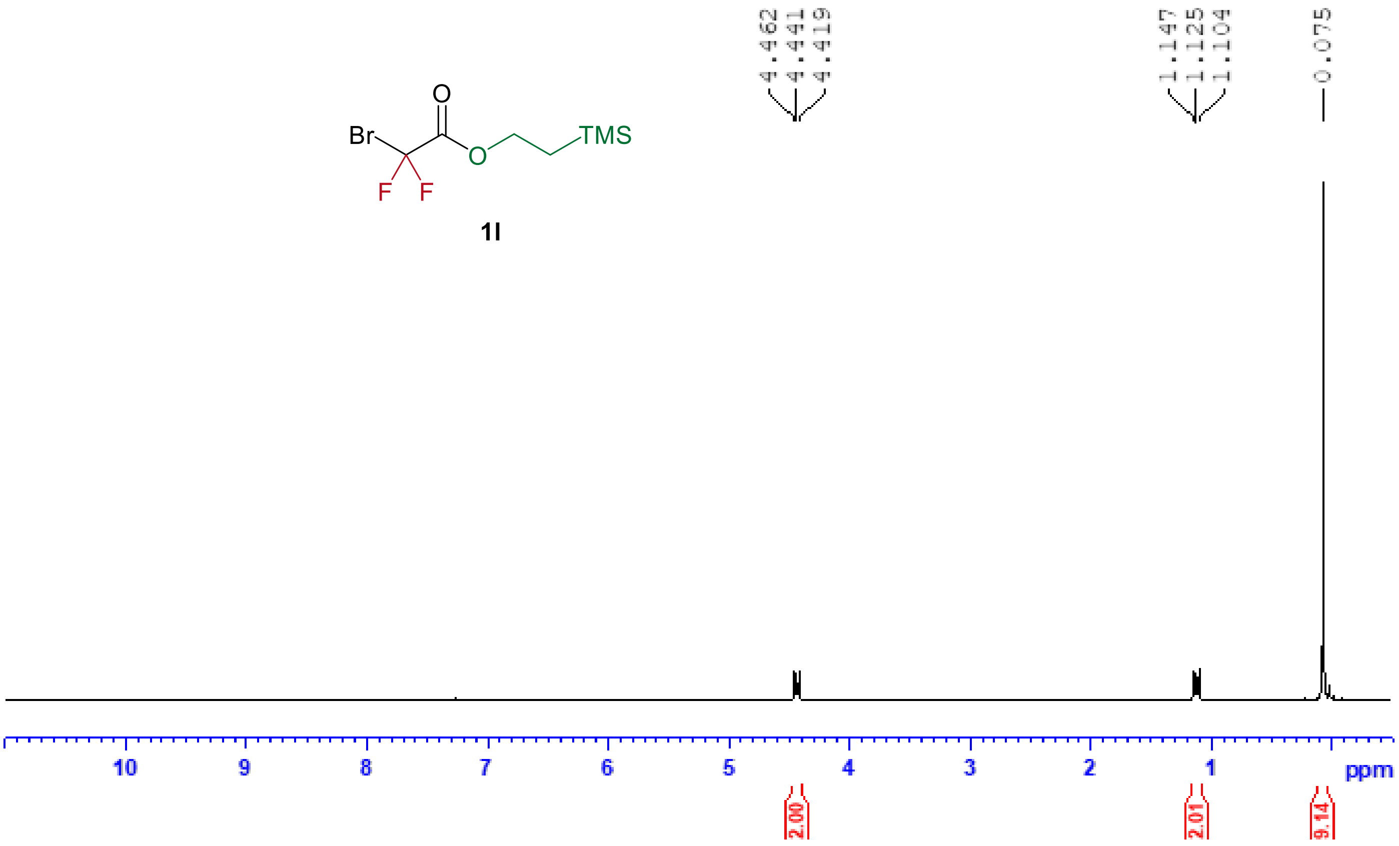

^{13}C NMR of **1l**

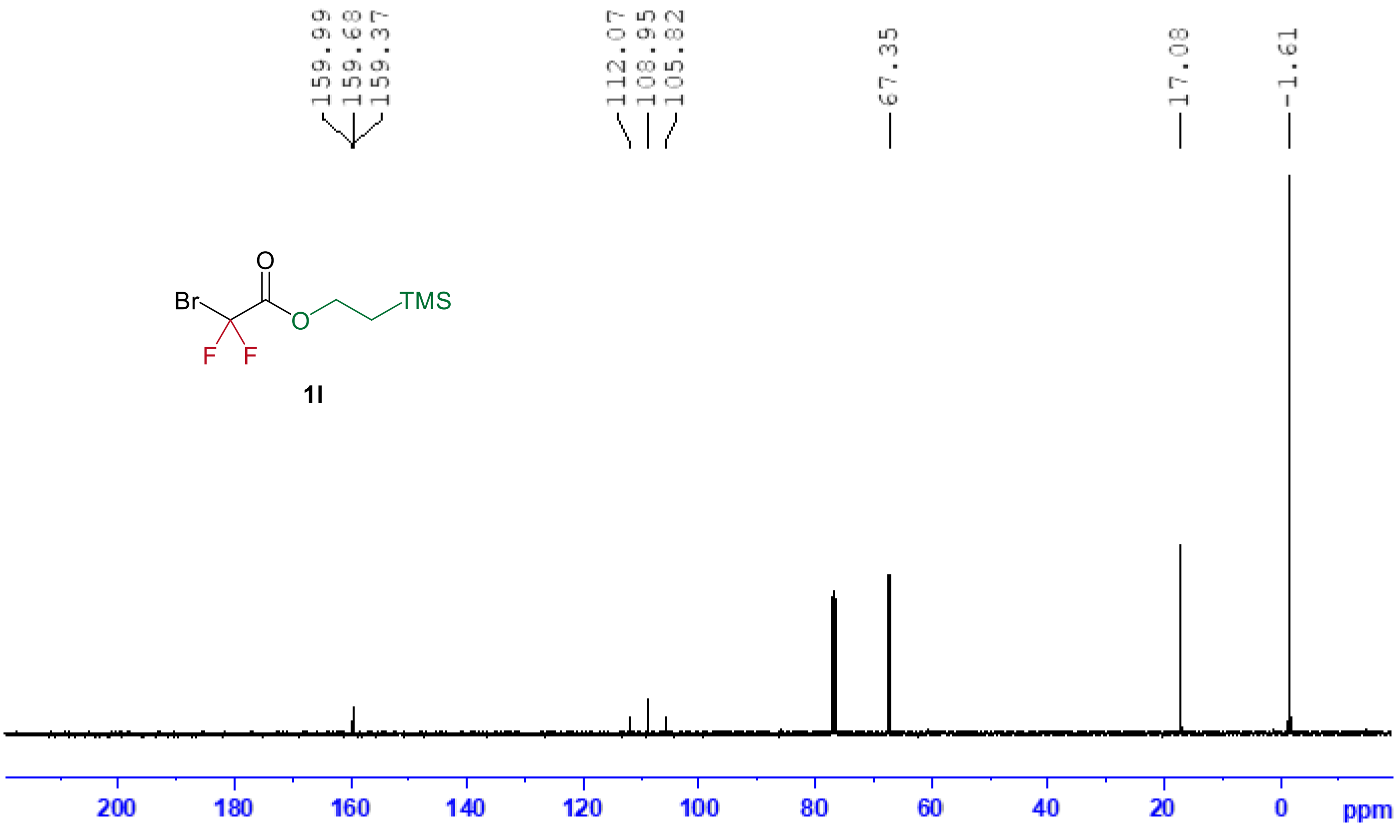

^{19}F NMR of **1l**

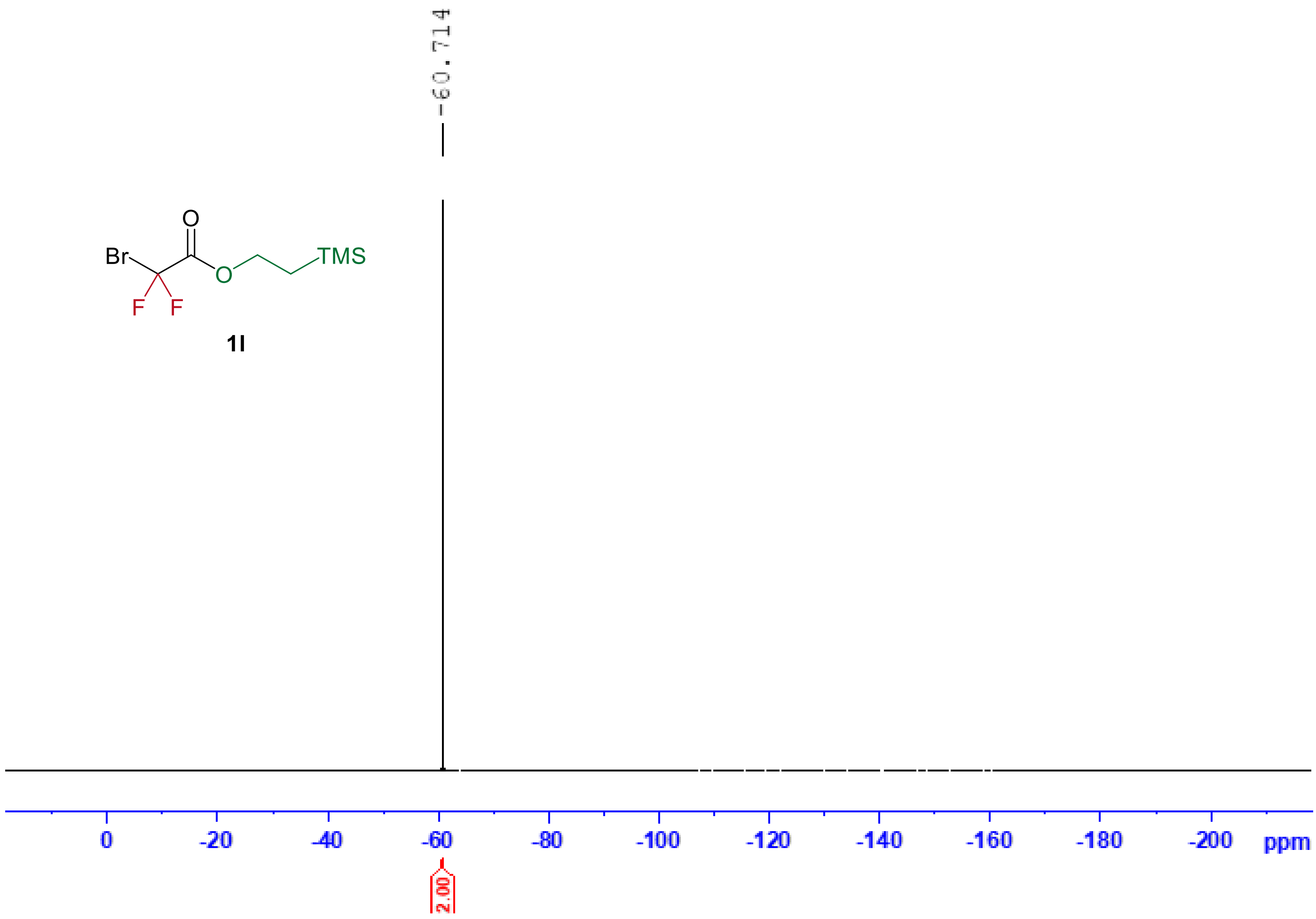

^{1}H NMR of **1m**

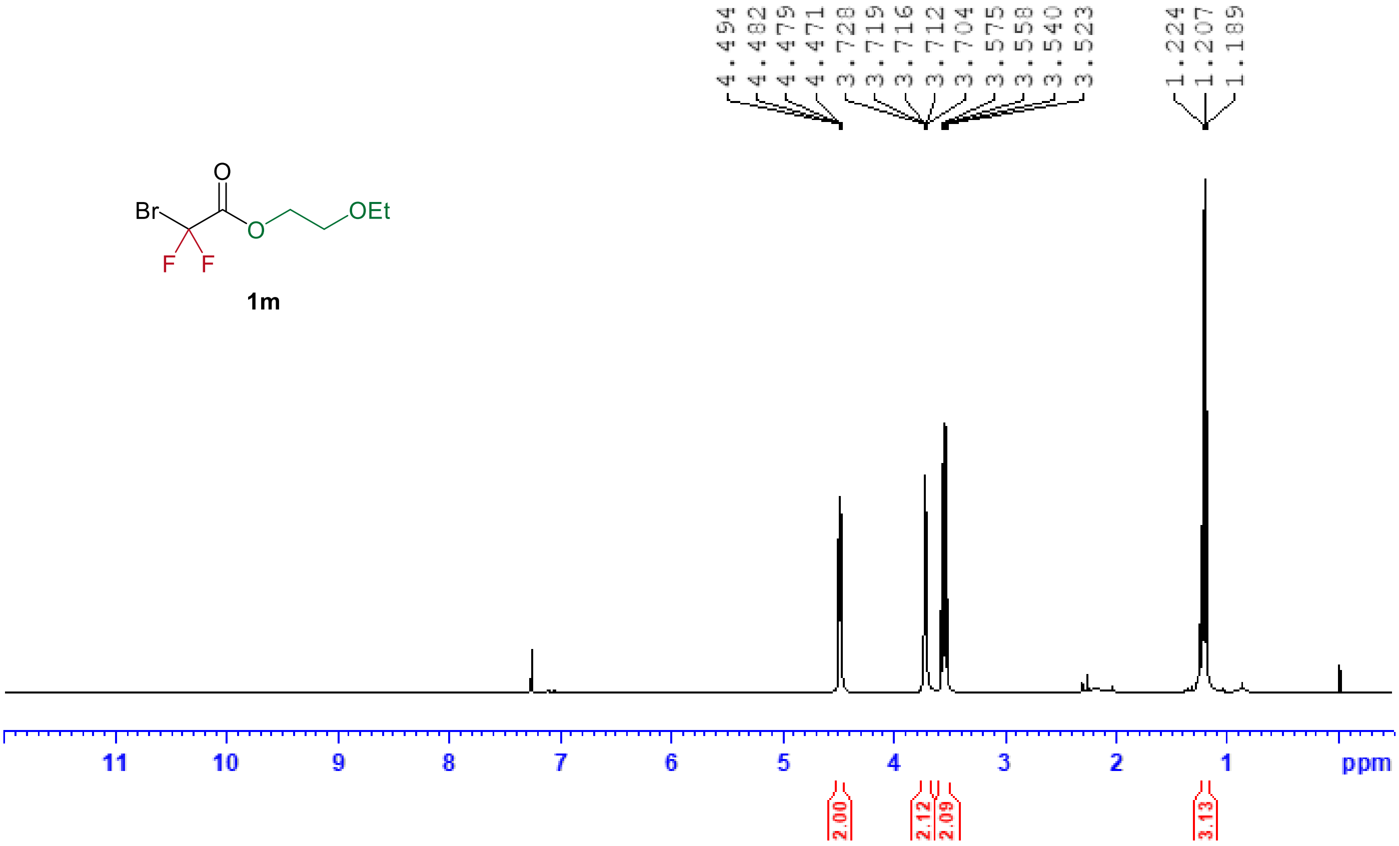

^{13}C NMR of **1m**

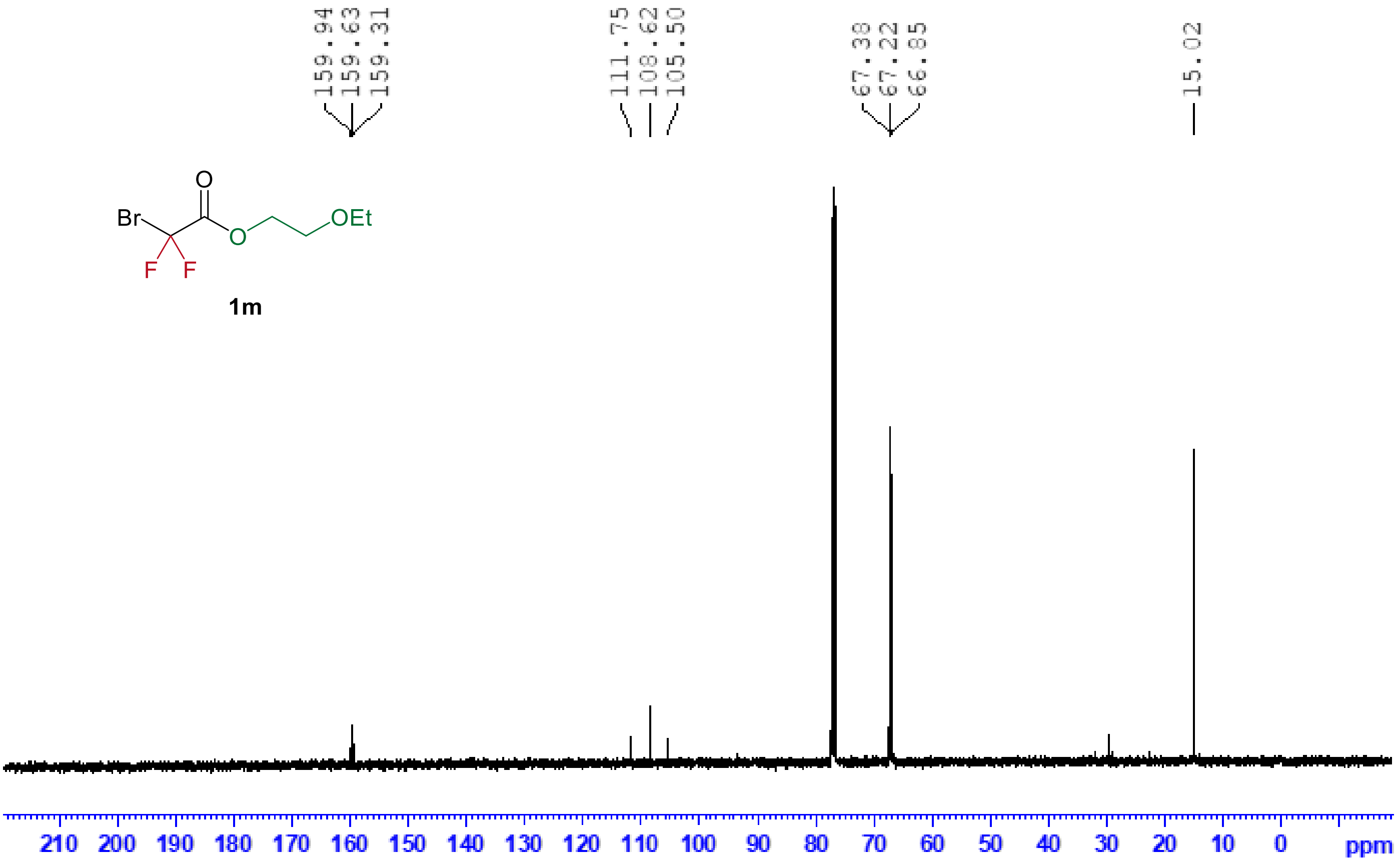

^{19}F NMR of **1m**

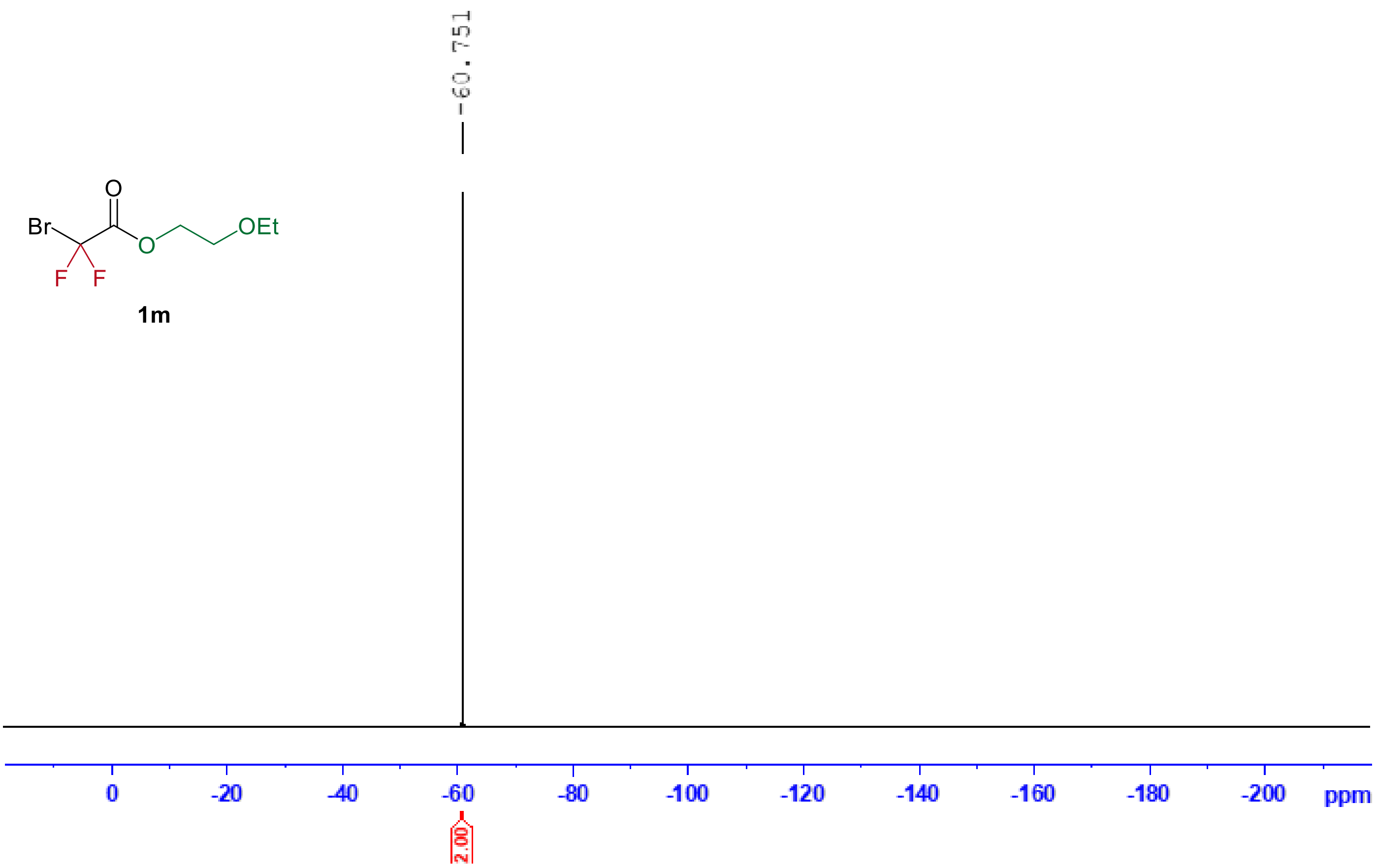

^{1}H NMR of **1n**

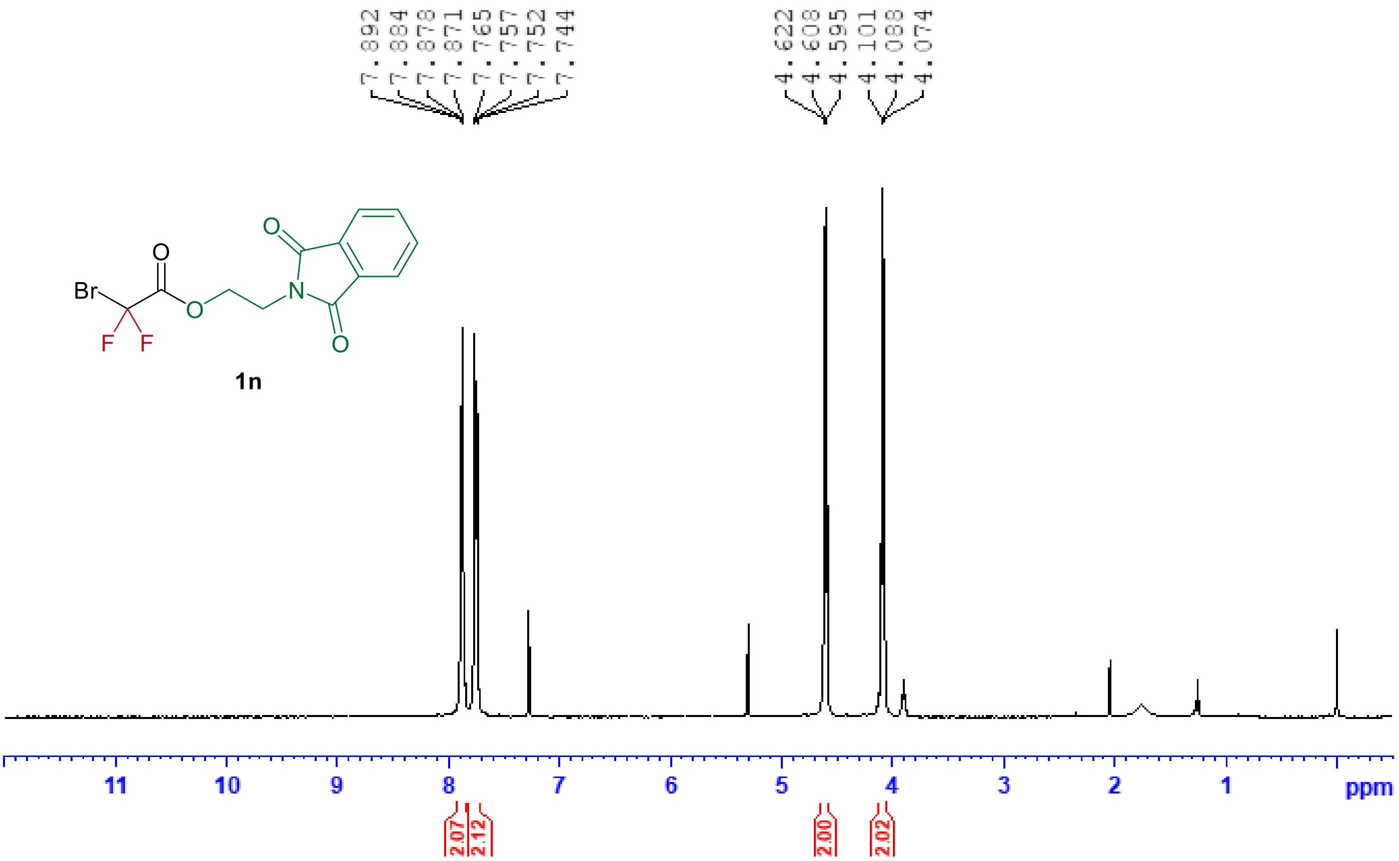

^{13}C NMR of **1n**

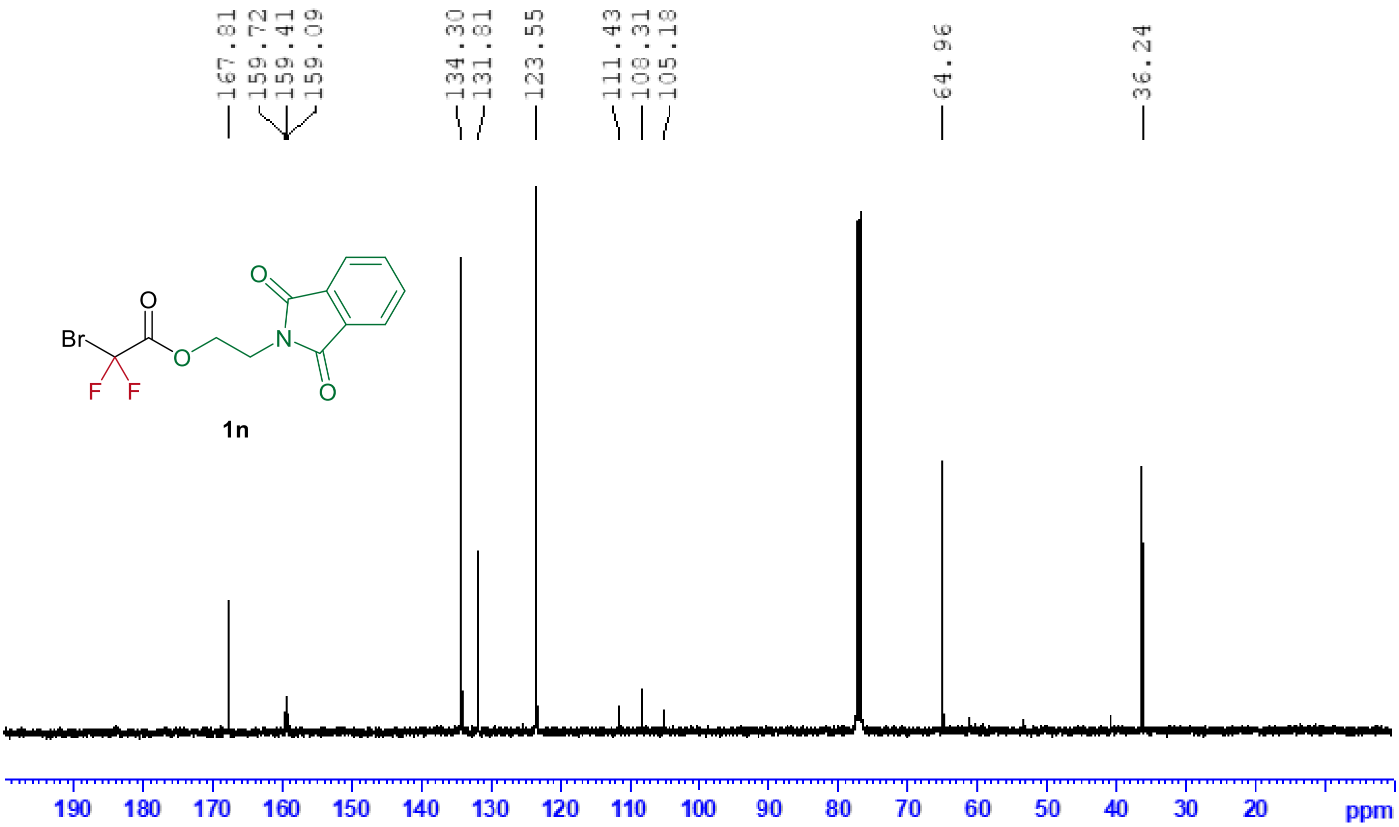

^{19}F NMR of **1n**

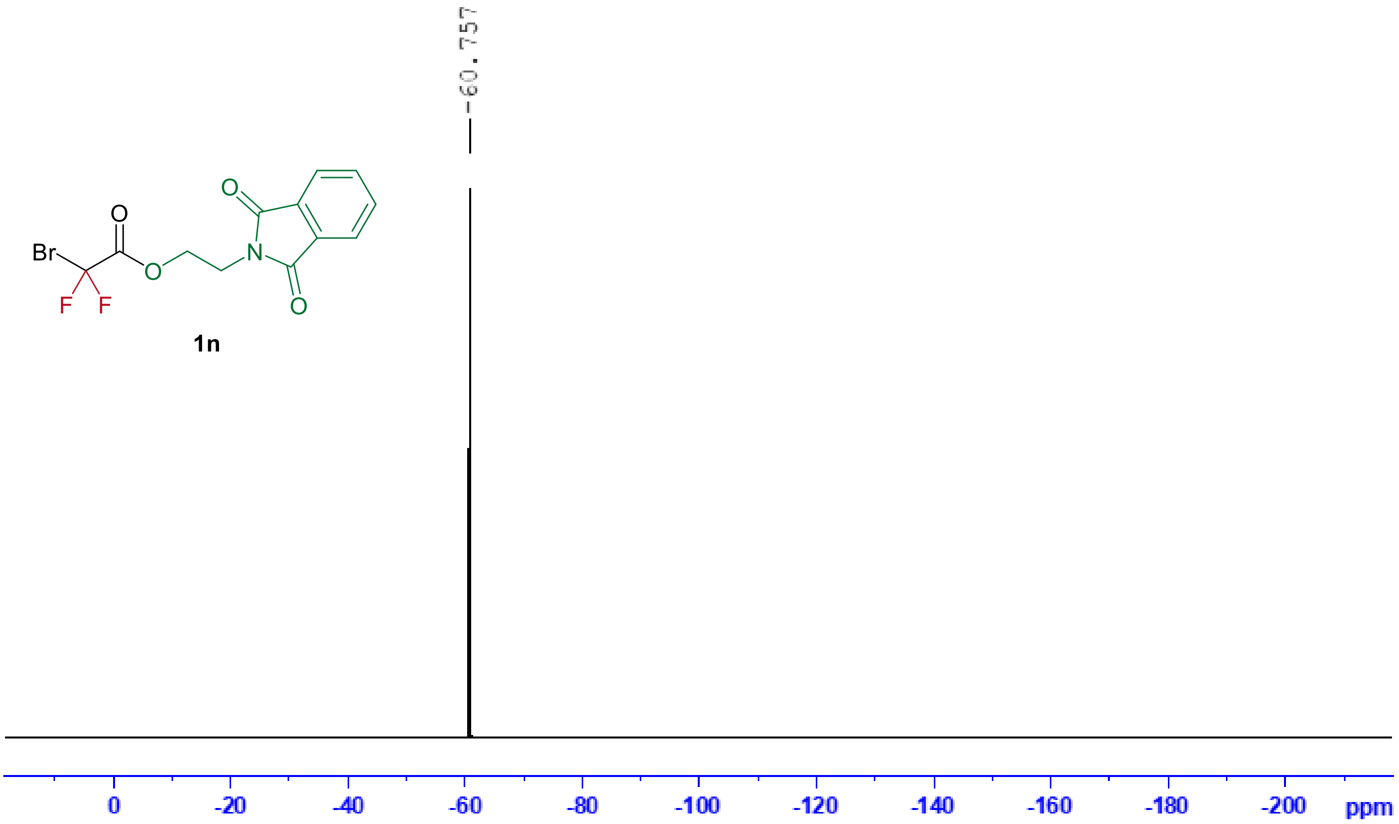

^{1}H NMR of **1o**

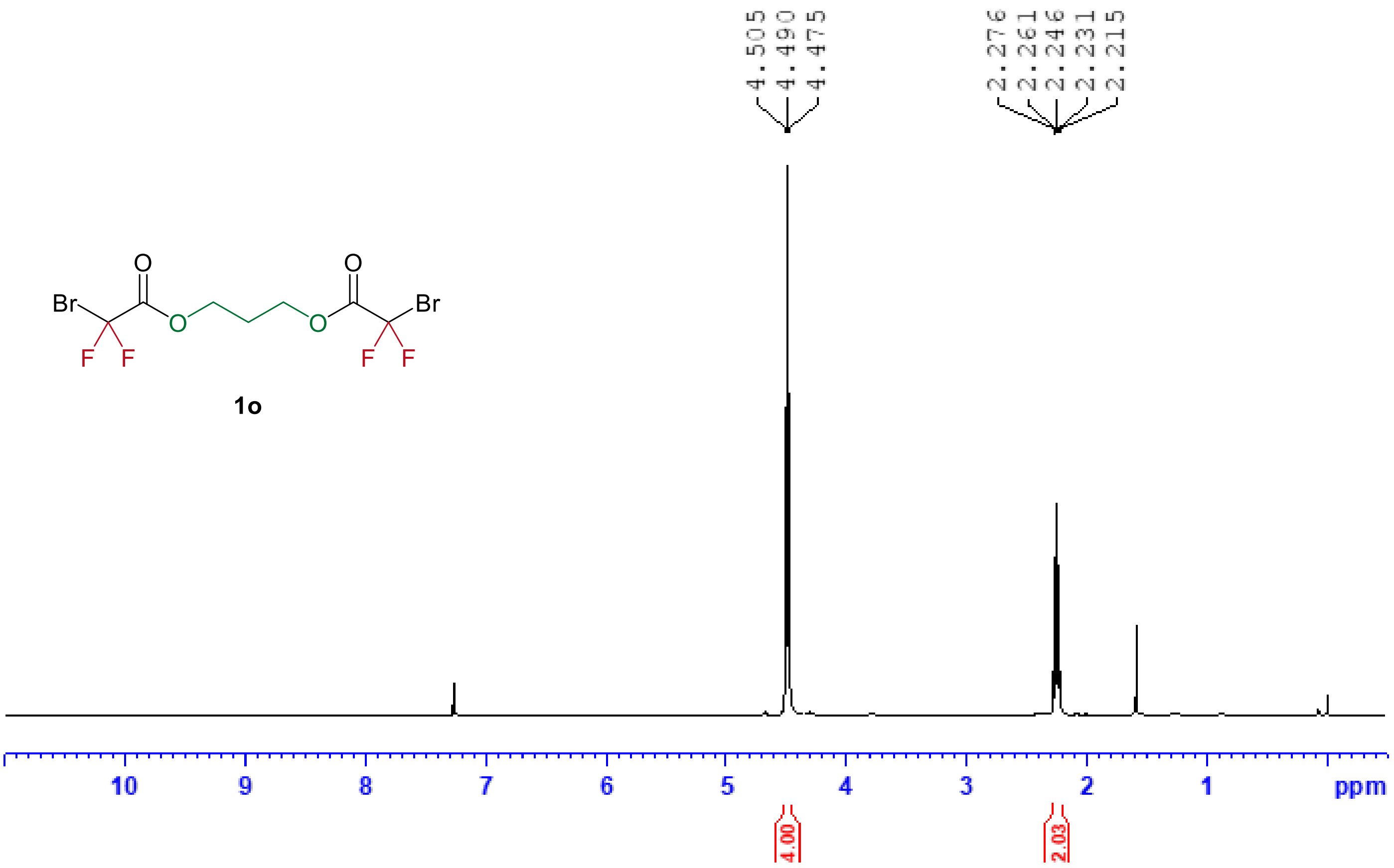

^{13}C NMR of **1o**

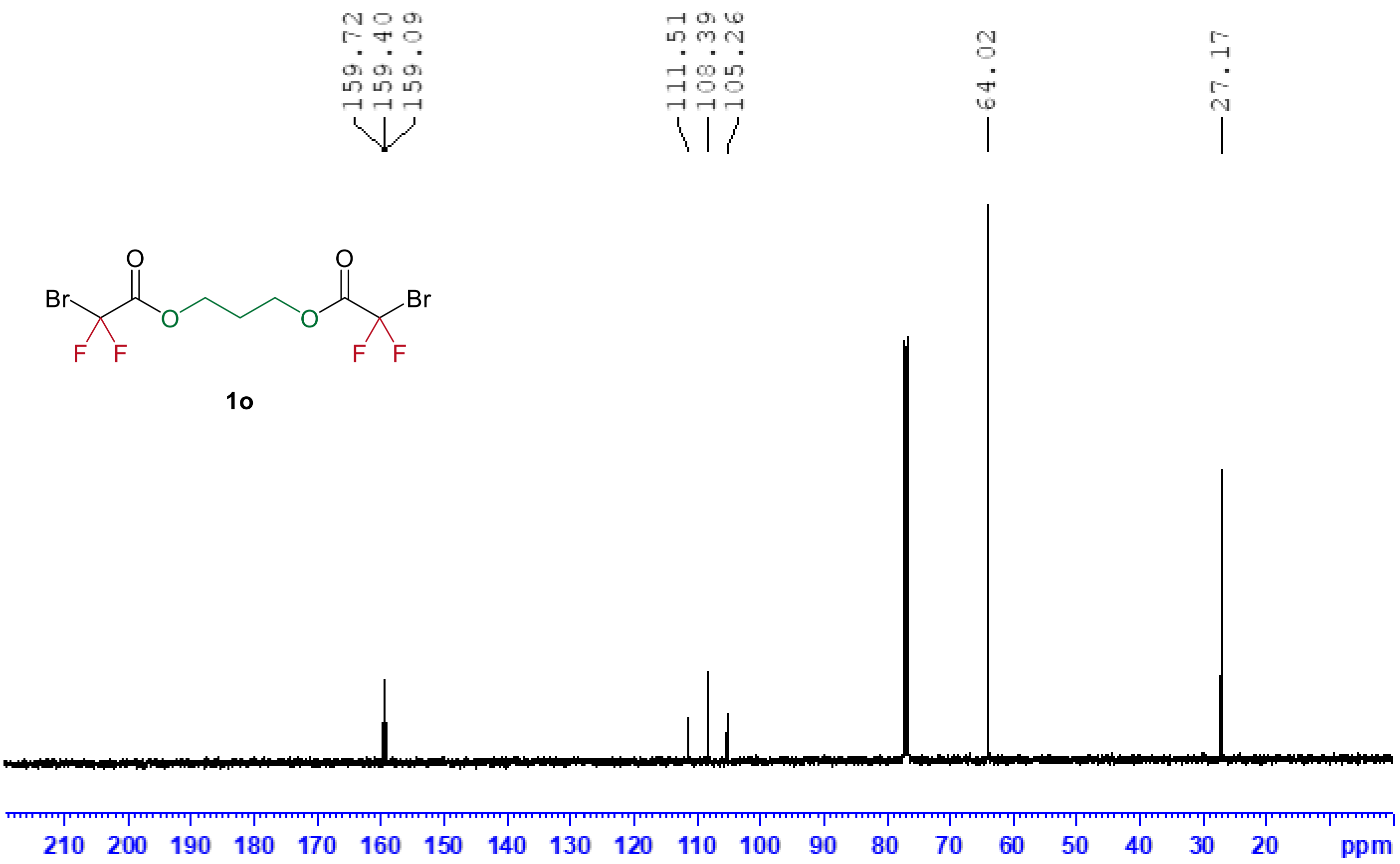

^{19}F NMR of **1o**

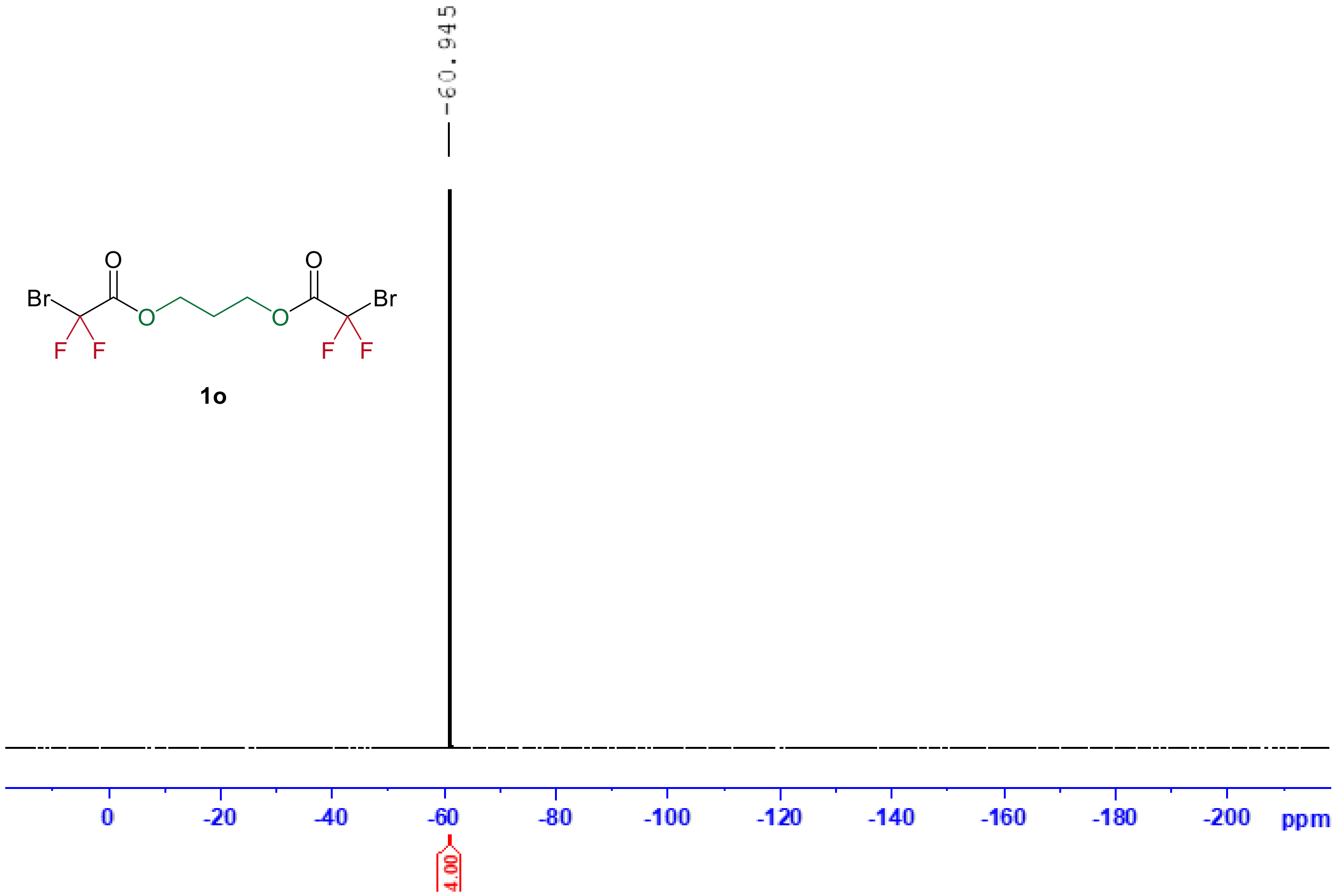

-60.945
Br
O
O
O
O
Br
F F
F F
1o
0
-20
-40
-60
-80
-100
-120
-140
-160
-180
-200
ppm
4.00

^{1}H NMR of **1p**

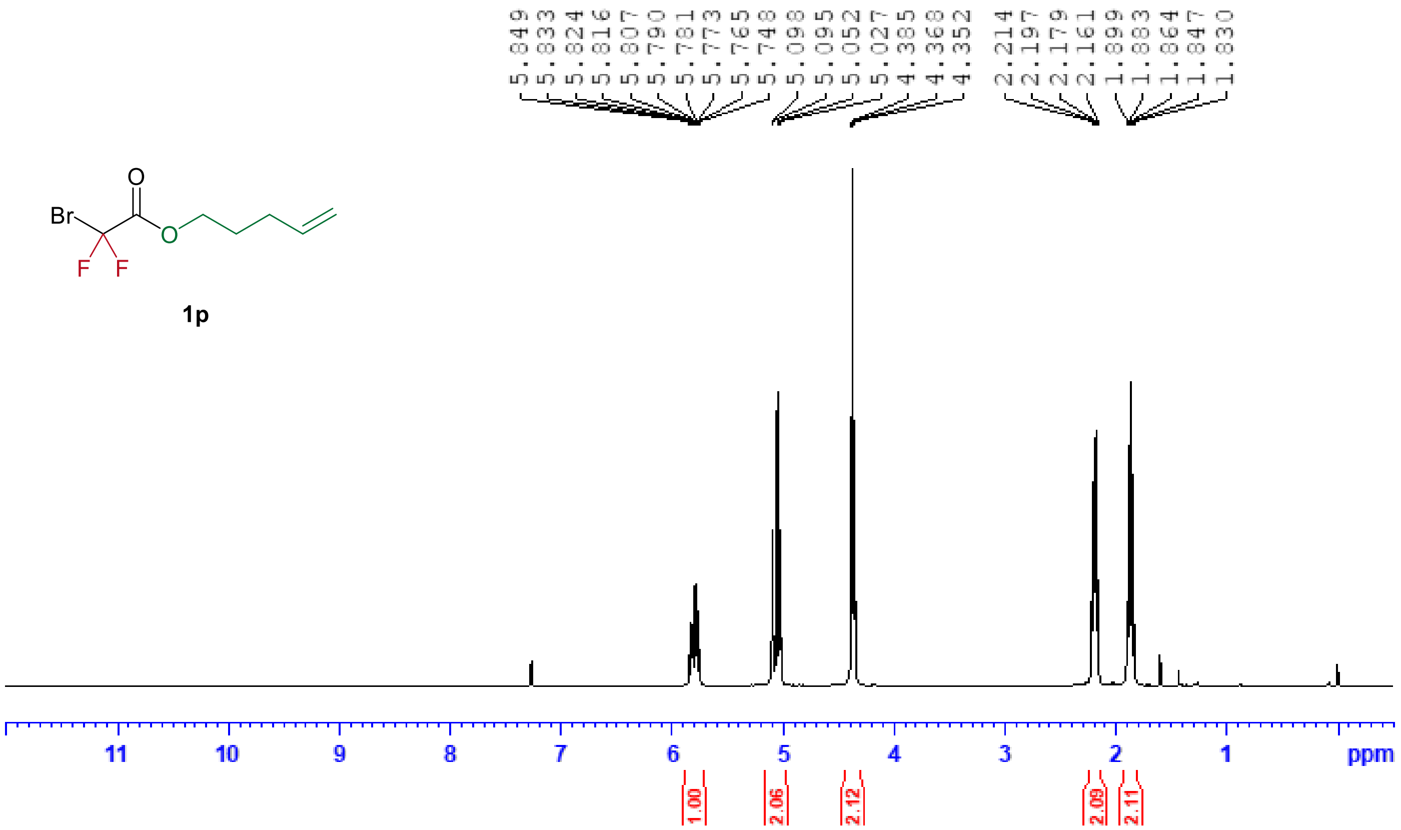

^{13}C NMR of **1p**

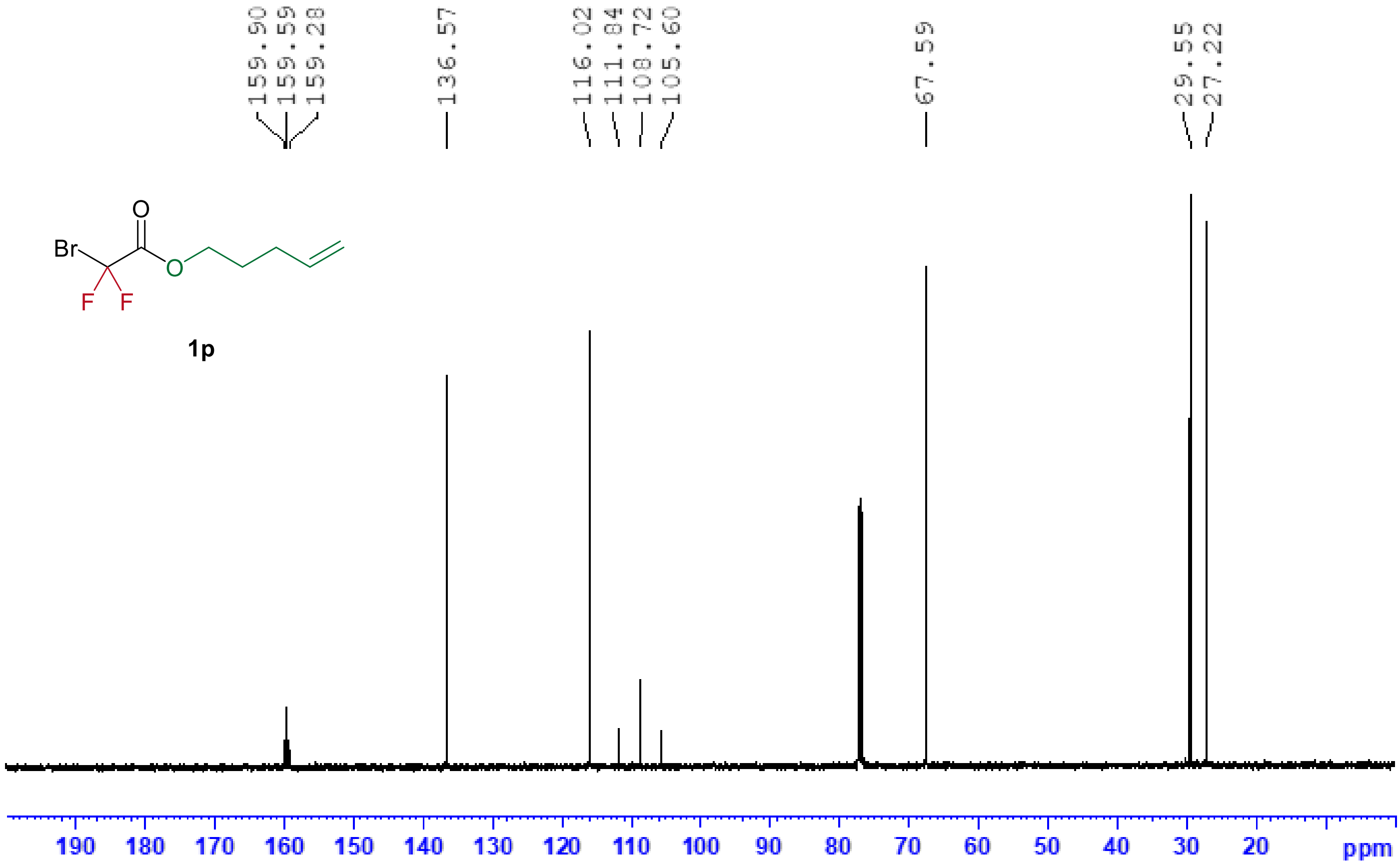

^{19}F NMR of **1p**

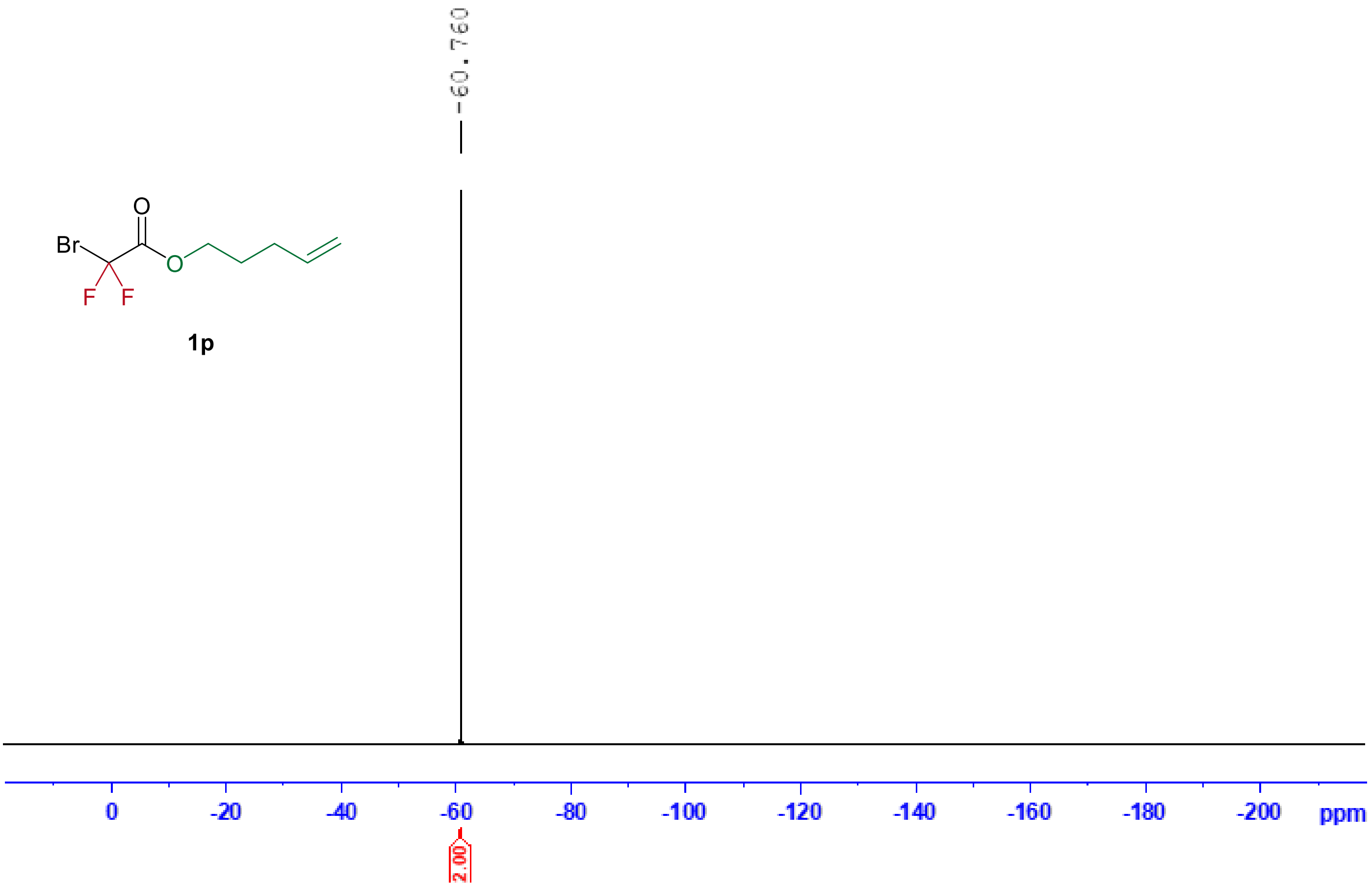

^{1}H NMR of **1q**

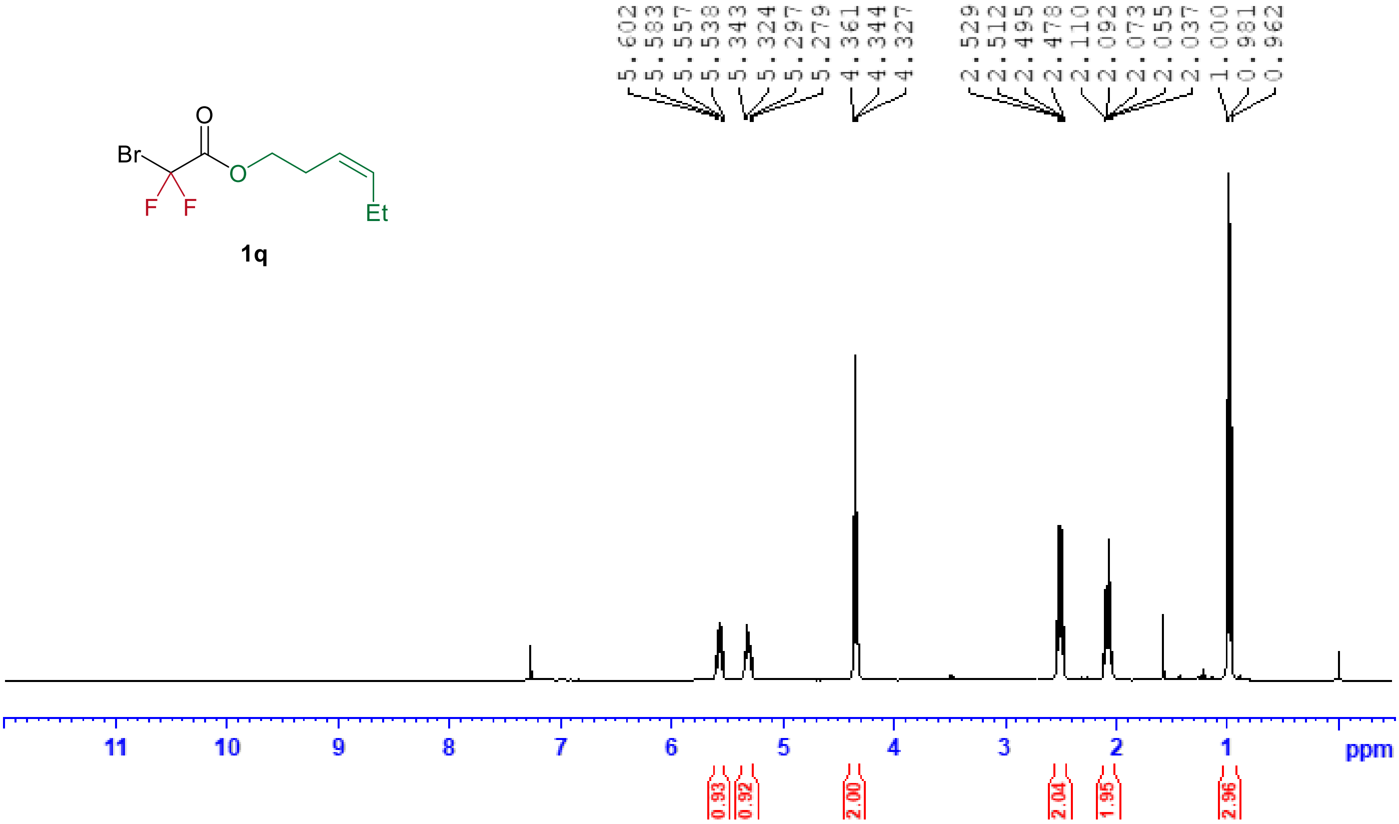

^{13}C NMR of **1q**

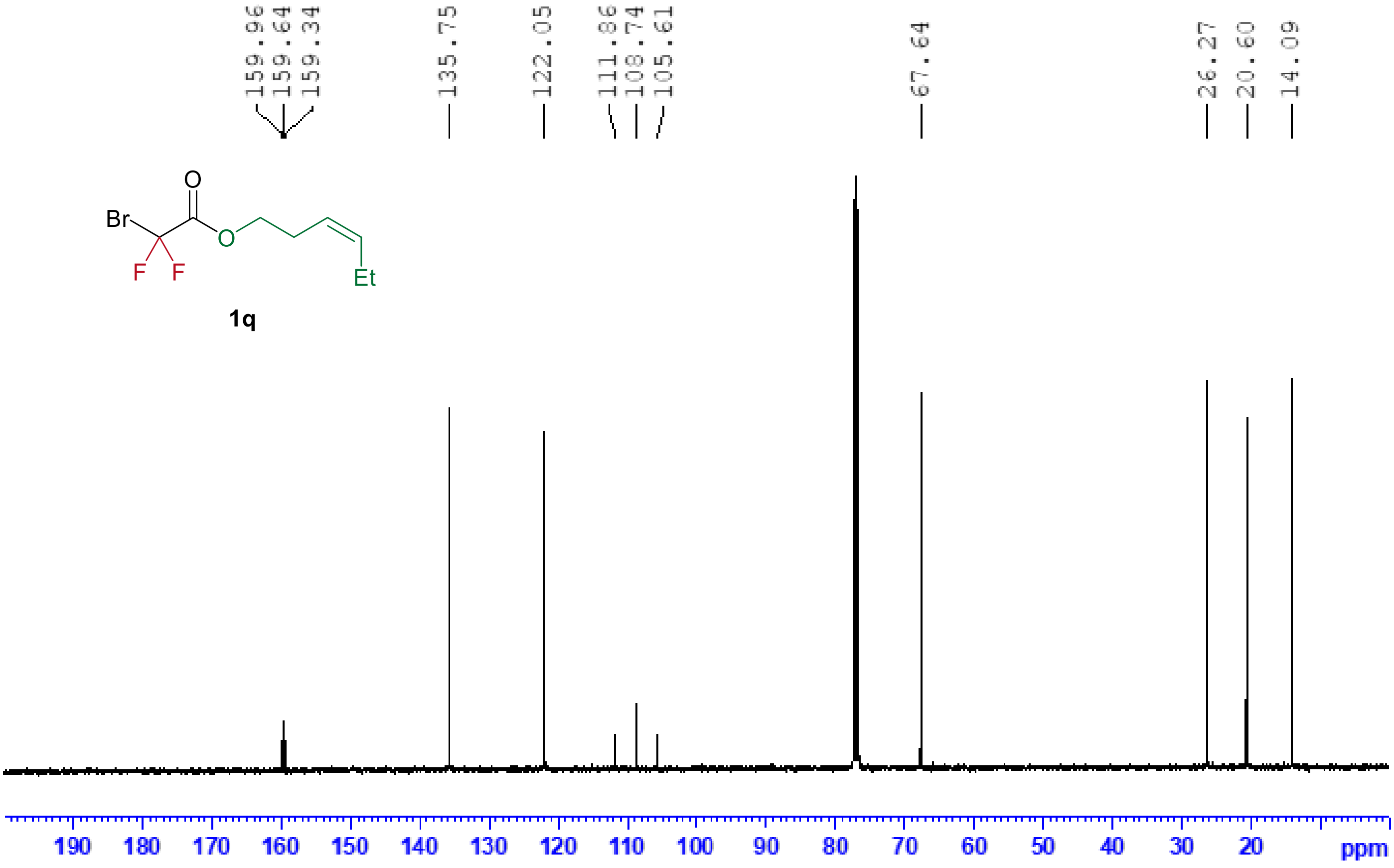

^{19}F NMR of **1q**

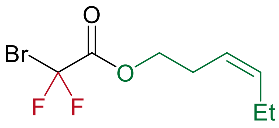

1q

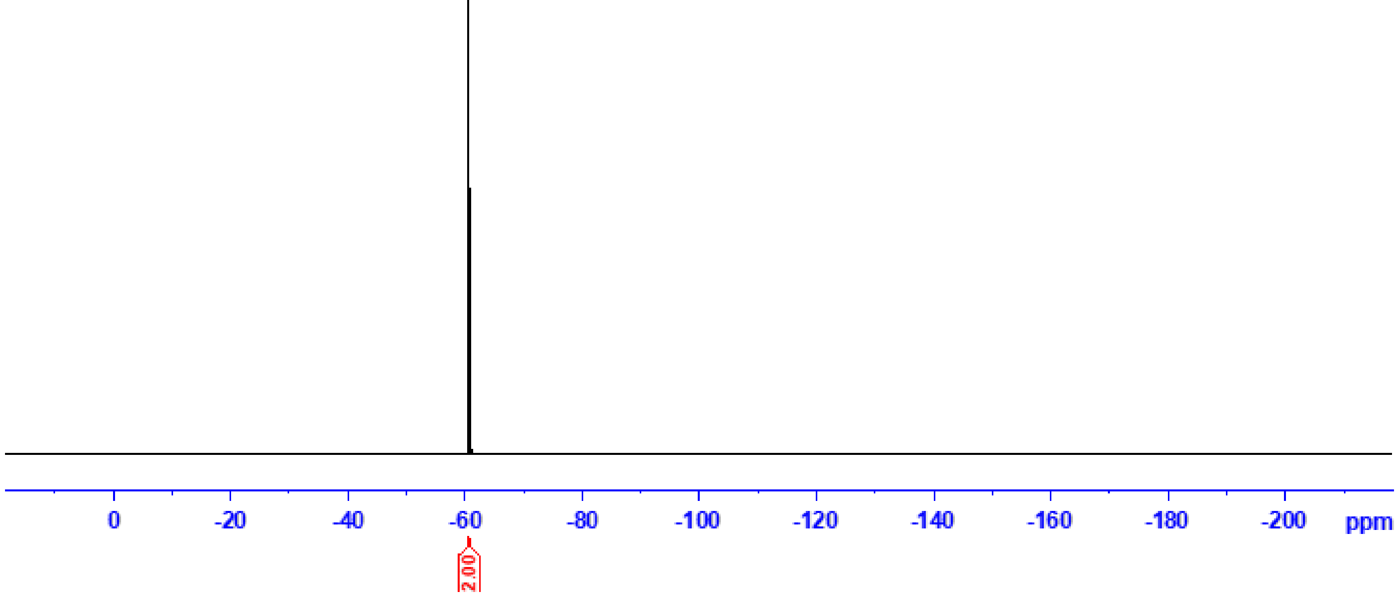

^{1}H NMR of **1r**

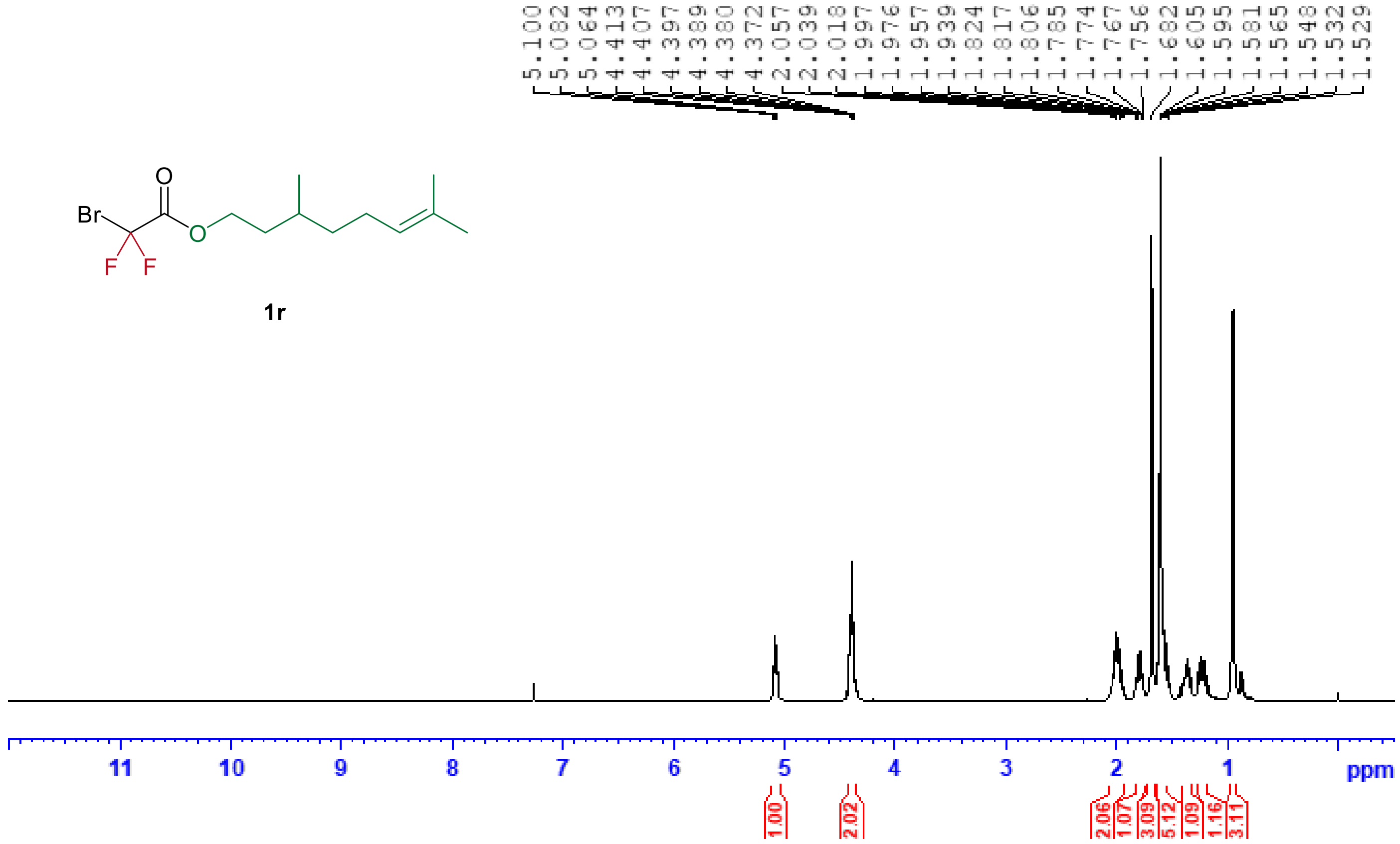

^{13}C NMR of **1r**

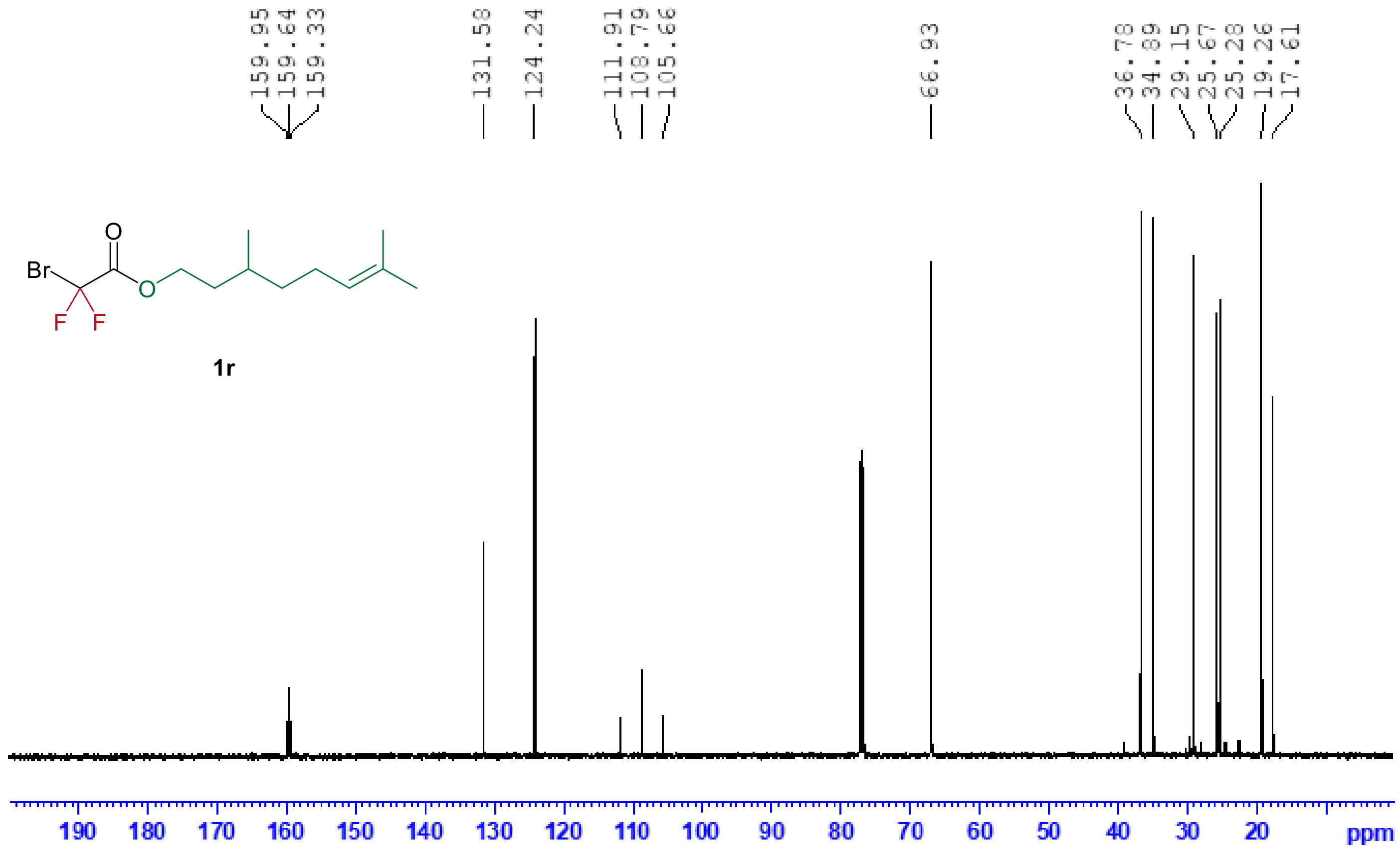

^{19}F NMR of **1r**

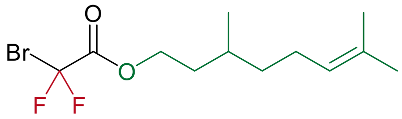

1r

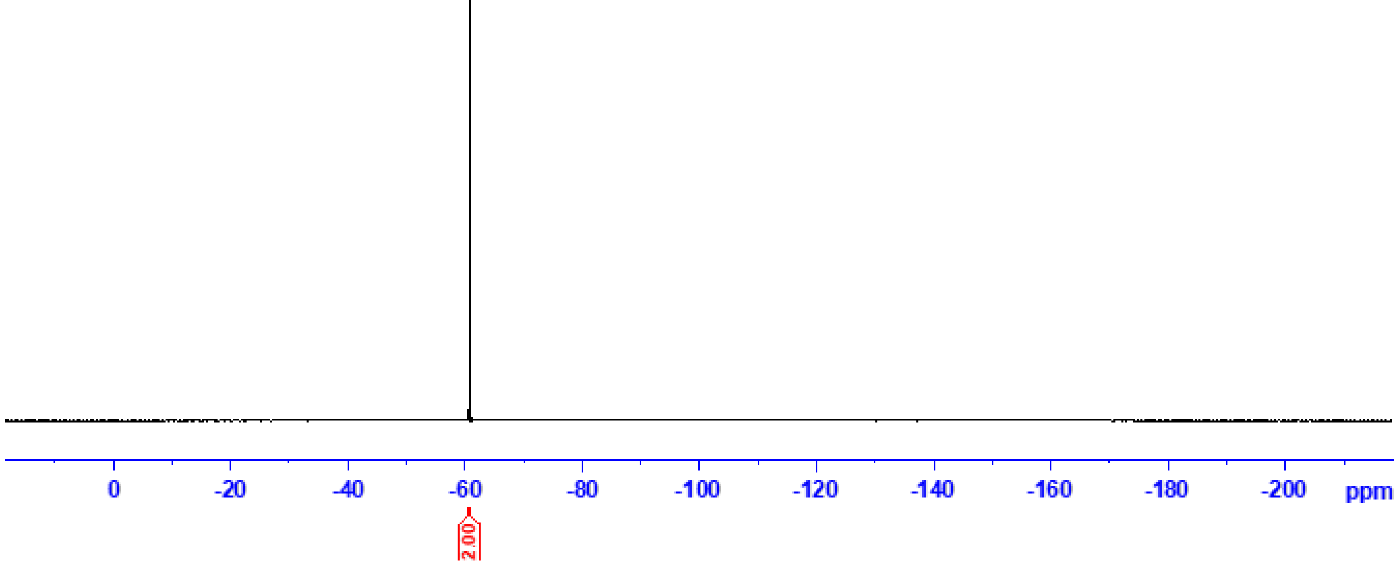

^{1}H NMR of **1s**

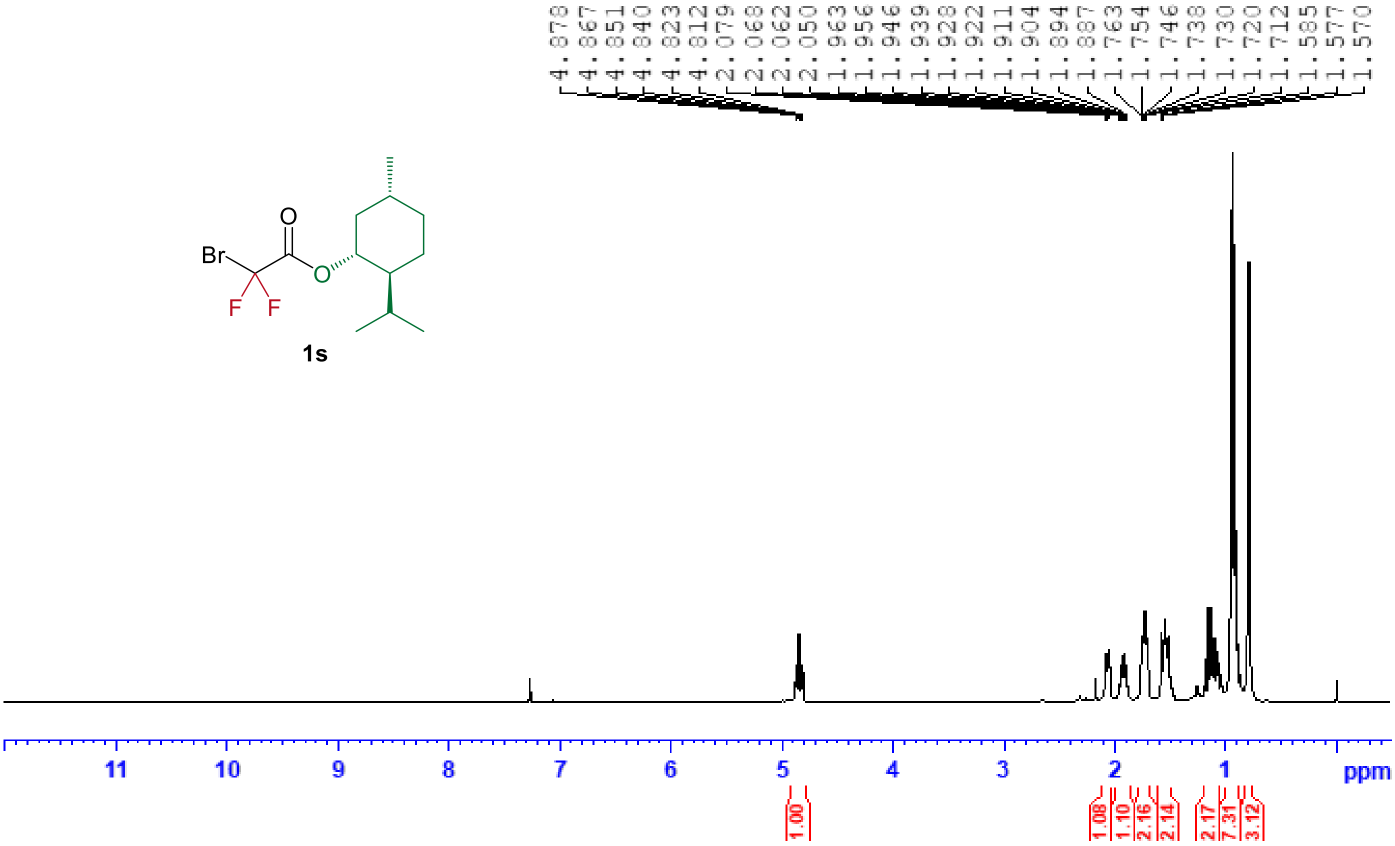

^{13}C NMR of **1s**

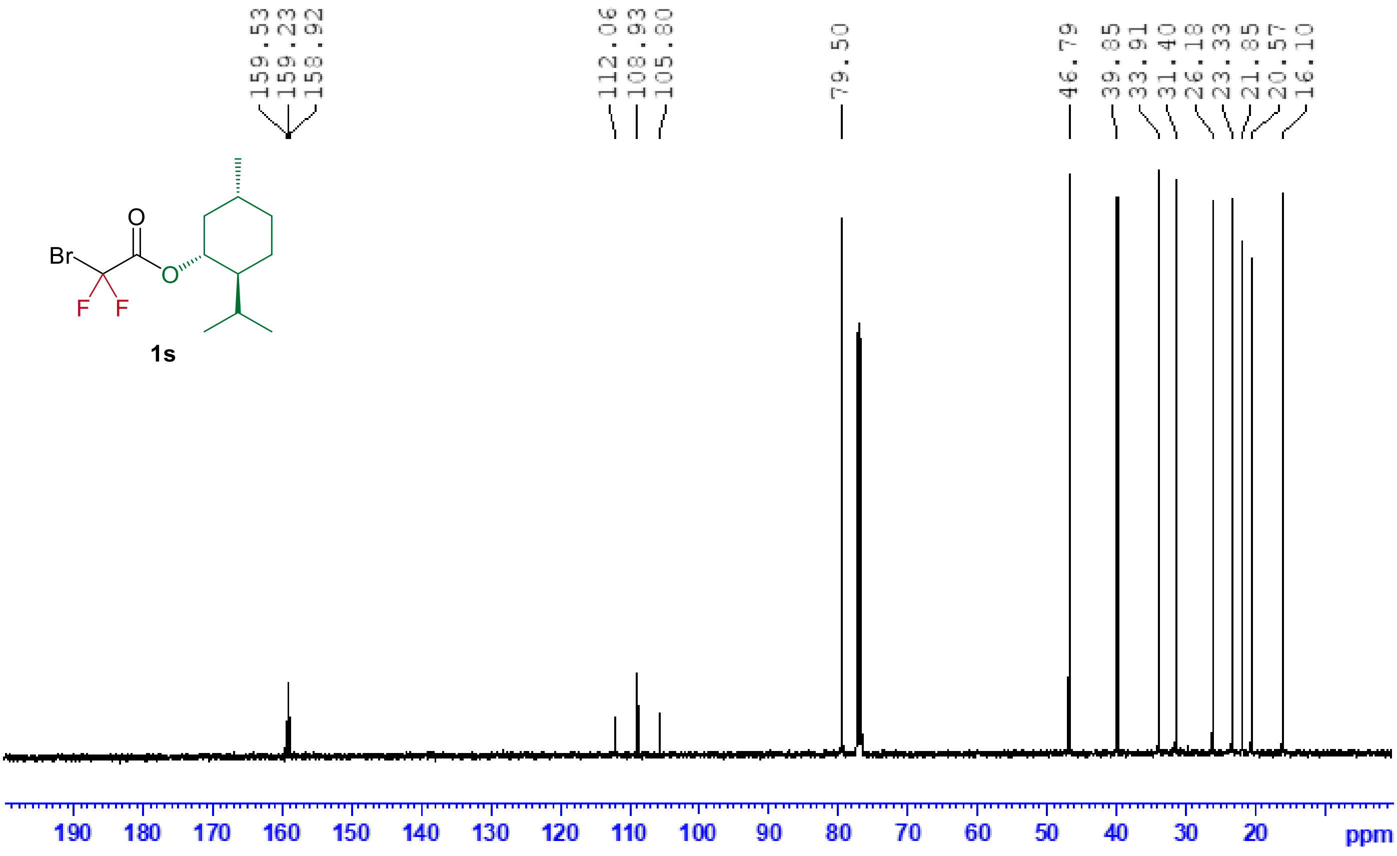

^{19}F NMR of **1s**

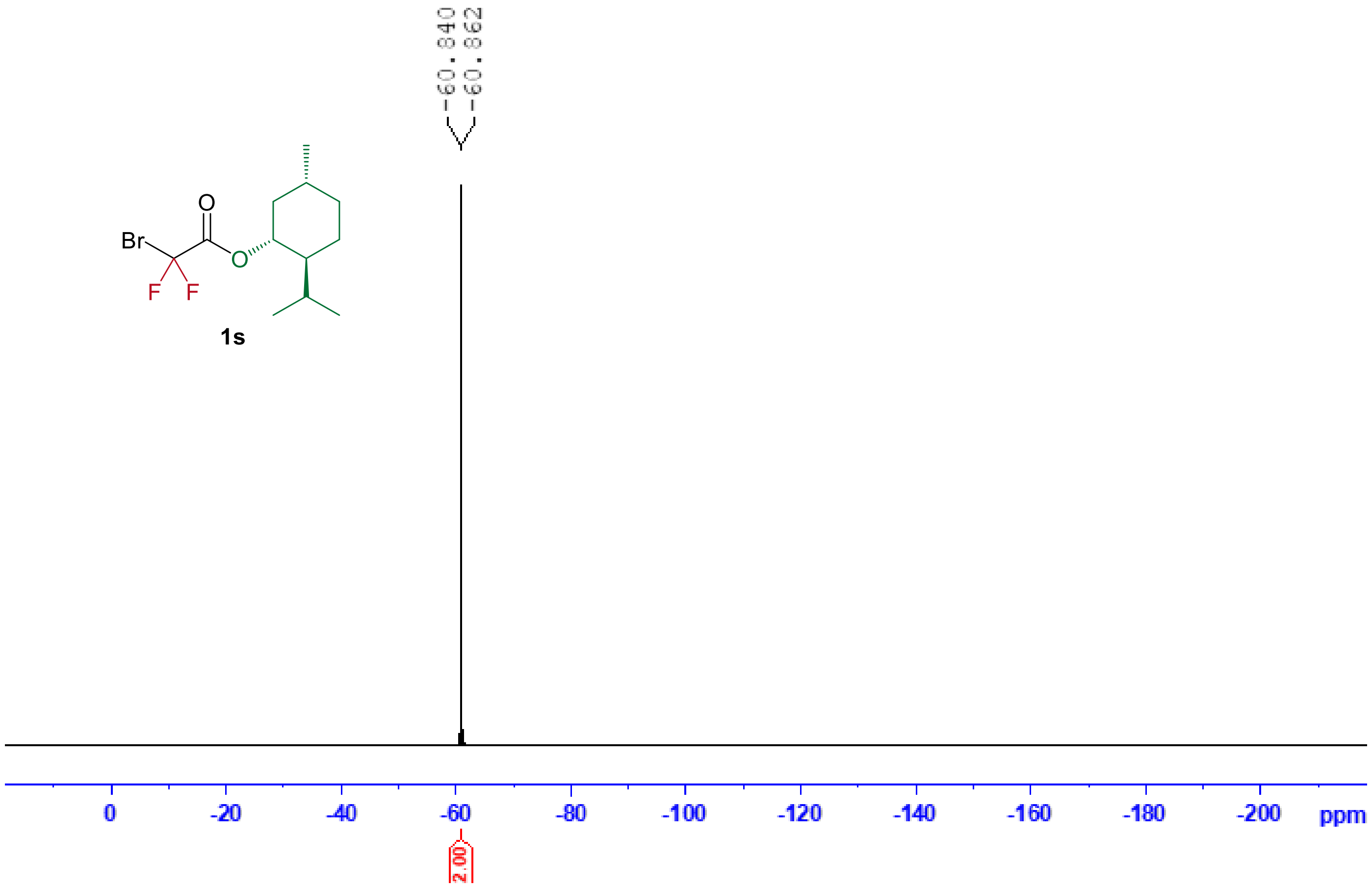

-60.840
-60.862
Br
O
O
F
F
1s
0
-20
-40
-60
-80
-100
-120
-140
-160
-180
-200
ppm
2.00

^{1}H NMR of **1t**

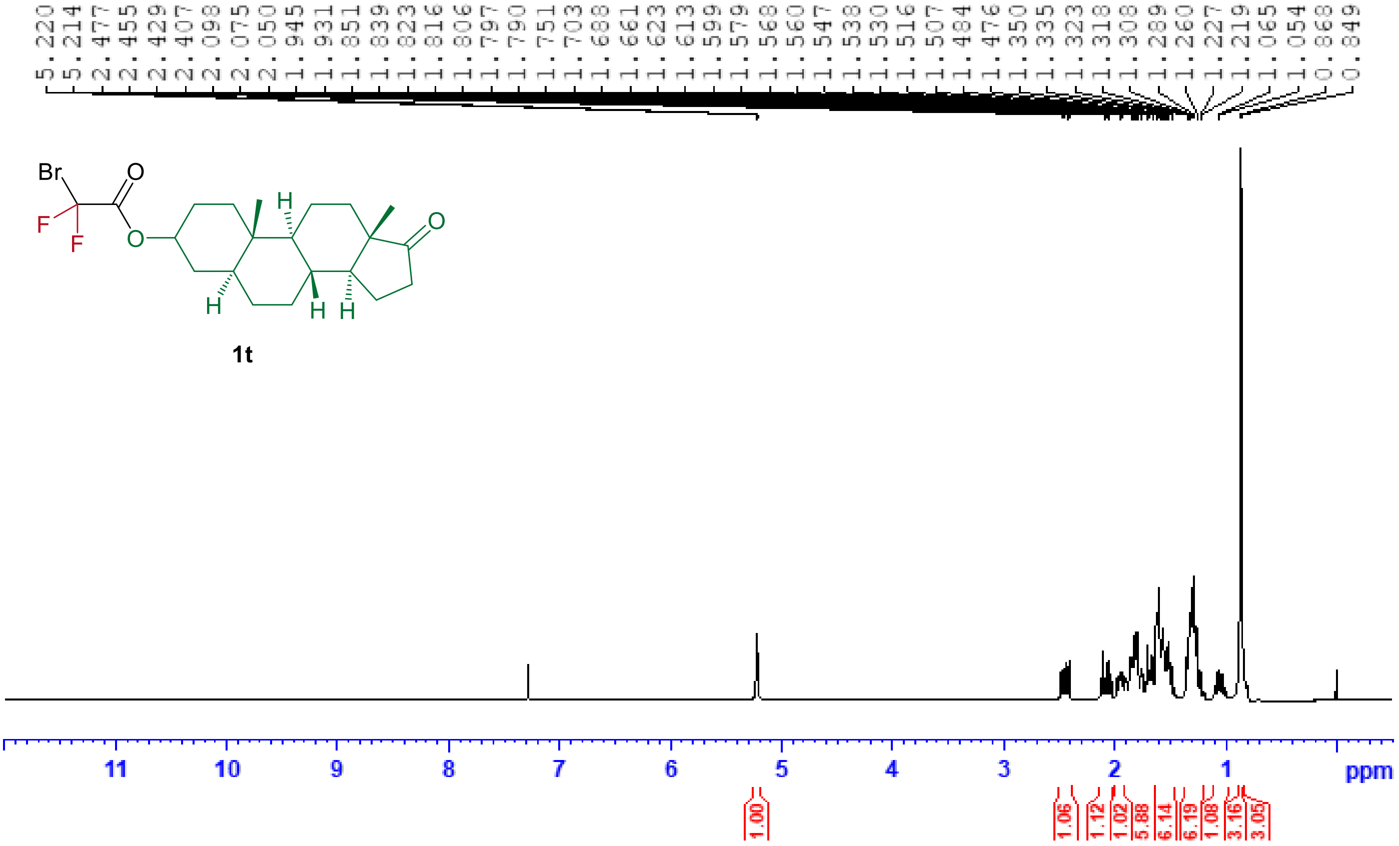

^{13}C NMR of **1t**

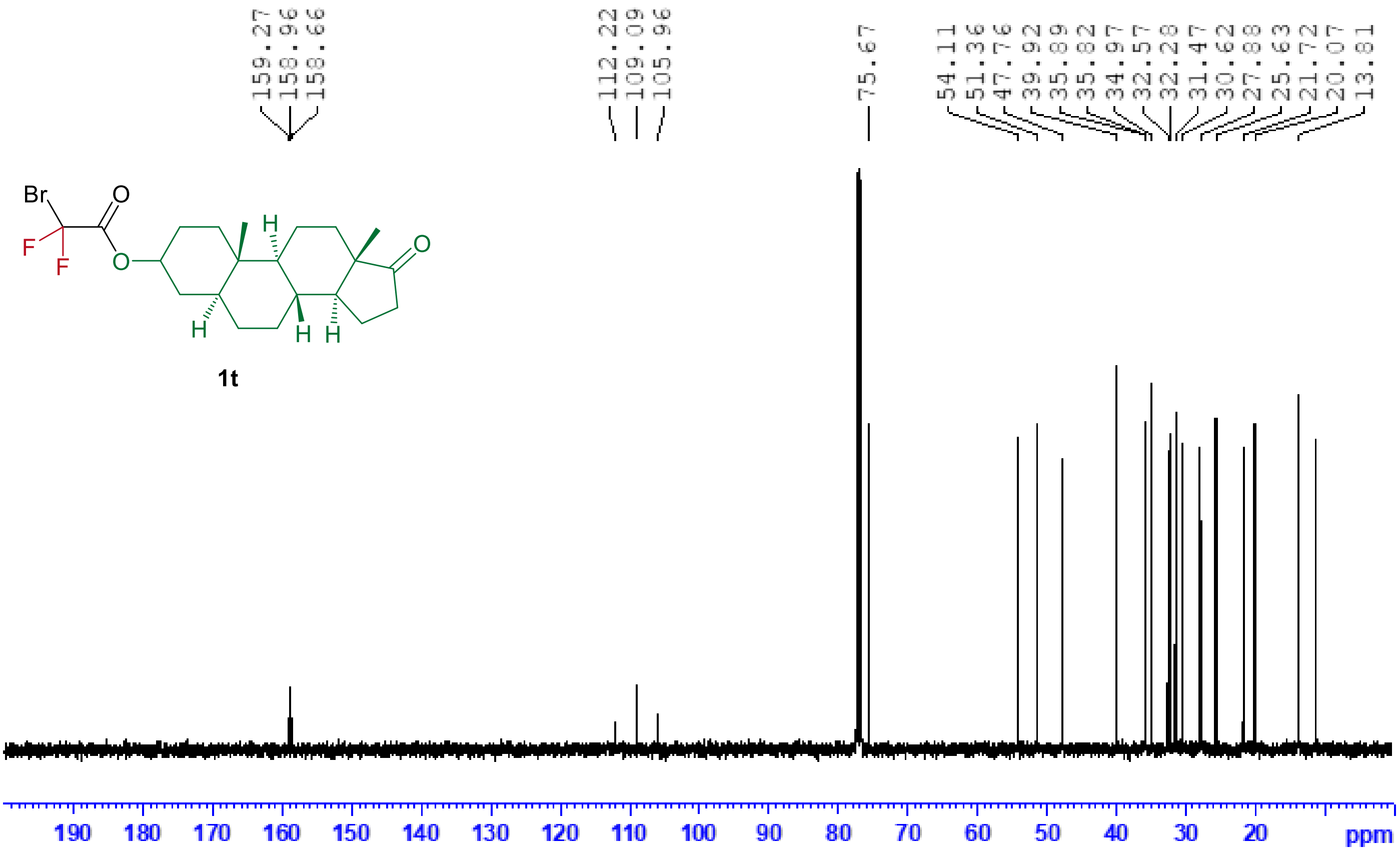

^{19}F NMR of **1t**

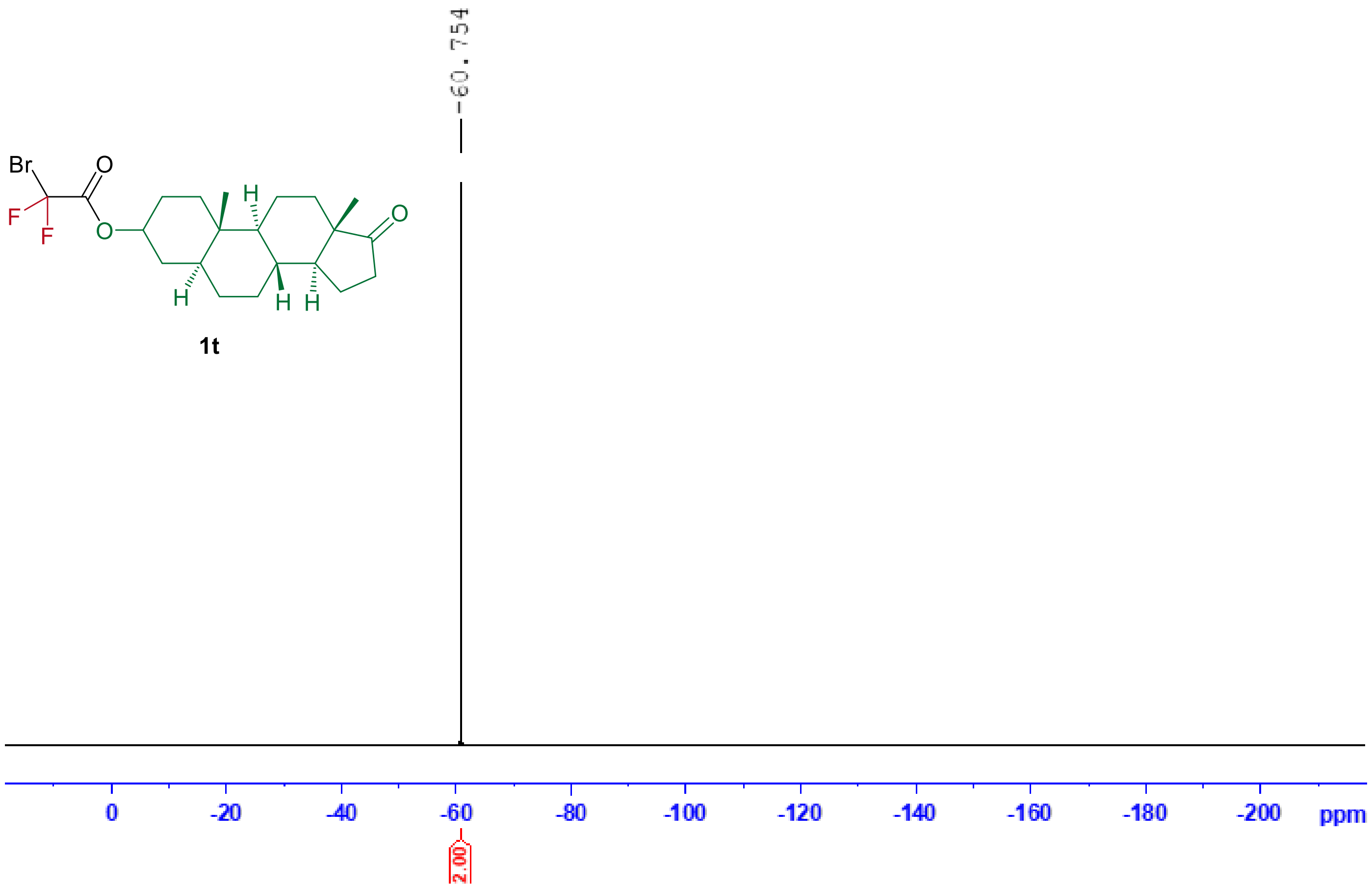

^{1}H NMR of **1u**

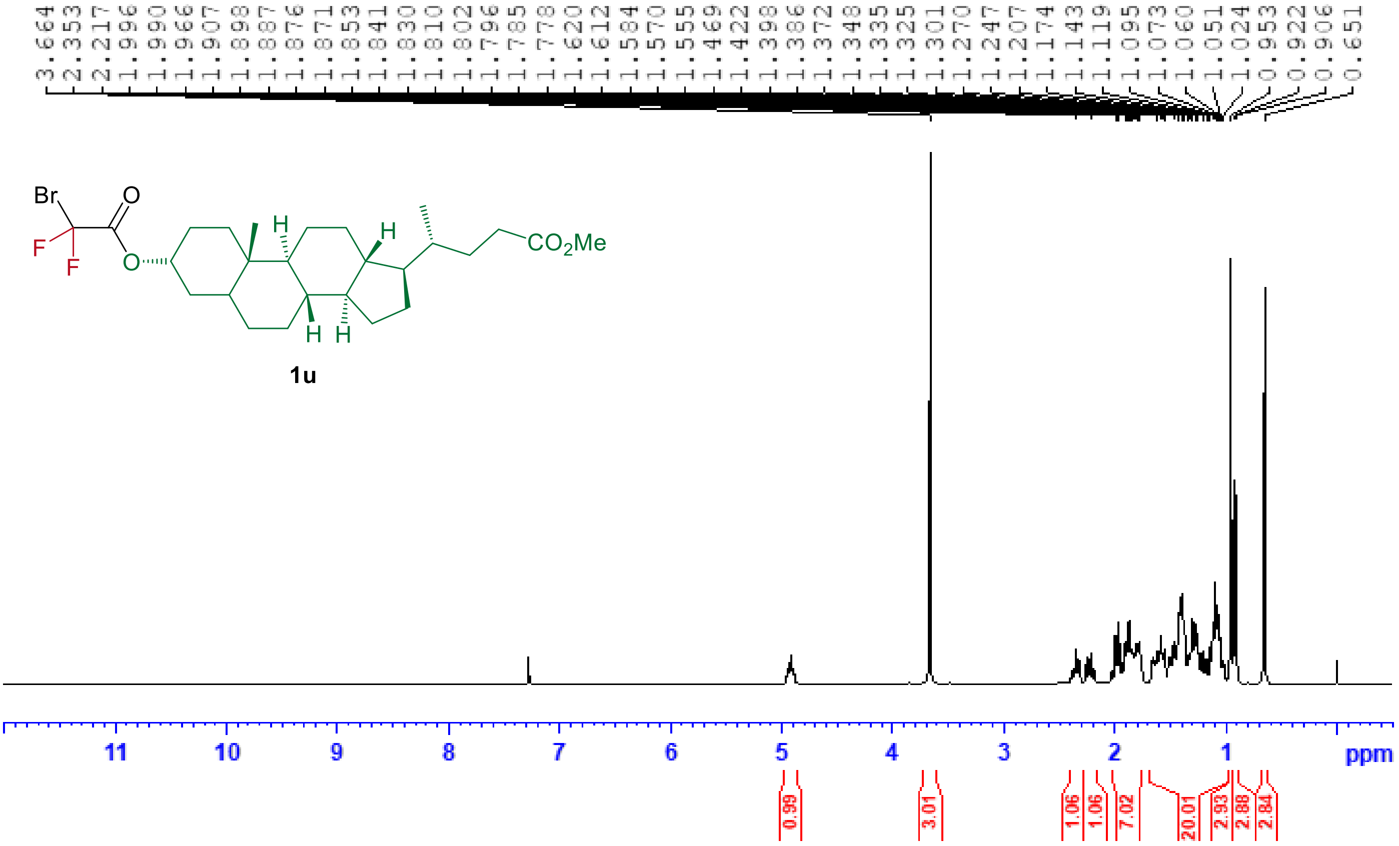

^{13}C NMR of **1u**

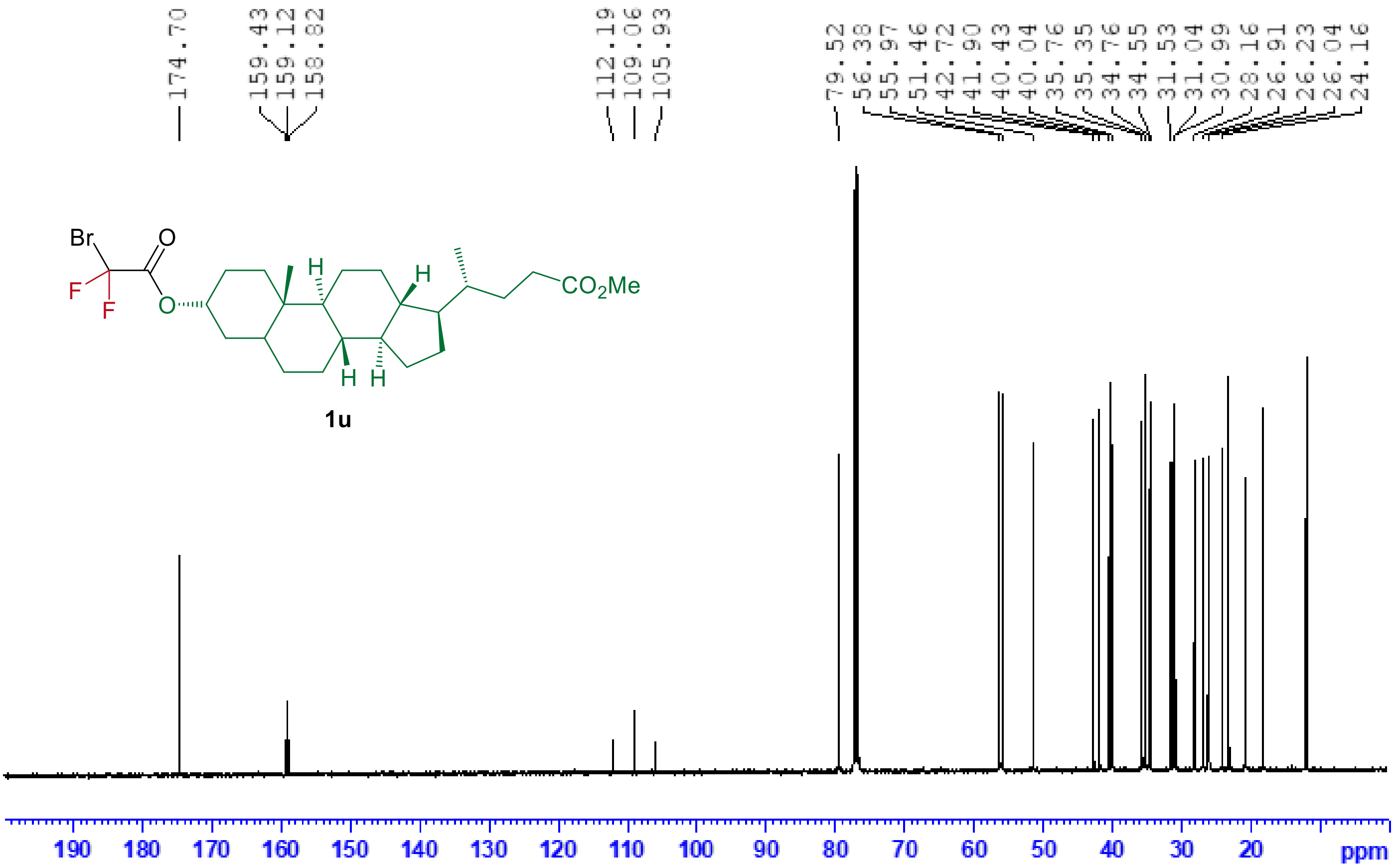

^{19}F NMR of **1u**

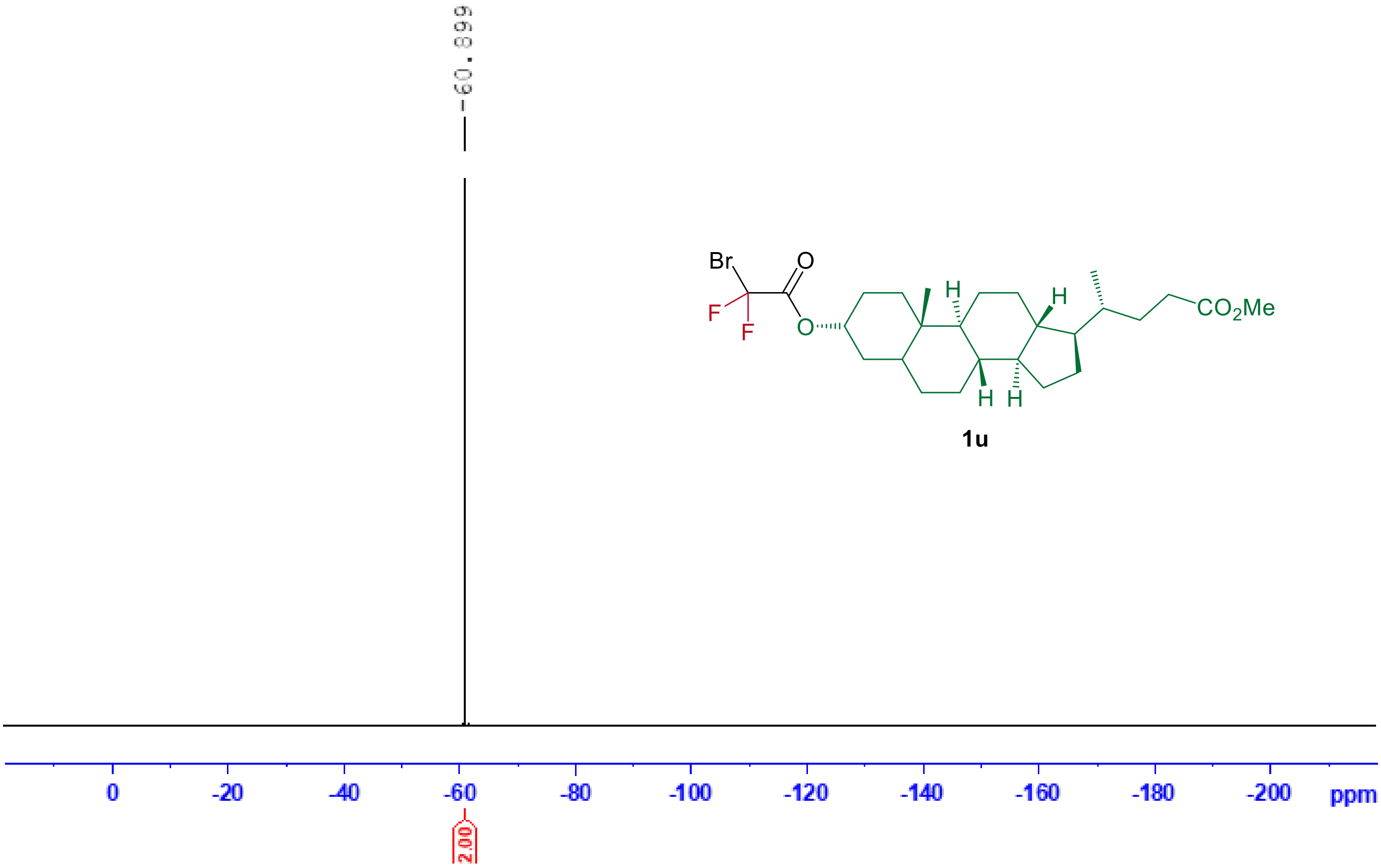

-60.899
Br
O
F
F
O
H
H
H
H
CO2Me
1u
0
-20
-40
-60
-80
-100
-120
-140
-160
-180
-200
ppm
2.00

^{1}H NMR of **1v**

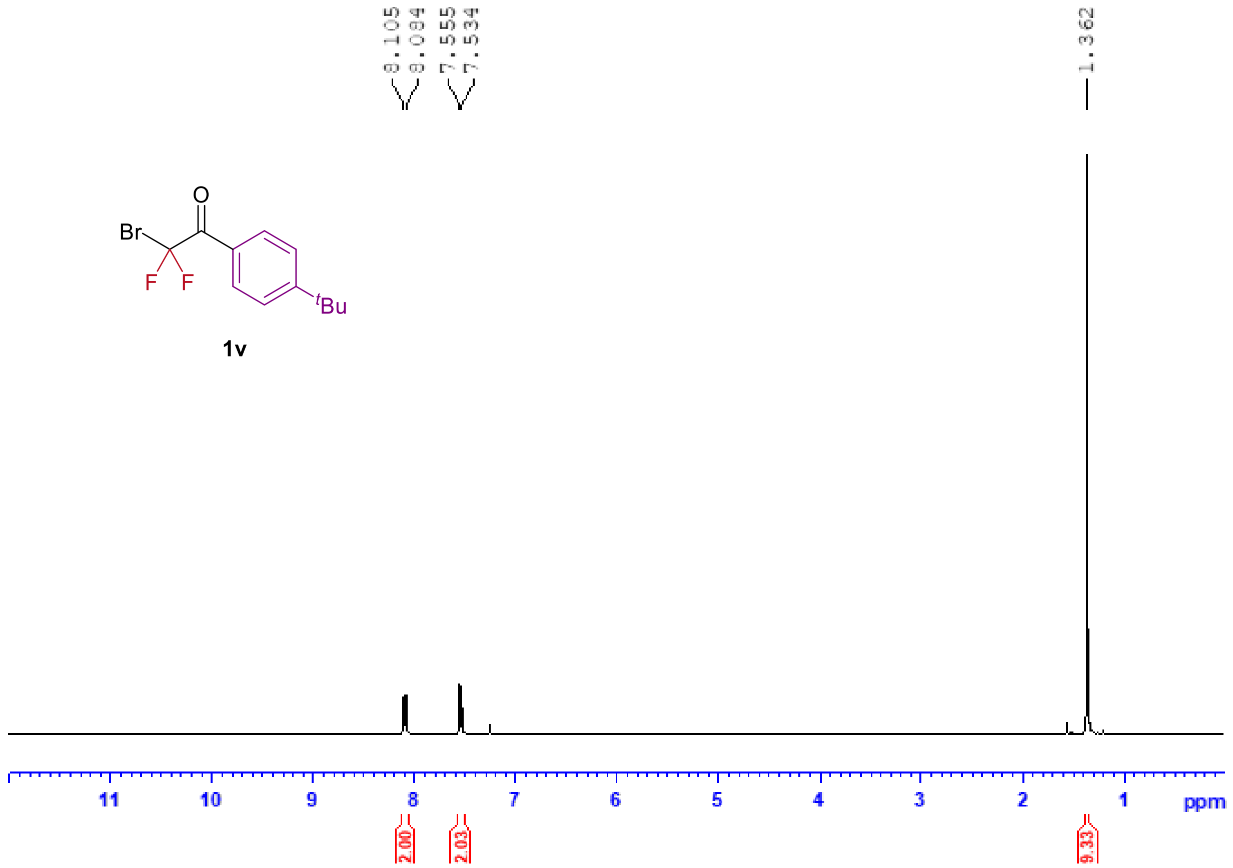

^{13}C NMR of **1v**

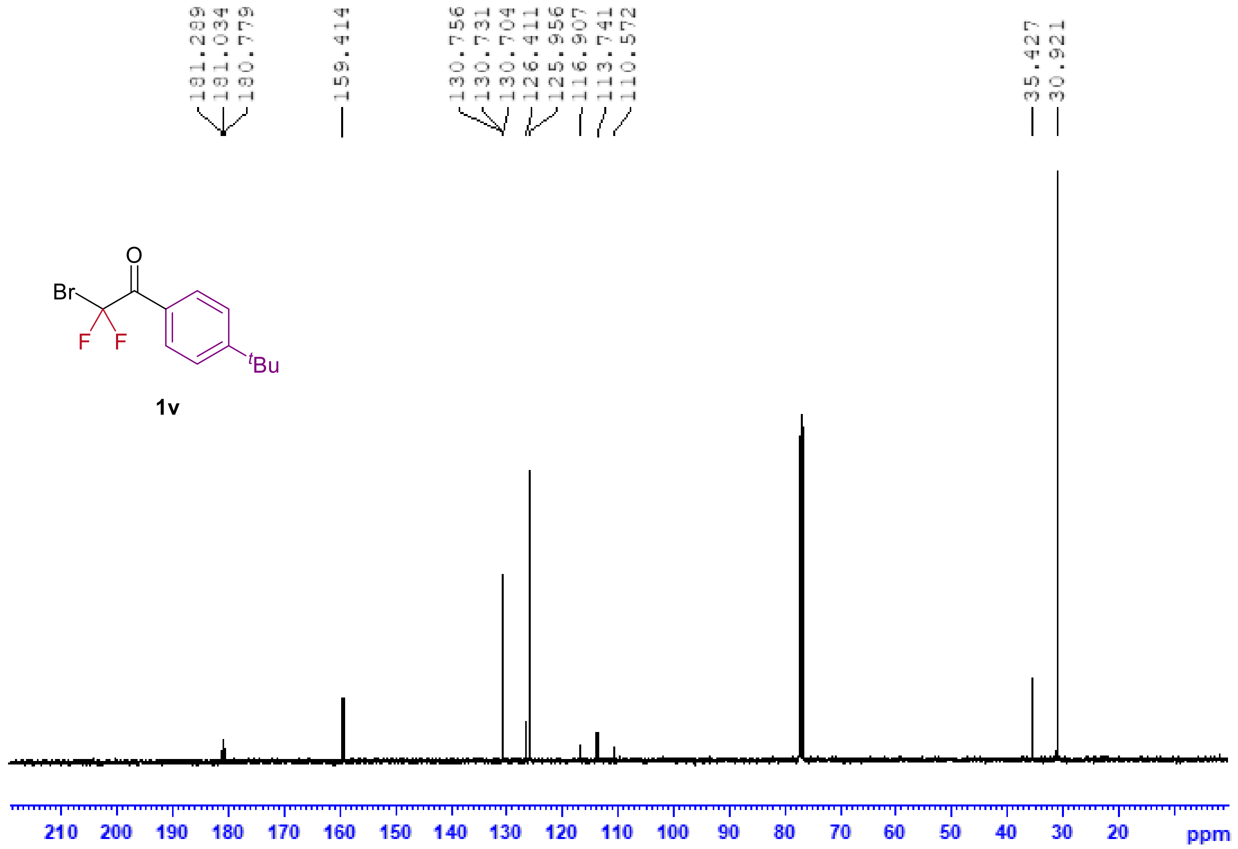

^{19}F NMR of **1v**

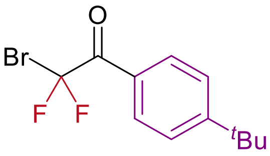

1v

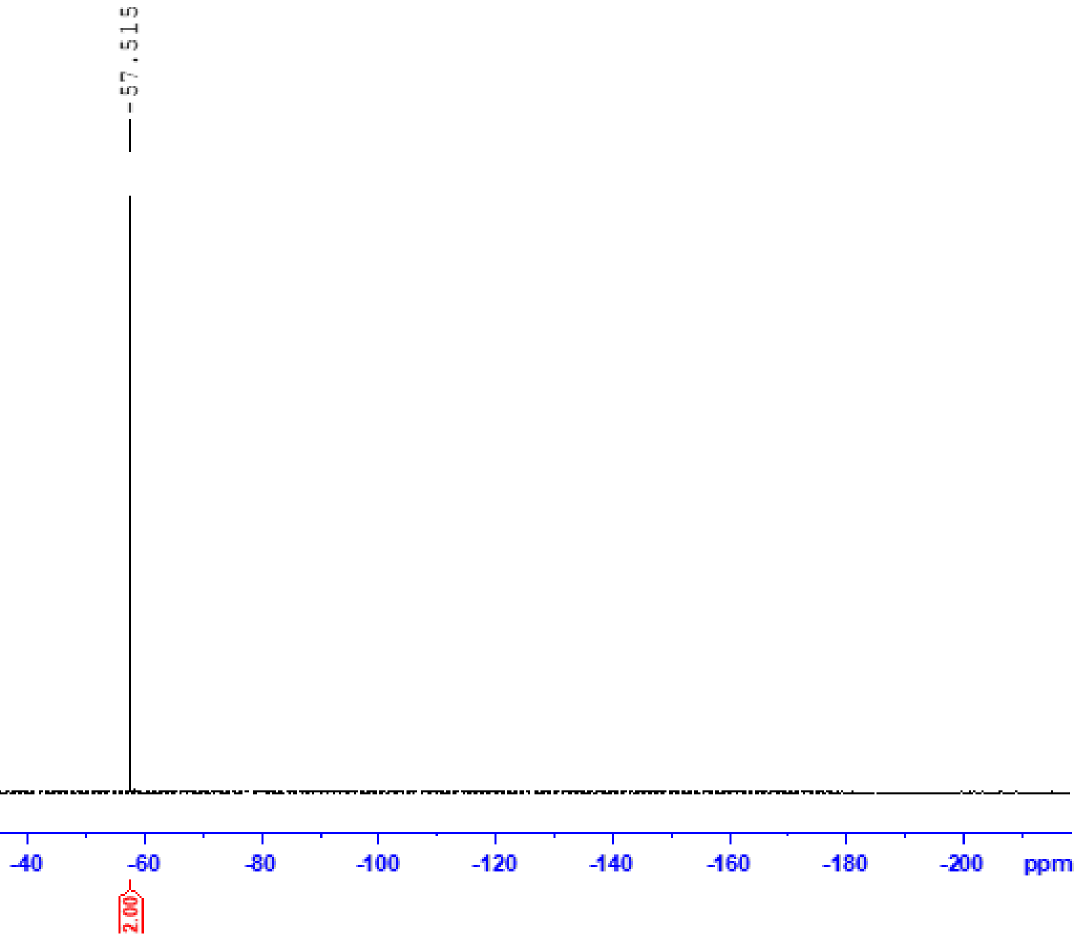

^{1}H NMR of **1w**

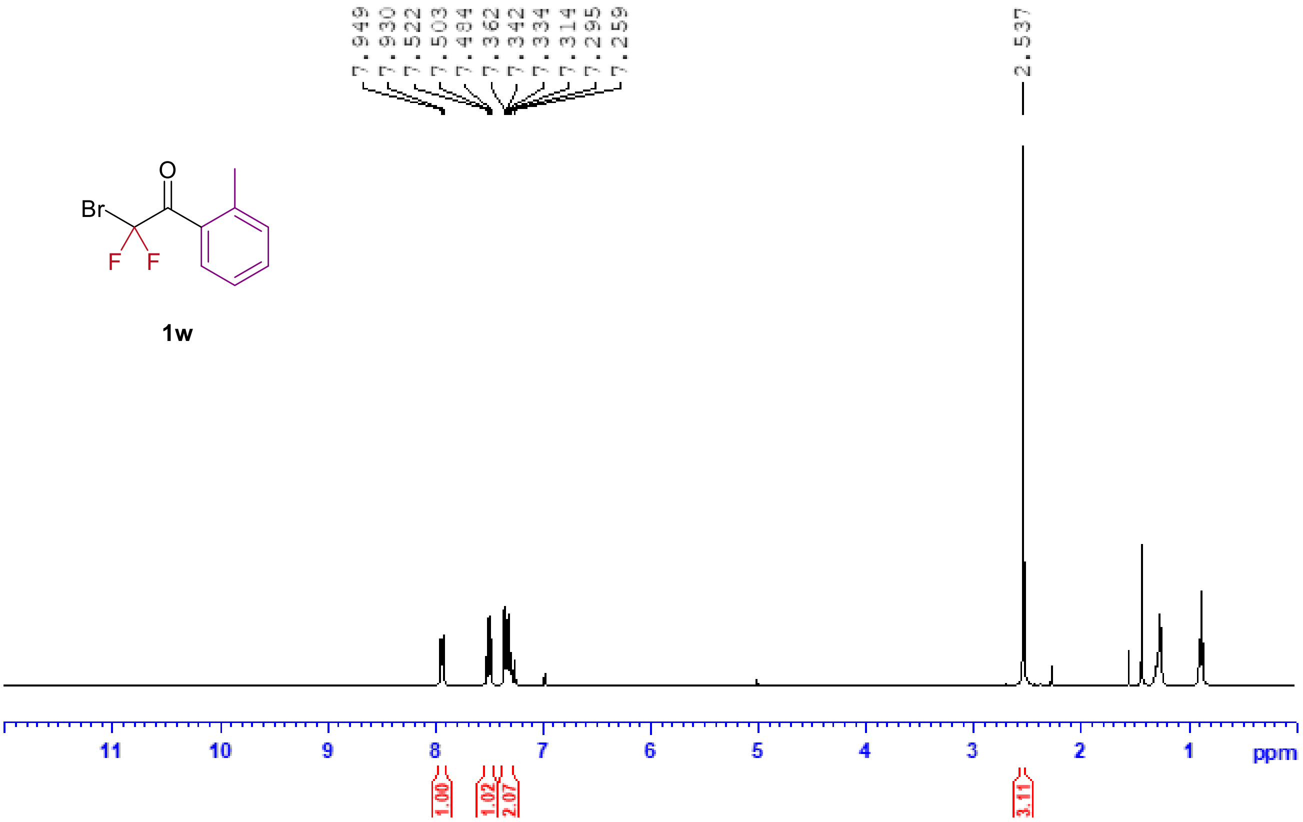

^{13}C NMR of **1w**

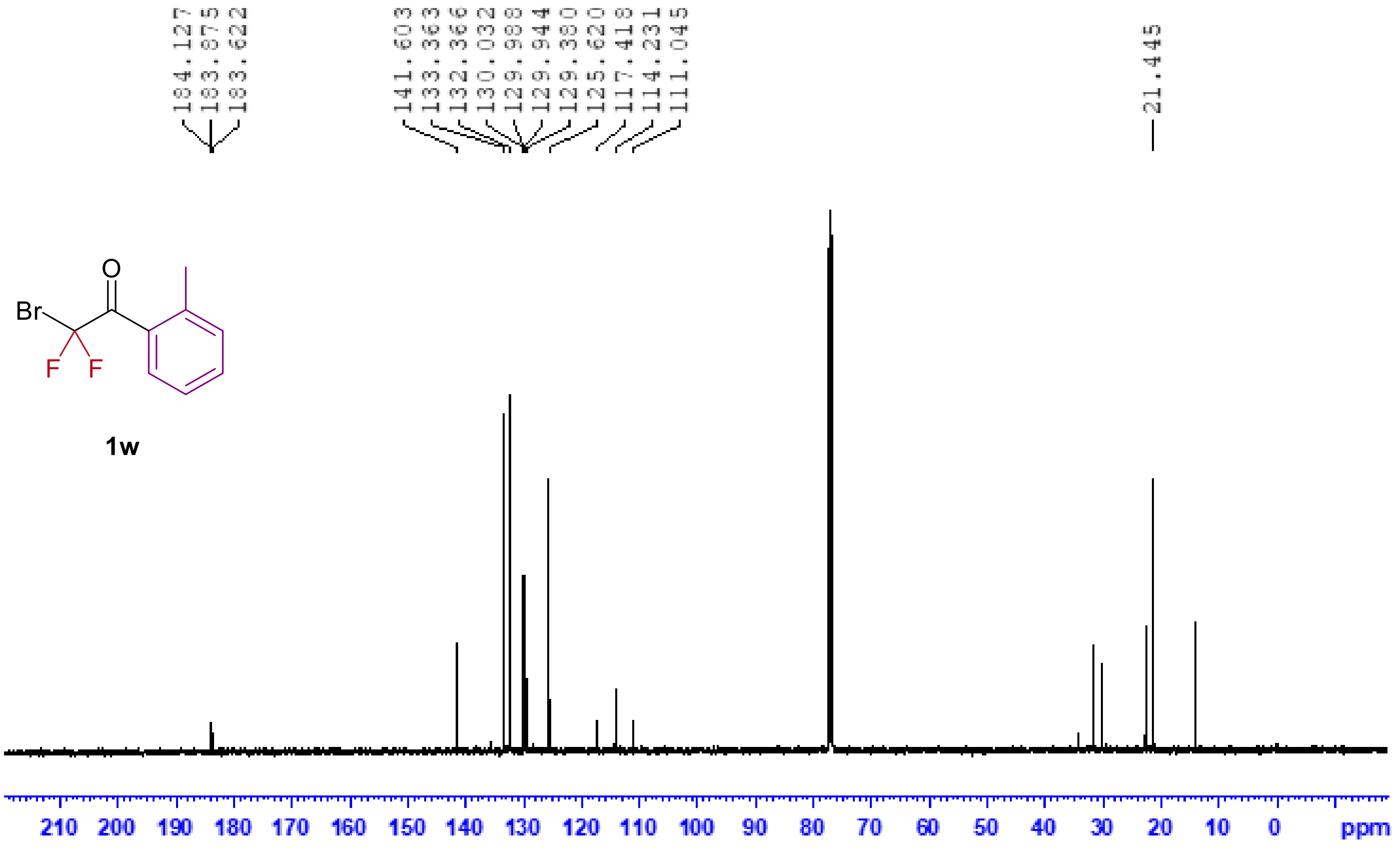

^{19}F NMR of **1w**

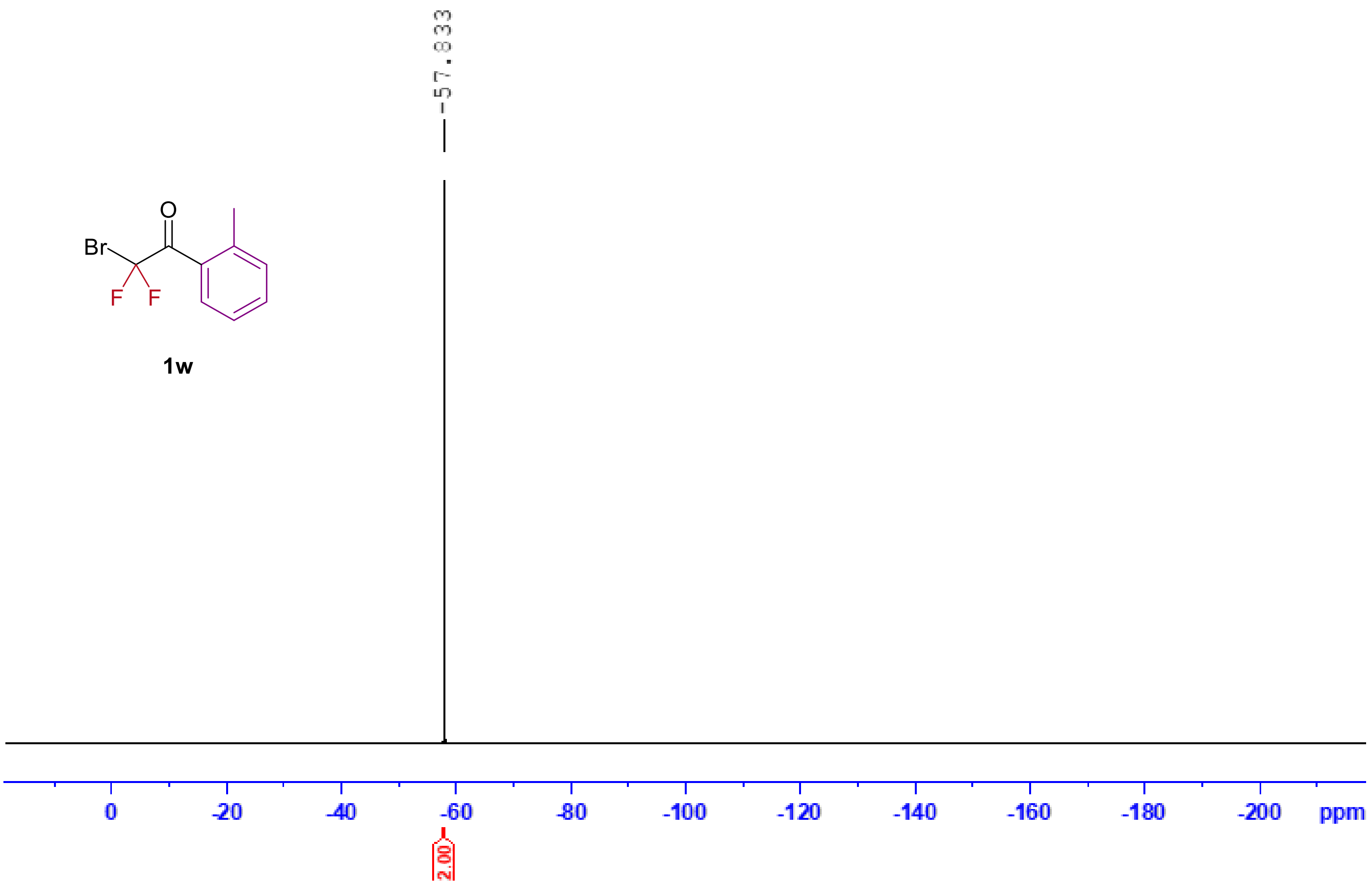

^{1}H NMR of **1x**

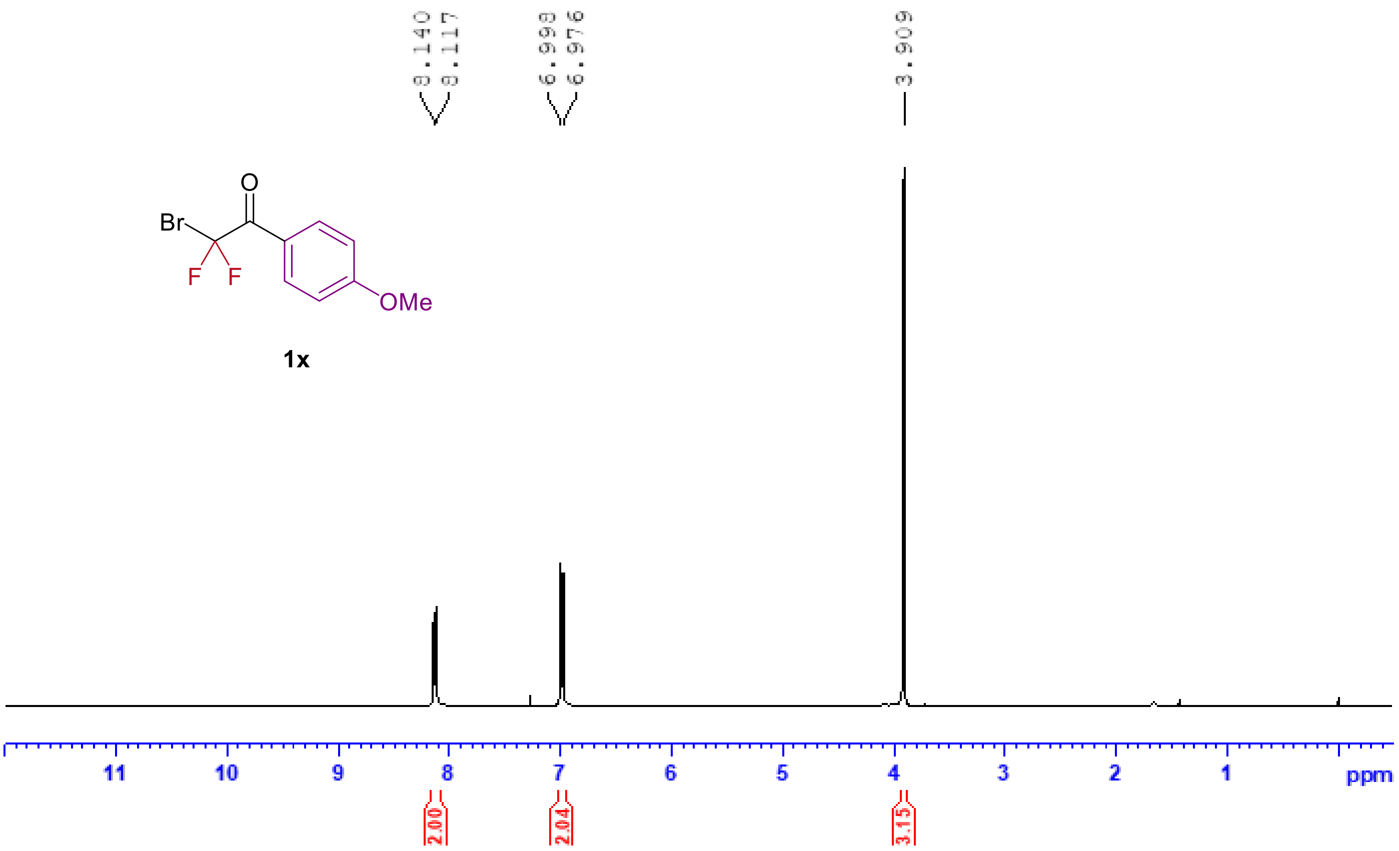

^{13}C NMR of **1x**

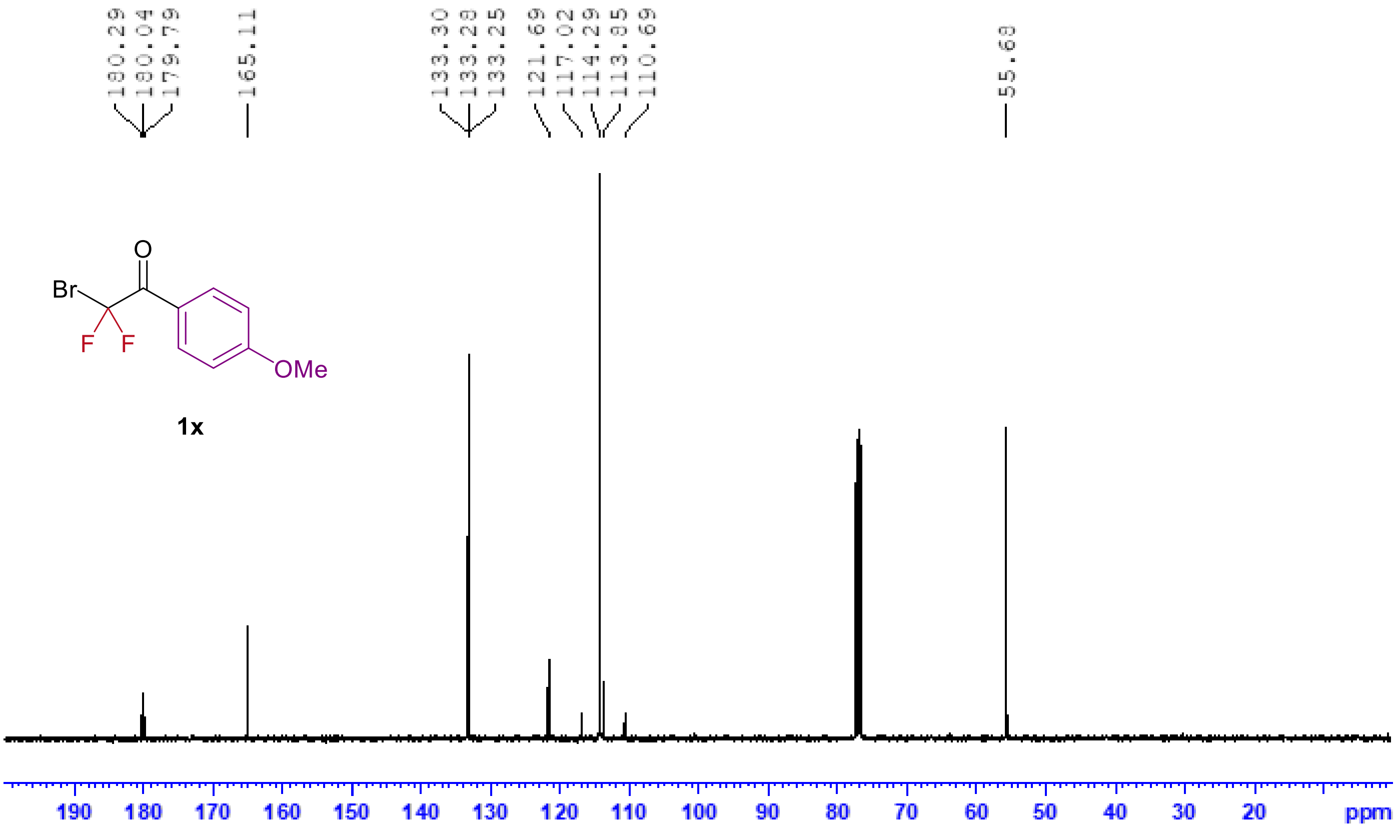

^{19}F NMR of **1x**

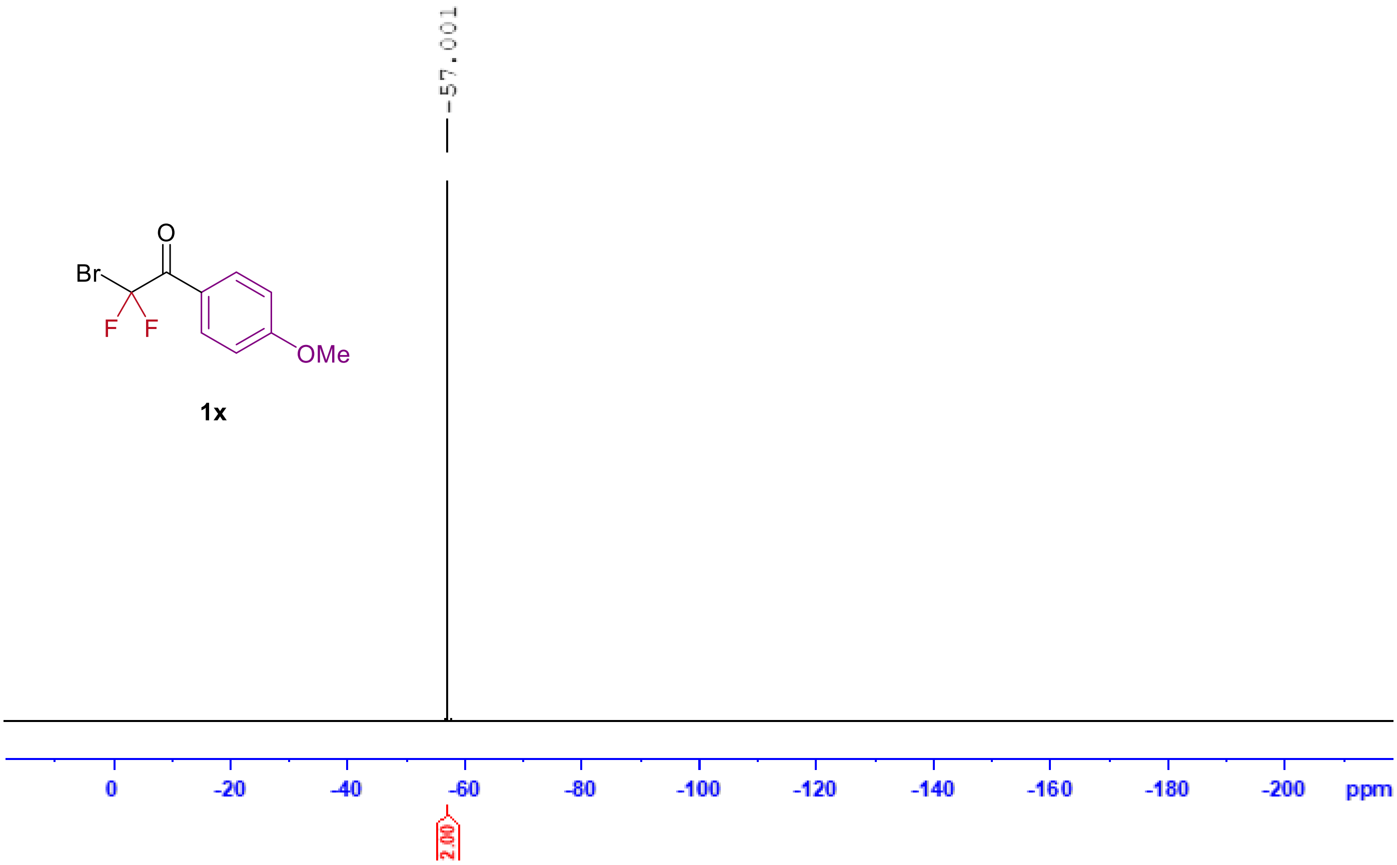

^{1}H NMR of **3a**

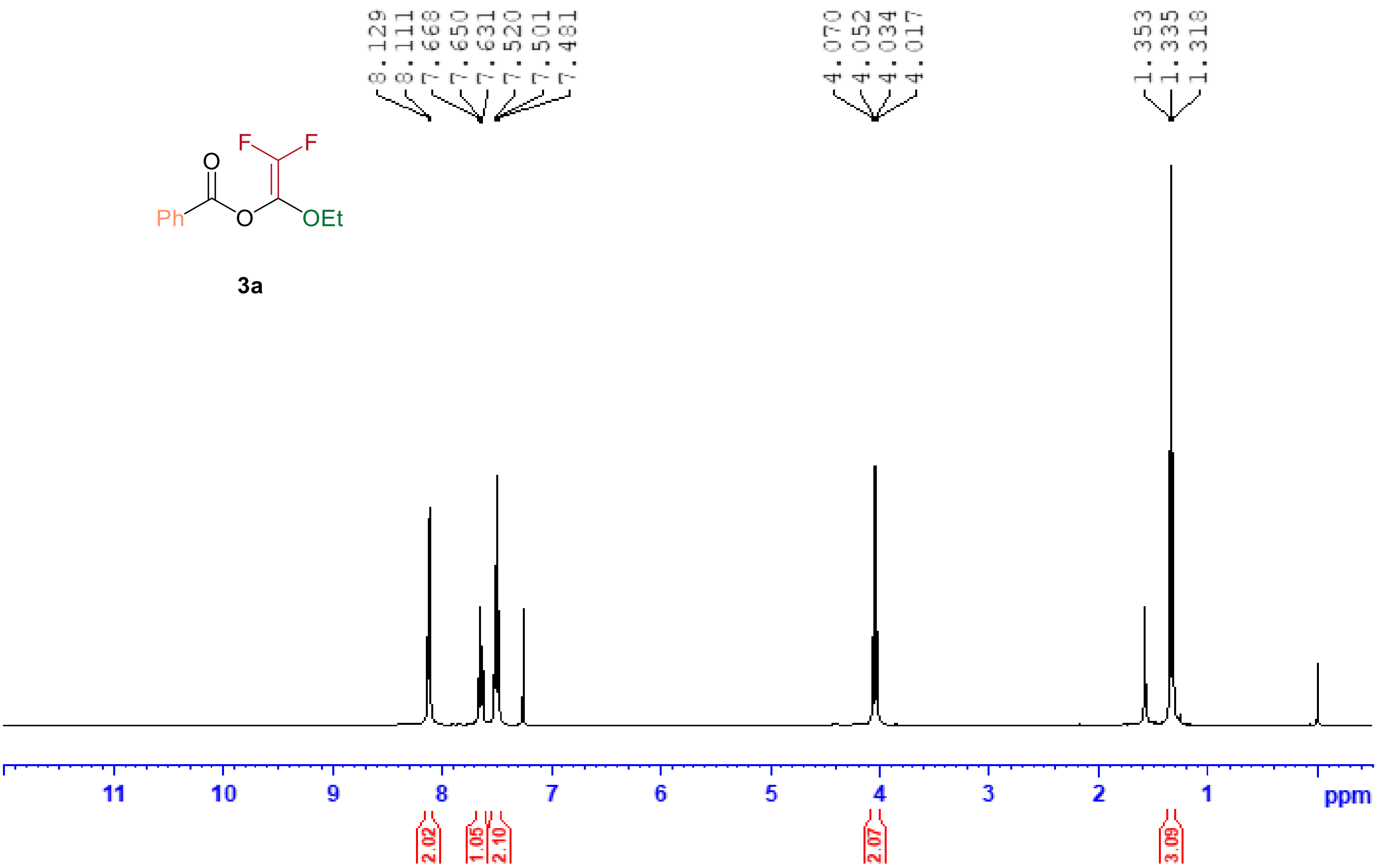

^{13}C NMR of **3a**

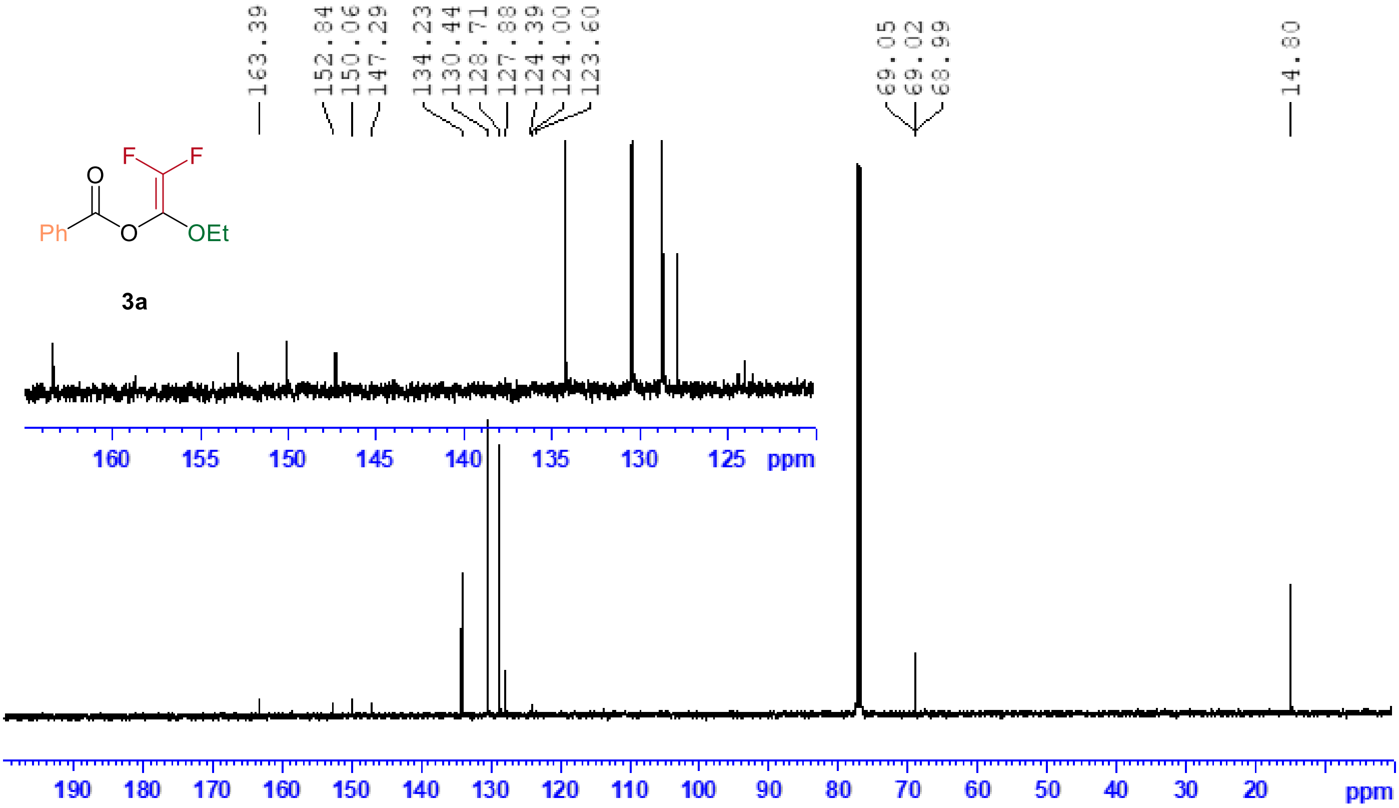

^{19}F NMR of **3a**

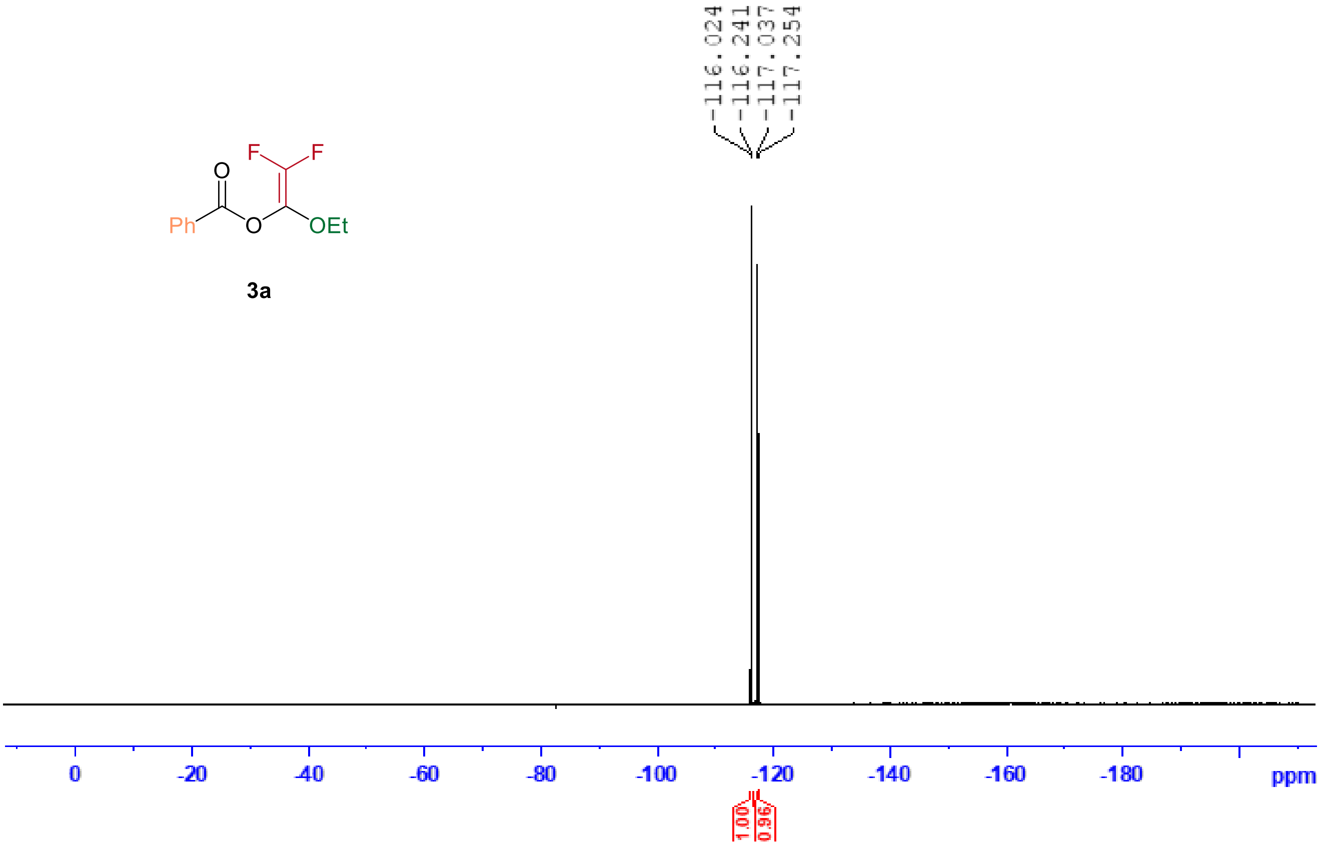

F
F
O
Ph
O
OEt
3a
-116.024
-116.241
-117.037
-117.254
1.00
0.96
0
-20
-40
-60
-80
-100
-120
-140
-160
-180
ppm

^{1}H NMR of **3b**

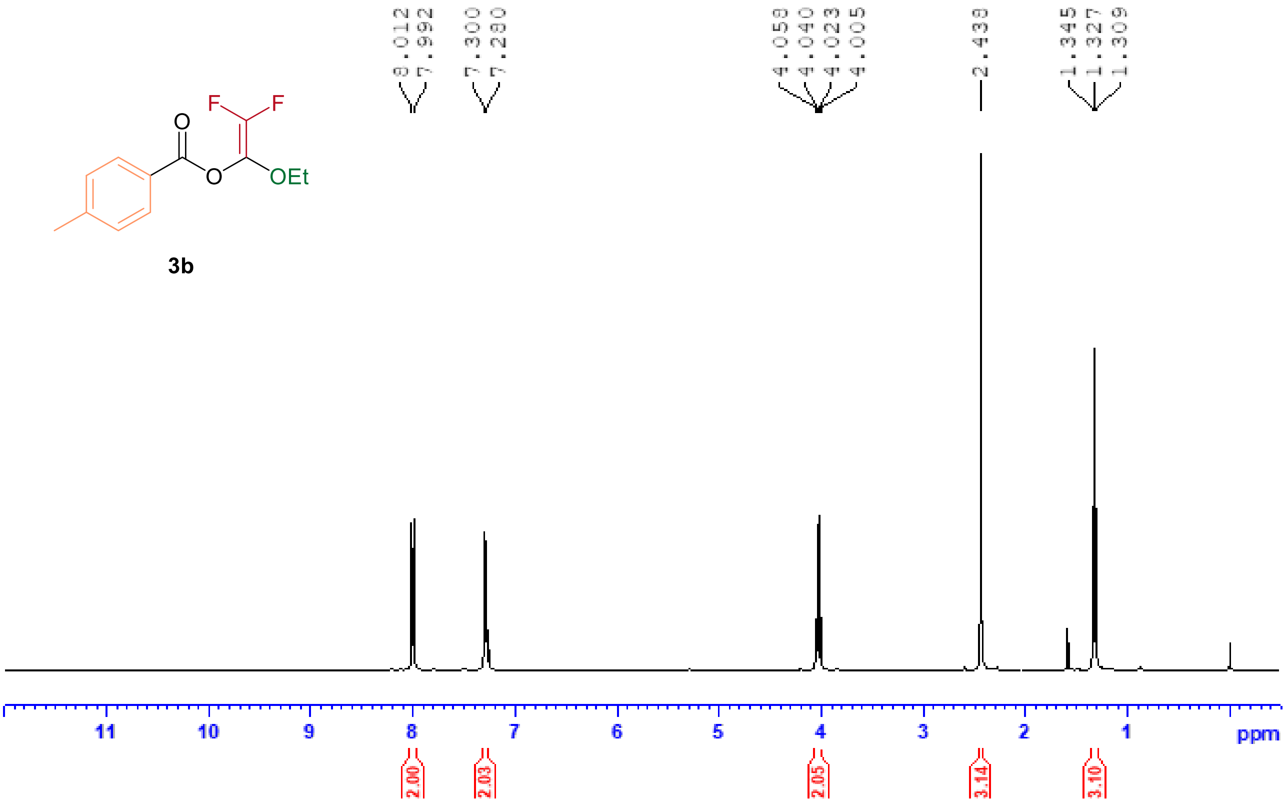

^{13}C NMR of **3b**

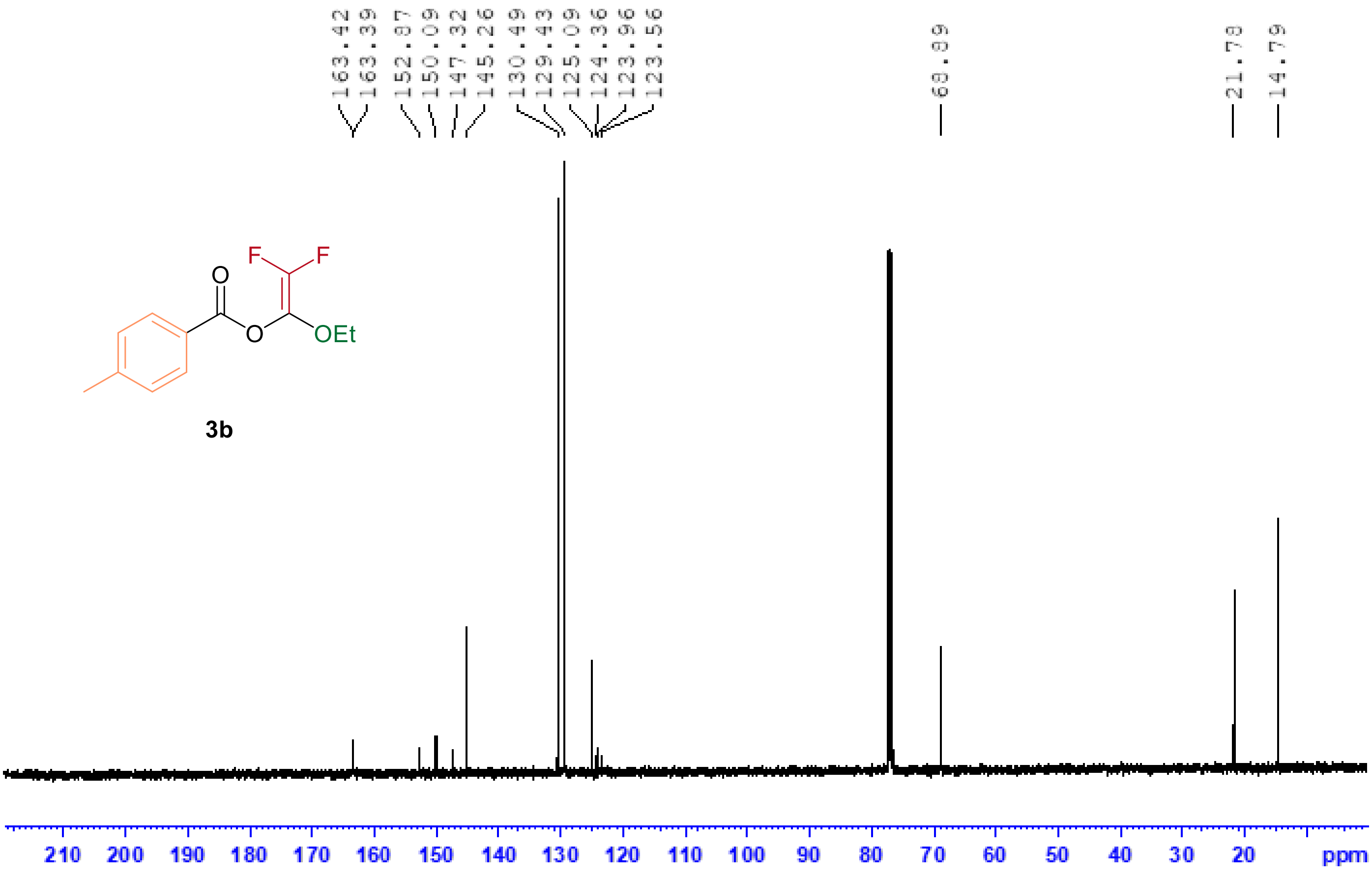

^{19}F NMR of **3b**

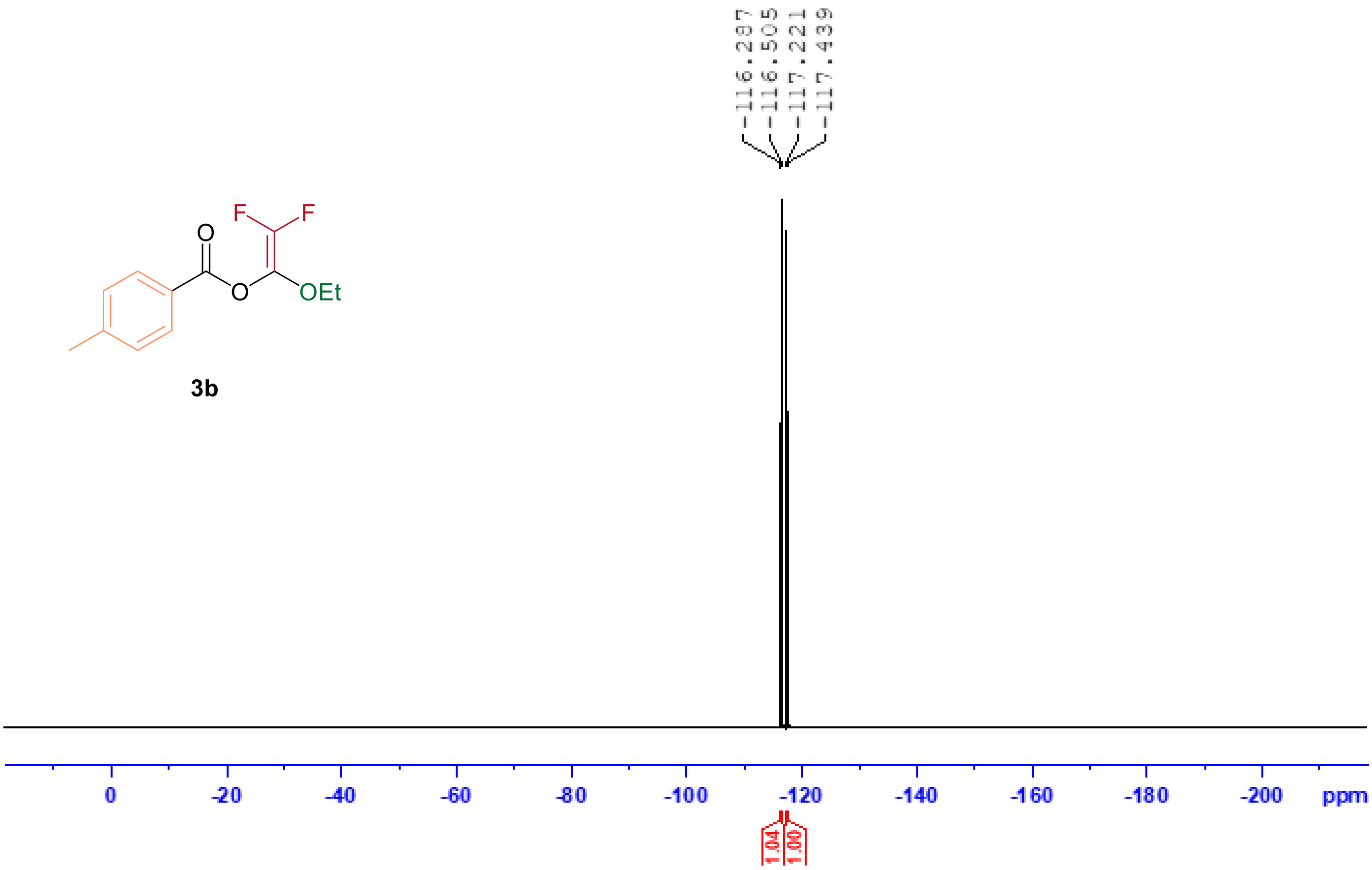

O
F
F
O
OEt
3b
-116.287
-116.505
-117.221
-117.439
0
-20
-40
-60
-80
-100
-120
-140
-160
-180
-200
ppm
1.04
1.00

^{1}H NMR of **3c**

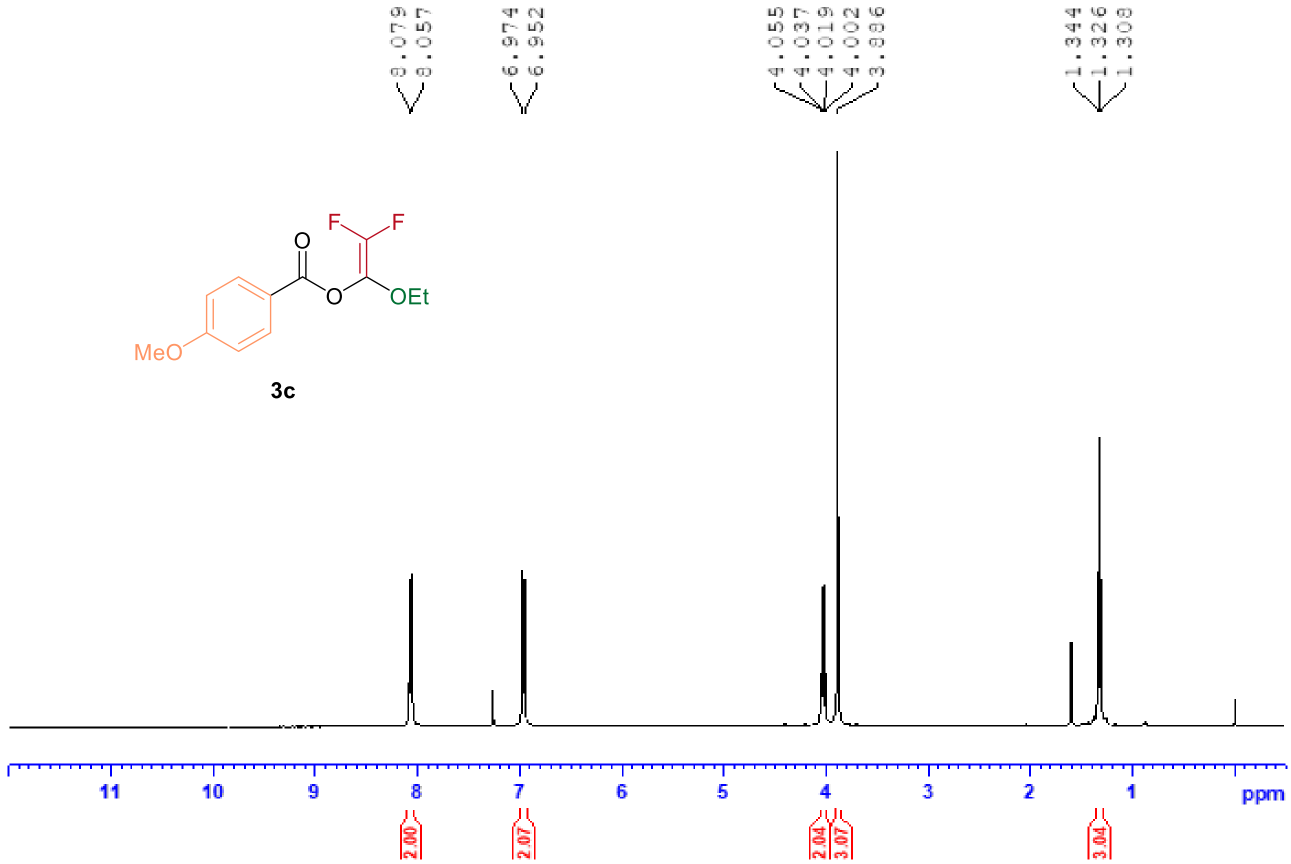

^{13}C NMR of **3c**

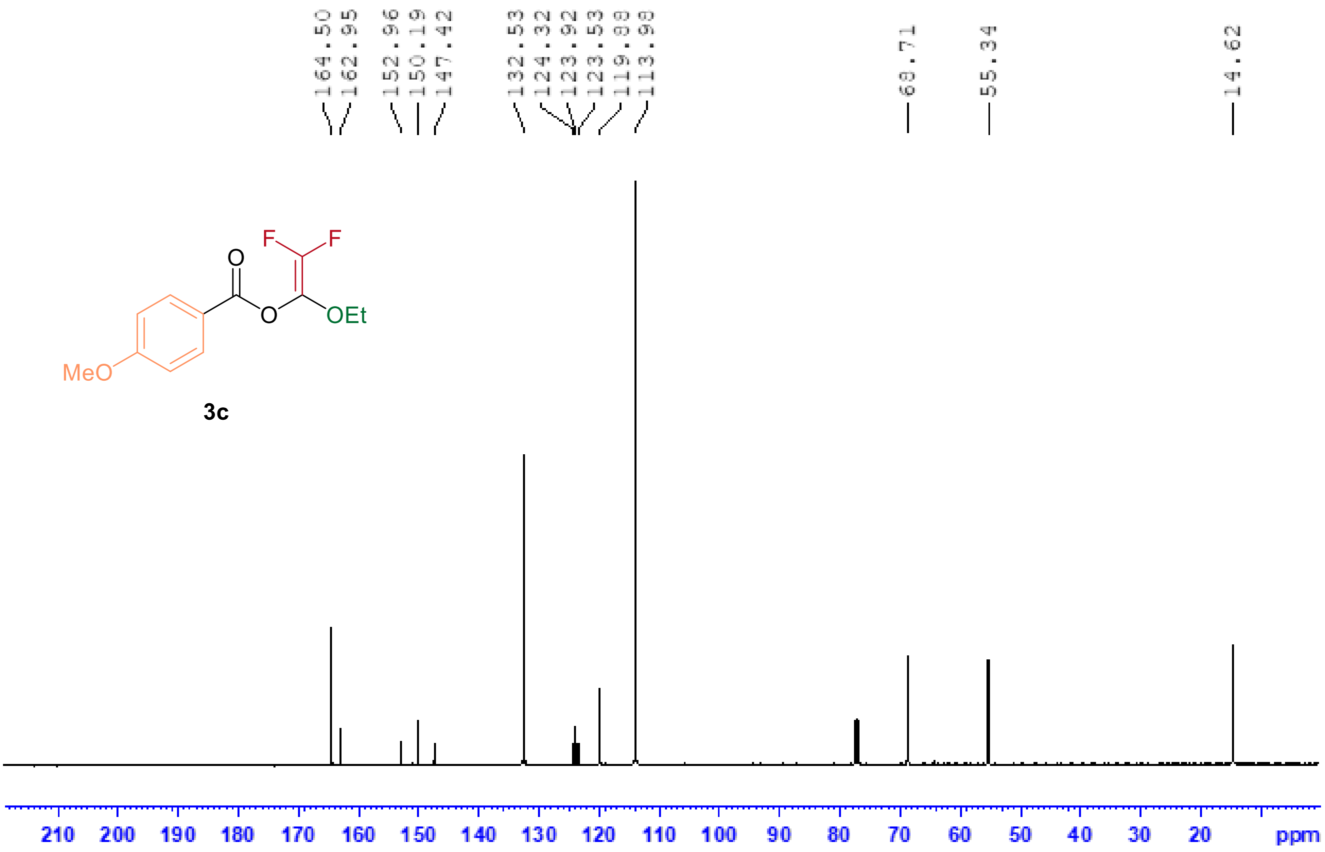

^{19}F NMR of **3c**

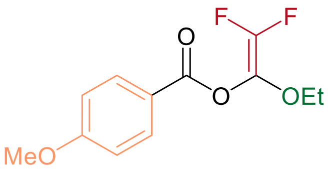

3c

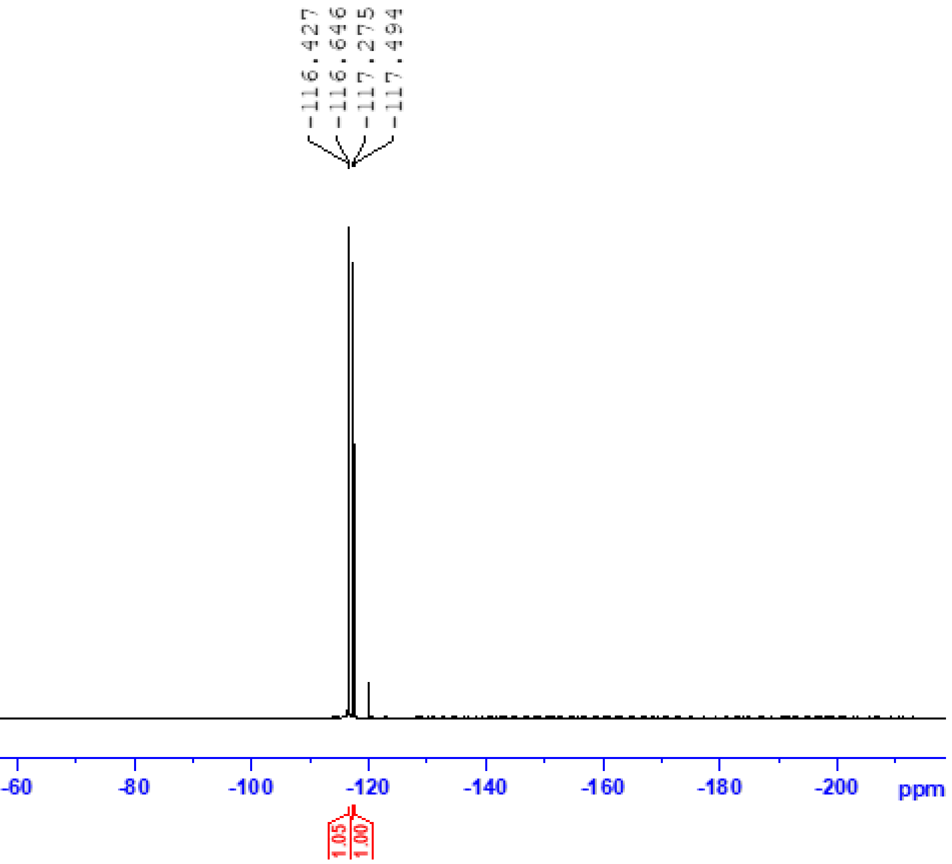

^{1}H NMR of **3d**

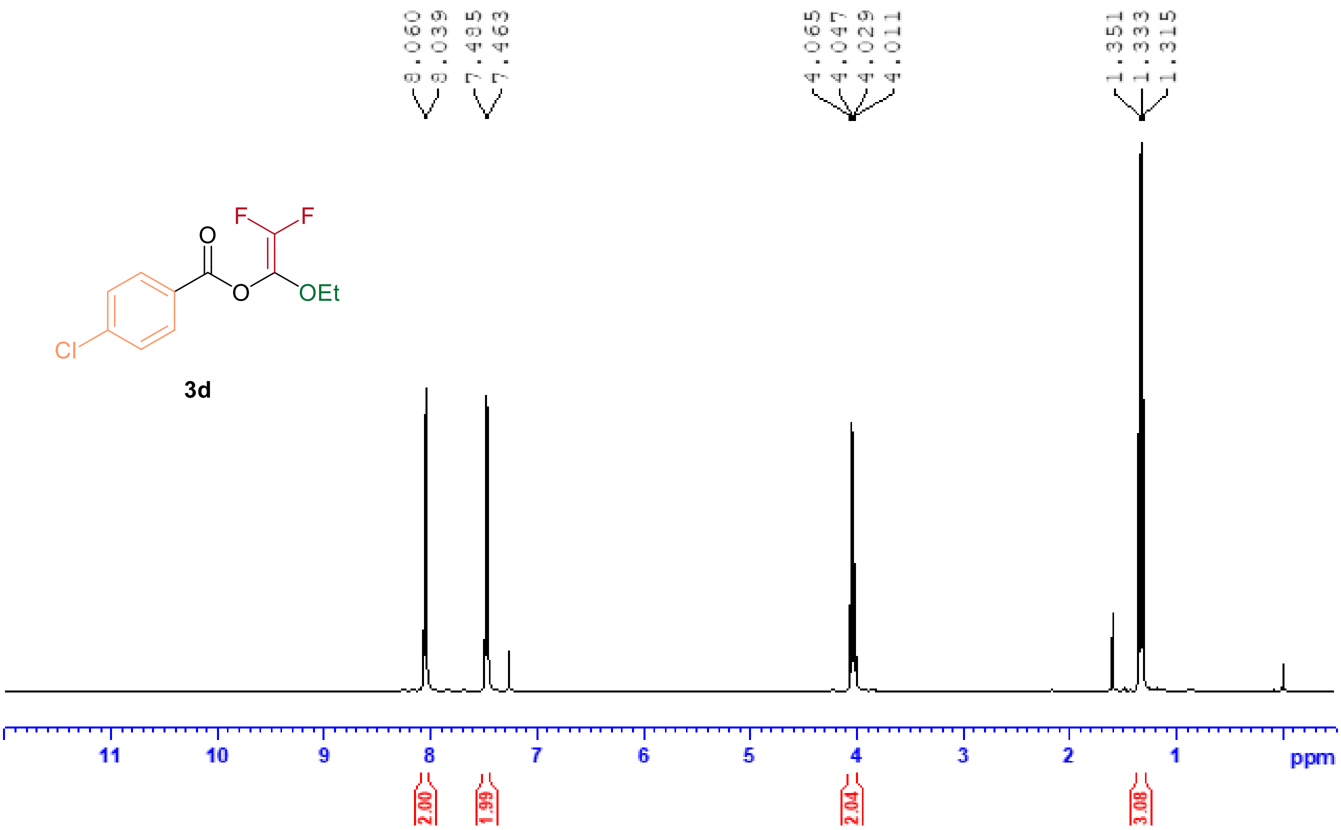

^{13}C NMR of **3d**

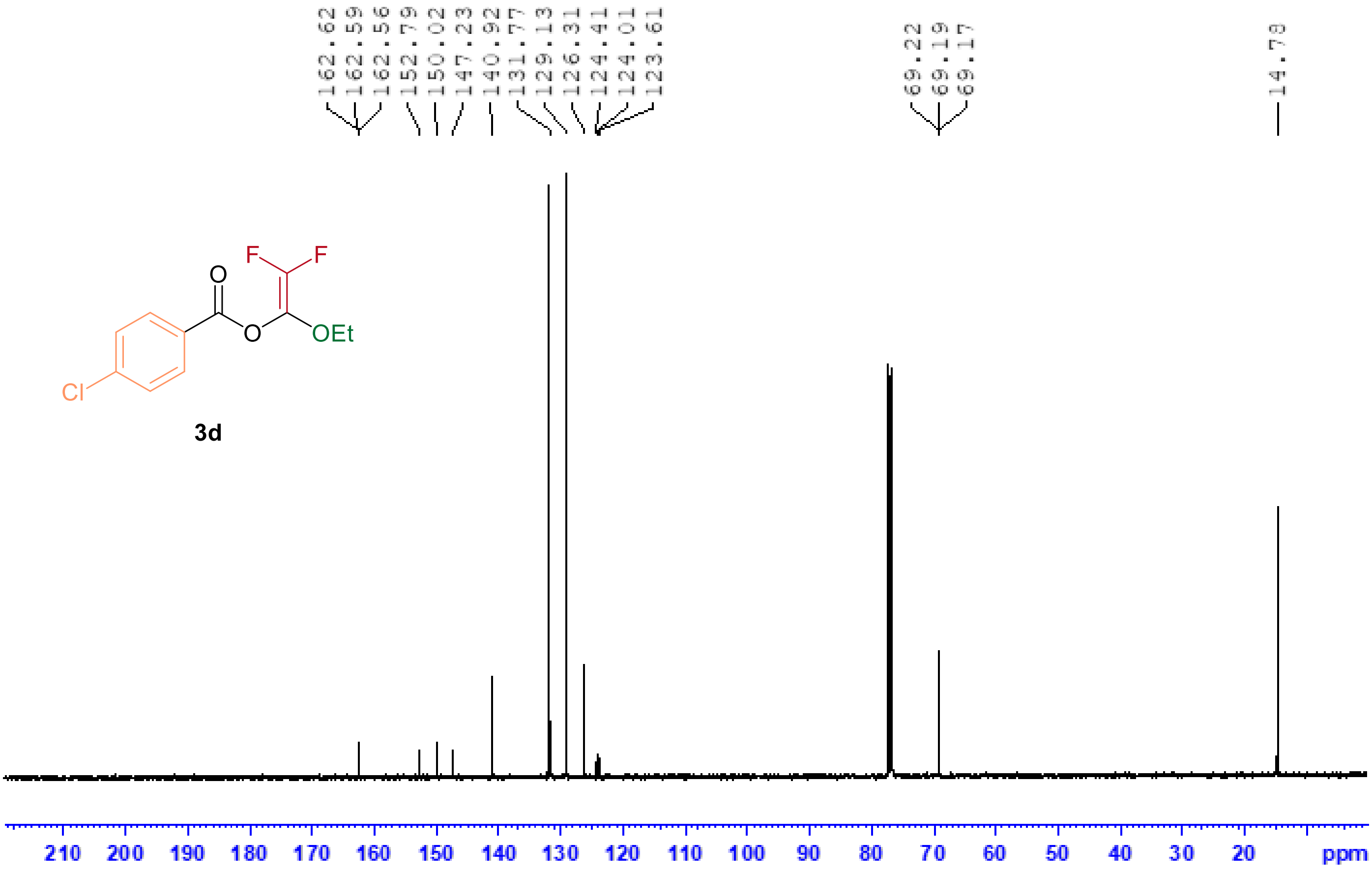

^{19}F NMR of **3d**

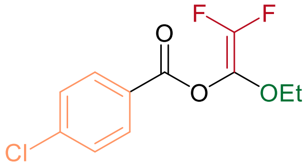

3d

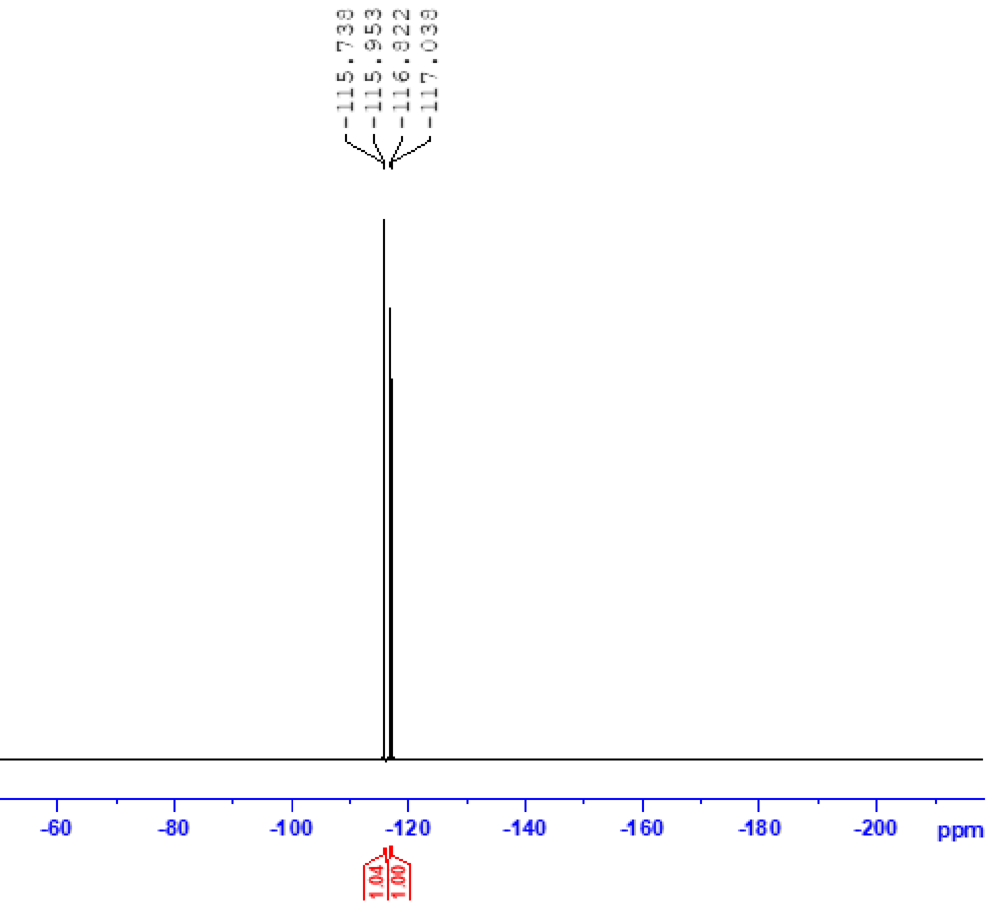

^{1}H NMR of **3e**

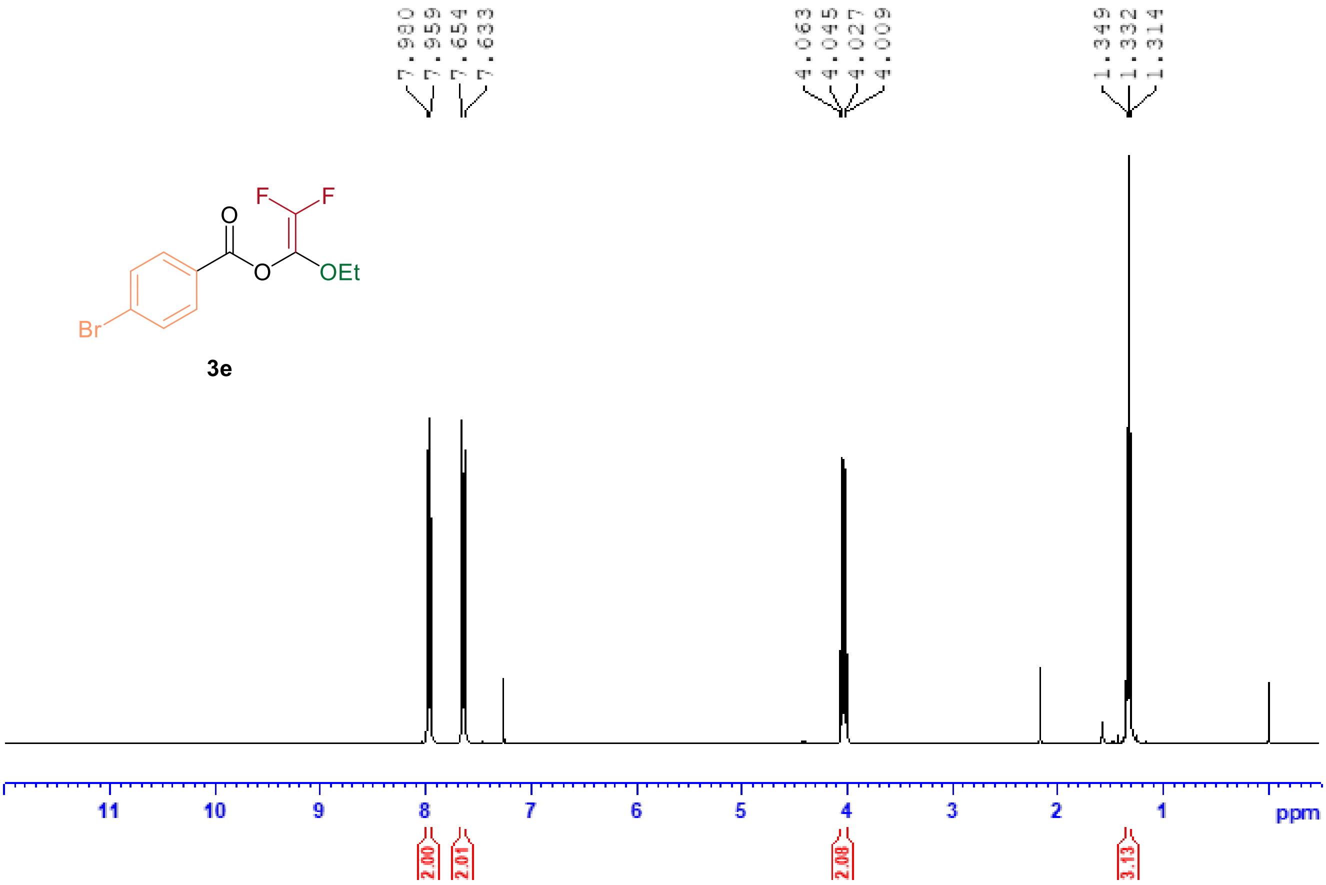

^{13}C NMR of **3e**

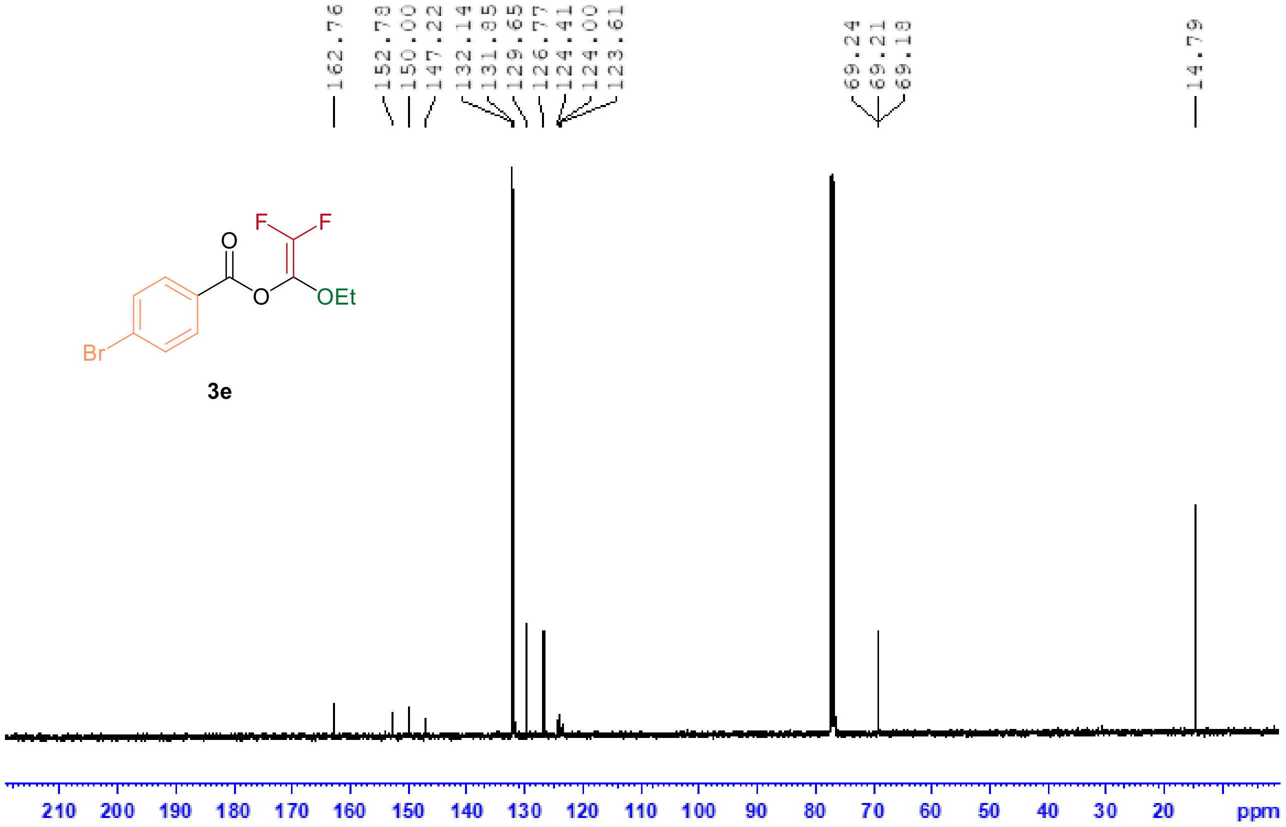

^{19}F NMR of **3e**

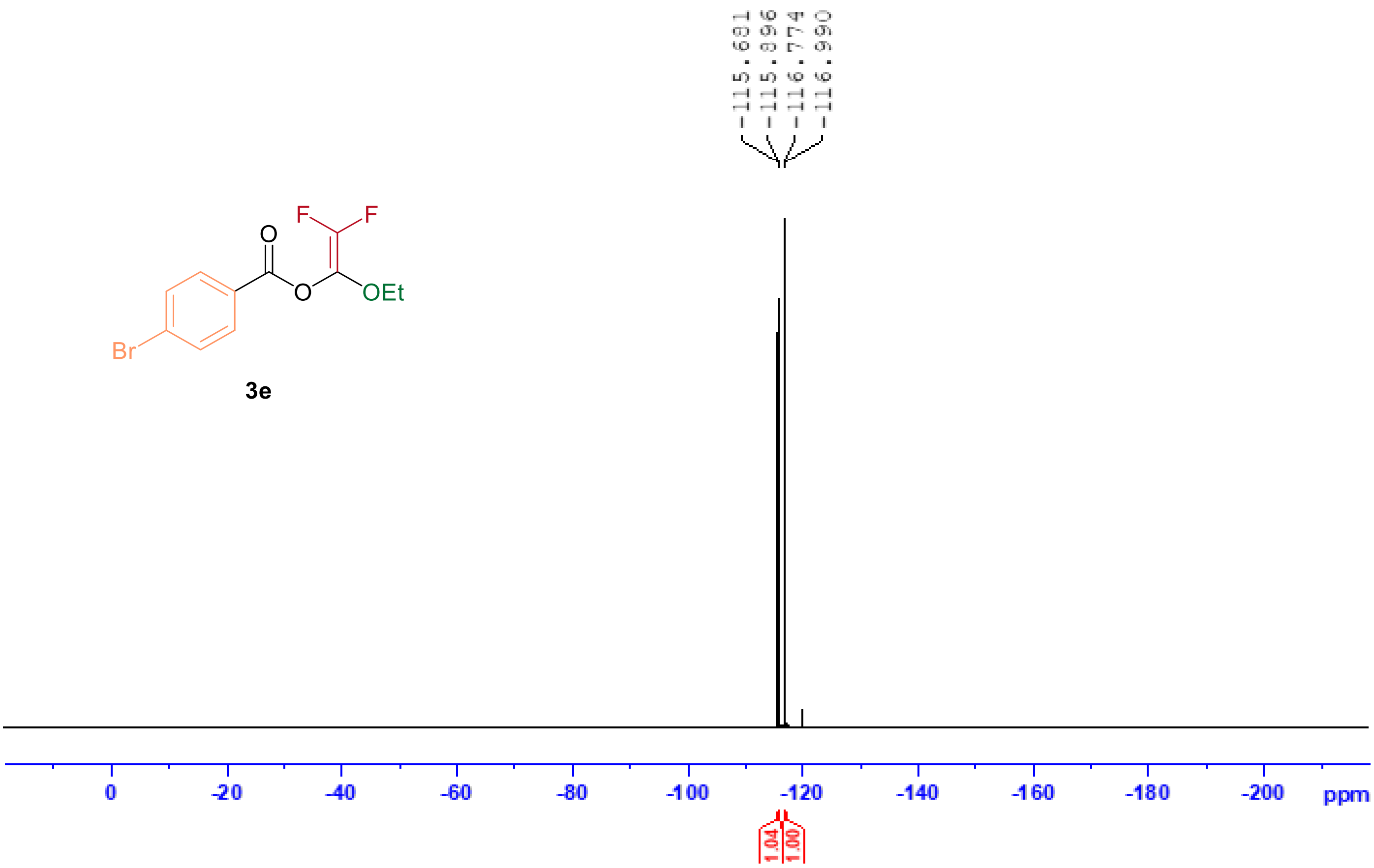

-115.681
-115.896
-116.774
-116.990
F
F
O
O
OEt
Br
3e
0
-20
-40
-60
-80
-100
-120
-140
-160
-180
-200
ppm
1.04
1.00

^{1}H NMR of **3f**

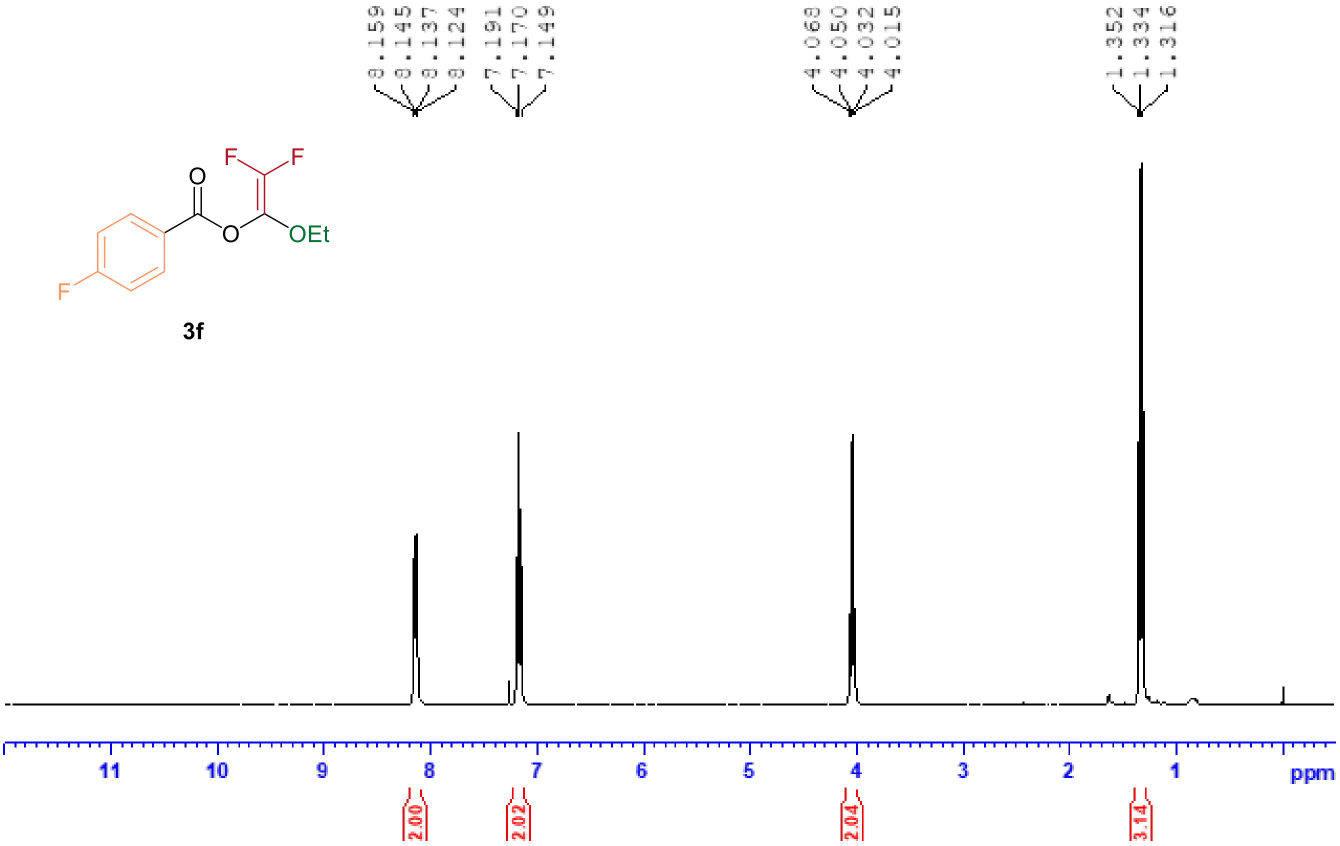

^{13}C NMR of **3f**

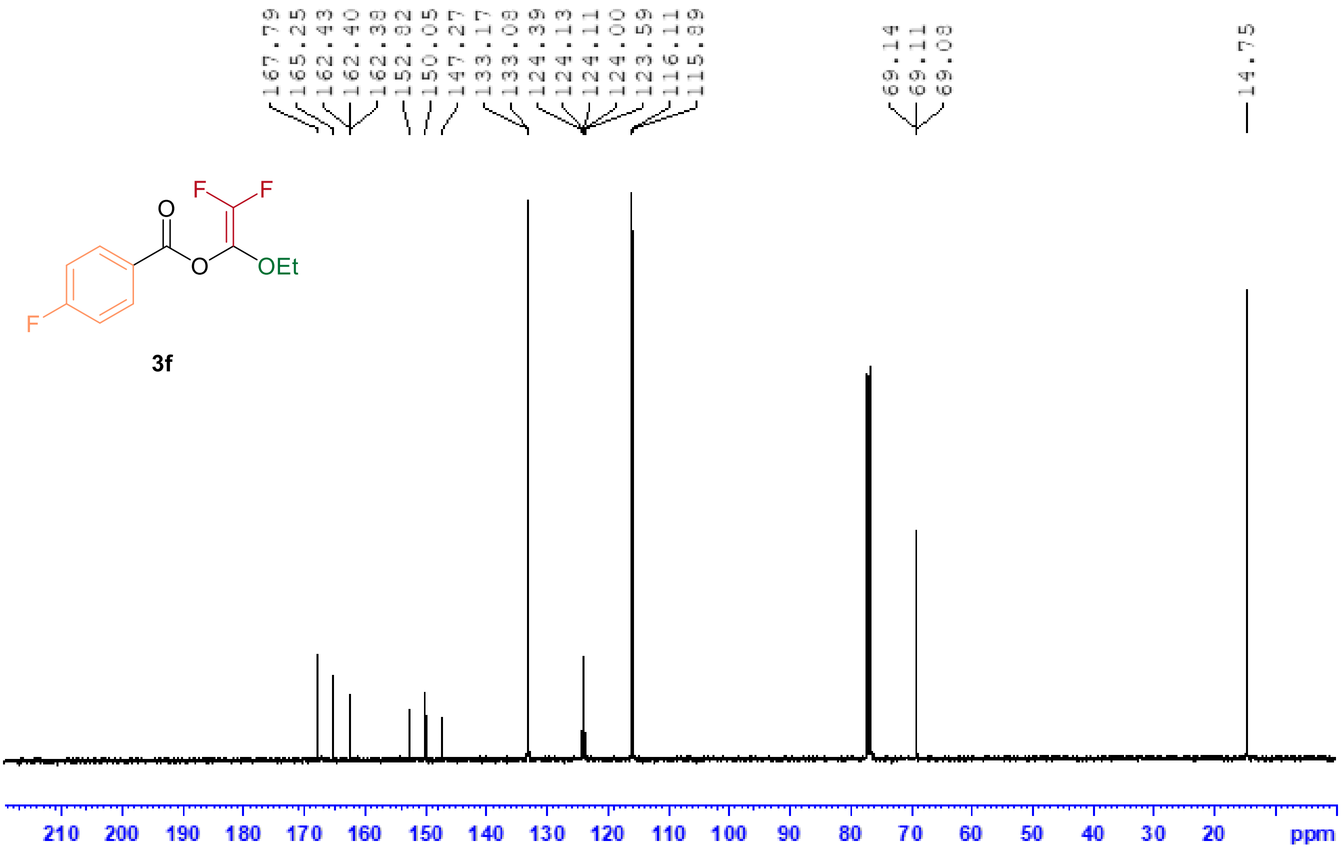

^{19}F NMR of **3f**

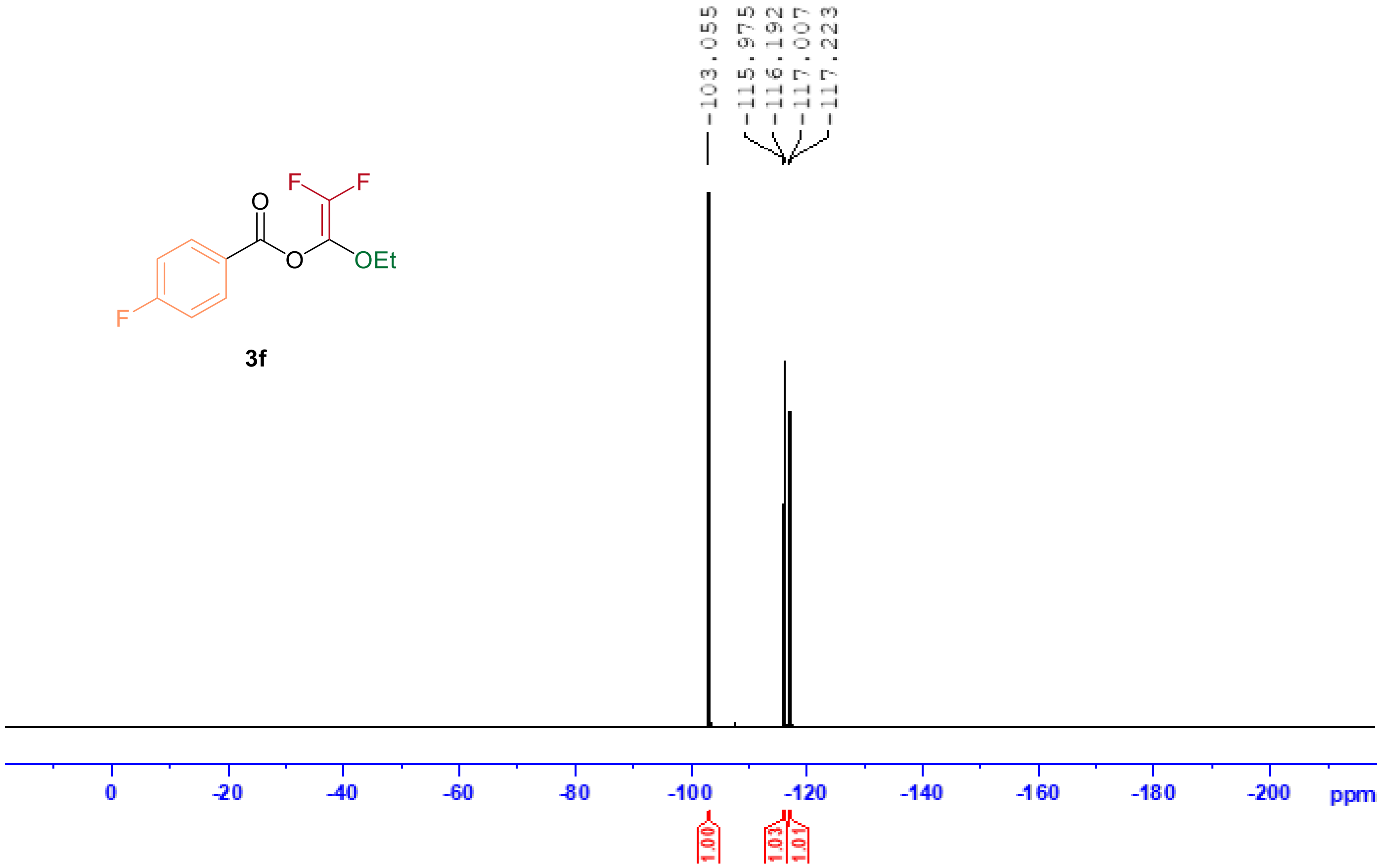

-103.055
-115.975
-116.192
-117.007
-117.223
F
F
O
O
OEt
F
3f
0
-20
-40
-60
-80
-100
-120
-140
-160
-180
-200
ppm
1.00
1.03
1.01

^{1}H NMR of **3g**

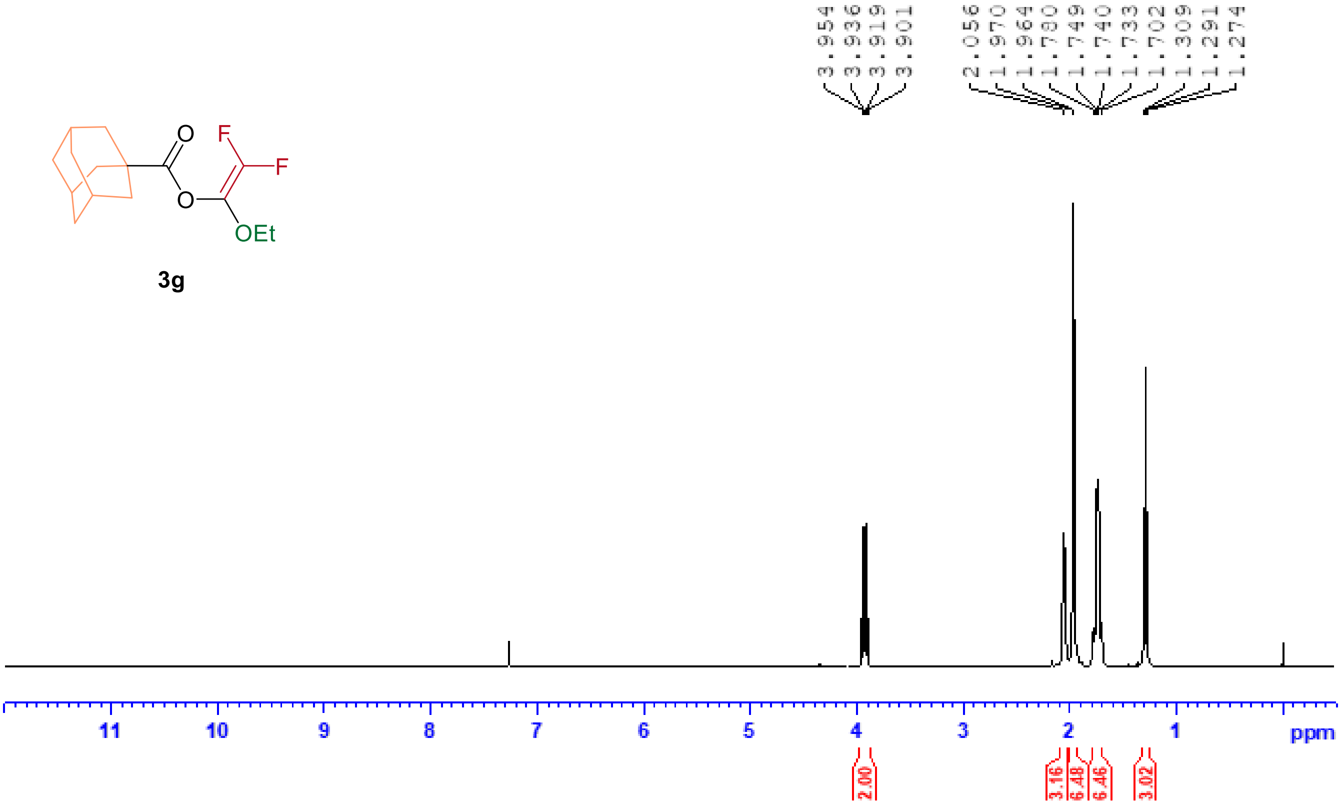

^{13}C NMR of **3g**

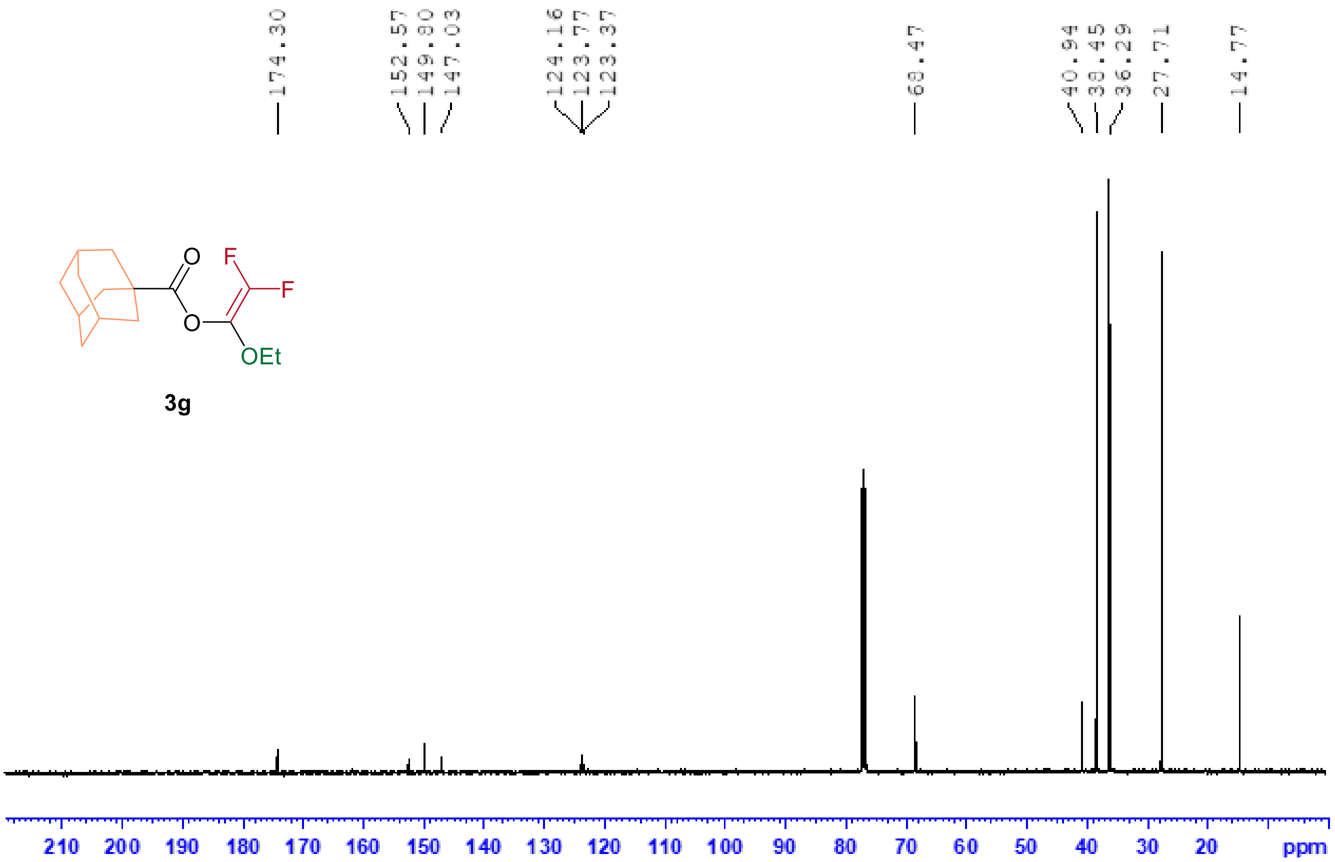

^{19}F NMR of **3g**

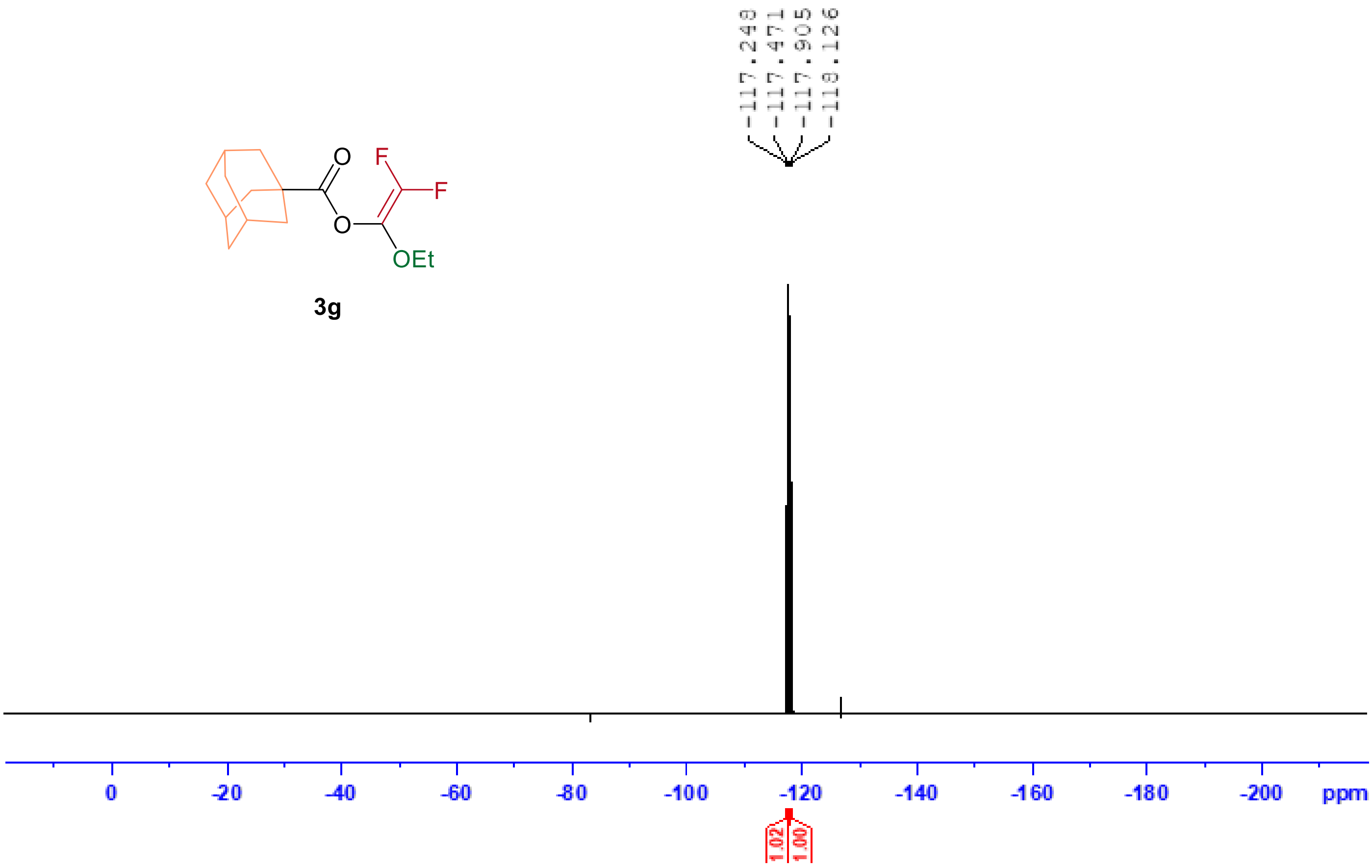

-117.248
-117.471
-117.905
-118.126
O
F
F
O
OEt
3g
0
-20
-40
-60
-80
-100
-120
-140
-160
-180
-200
ppm
1.02
1.00

^{1}H NMR of **4a**

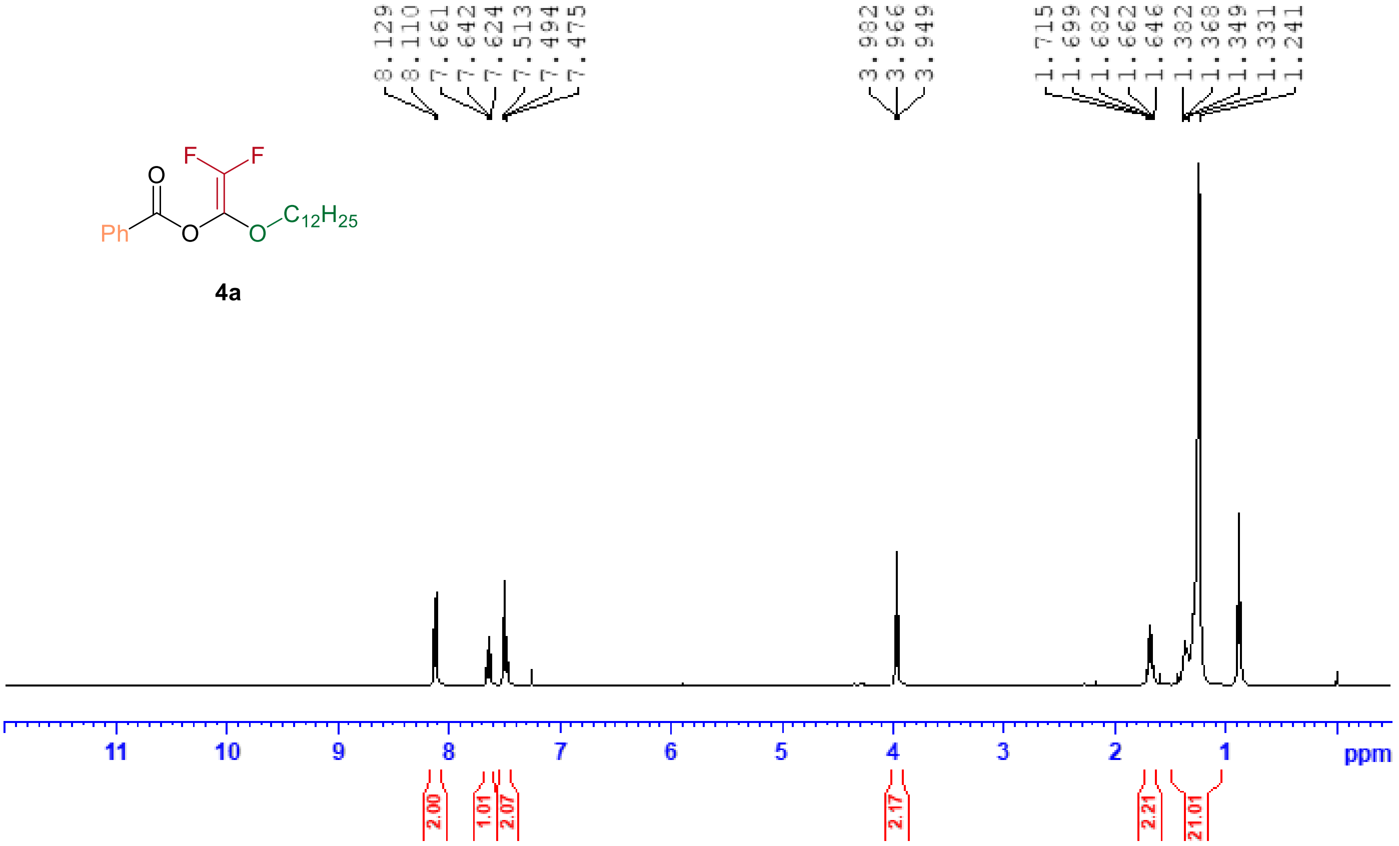

^{13}C NMR of **4a**

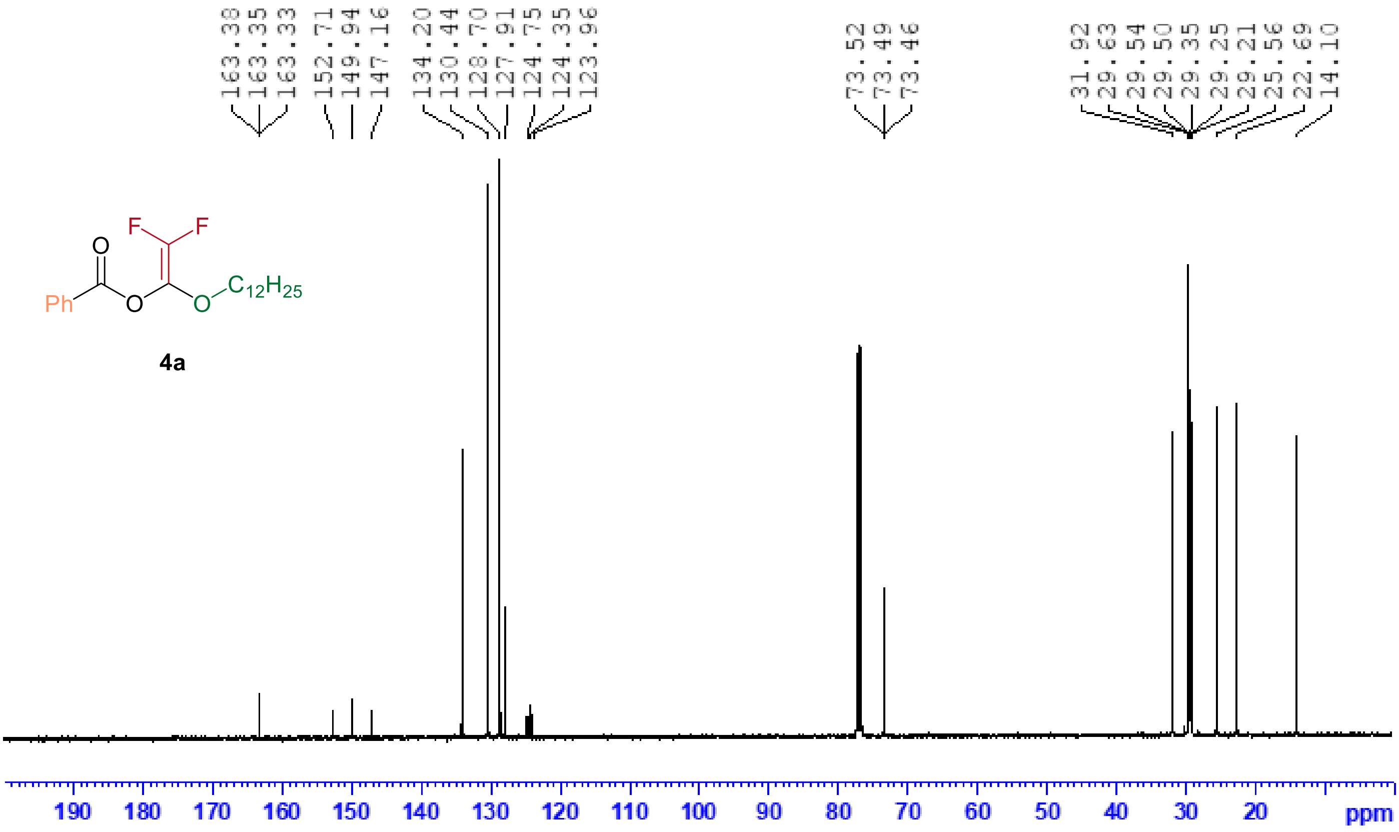

^{19}F NMR of **4a**

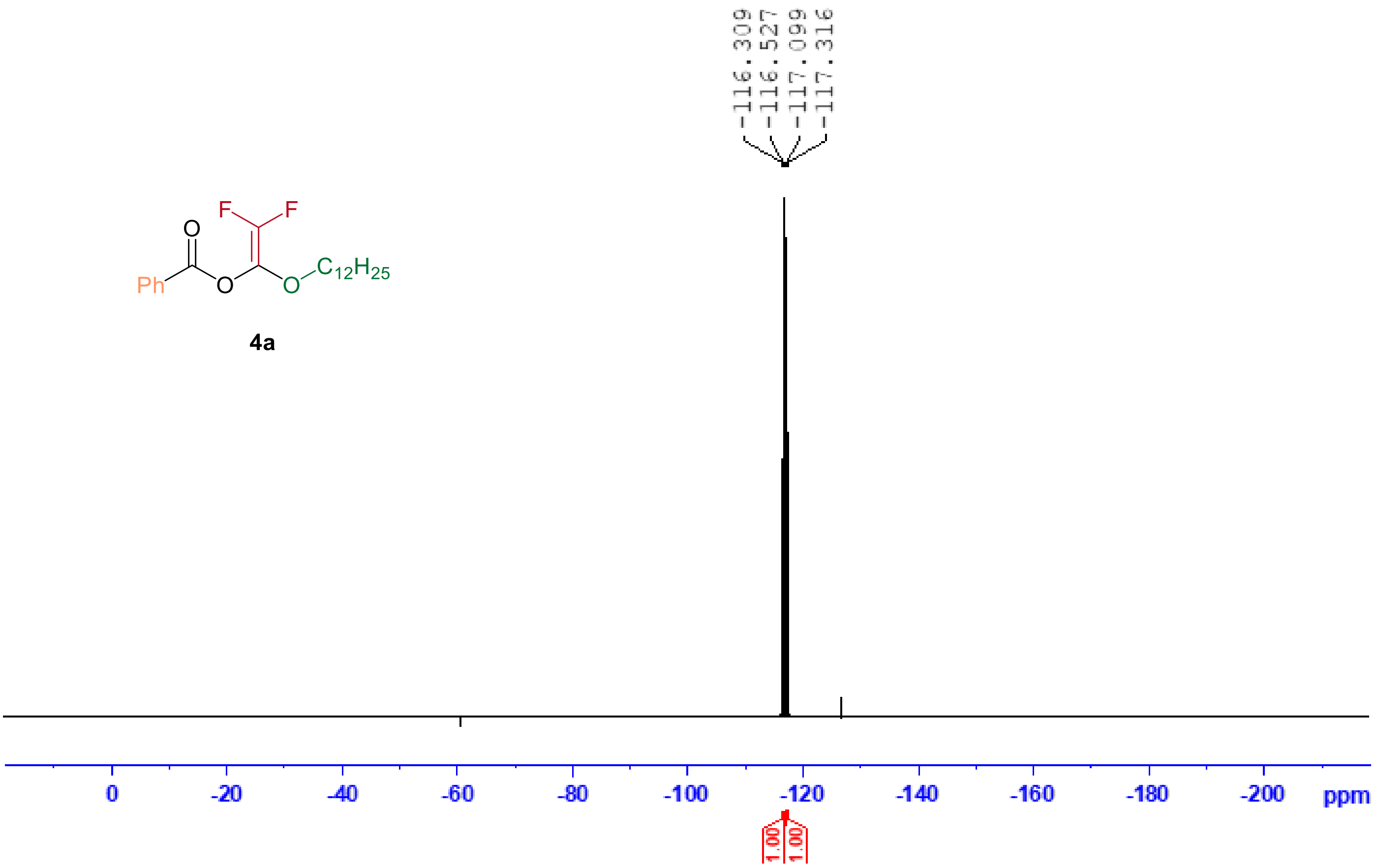

-116.309
-116.527
-117.099
-117.316
Ph
O
O
F
F
O
$C_{12}H_{25}$
4a
0
-20
-40
-60
-80
-100
-120
-140
-160
-180
-200
ppm
1.00
1.00

^{1}H NMR of **4b**

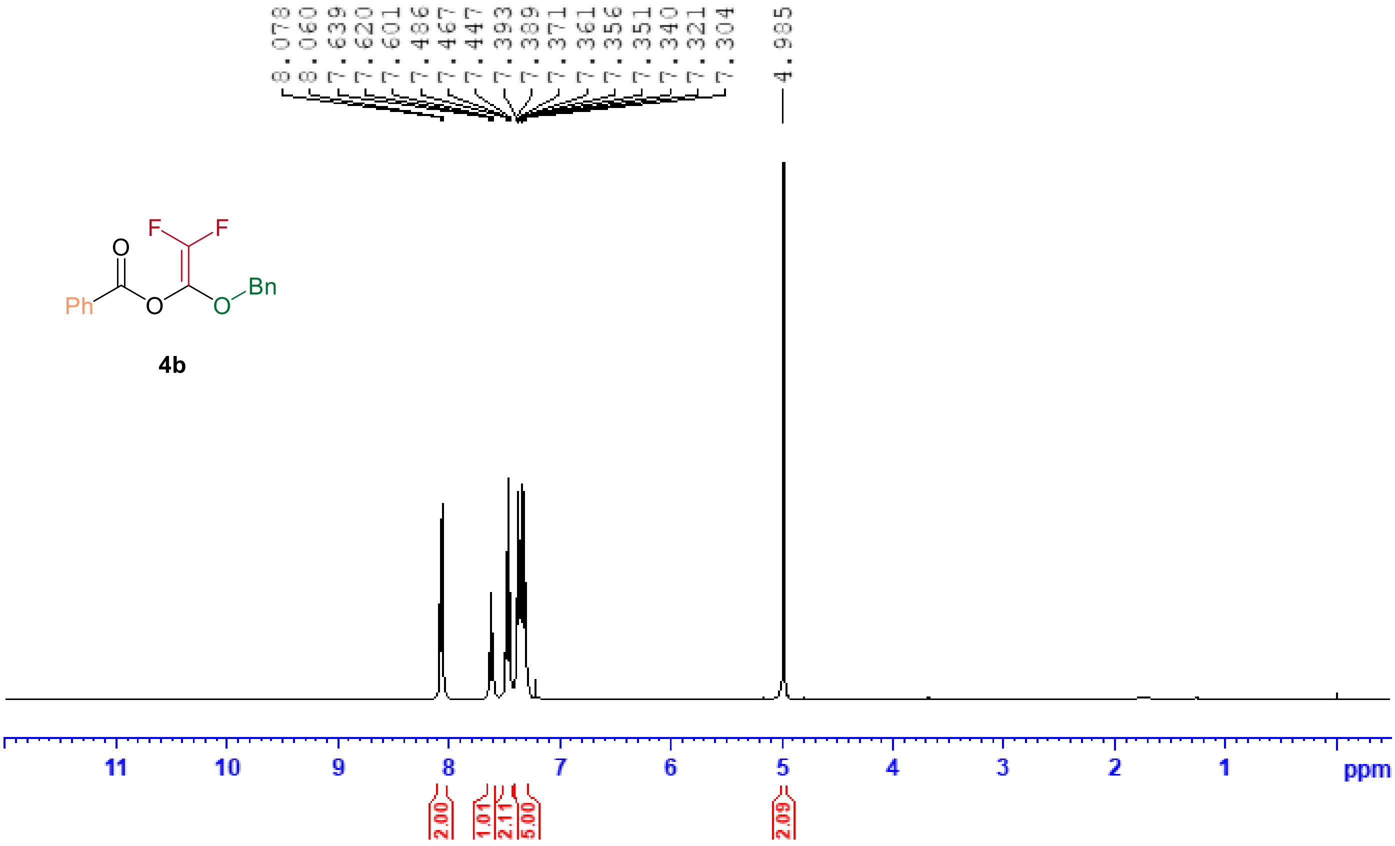

^{13}C NMR of **4b**

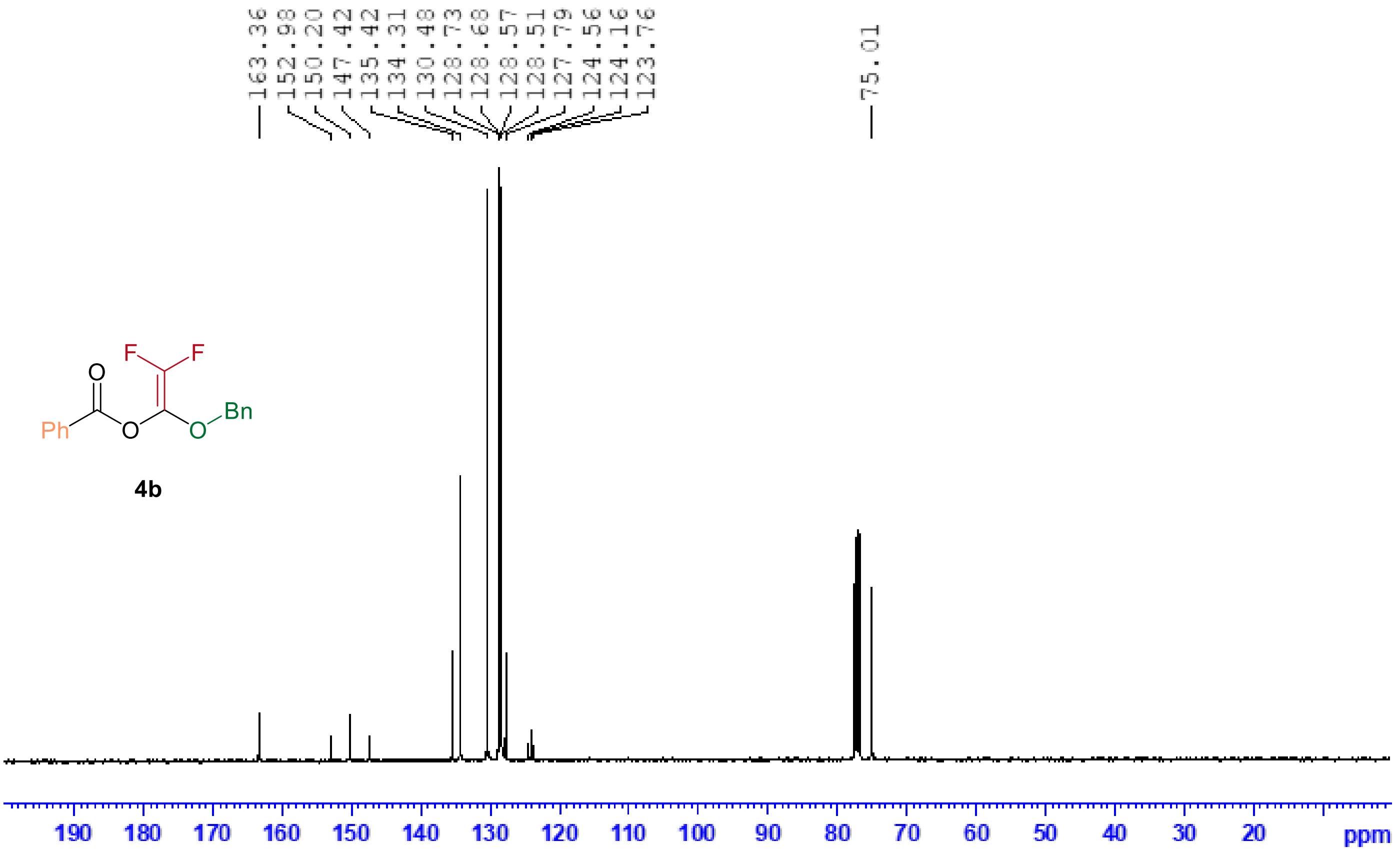

^{19}F NMR of **4b**

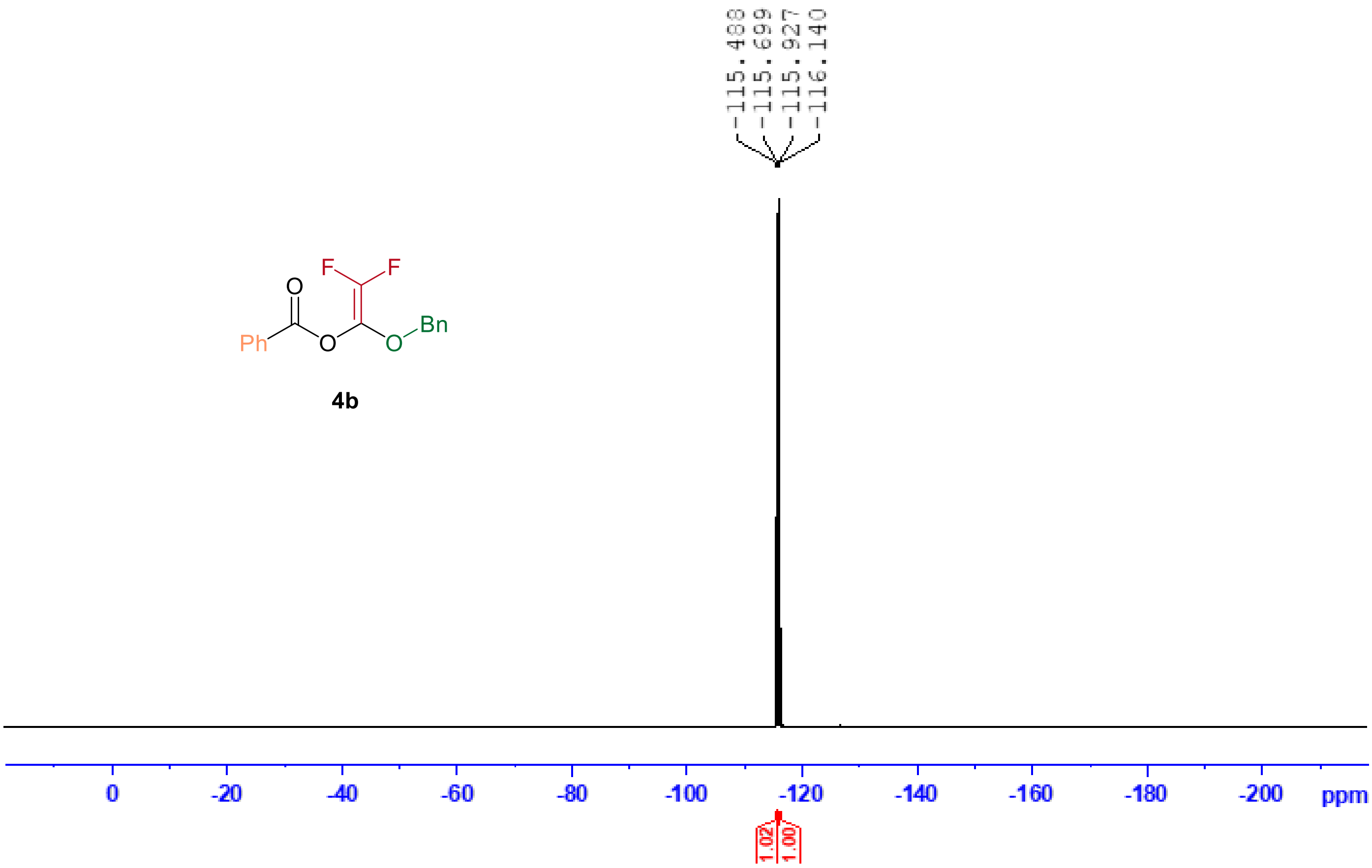

-115.488
-115.699
-115.927
-116.140
F
F
O
Ph
O
O
Bn
4b
0
-20
-40
-60
-80
-100
-120
-140
-160
-180
-200
ppm
1.02
1.00

^{1}H NMR of **4c**

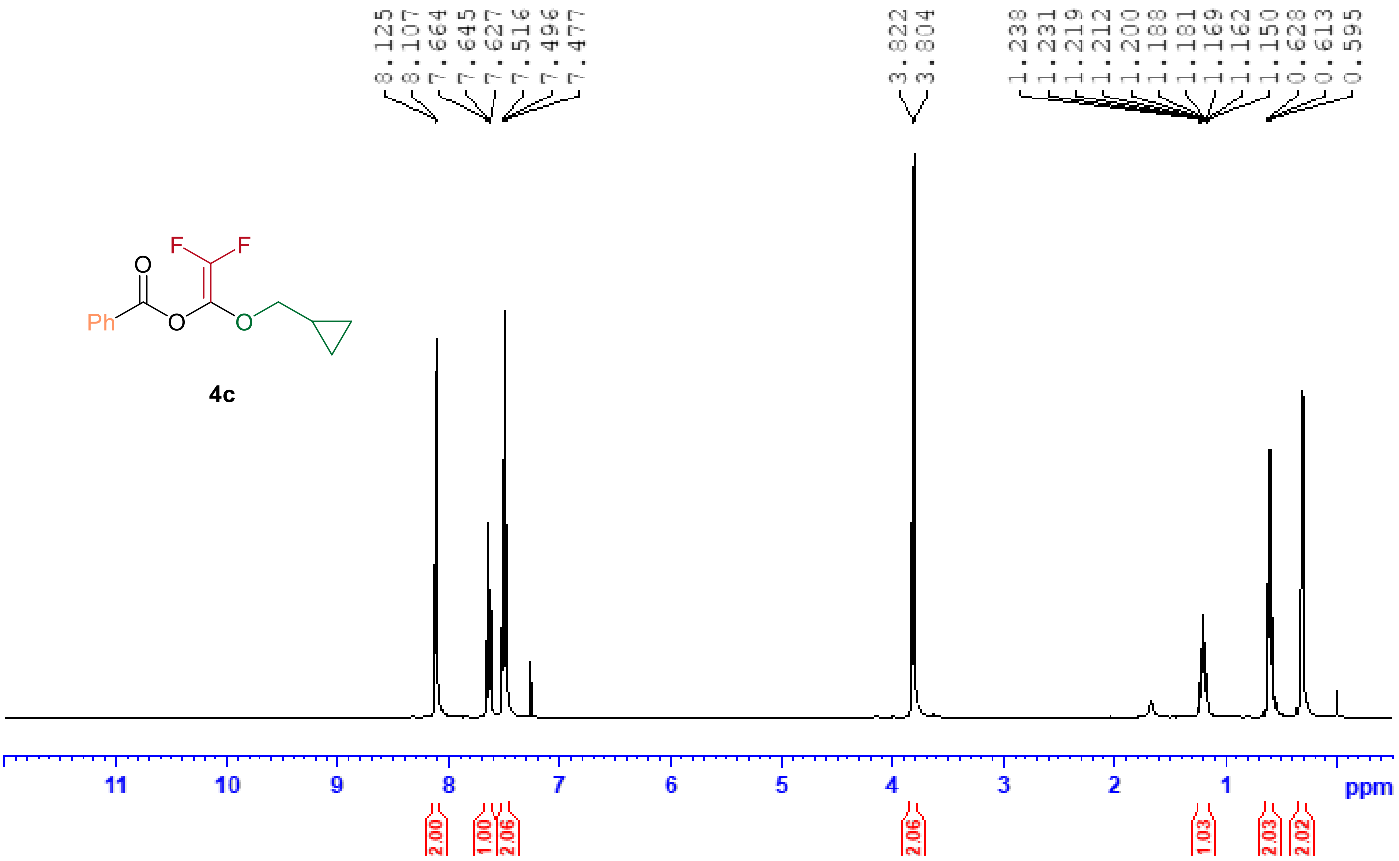

^{13}C NMR of **4c**

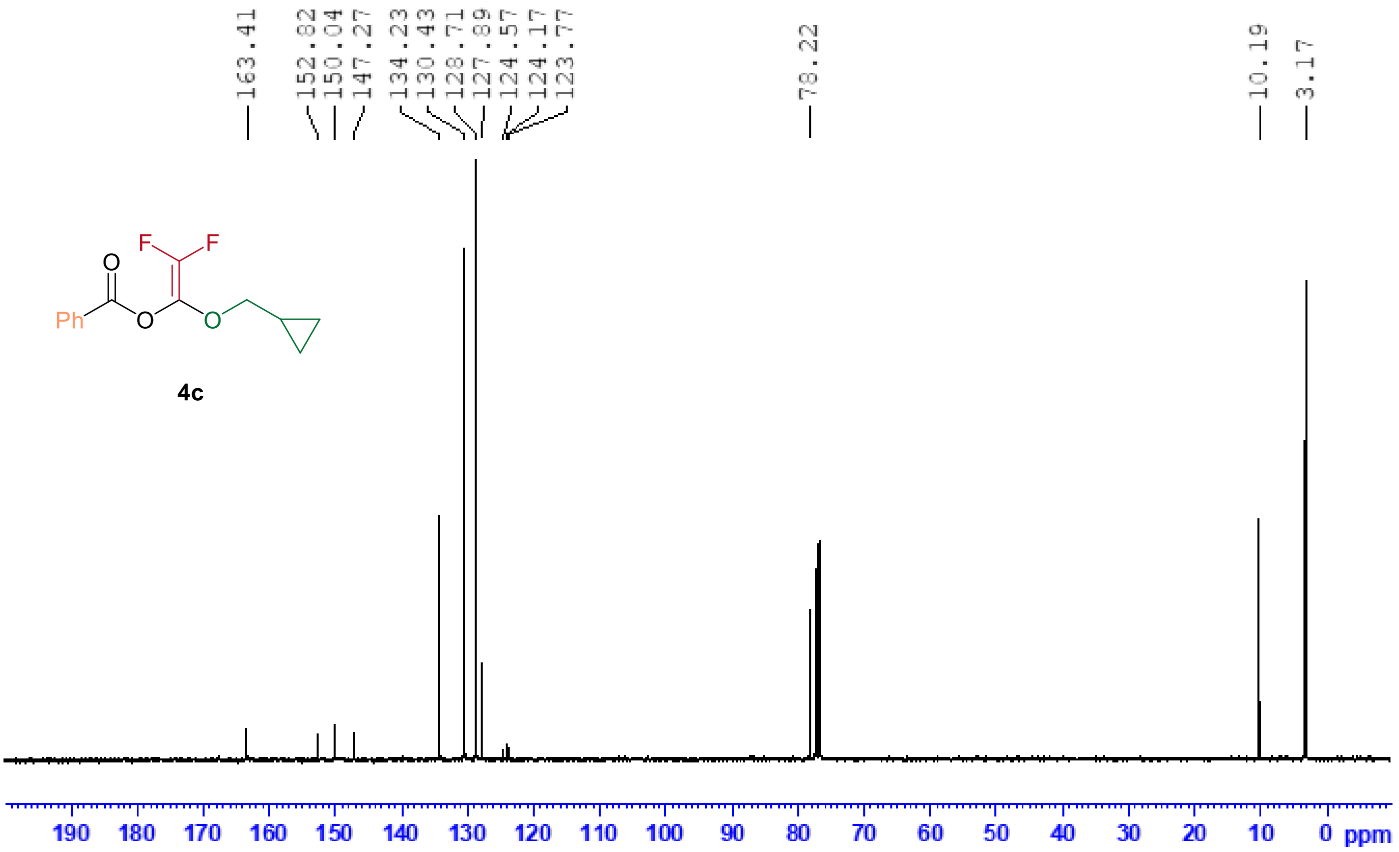

^{19}F NMR of **4c**

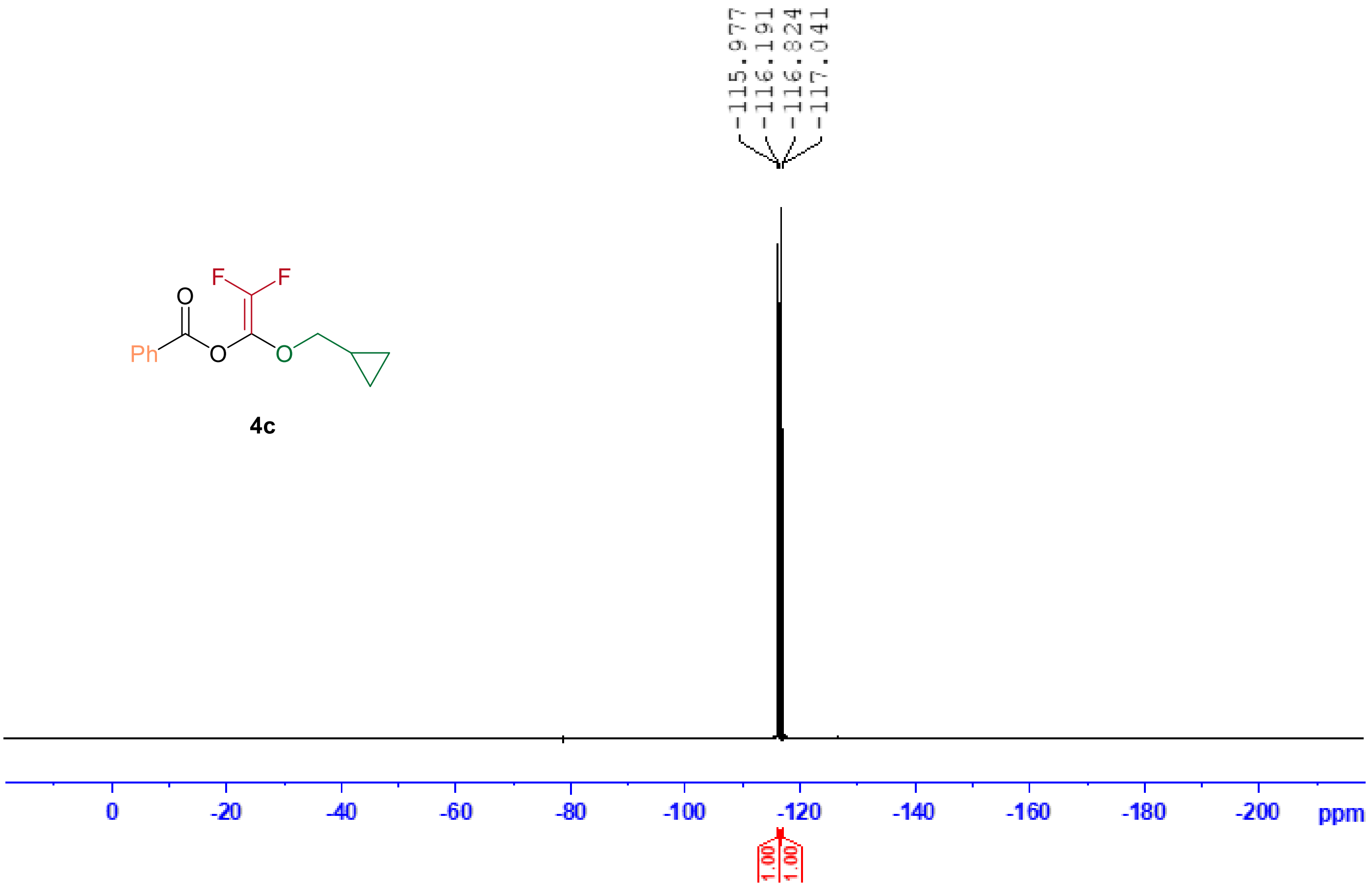
4c
-115.977
-116.191
-116.824
-117.041
0
-20
-40
-60
-80
-100
-120
-140
-160
-180
-200
ppm
1.00
1.00

^{1}H NMR of **4d**

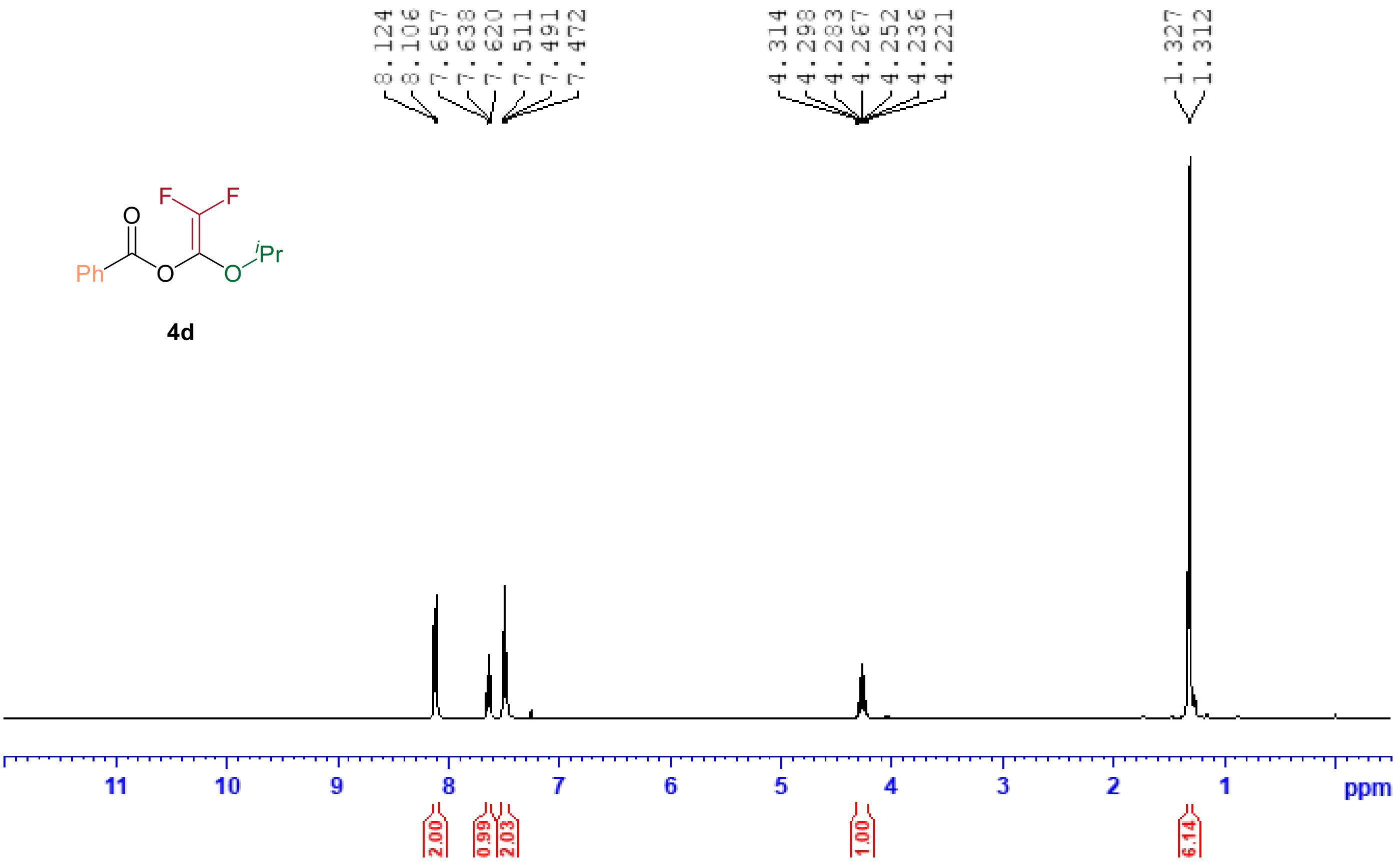

^{13}C NMR of **4d**

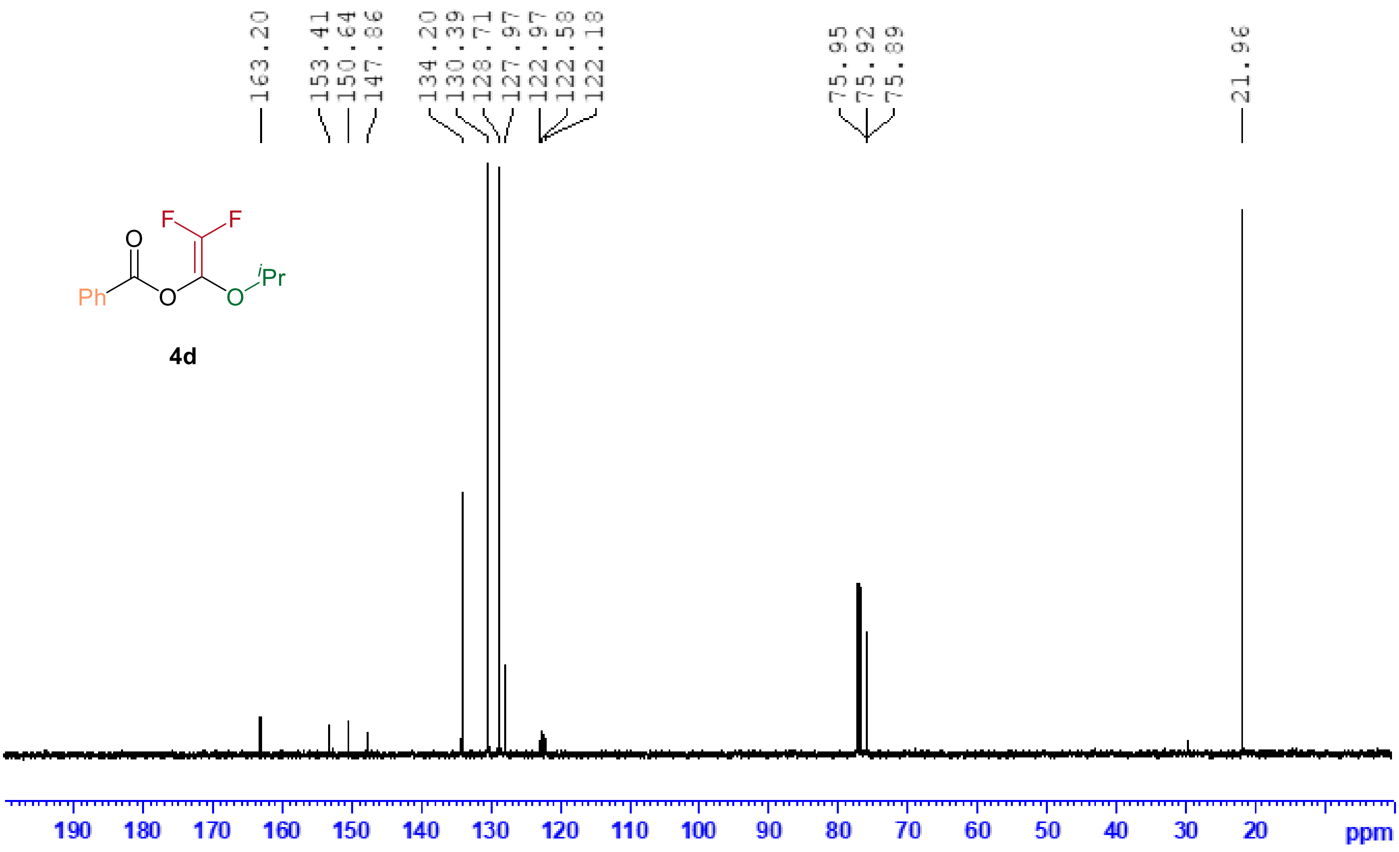

^{19}F NMR of **4d**

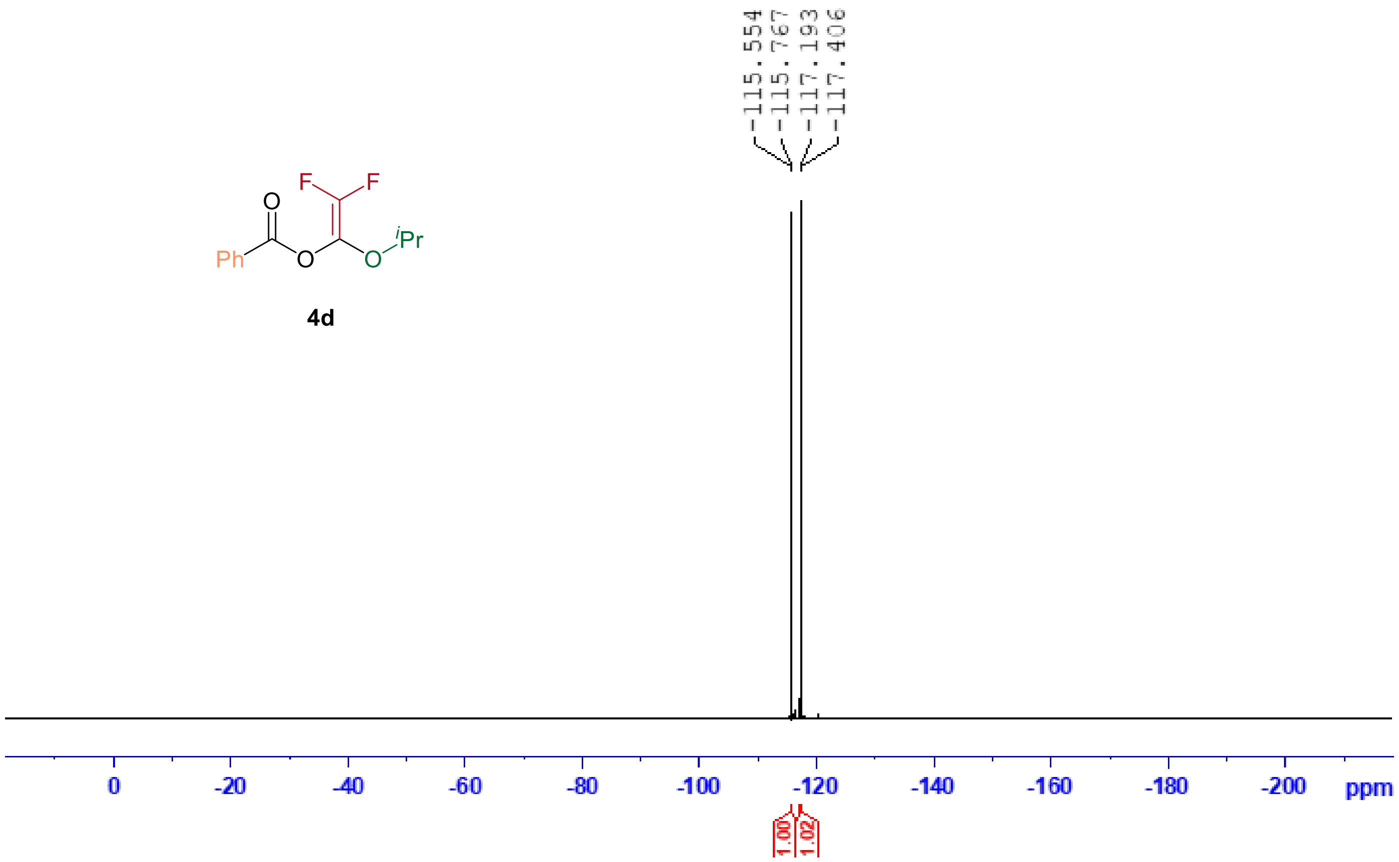

4d
-115.554
-115.767
-117.193
-117.406
0
-20
-40
-60
-80
-100
-120
-140
-160
-180
-200
ppm
1.00
1.02

^{1}H NMR of **4e**

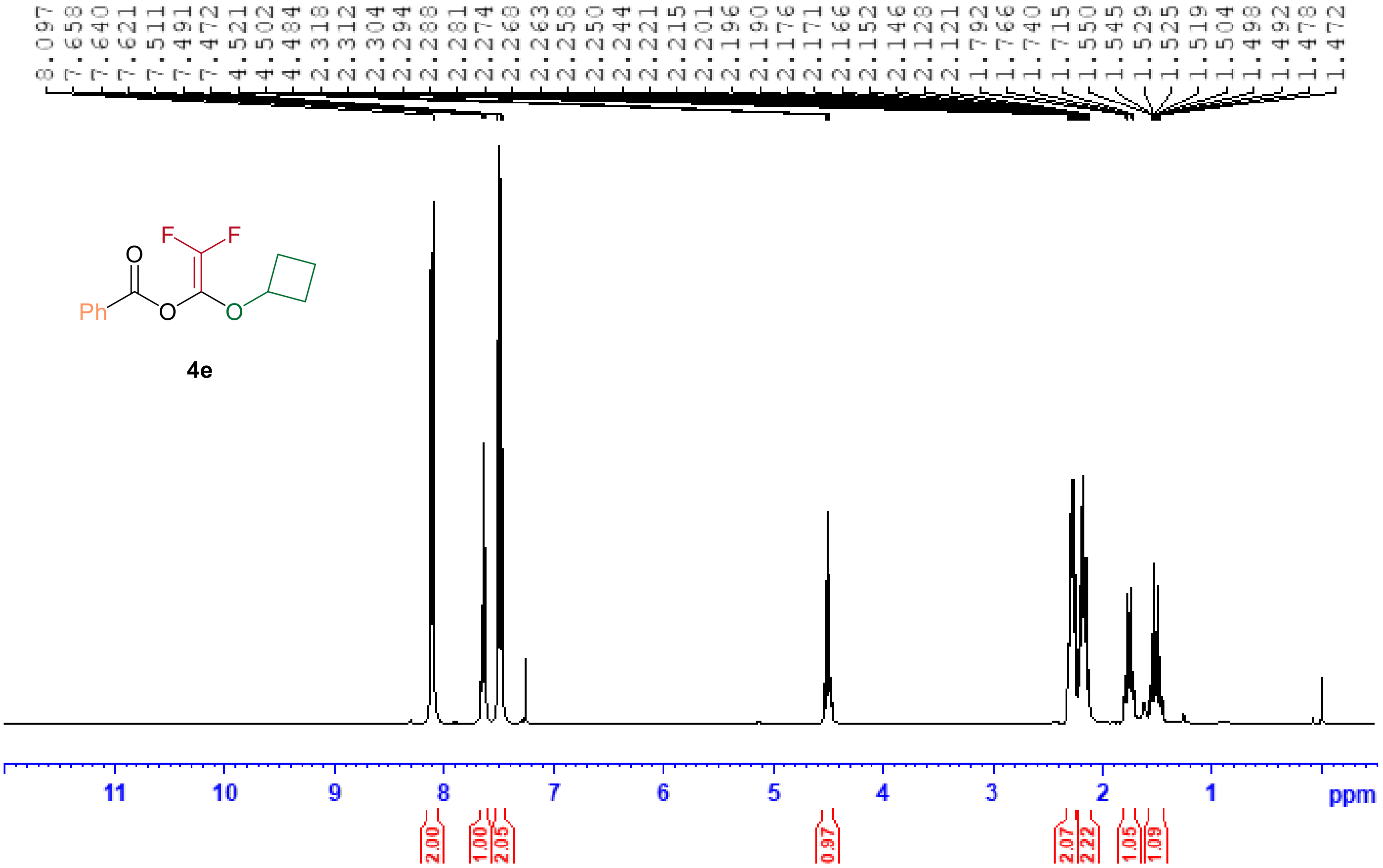

^{13}C NMR of **4e**

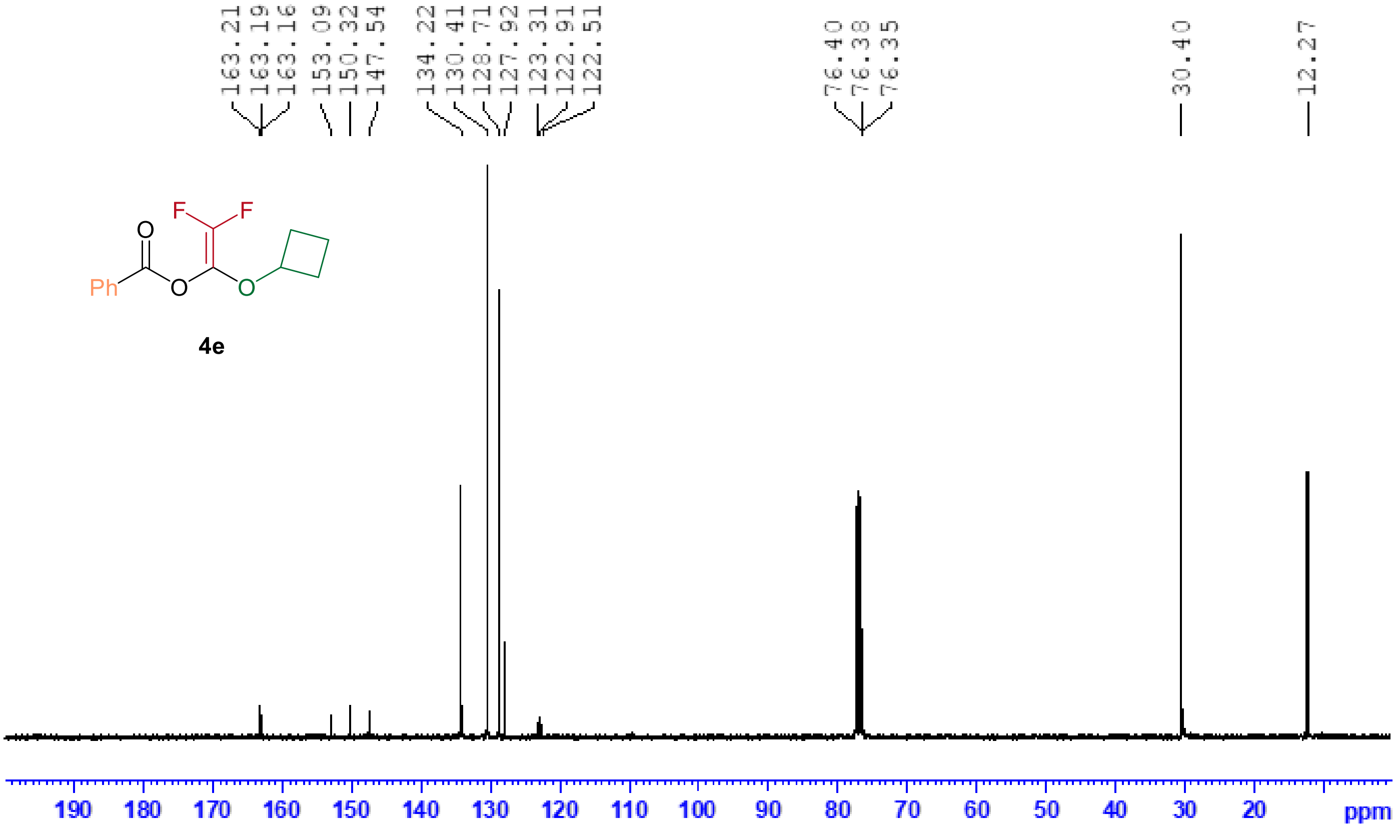

^{19}F NMR of **4e**

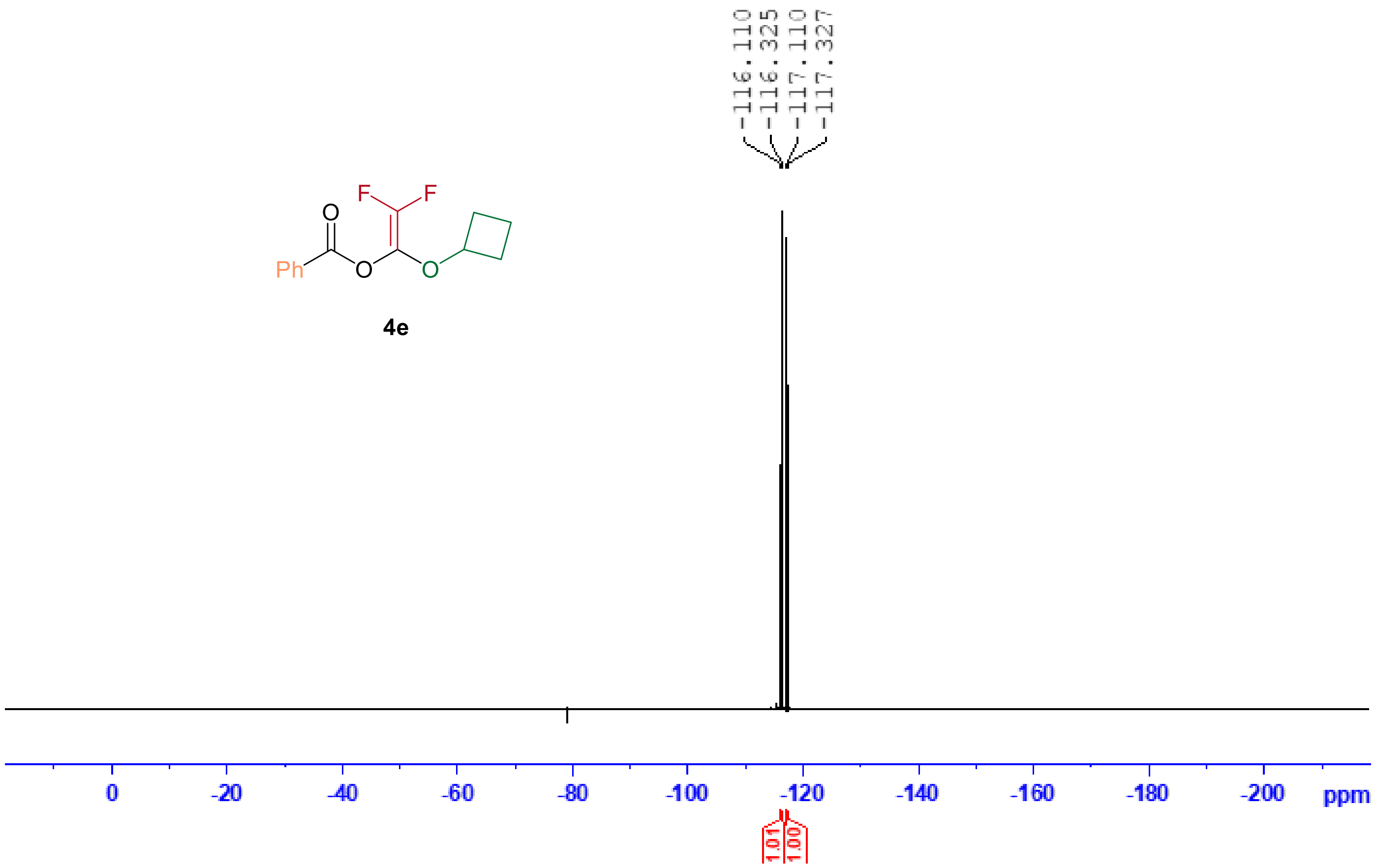

-116.110
-116.325
-117.110
-117.327
Ph
O
O
O
F
F
4e
0
-20
-40
-60
-80
-100
-120
-140
-160
-180
-200
ppm
1.01
1.00

^{1}H NMR of **4f**

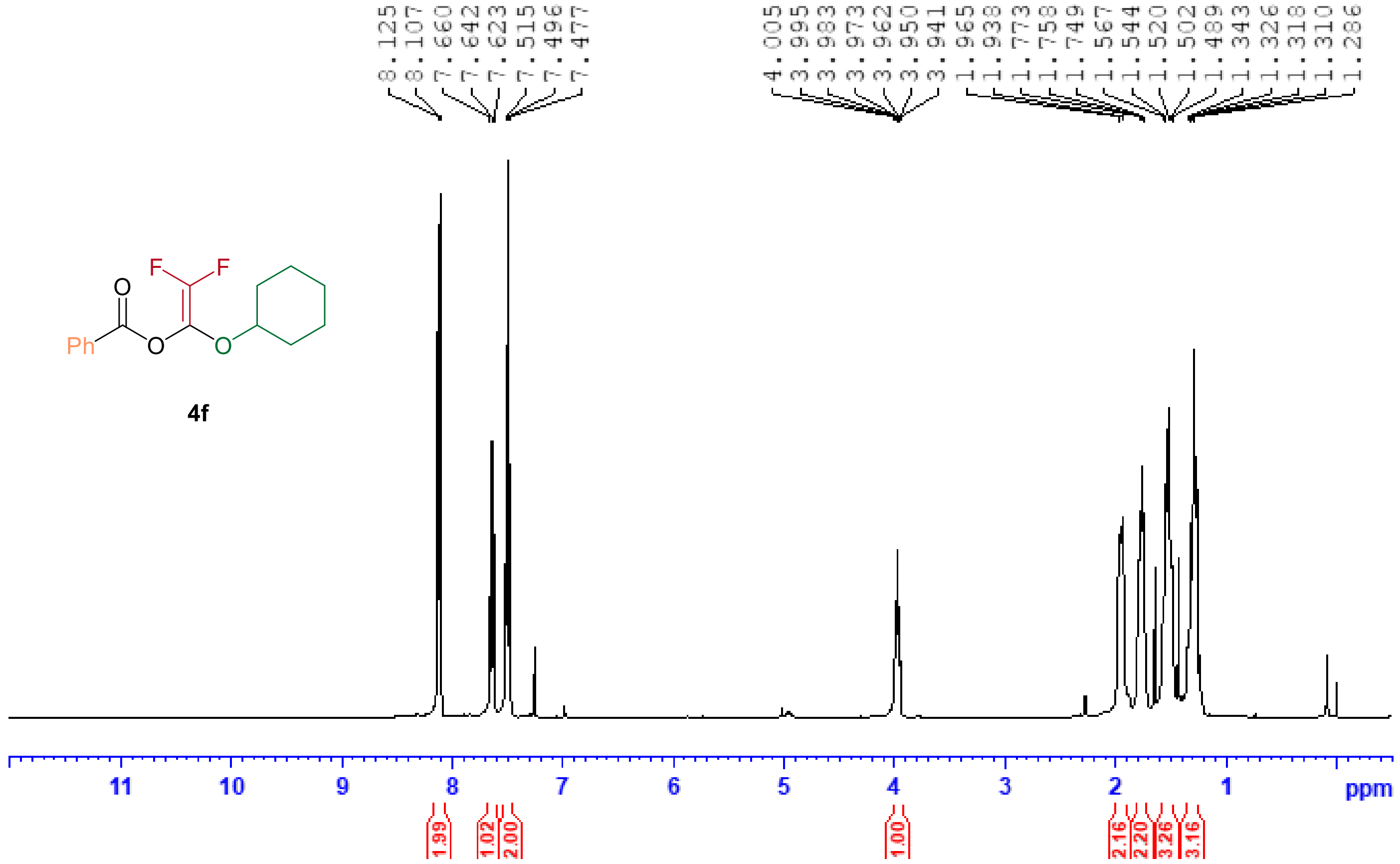

^{13}C NMR of **4f**

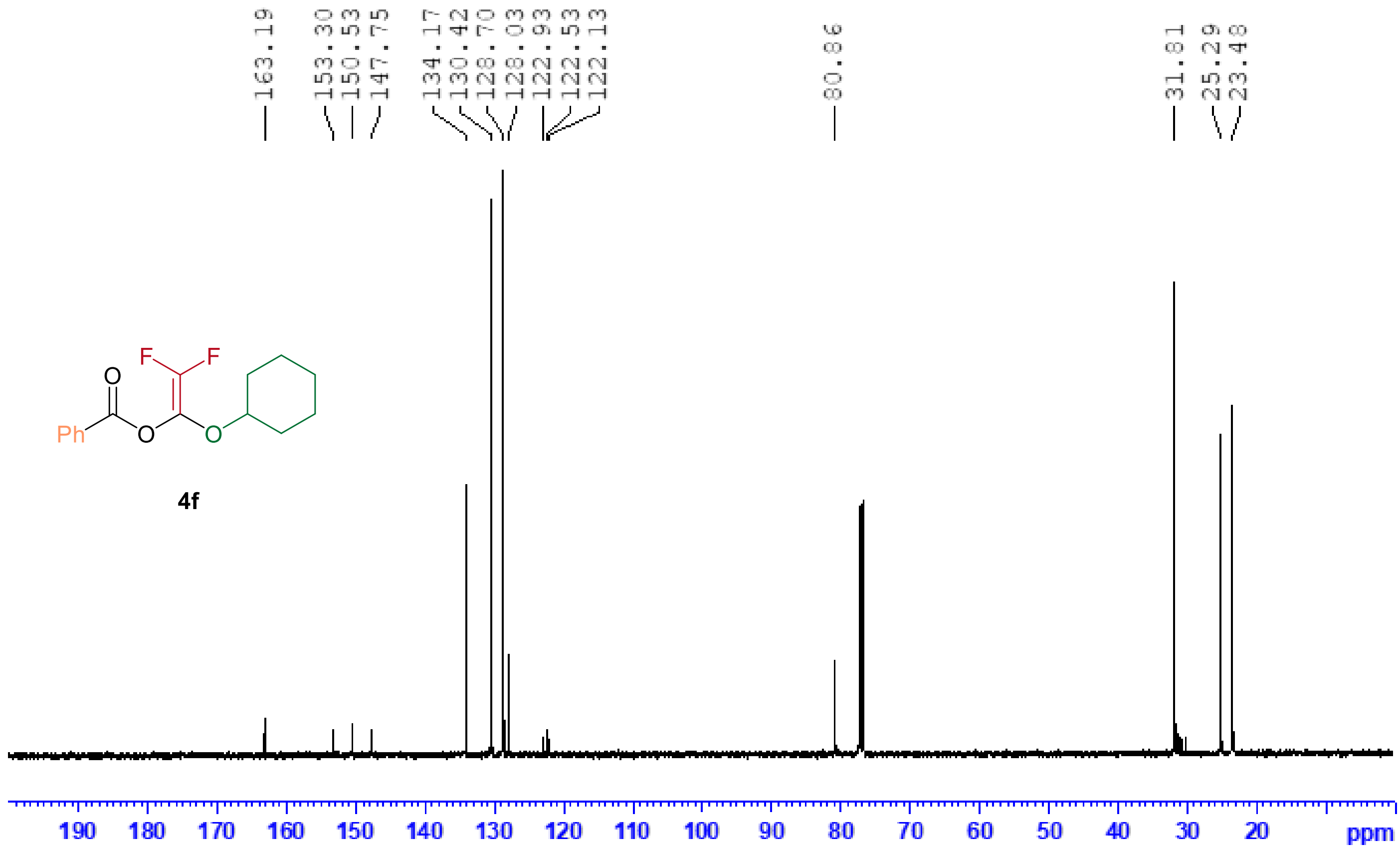

^{19}F NMR of **4f**

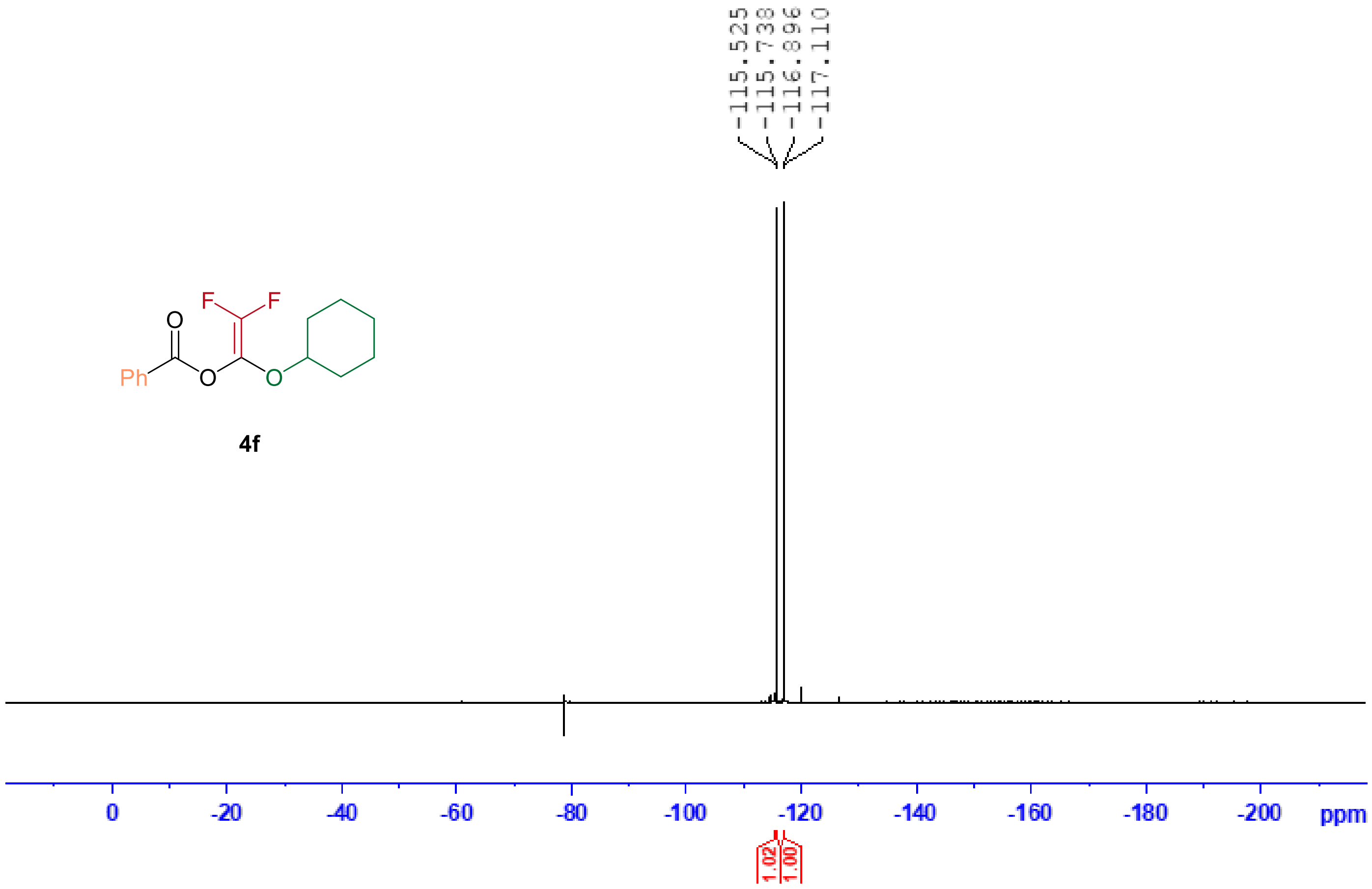

-115.525
-115.738
-116.896
-117.110
0
-20
-40
-60
-80
-100
-120
-140
-160
-180
-200
ppm
1.02
1.00

^{1}H NMR of **4g**

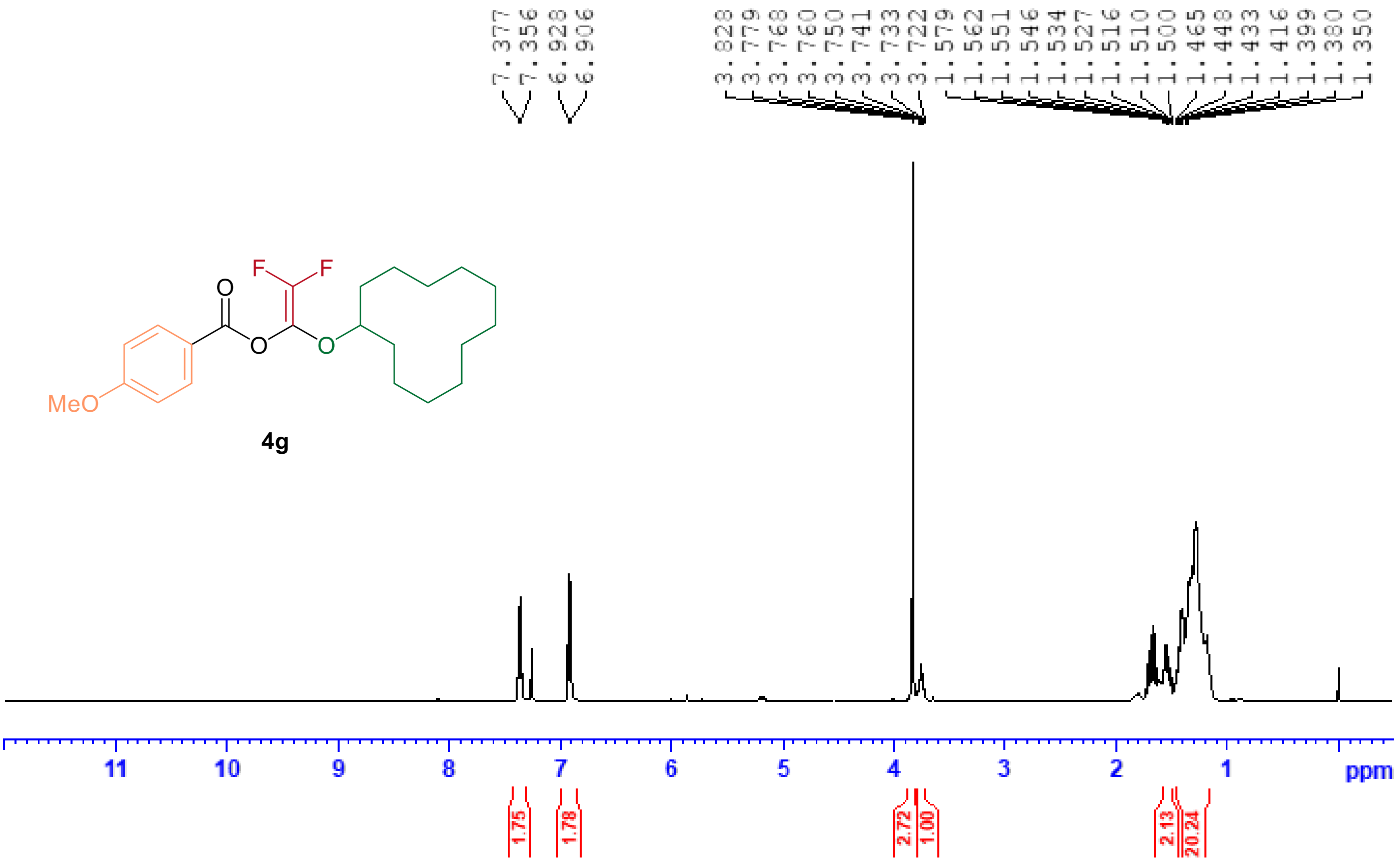

^{13}C NMR of **4g**

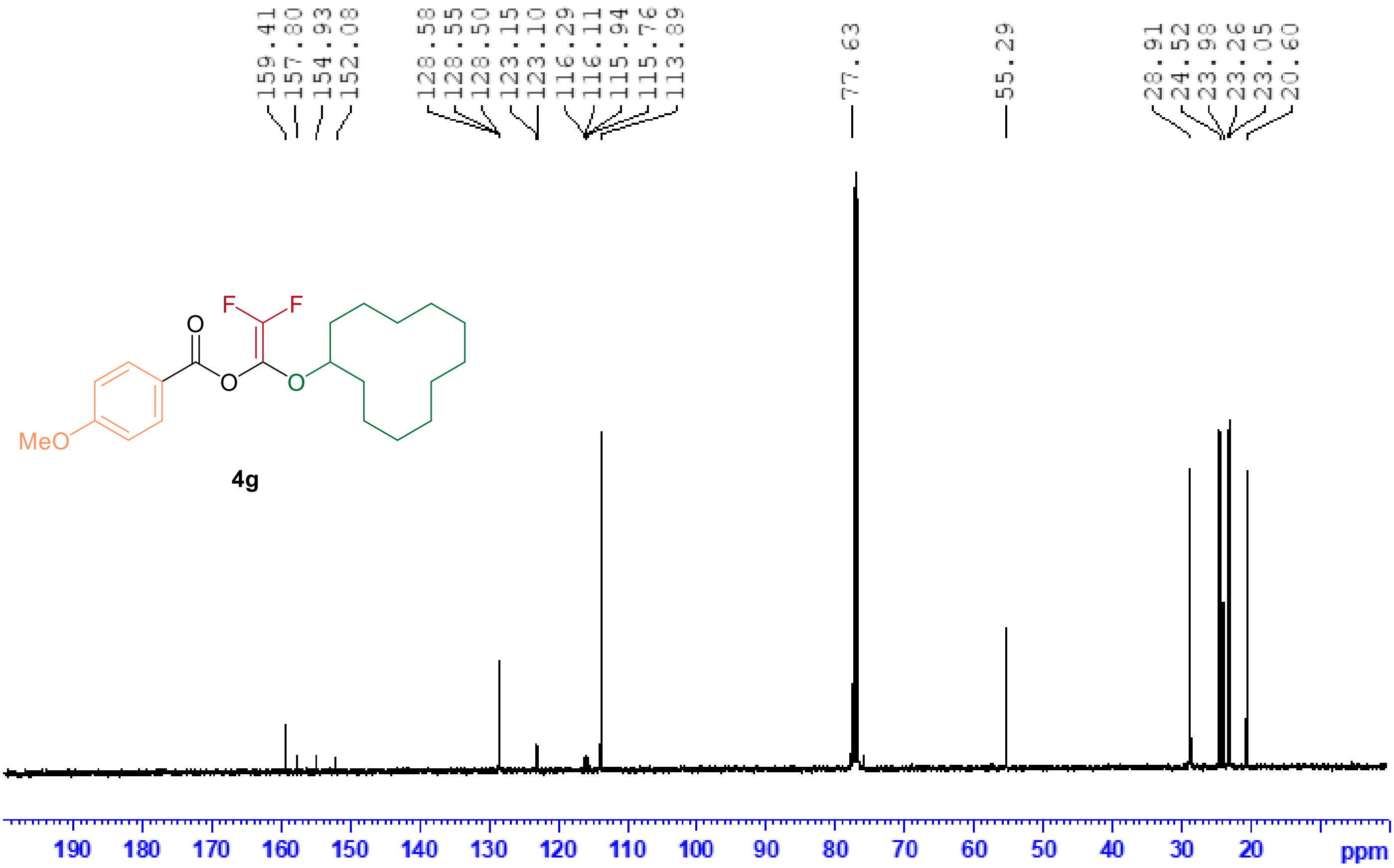

^{19}F NMR of **4g**

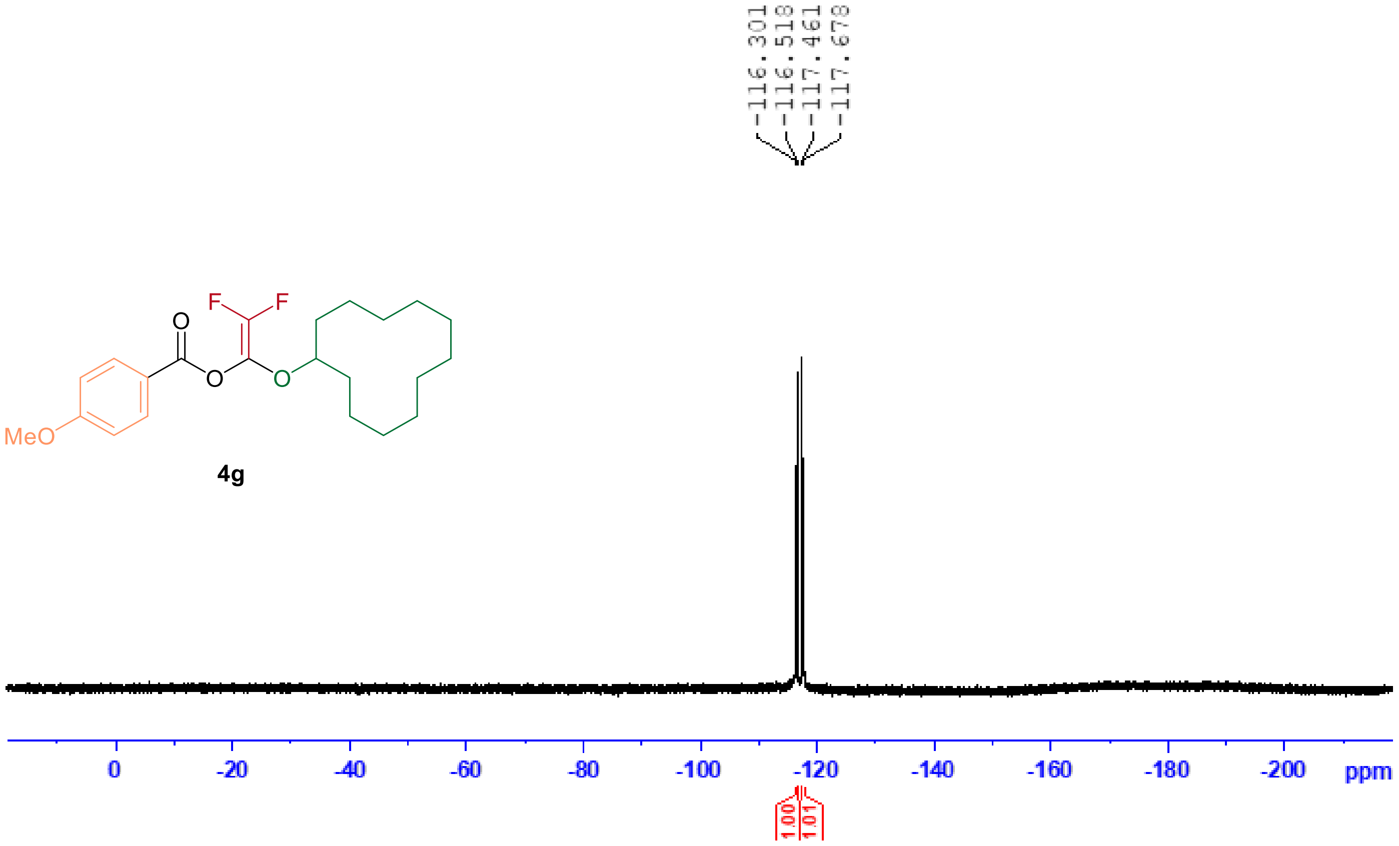
-116.301
-116.518
-117.461
-117.678
F
F
O
O
O
MeO
4g
0
-20
-40
-60
-80
-100
-120
-140
-160
-180
-200
ppm
1.00
1.01

^{1}H NMR of **4h**

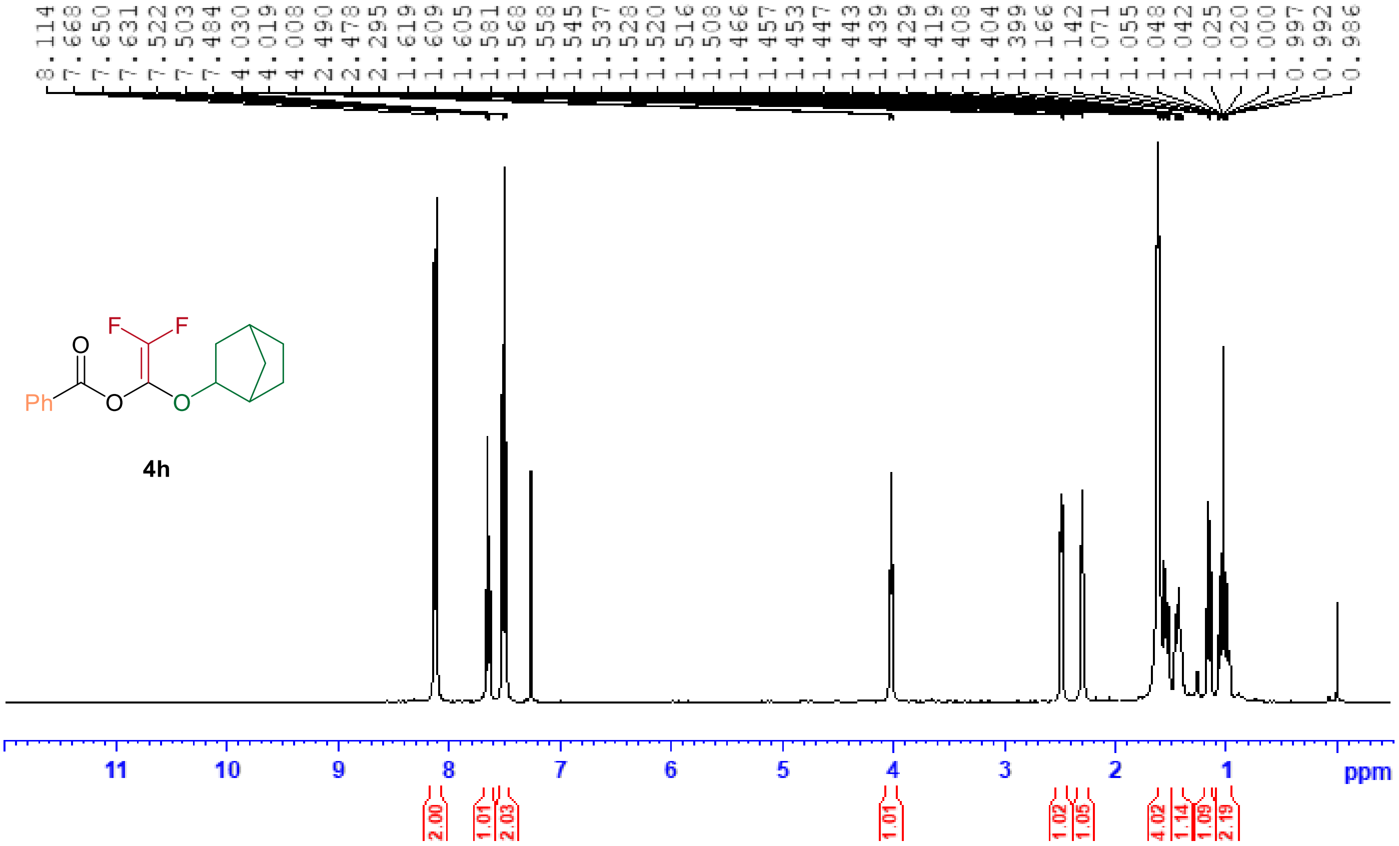

^{13}C NMR of **4h**

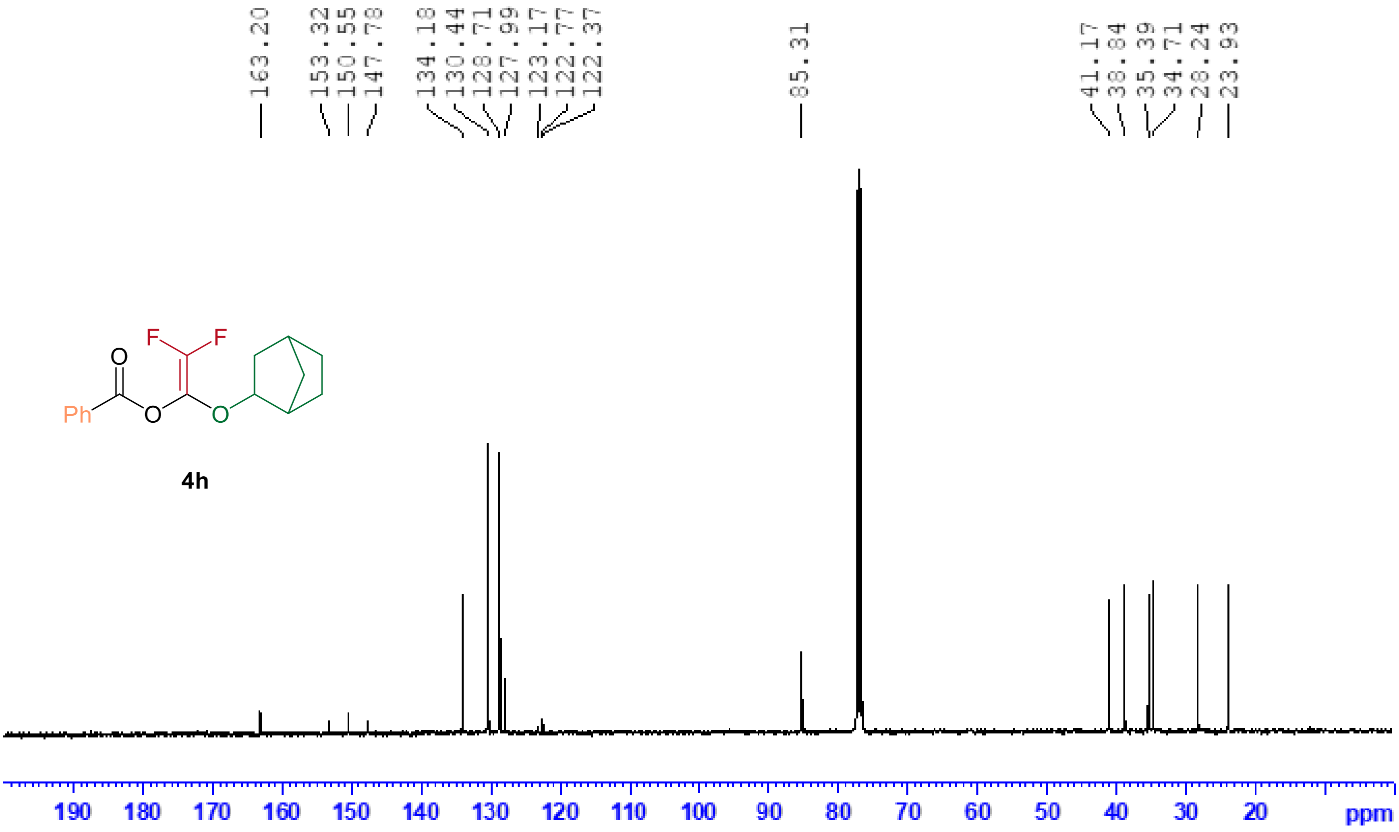

^{19}F NMR of **4h**

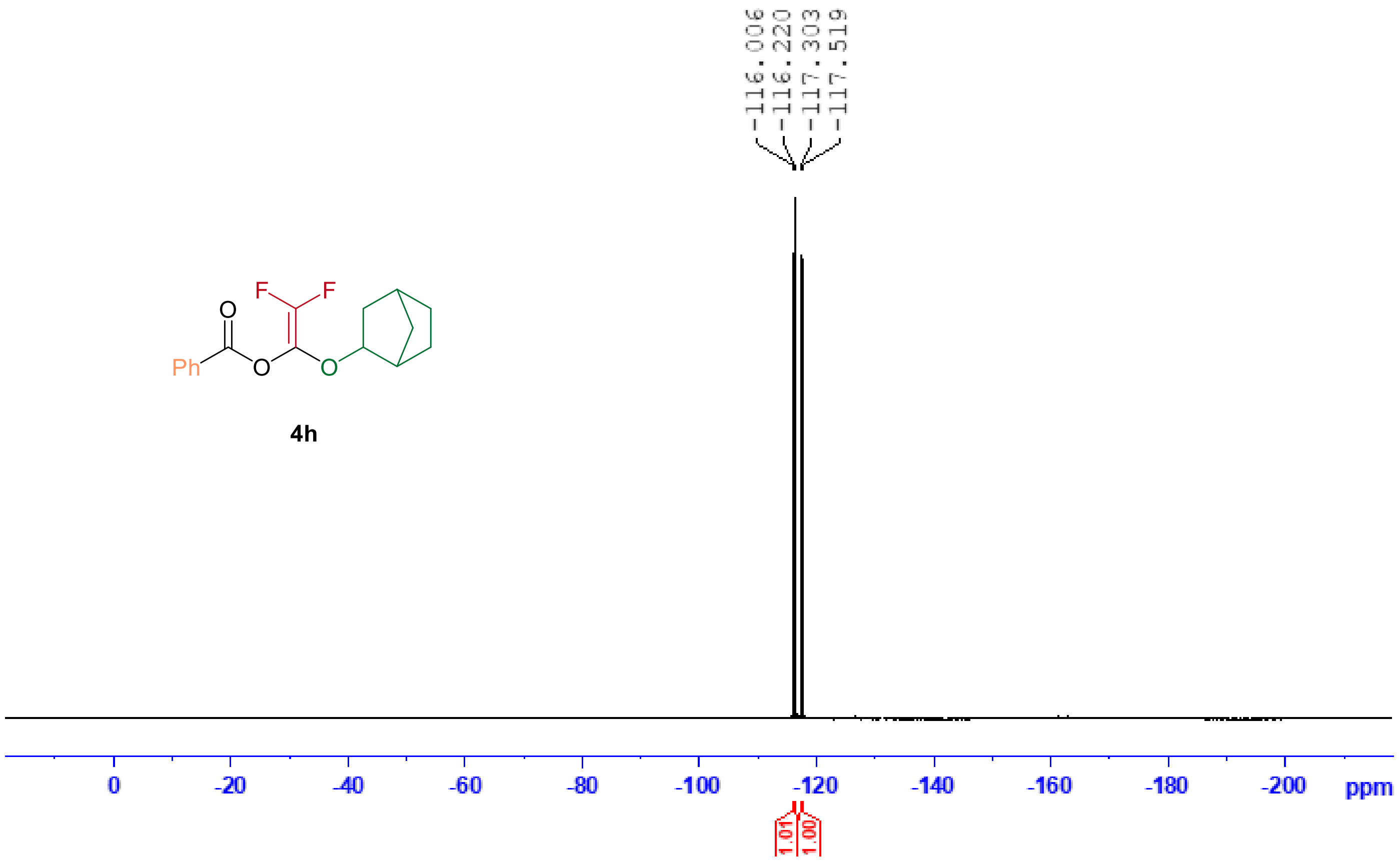

4h

^{1}H NMR of **4i**

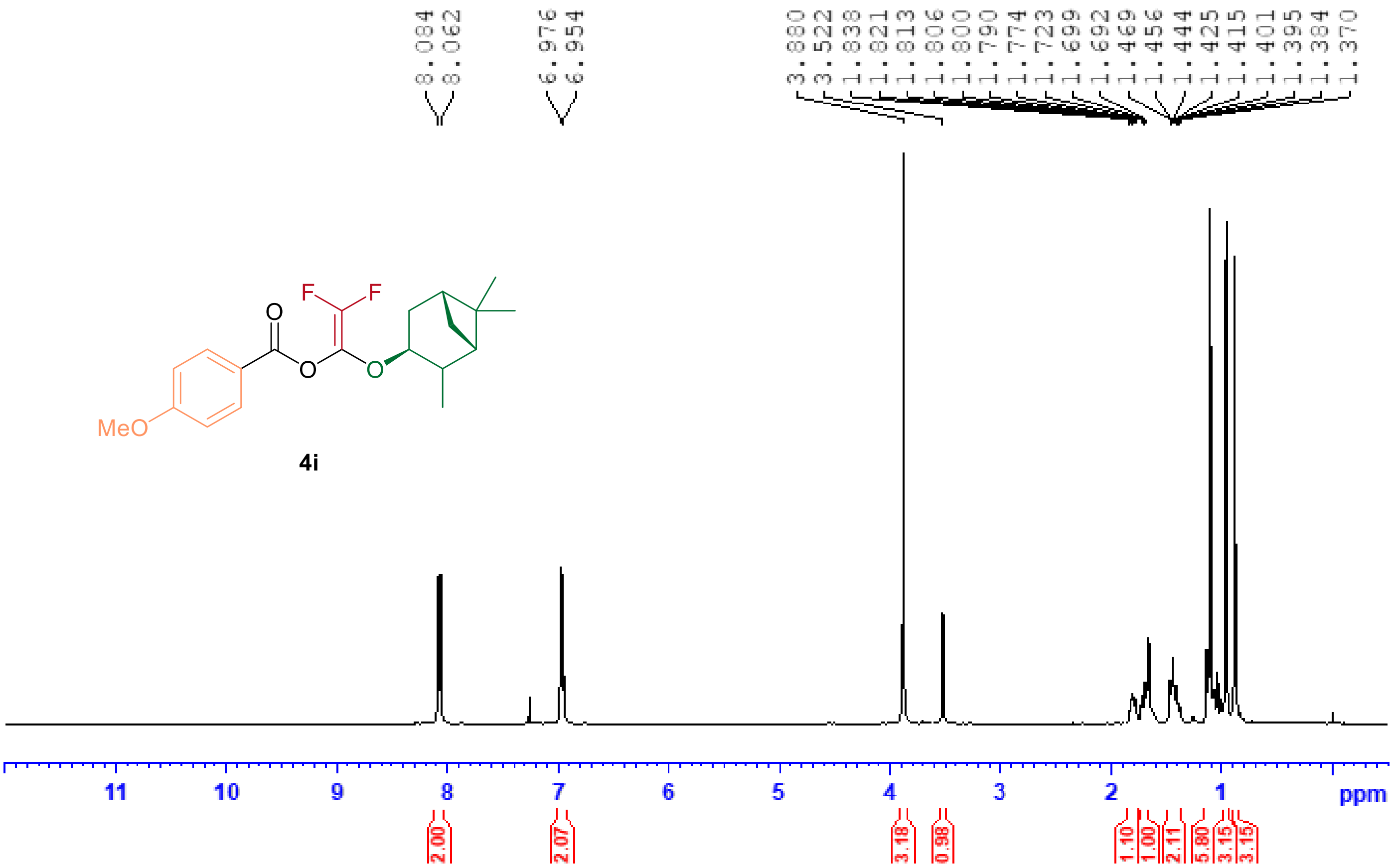

^{13}C NMR of **4i**

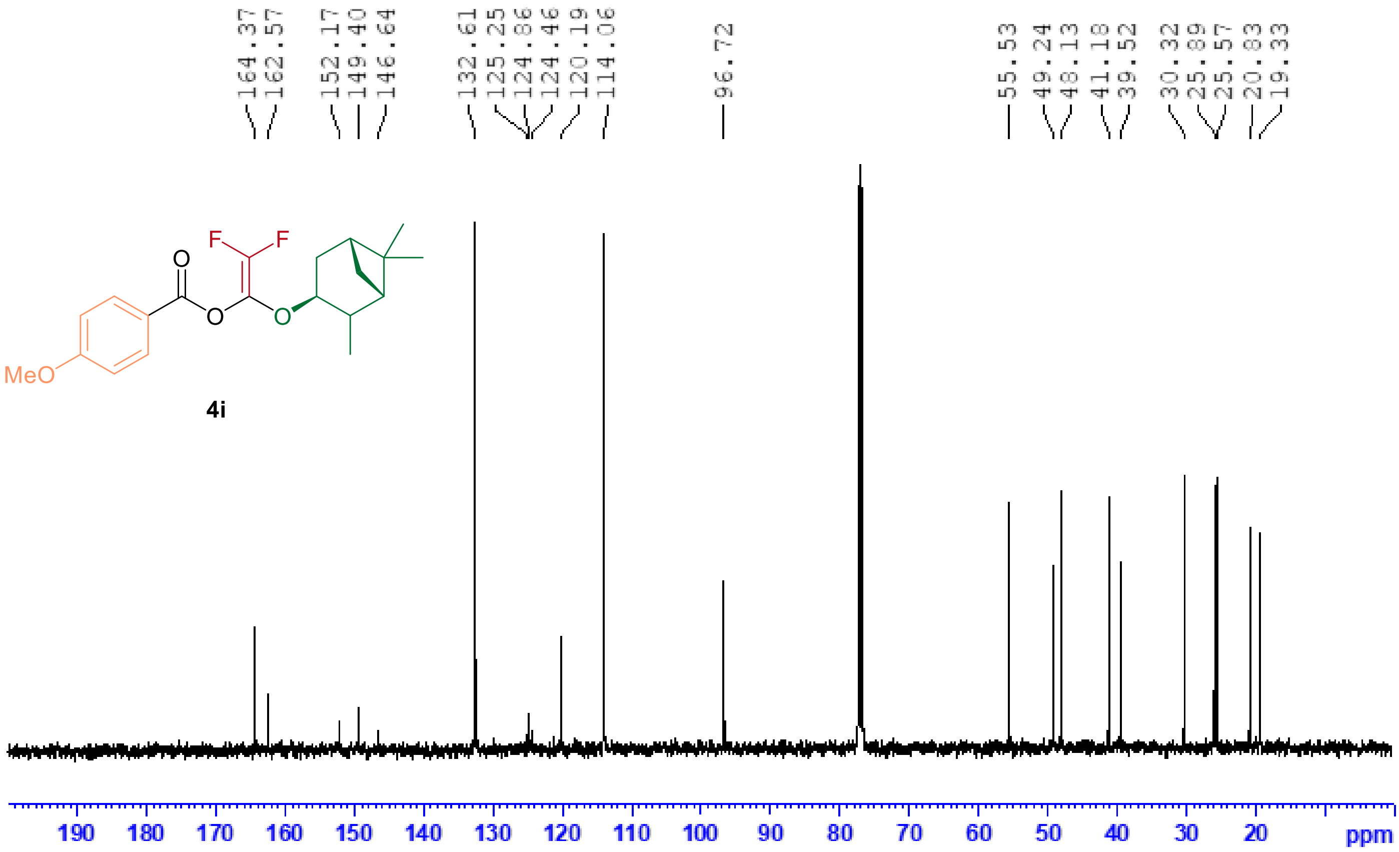

^{19}F NMR of **4i**

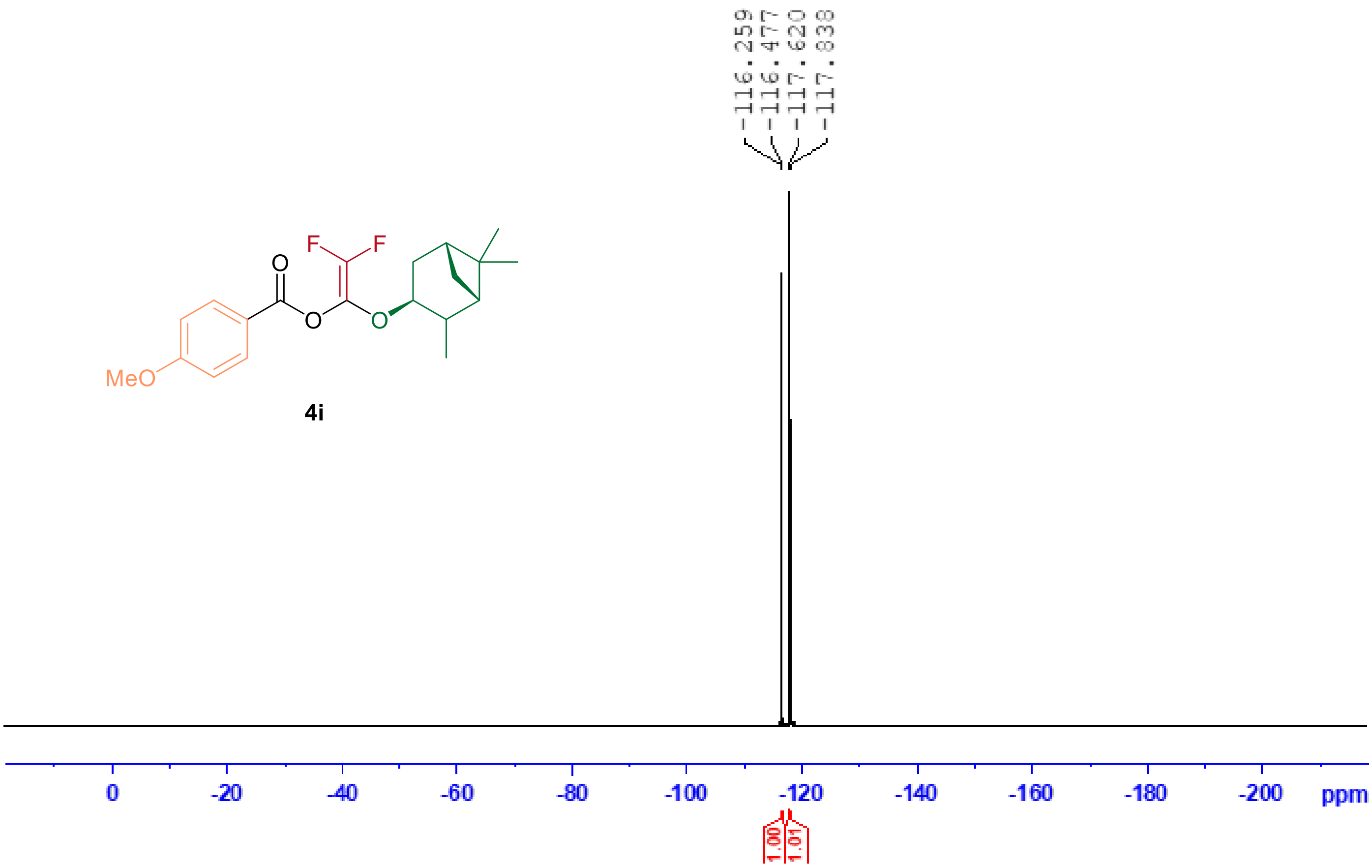

MeO
O
O
F
F
O
4i
-116.259
-116.477
-117.620
-117.838
0
-20
-40
-60
-80
-100
-120
-140
-160
-180
-200
ppm
1.00
1.01

^{1}H NMR of **4j**

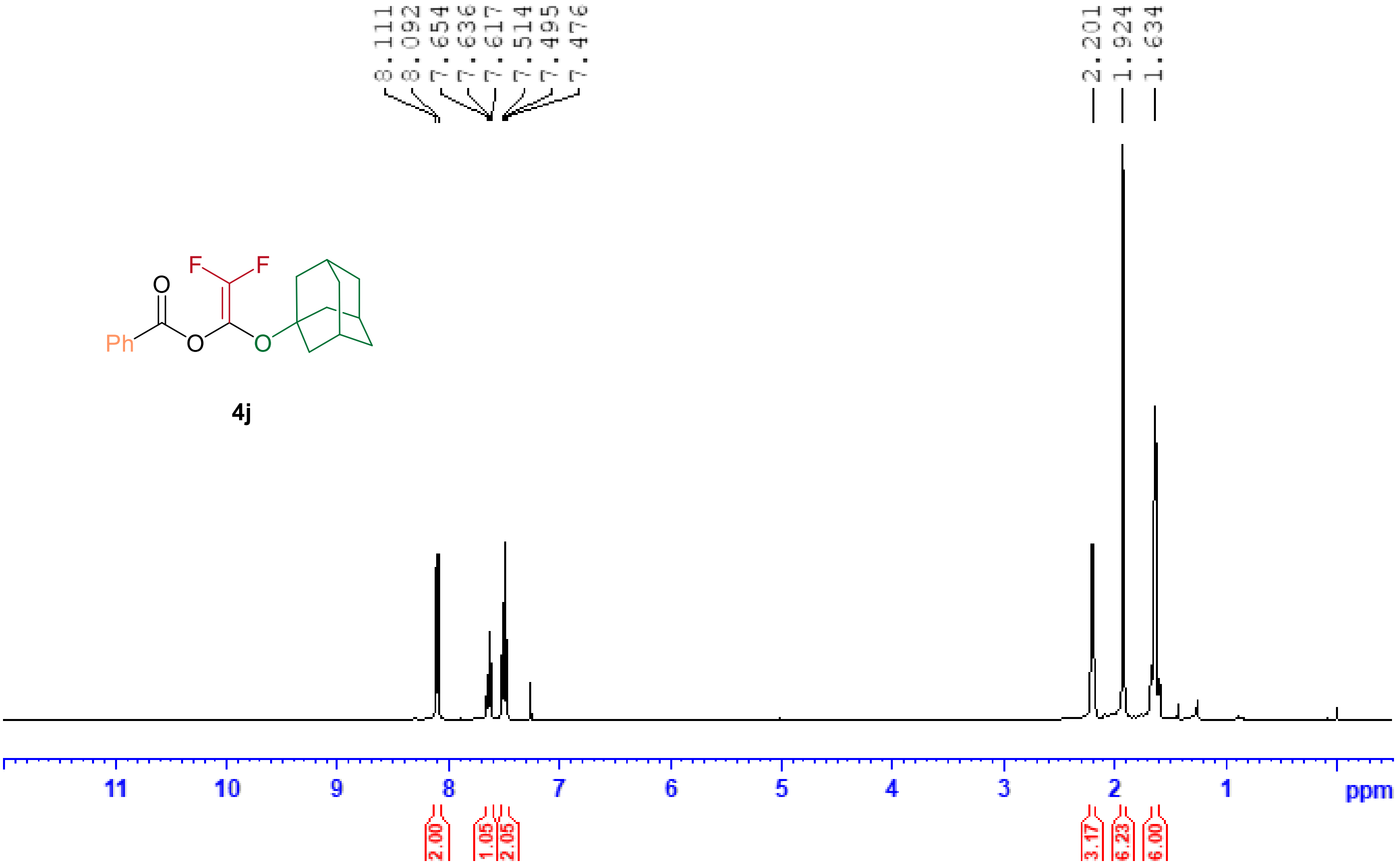

^{13}C NMR of **4j**

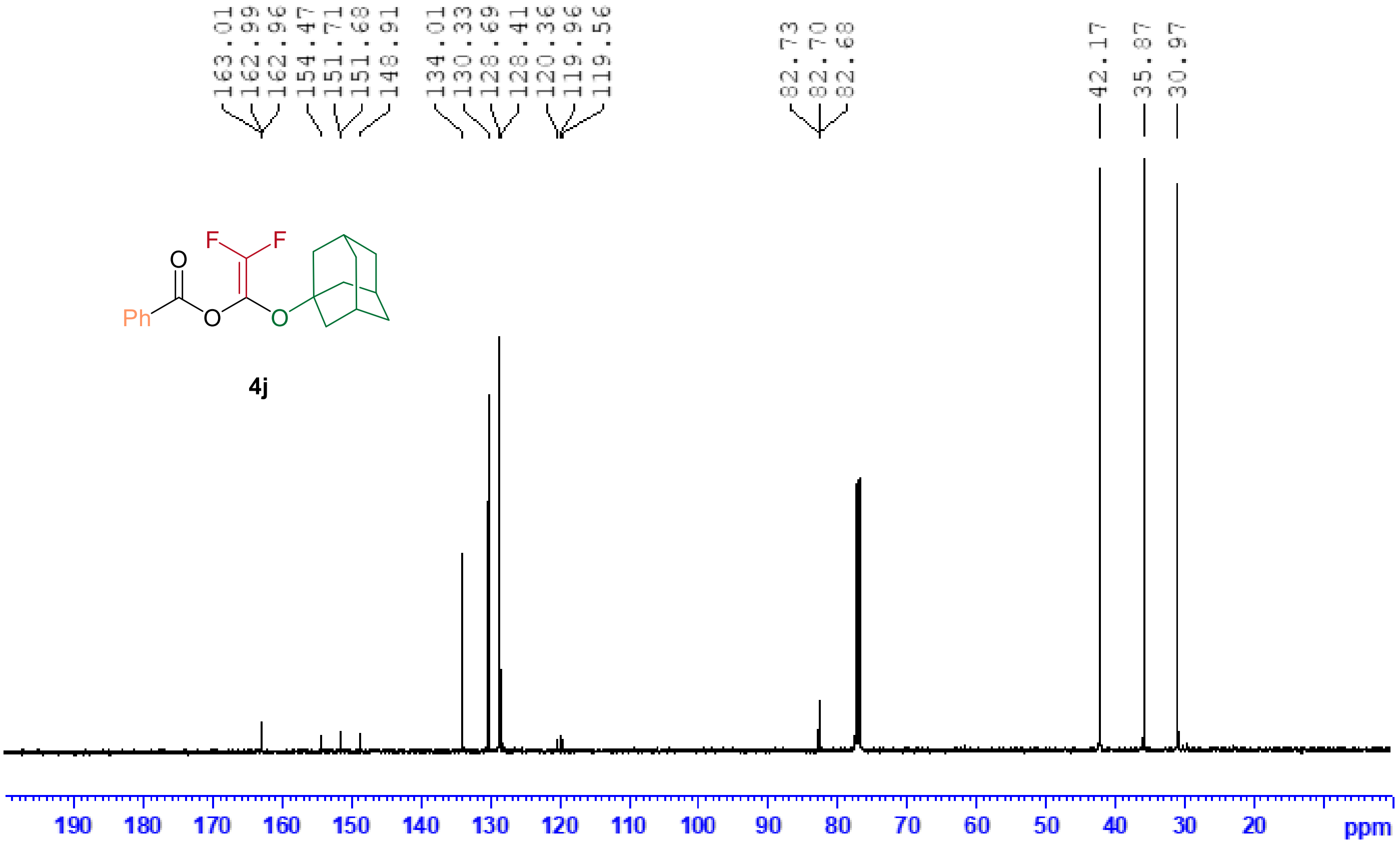

^{19}F NMR of **4j**

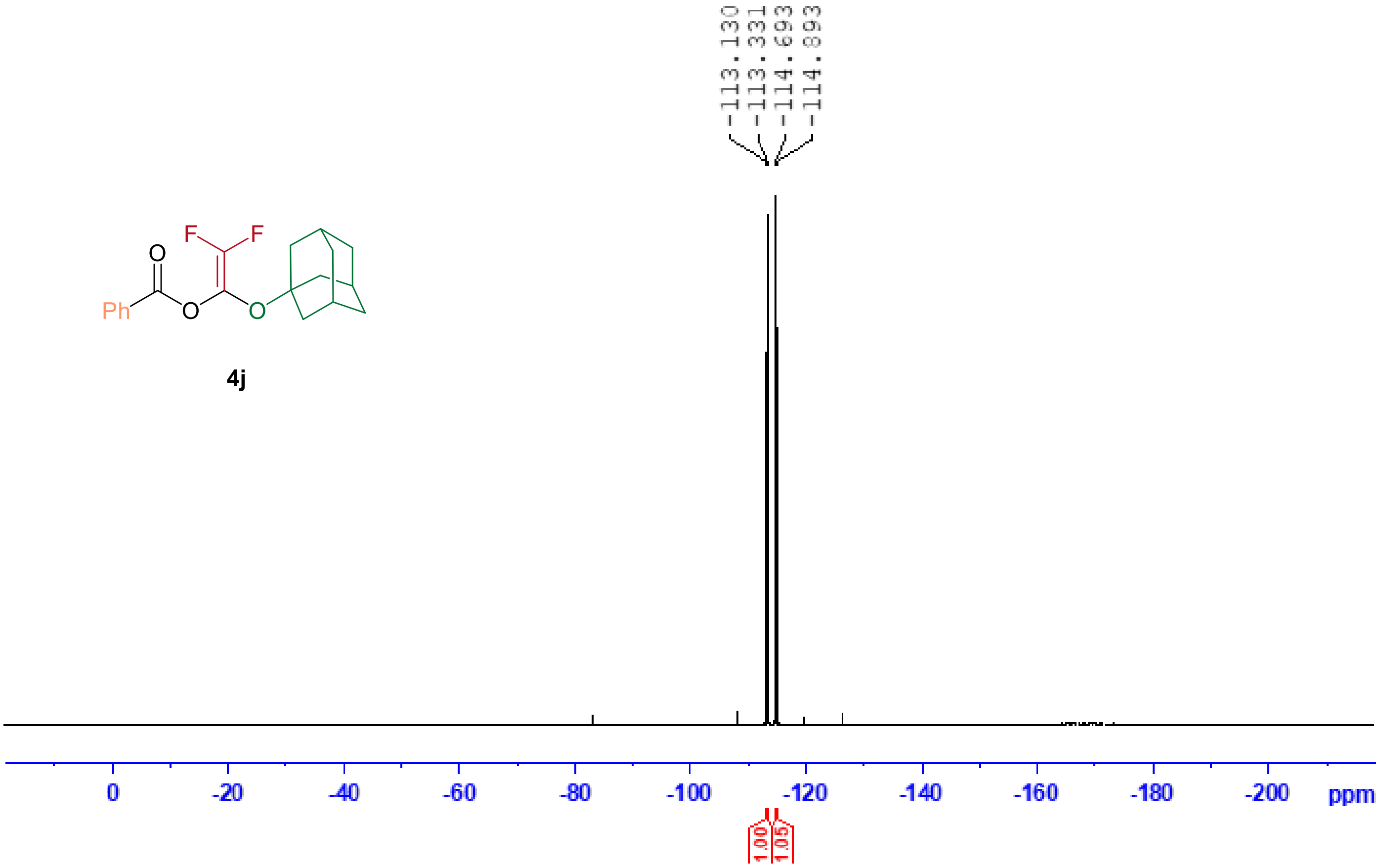

4j

^{1}H NMR of **4k**

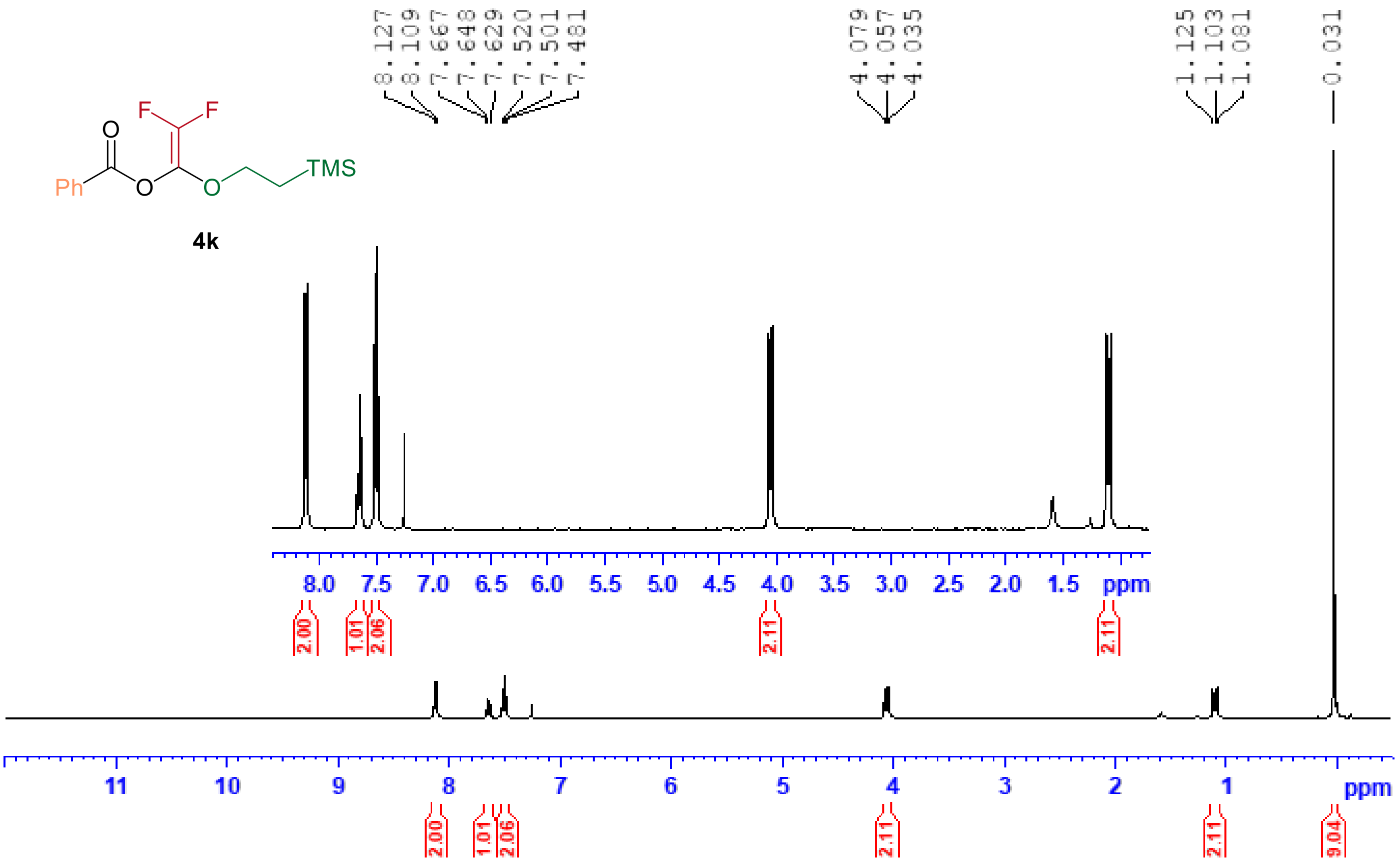

^{13}C NMR of **4k**

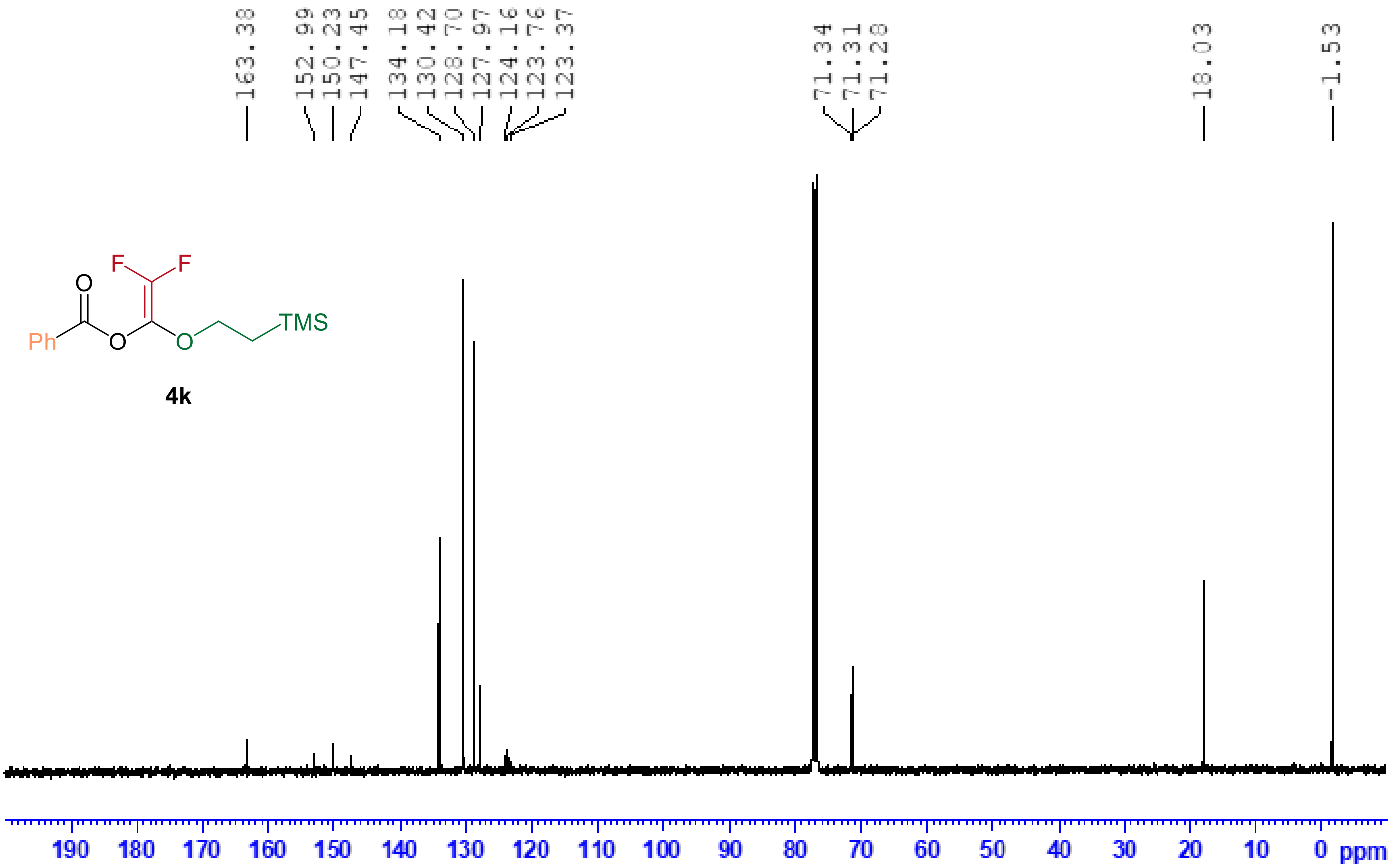

^{19}F NMR of **4k**

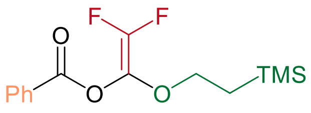

4k

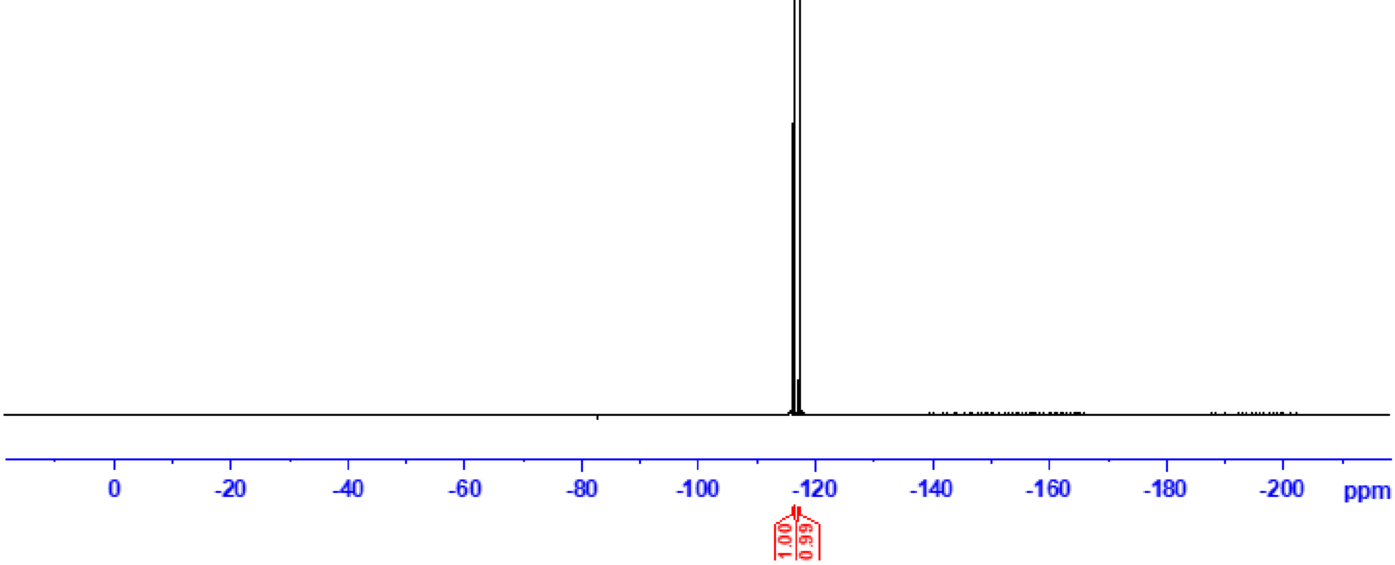

^{1}H NMR of **4l**

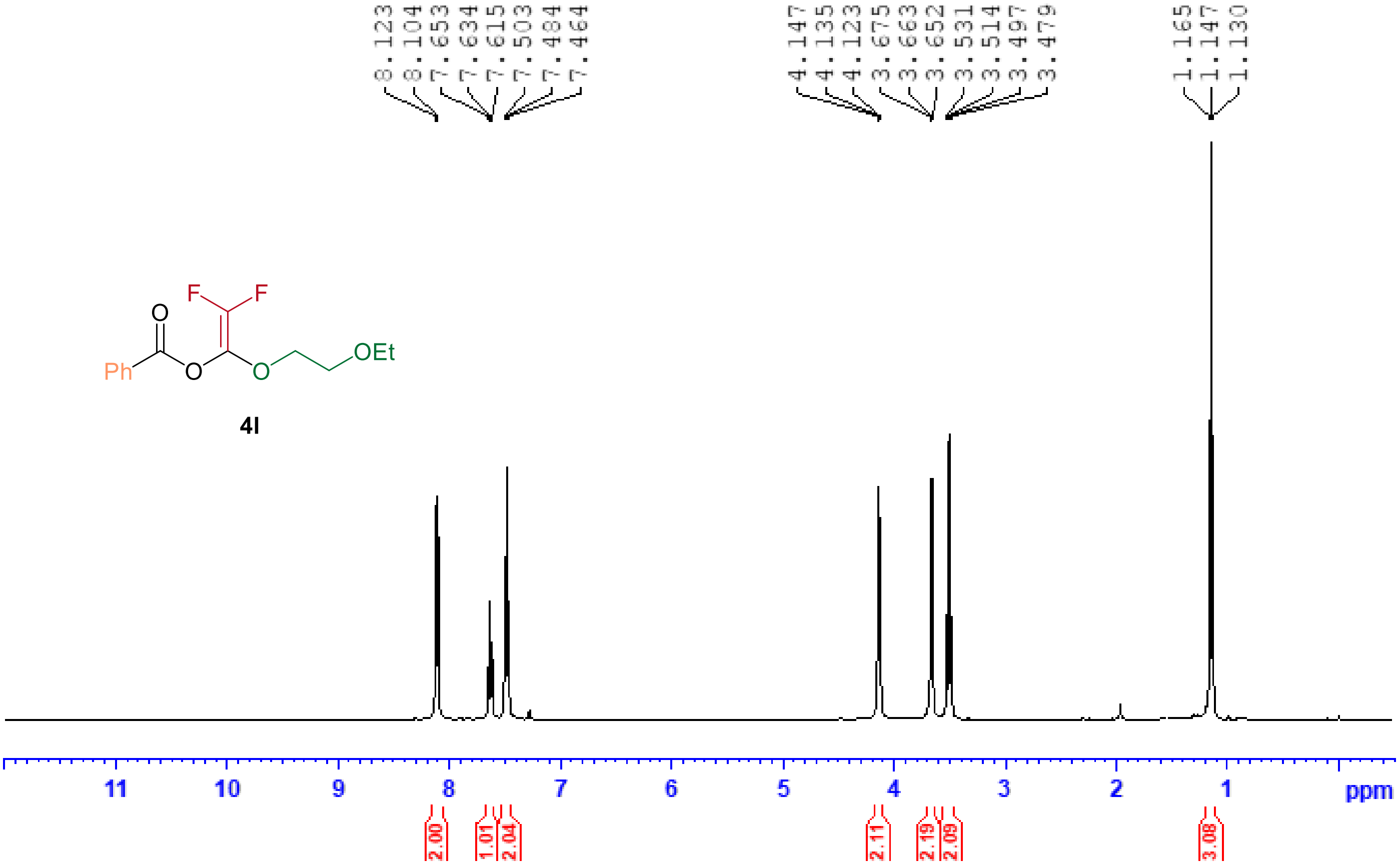

^{13}C NMR of **4l**

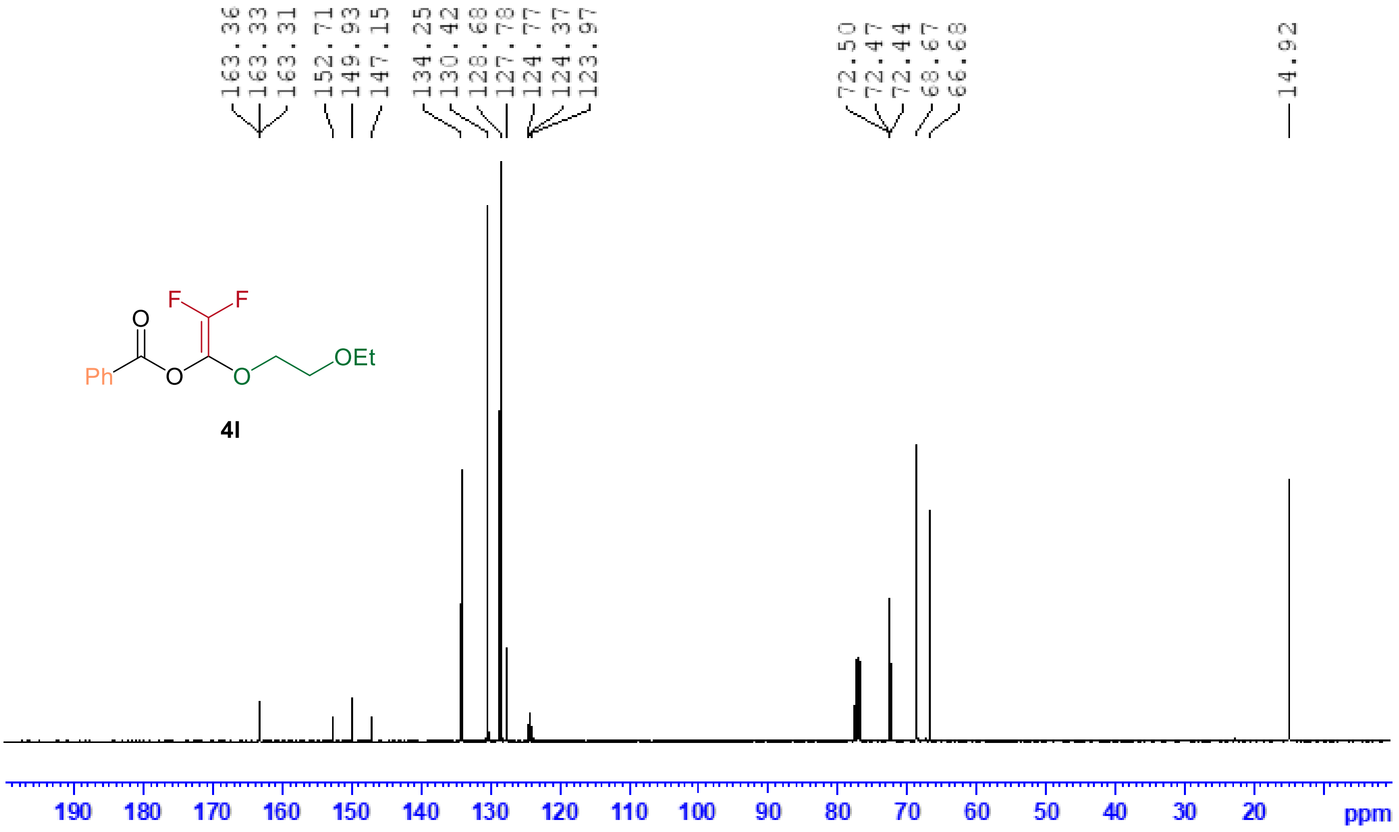

^{19}F NMR of **4l**

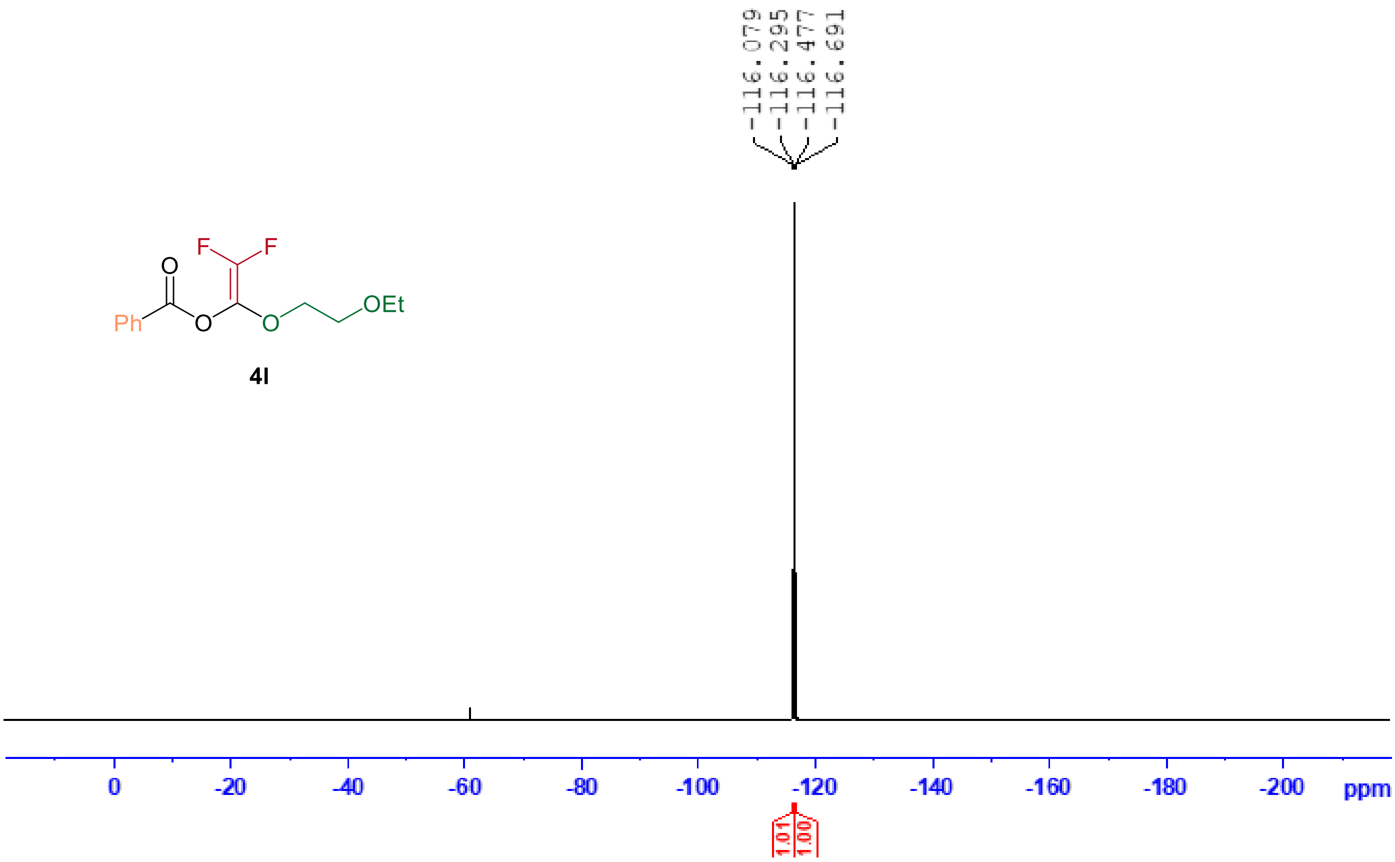

-116.079
-116.295
-116.477
-116.691
F
F
O
Ph
O
O
OEt
4l
0
-20
-40
-60
-80
-100
-120
-140
-160
-180
-200
ppm
1.01
1.00

^{1}H NMR of **4m**

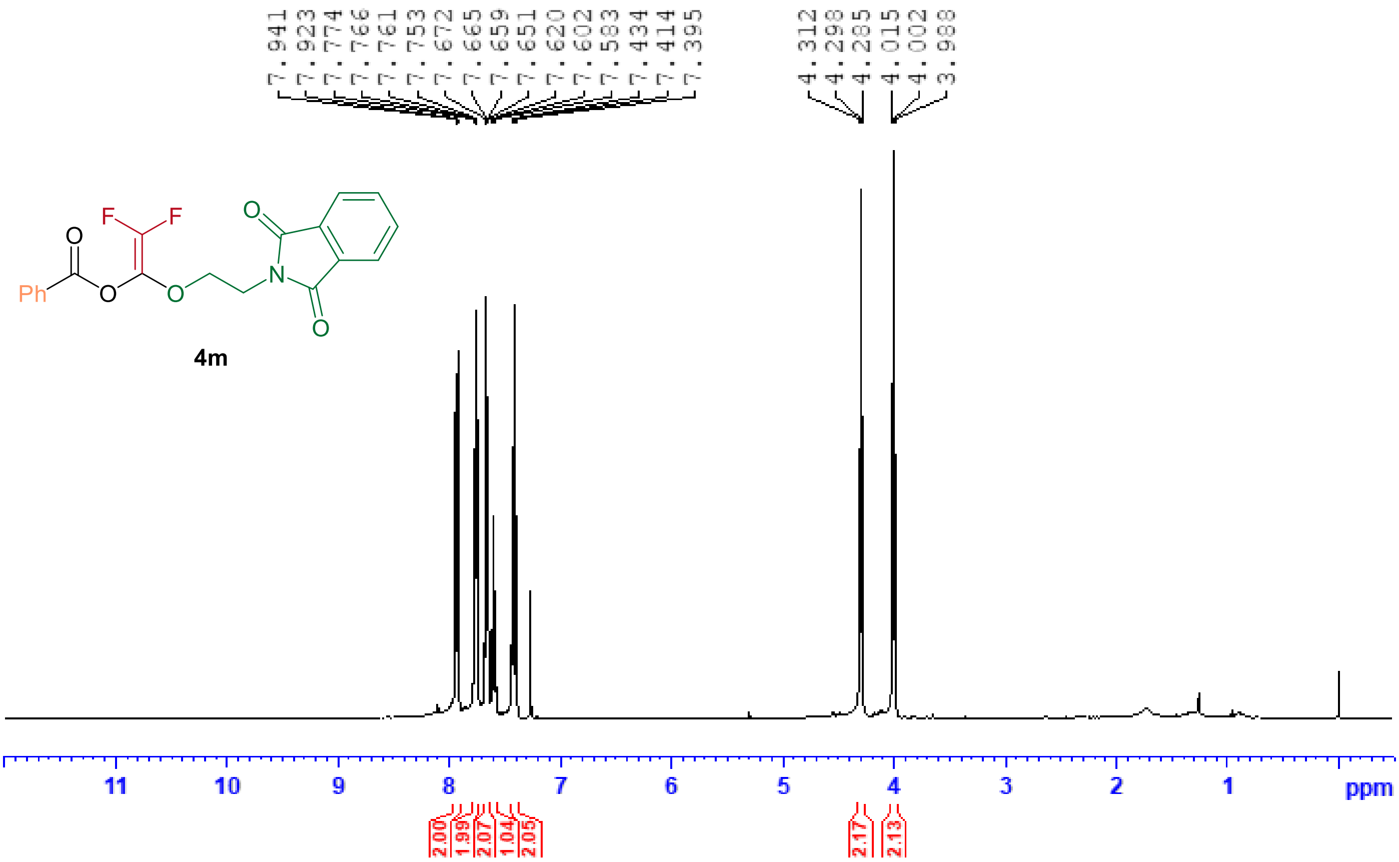

^{13}C NMR of **4m**

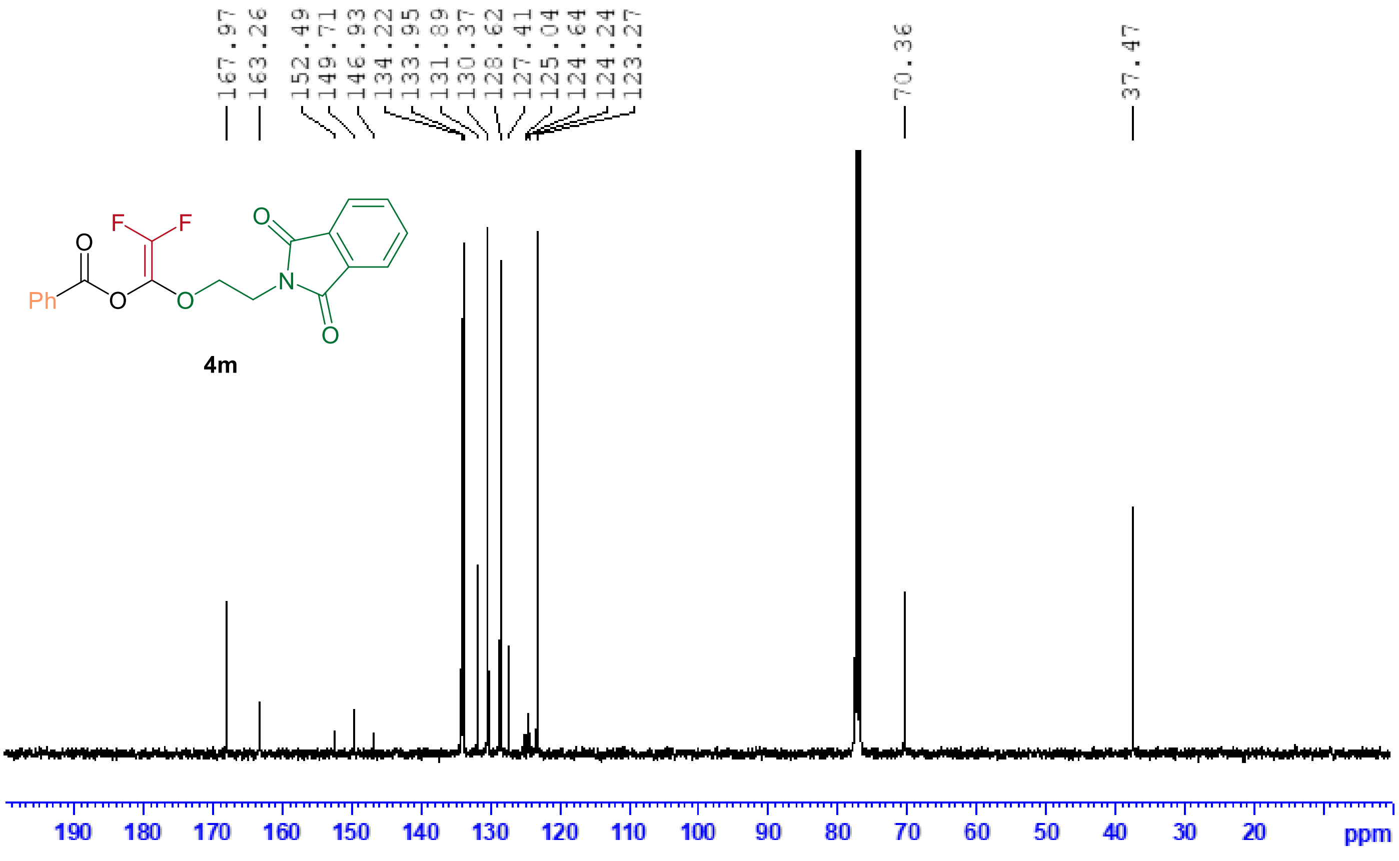

^{19}F NMR of **4m**

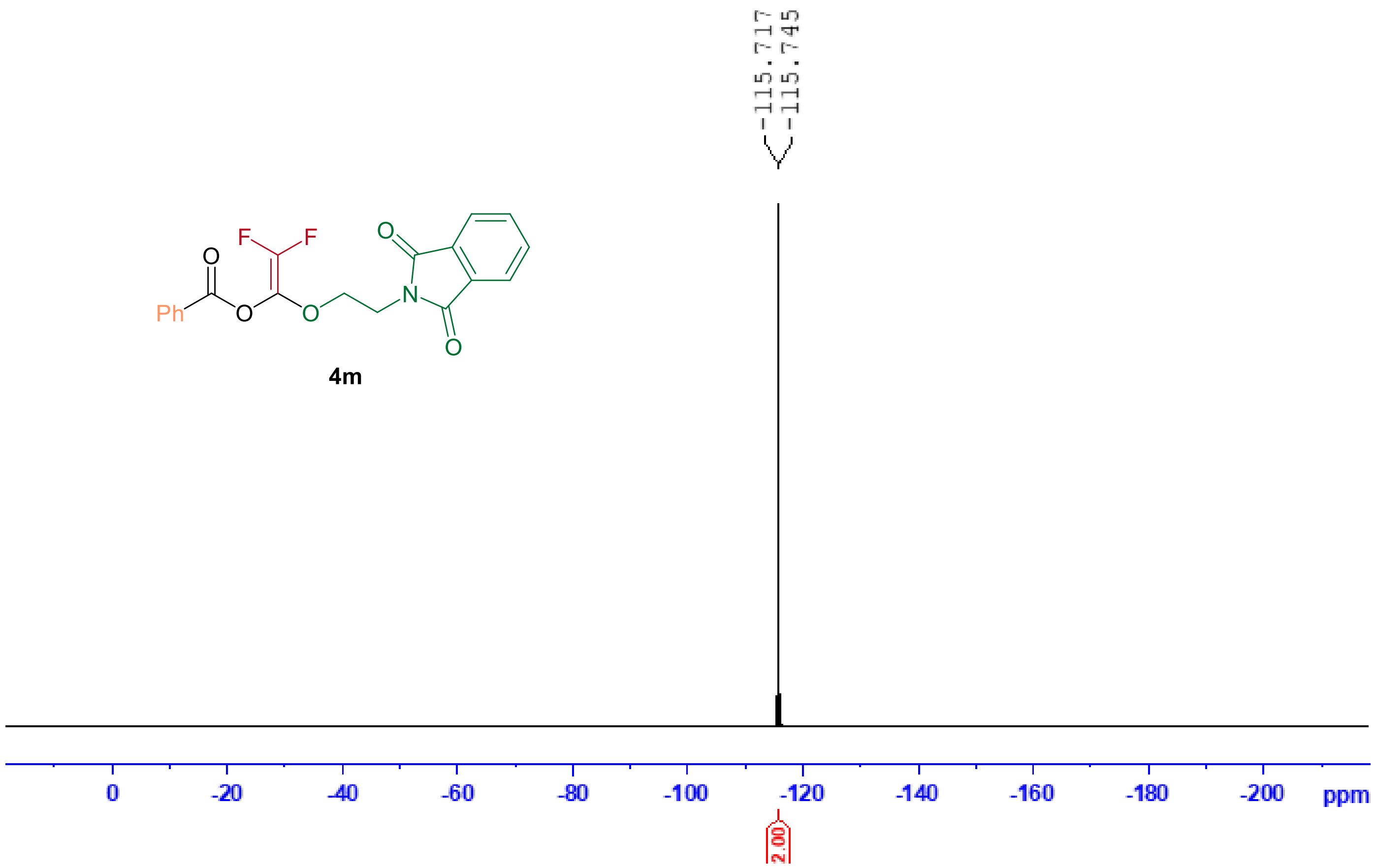
F
F
O
O
Ph
O
O
N
O
4m
-115.717
-115.745
0
-20
-40
-60
-80
-100
-120
-140
-160
-180
-200
ppm
2.00

^{1}H NMR of **4n**

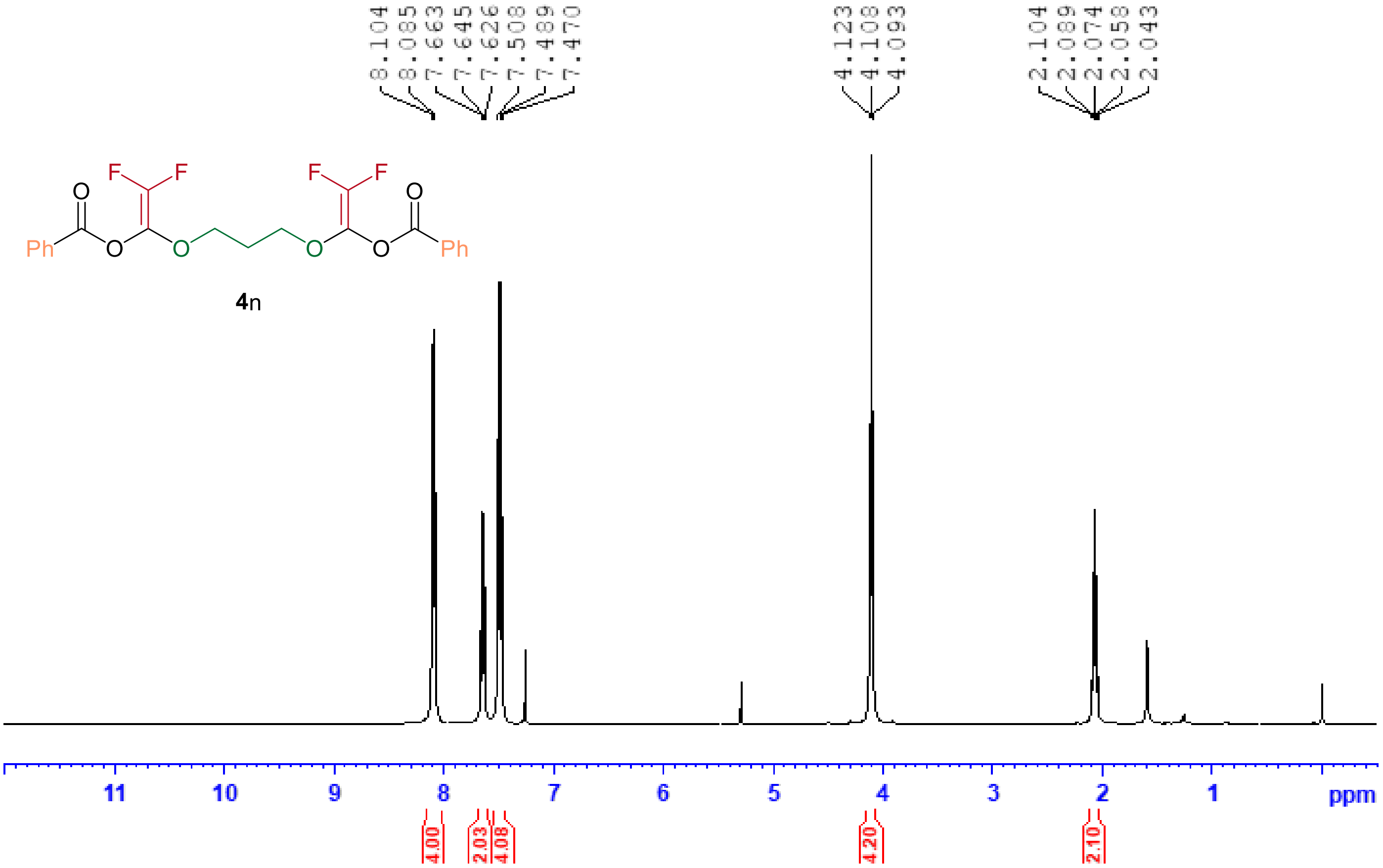

^{13}C NMR of **4n**

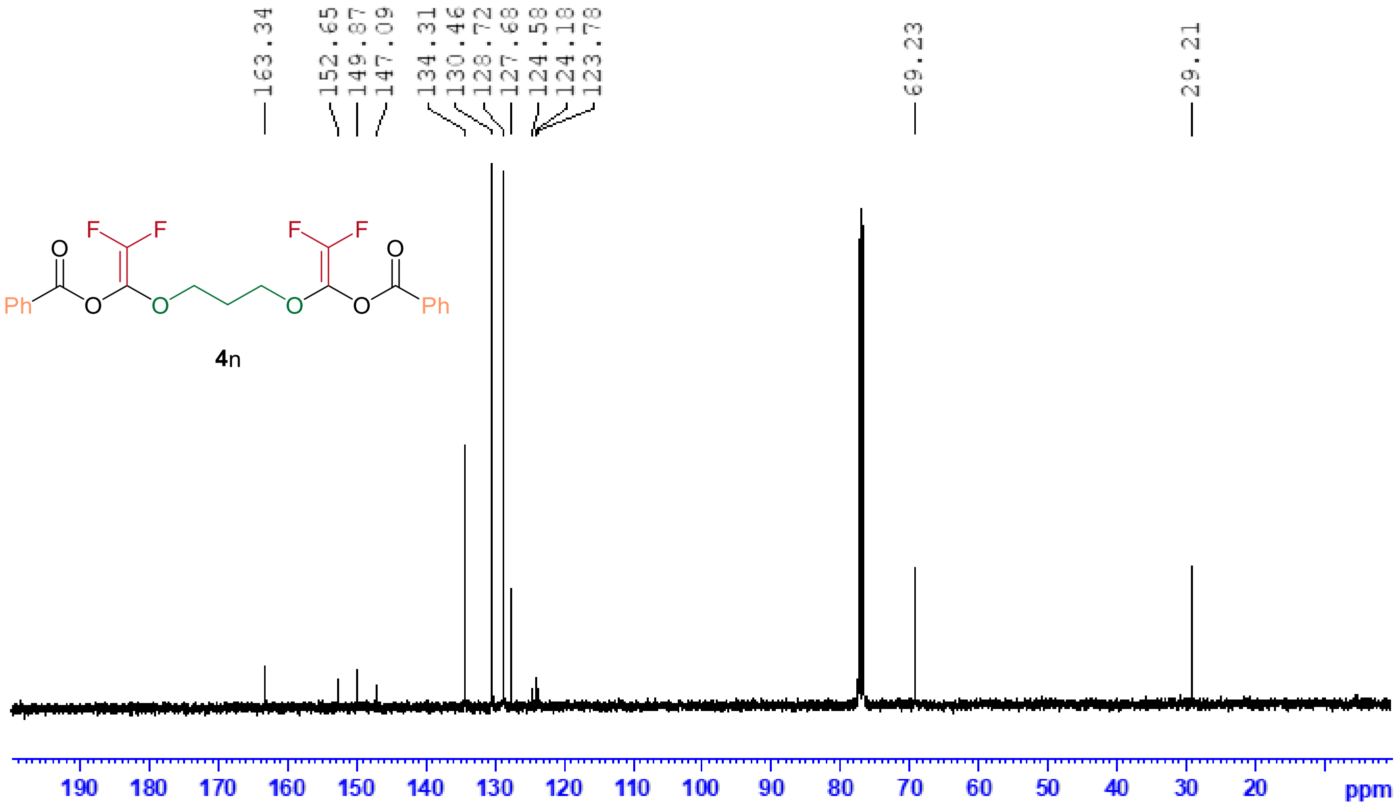

^{19}F NMR of **4n**

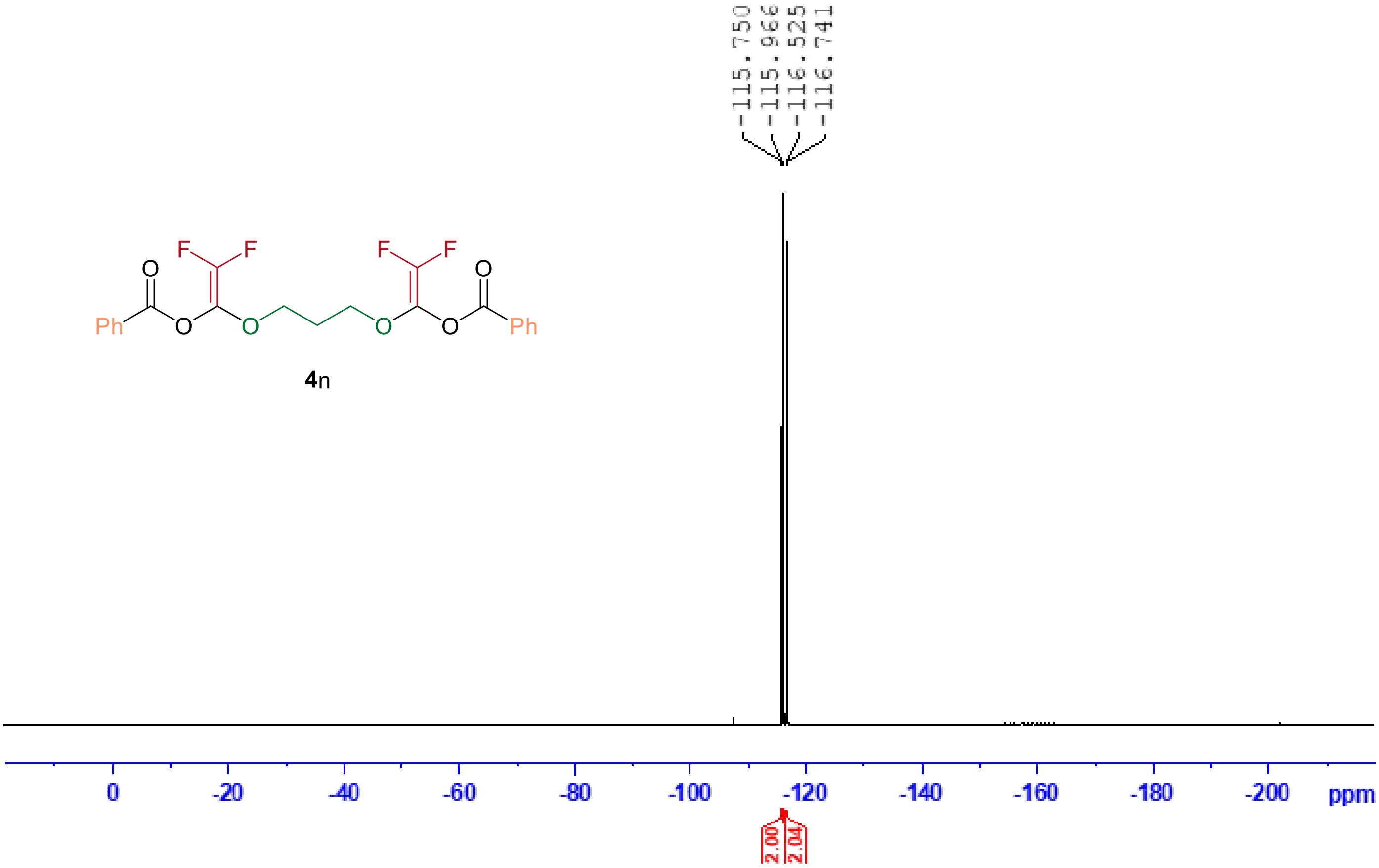
F
F
F
F
O
O
Ph
O
O
O
O
O
O
Ph
4n
-115.750
-115.966
-116.525
-116.741
0
-20
-40
-60
-80
-100
-120
-140
-160
-180
-200
ppm
2.00
2.04

^{1}H NMR of **4o**

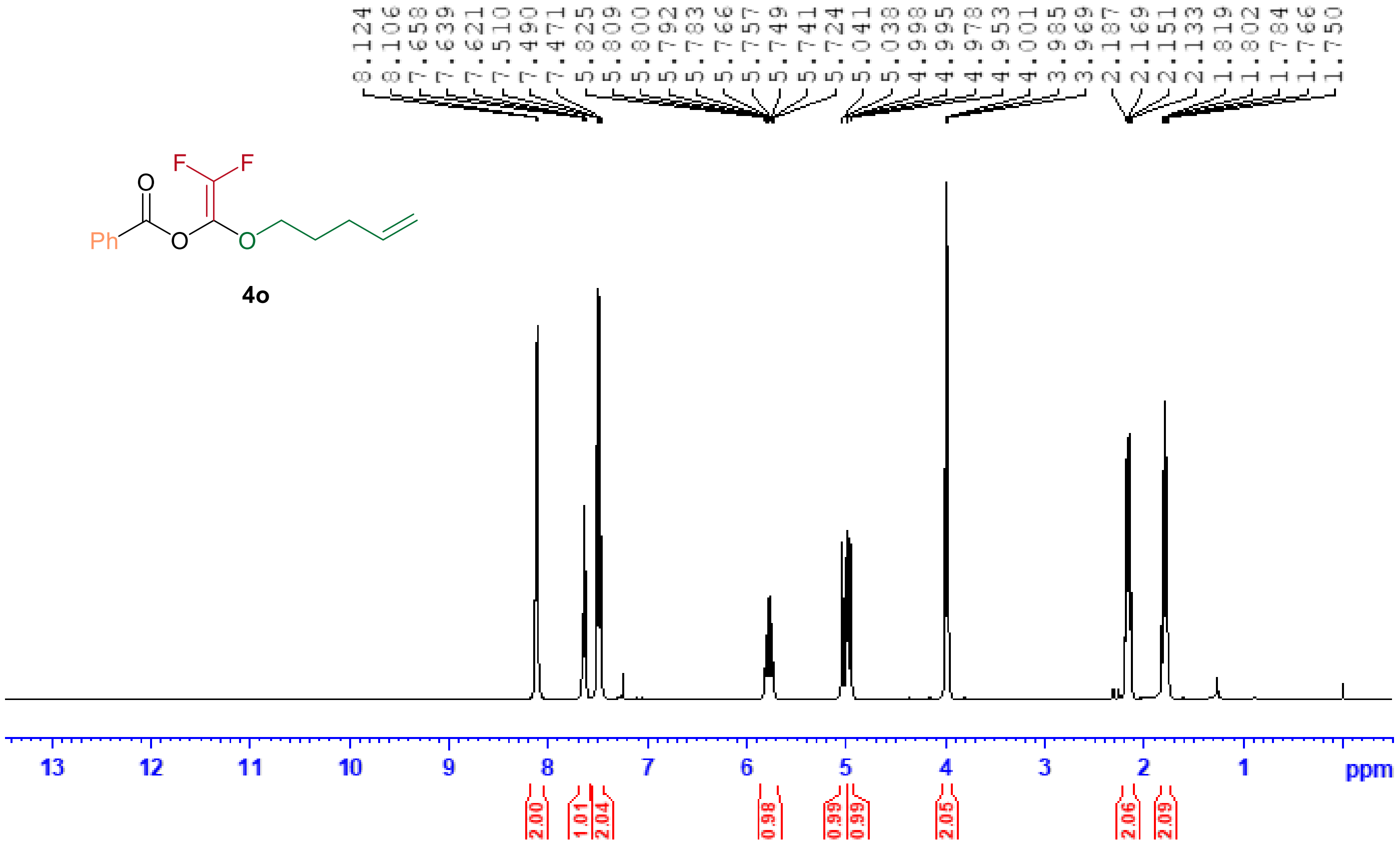

^{13}C NMR of **4o**

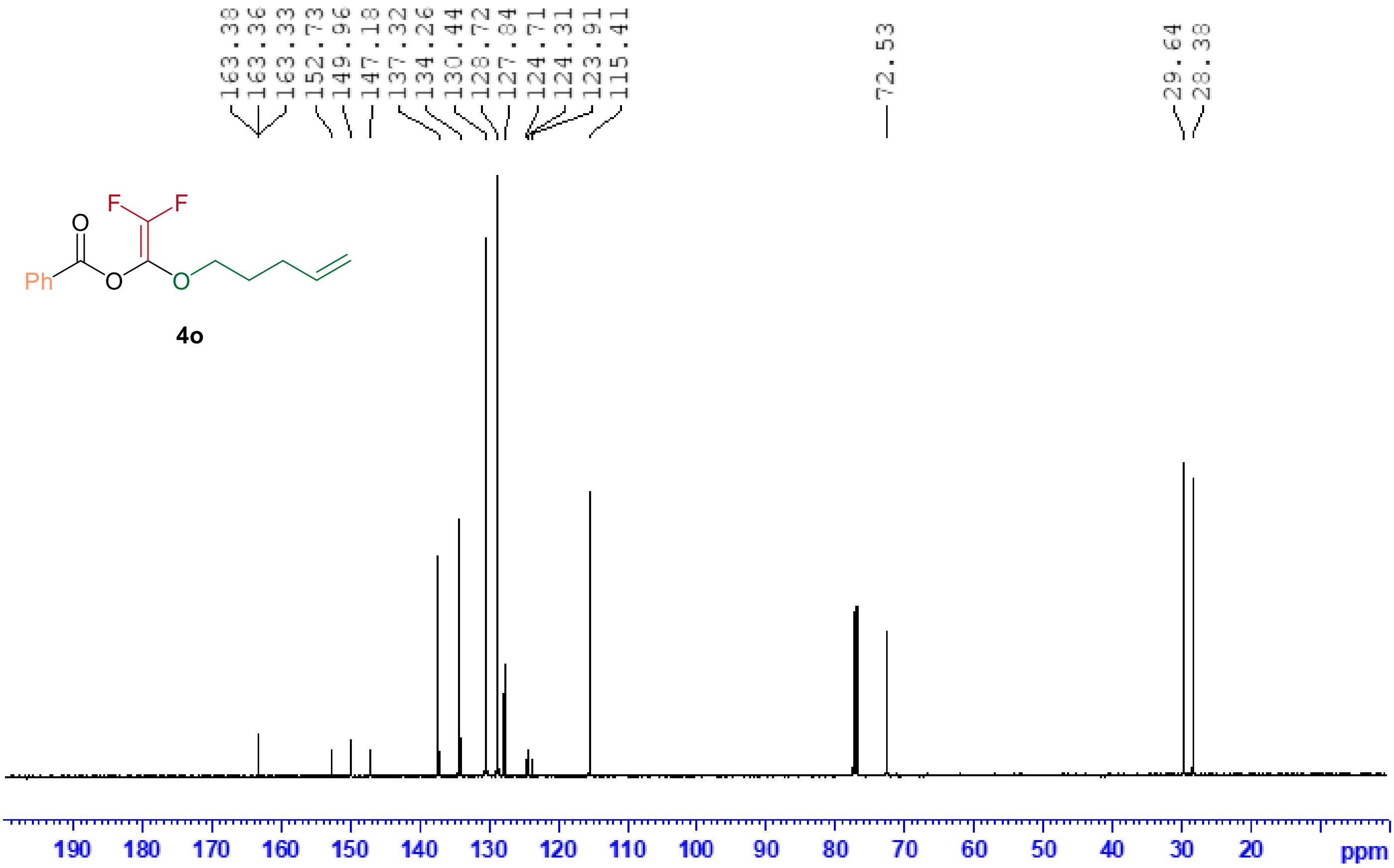

^{19}F NMR of **4o**

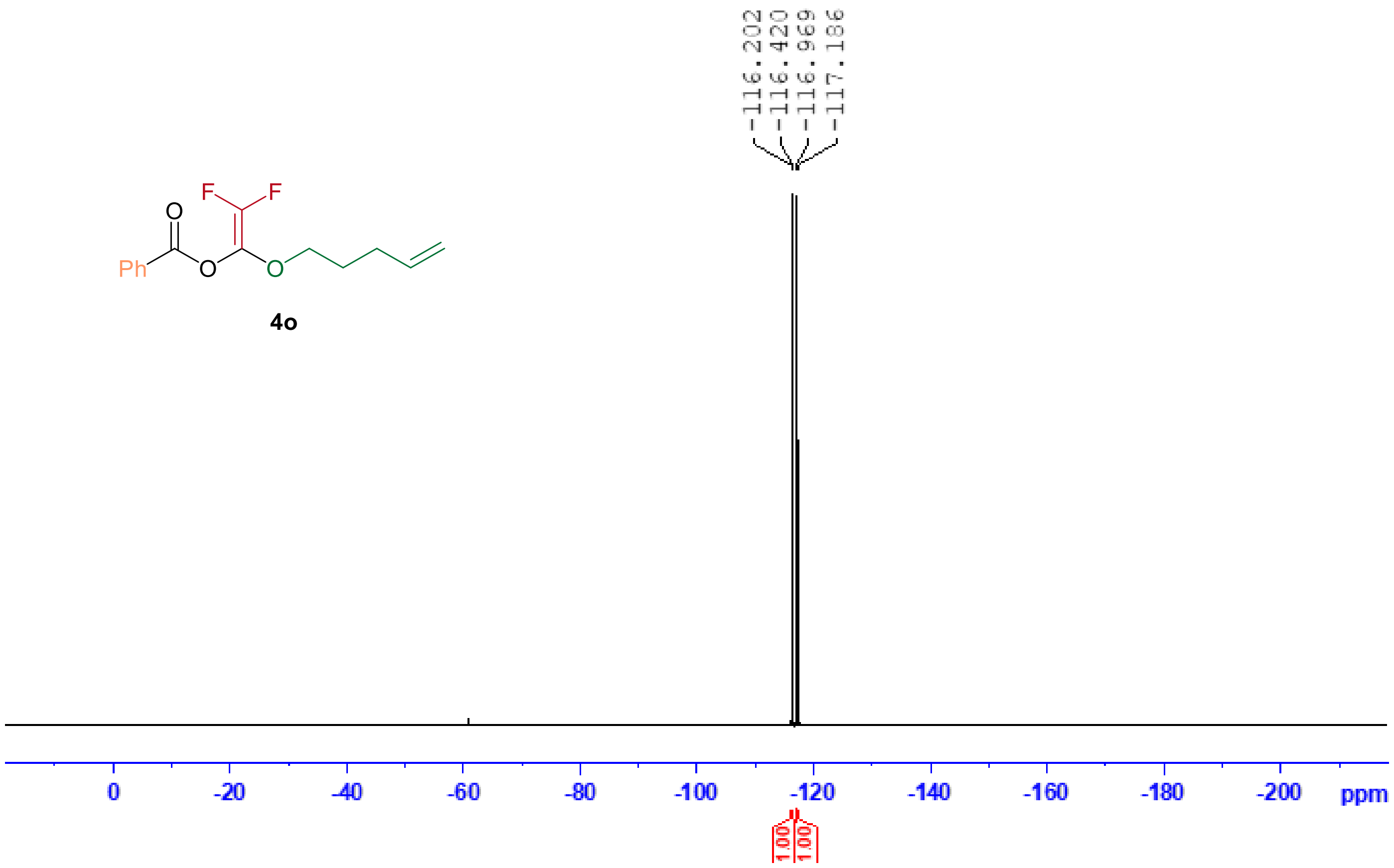
Ph
O
F
4o
-116.202
-116.420
-116.969
-117.186
0
-20
-40
-60
-80
-100
-120
-140
-160
-180
-200
ppm
1.00
1.00

^{1}H NMR of **4p**

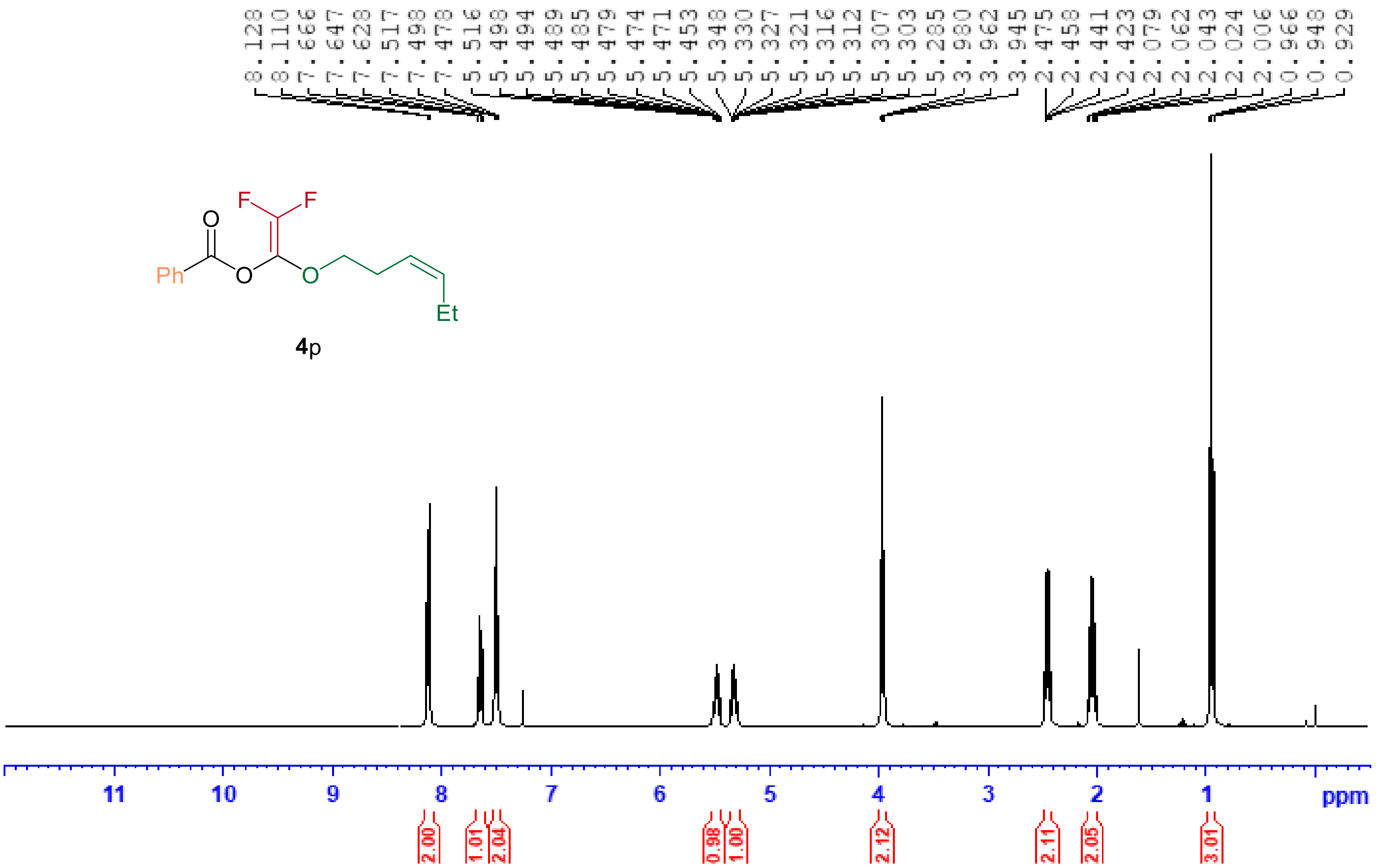

^{13}C NMR of **4p**

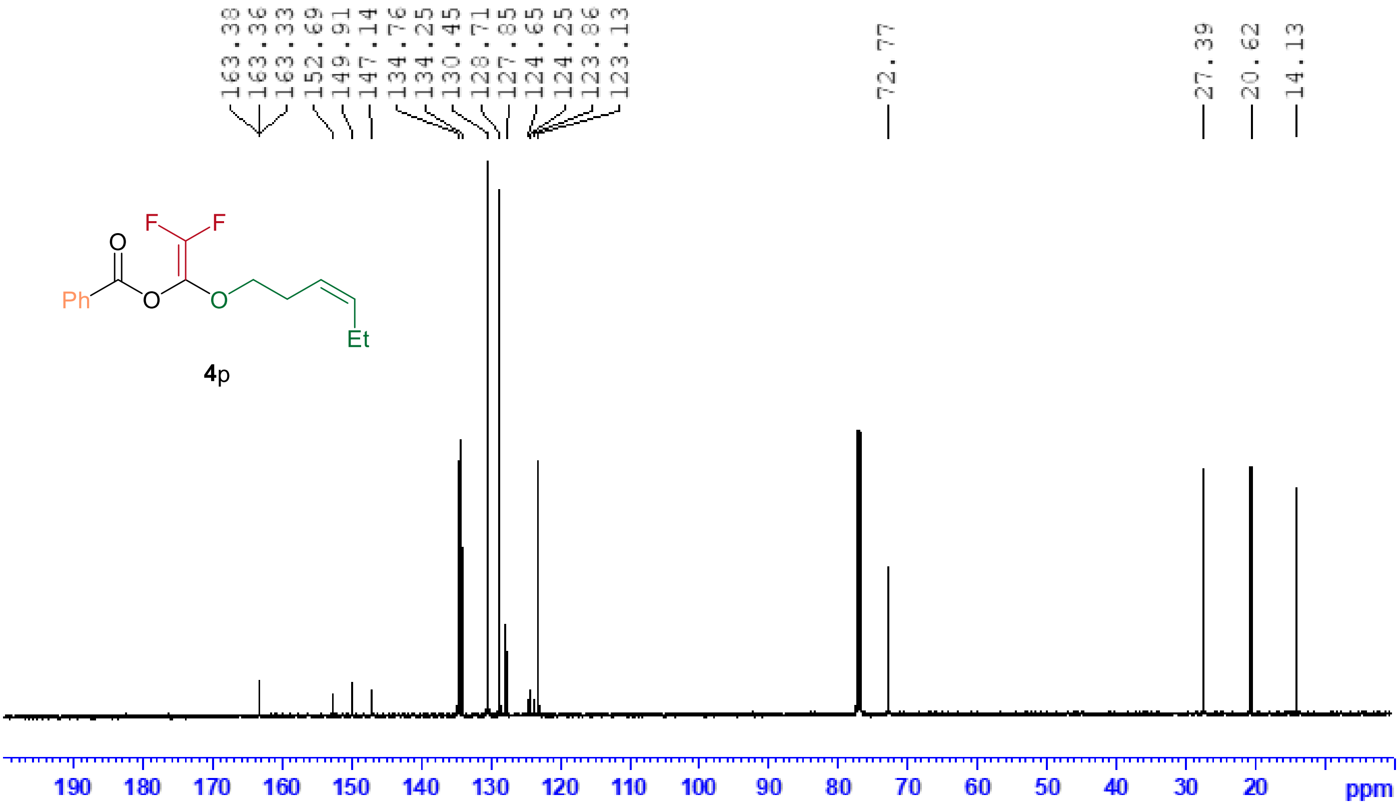

^{19}F NMR of **4p**

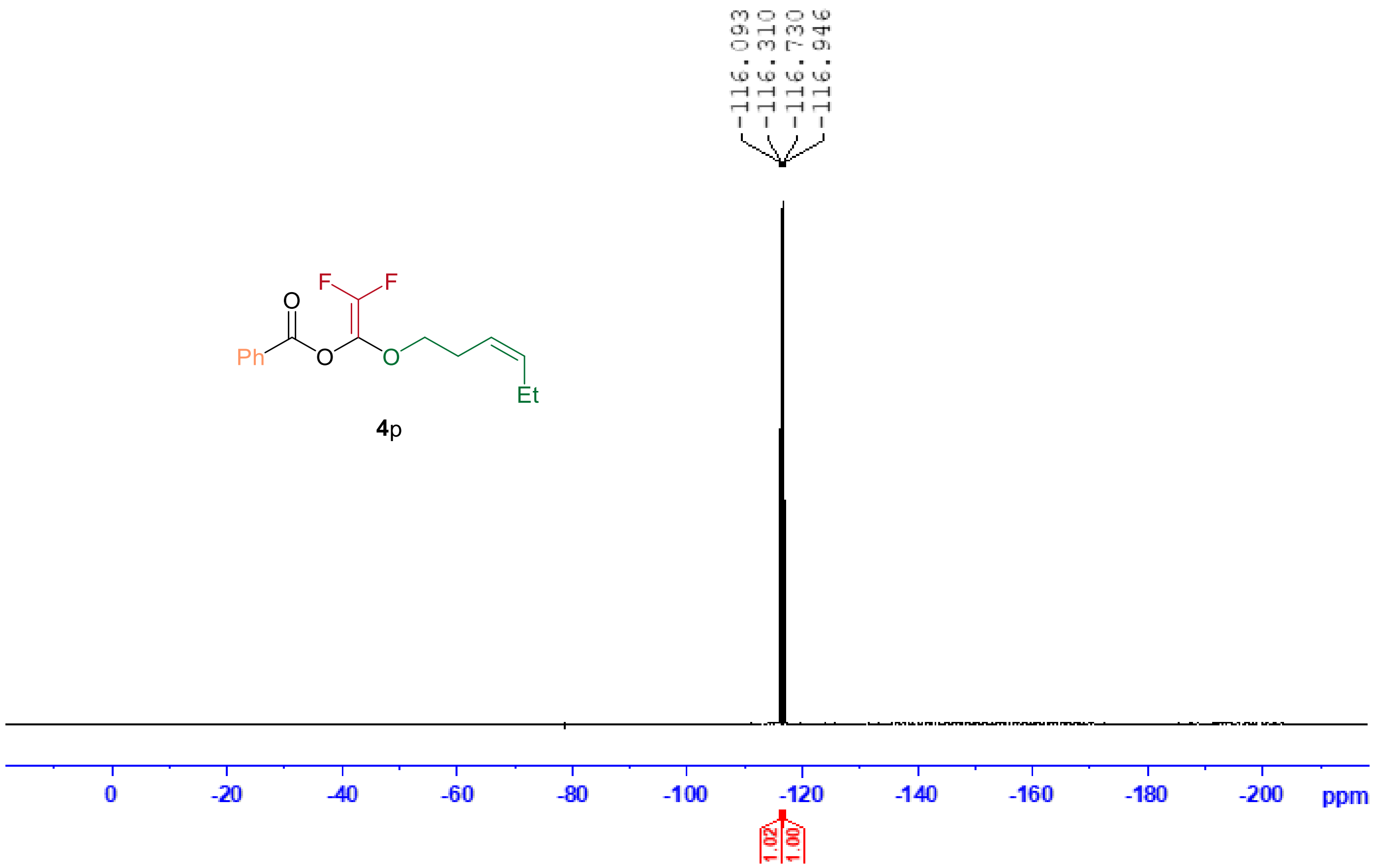
-116.093
-116.310
-116.730
-116.946
F
F
O
Ph
O
O
Et
4p
0
-20
-40
-60
-80
-100
-120
-140
-160
-180
-200
ppm
1.02
1.00

^{1}H NMR of **4q**

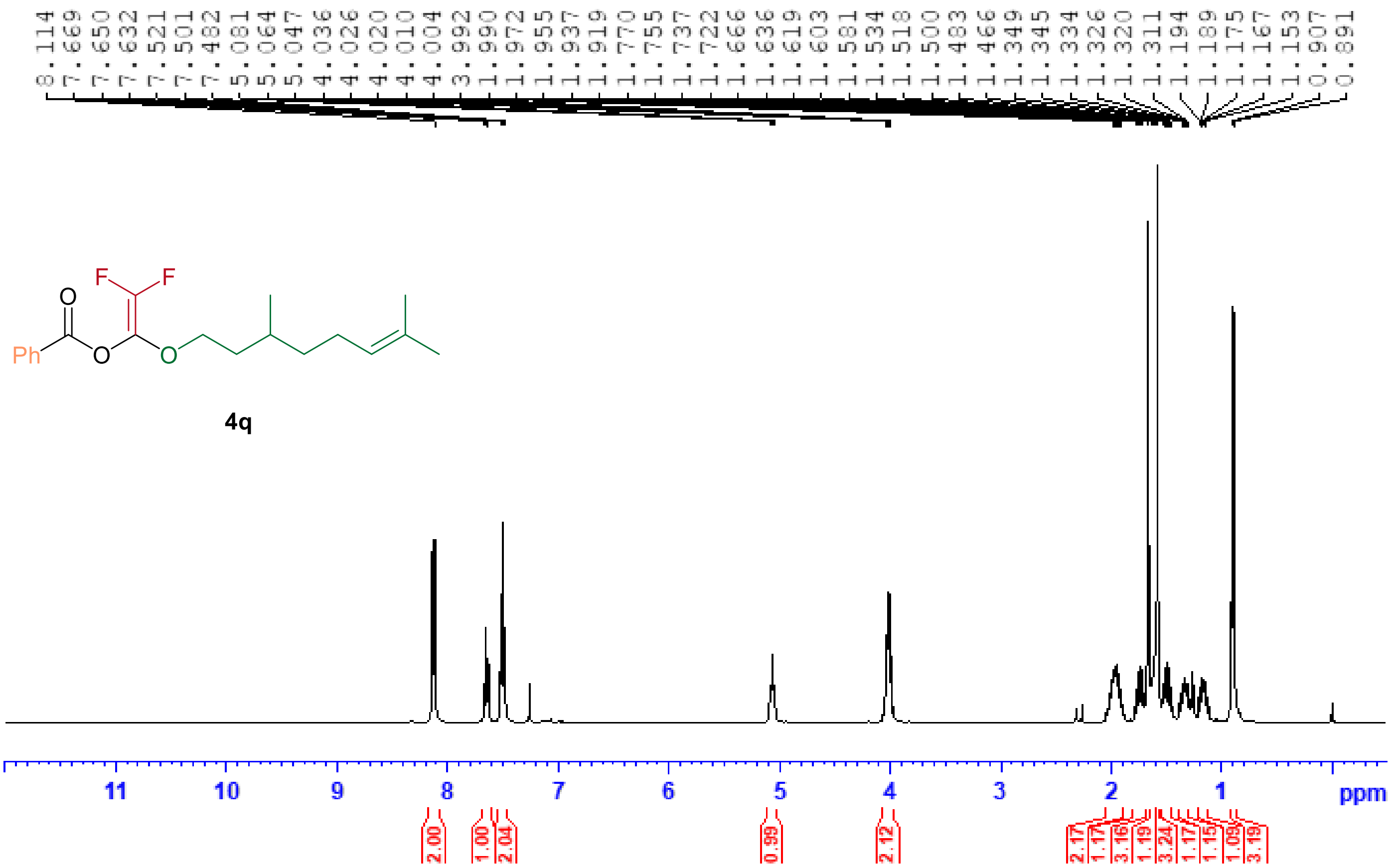

^{13}C NMR of **4q**

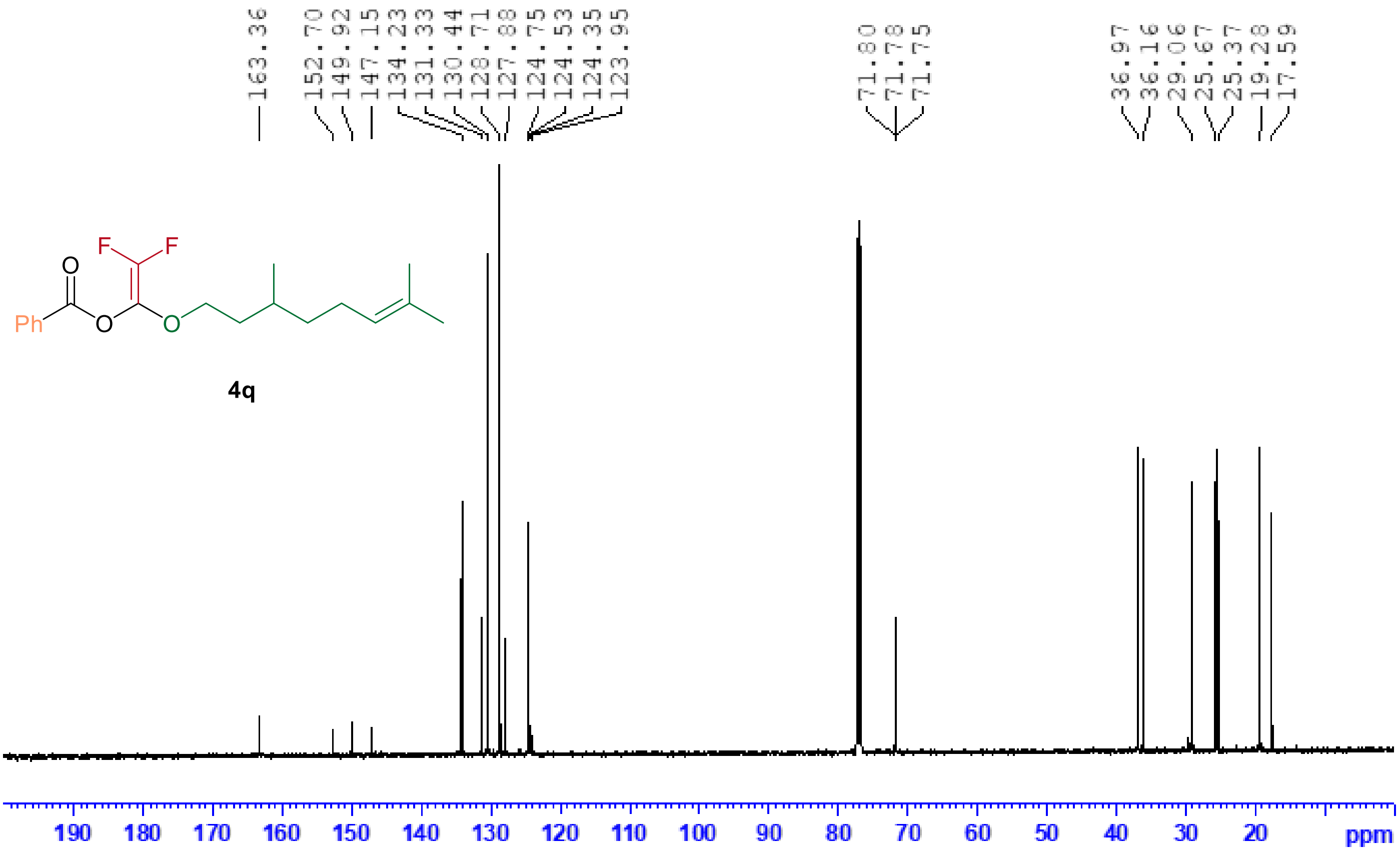

^{19}F NMR of **4q**

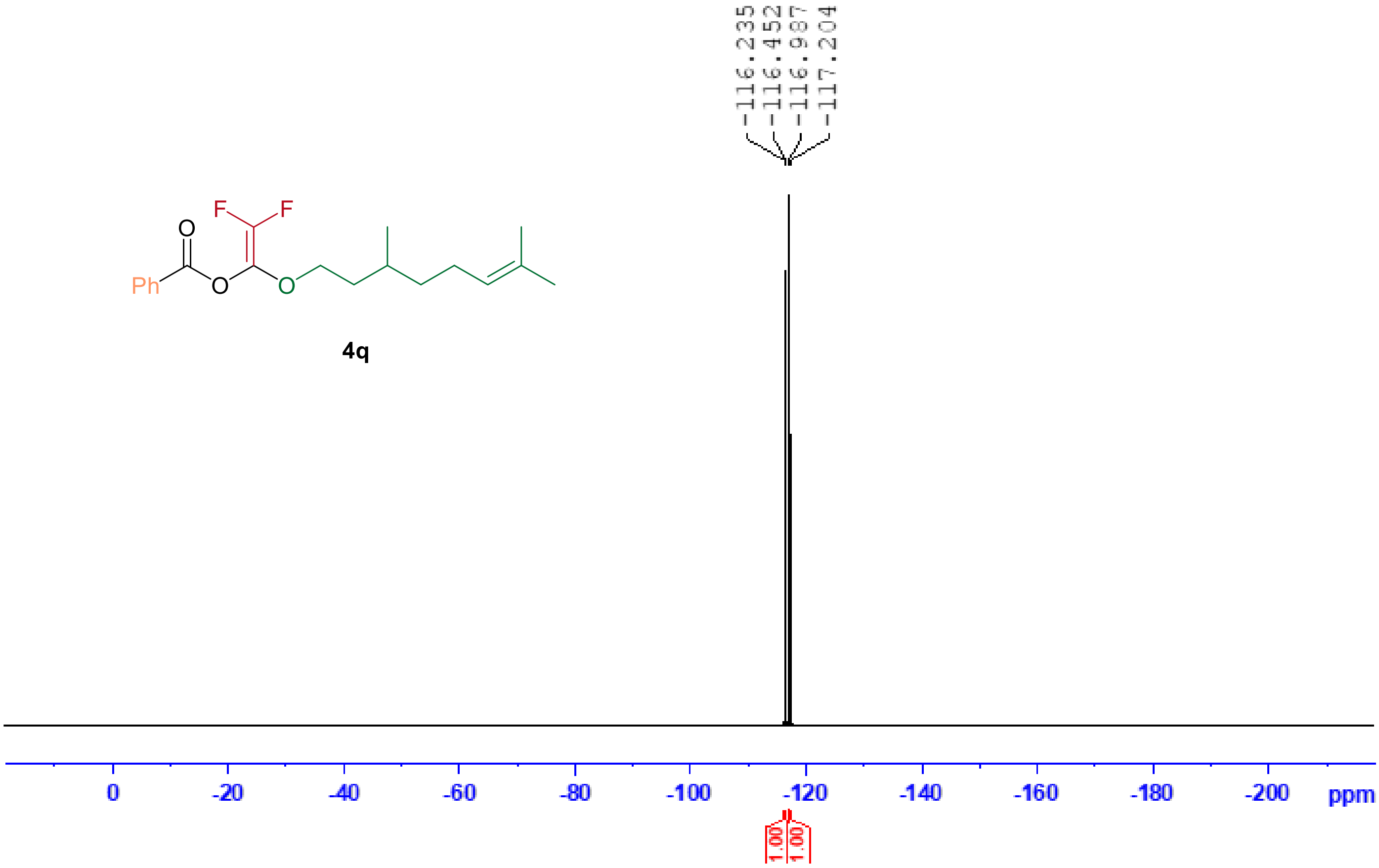

4q
-116.235
-116.452
-116.987
-117.204
0
-20
-40
-60
-80
-100
-120
-140
-160
-180
-200
ppm
1.00
1.00

^{1}H NMR of **4r**

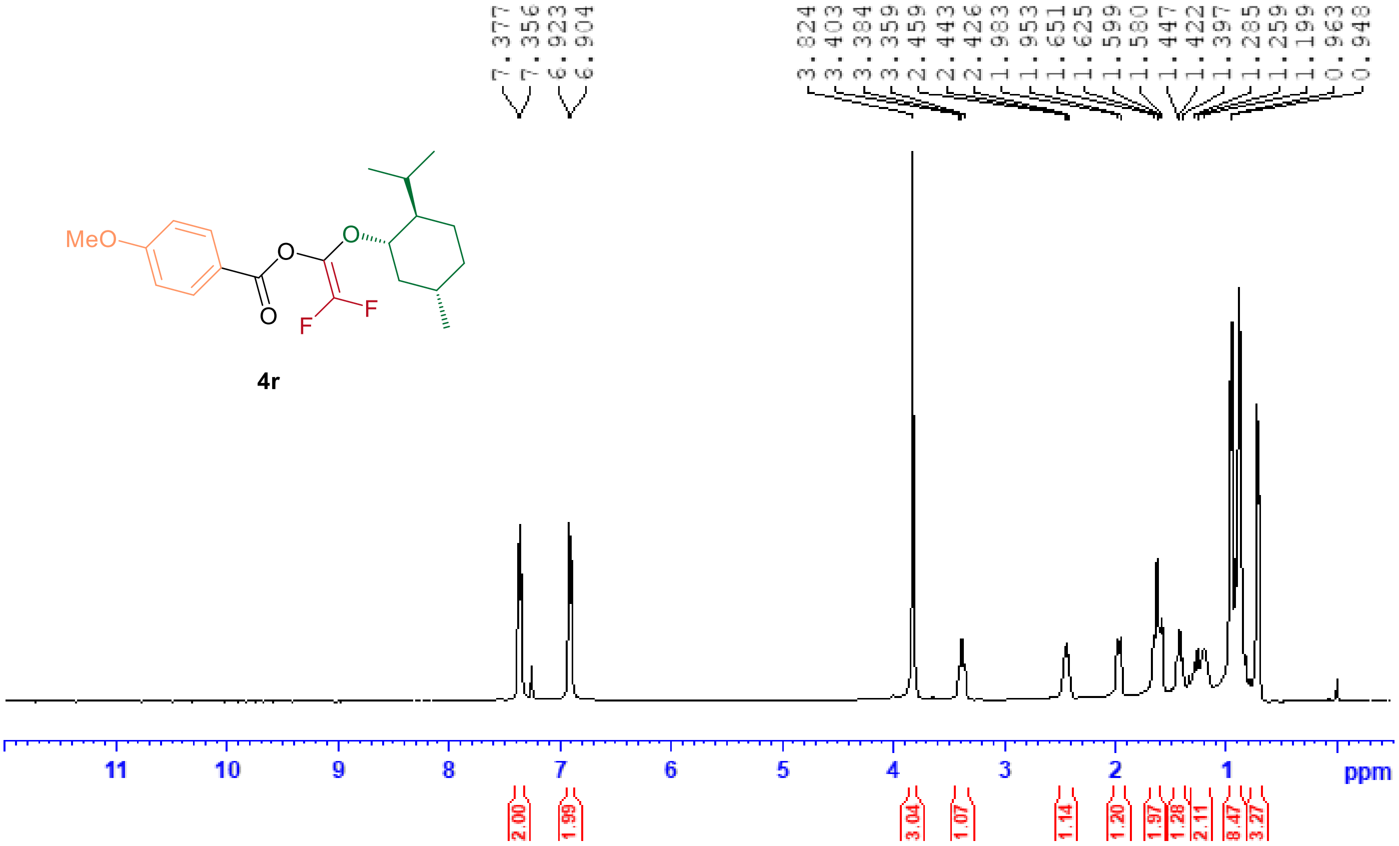

^{13}C NMR of **4r**

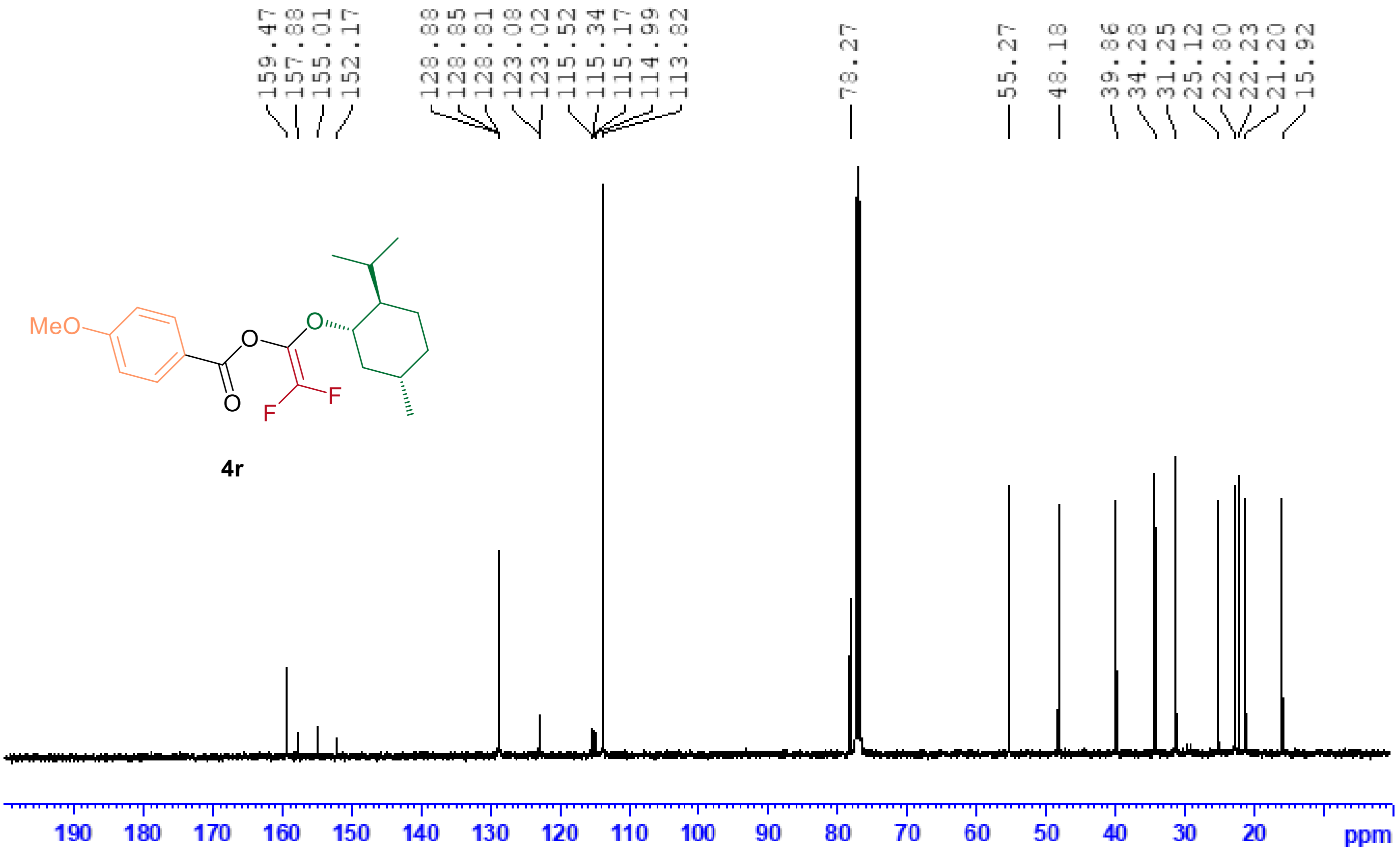

^{19}F NMR of **4r**

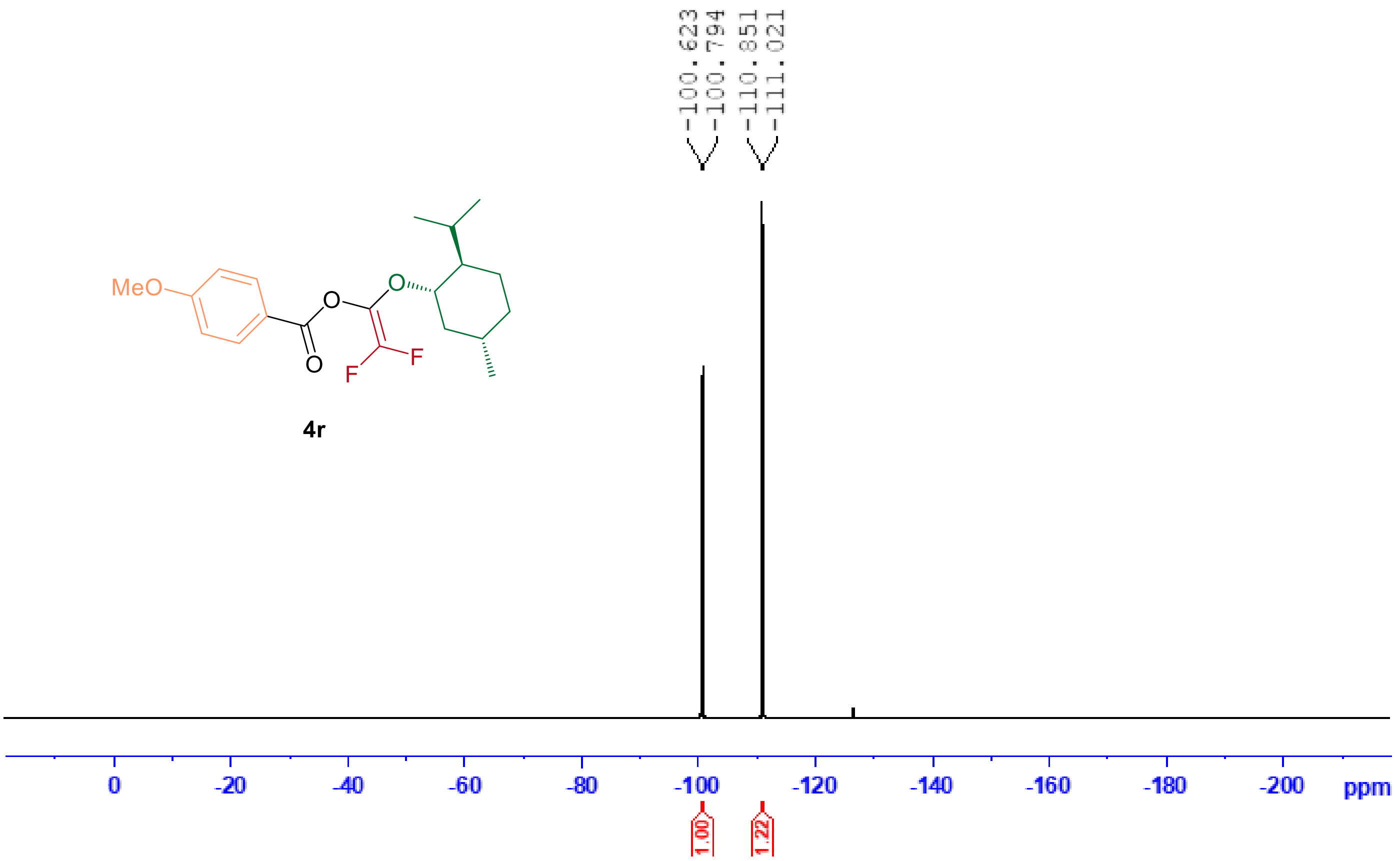
-100.623
-100.794
-110.851
-111.021
MeO
O
O
O
F
F
4r
0
-20
-40
-60
-80
-100
-120
-140
-160
-180
-200
ppm
1.00
1.22

^{1}H NMR of **4s**

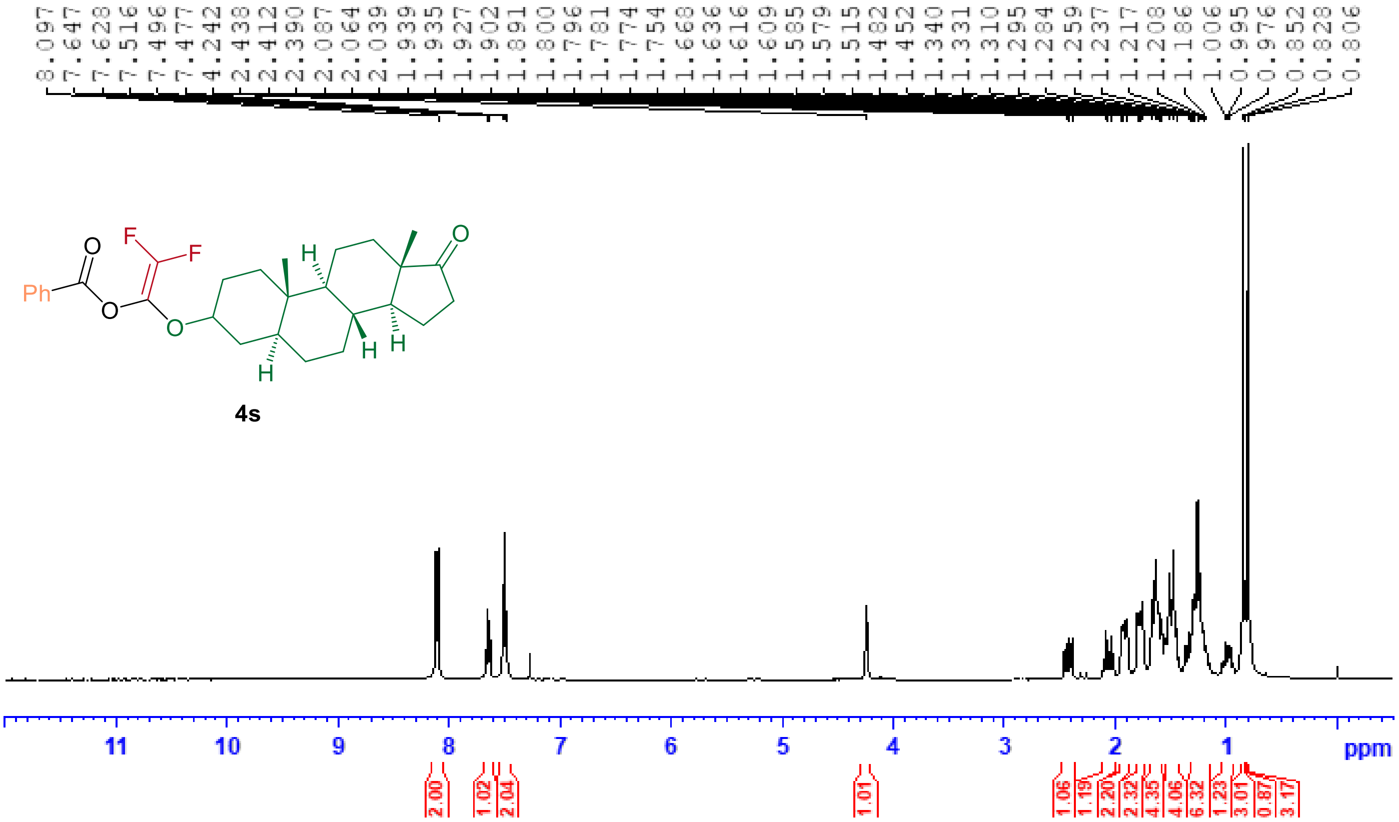

^{13}C NMR of **4s**

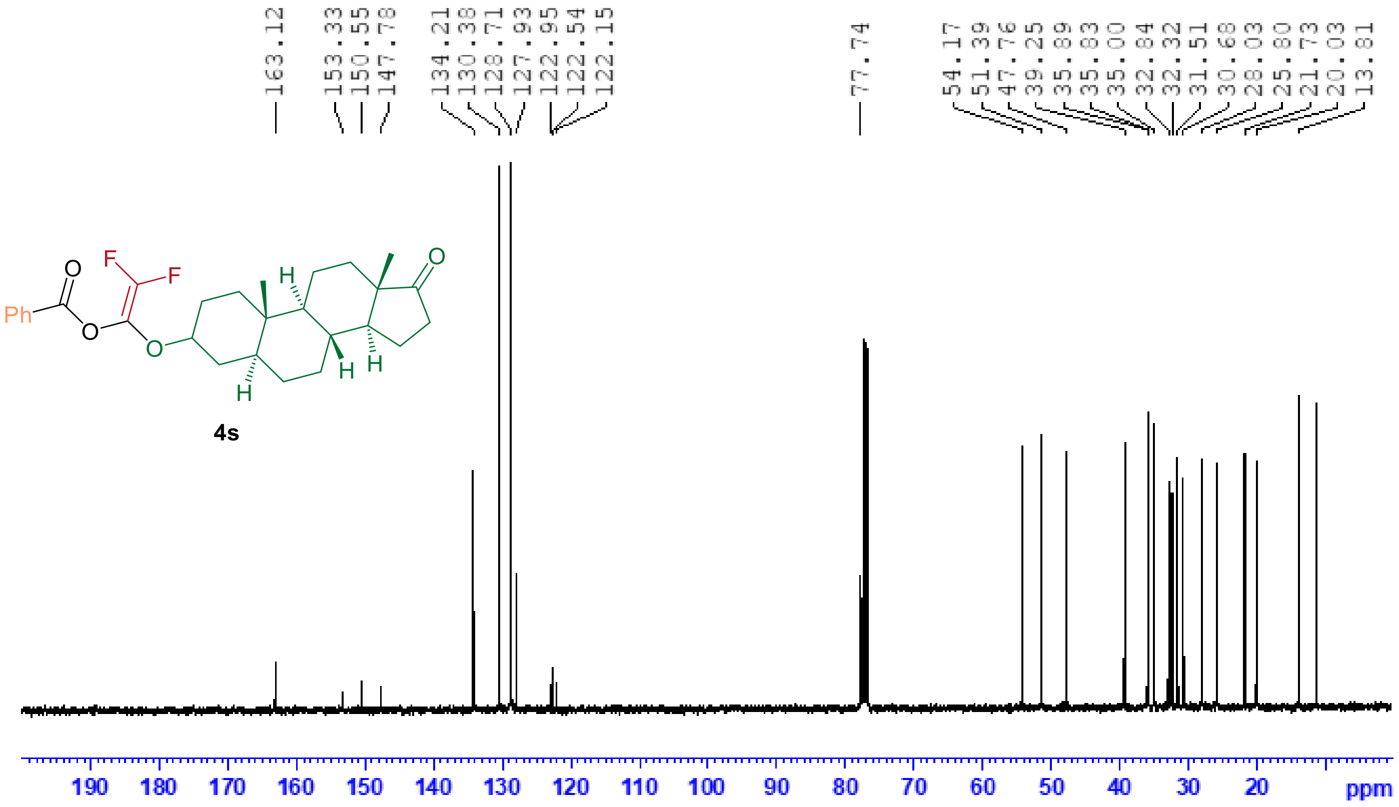

^{19}F NMR of **4s**

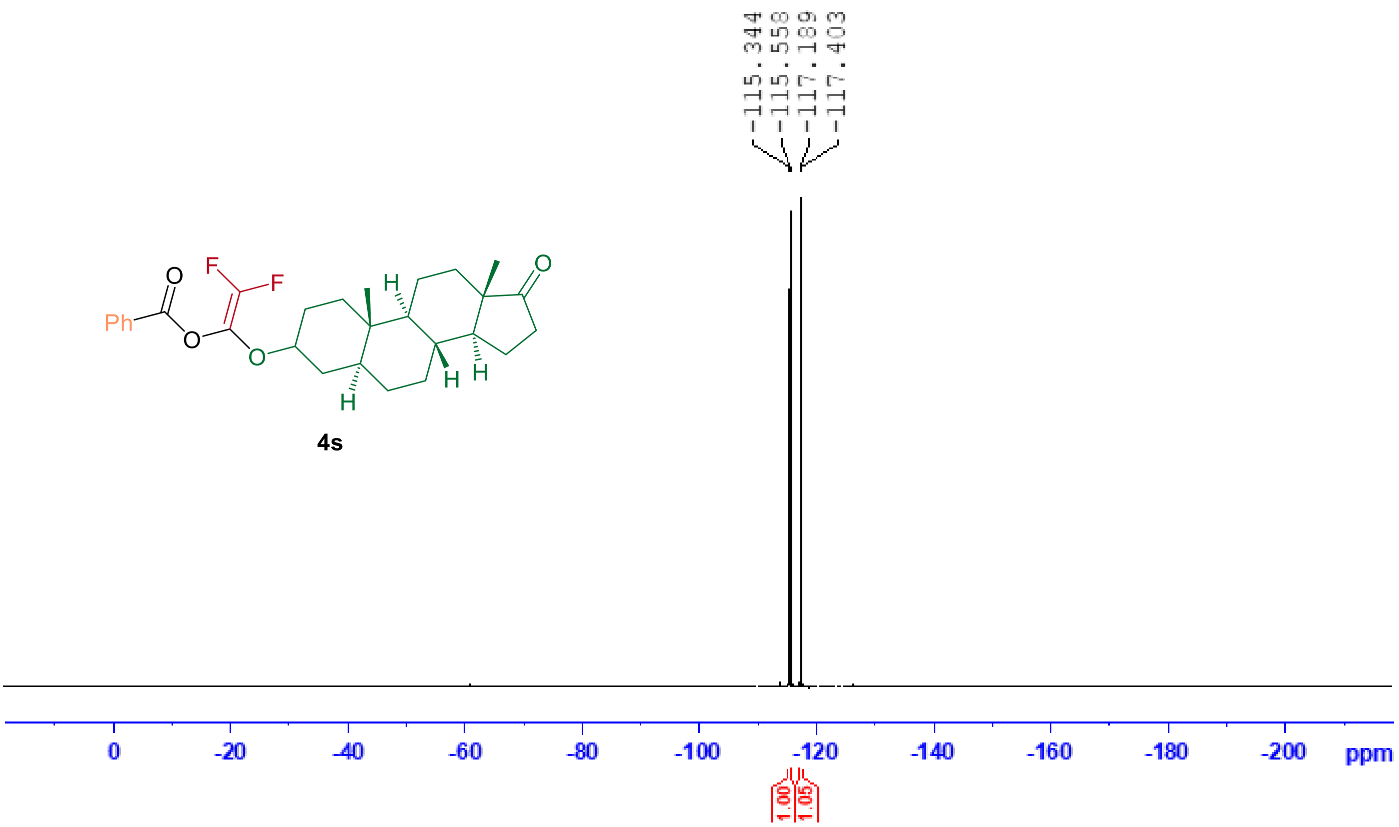

-115.344
-115.558
-117.189
-117.403
Ph
O
O
F
F
O
H
H
H
H
O
4s
0
-20
-40
-60
-80
-100
-120
-140
-160
-180
-200
ppm
1.00
1.05

^{1}H NMR of **4t**

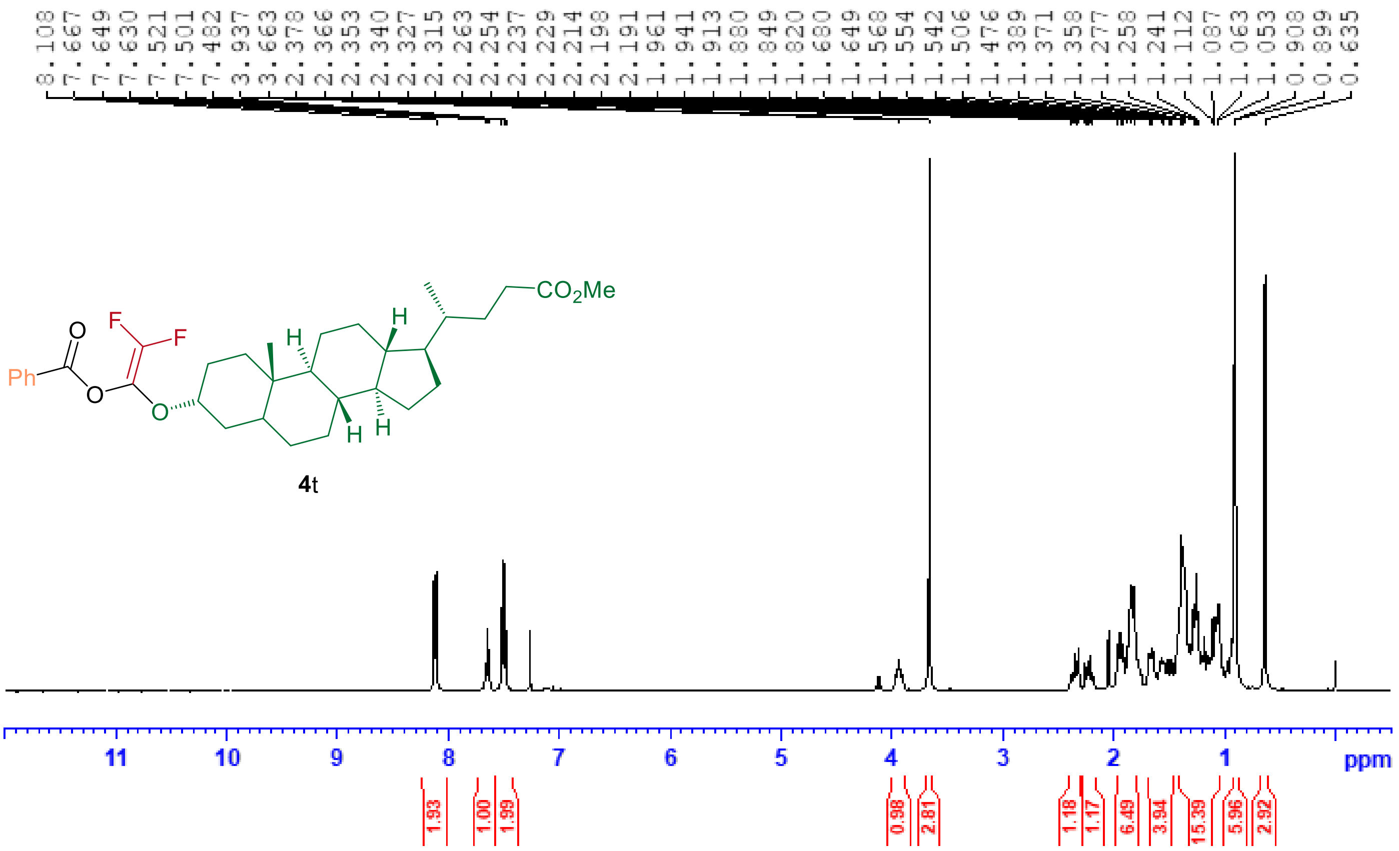

^{13}C NMR of **4t**

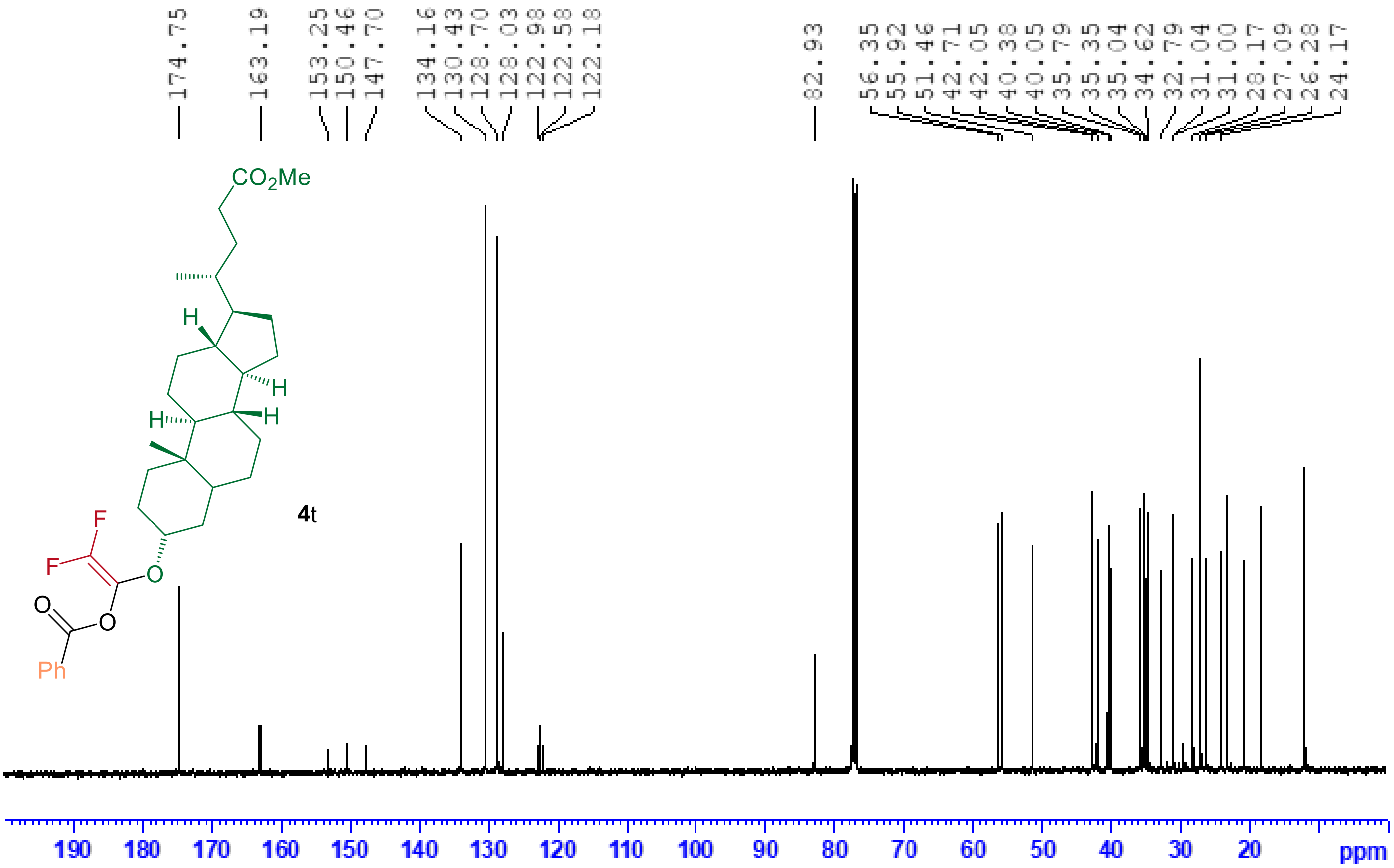

^{19}F NMR of **4t**

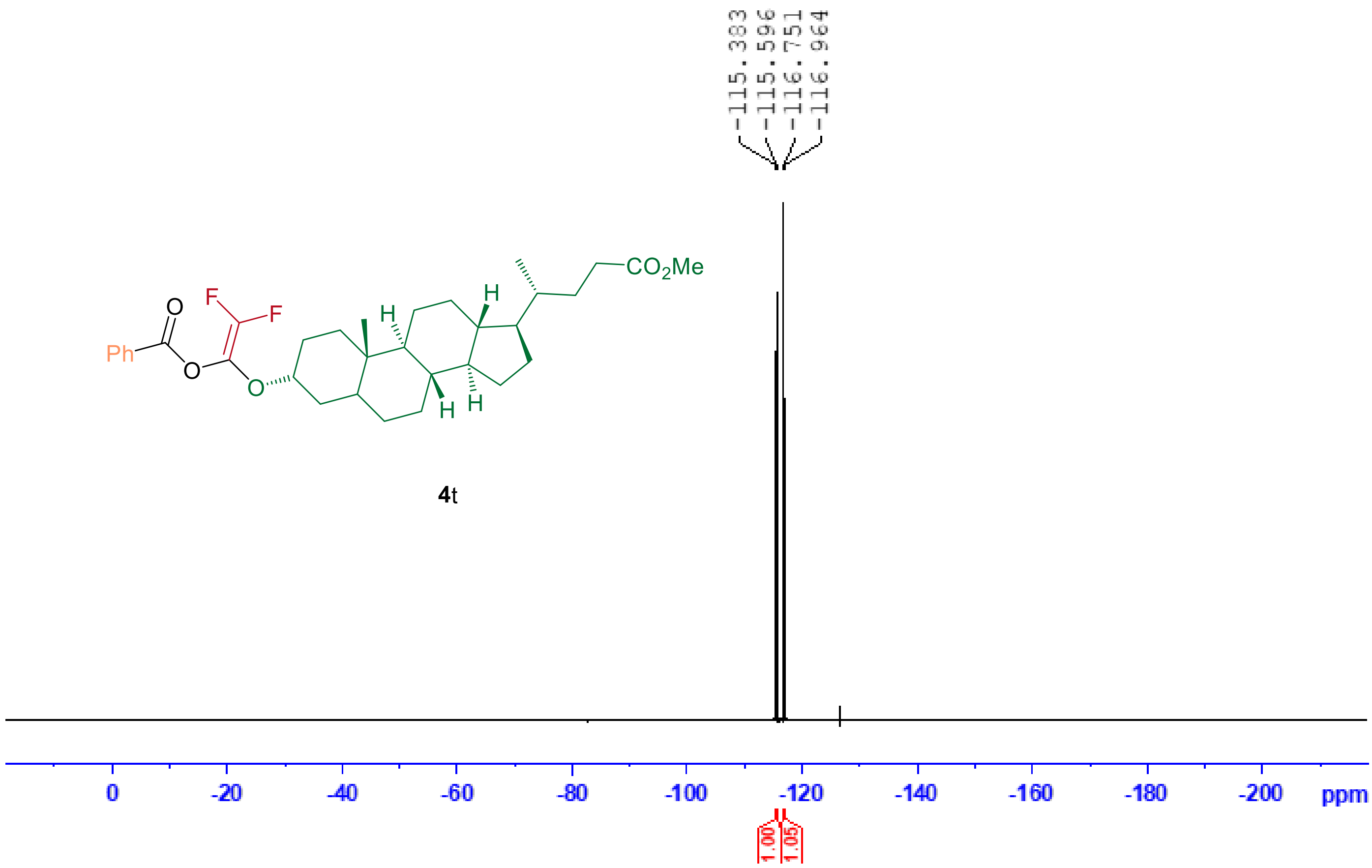
-115.383
-115.596
-116.751
-116.964
Ph
O
O
F
F
O
H
H
H
H
CO2Me
4t
0
-20
-40
-60
-80
-100
-120
-140
-160
-180
-200
ppm
1.00
1.05

^{1}H NMR of **5a**

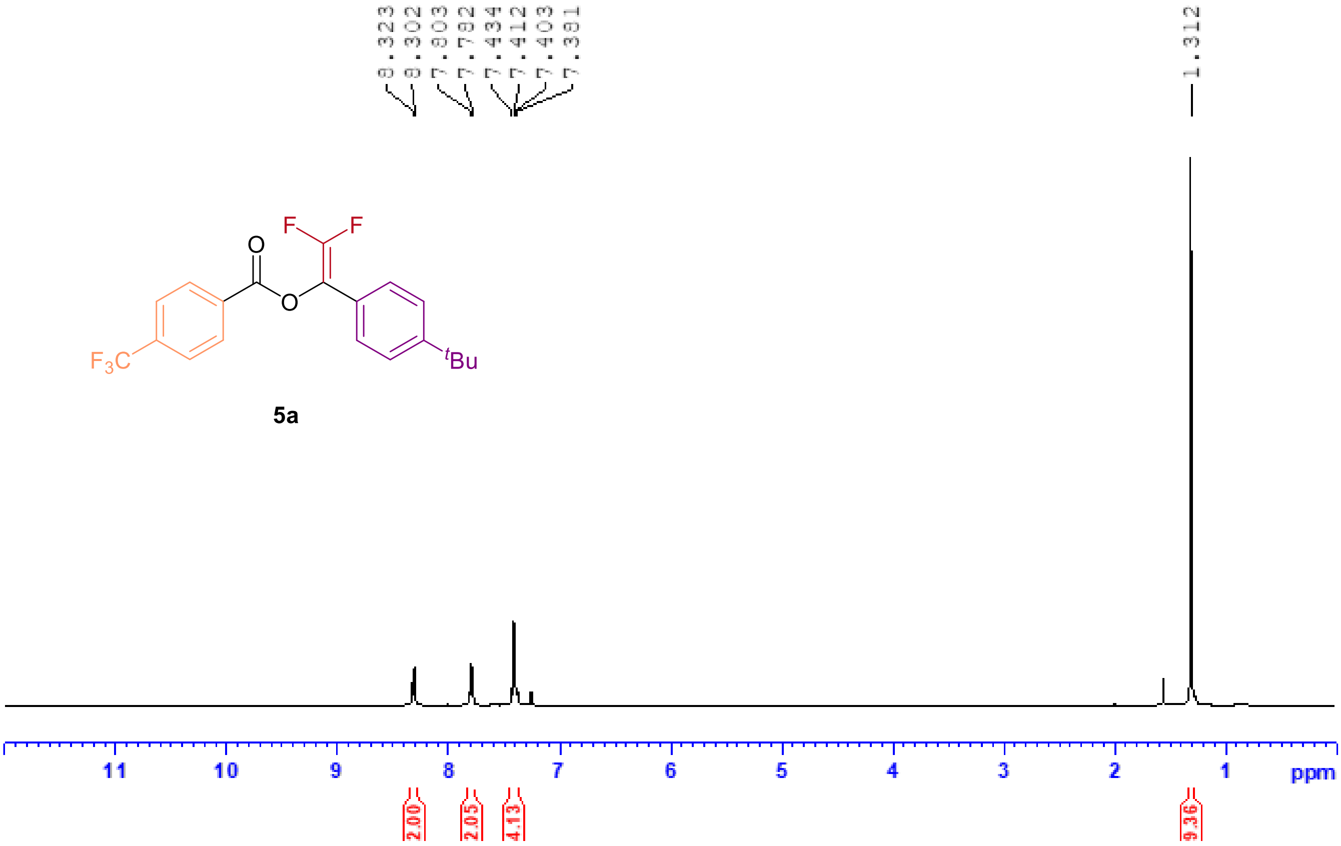

^{13}C NMR of **5a**

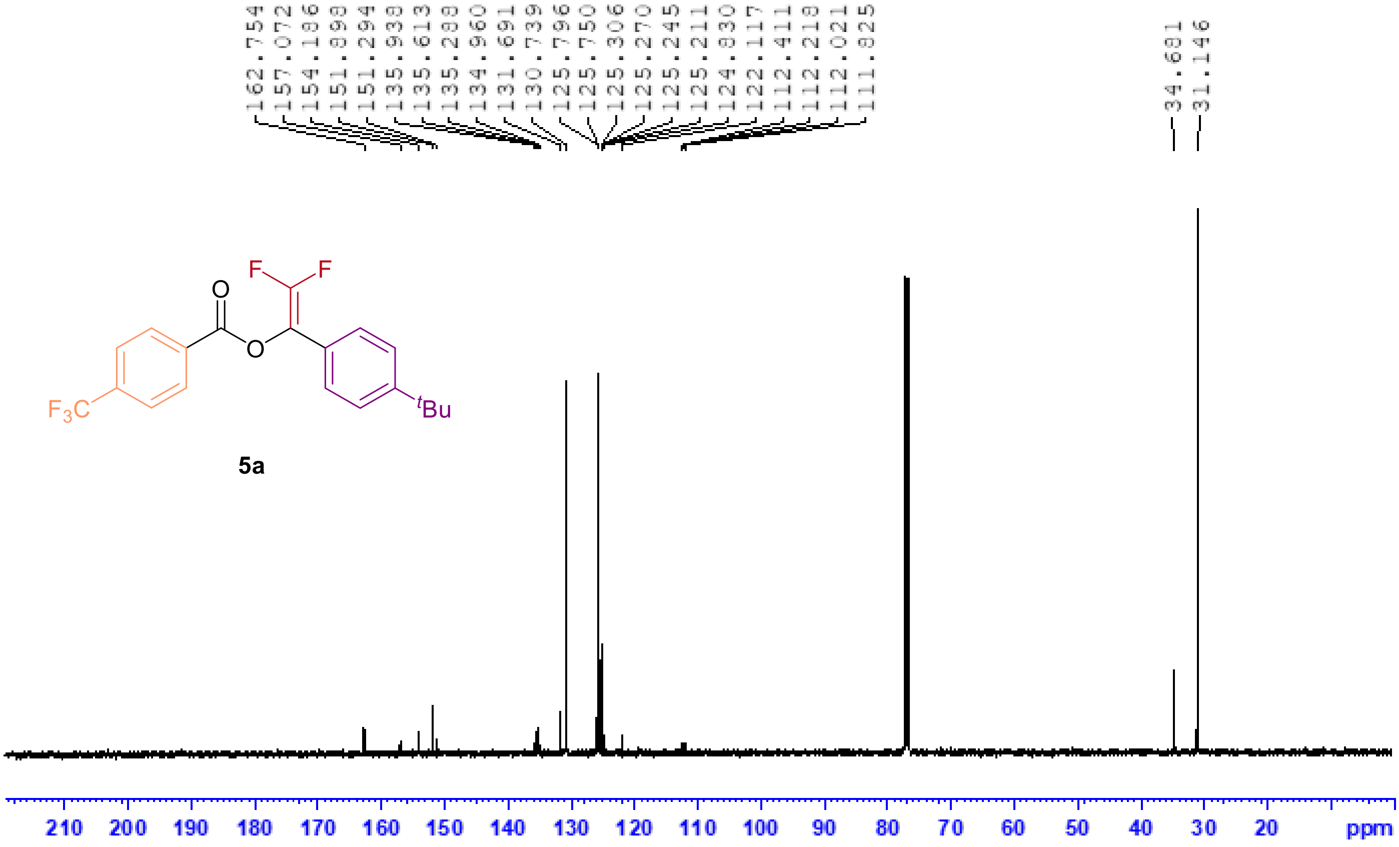

^{19}F NMR of **5a**

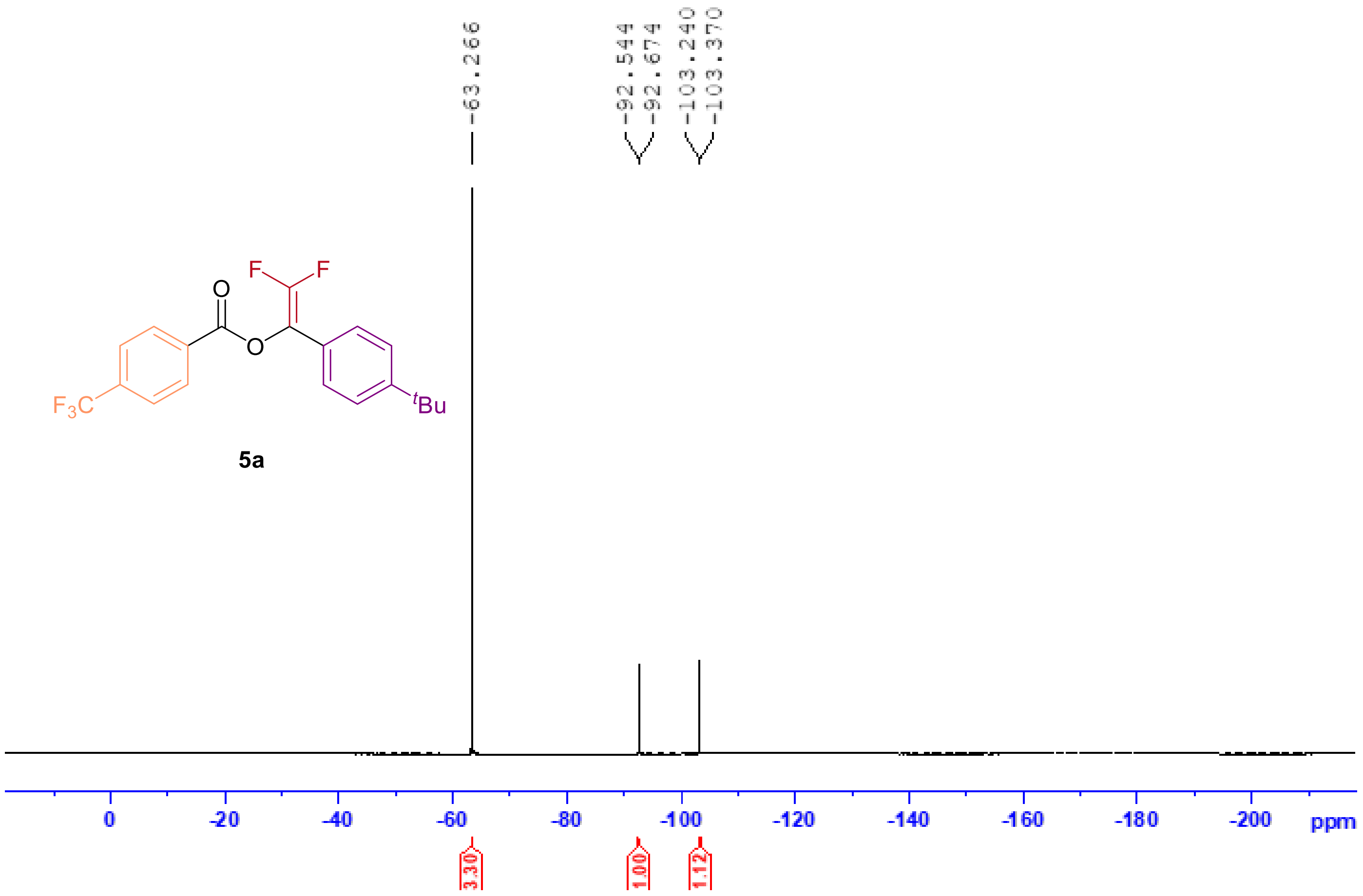

-63.266
-92.544
-92.674
-103.240
-103.370
F
F
O
O
F3C
tBu
5a
0
-20
-40
-60
-80
-100
-120
-140
-160
-180
-200
ppm
3.30
1.00
1.12

^{1}H NMR of **5b**

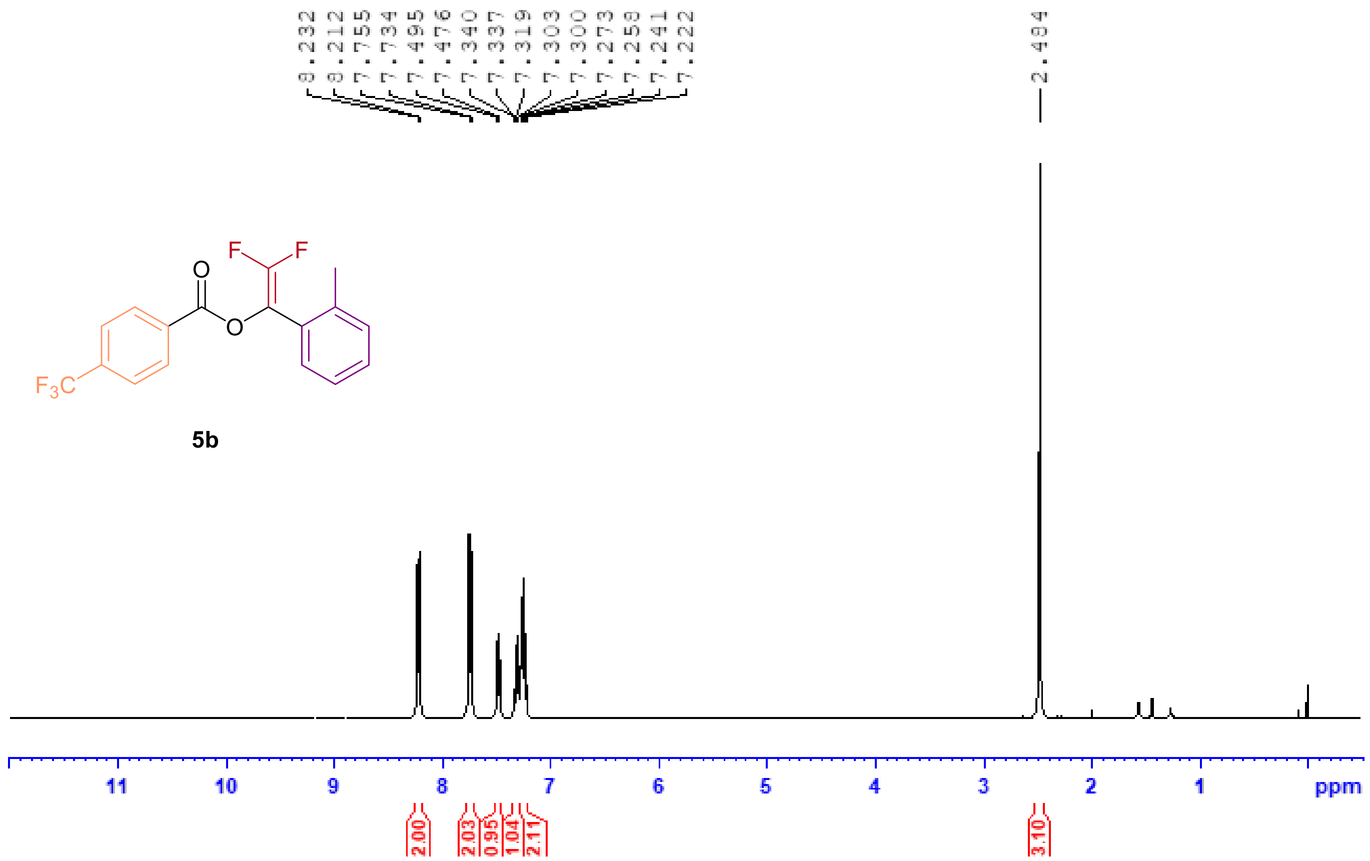

^{13}C NMR of **5b**

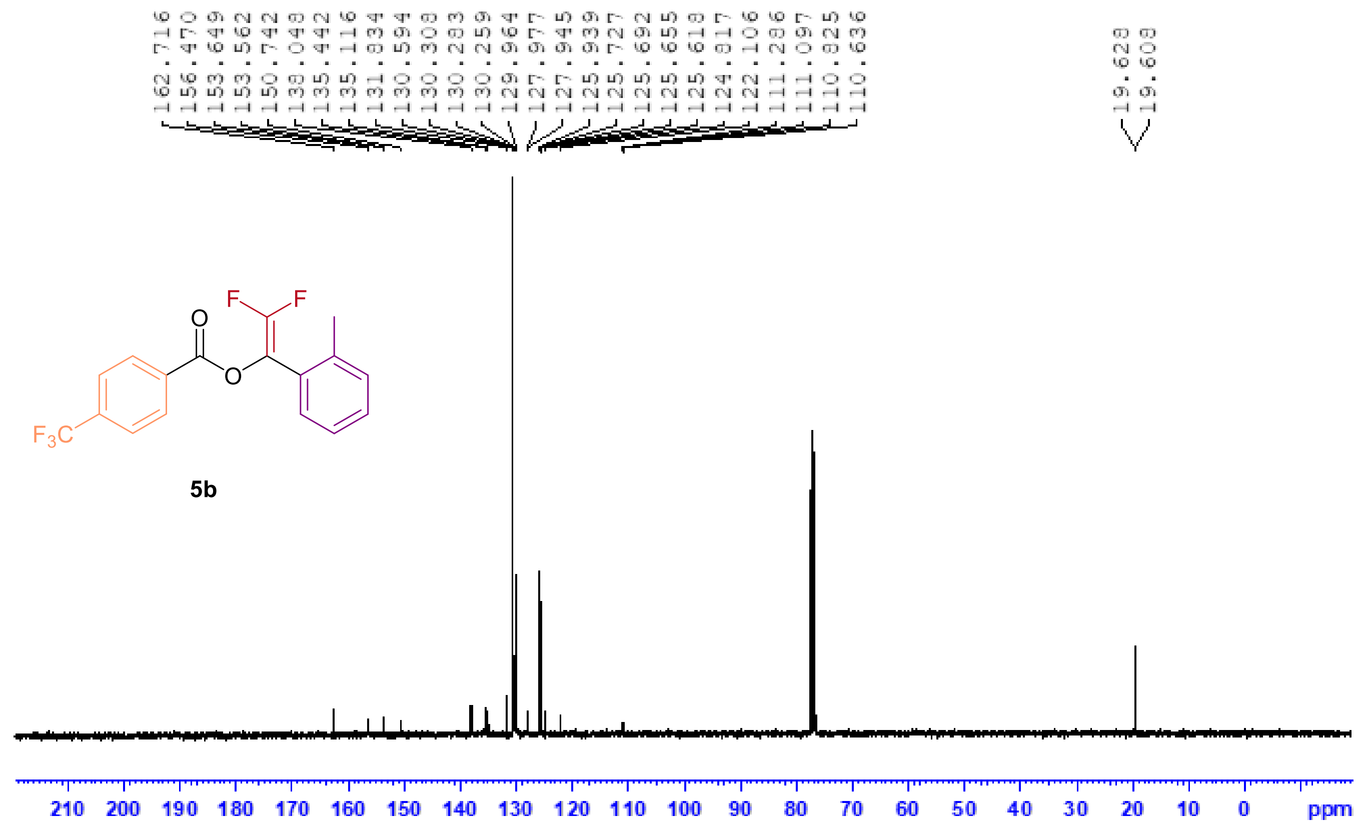

^{19}F NMR of **5b**

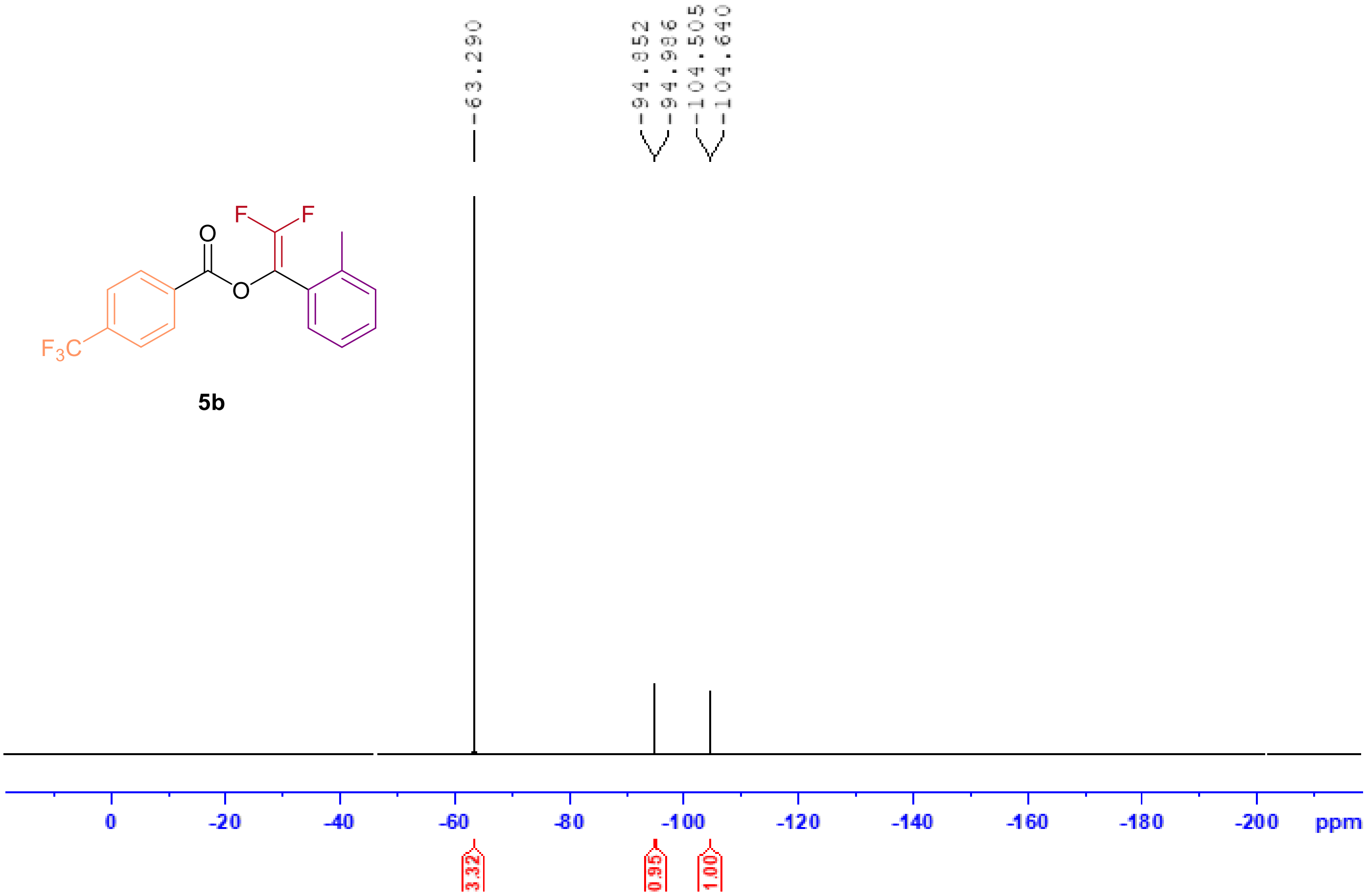

-63.290
-94.852
-94.986
-104.505
-104.640
O
F
F
O
F3C
5b
0
-20
-40
-60
-80
-100
-120
-140
-160
-180
-200
ppm
3.32
0.95
1.00

^{1}H NMR of **5c**

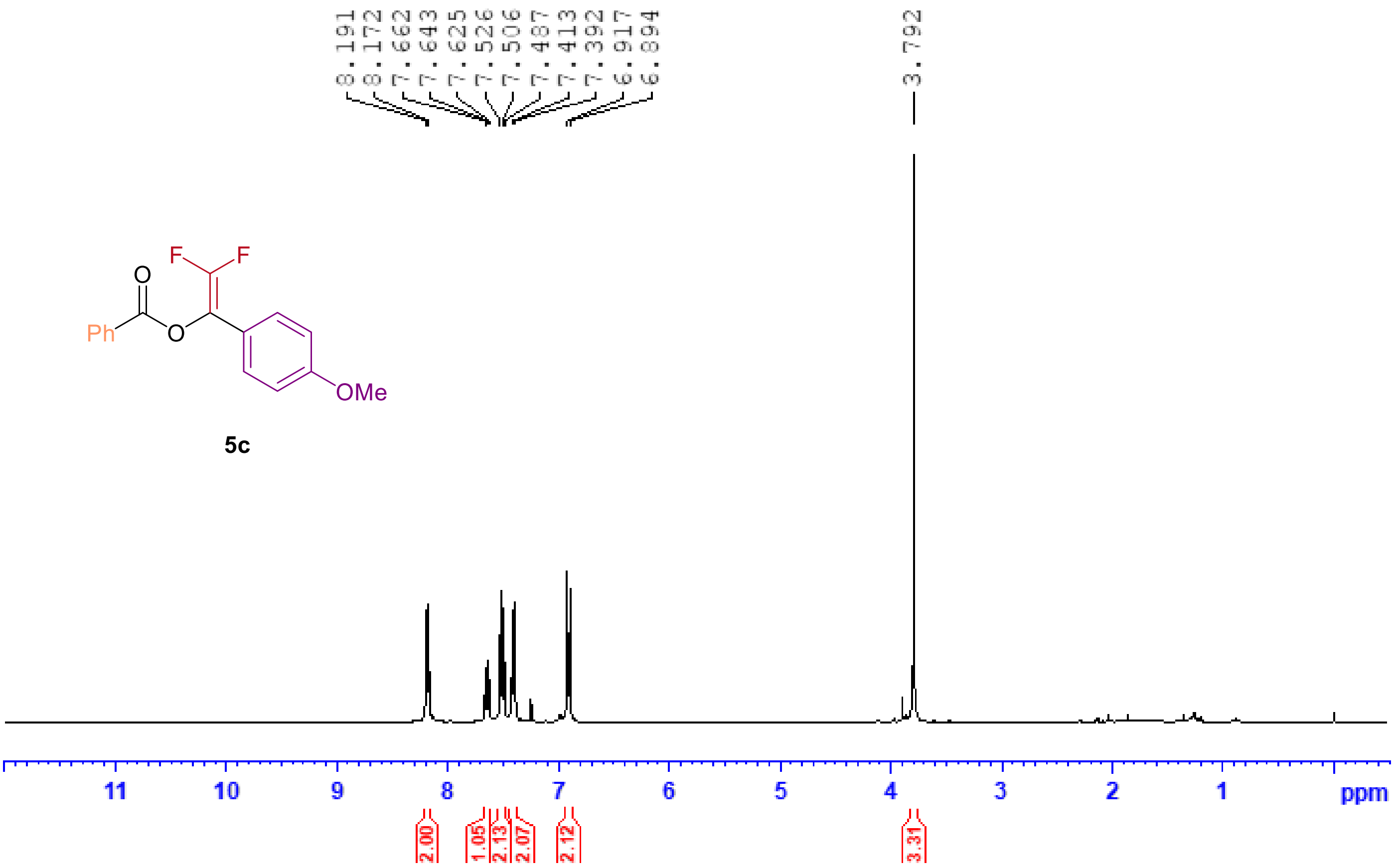

^{13}C NMR of **5c**

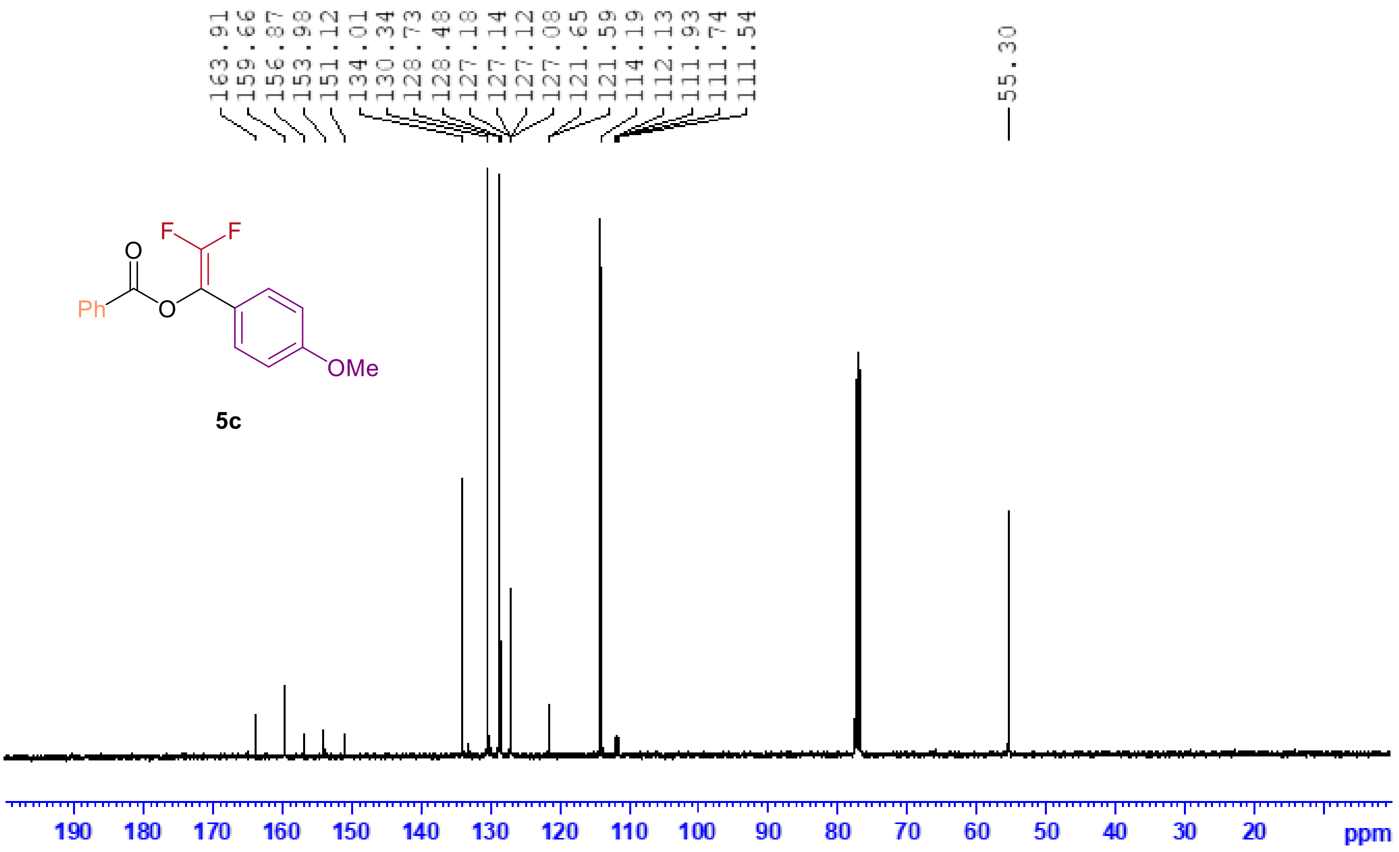

^{19}F NMR of **5c**

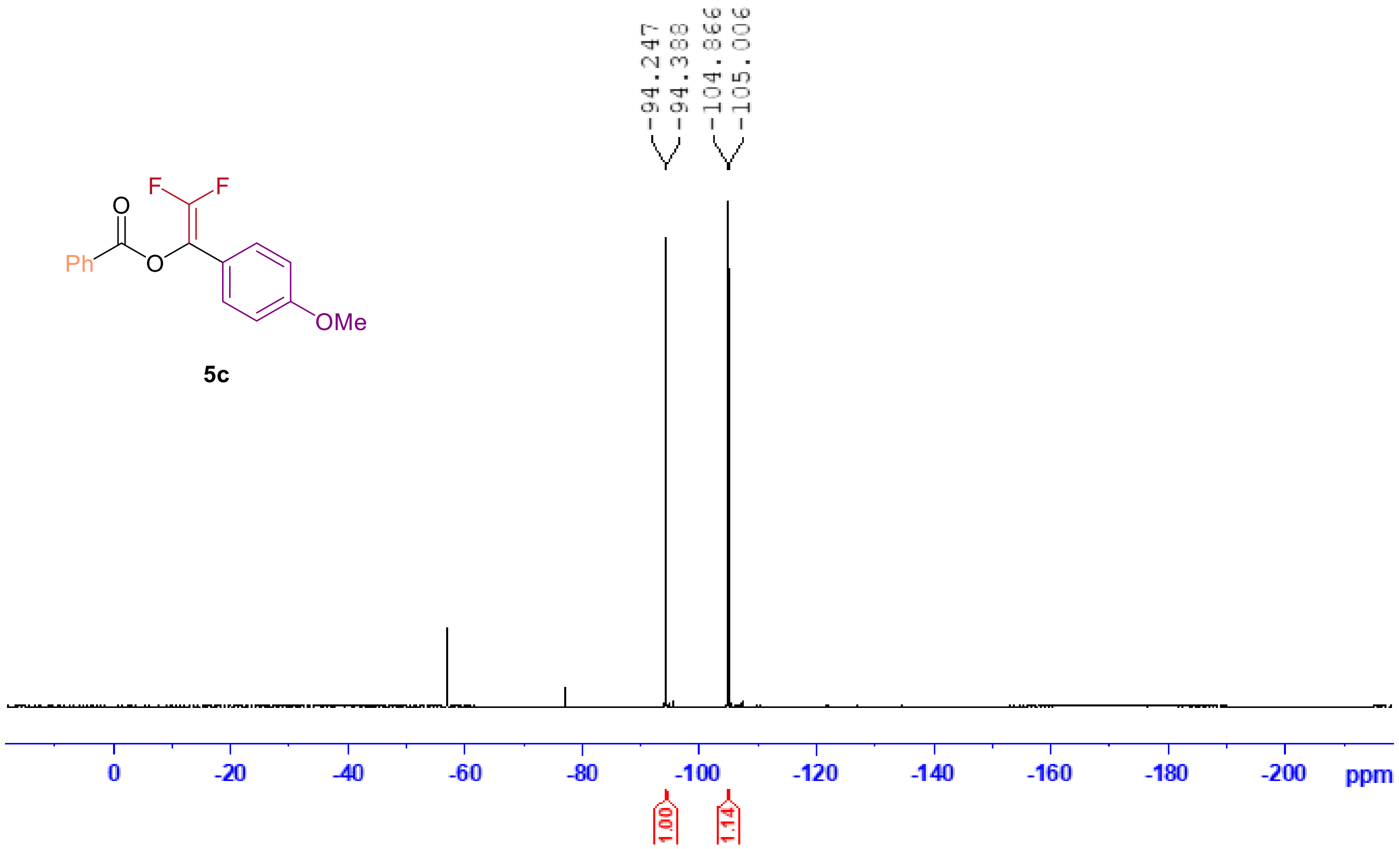

-94.247
-94.388
-104.866
-105.006
F
F
O
Ph
O
OMe
5c
0
-20
-40
-60
-80
-100
-120
-140
-160
-180
-200
ppm
1.00
1.14

^{1}H NMR of **7a**

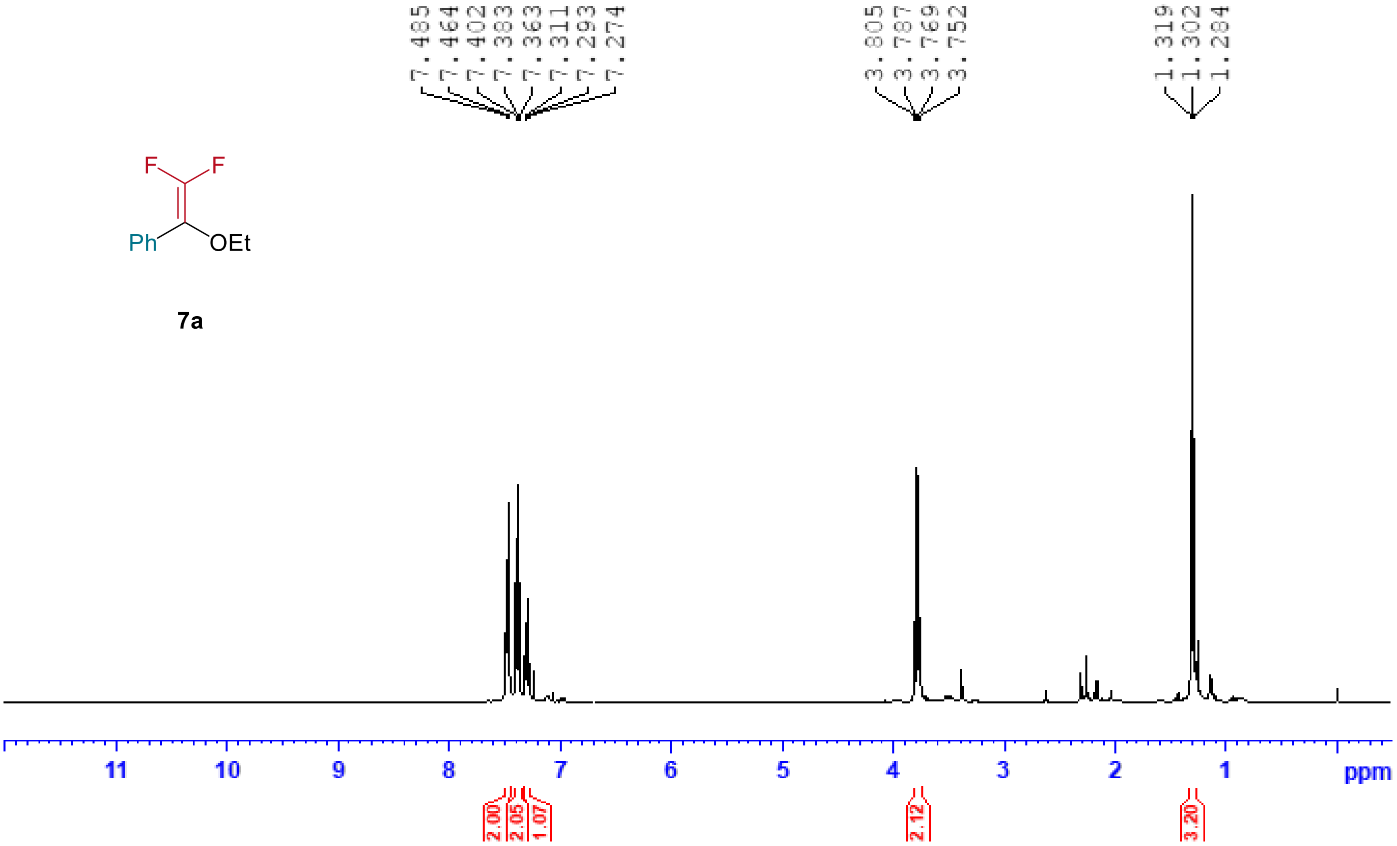

^{13}C NMR of **7a**

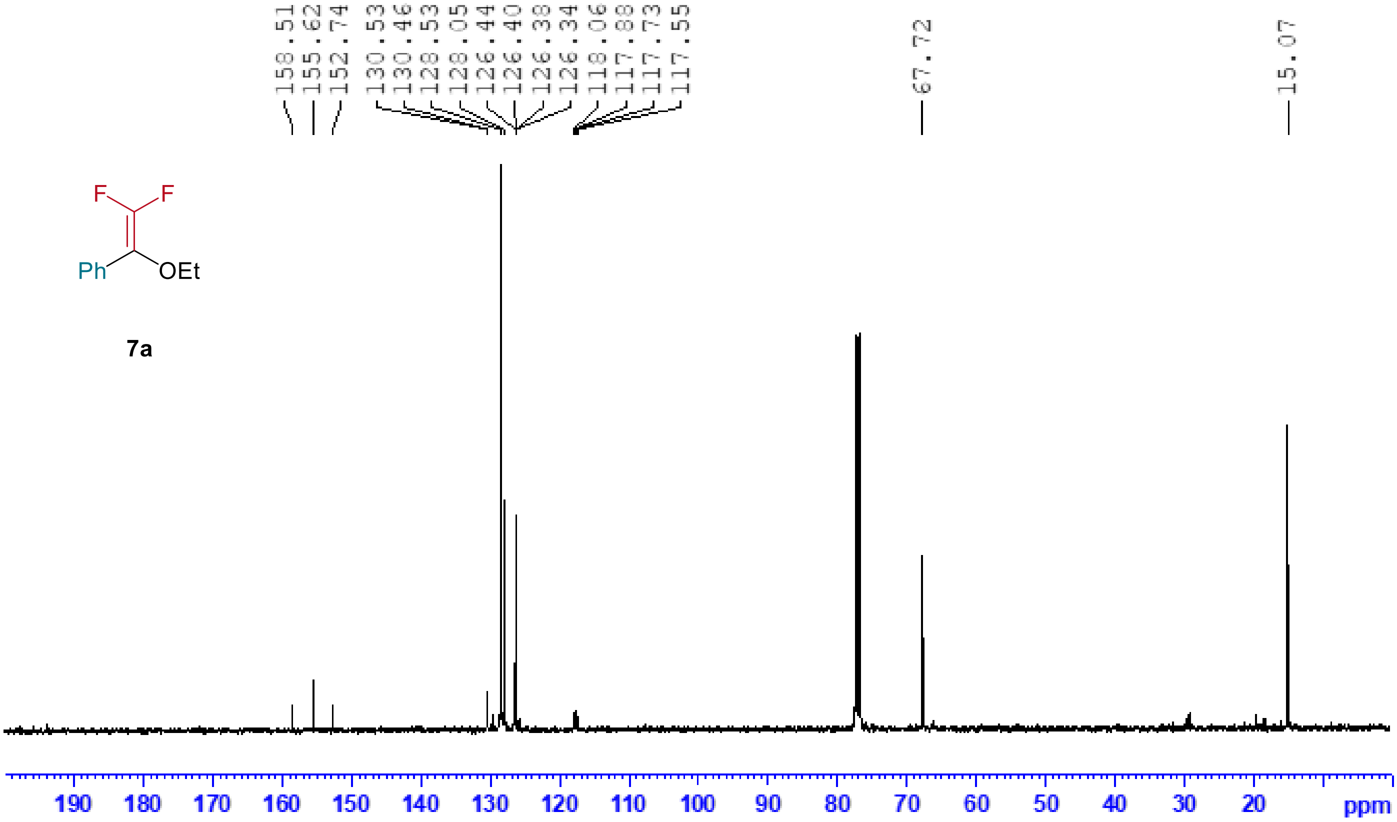

^{19}F NMR of **7a**

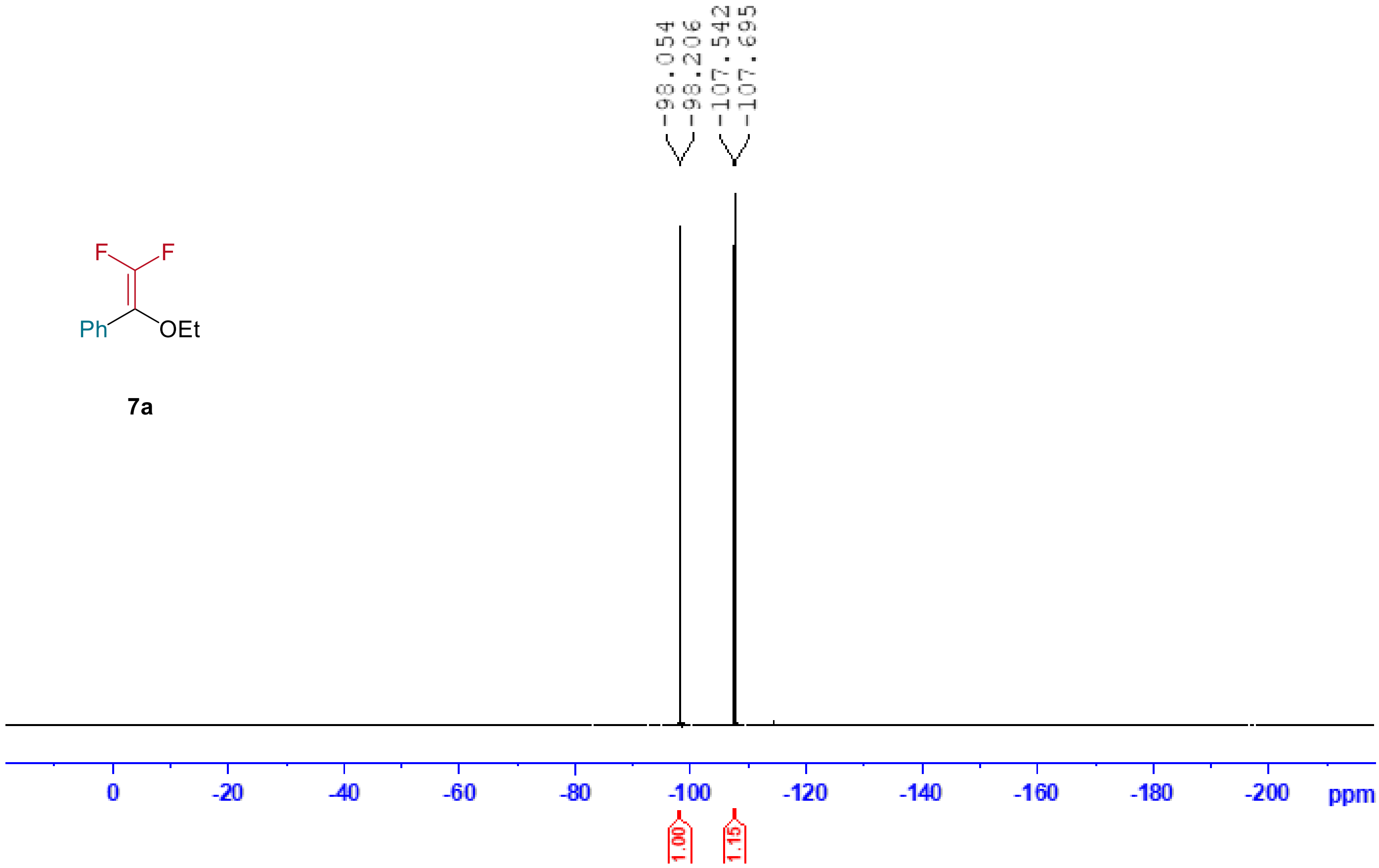
F
F
Ph
OEt
7a
-98.054
-98.206
-107.542
-107.695
0
-20
-40
-60
-80
-100
-120
-140
-160
-180
-200
ppm
1.00
1.15

^{1}H NMR of **7b**

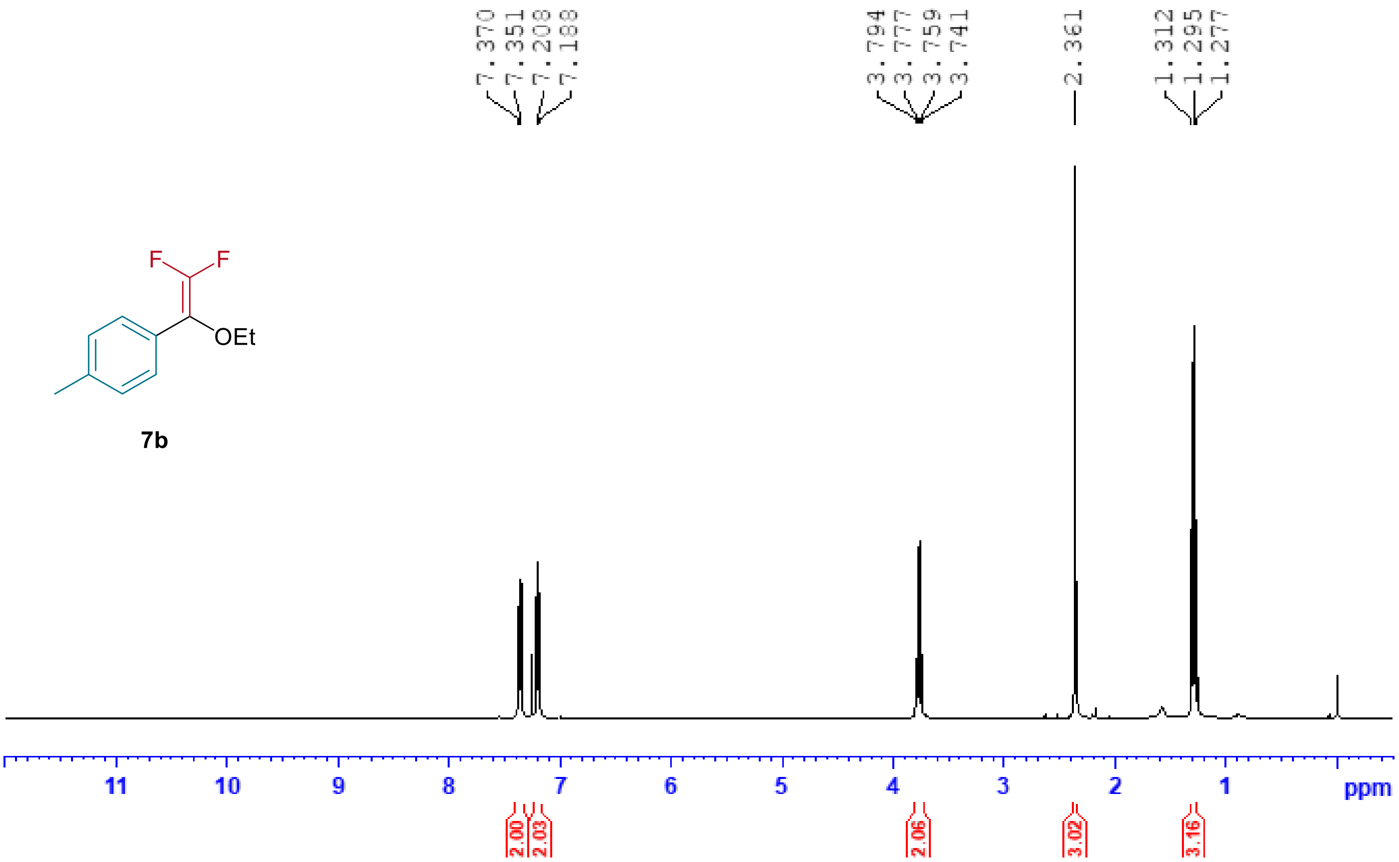

^{13}C NMR of **7b**

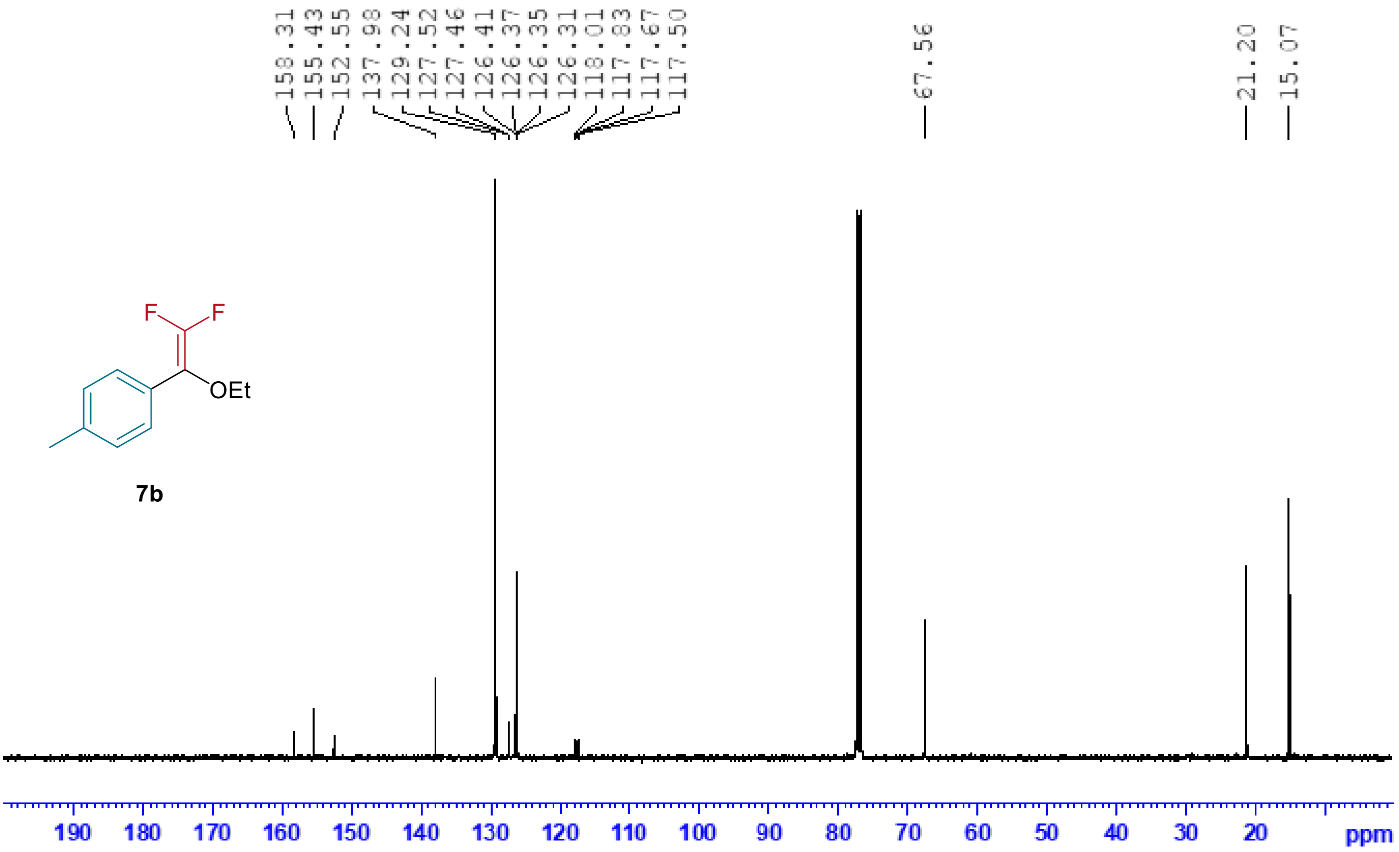

^{19}F NMR of **7b**

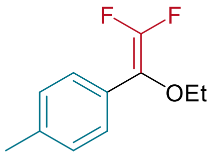

7b

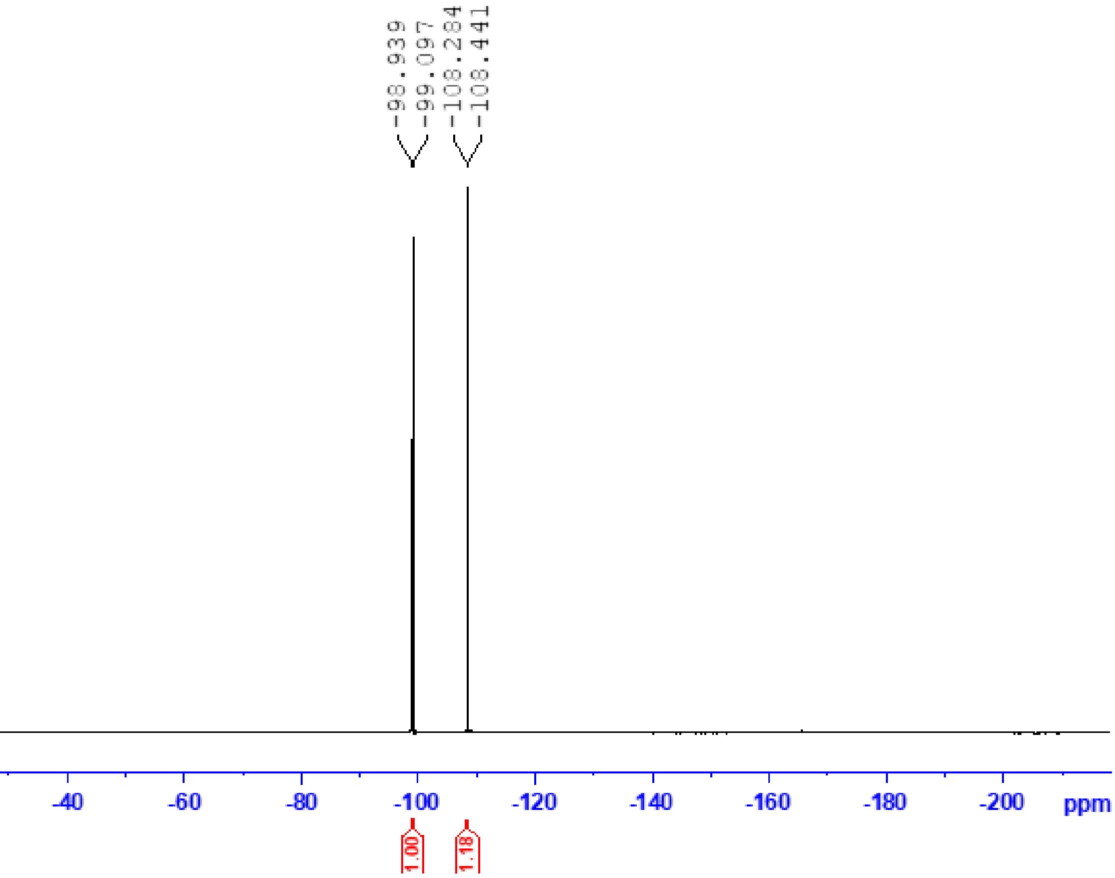

^{1}H NMR of **7c**

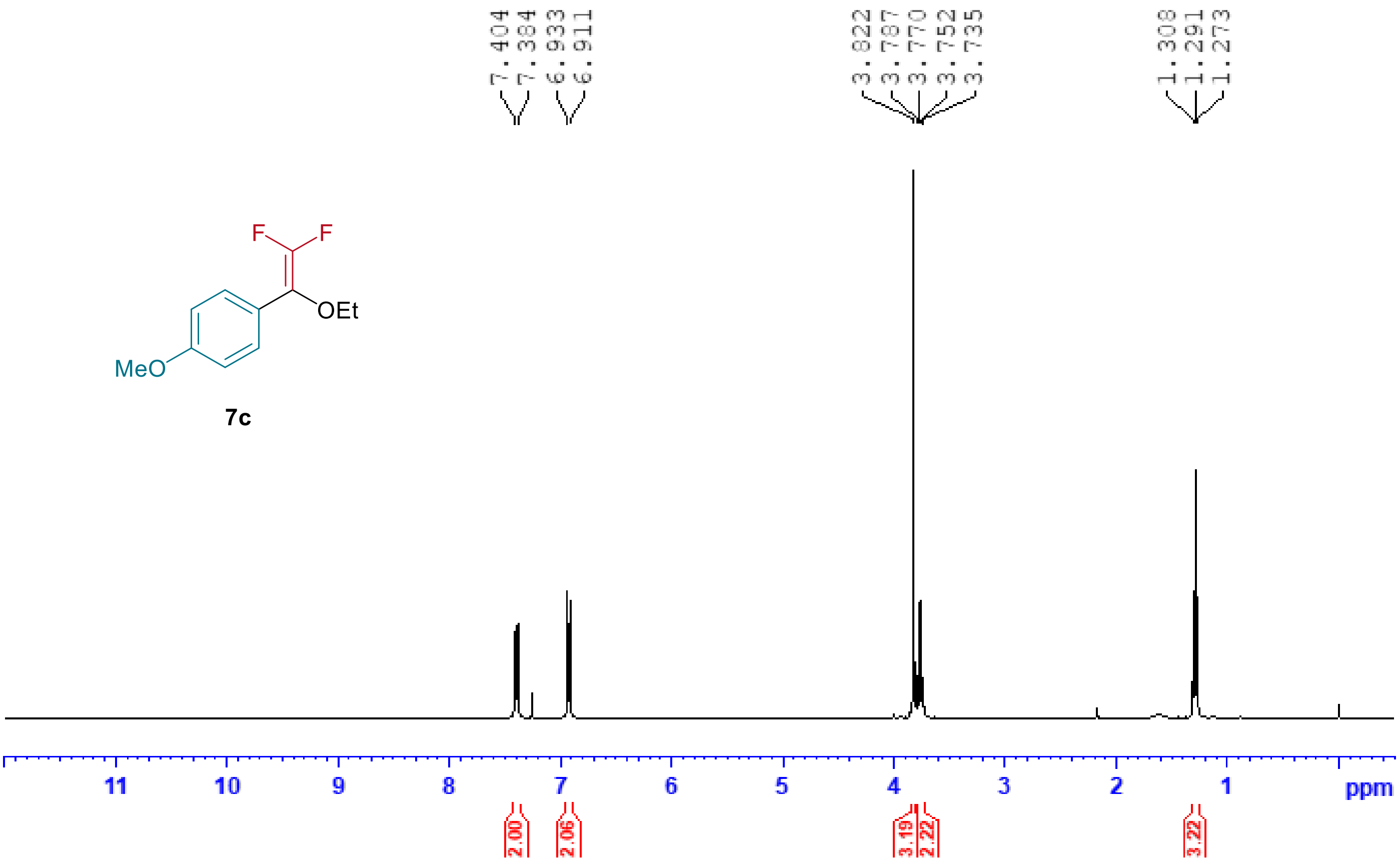

^{13}C NMR of **7c**

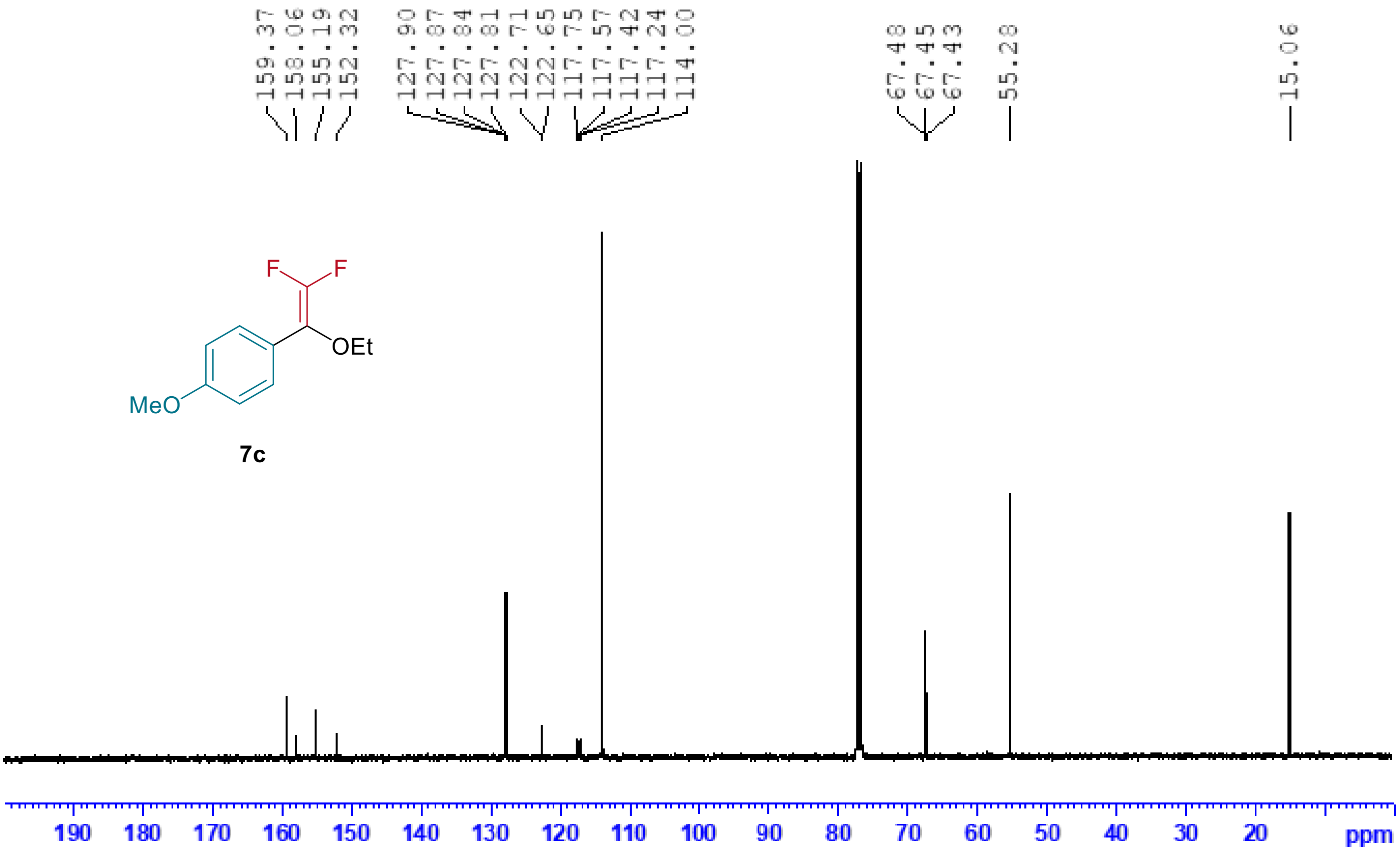

^{19}F NMR of **7c**

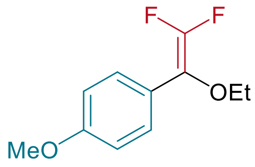

7c

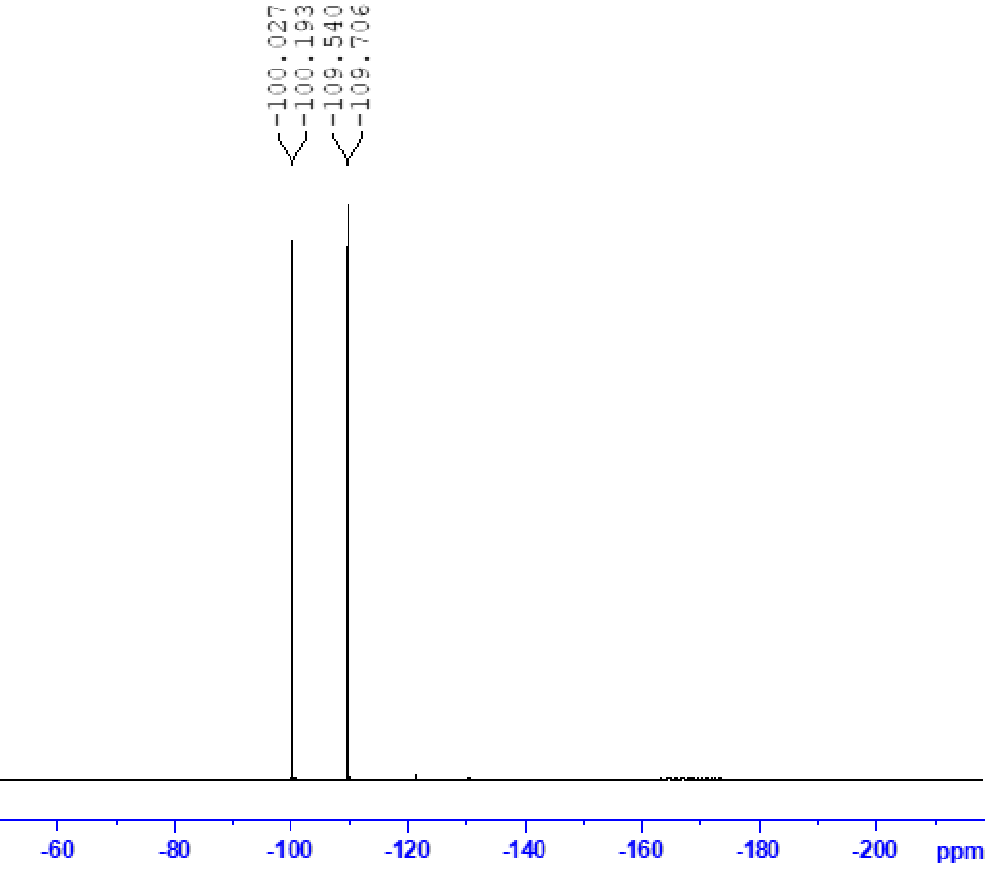

^{1}H NMR of **7d**

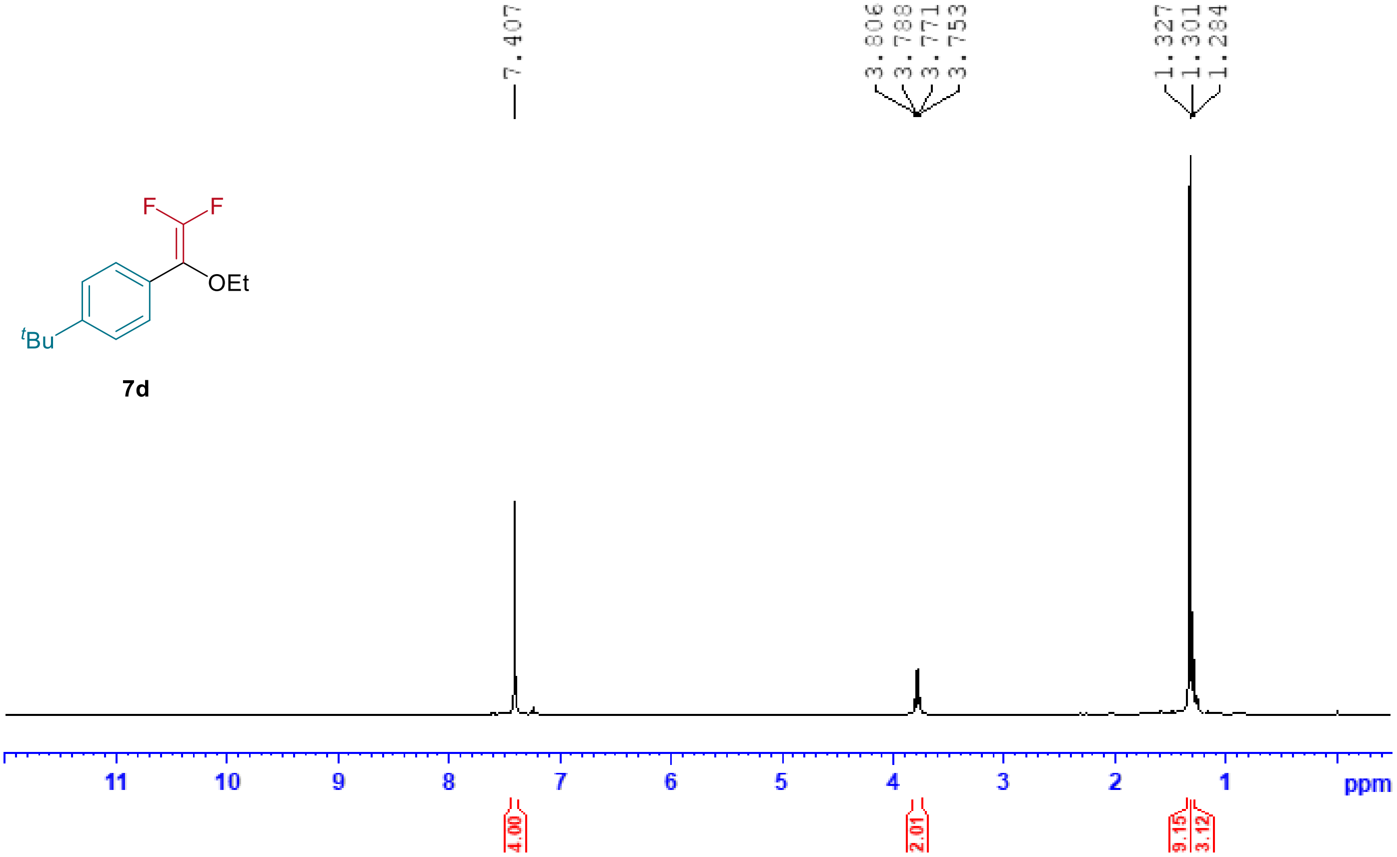

^{13}C NMR of **7d**

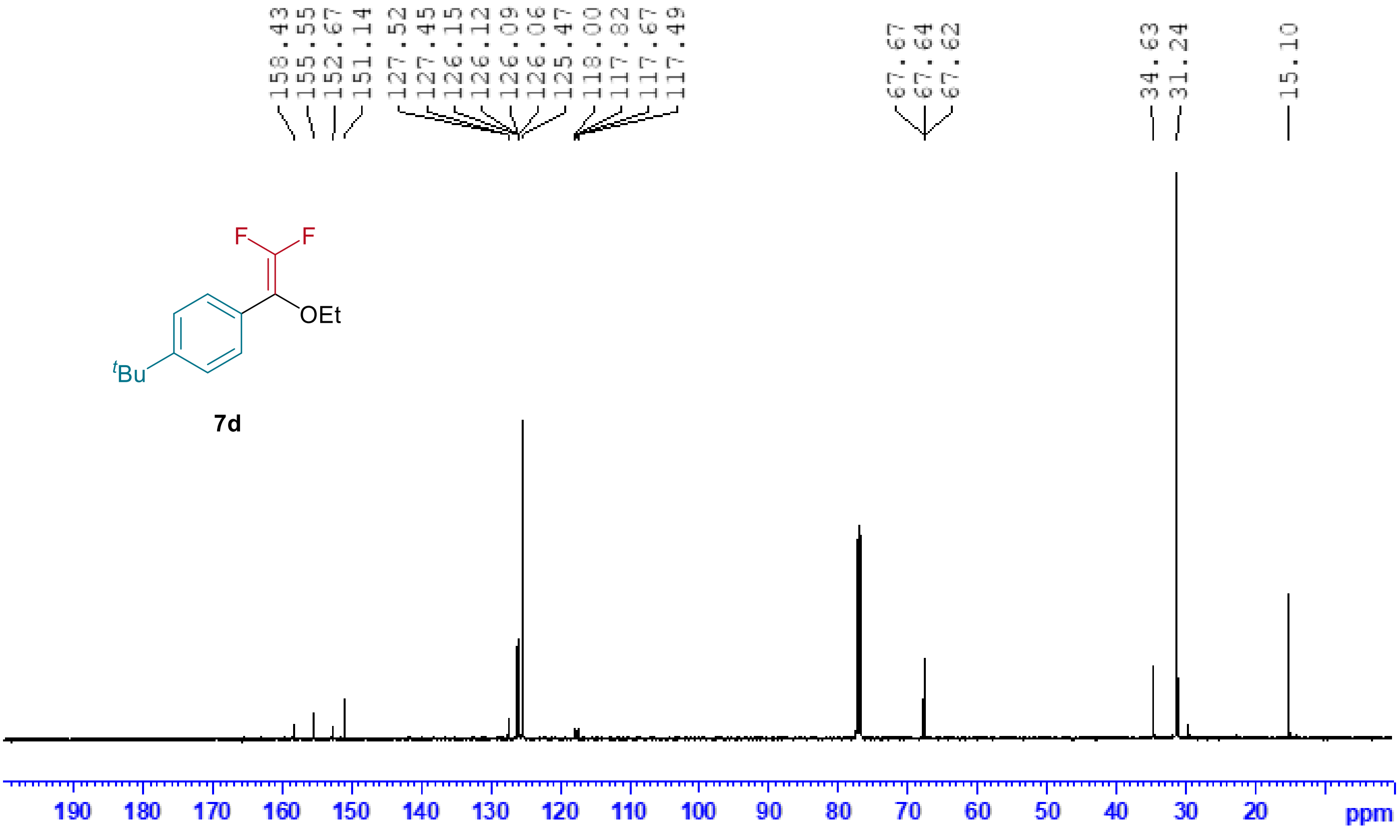

^{19}F NMR of **7d**

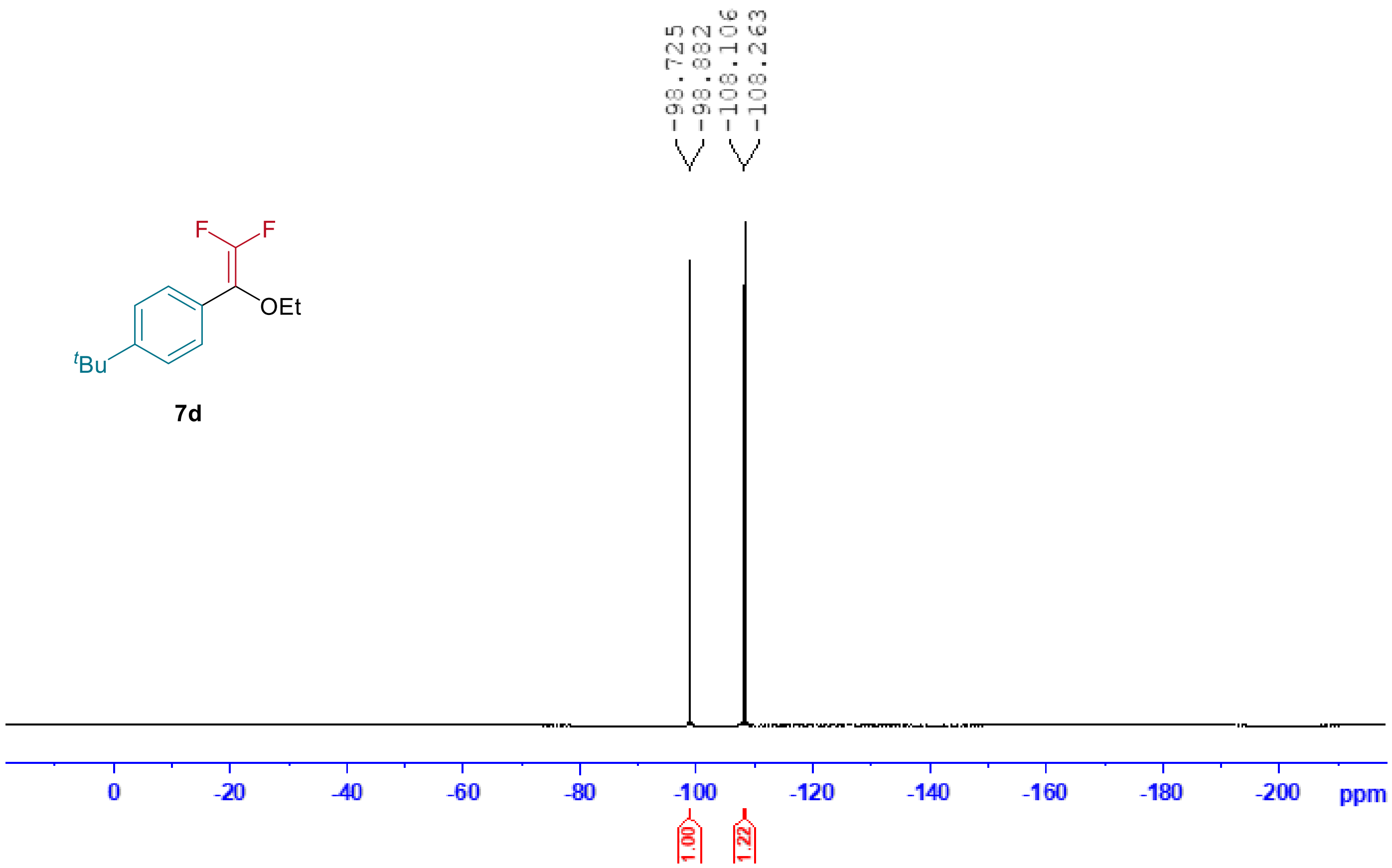

-98.725
-98.882
-108.106
-108.263
F
F
OEt
^{t}Bu
7d
0
-20
-40
-60
-80
-100
-120
-140
-160
-180
-200
ppm
1.00
1.22

^{1}H NMR of **7e**

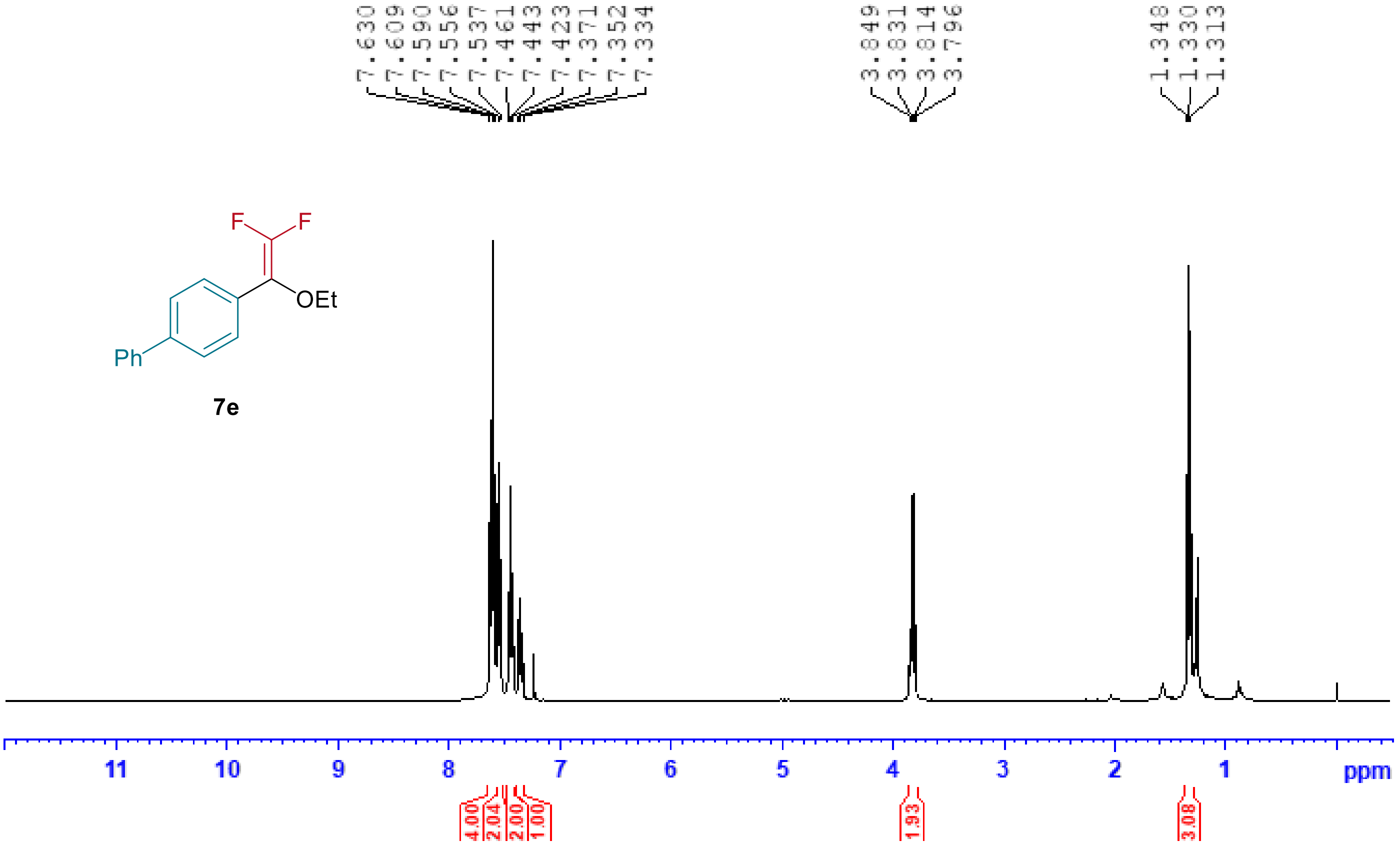

^{13}C NMR of **7e**

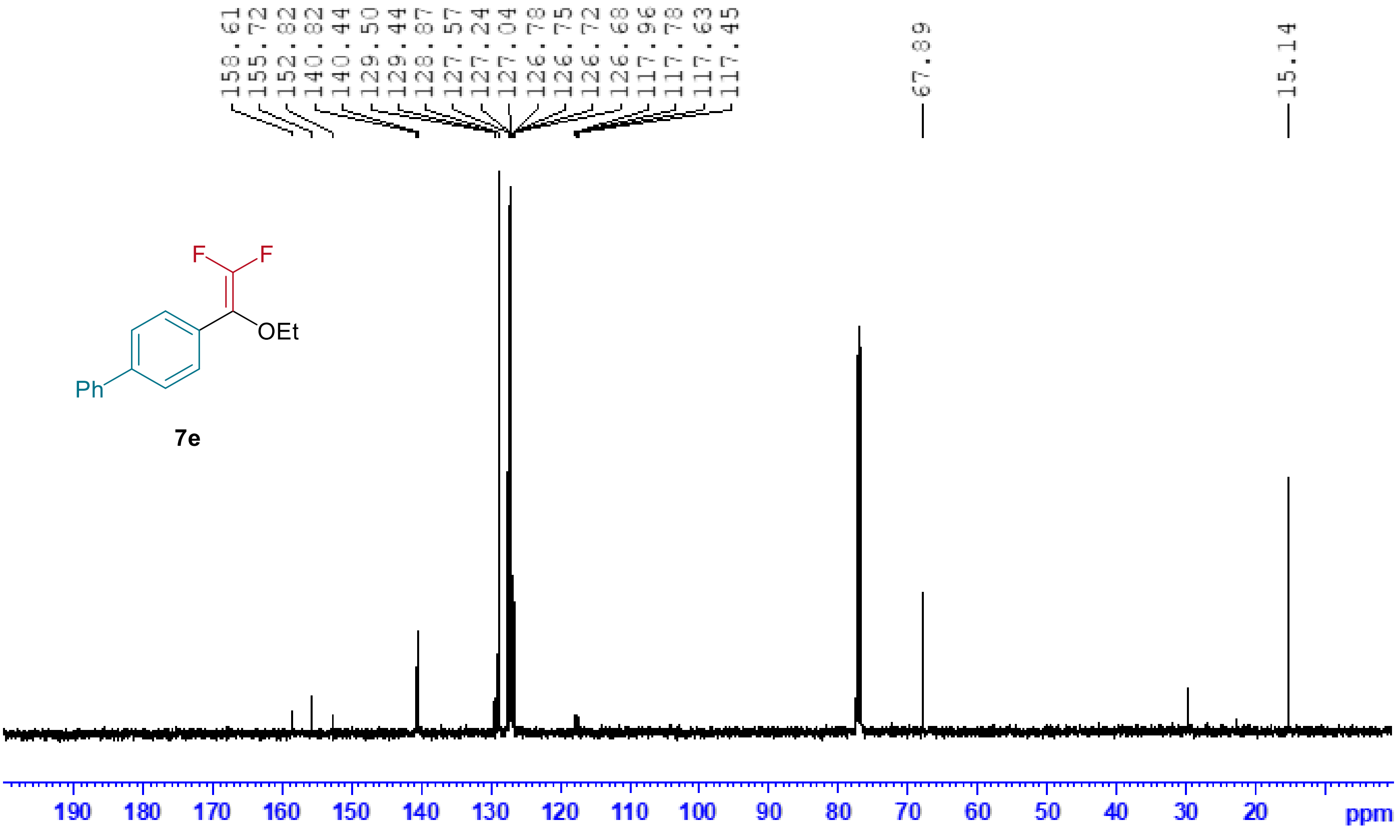

^{19}F NMR of **7e**

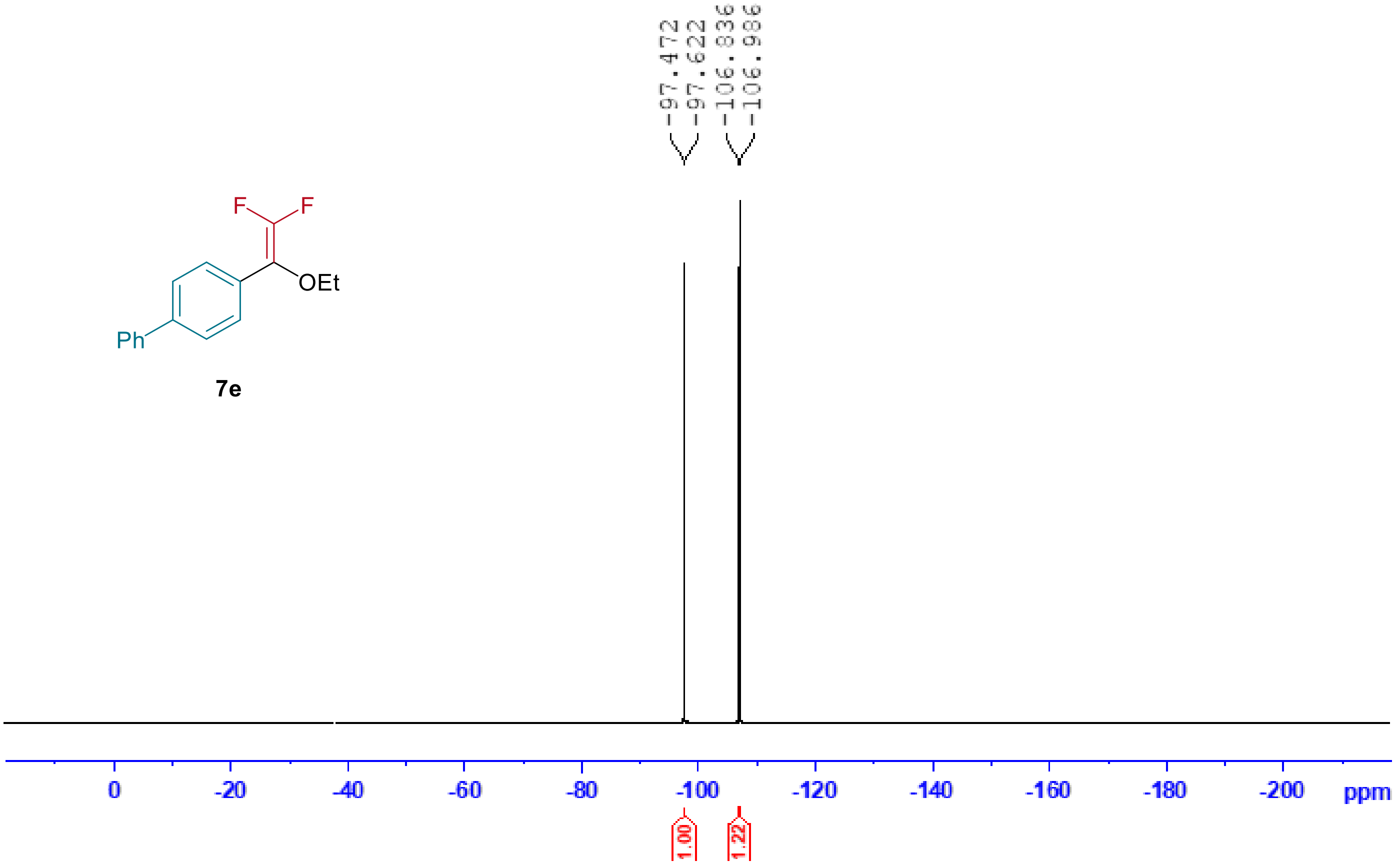
F
F
OEt
Ph
7e
-97.472
-97.622
-106.836
-106.986
0
-20
-40
-60
-80
-100
-120
-140
-160
-180
-200
ppm
1.00
1.22

^{1}H NMR of **7f**

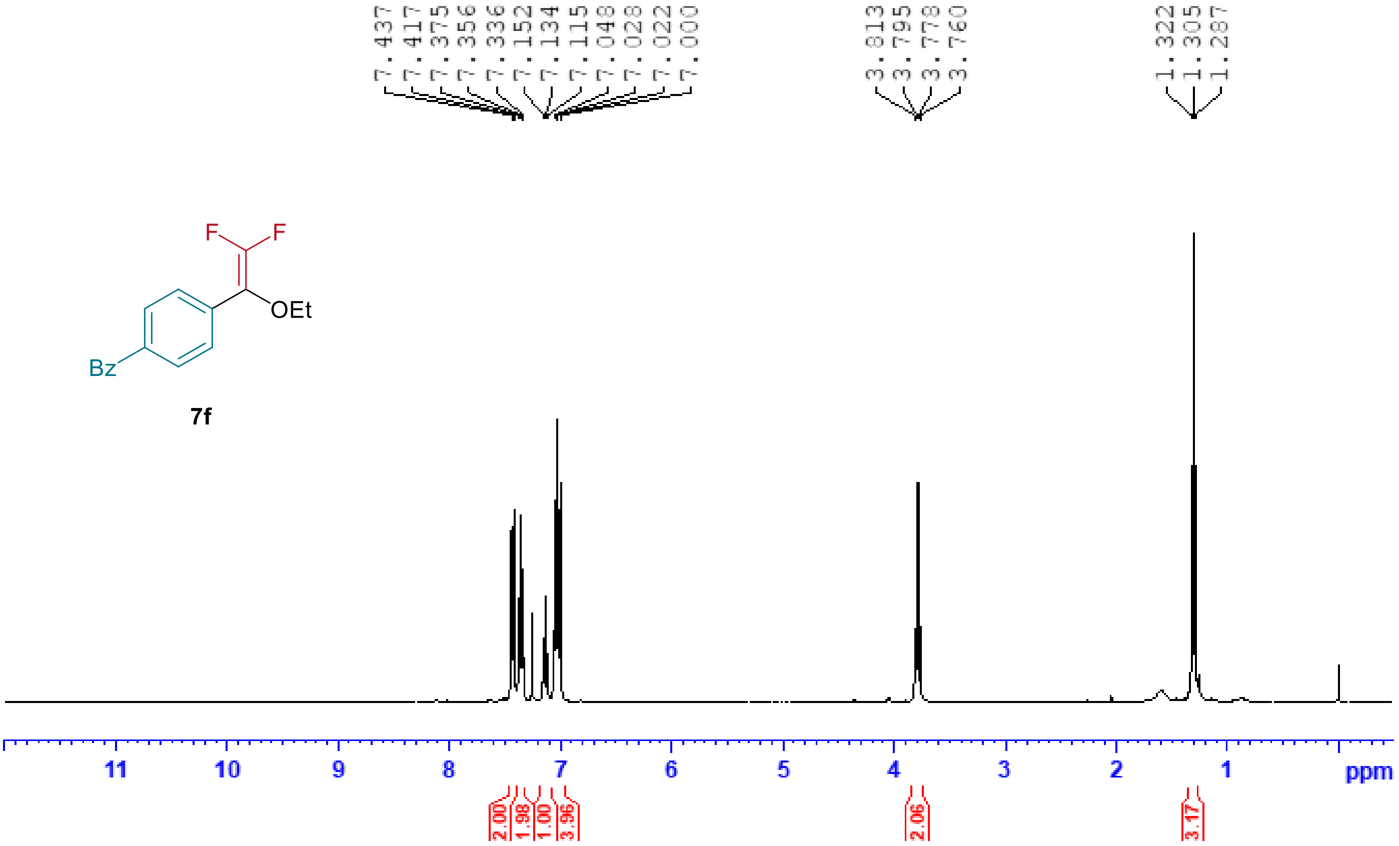

^{13}C NMR of **7f**

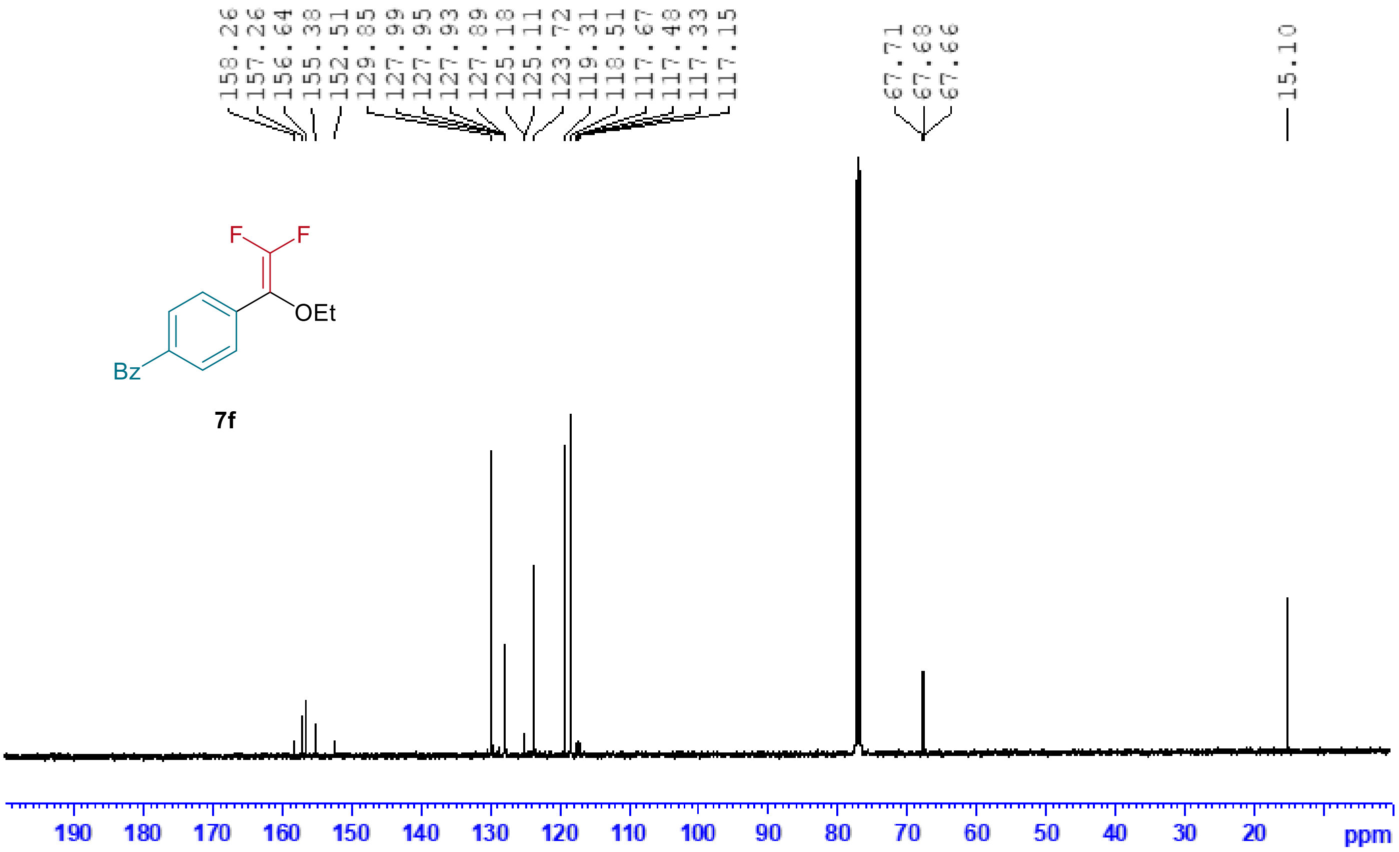

^{19}F NMR of **7f**

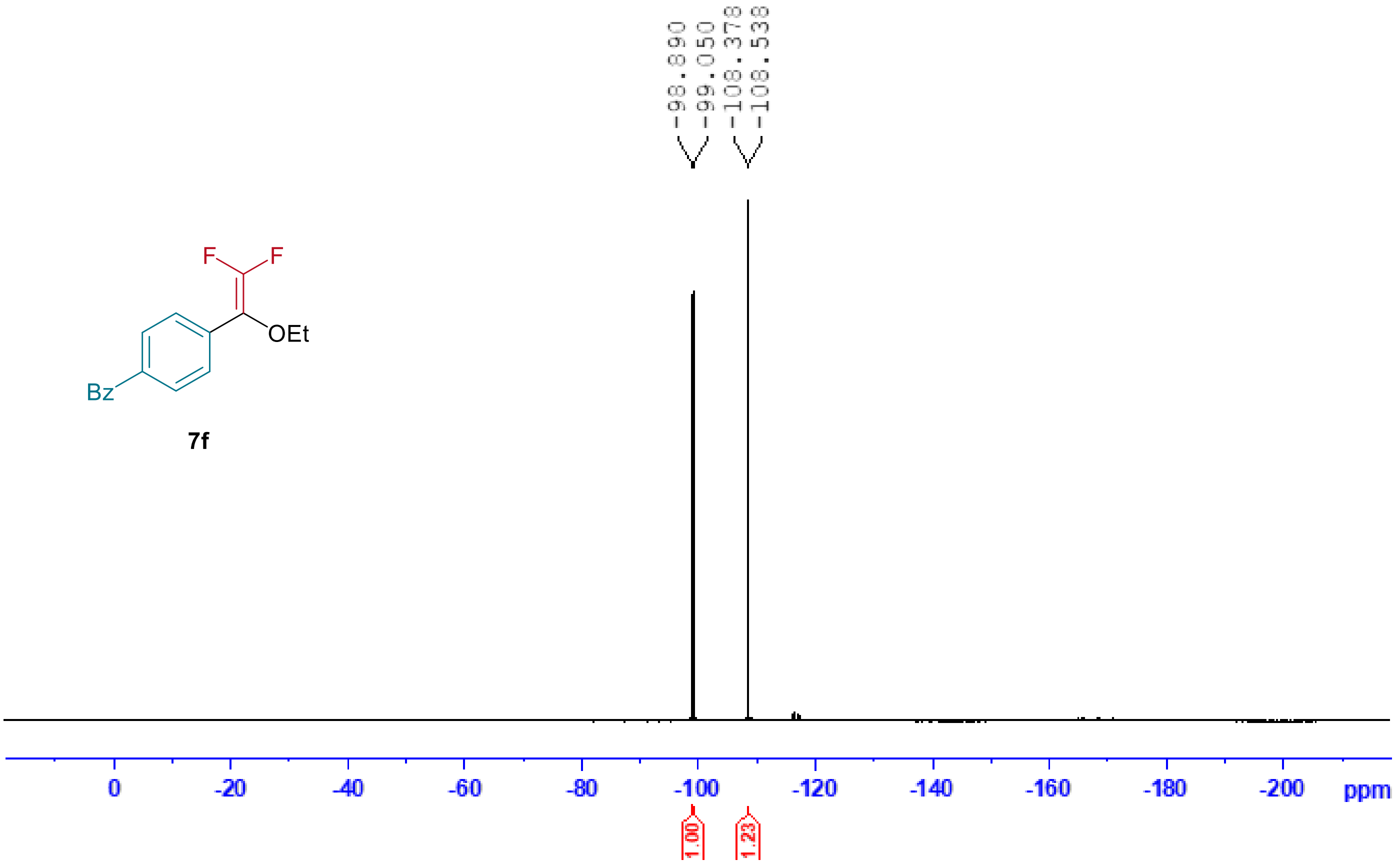

F
F
OEt
Bz
7f
-98.890
-99.050
-108.378
-108.538
0
-20
-40
-60
-80
-100
-120
-140
-160
-180
-200
ppm
1.00
1.23

^{1}H NMR of **7g**

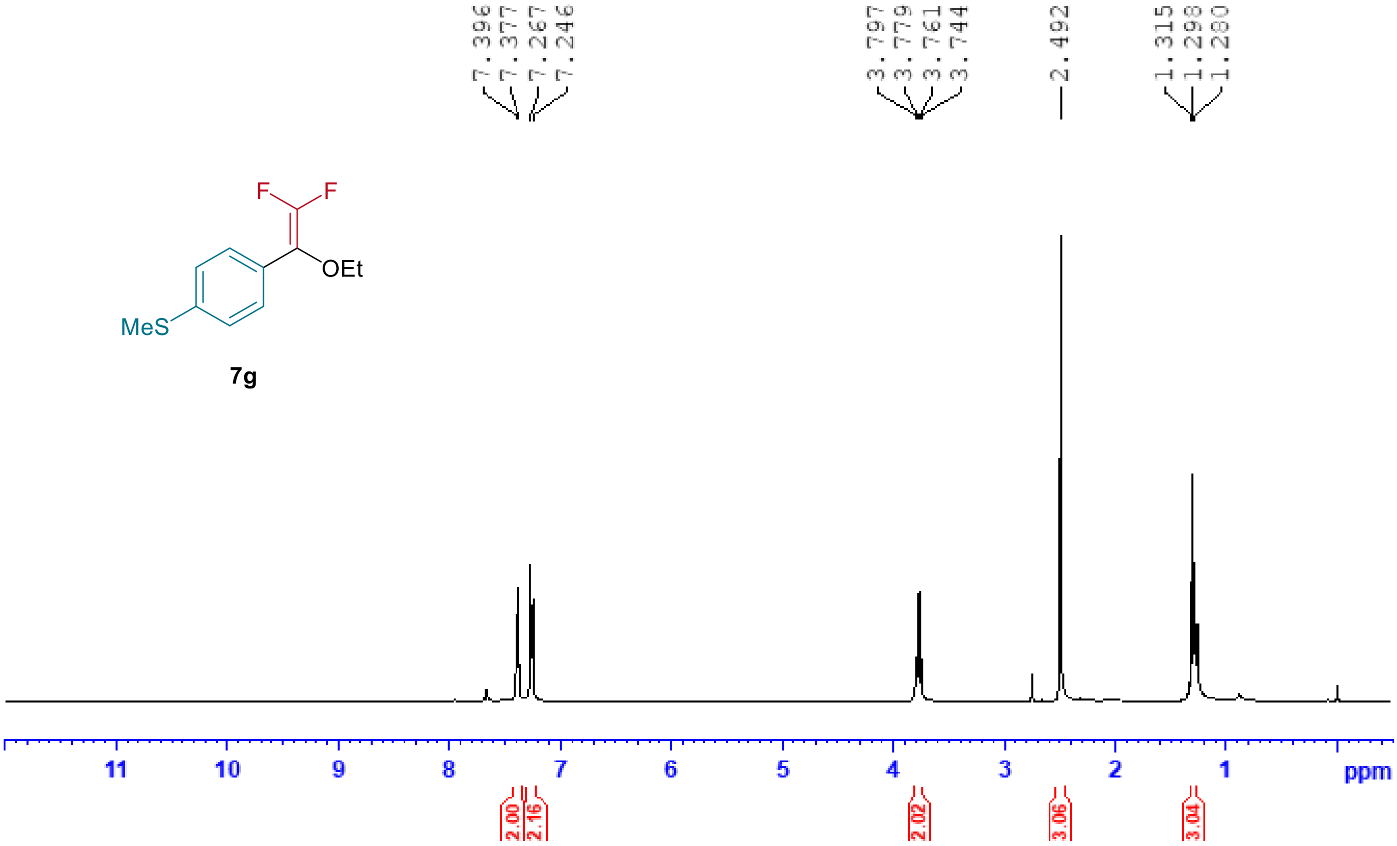

^{13}C NMR of **7g**

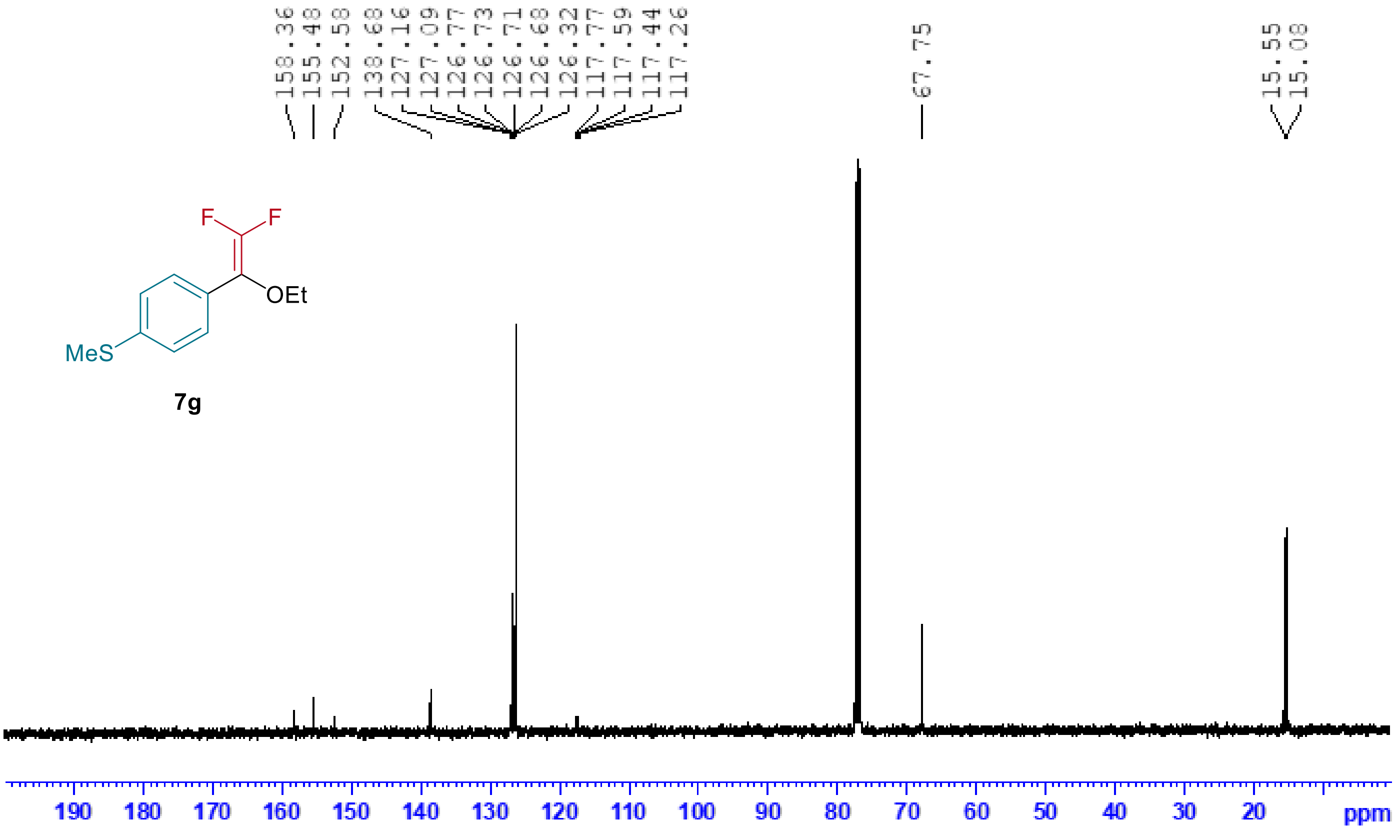

^{19}F NMR of **7g**

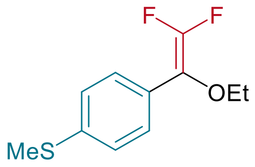

7g

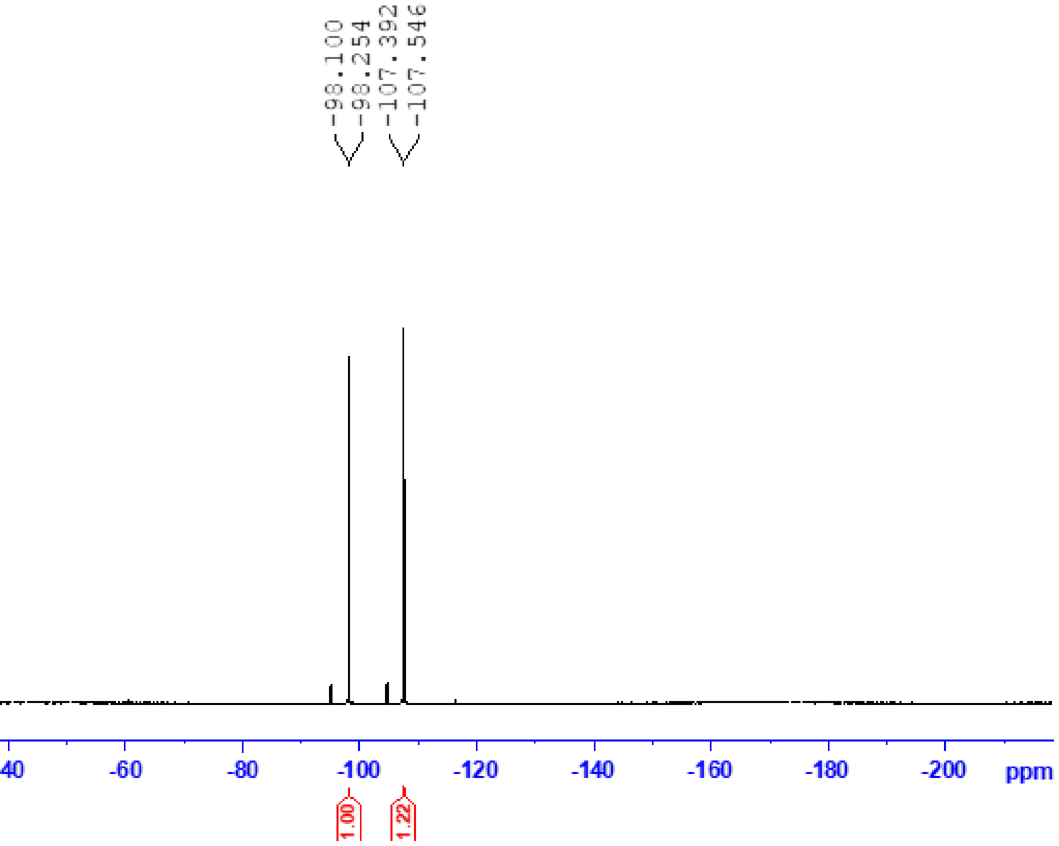

^{1}H NMR of **7h**

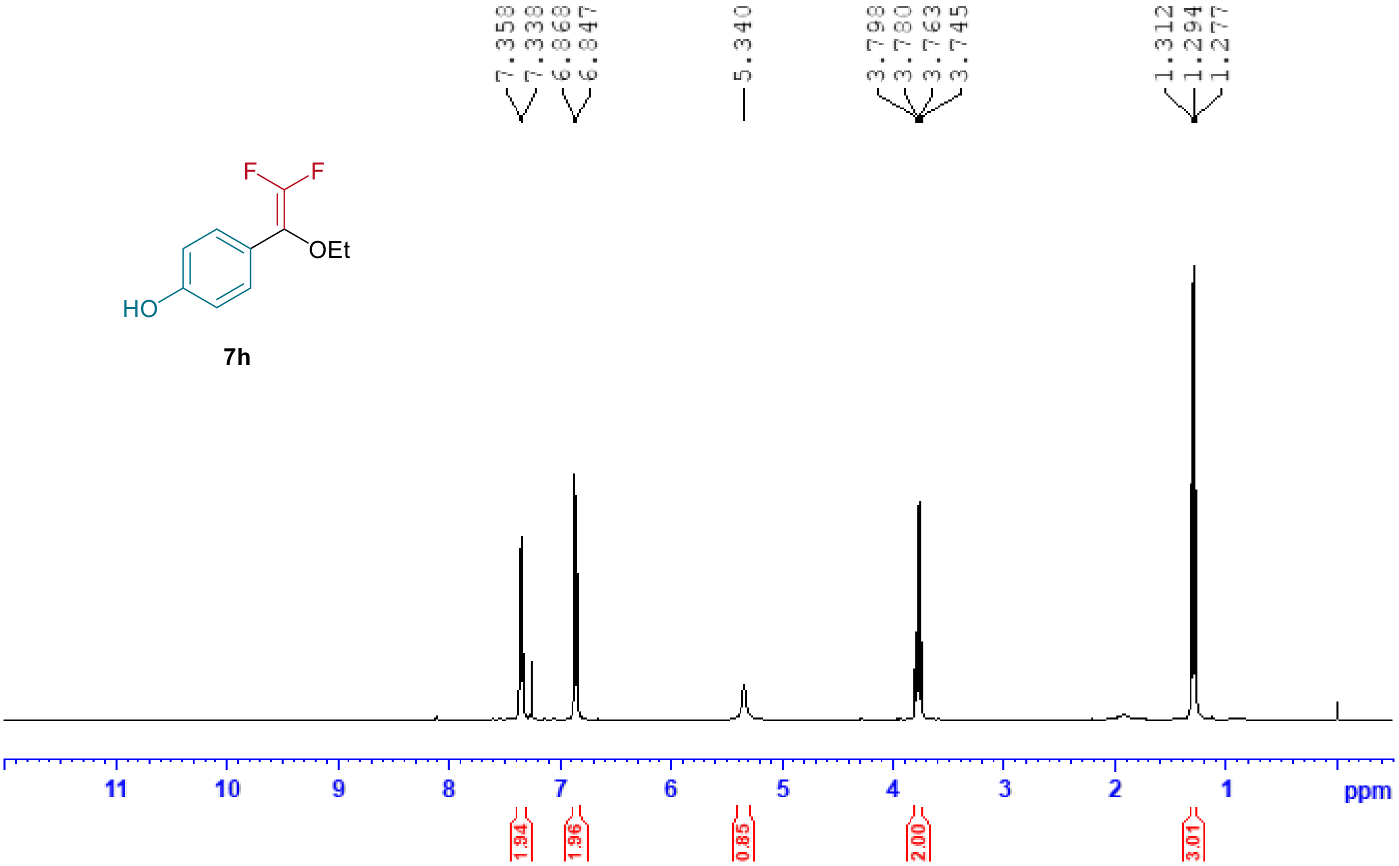

^{13}C NMR of **7h**

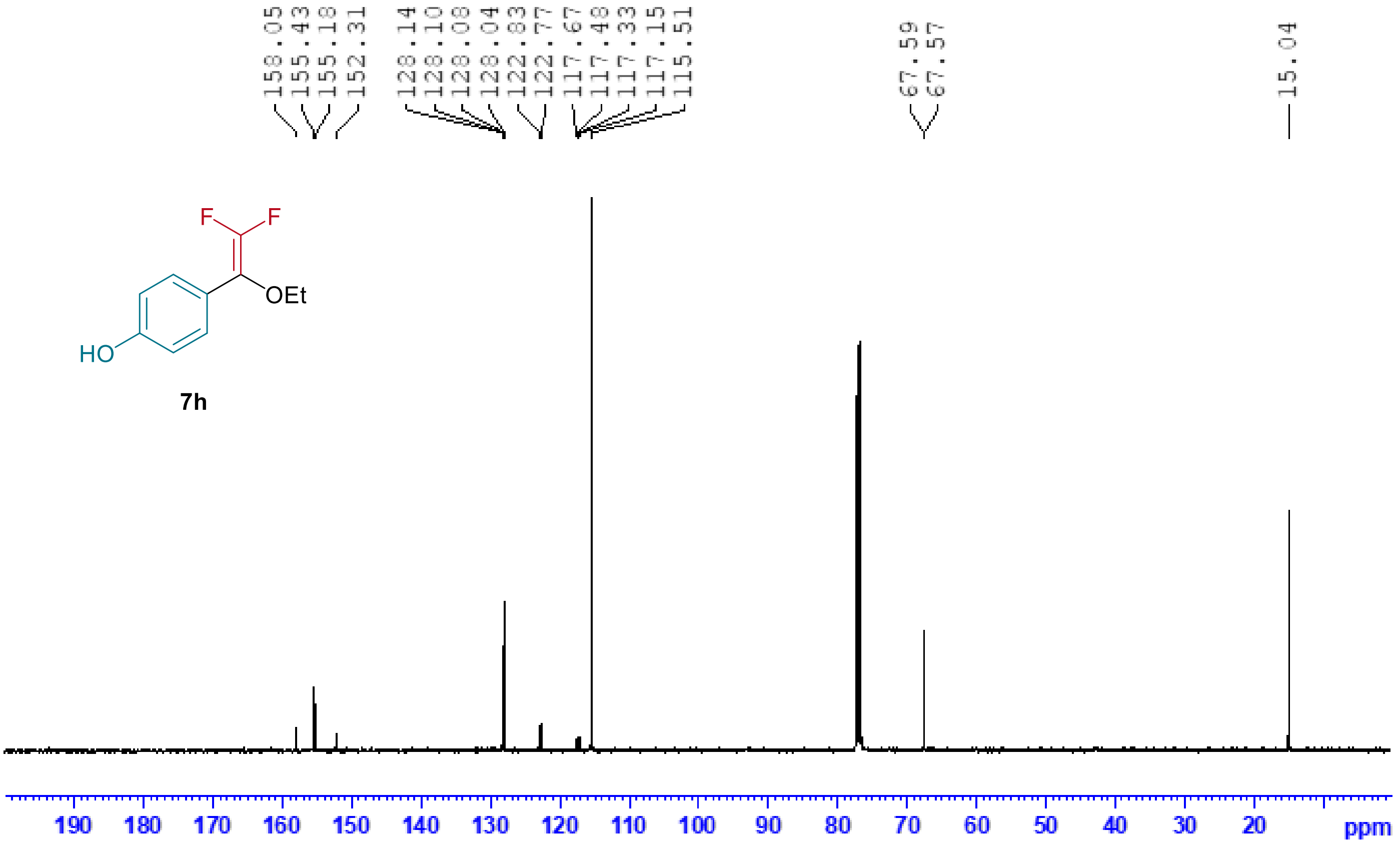

^{19}F NMR of **7h**

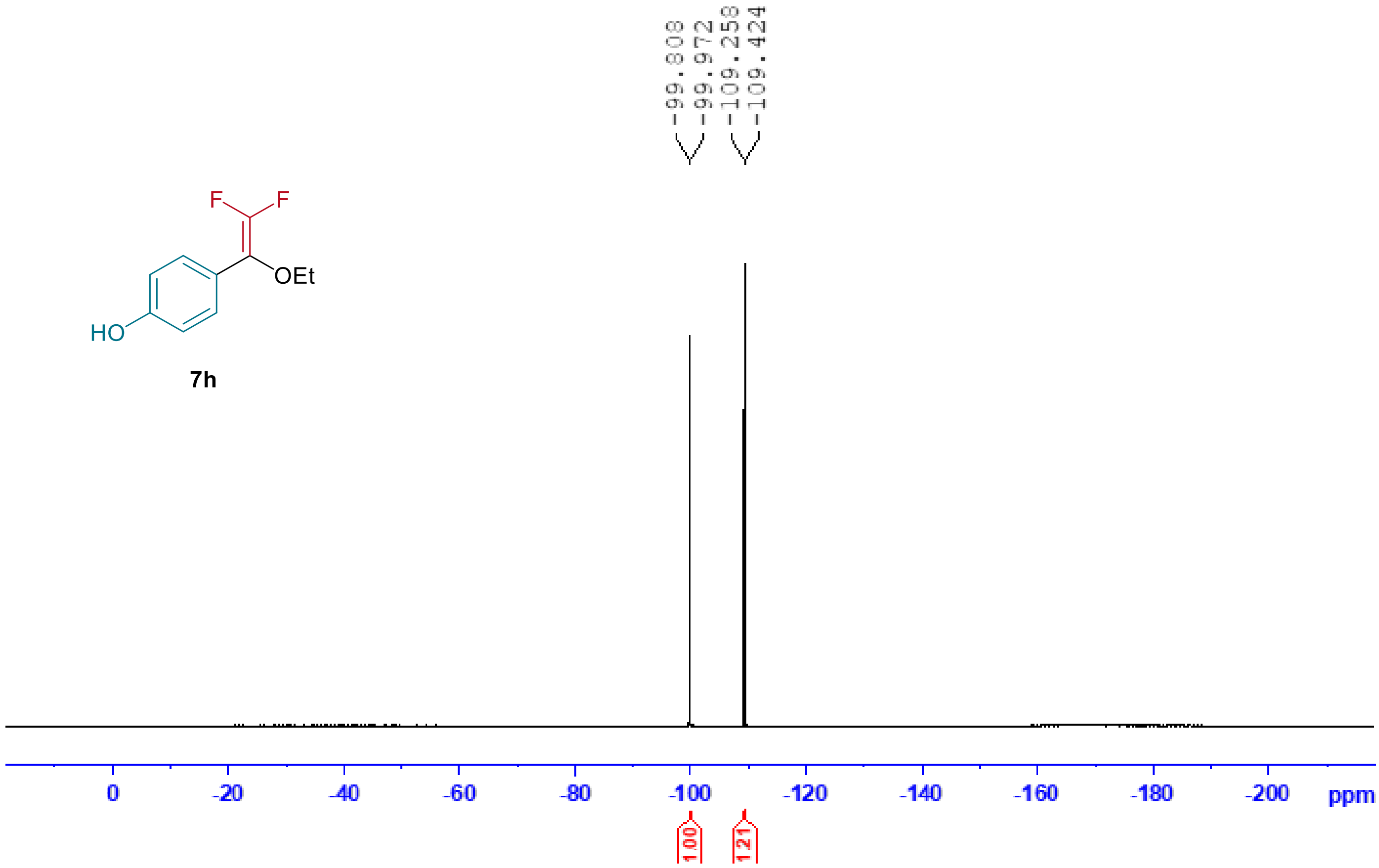

F
F
OEt
HO
7h
-99.808
-99.972
-109.258
-109.424
0
-20
-40
-60
-80
-100
-120
-140
-160
-180
-200
ppm
1.00
1.21

^{1}H NMR of **7i**

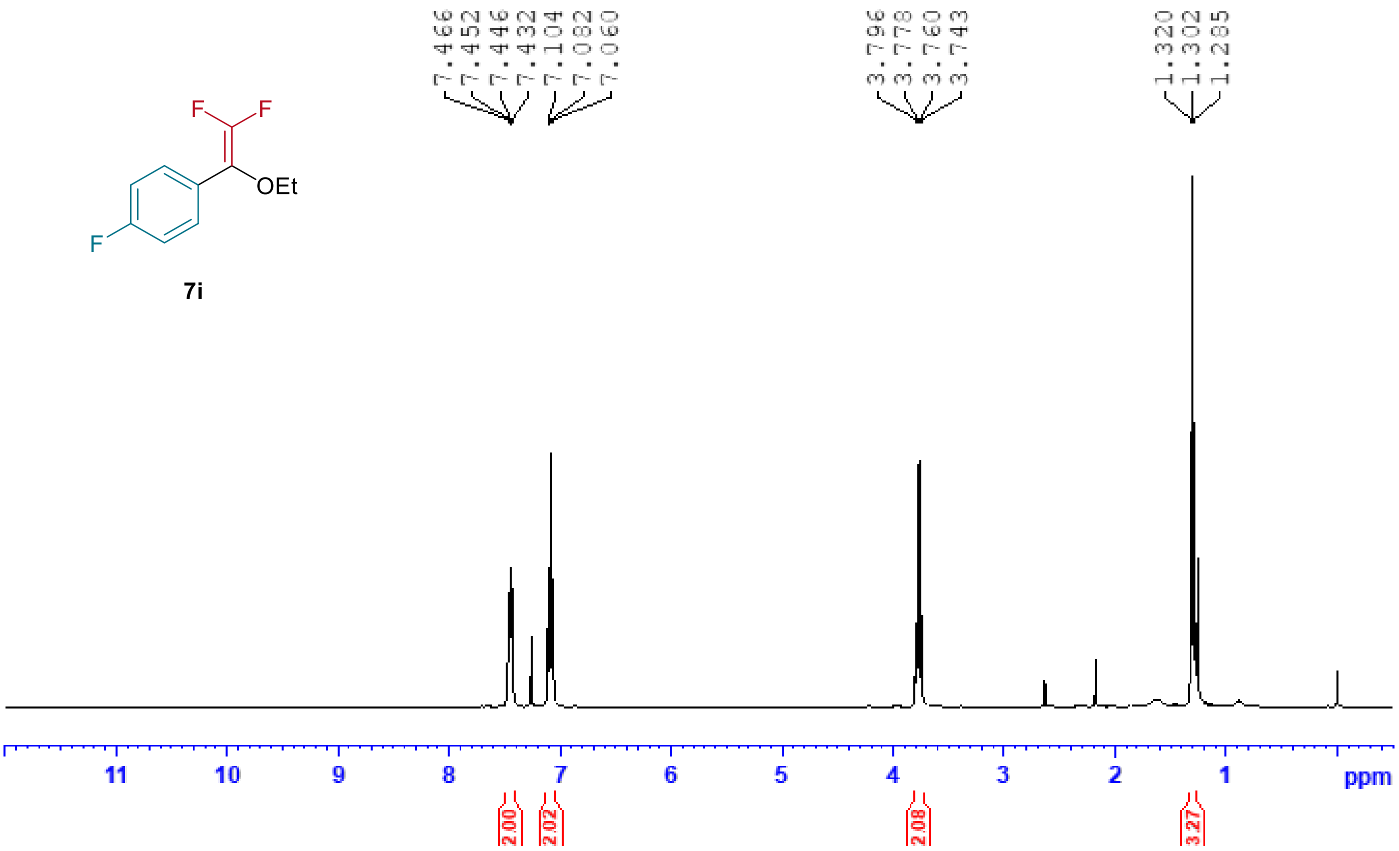

^{13}C NMR of **7i**

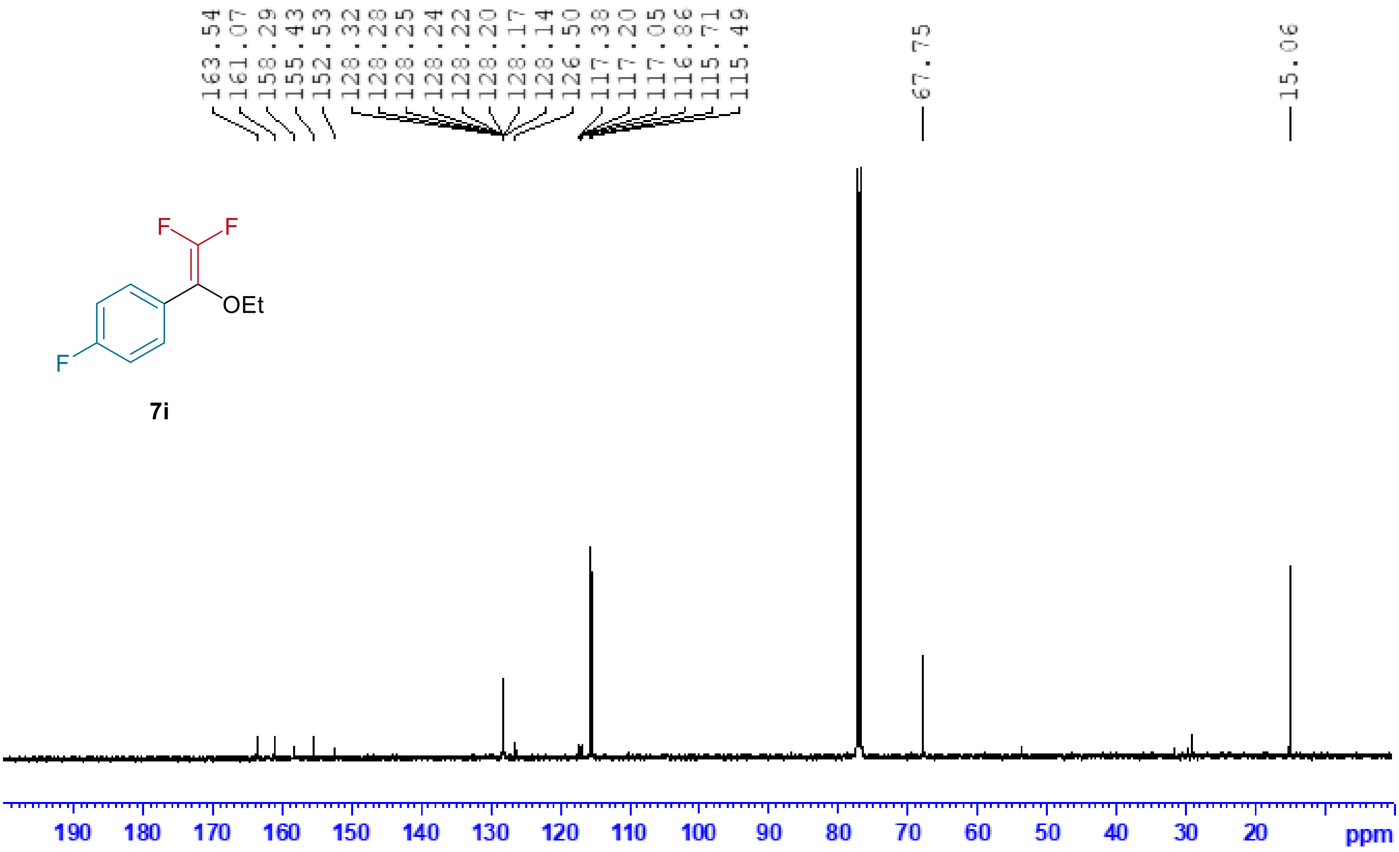

^{19}F NMR of **7i**

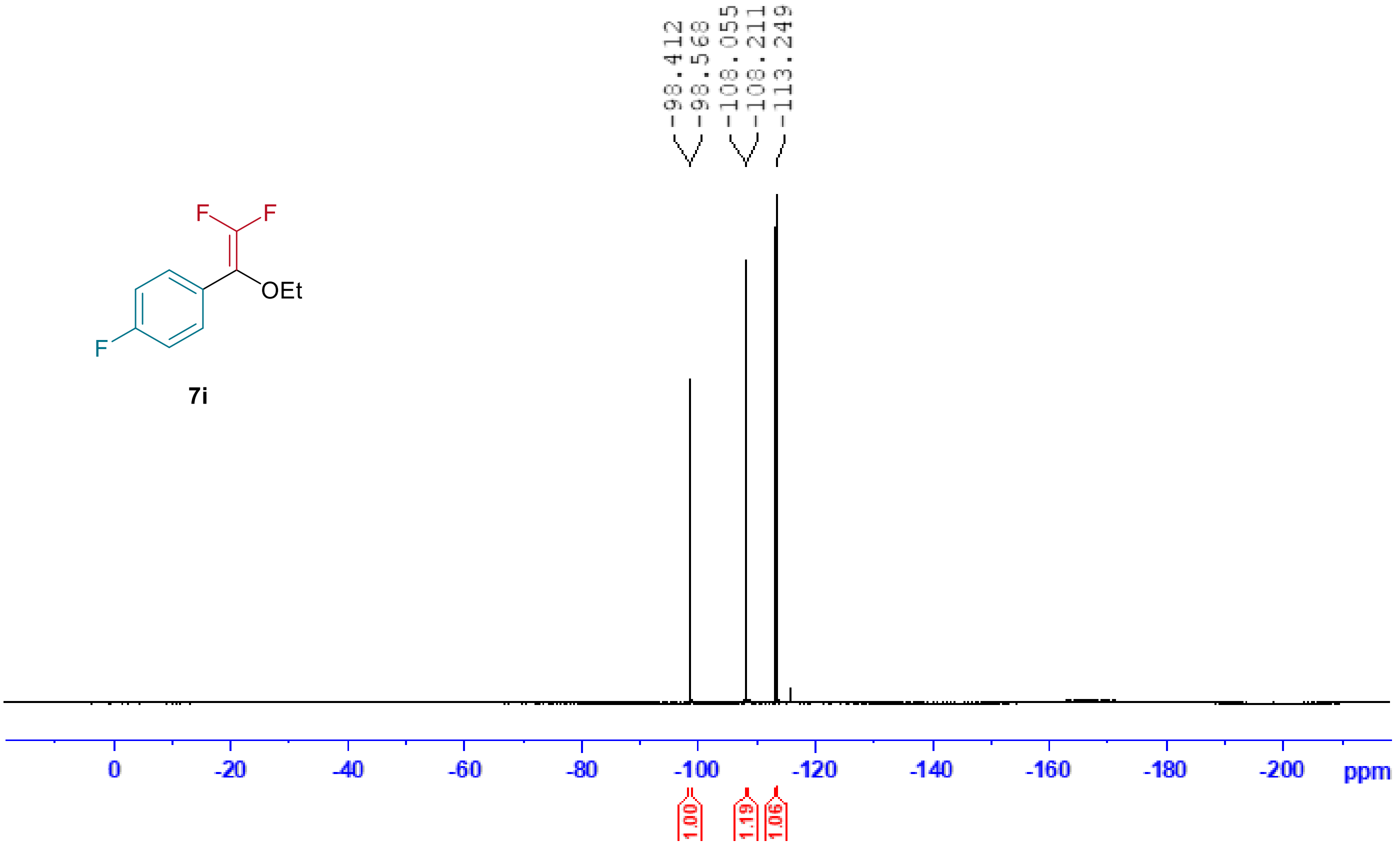

7i

^{1}H NMR of **7j**

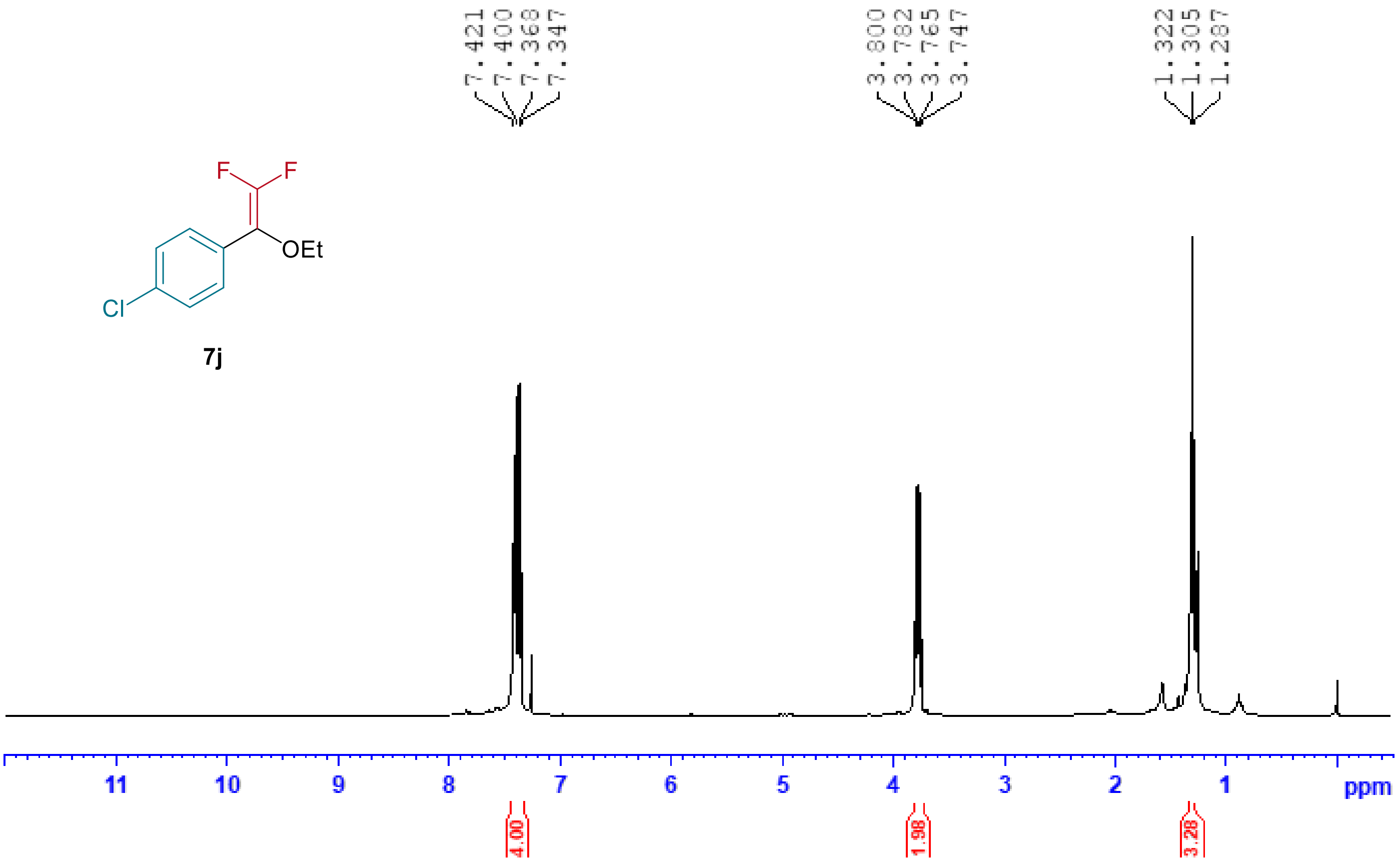

^{13}C NMR of **7j**

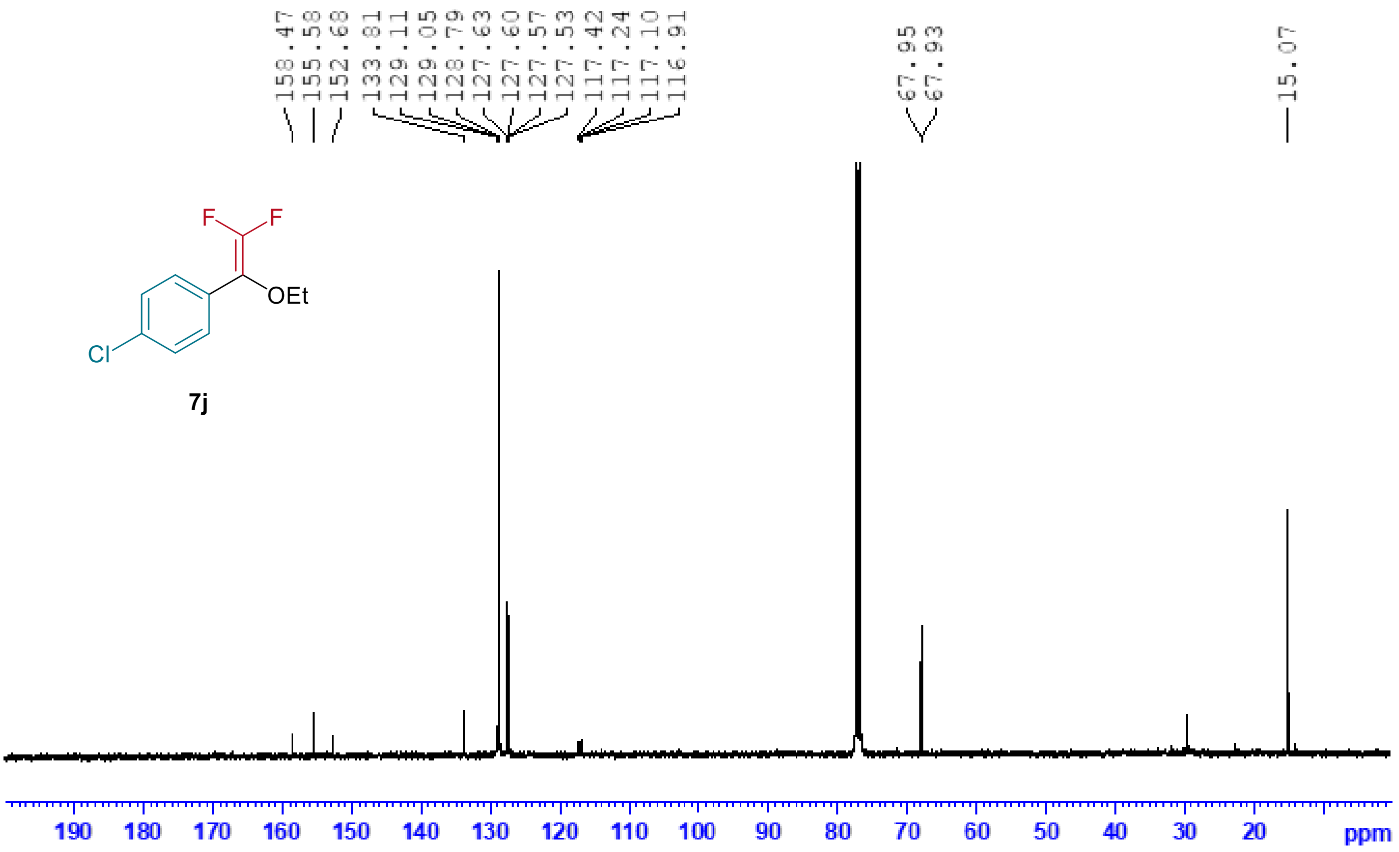

^{19}F NMR of **7j**

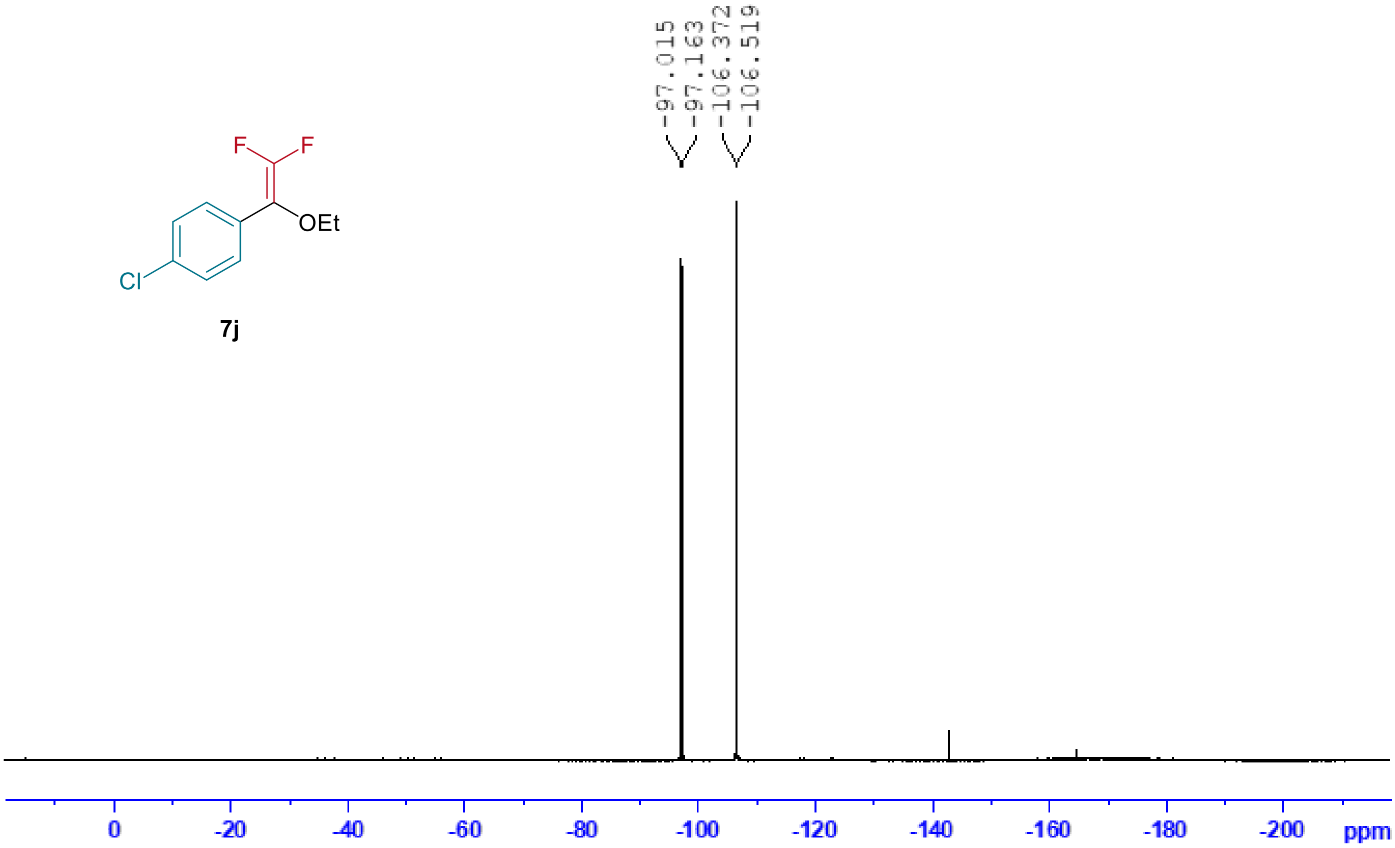
-97.015
-97.163
-106.372
-106.519
F
F
OEt
Cl
7j
0
-20
-40
-60
-80
-100
-120
-140
-160
-180
-200
ppm

^{1}H NMR of **7k**

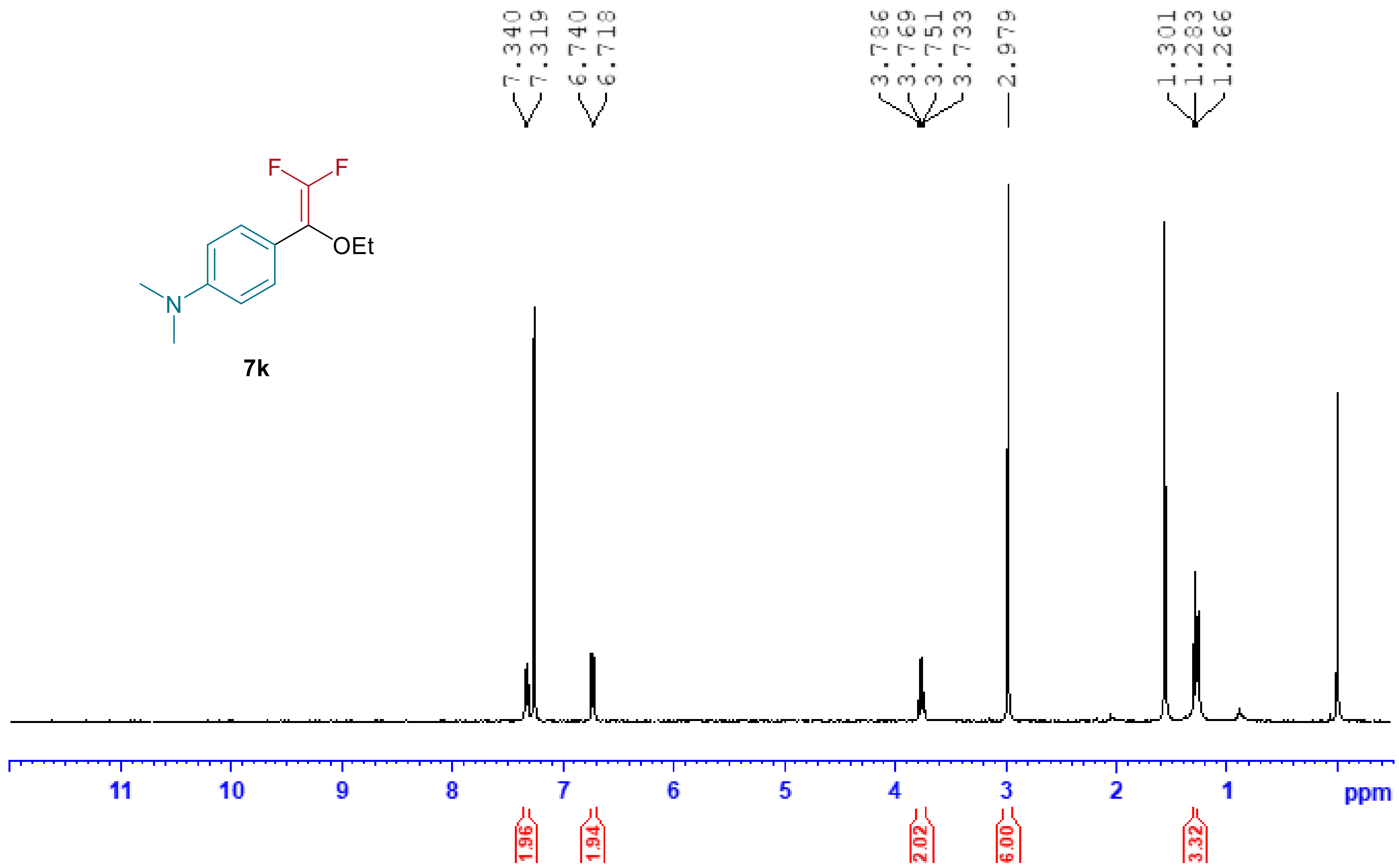

^{13}C NMR of **7k**

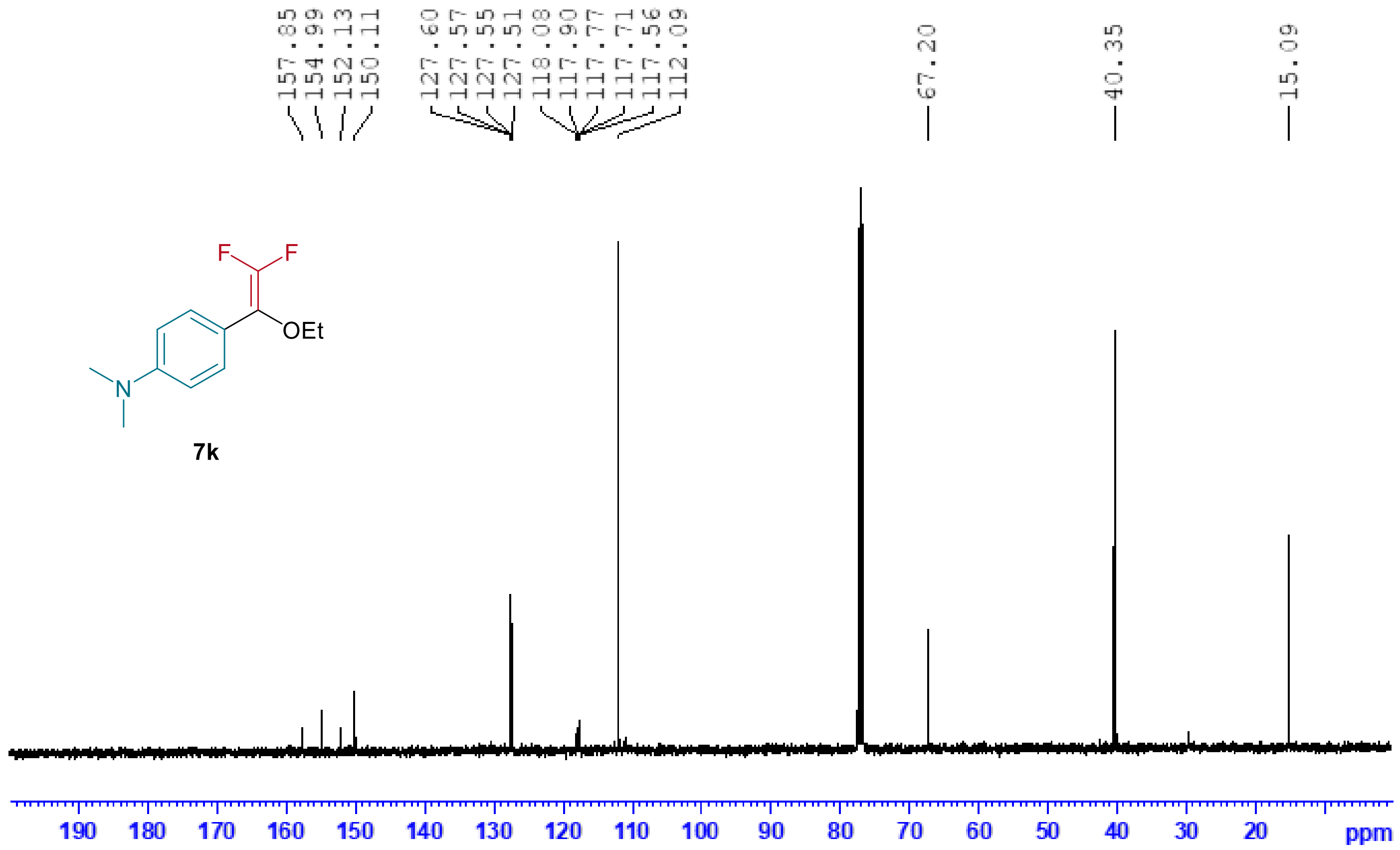

^{19}F NMR of **7k**

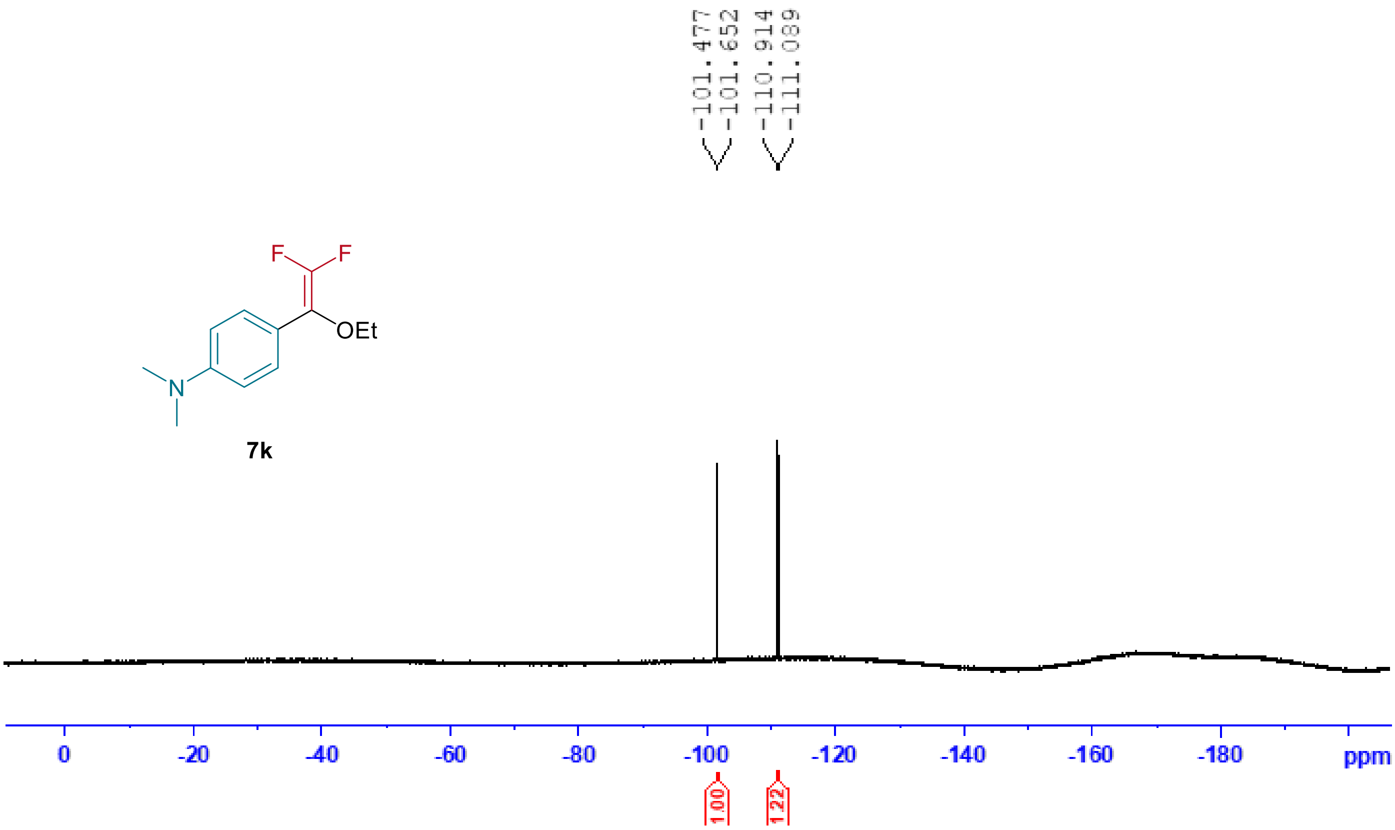

-101.477
-101.652
-110.914
-111.089
F
F
OEt
N
7k
0
-20
-40
-60
-80
-100
-120
-140
-160
-180
ppm
1.00
1.22

^{1}H NMR of **7l**

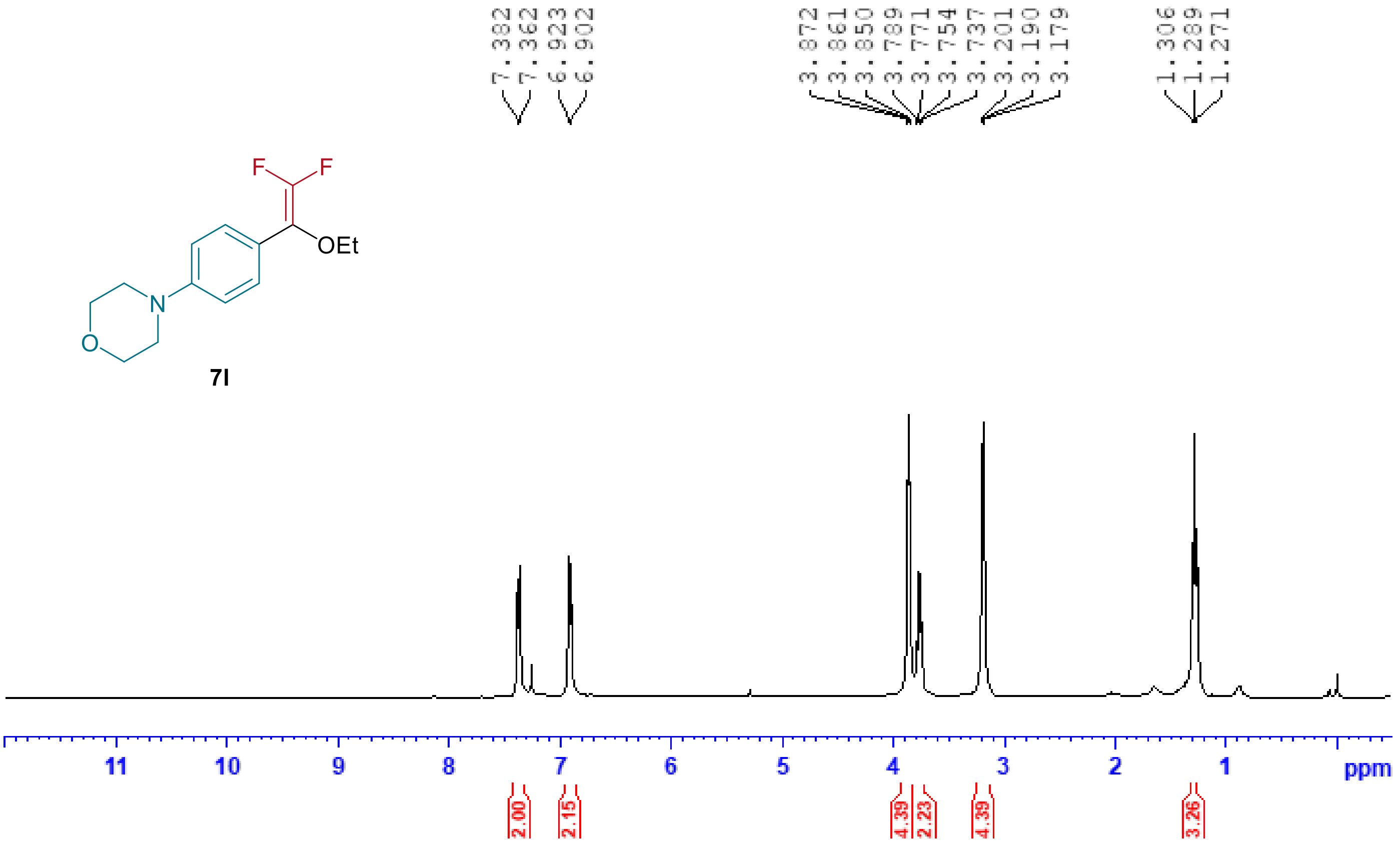

^{13}C NMR of **7l**

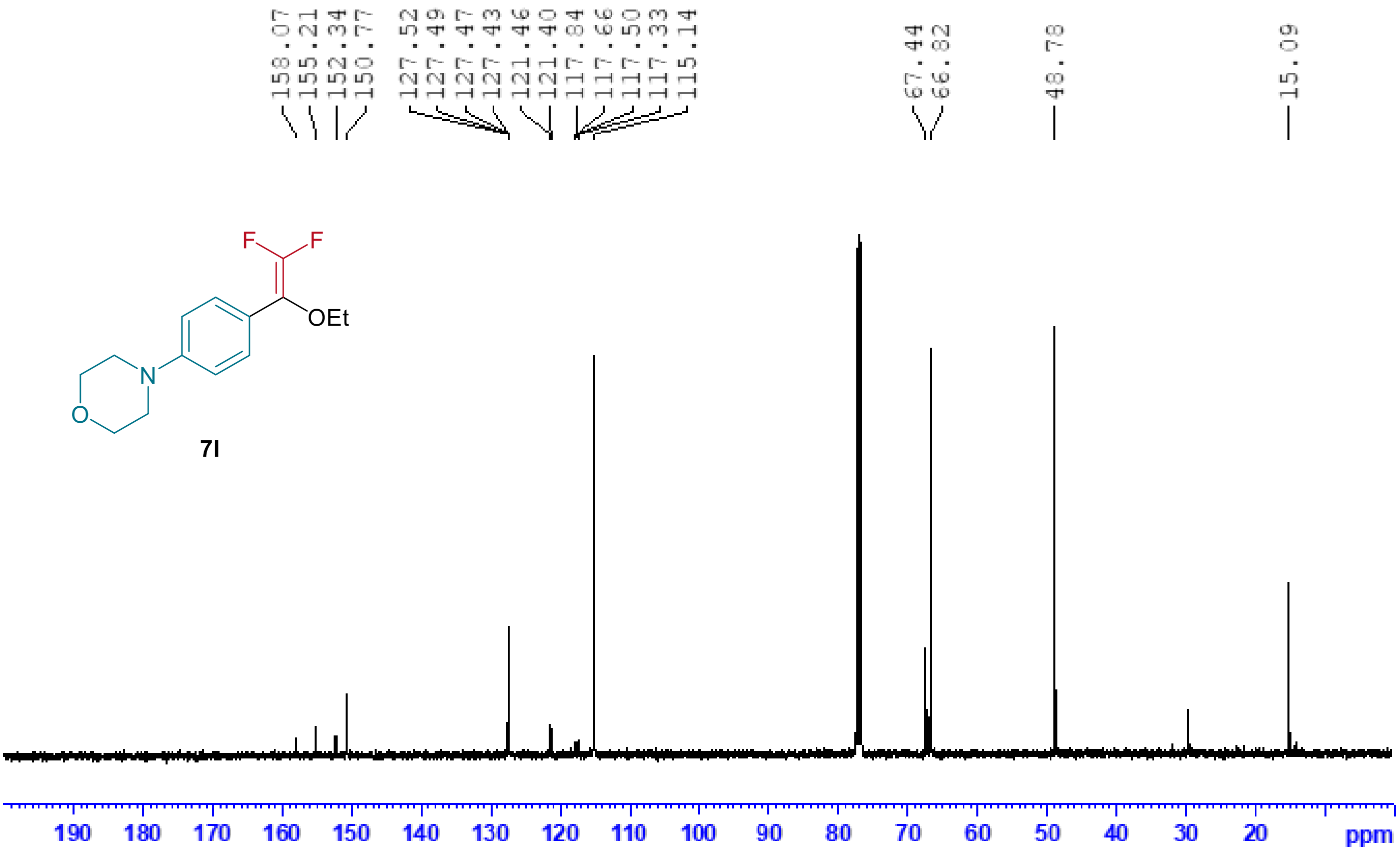

^{19}F NMR of **7l**

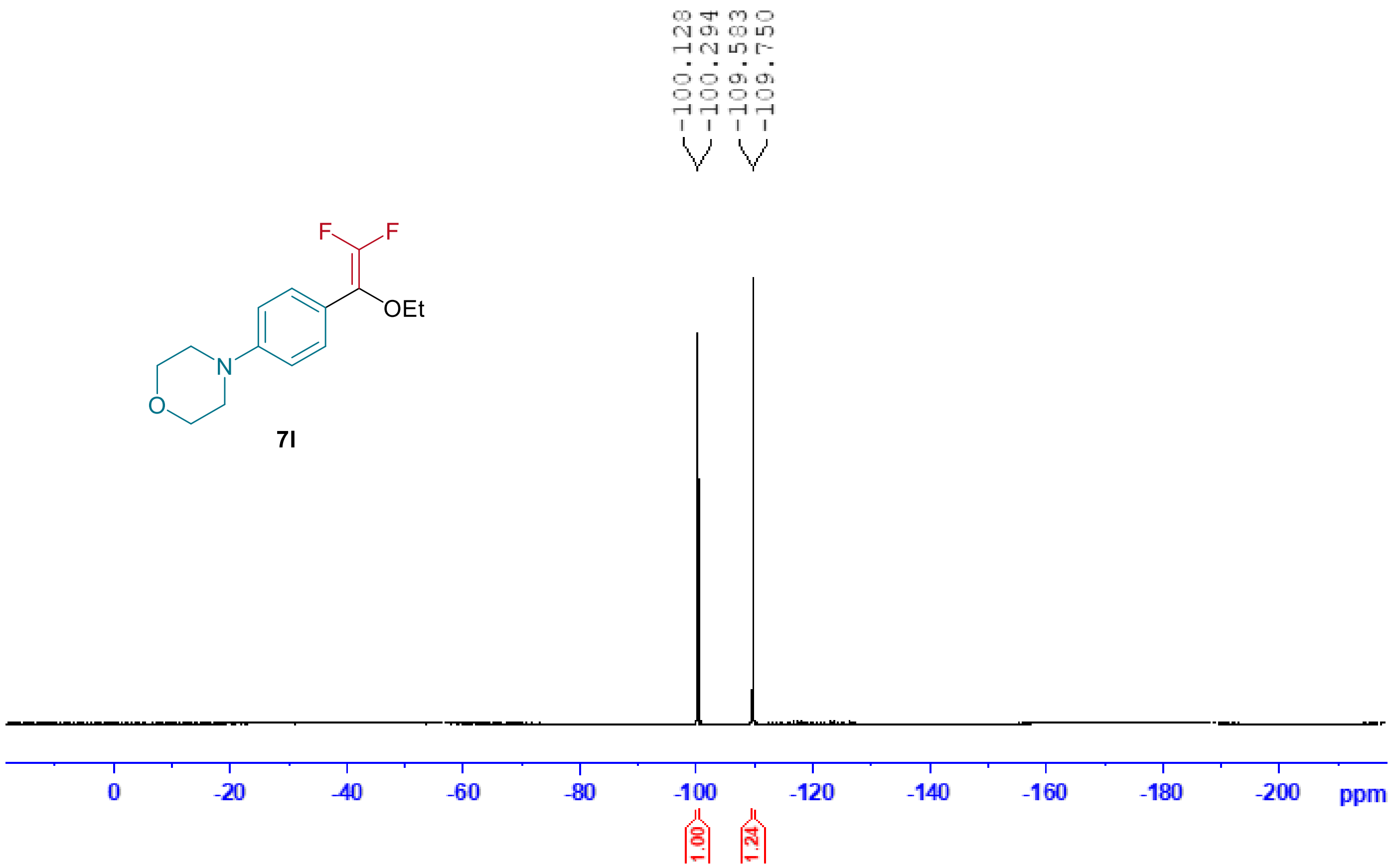

-100.128
-100.294
-109.583
-109.750
OEt
F
F
N
O
7l
0
-20
-40
-60
-80
-100
-120
-140
-160
-180
-200
ppm
1.00
1.24

^{1}H NMR of **7m**

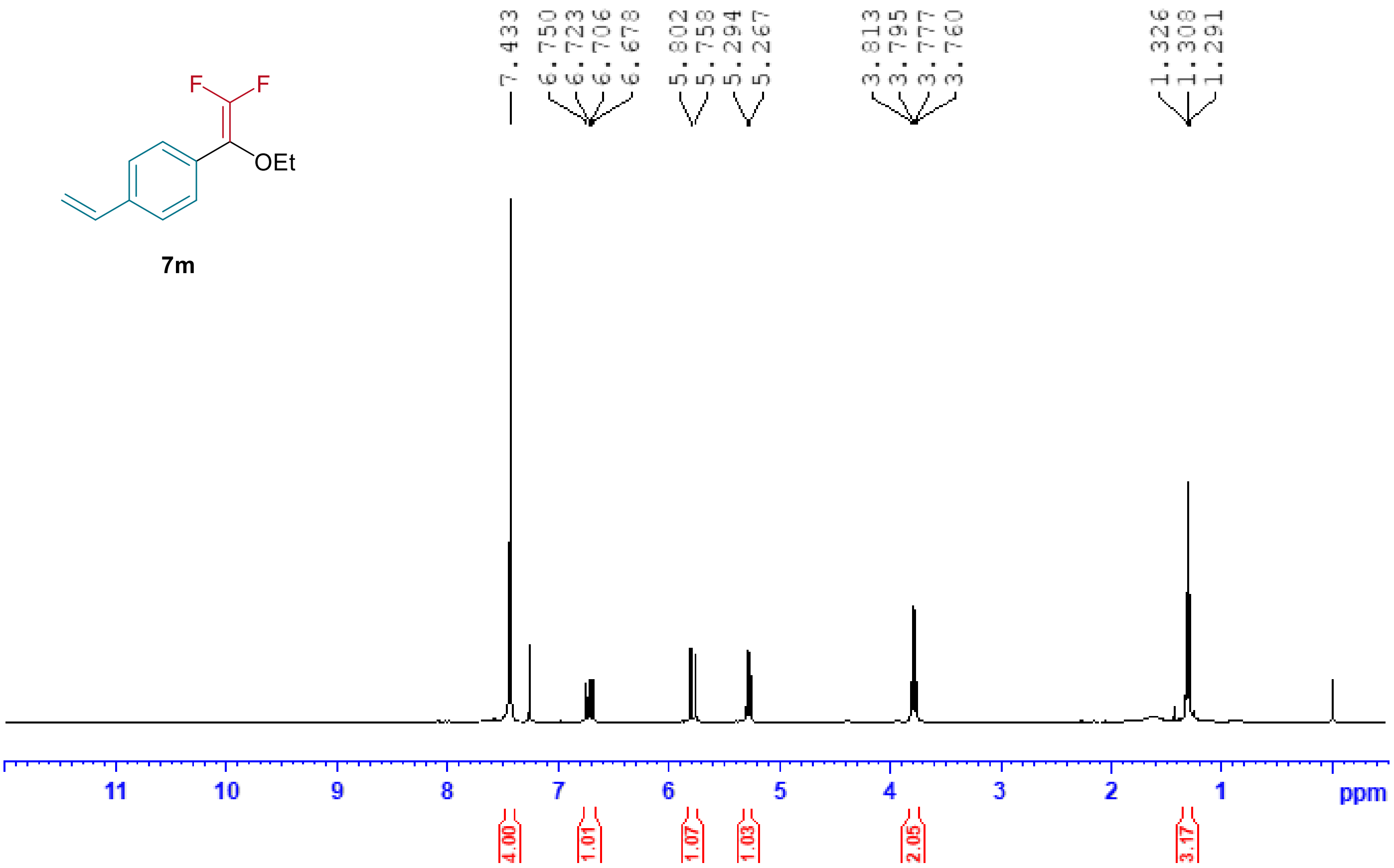

^{13}C NMR of **7m**

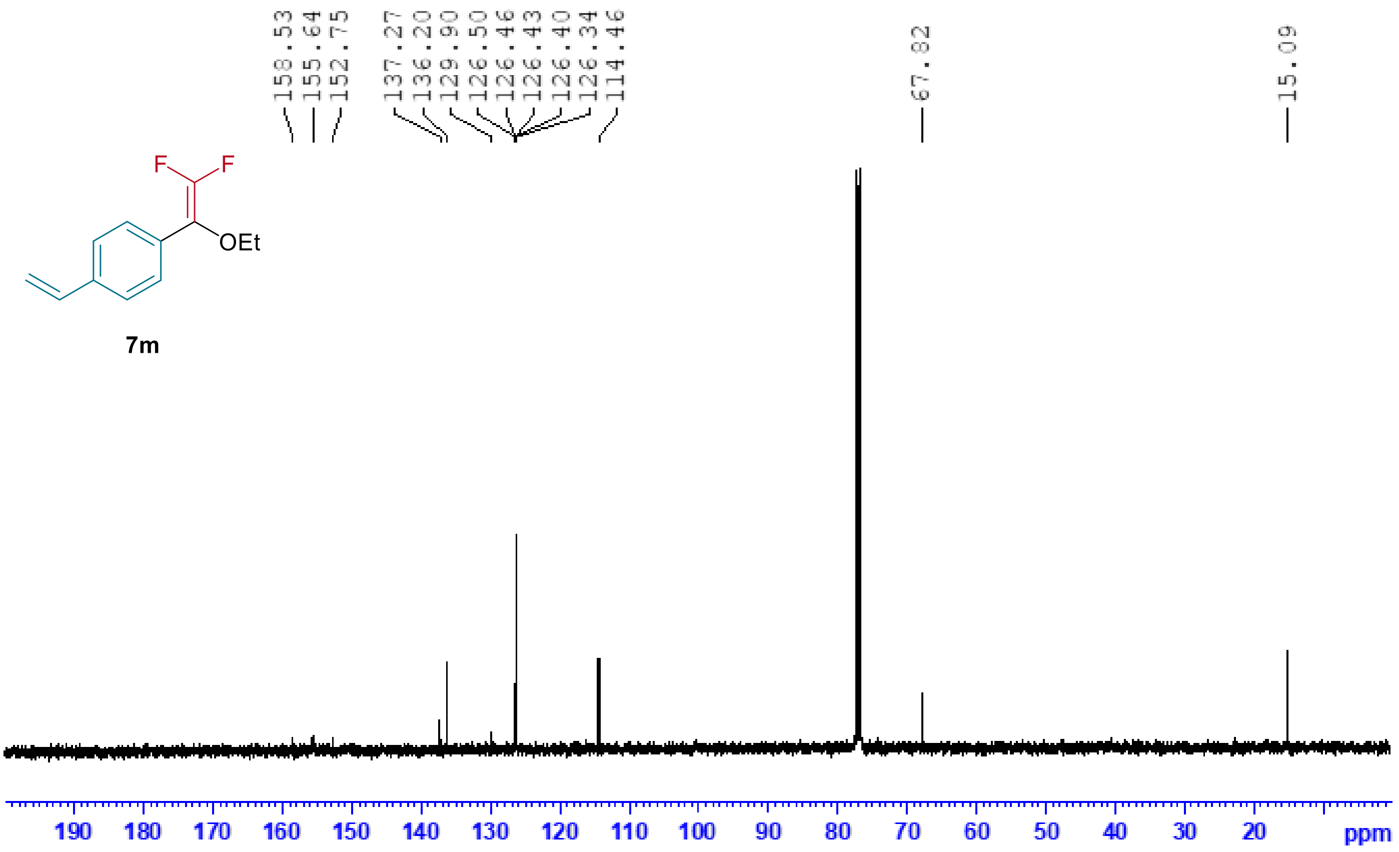

^{19}F NMR of **7m**

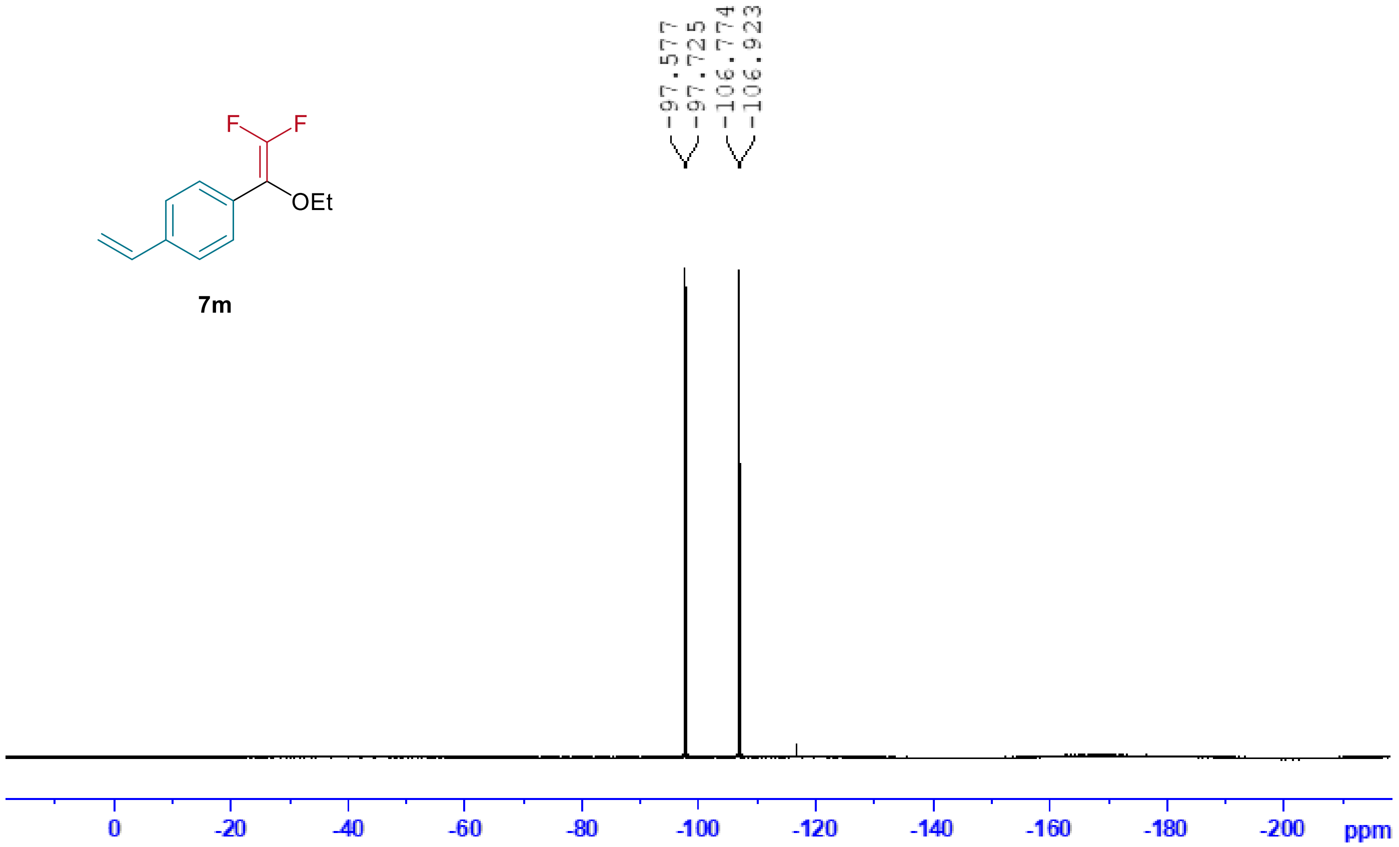

F
F
OEt
7m
-97.577
-97.725
-106.774
-106.923
0
-20
-40
-60
-80
-100
-120
-140
-160
-180
-200
ppm

^{1}H NMR of **7n**

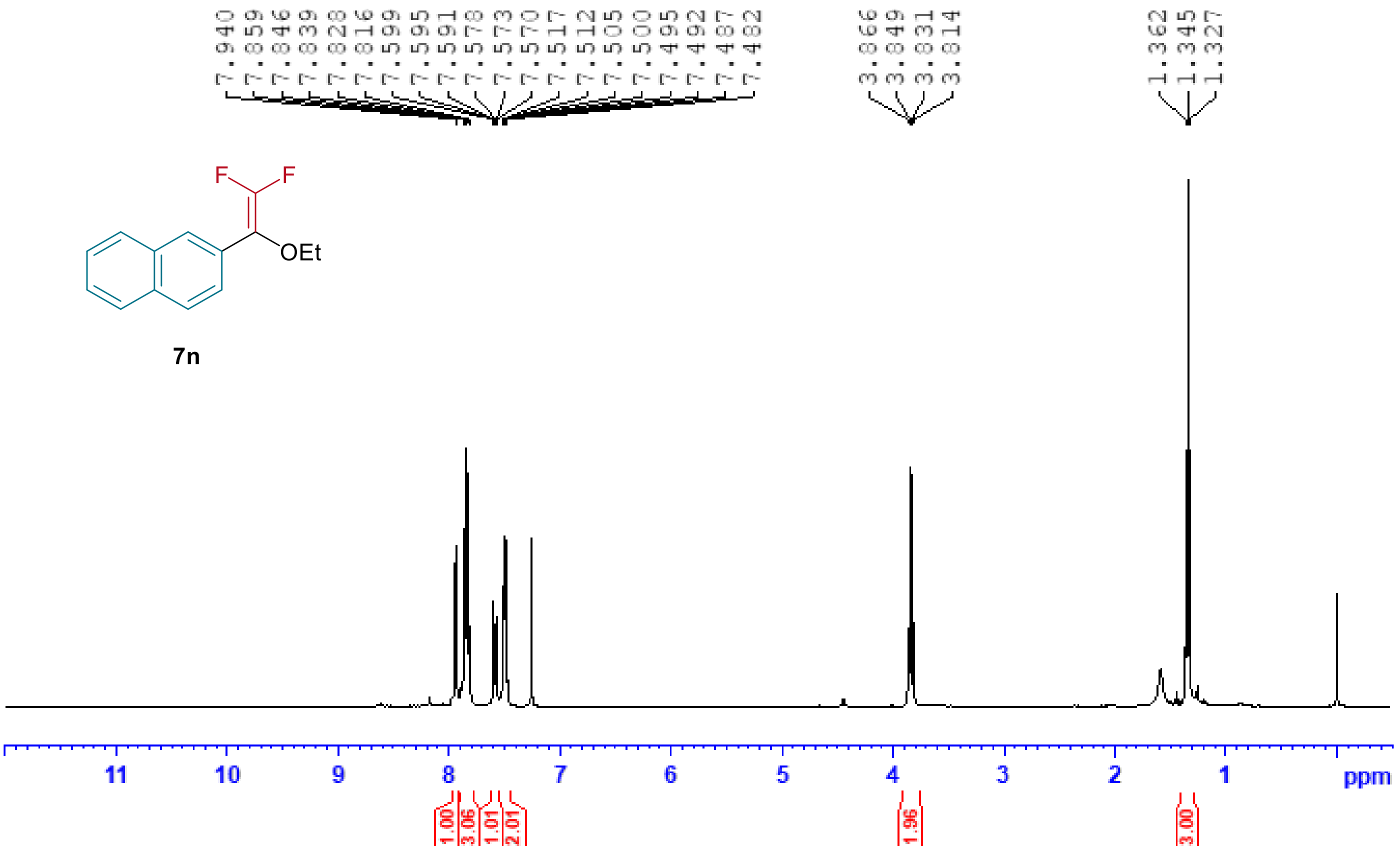

^{13}C NMR of **7n**

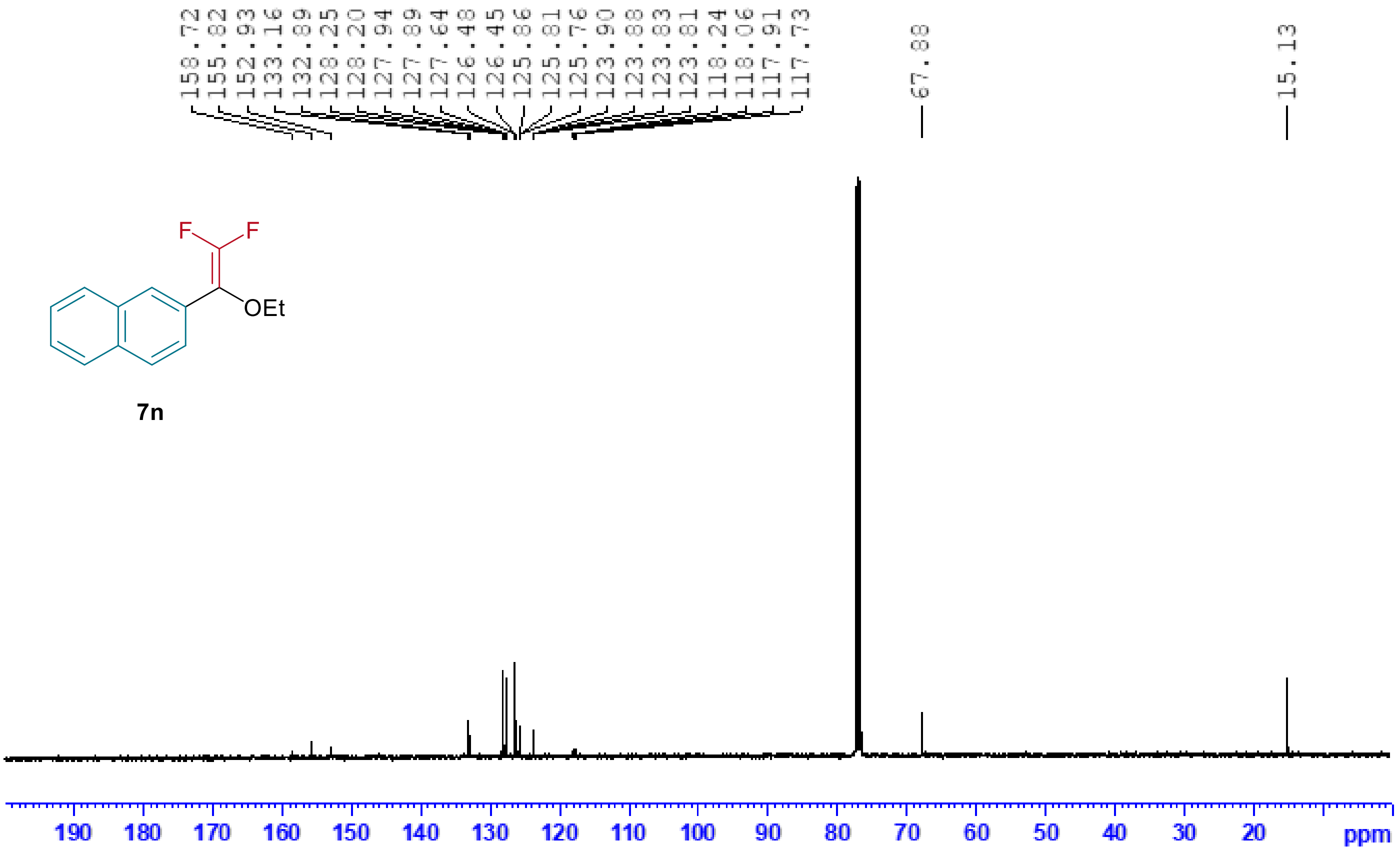

^{19}F NMR of **7n**

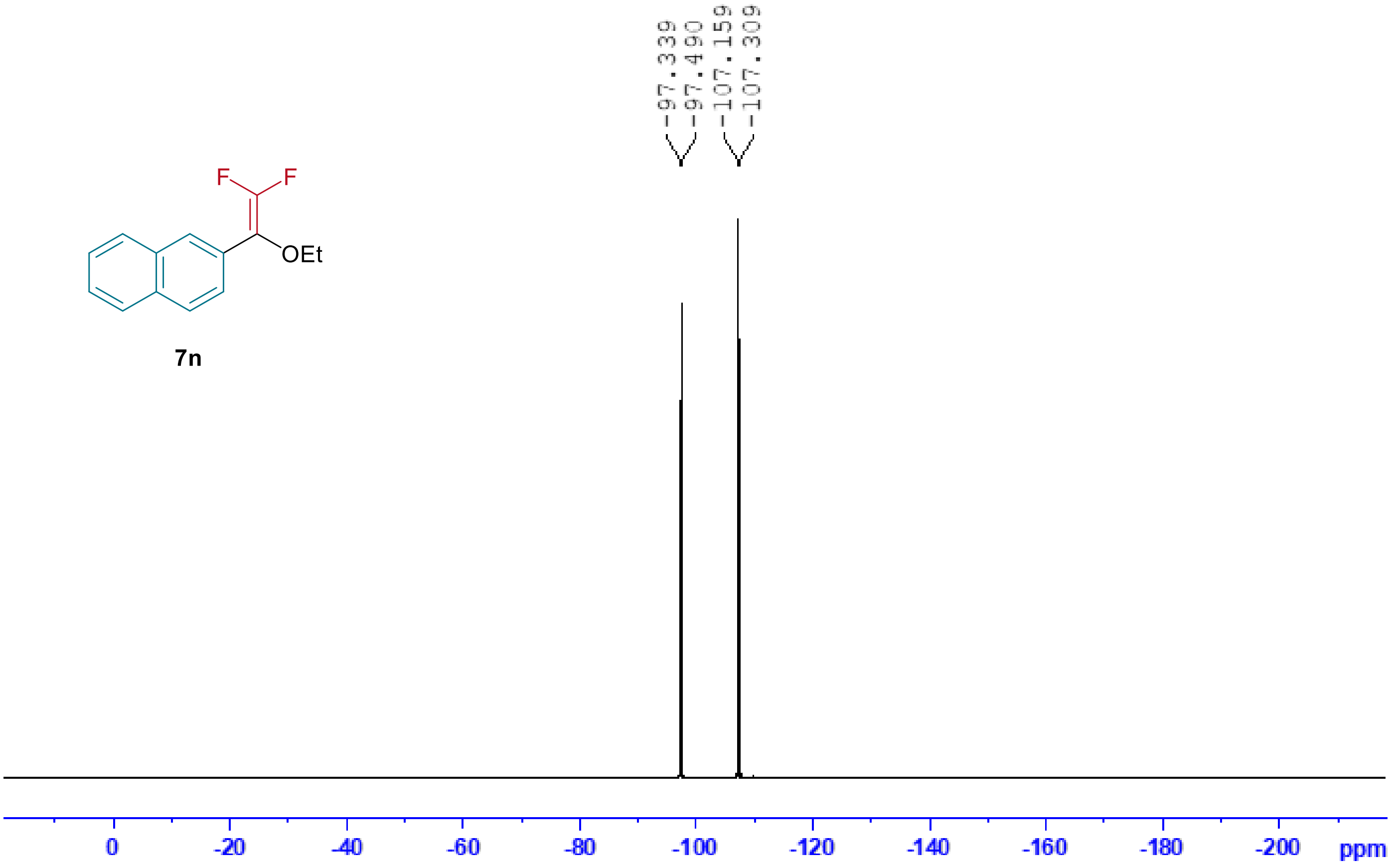

-97.339
-97.490
-107.159
-107.309
F
F
OEt
7n
0
-20
-40
-60
-80
-100
-120
-140
-160
-180
-200
ppm

^{1}H NMR of **7o**

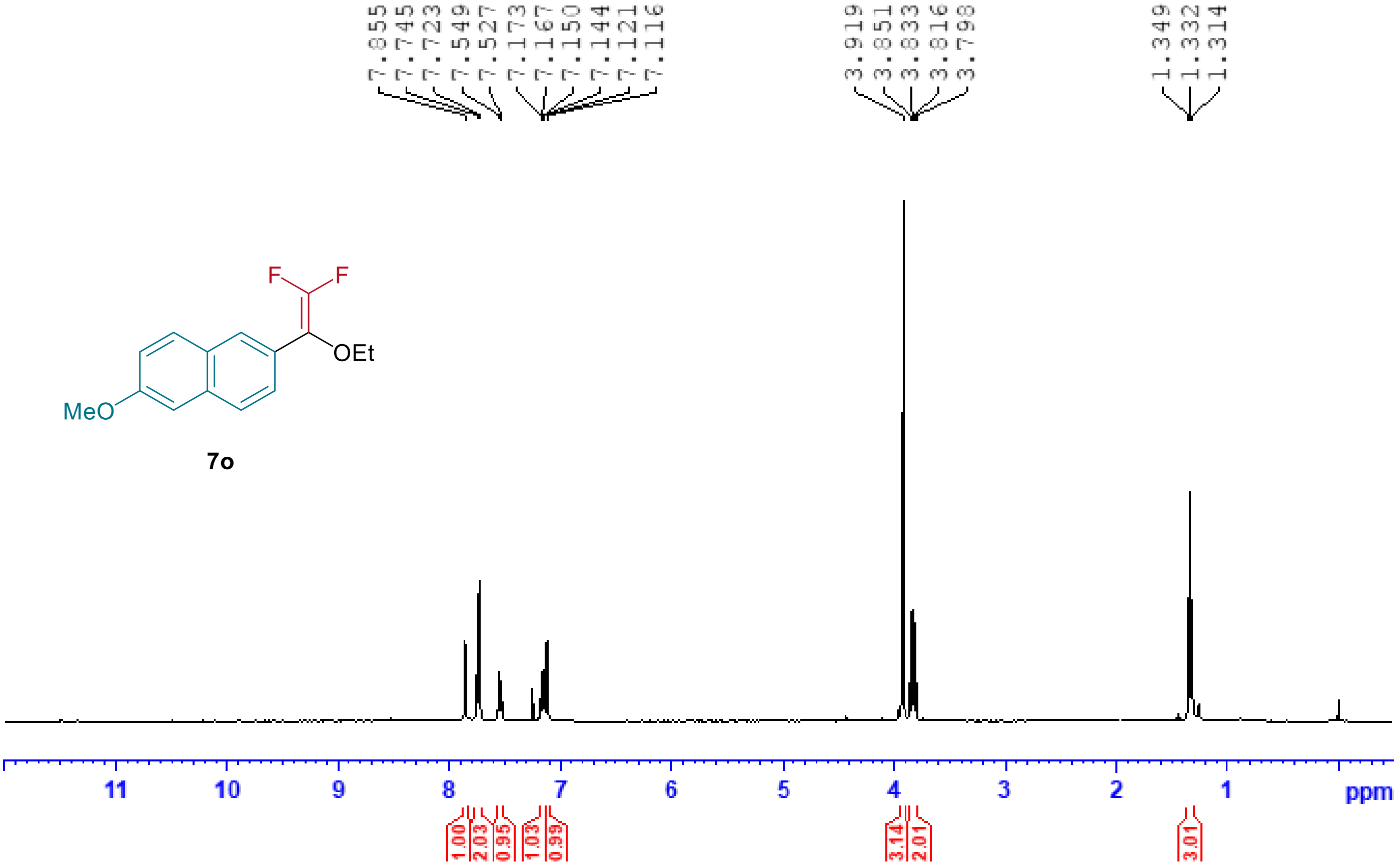

^{13}C NMR of **7o**

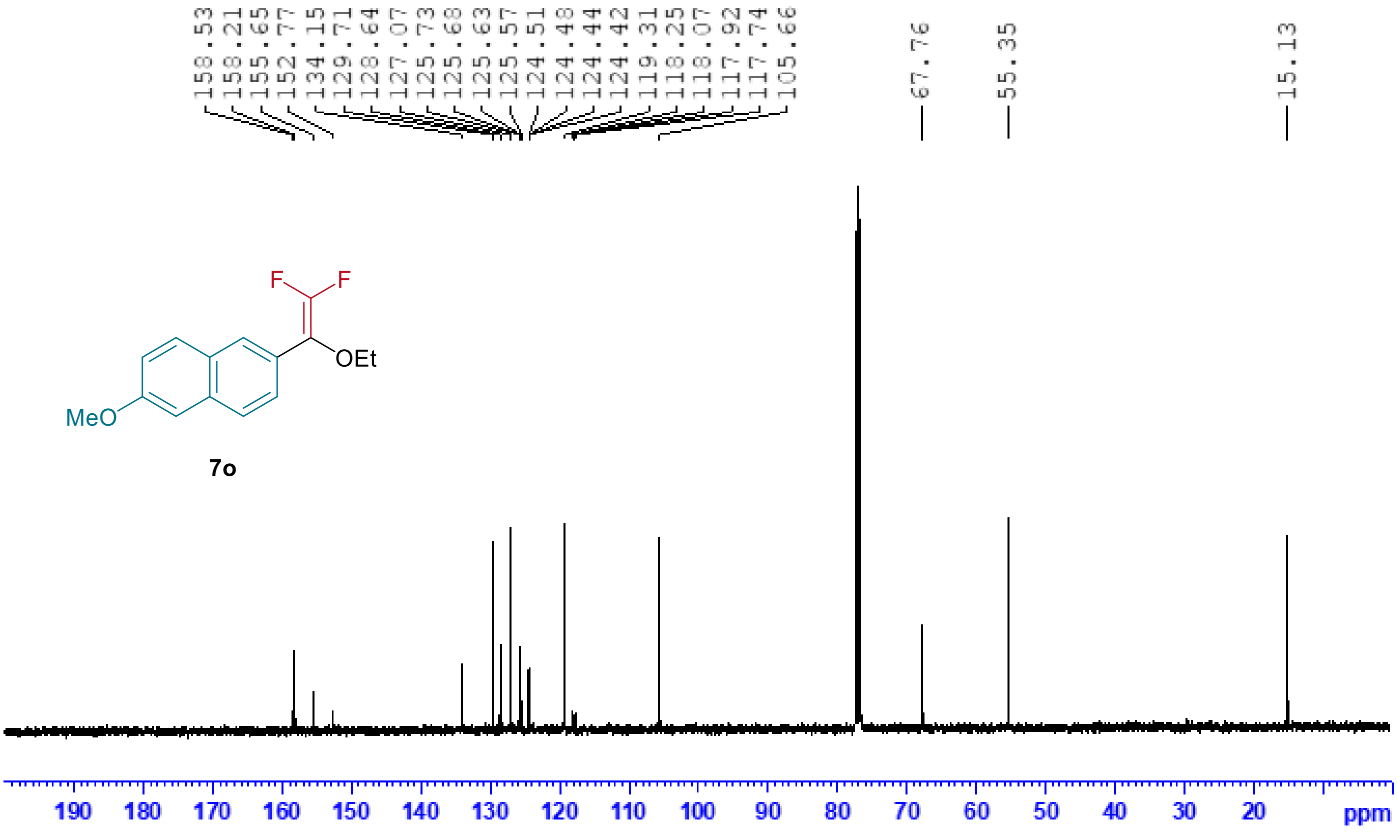

^{19}F NMR of **7o**

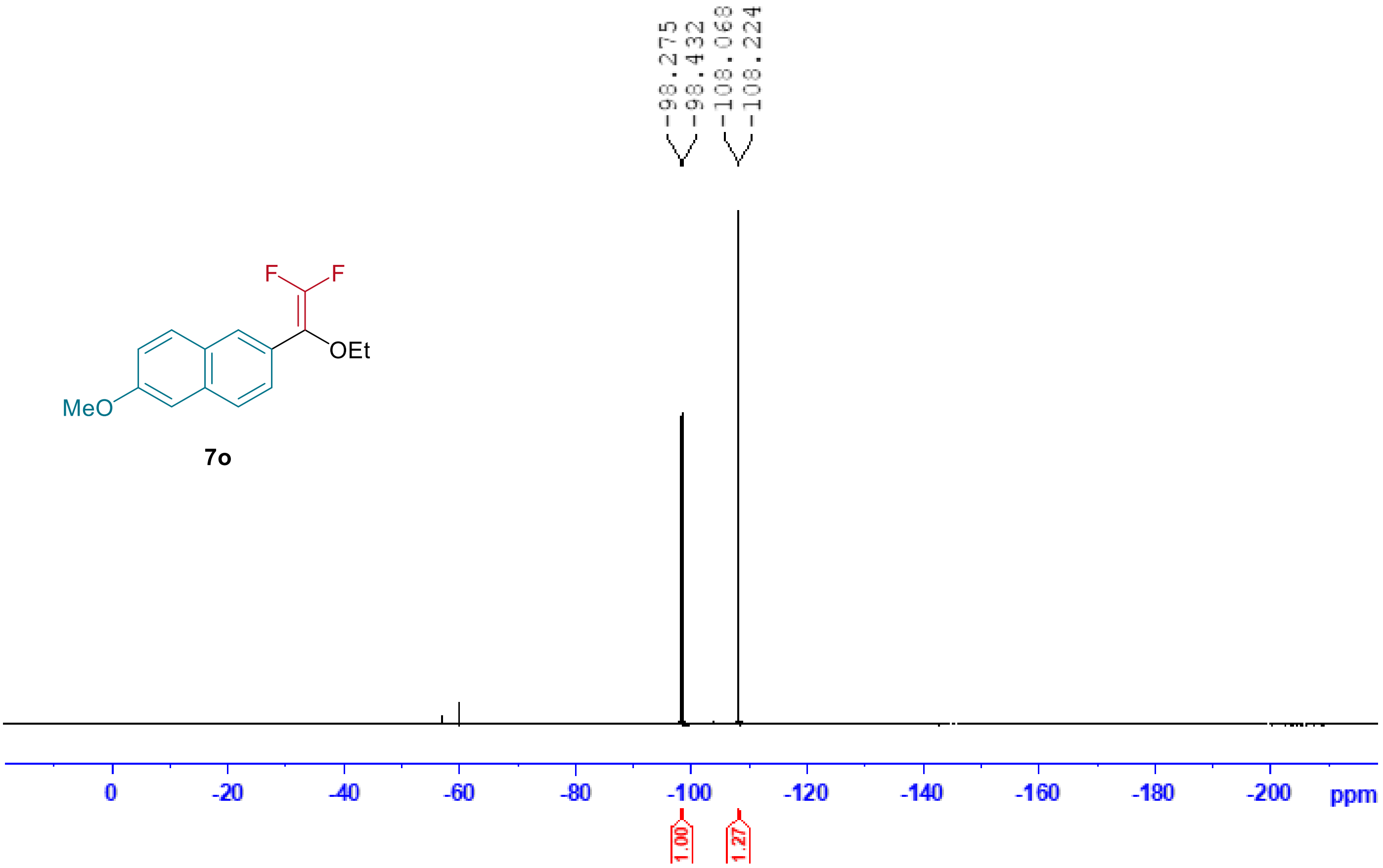

-98.275
-98.432
-108.068
-108.224
F
F
OEt
MeO
7o
0
-20
-40
-60
-80
-100
-120
-140
-160
-180
-200
ppm
1.00
1.27

^{1}H NMR of **7p**

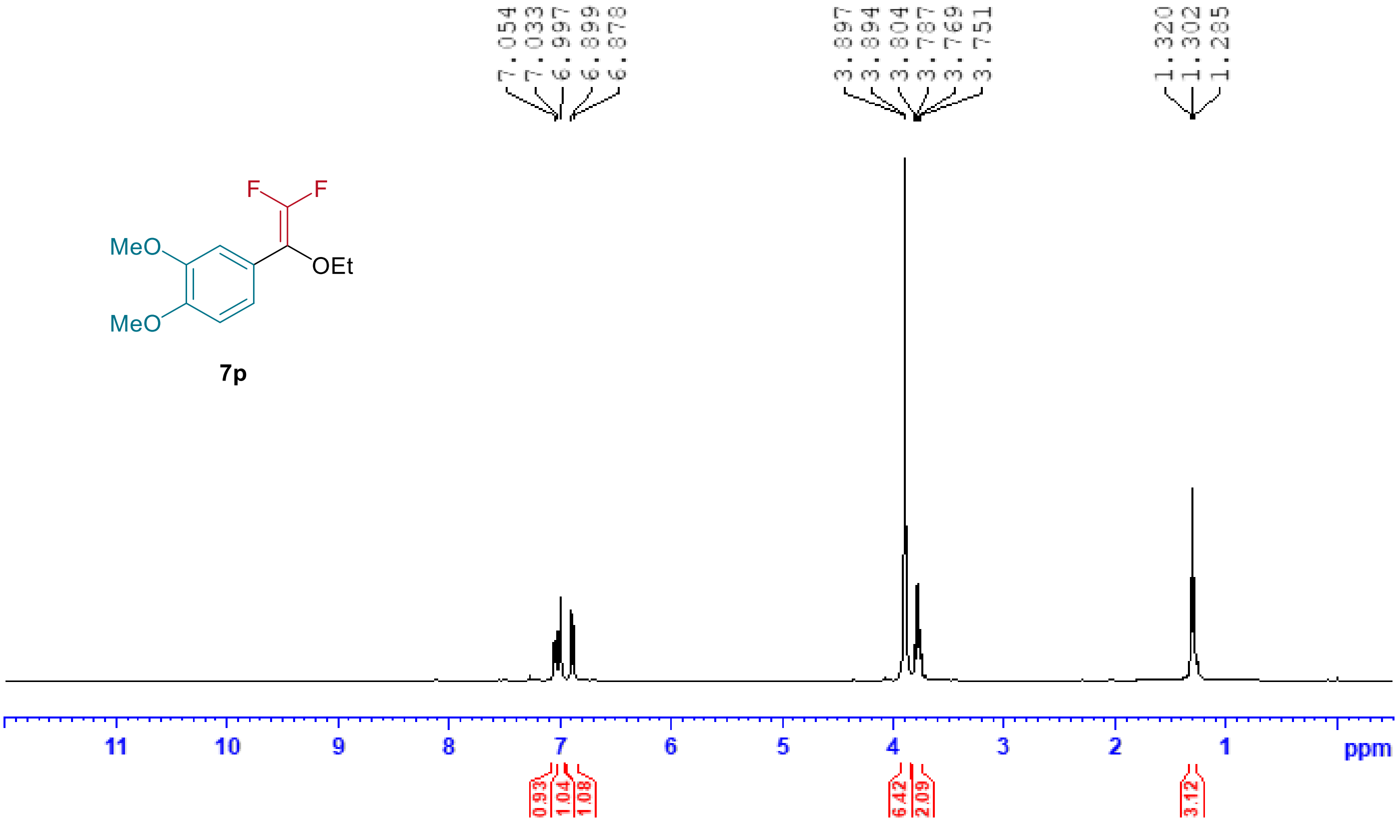

^{13}C NMR of **7p**

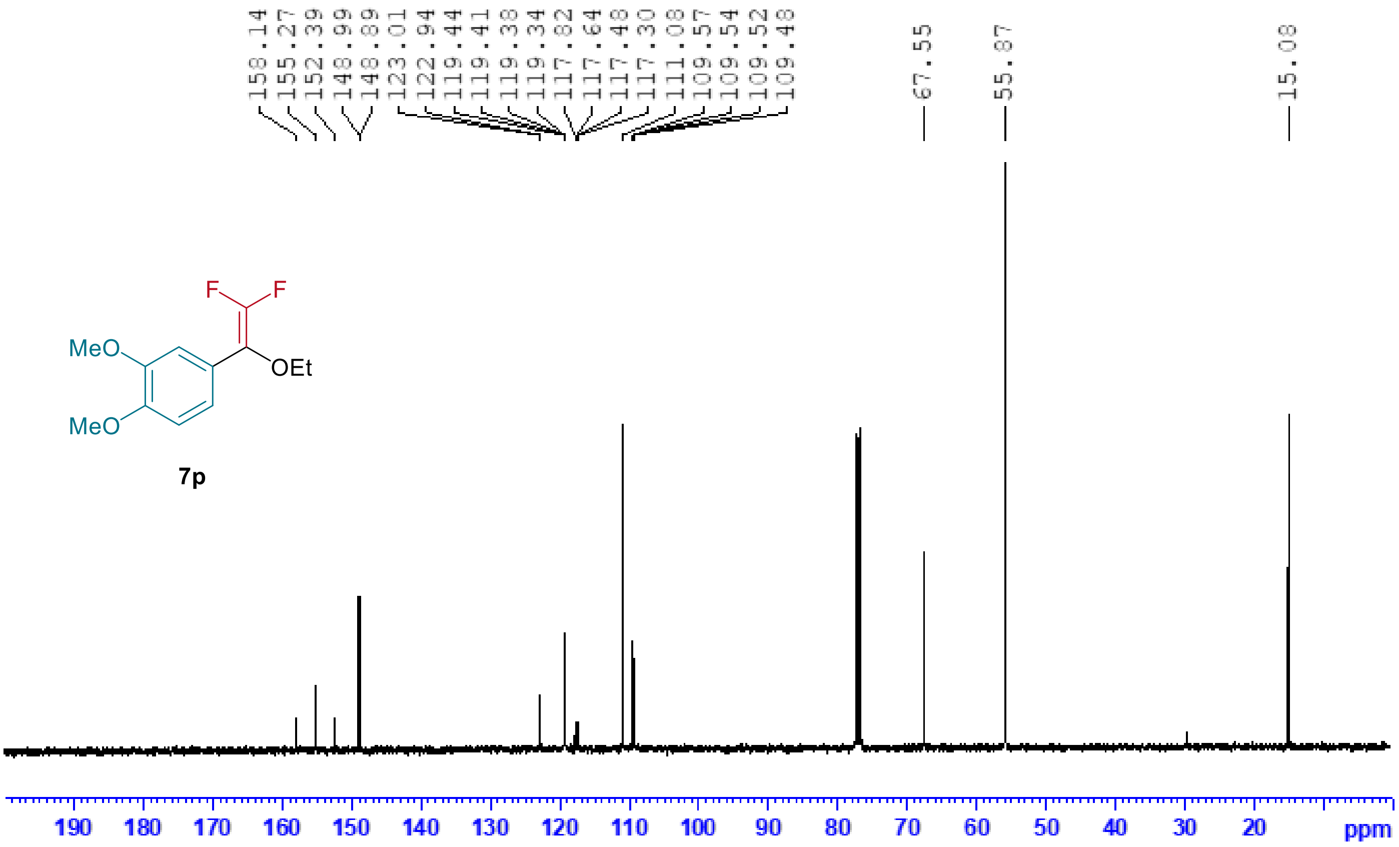

^{19}F NMR of **7p**

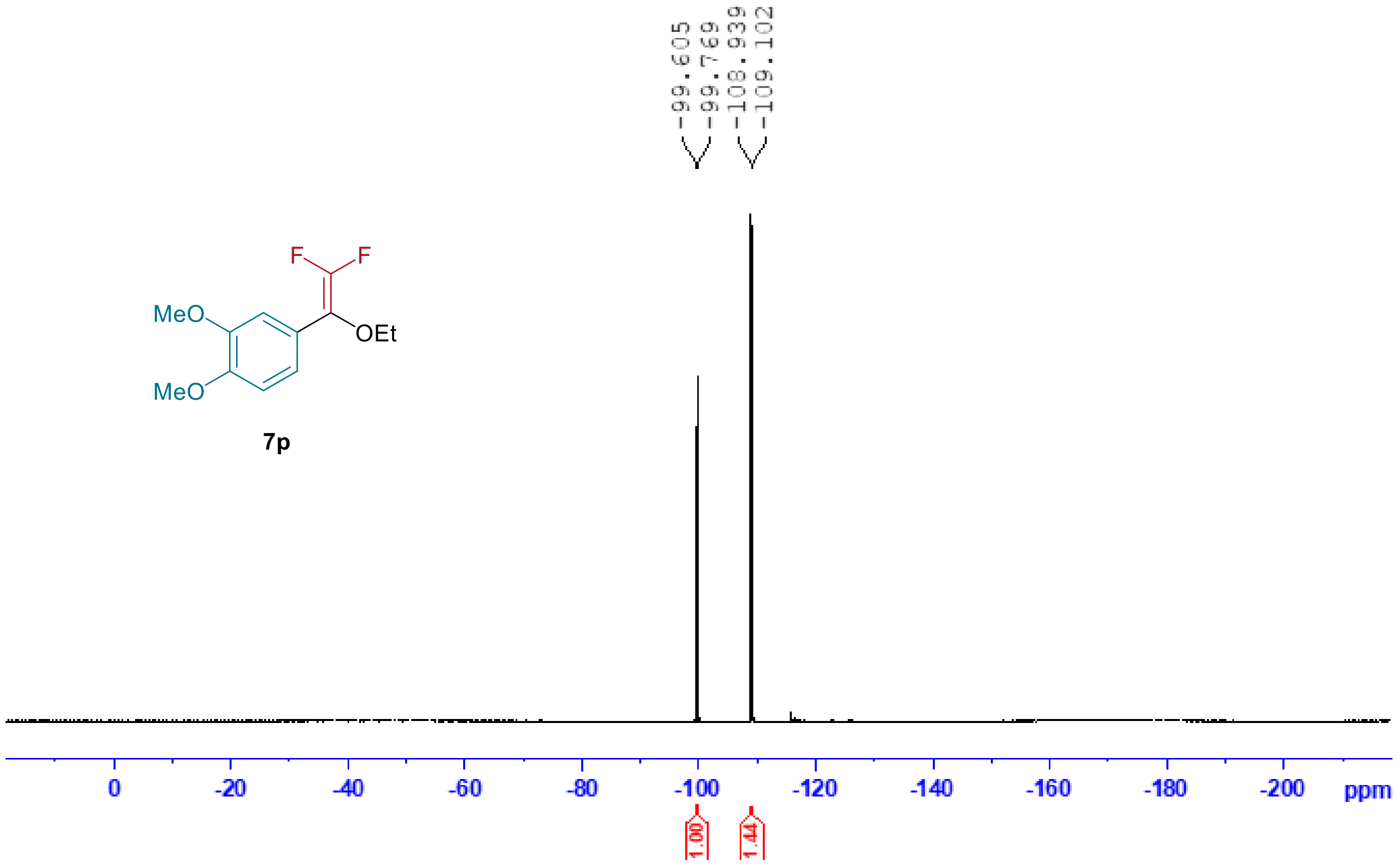
-99.605
-99.769
-108.939
-109.102
F
F
MeO
OEt
MeO
7p
0
-20
-40
-60
-80
-100
-120
-140
-160
-180
-200
ppm
1.00
1.44

^{1}H NMR of **7q**

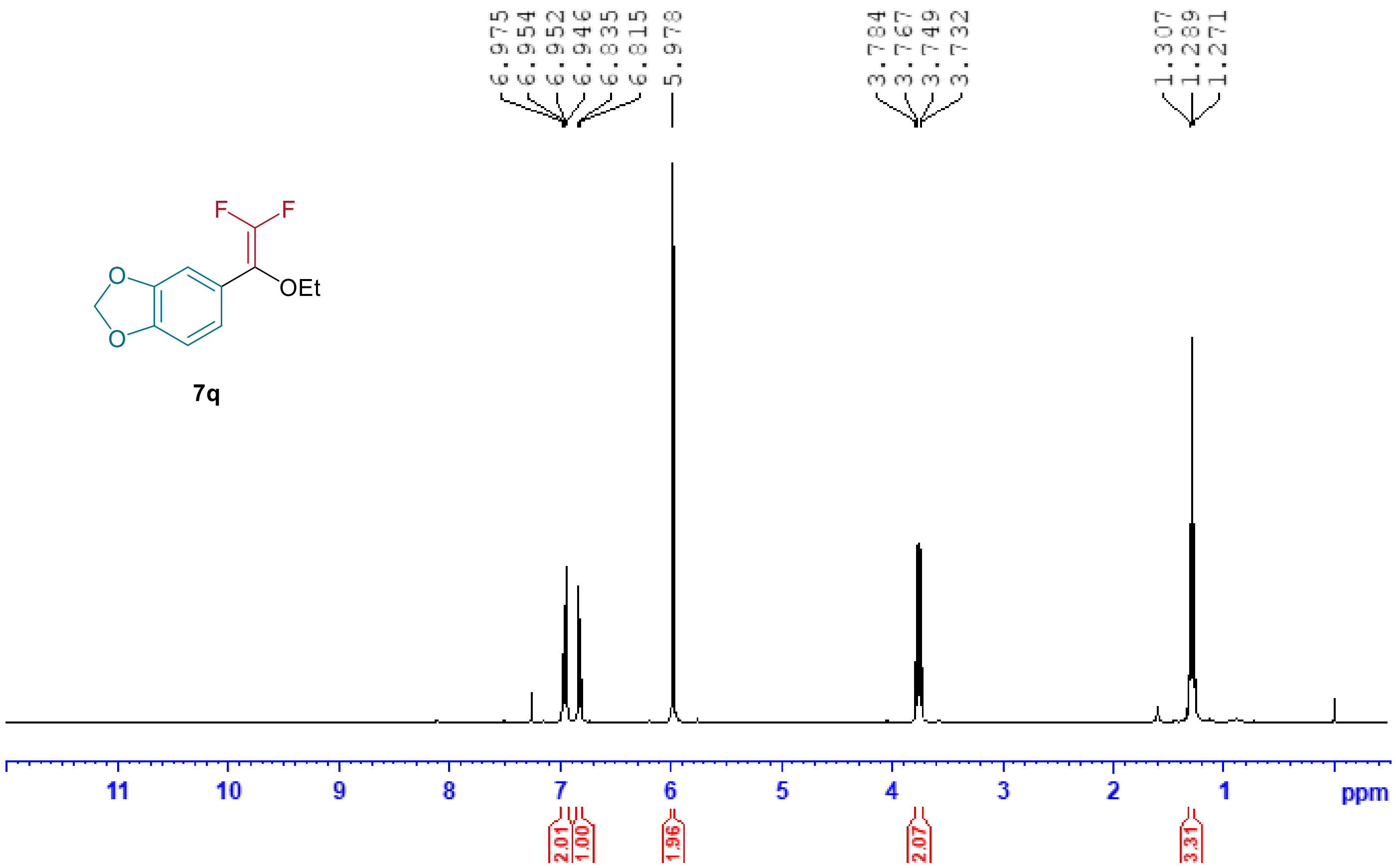

^{13}C NMR of **7q**

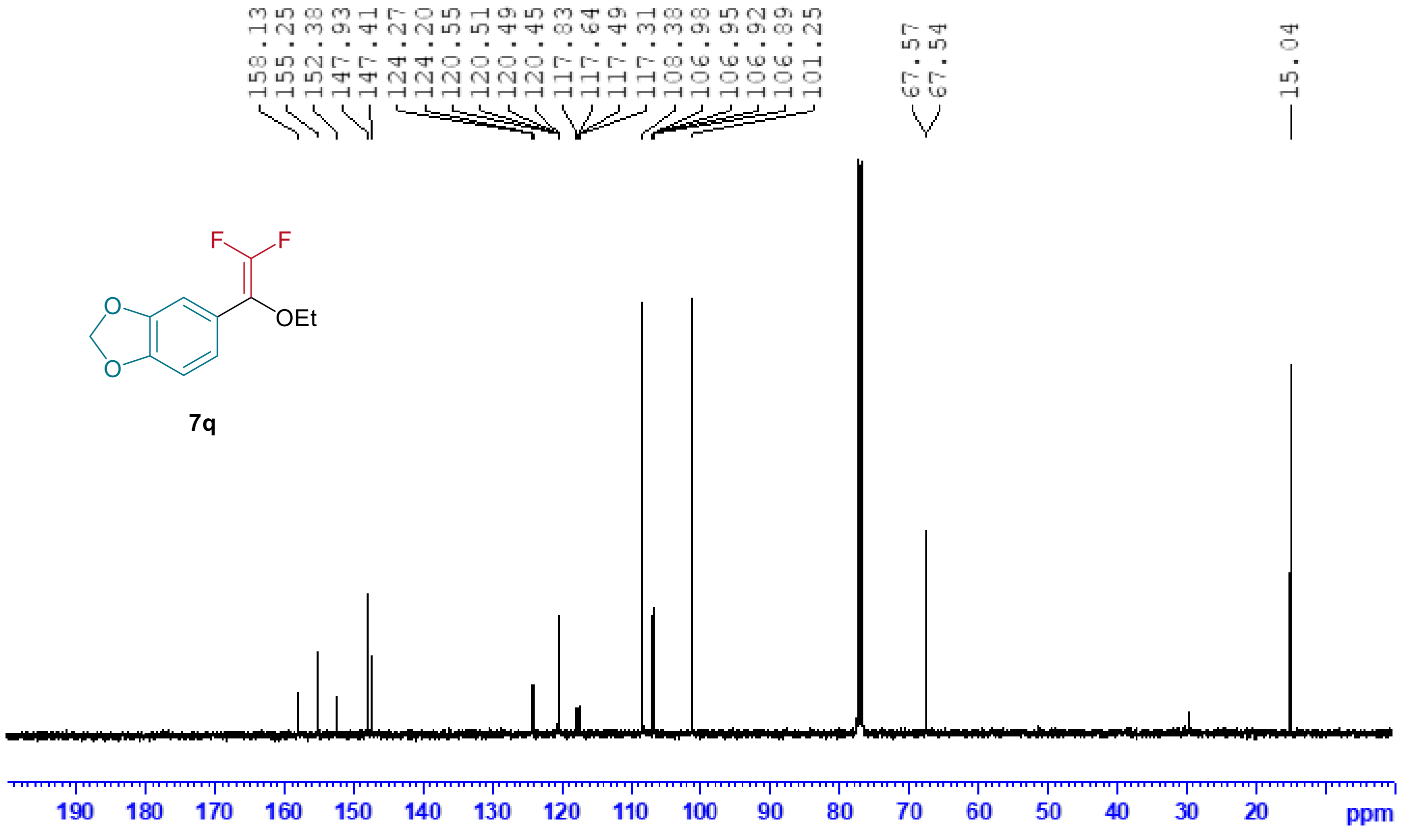

^{19}F NMR of **7q**

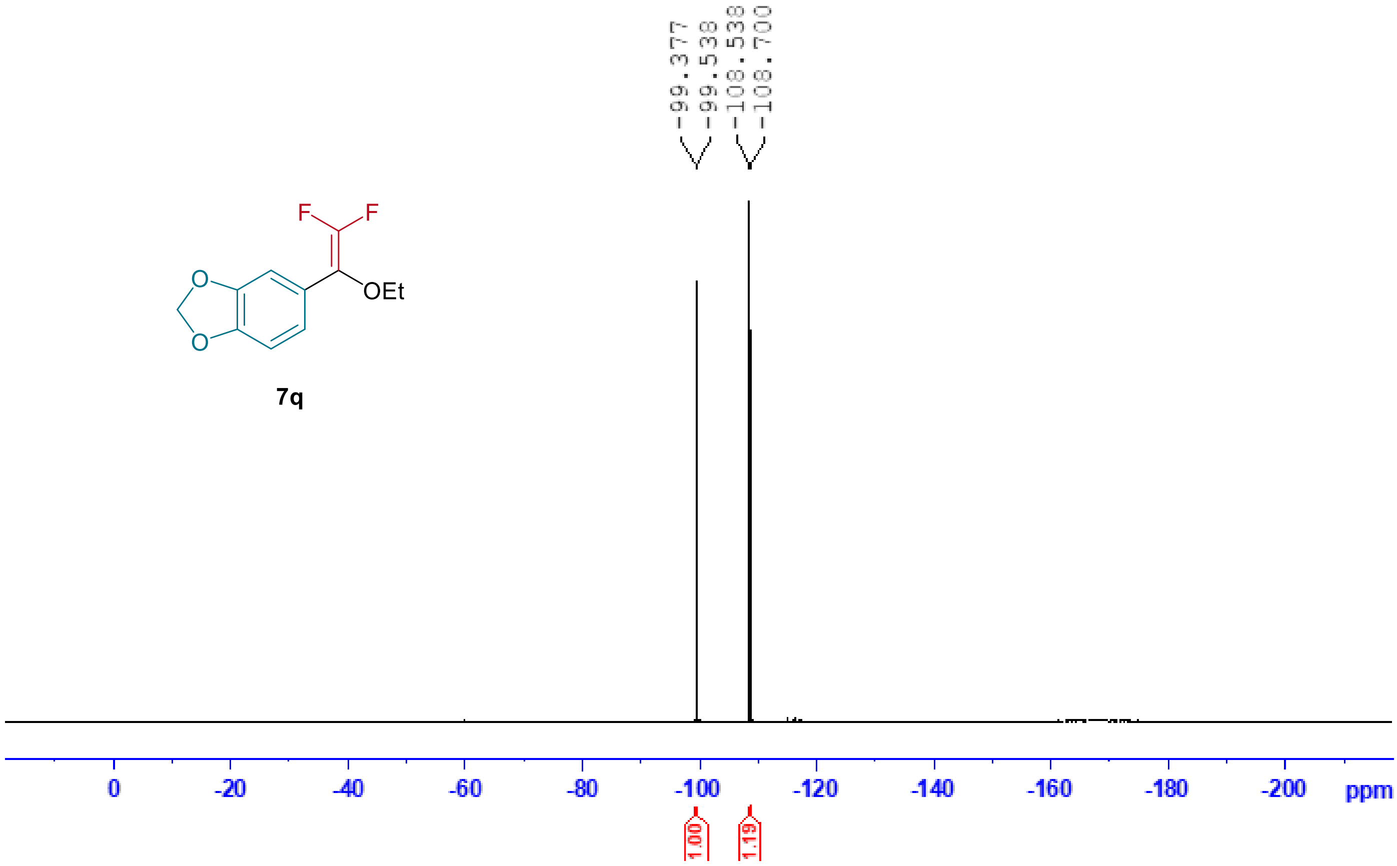

F
F
O
O
OEt
7q
-99.377
-99.538
-108.538
-108.700
0
-20
-40
-60
-80
-100
-120
-140
-160
-180
-200
ppm
1.00
1.19

^{1}H NMR of **7r**

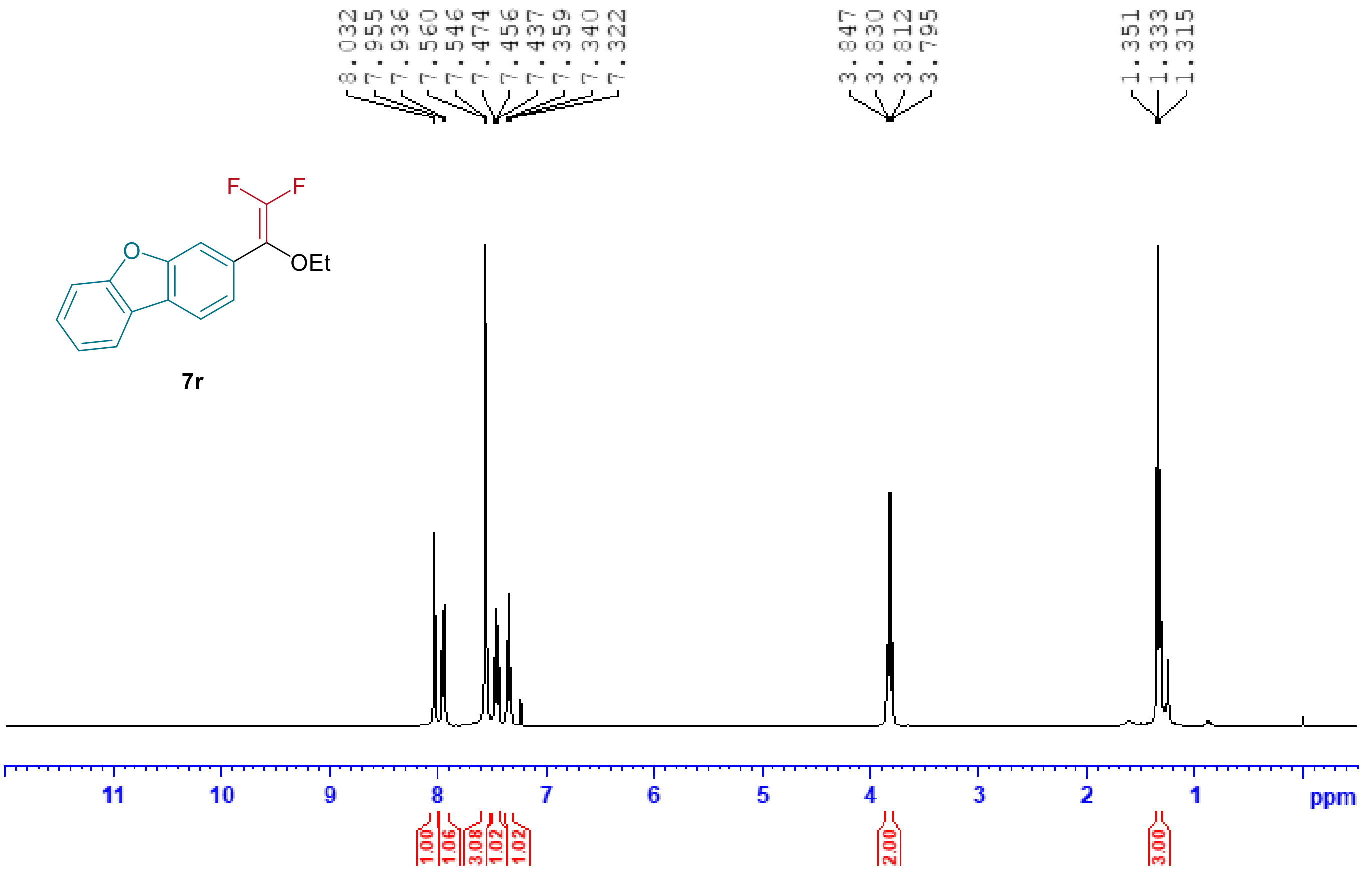

^{13}C NMR of **7r**

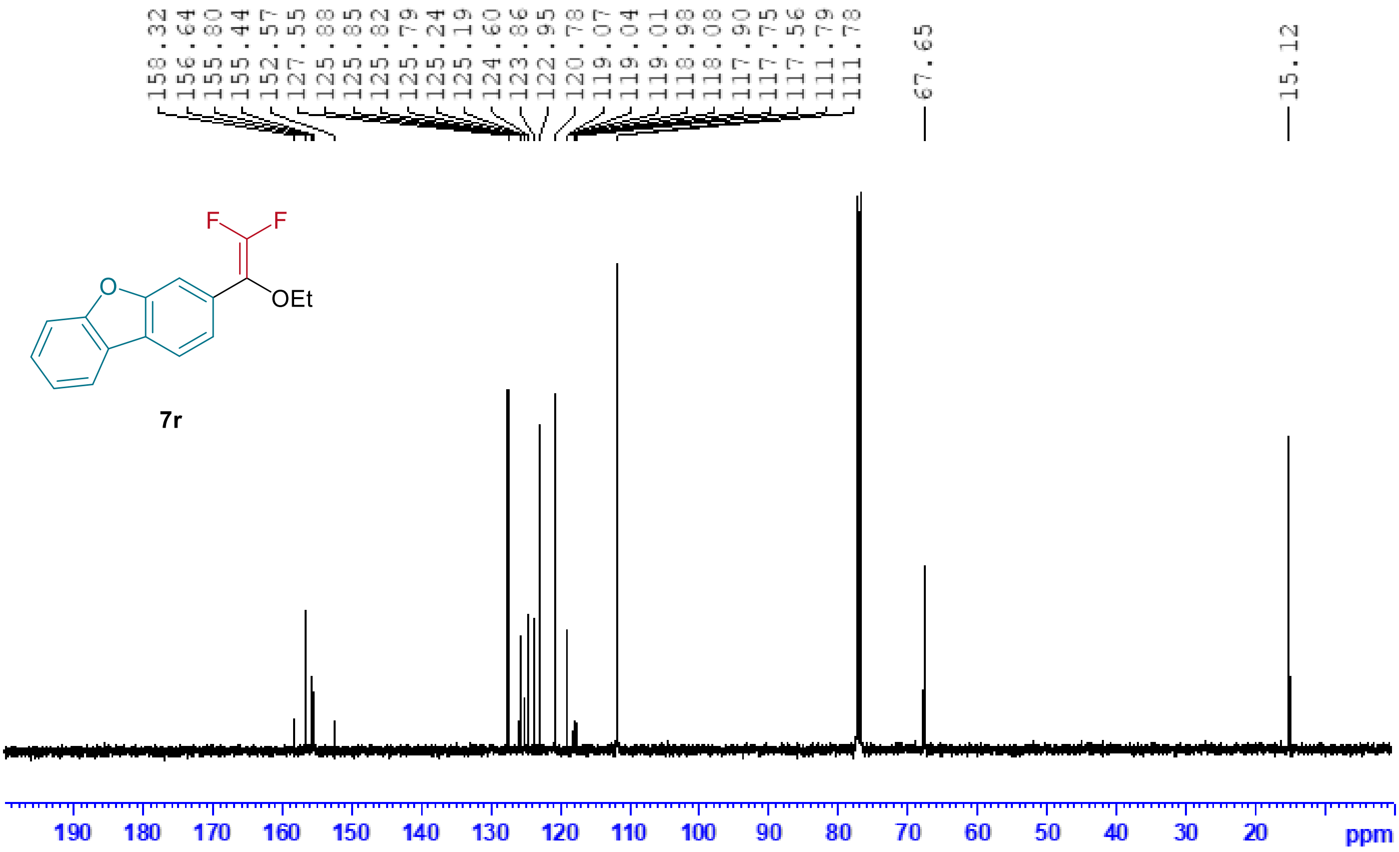

^{19}F NMR of **7r**

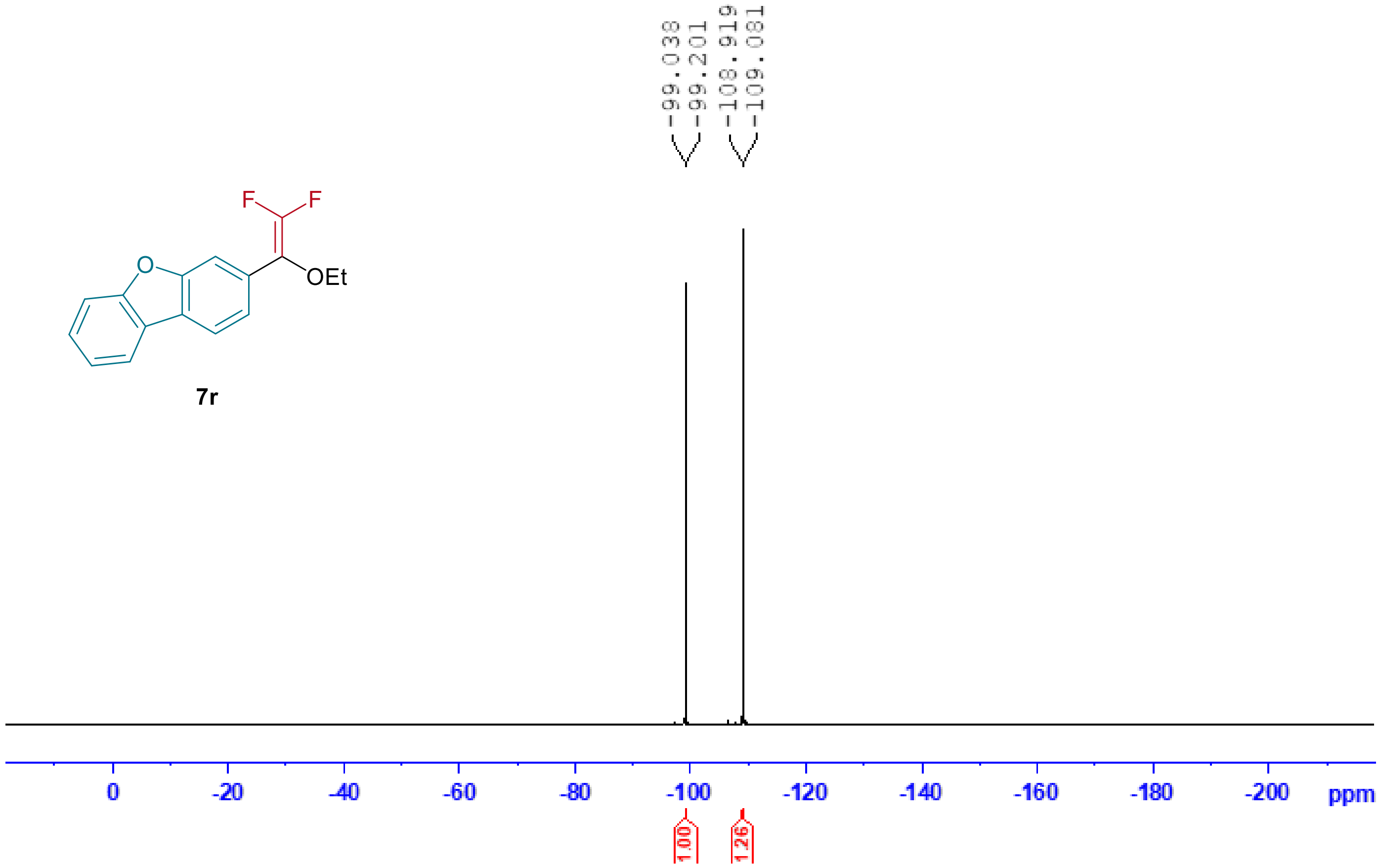

-99.038
-99.201
-108.919
-109.081
F
F
O
OEt
7r
0
-20
-40
-60
-80
-100
-120
-140
-160
-180
-200
ppm
1.00
1.26

^{1}H NMR of **7s**

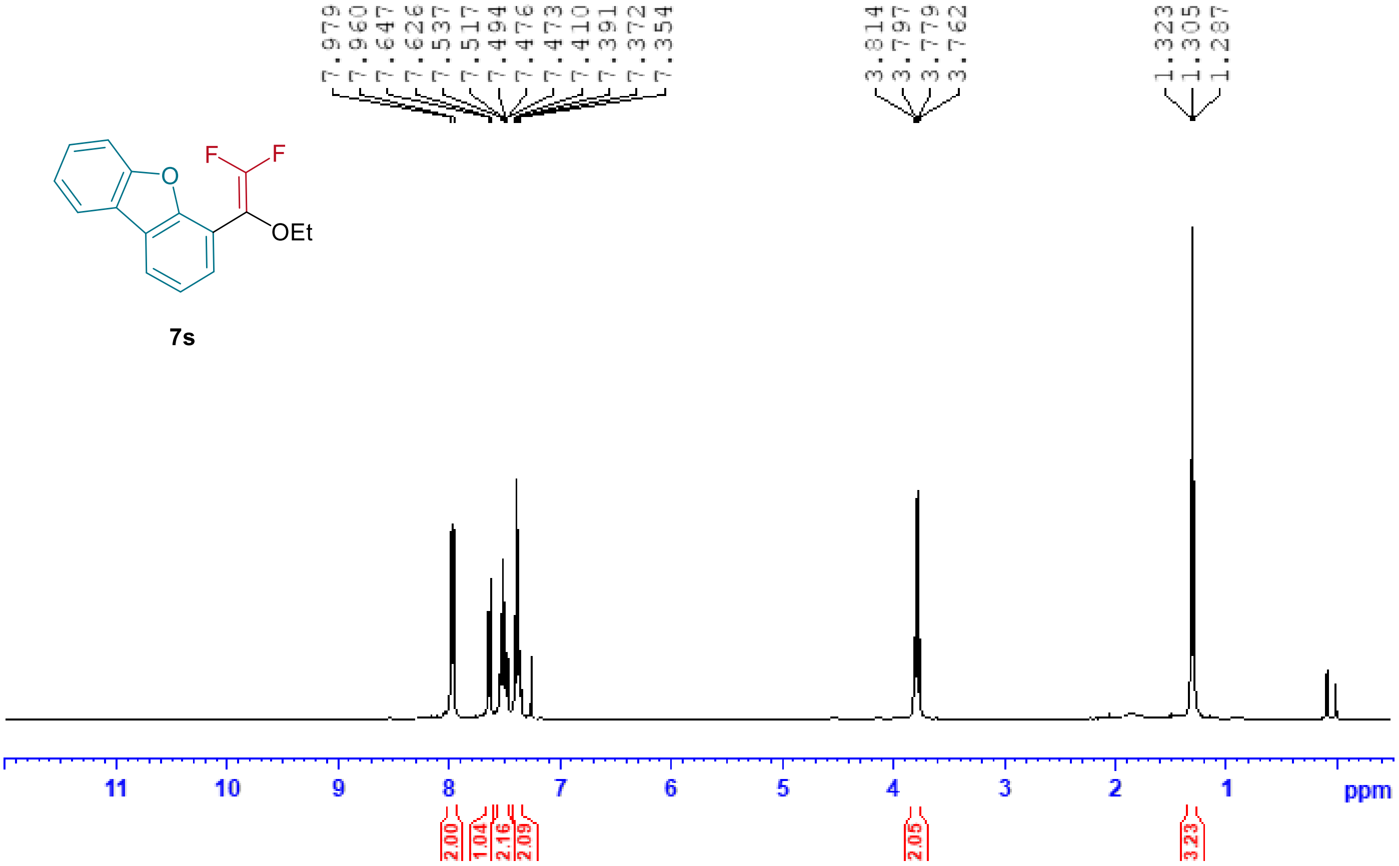

^{13}C NMR of **7s**

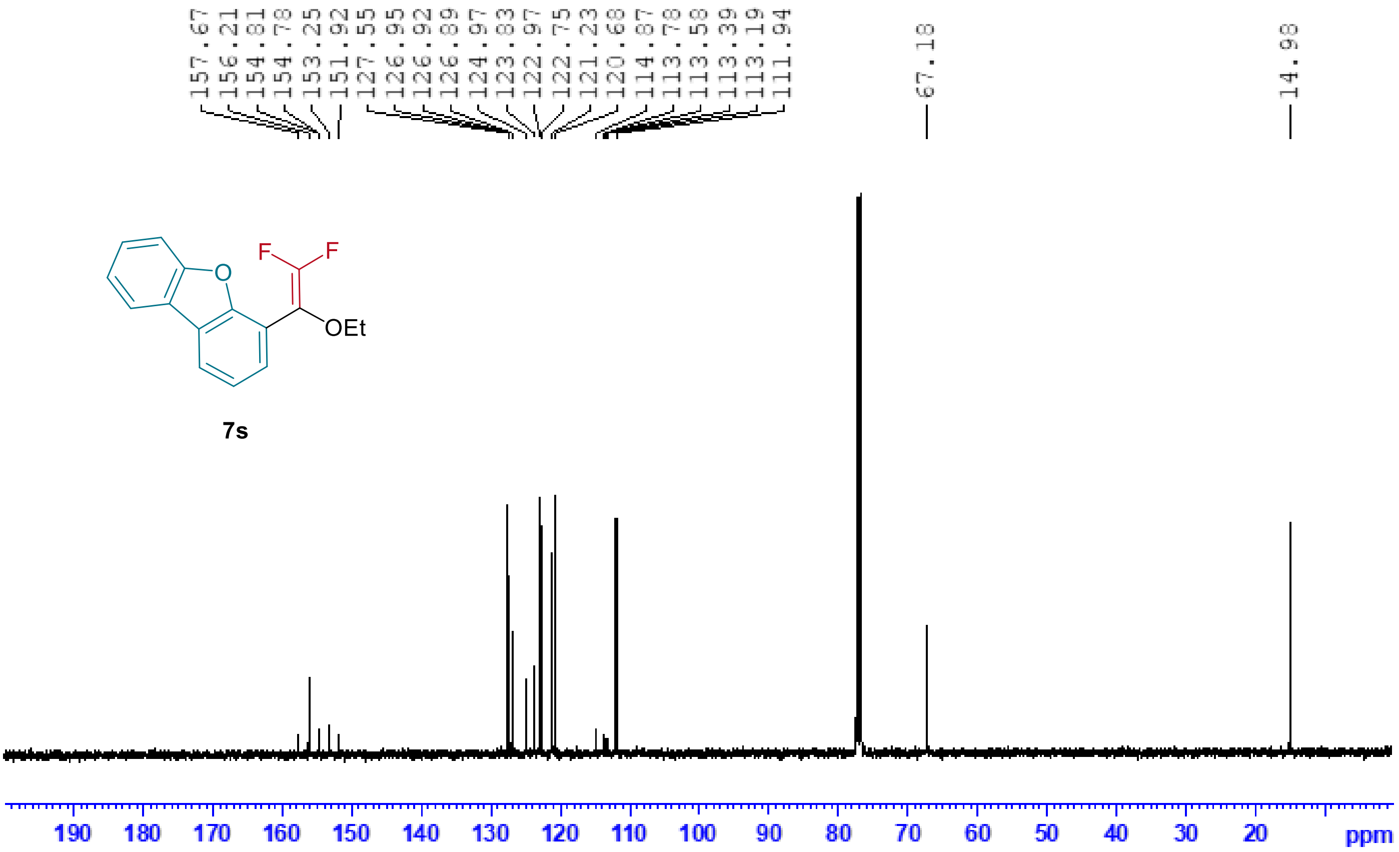

^{19}F NMR of **7s**

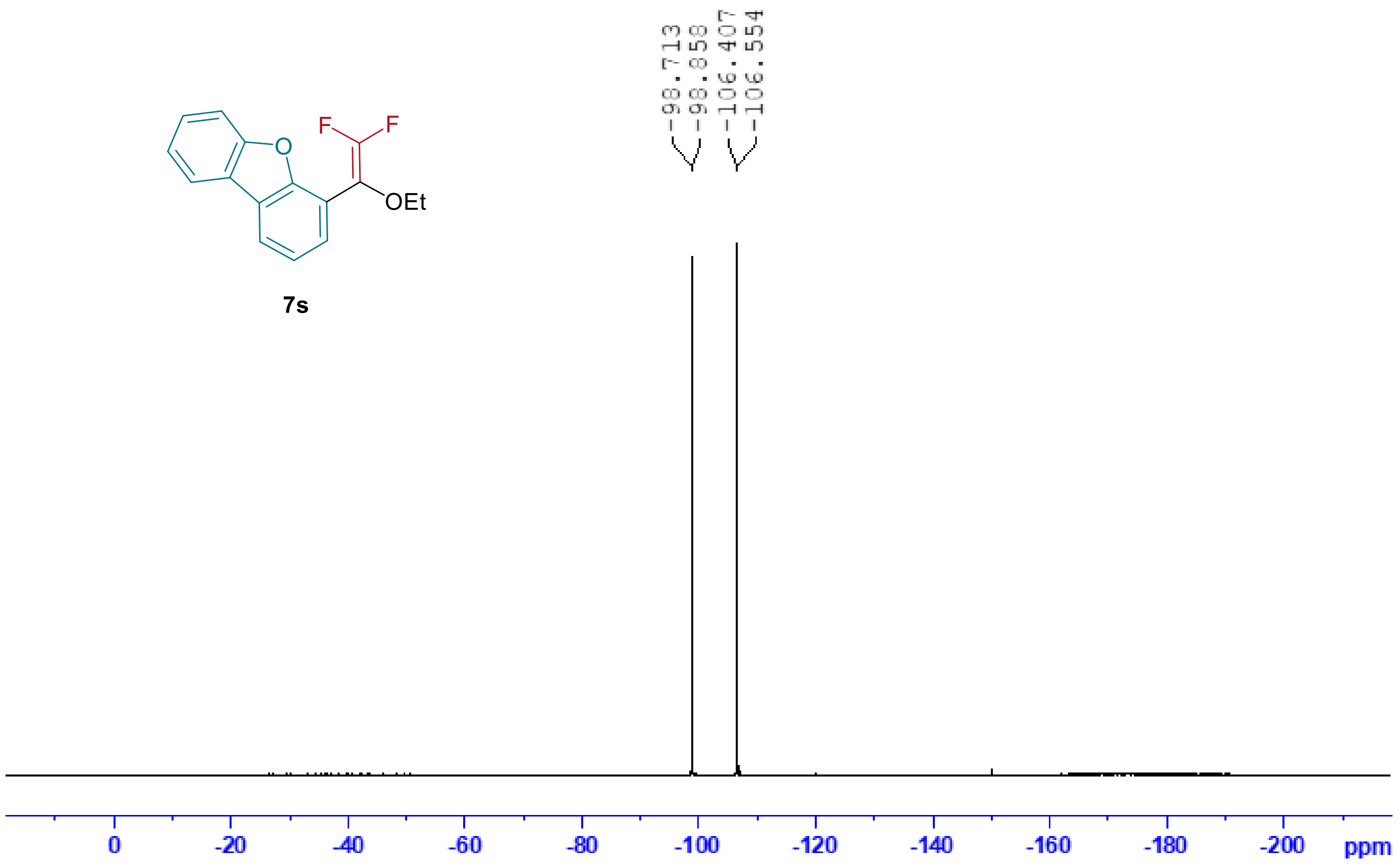

F
F
O
OEt
7s
-98.713
-98.858
-106.407
-106.554
0
-20
-40
-60
-80
-100
-120
-140
-160
-180
-200
ppm

^{1}H NMR of **7t**

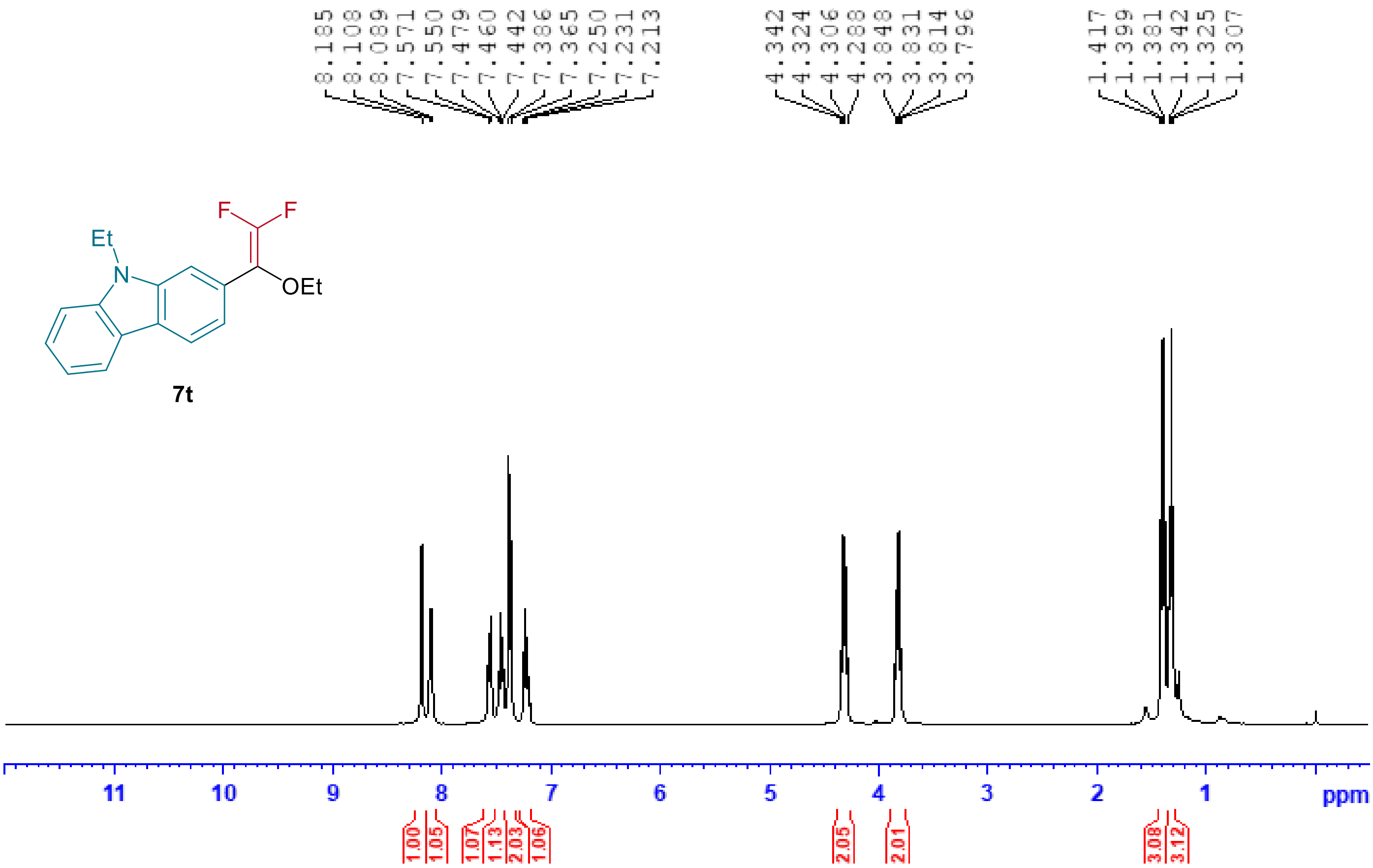

^{13}C NMR of **7t**

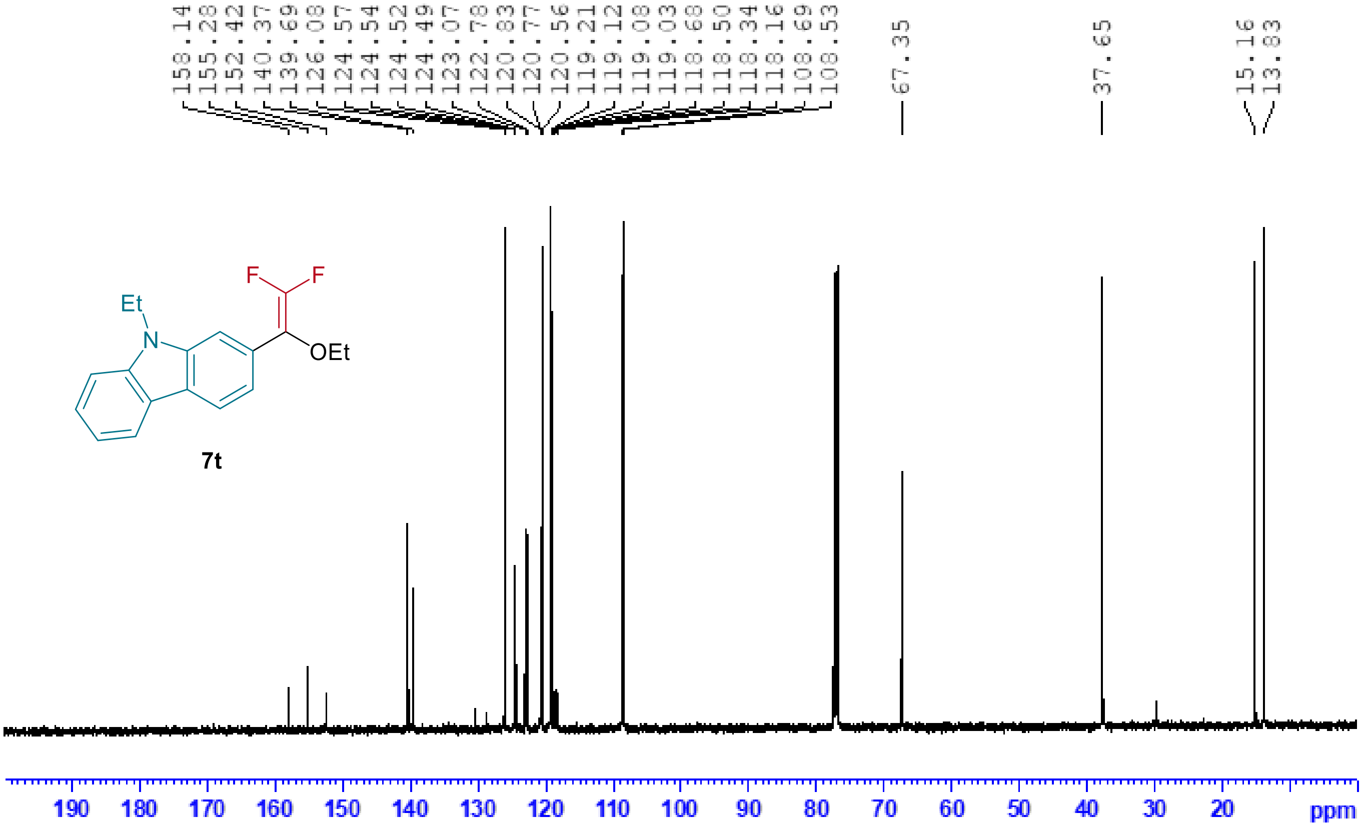

^{19}F NMR of **7t**

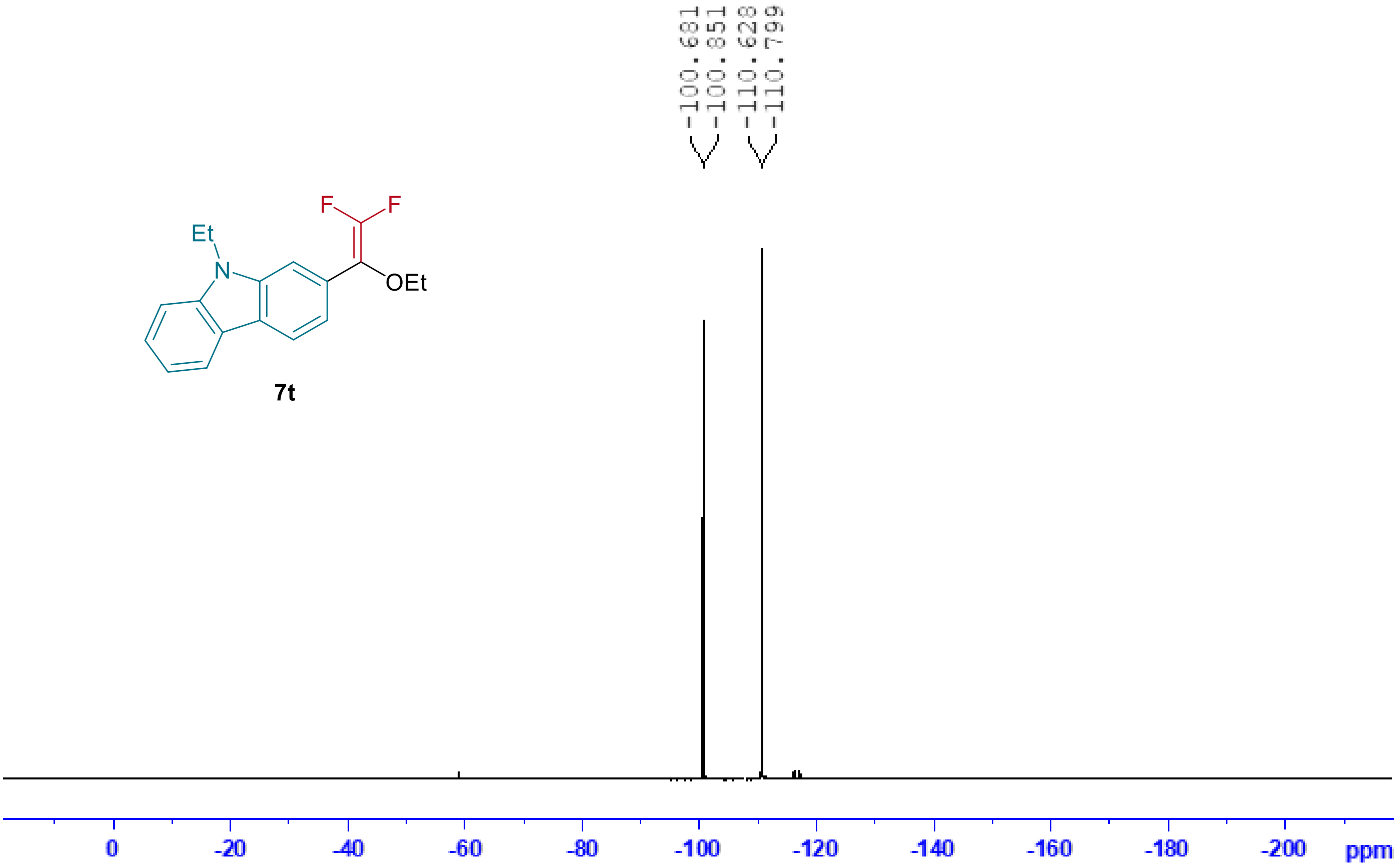

Et
N
F
F
OEt
7t
-100.681
-100.851
-110.628
-110.799
0
-20
-40
-60
-80
-100
-120
-140
-160
-180
-200
ppm

^{1}H NMR of **S-1-2 (Vitamin E OTf)**

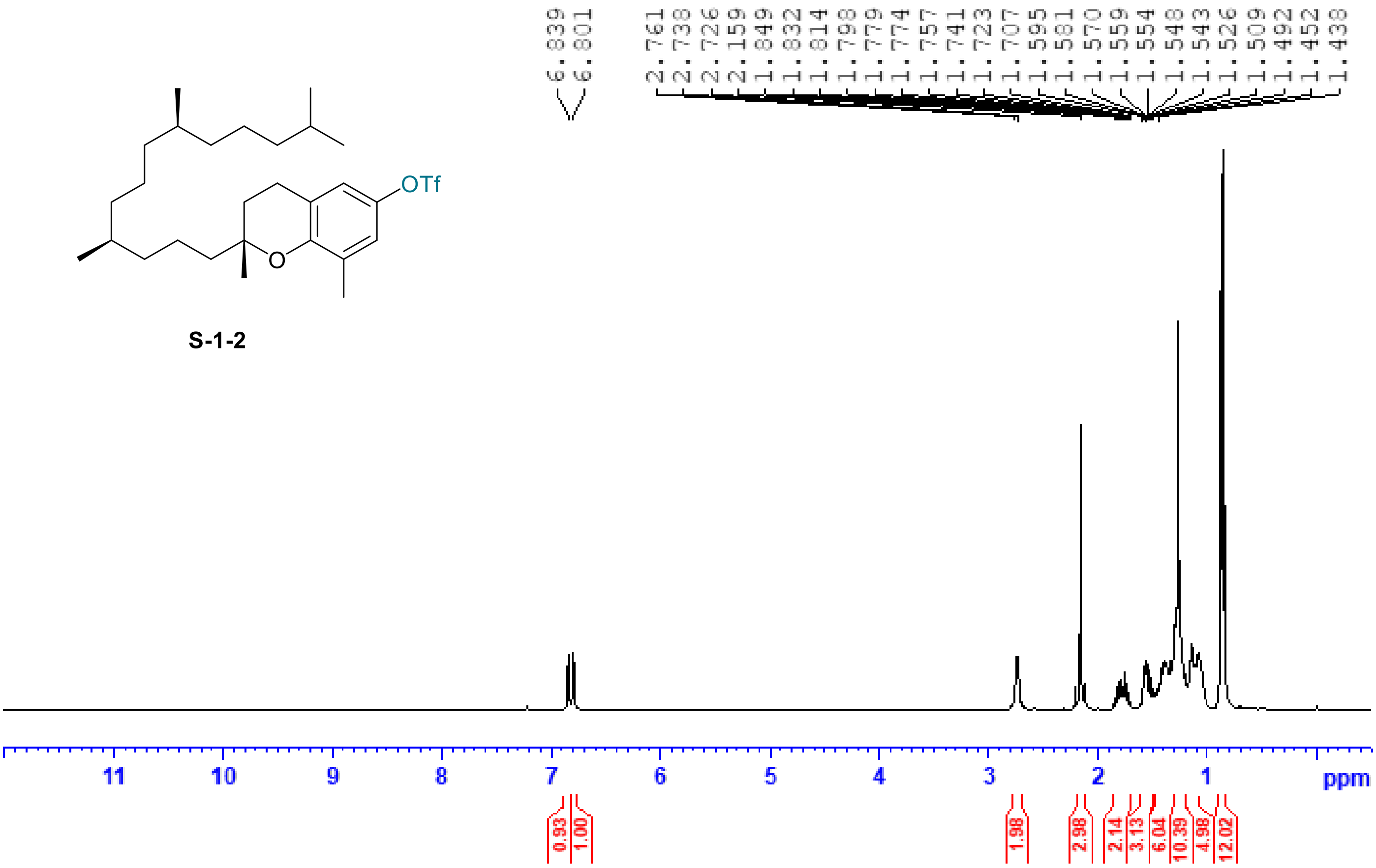

^{13}C NMR of **S-1-2 (Vitamin E OTf)**

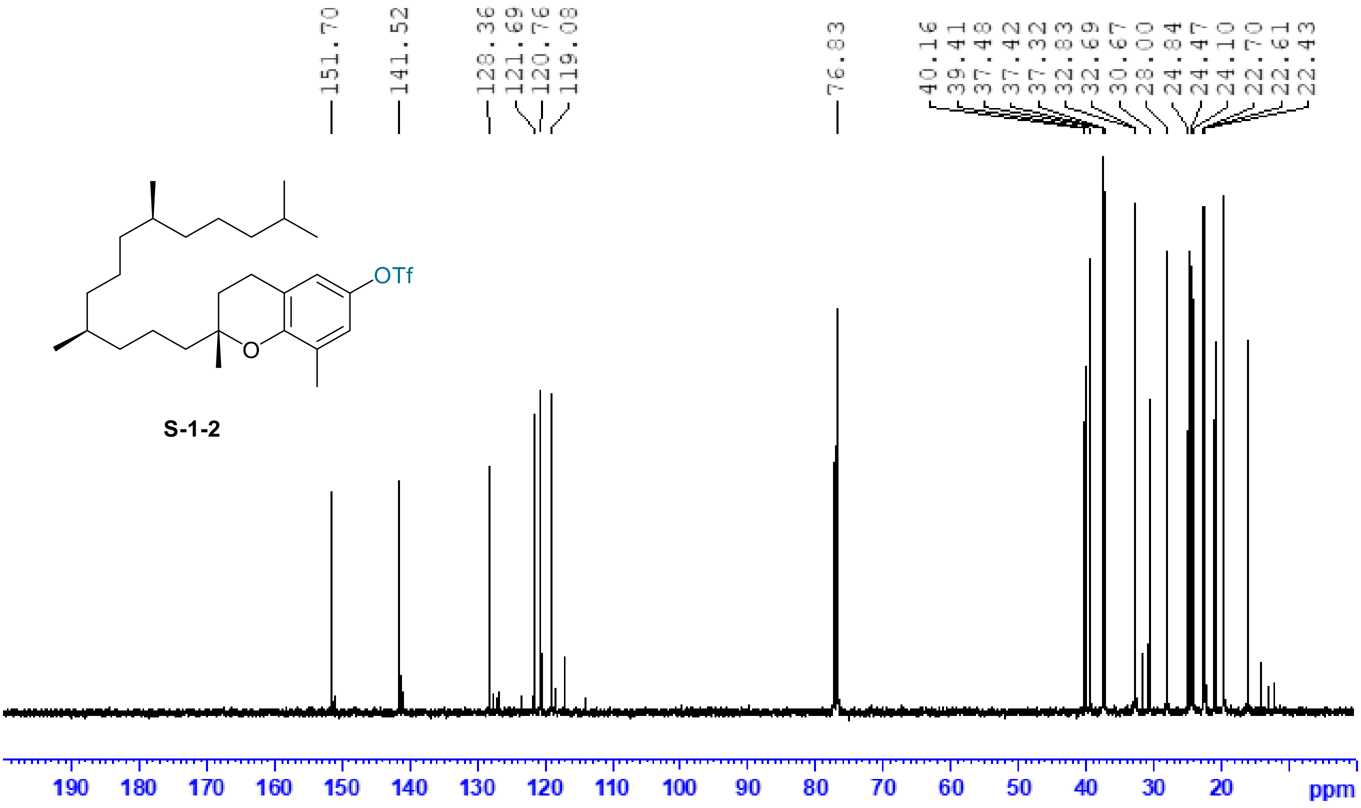

^{19}F NMR of **S-1-2 (Vitamin E OTf)**

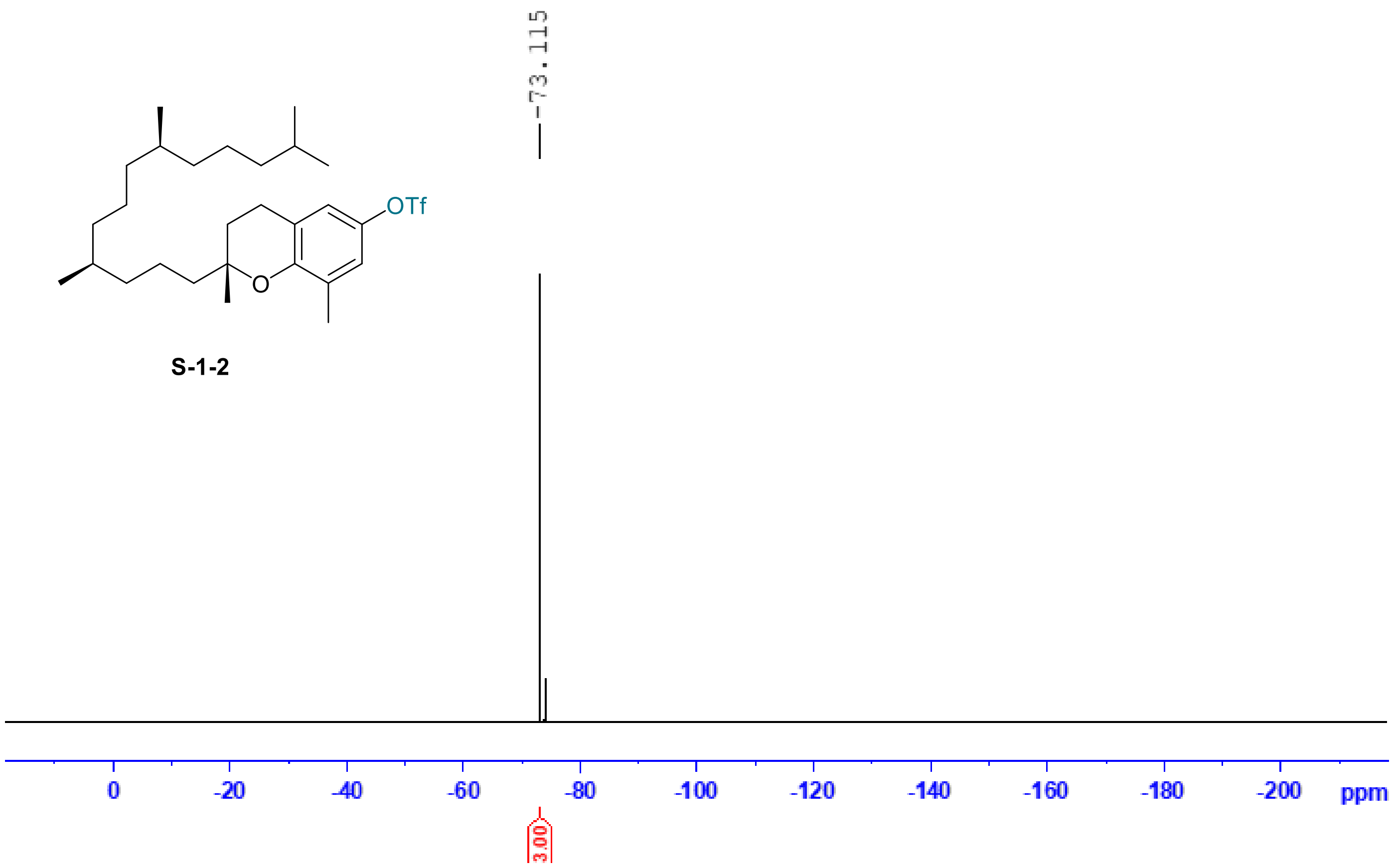

^{1}H NMR of **S-1-3 (Vitamin E Bpin)**

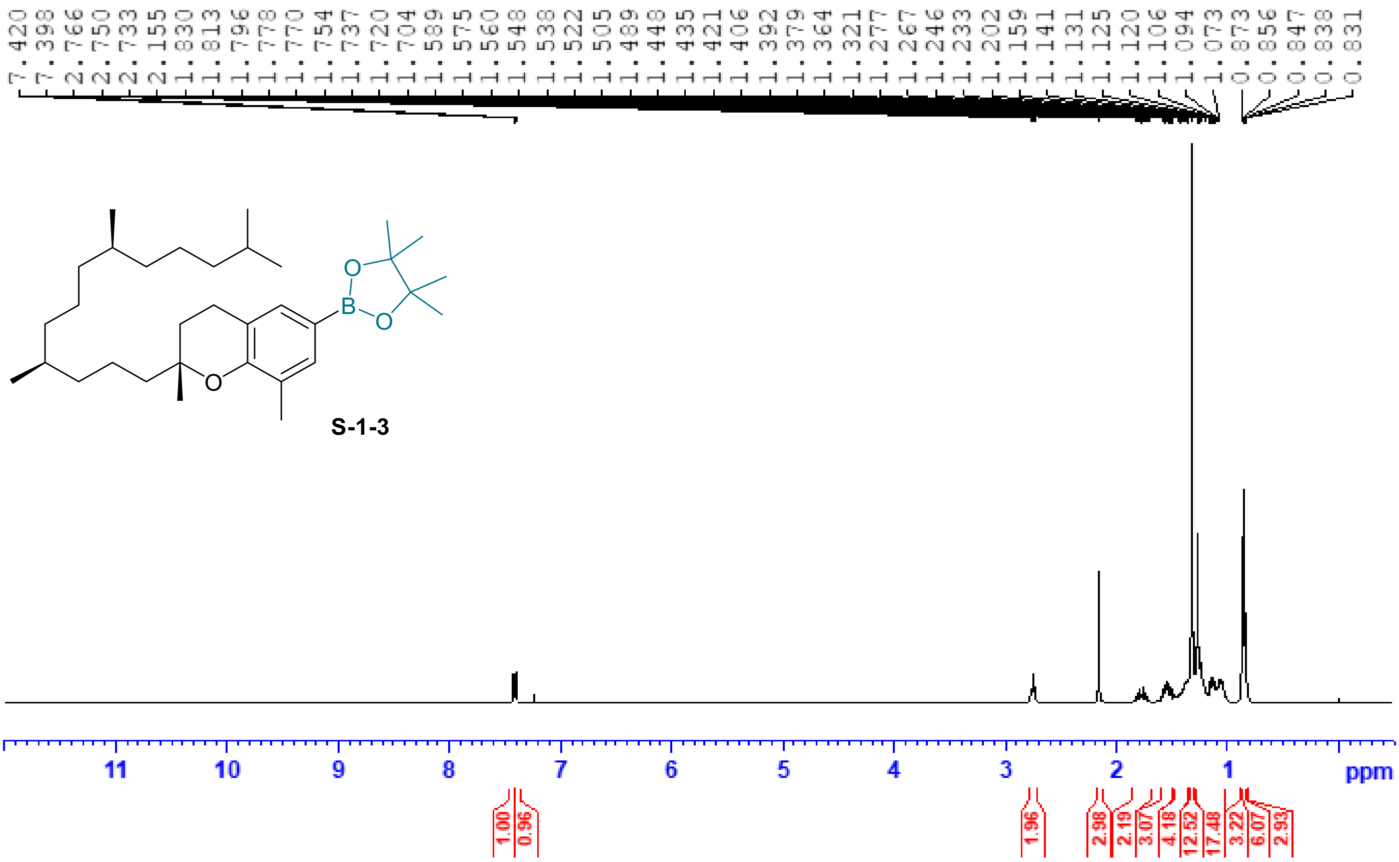

^{13}C NMR of **S-1-3 (Vitamin E Bpin)**

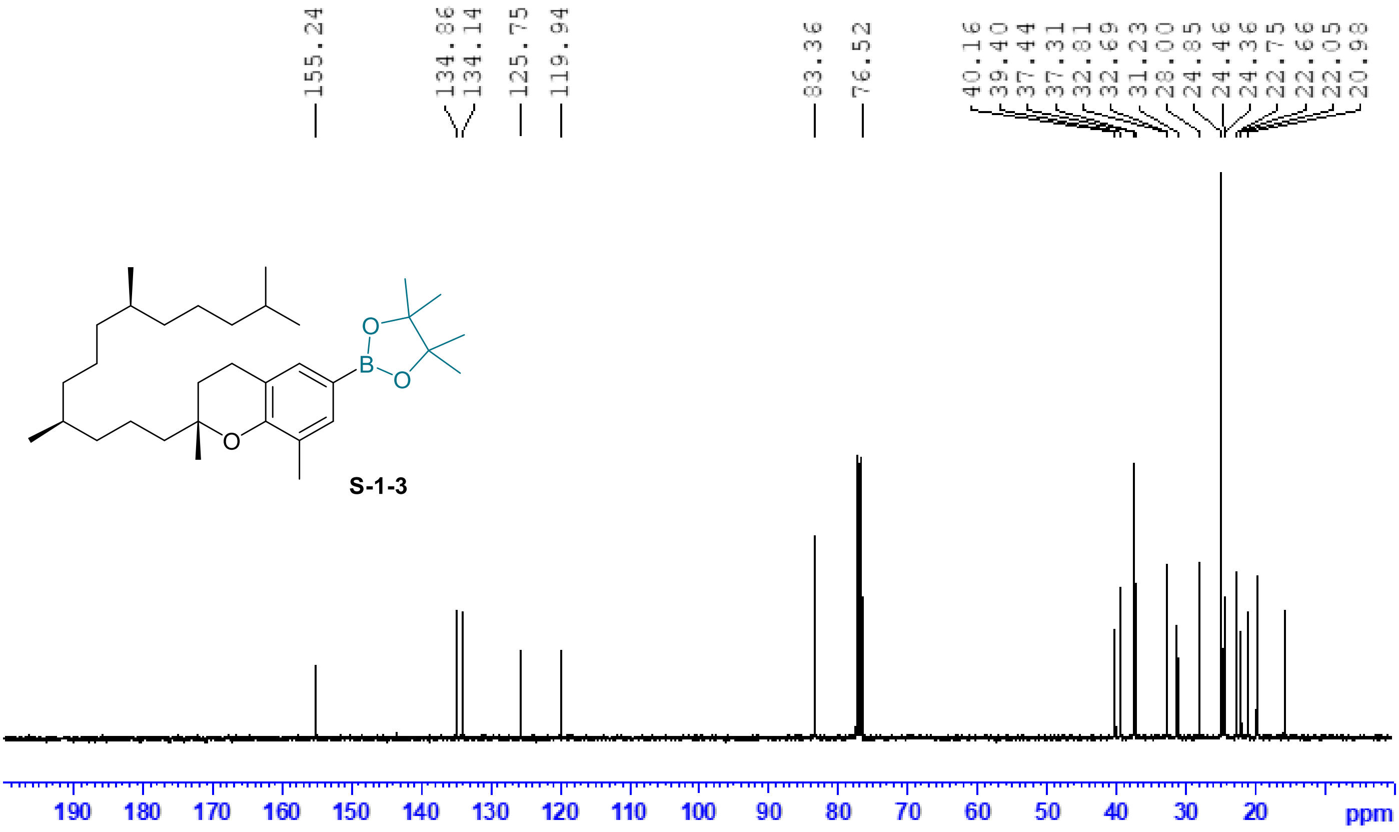

^{1}H NMR of **S-1-4 (Vitamin E B(OH)$_2$)**

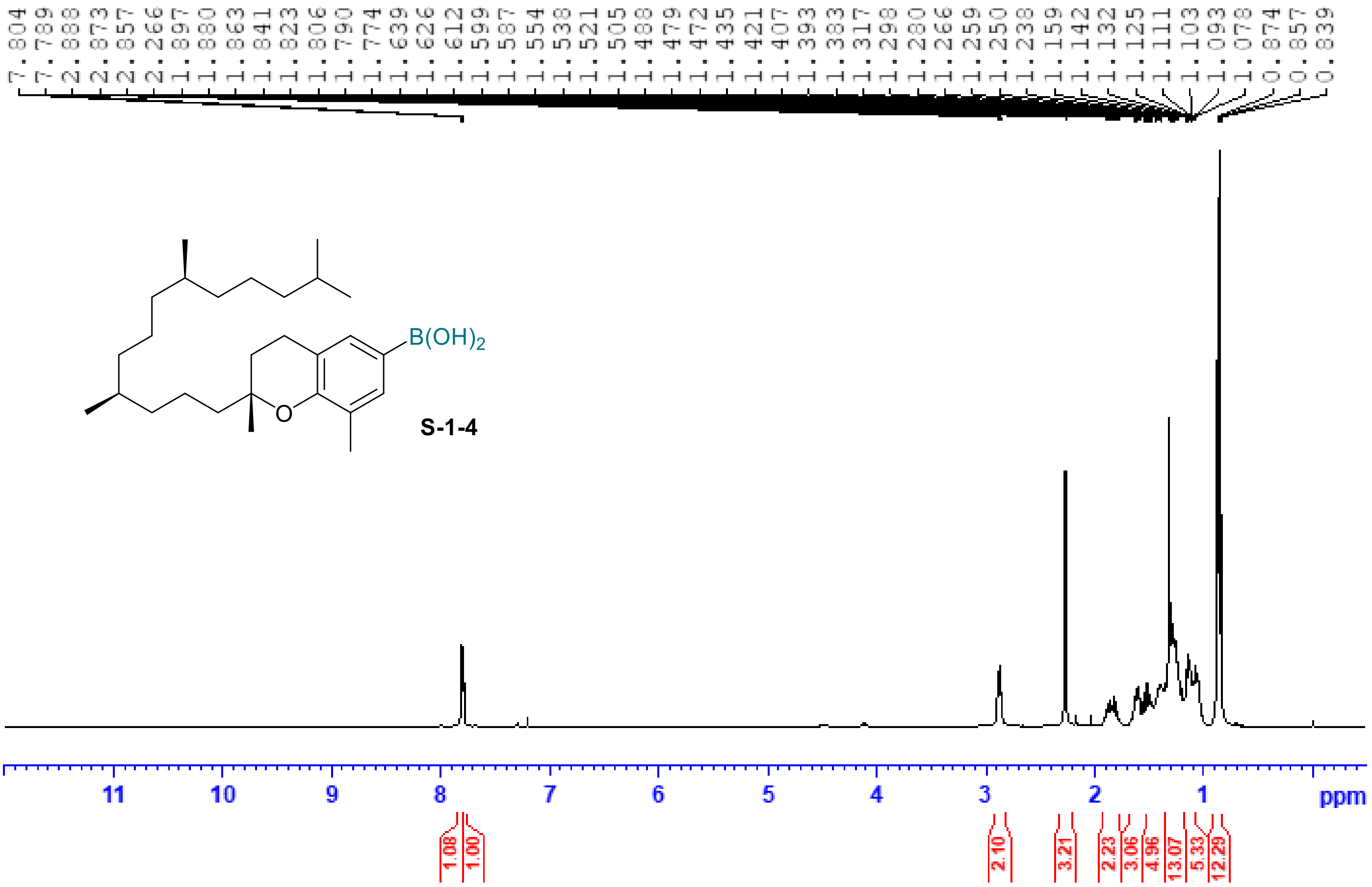

^{13}C NMR of **S-1-4 (Vitamin E B(OH)$_2$)**

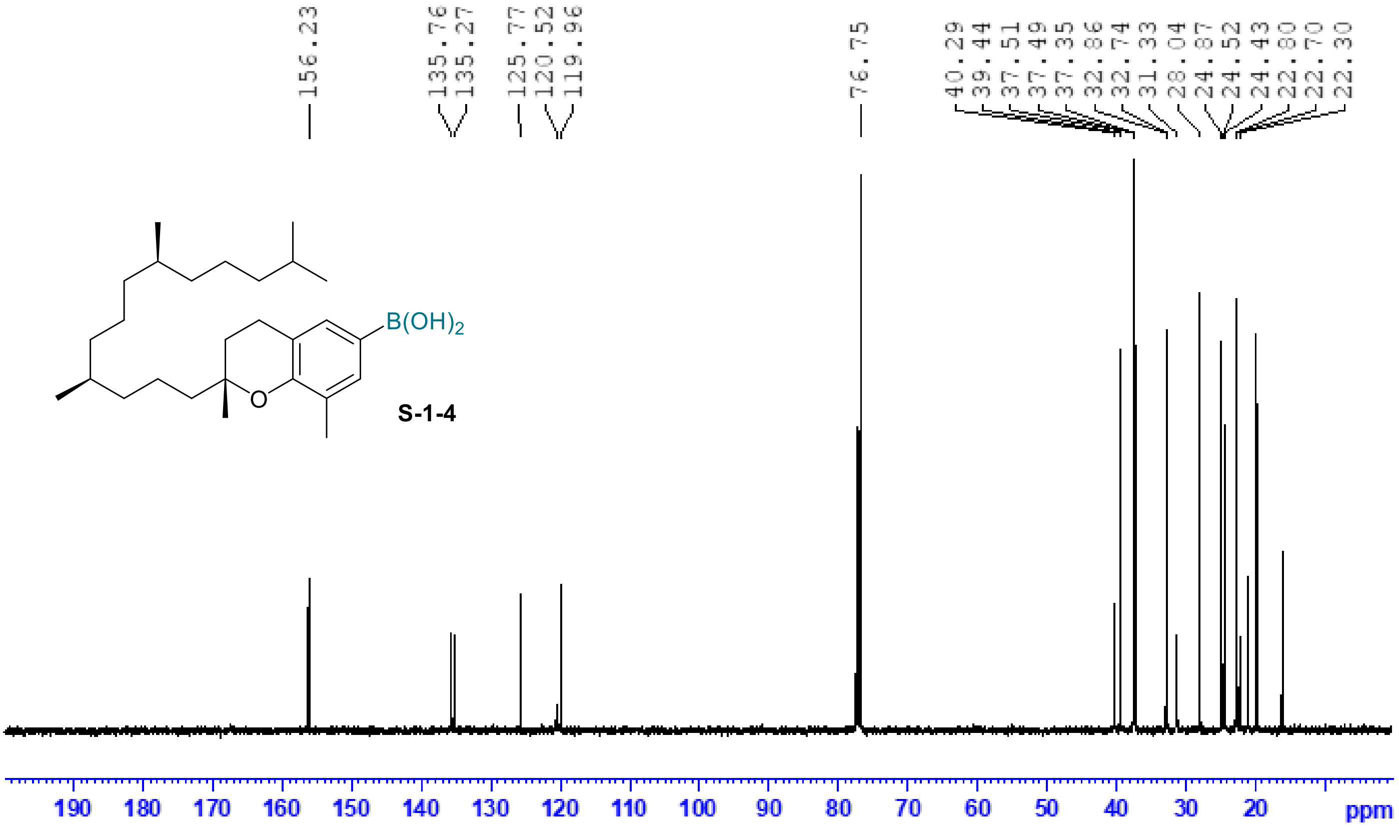

^{1}H NMR of **7u**

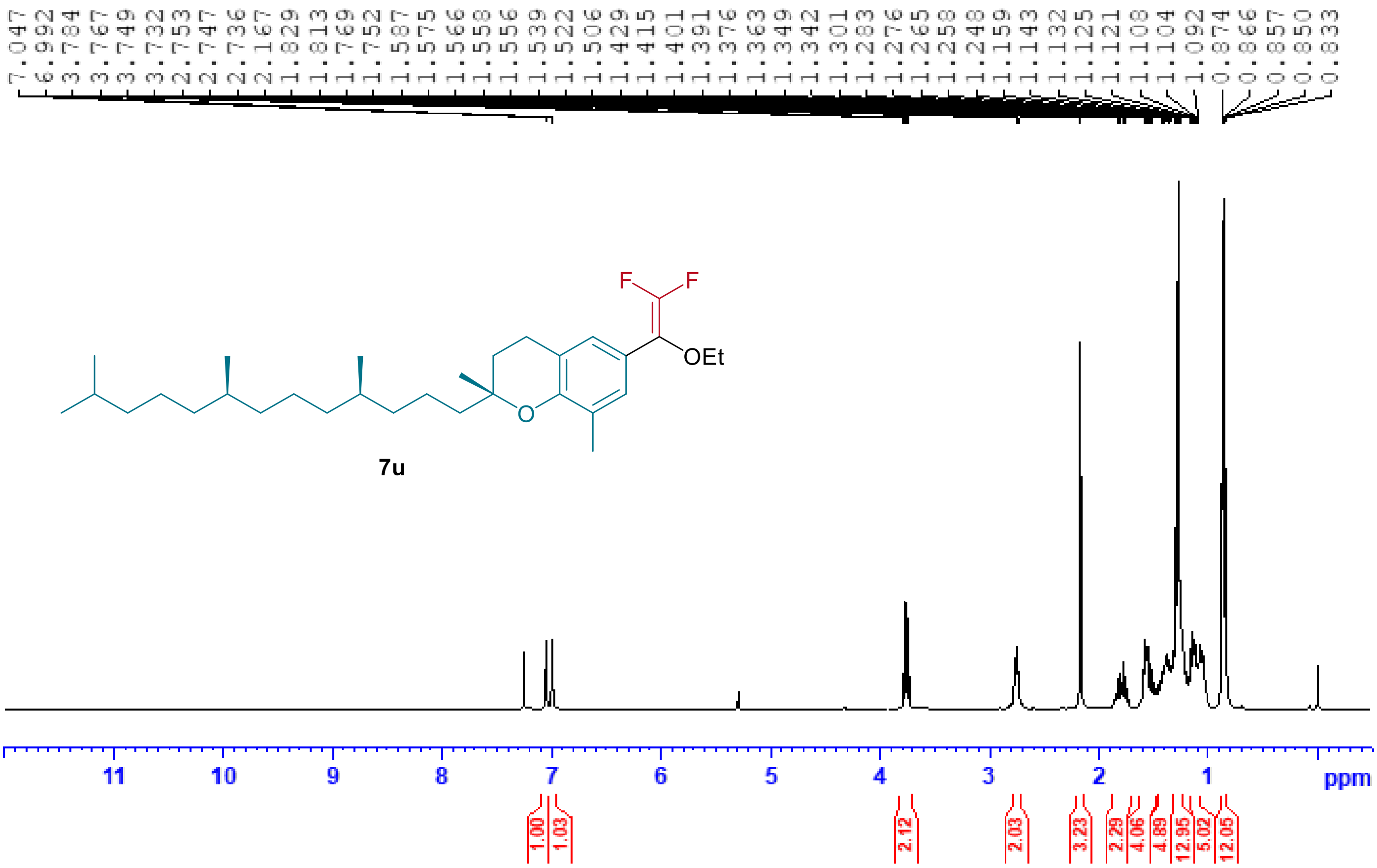

^{13}C NMR of **7u**

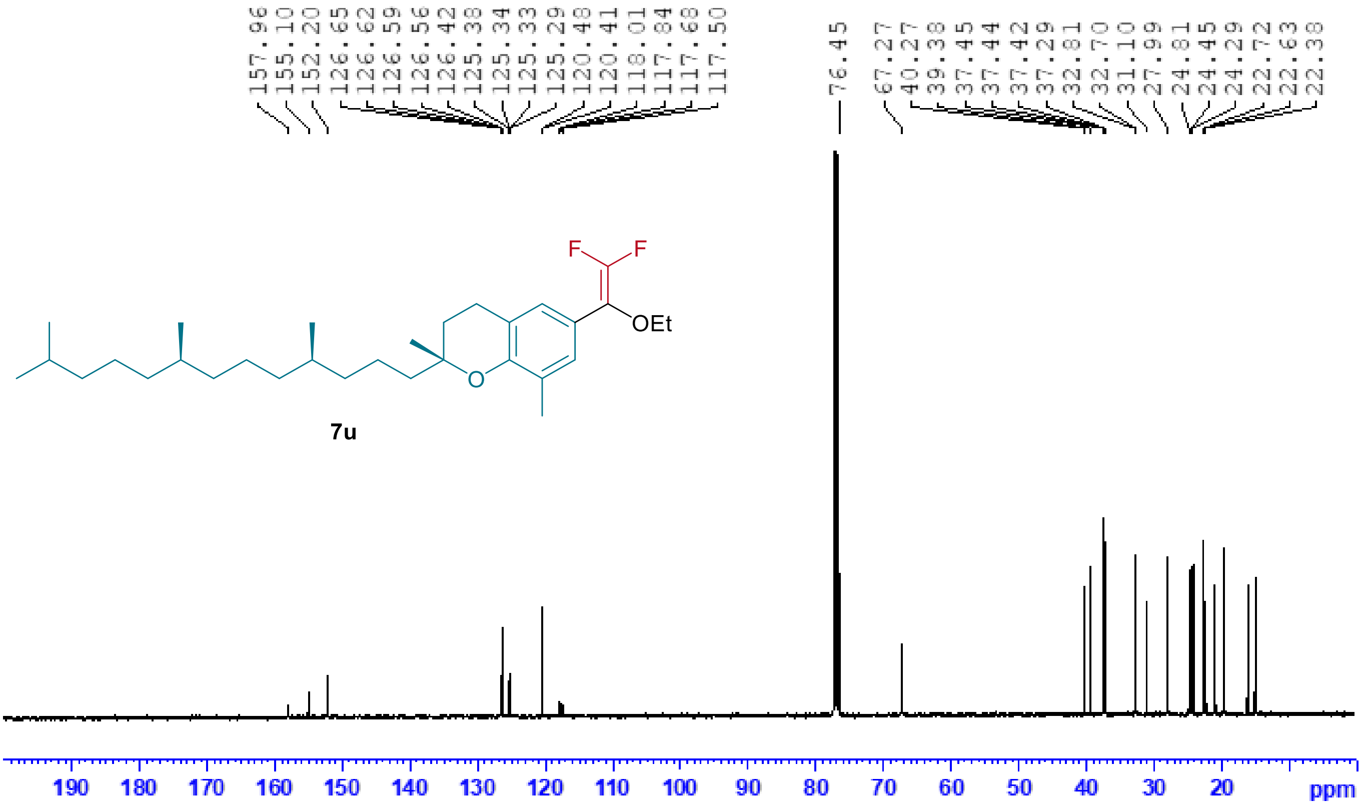

^{19}F NMR of **7u**

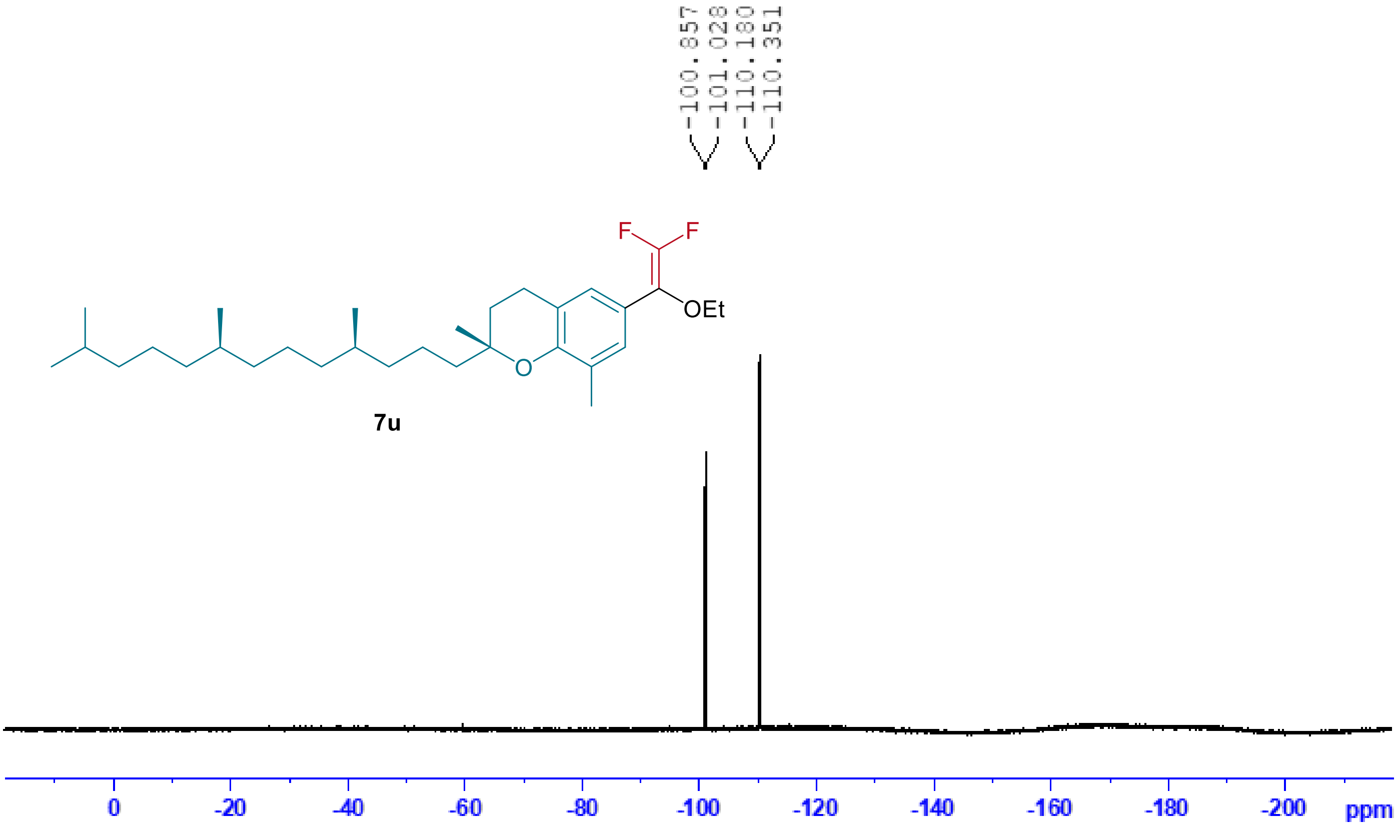

-100.857
-101.028
-110.180
-110.351
F
F
OEt
O
7u
0
-20
-40
-60
-80
-100
-120
-140
-160
-180
-200
ppm

^{1}H NMR of **S-3-2 (Estrone OTf)**

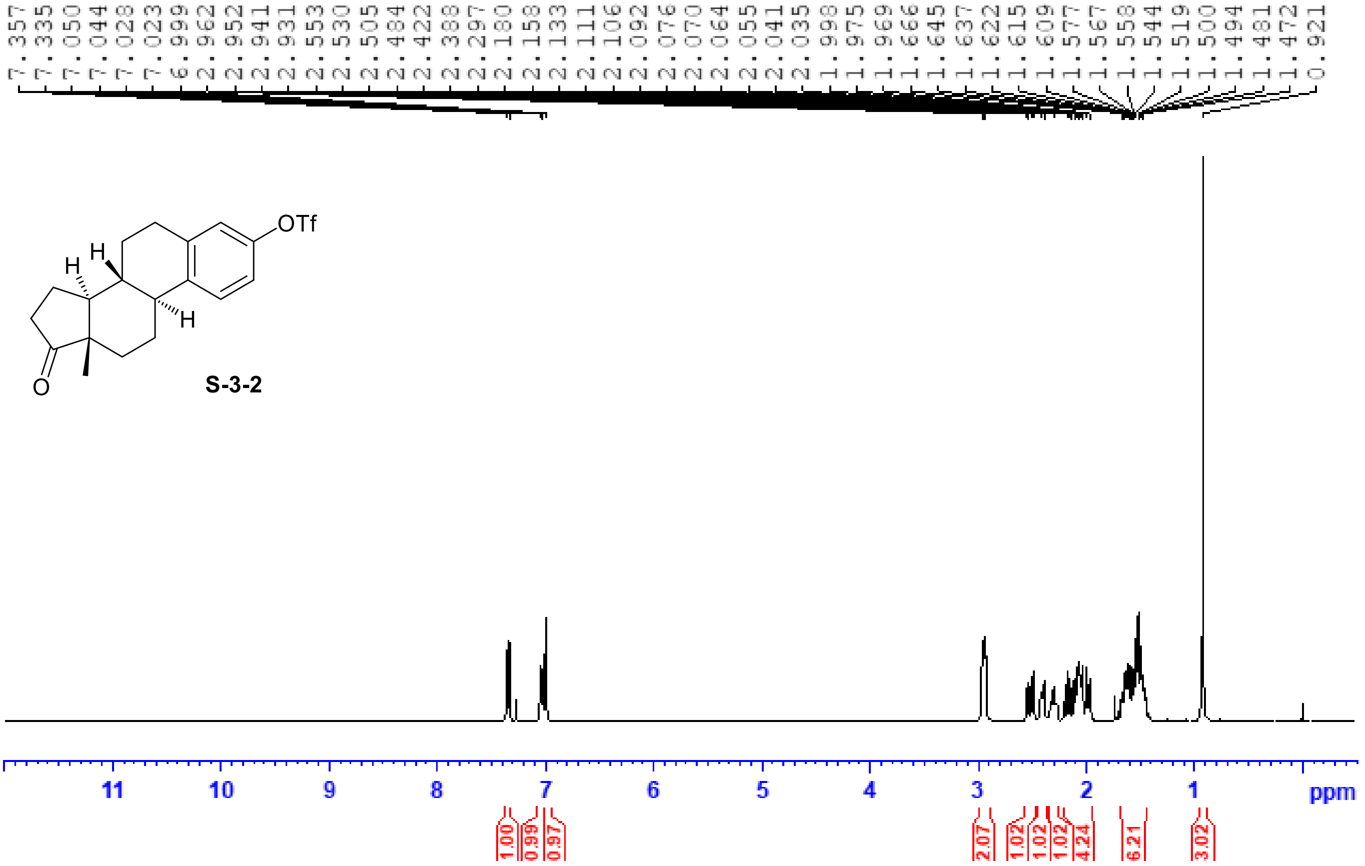

^{13}C NMR of **S-3-2 (Estrone OTf)**

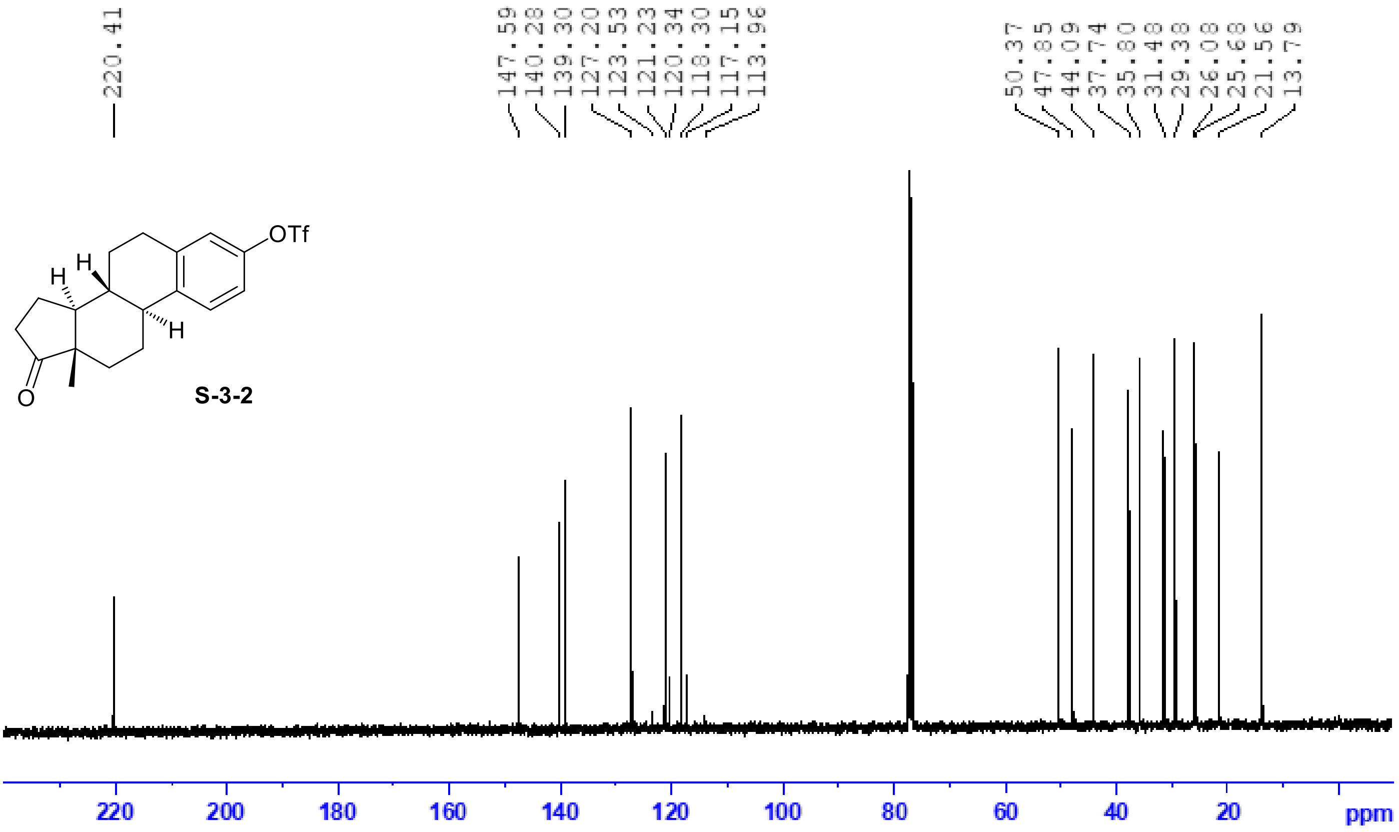

^{19}F NMR of **S-3-2 (Estrone OTf)**

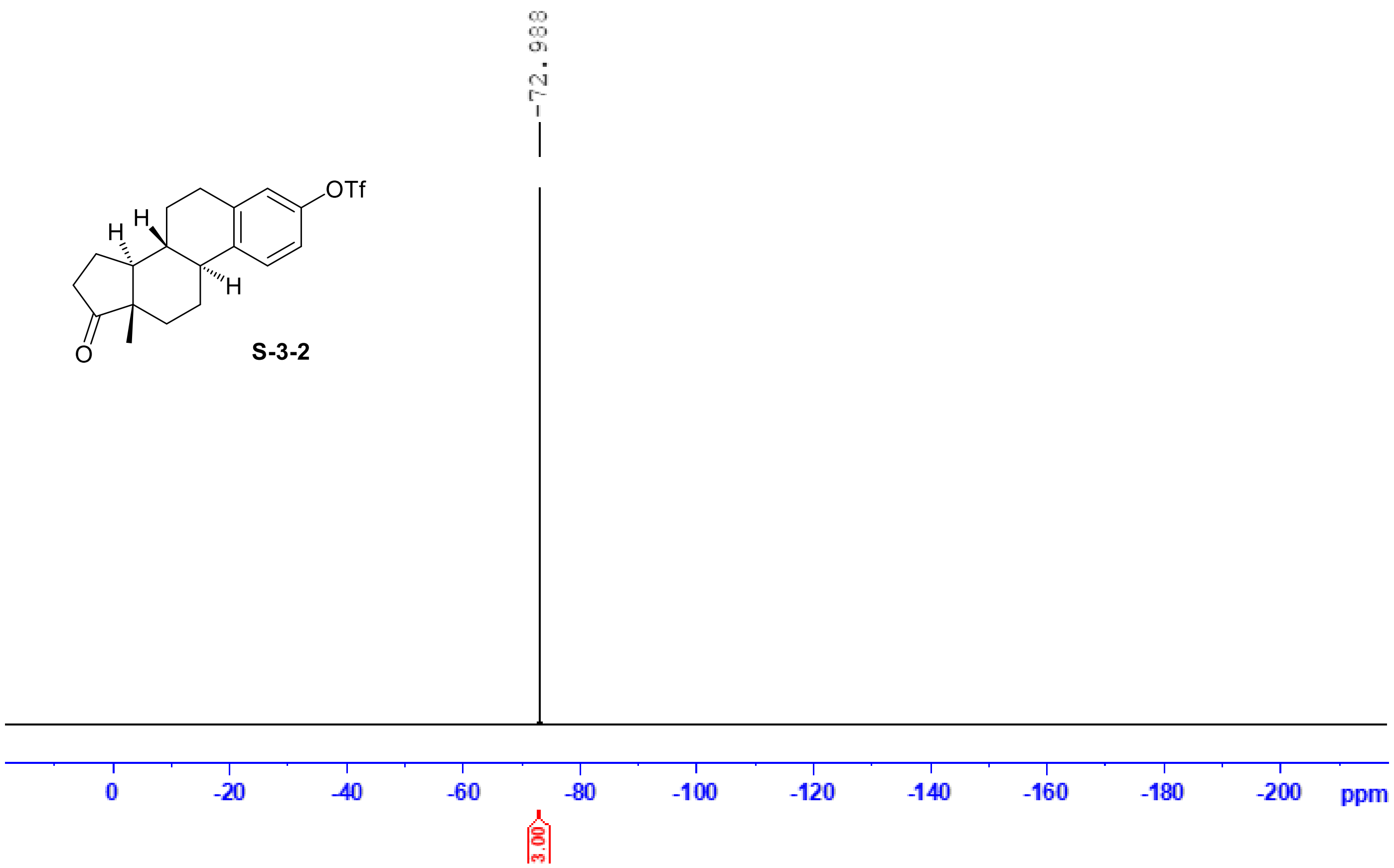

^{1}H NMR of **S-3-3 (Estrone Bpin)**

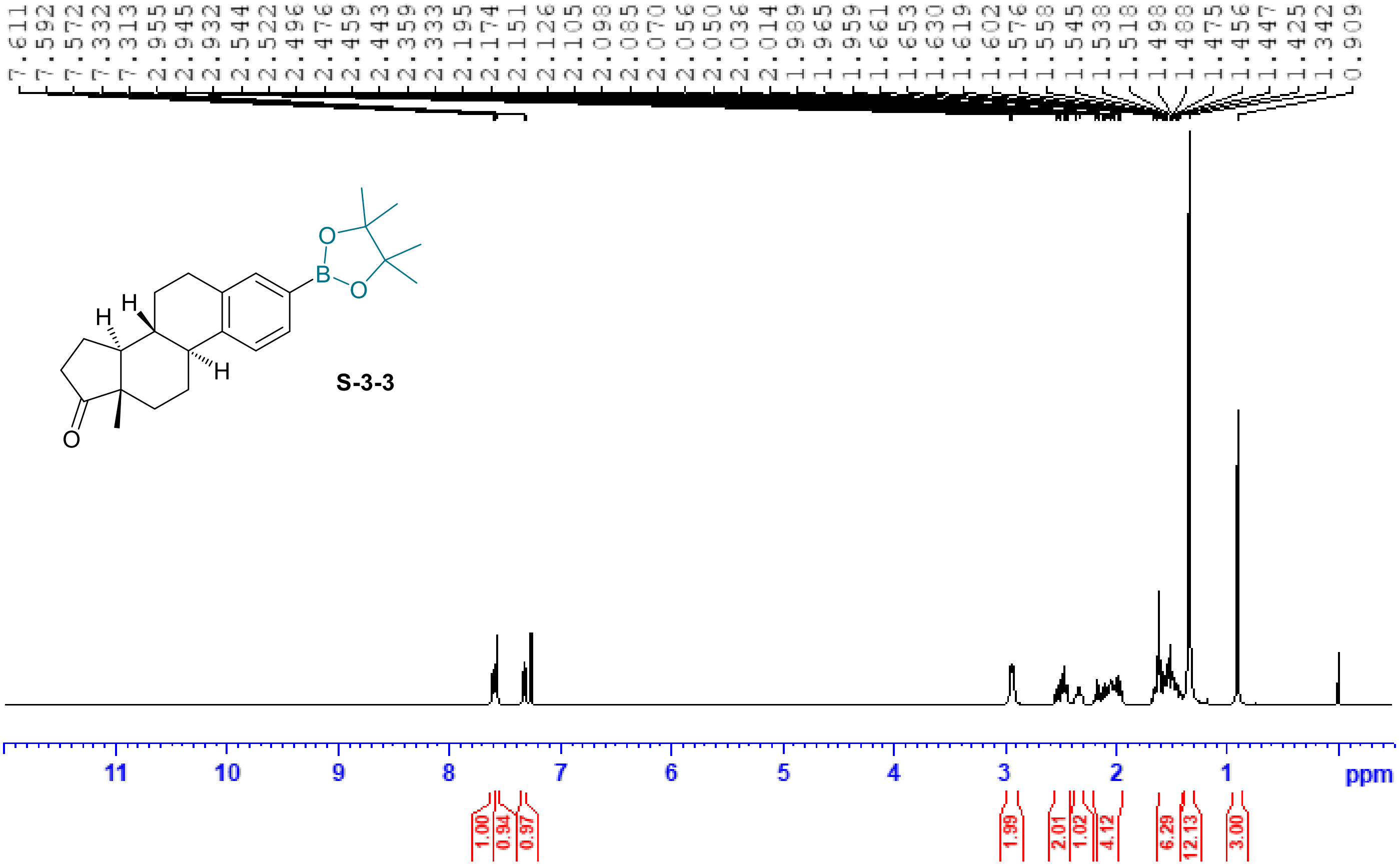

^{13}C NMR of **S-3-3 (Estrone Bpin)**

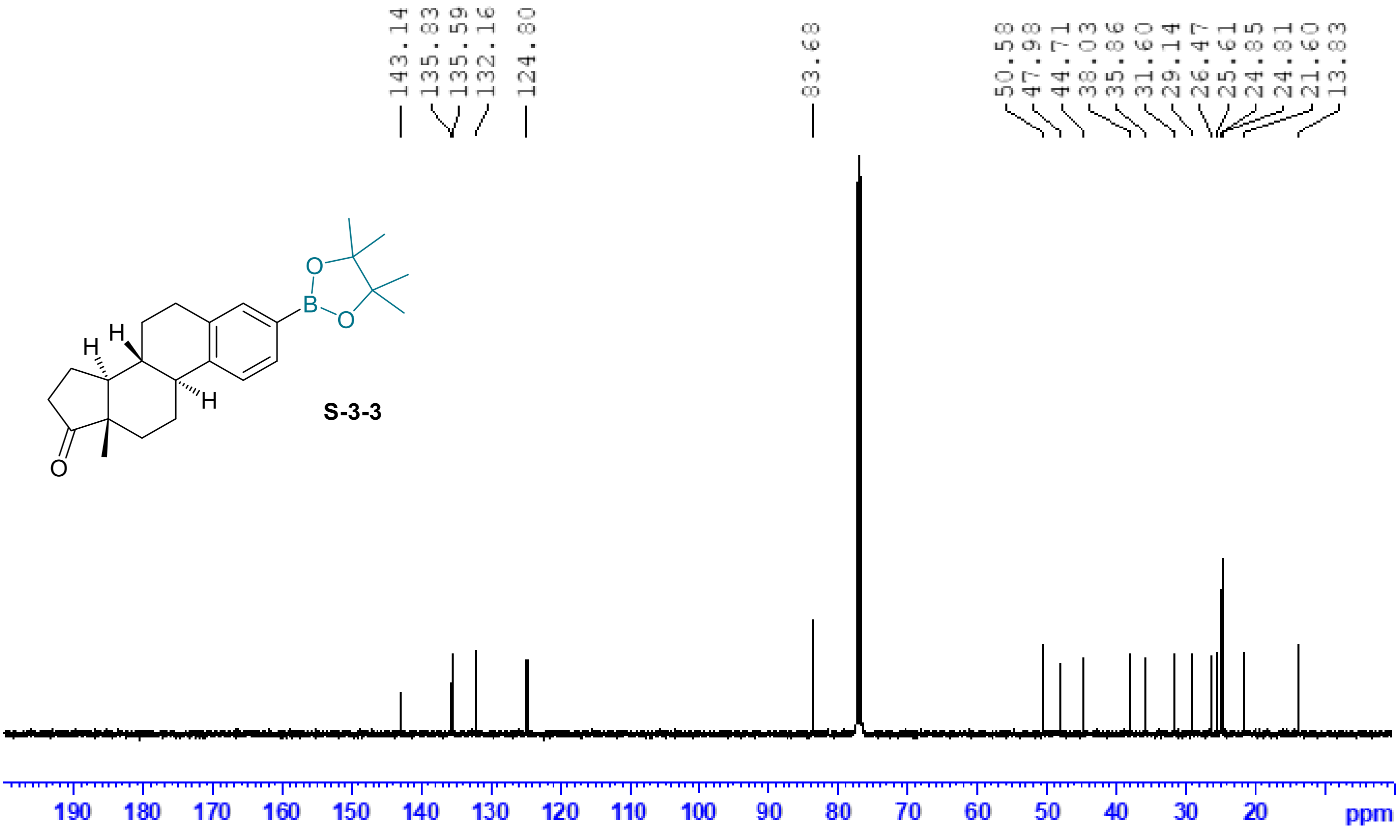

^{1}H NMR of **S-3-4 (Estrone B(OH)$_2$)**

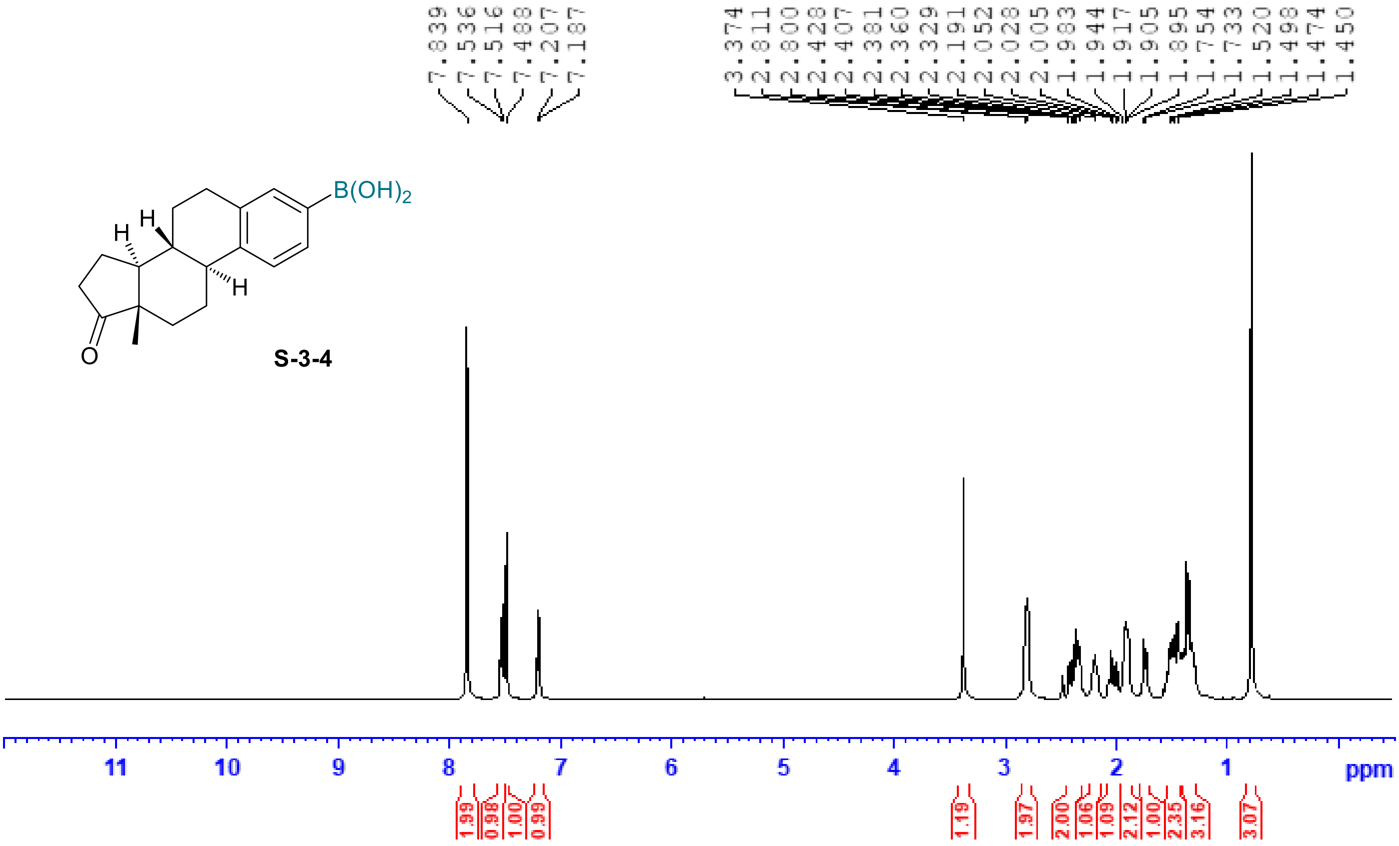

^{13}C NMR of **S-3-4 (Estrone B(OH)$_2$)**

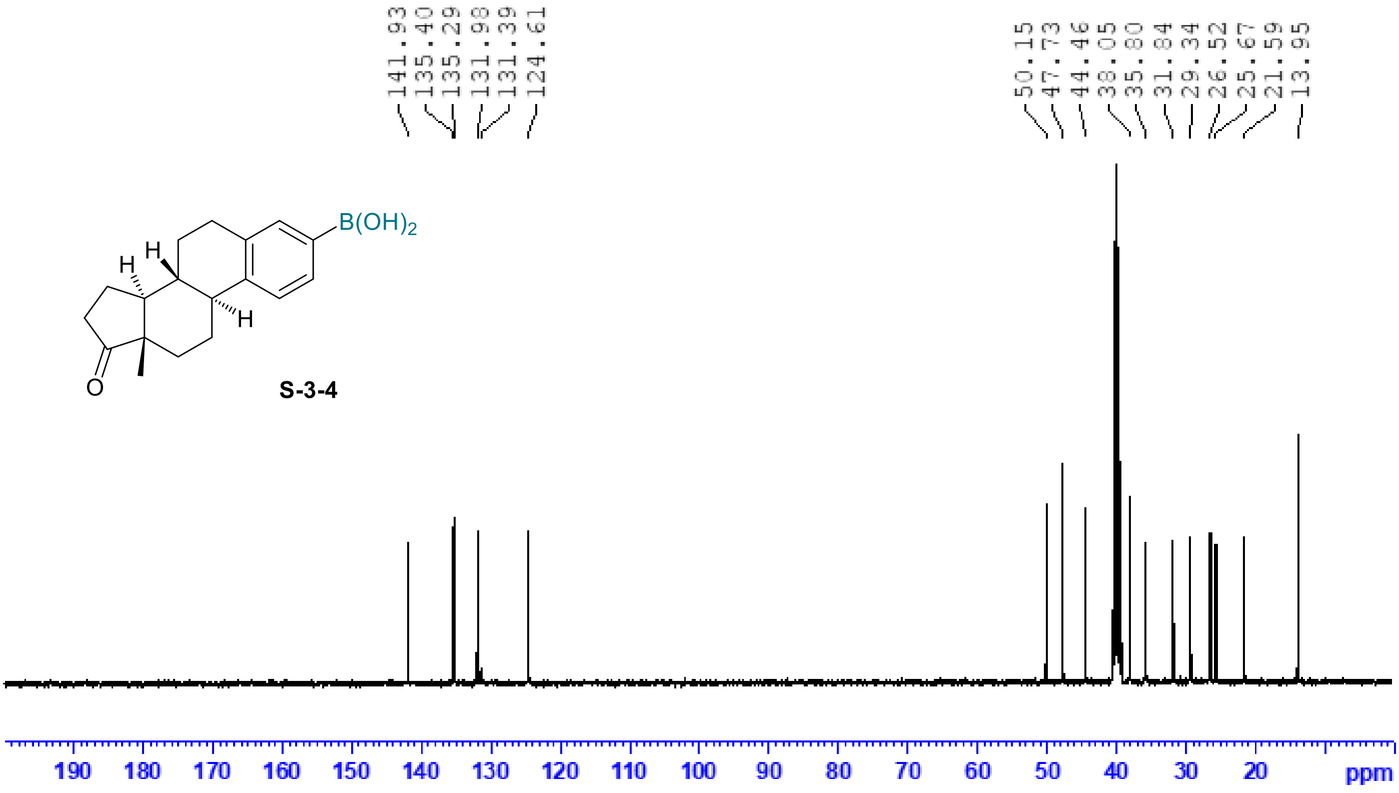

^{1}H NMR of **7v**

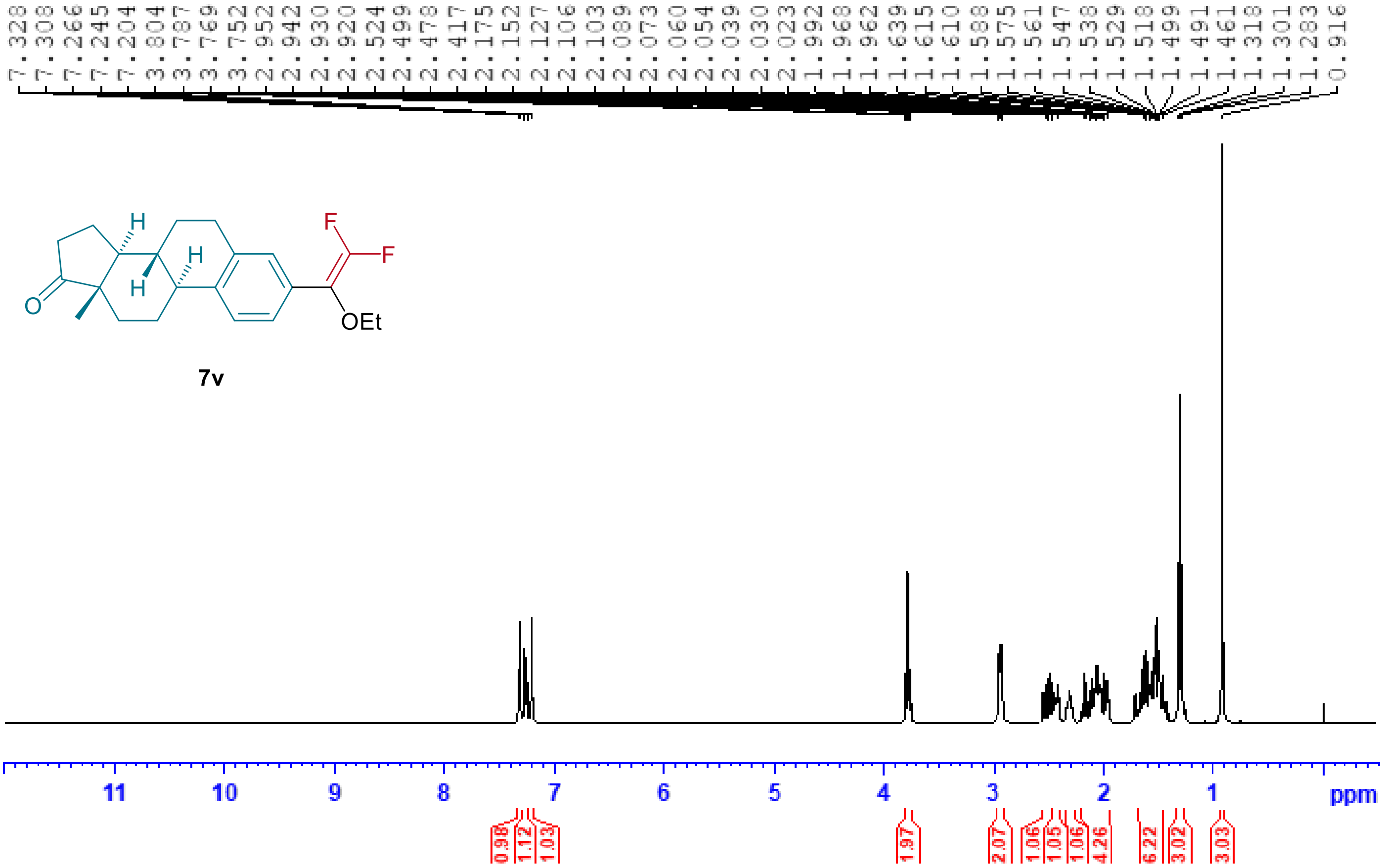

^{13}C NMR of **7v**

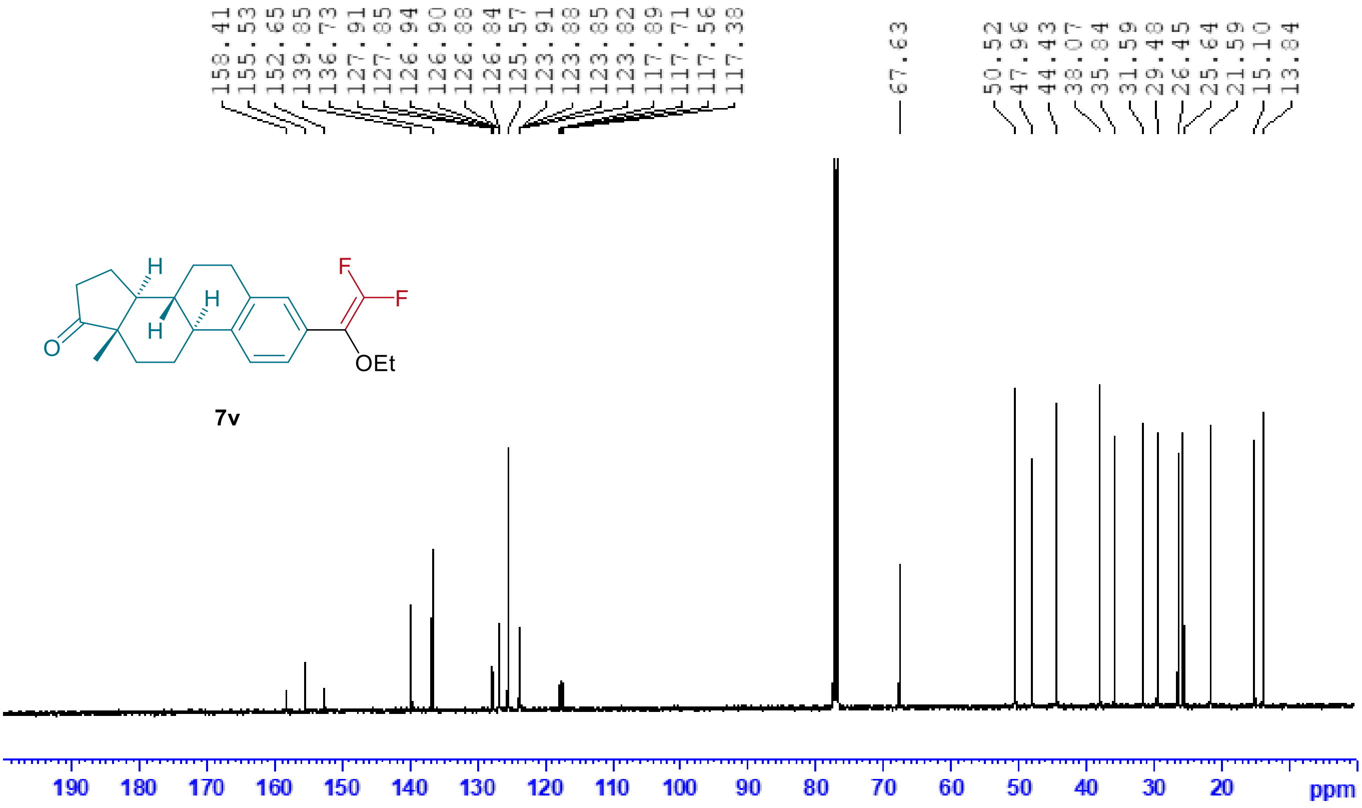

^{19}F NMR of **7v**

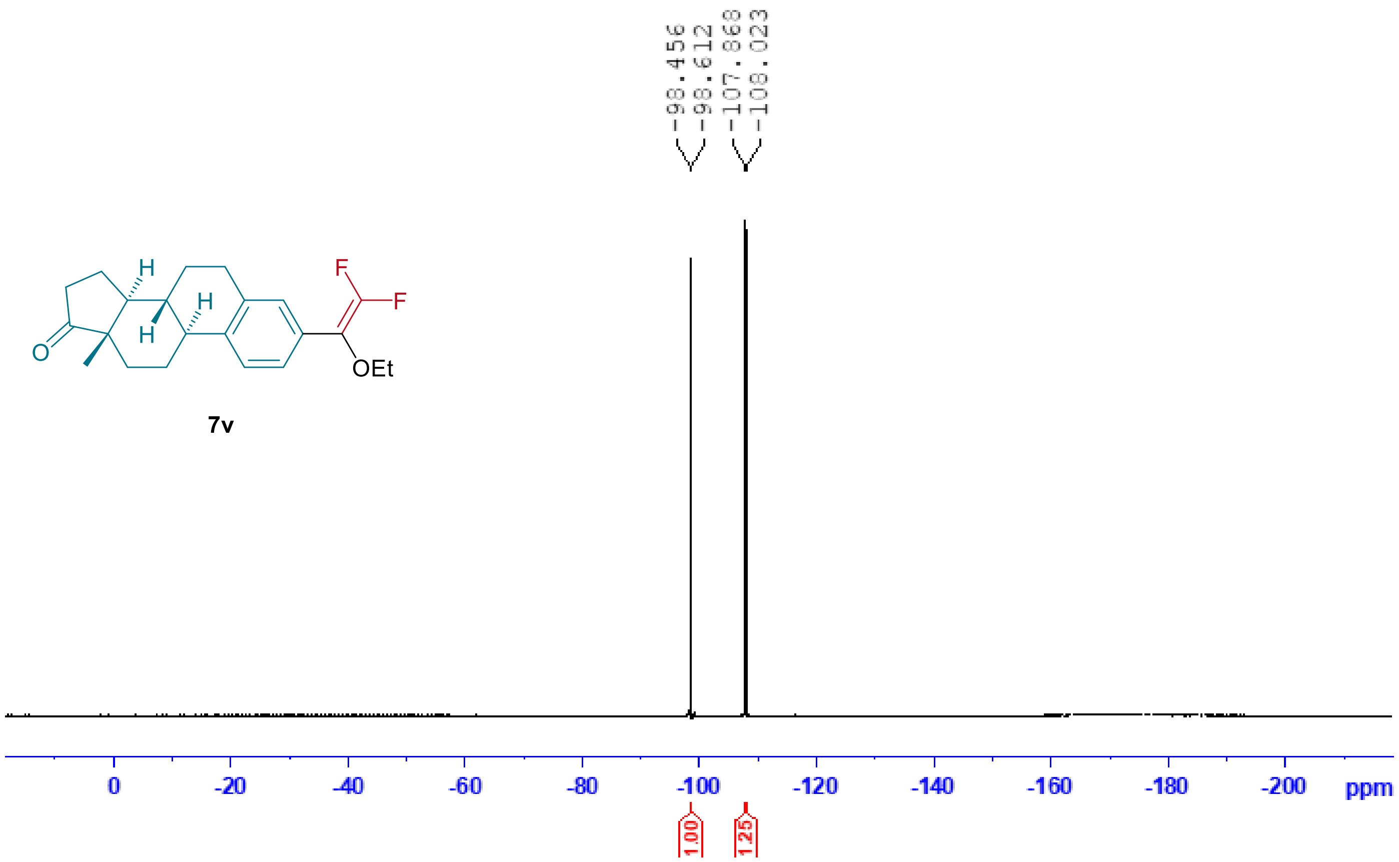

-98.456
-98.612
-107.868
-108.023
H
H
H
O
F
F
OEt
7v
0
-20
-40
-60
-80
-100
-120
-140
-160
-180
-200
ppm
1.00
1.25

^{1}H NMR of **S-2-2 4-(4-Oxochroman-2-yl)phenyl trifluoromethanesulfonate**

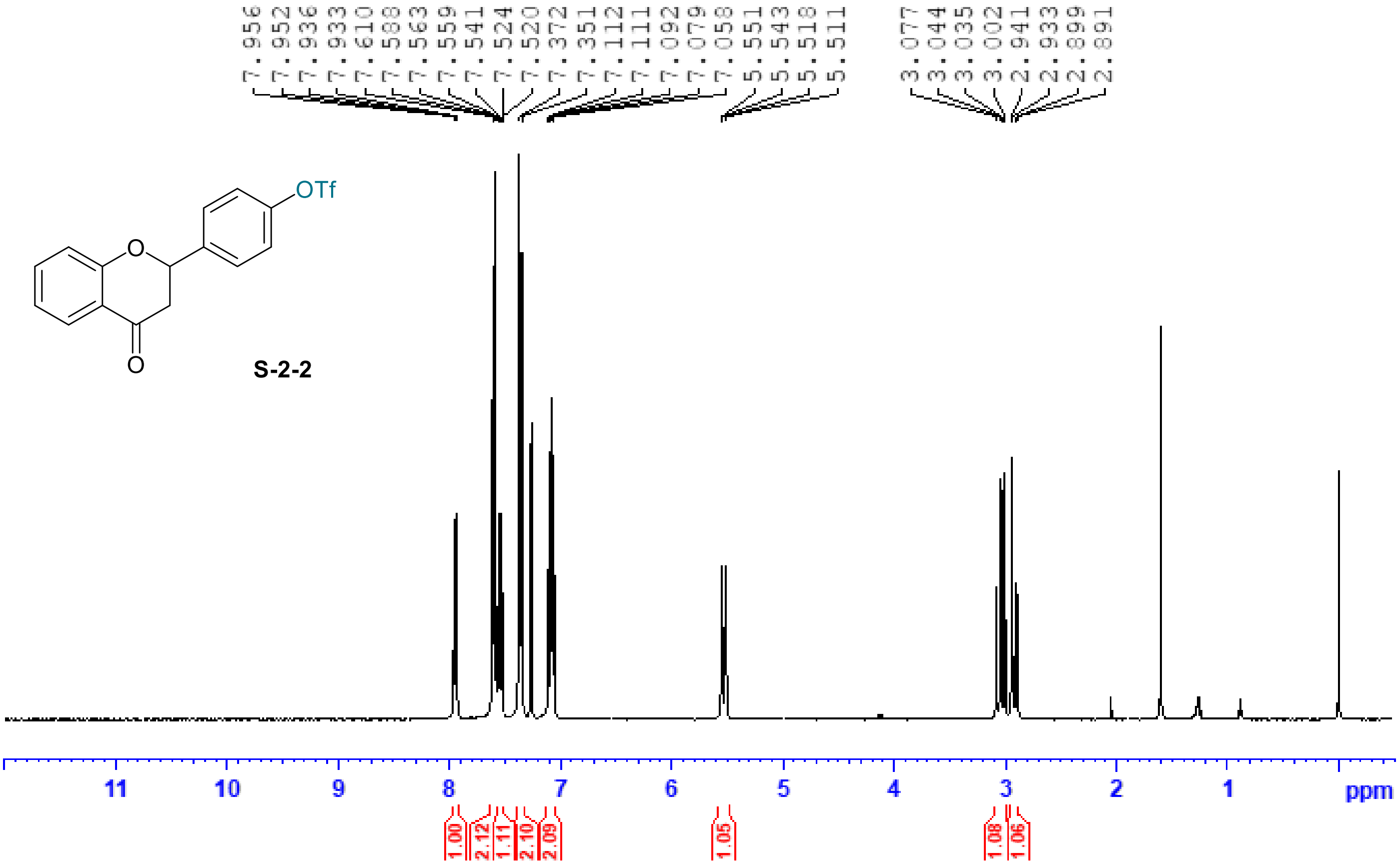

^{13}C NMR of **S-2-2 4-(4-Oxochroman-2-yl)phenyl trifluoromethanesulfonate**

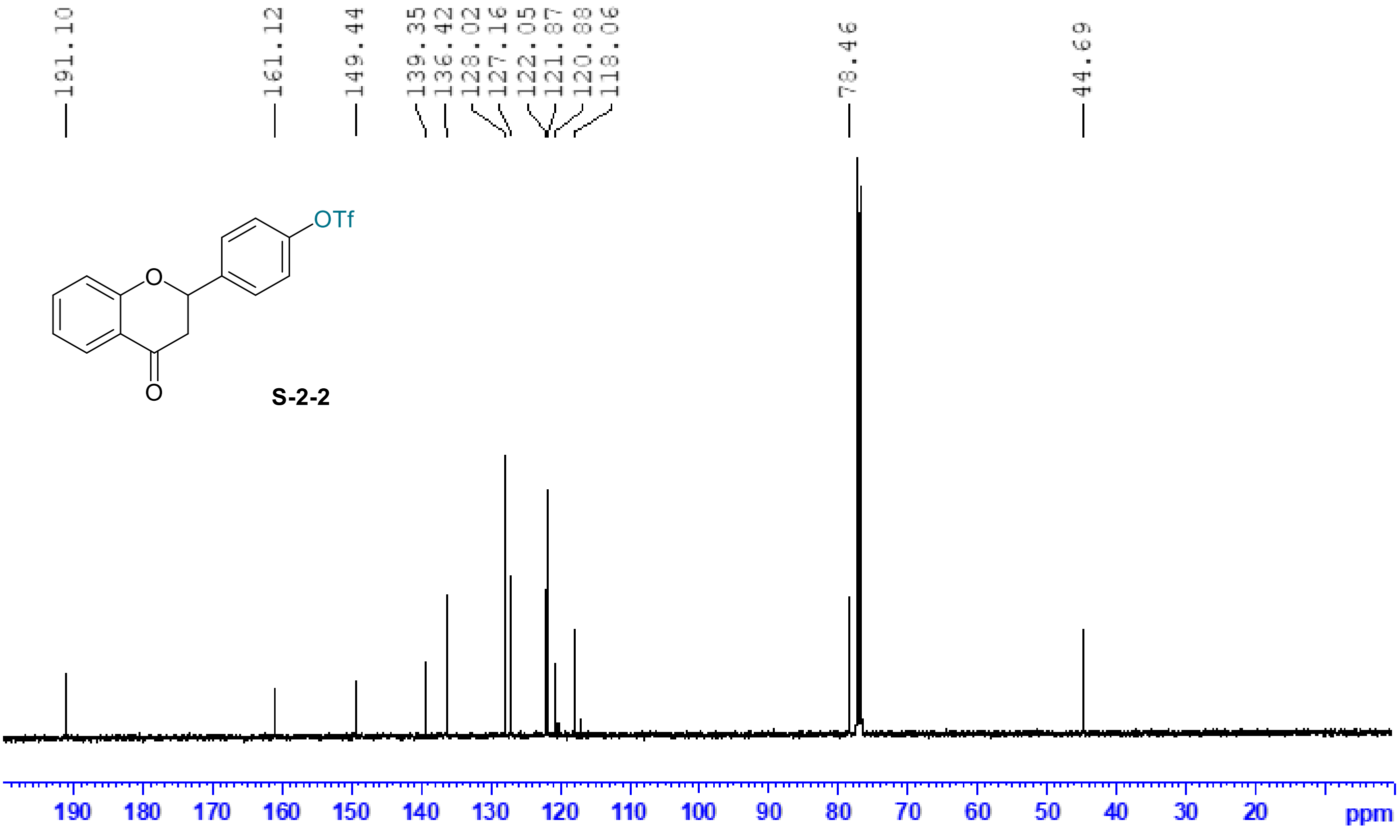

^{19}F NMR of **S-2-2 4-(4-Oxochroman-2-yl)phenyl trifluoromethanesulfonate**

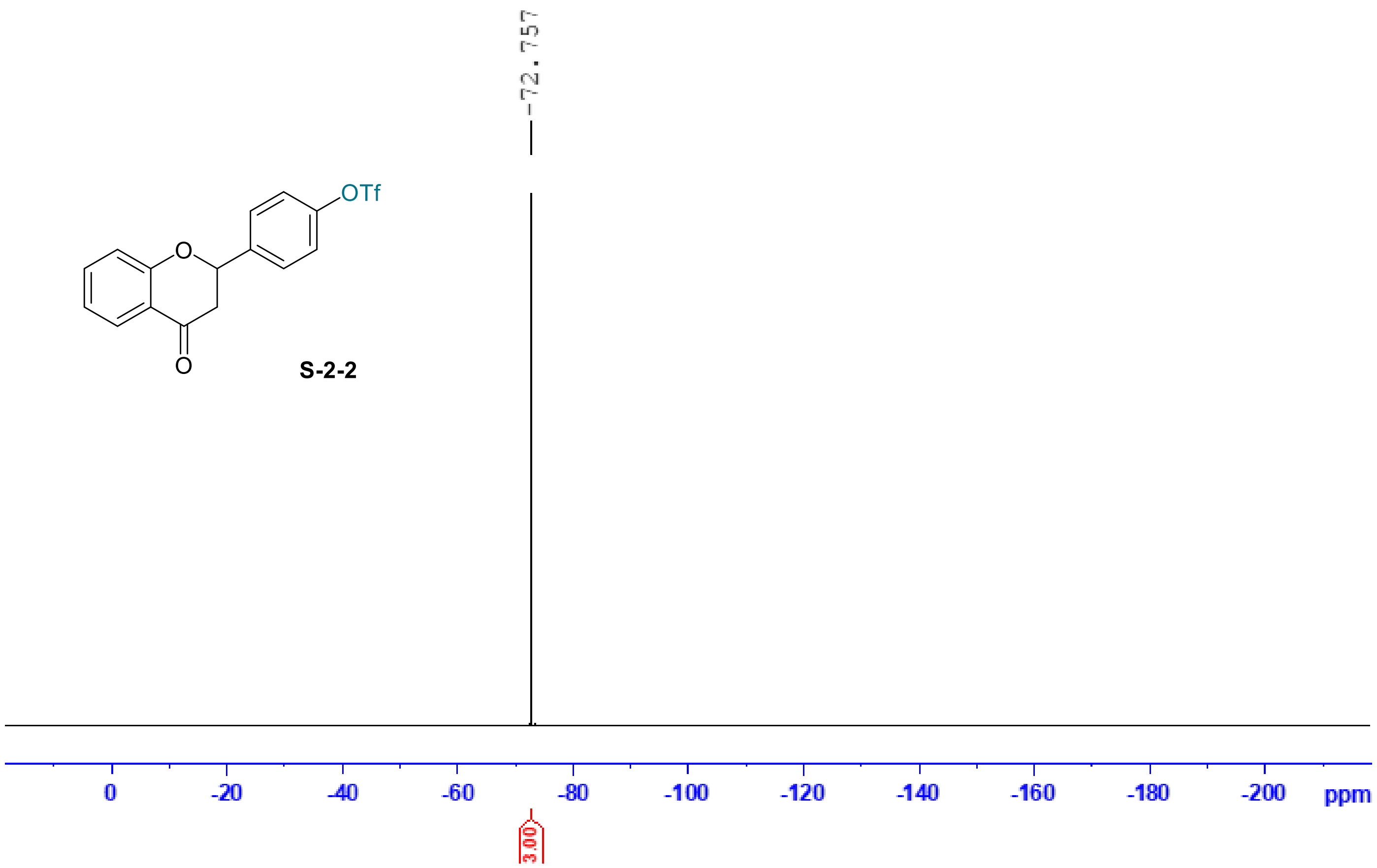

^{1}H NMR of **S-2-3 2-(4-(4,4,5,5-Tetramethyl-1,3,2-dioxaborolan-2-yl)phenyl)chroman-4-one**

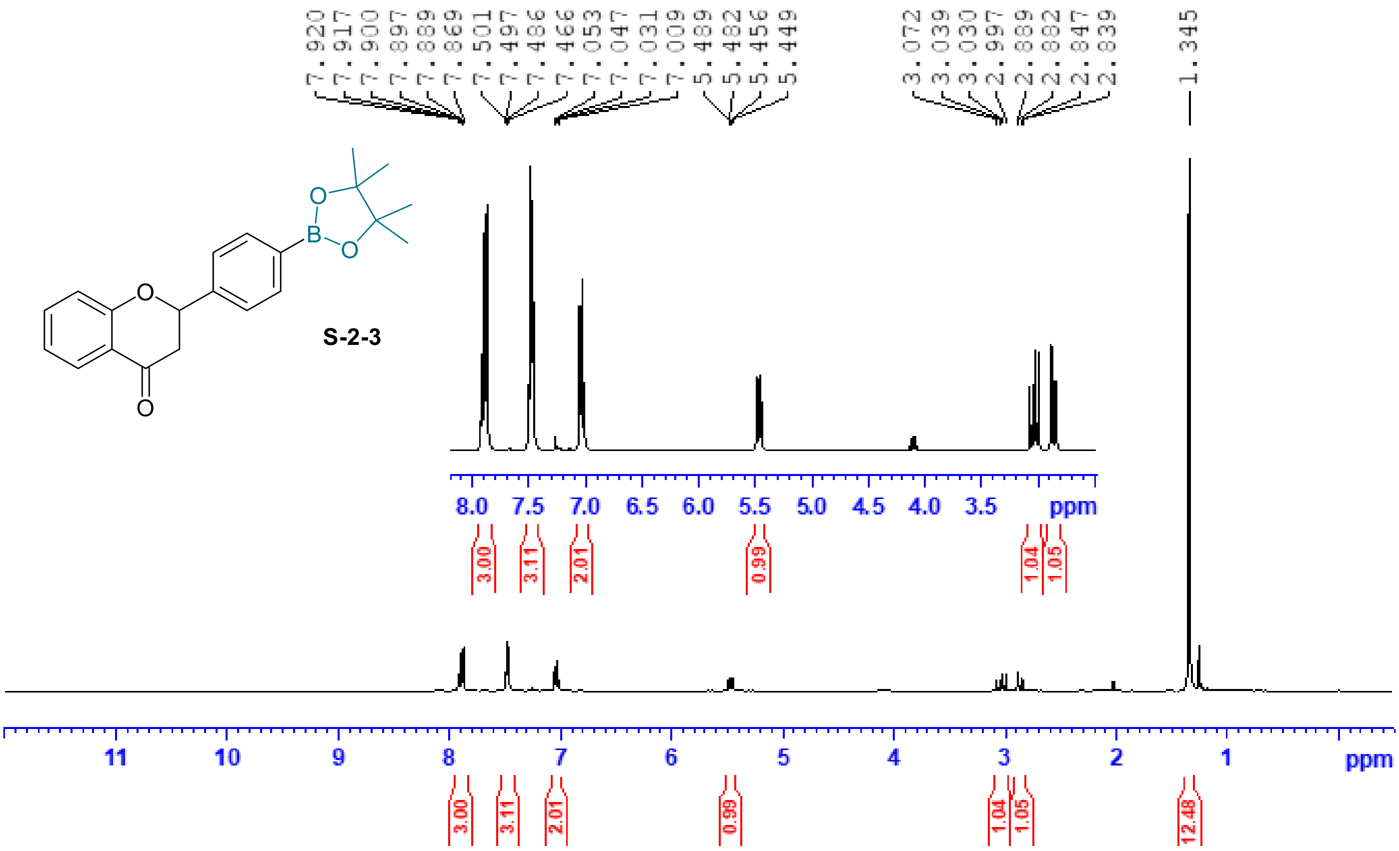

^{13}C NMR of **S-2-3 2-(4-(4,4,5,5-Tetramethyl-1,3,2-dioxaborolan-2-yl)phenyl)chroman-4-one**

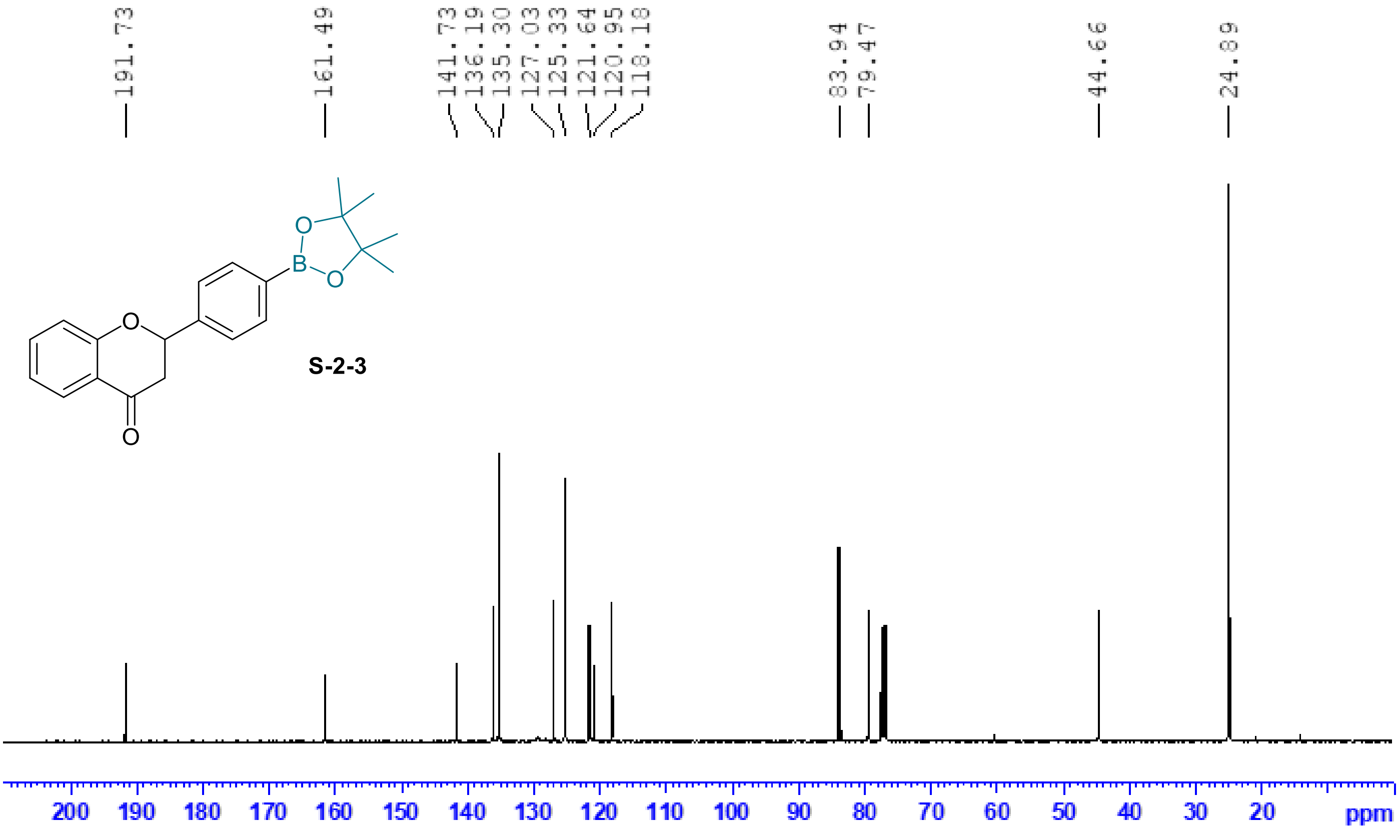

^{1}H NMR of **S-2-4 (4-(4-Oxochroman-2-yl)phenyl)boronic acid**

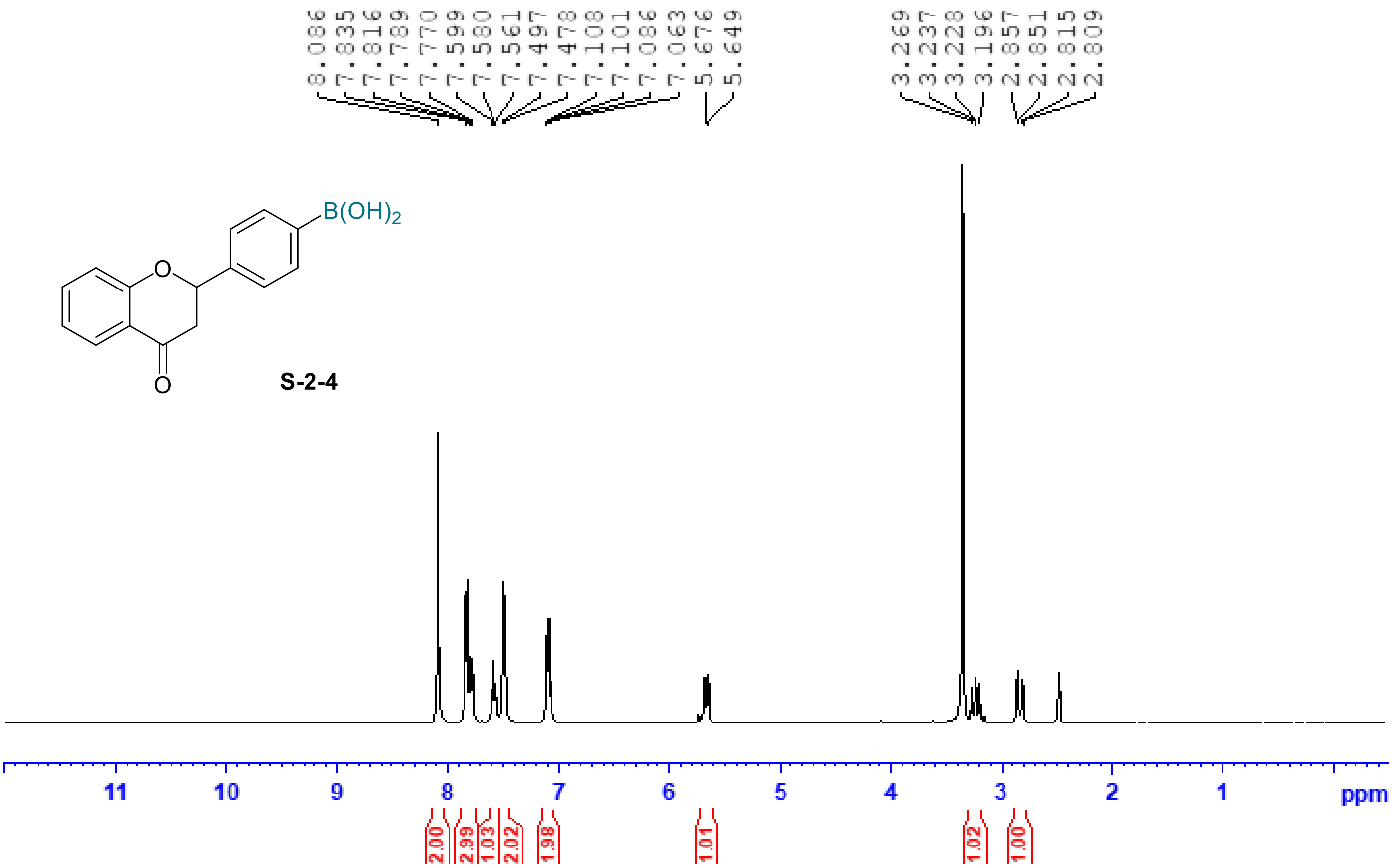

^{13}C NMR of **S-2-4 (4-(4-Oxochroman-2-yl)phenyl)boronic acid**

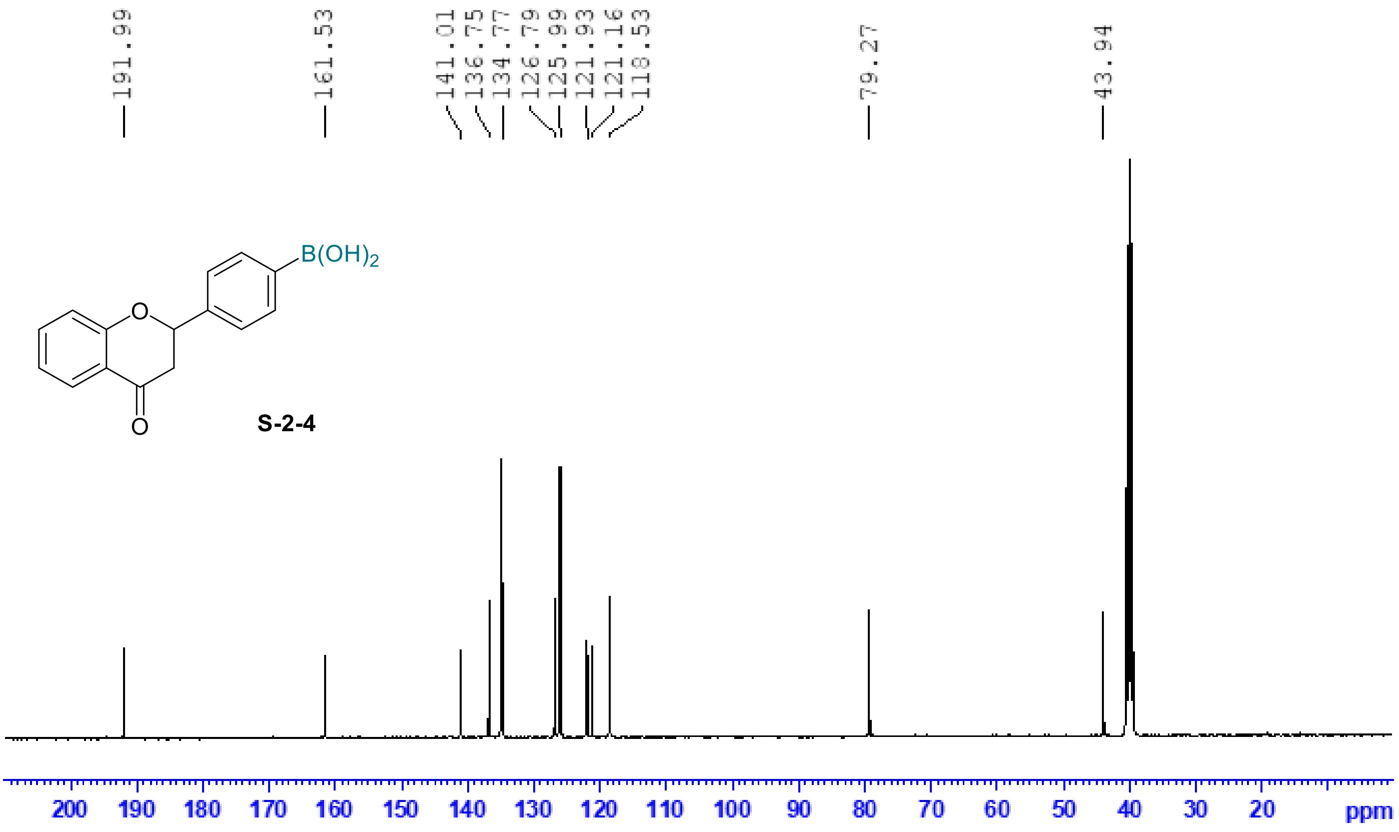

^{1}H NMR of **7w**

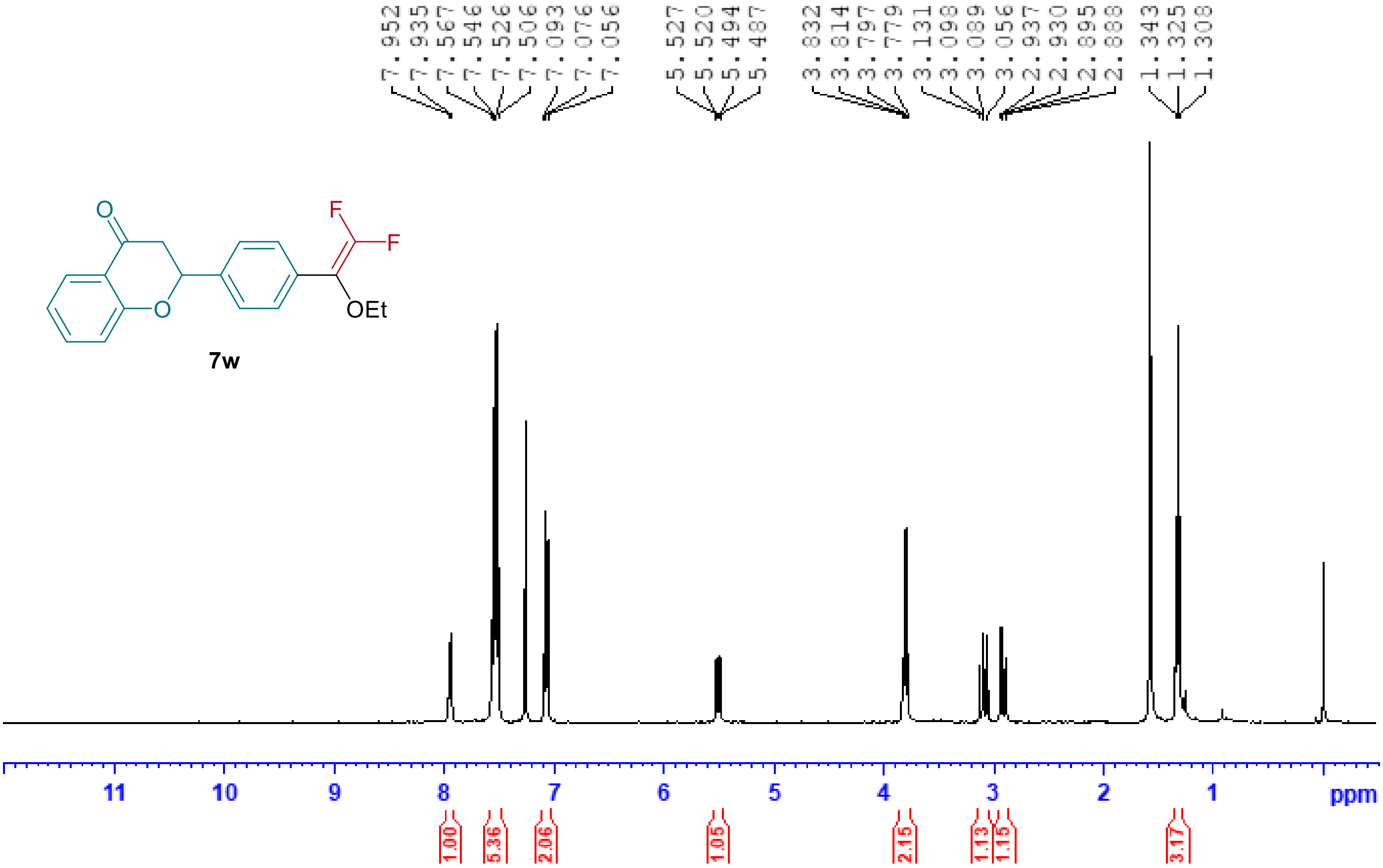

^{13}C NMR of **7w**

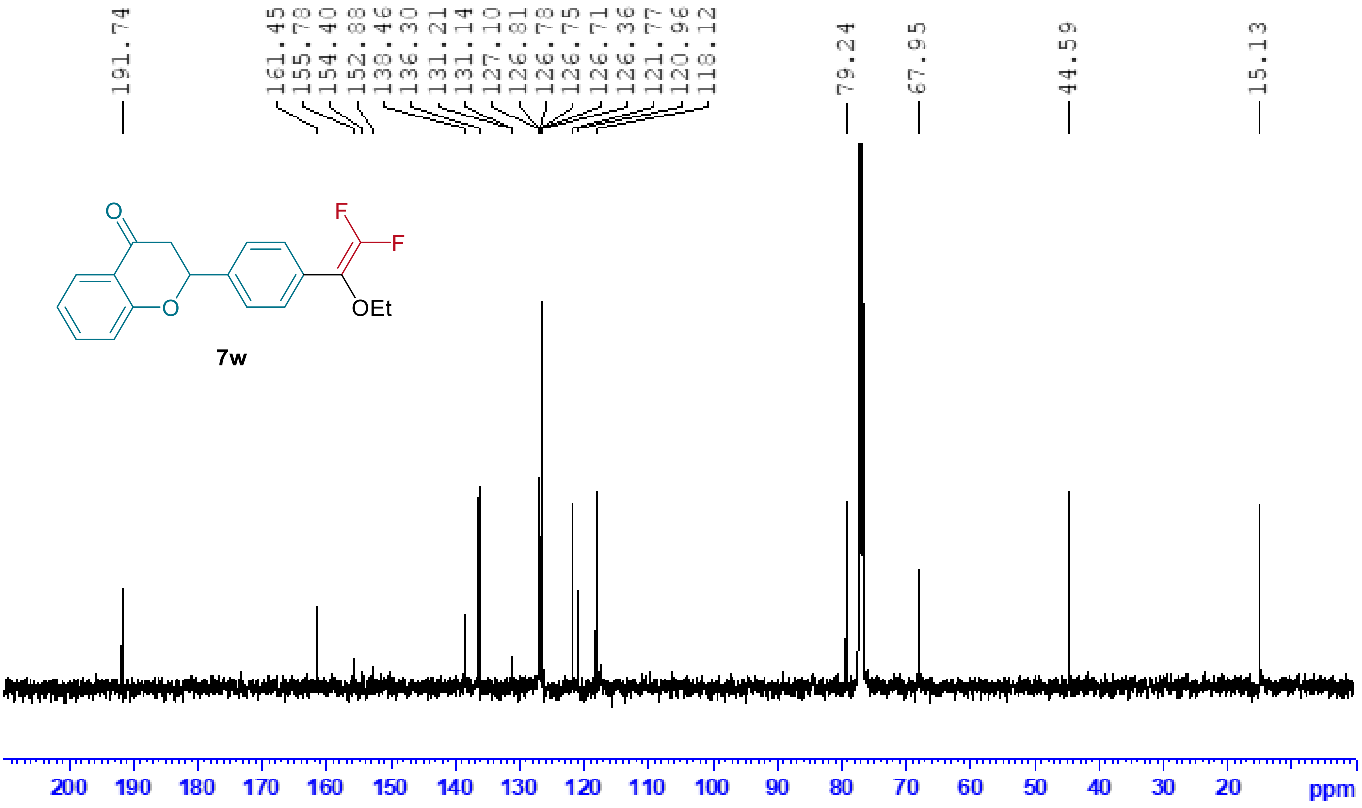

^{19}F NMR of **7w**

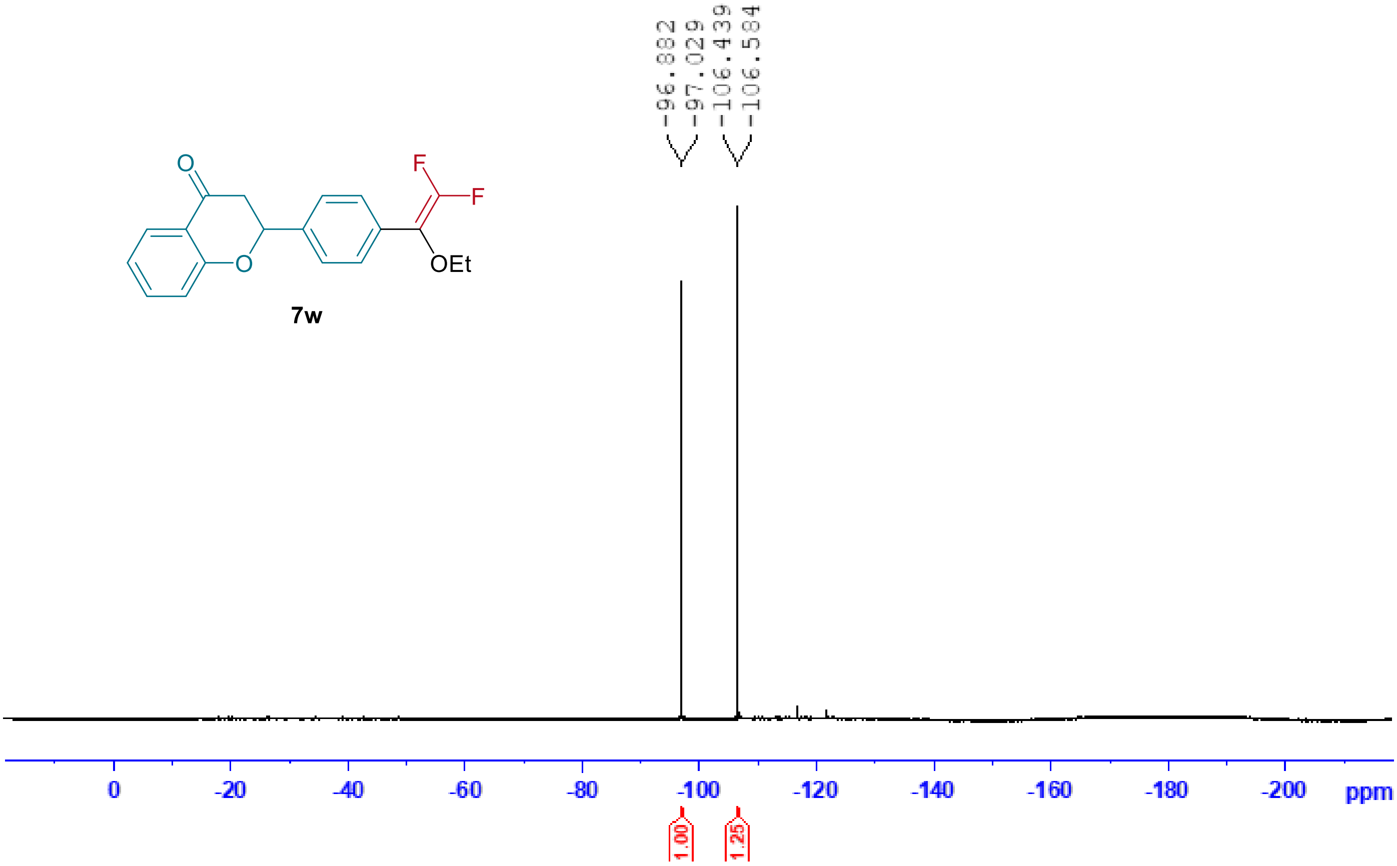

O
F
F
O
OEt
7w
-96.882
-97.029
-106.439
-106.584
0
-20
-40
-60
-80
-100
-120
-140
-160
-180
-200
ppm
1.00
1.25

^{1}H NMR of **8a**

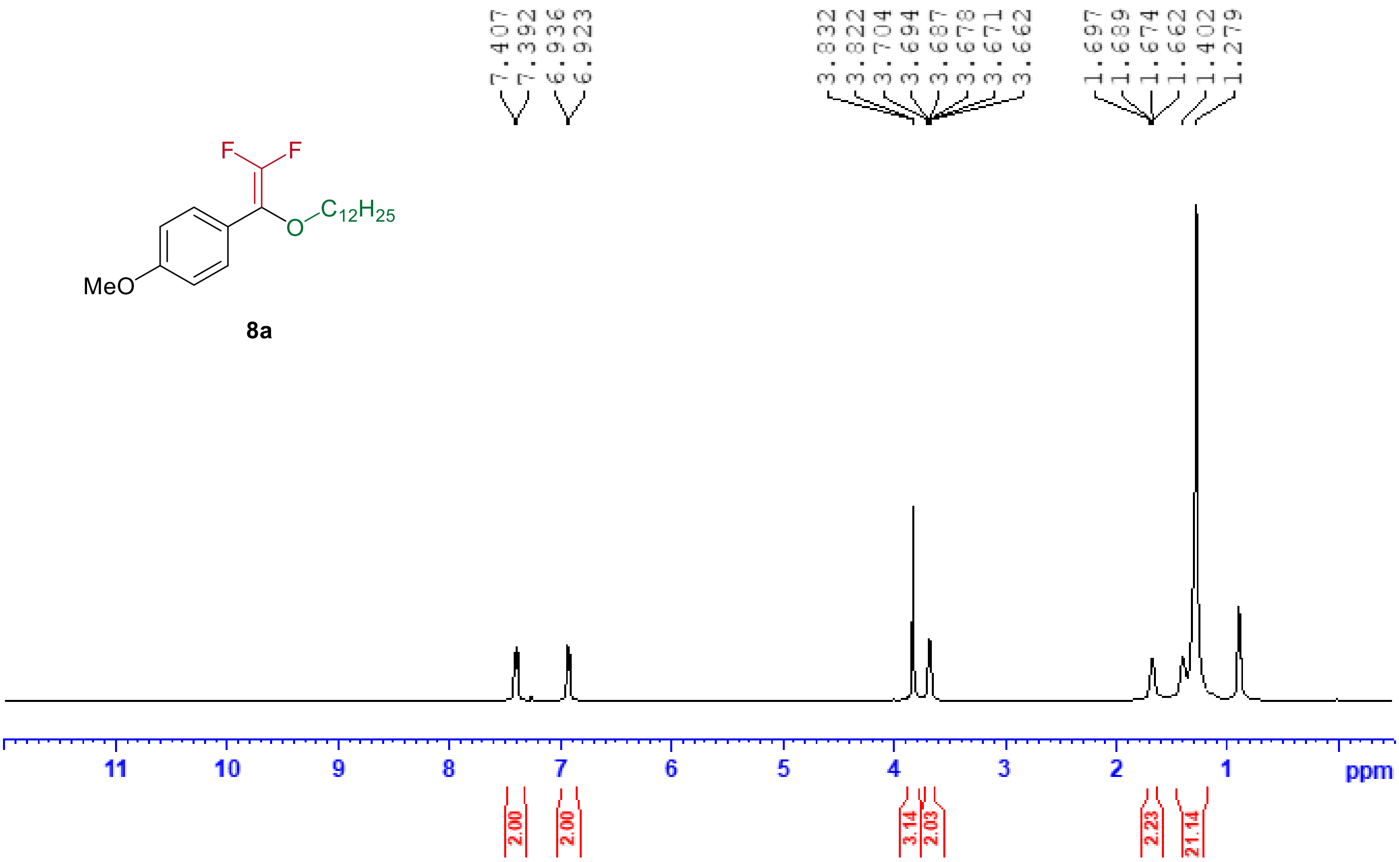

^{13}C NMR of **8a**

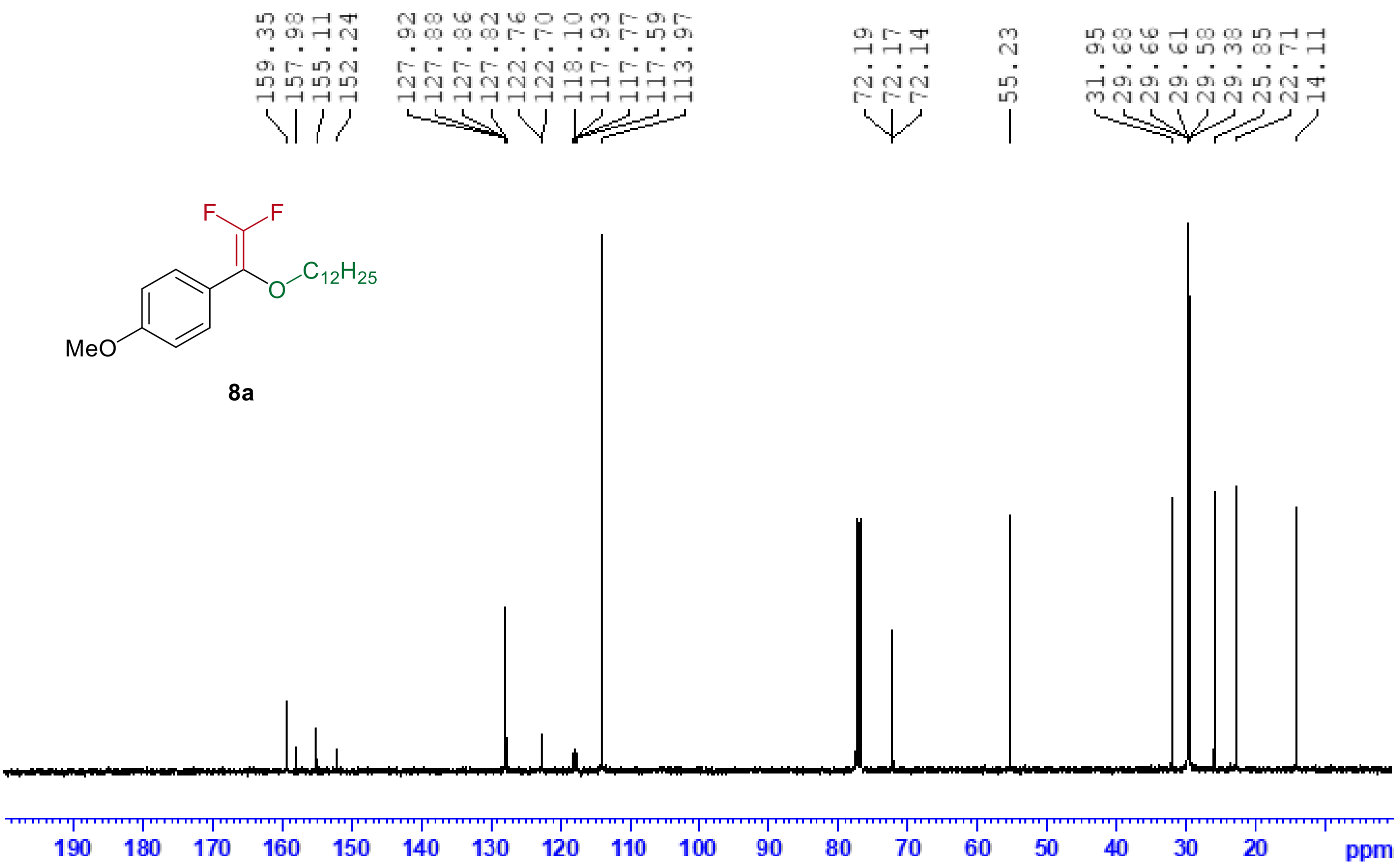

^{19}F NMR of **8a**

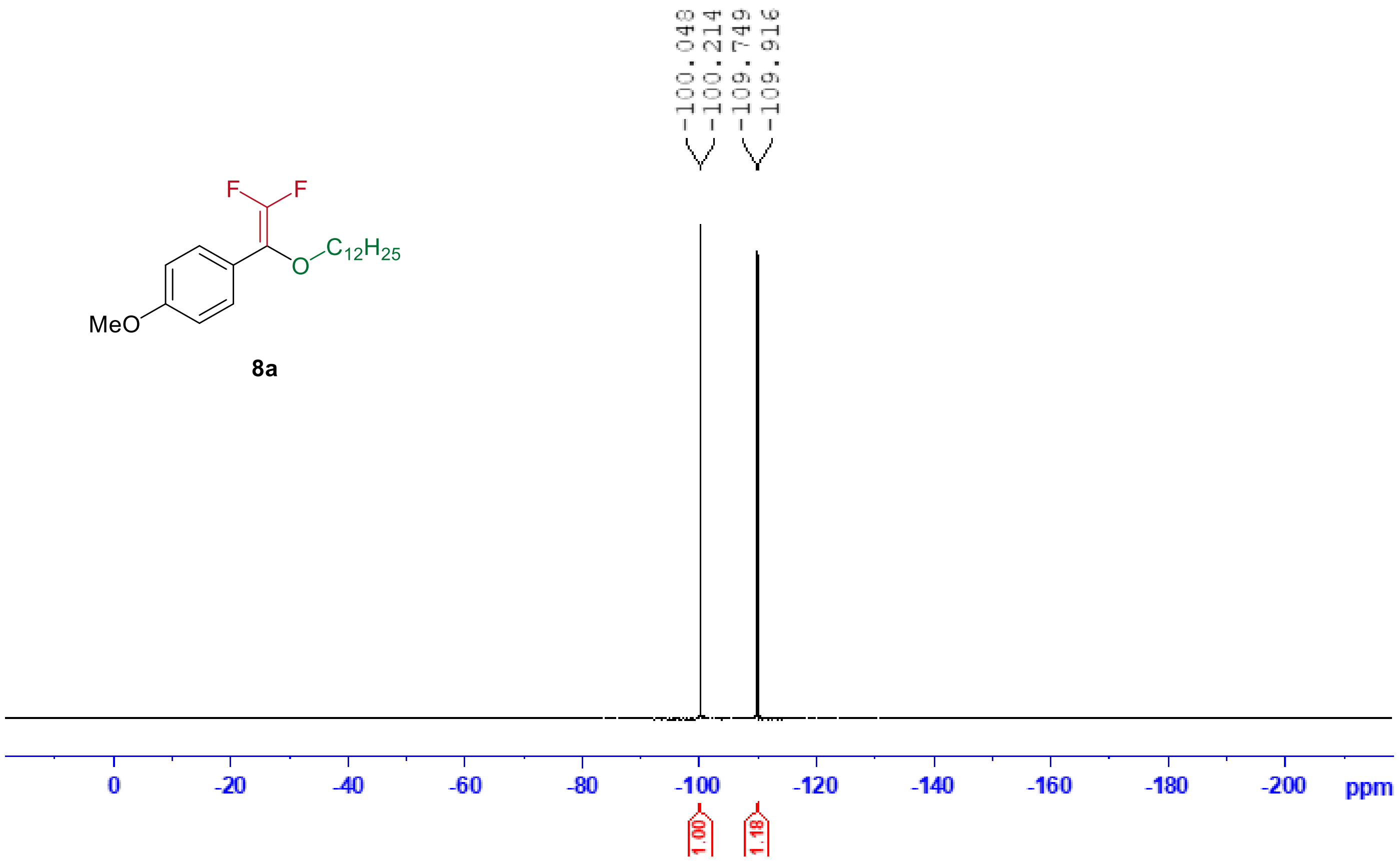

-100.048
-100.214
-109.749
-109.916
F
F
O
C12H25
MeO
8a
0
-20
-40
-60
-80
-100
-120
-140
-160
-180
-200
ppm
1.00
1.18

^{1}H NMR of **8b**

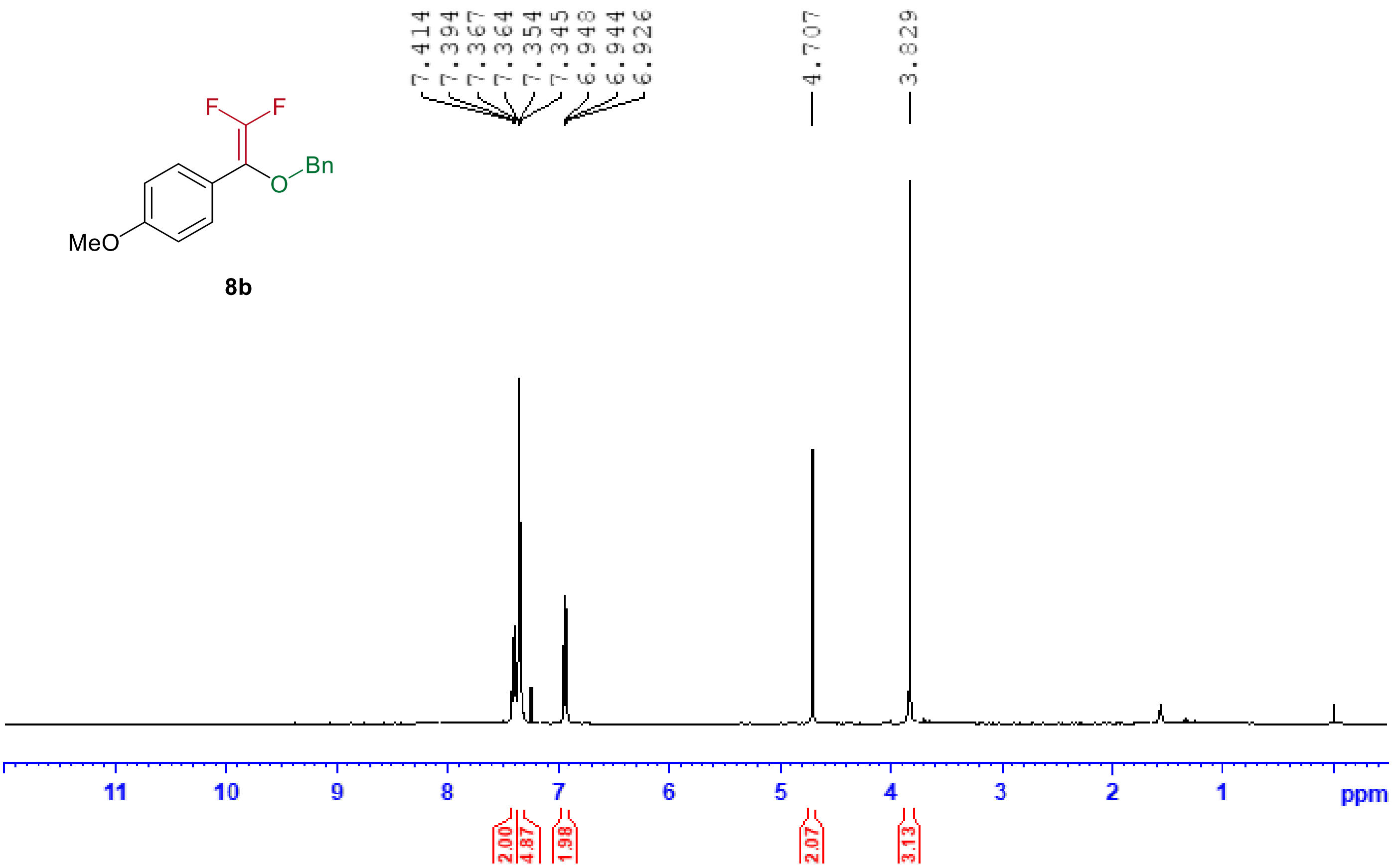

^{13}C NMR of **8b**

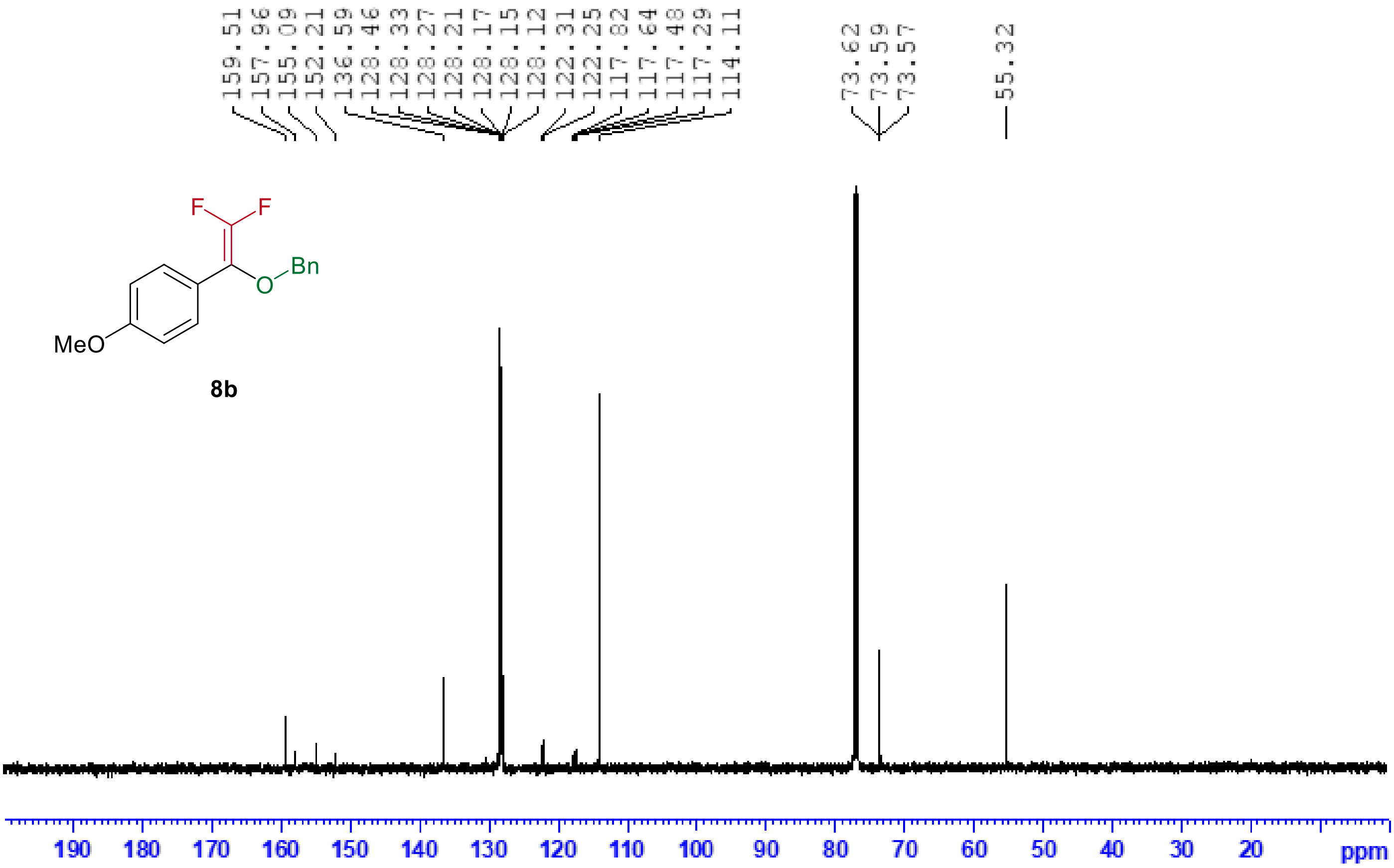

^{19}F NMR of **8b**

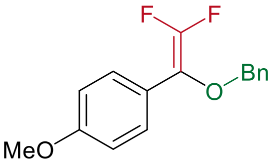

8b

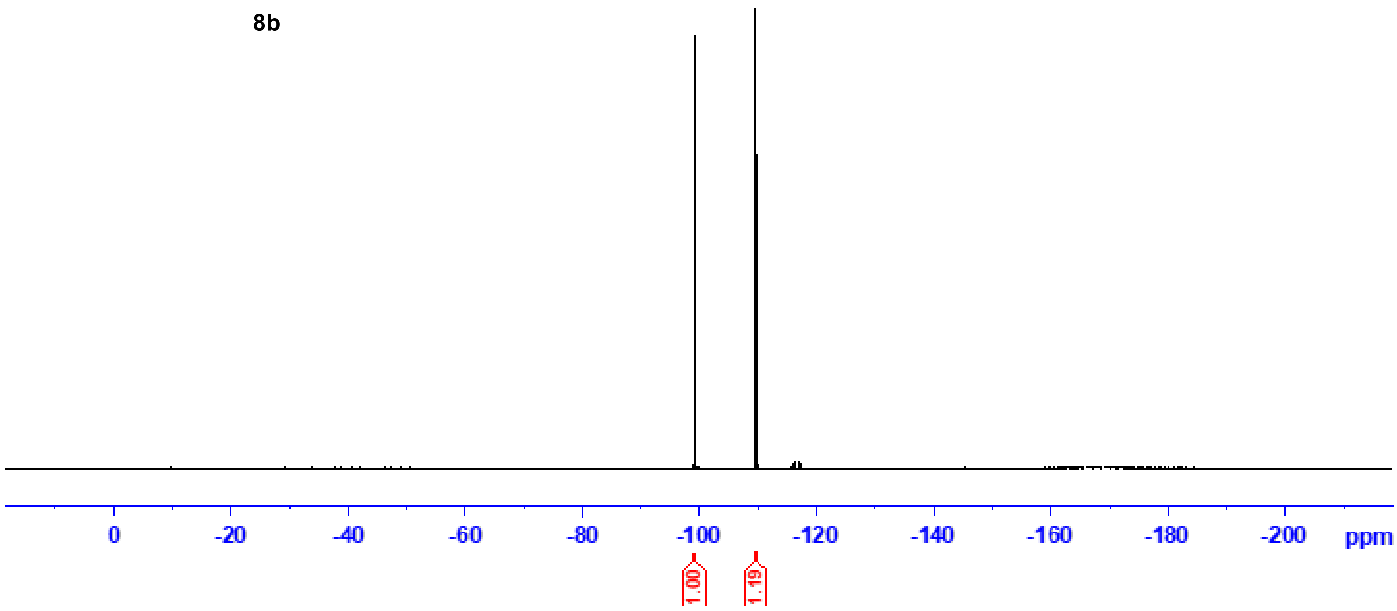

^{1}H NMR of **8c**

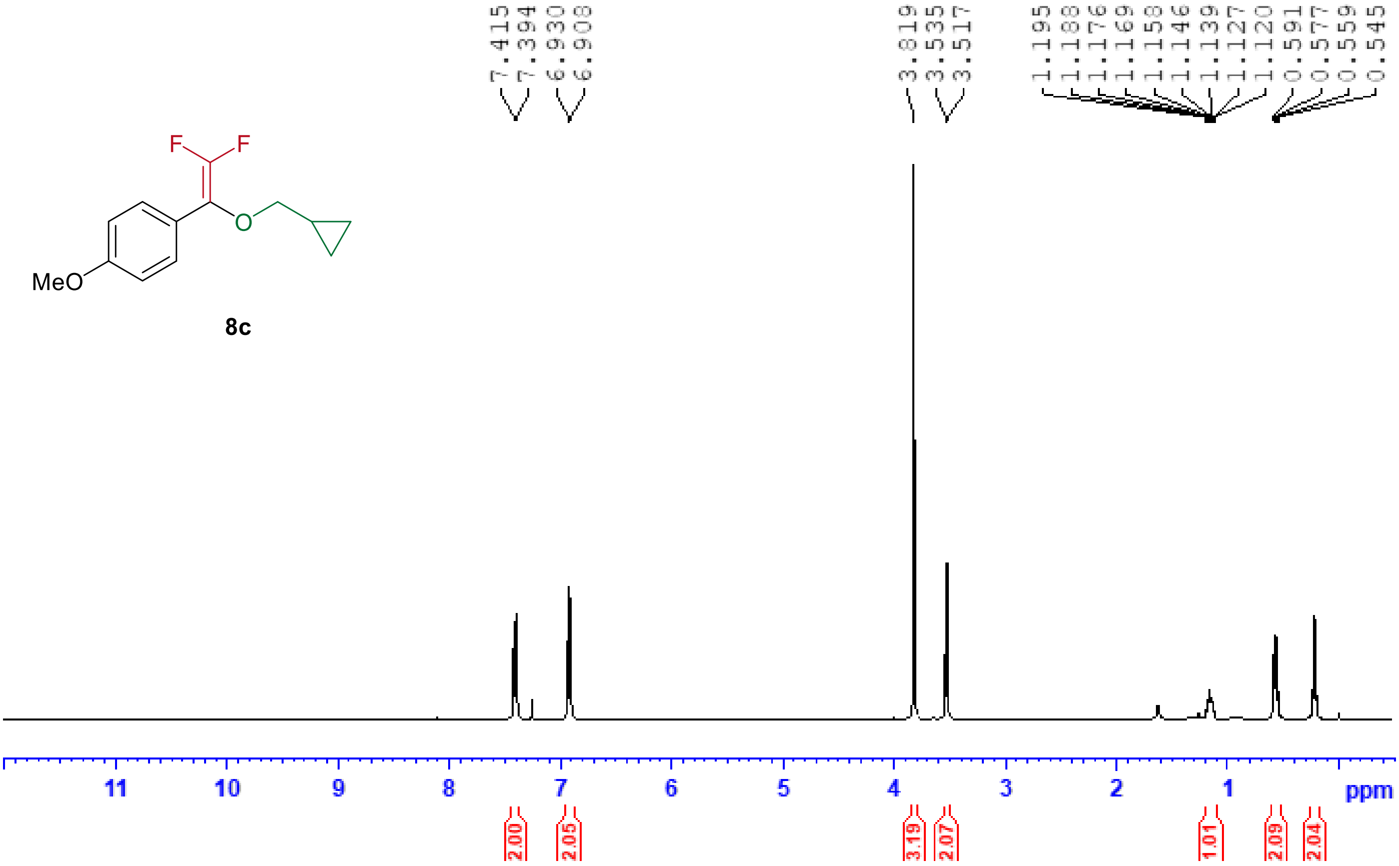

^{13}C NMR of **8c**

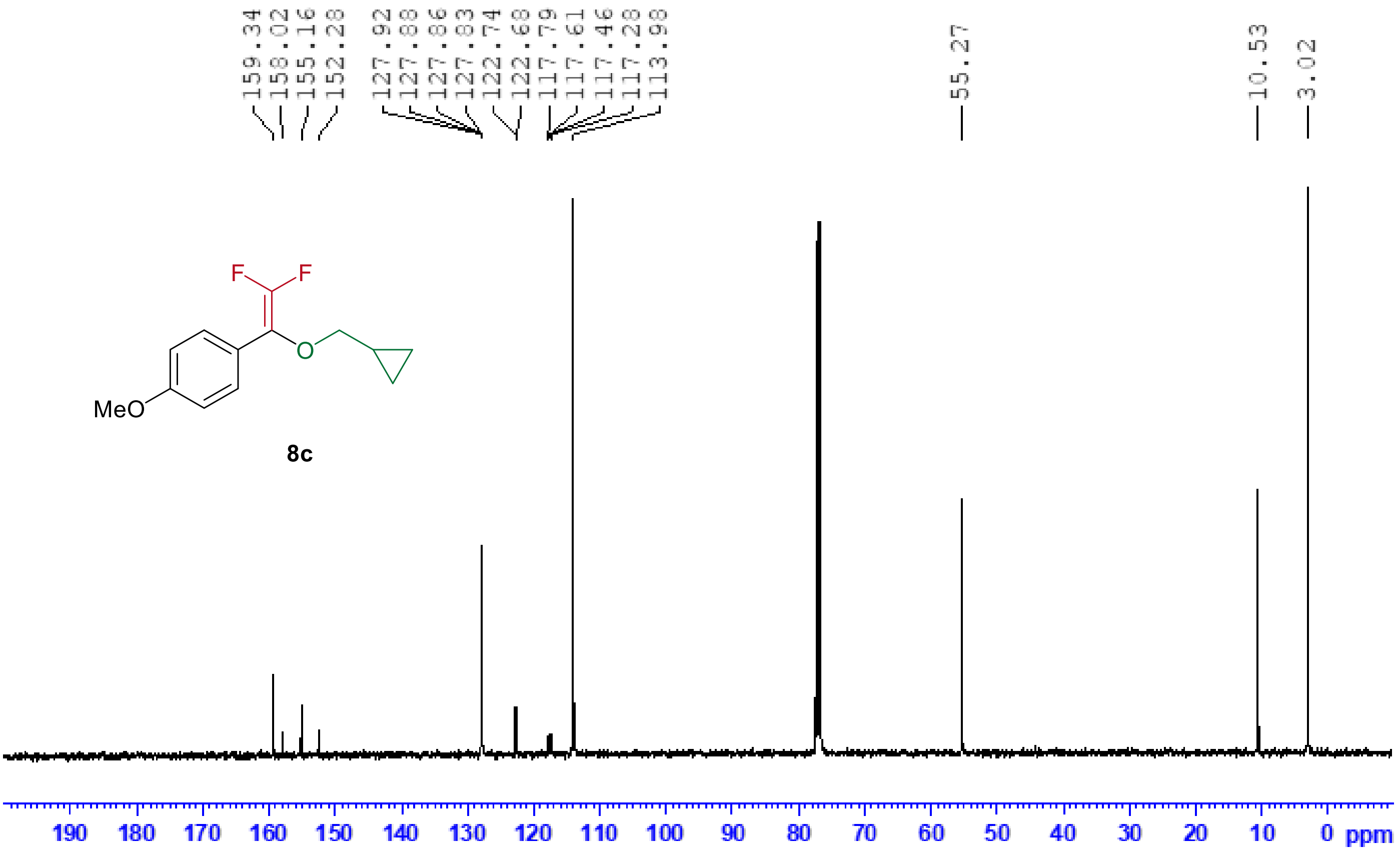

^{19}F NMR of **8c**

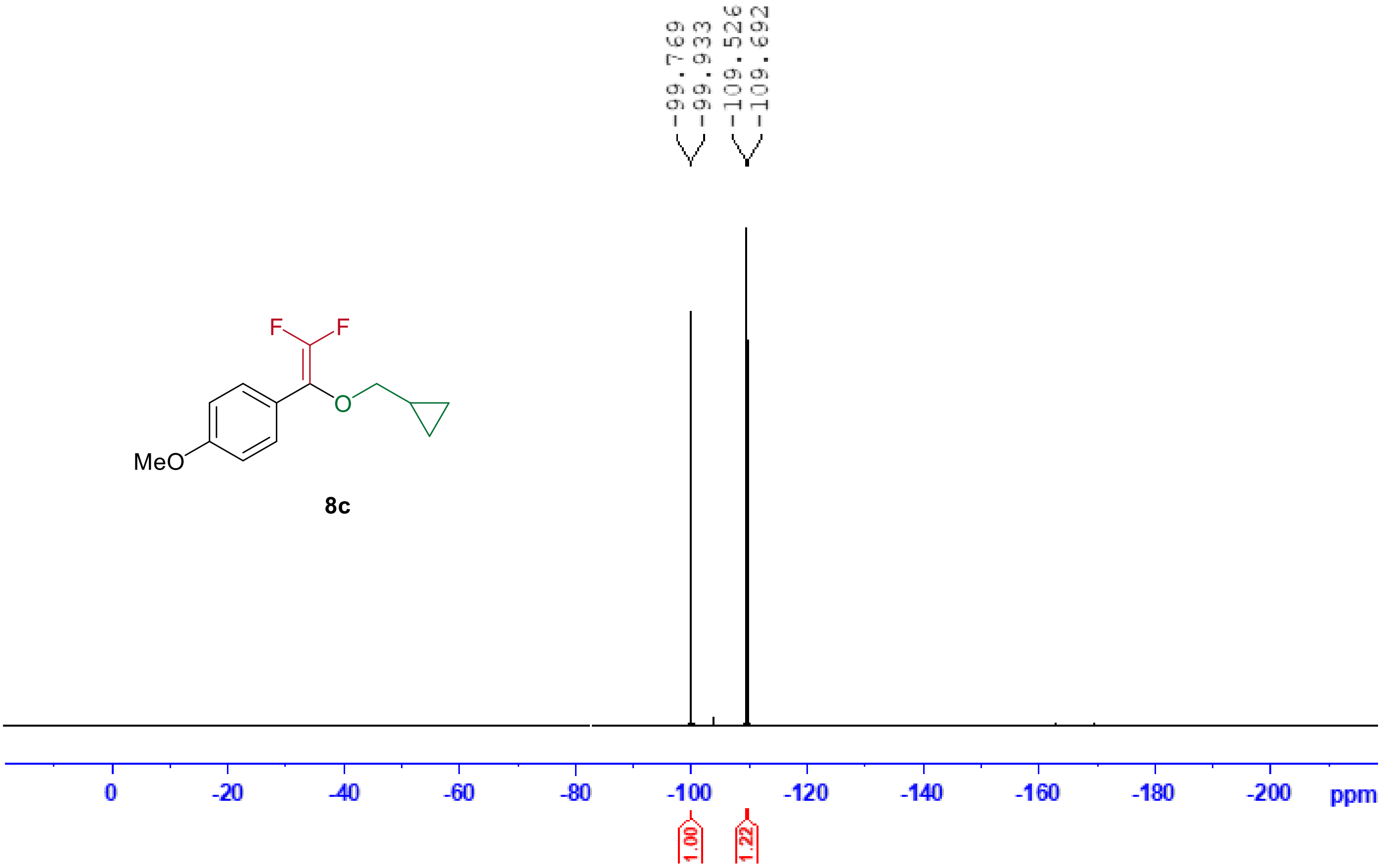
-99.769
-99.933
-109.526
-109.692
F
F
O
MeO
8c
0
-20
-40
-60
-80
-100
-120
-140
-160
-180
-200
ppm
1.00
1.22

^{1}H NMR of **8d**

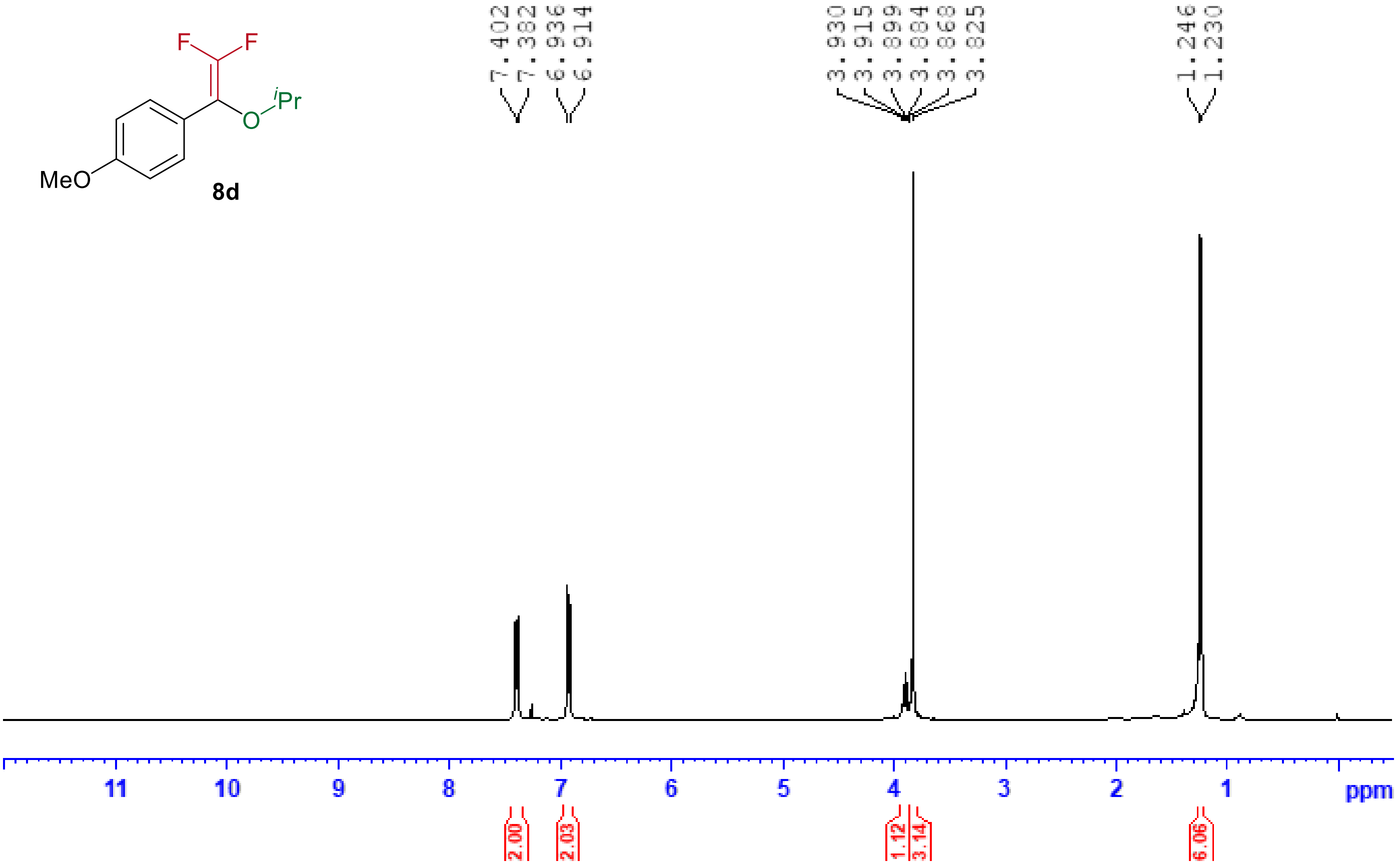

^{13}C NMR of **8d**

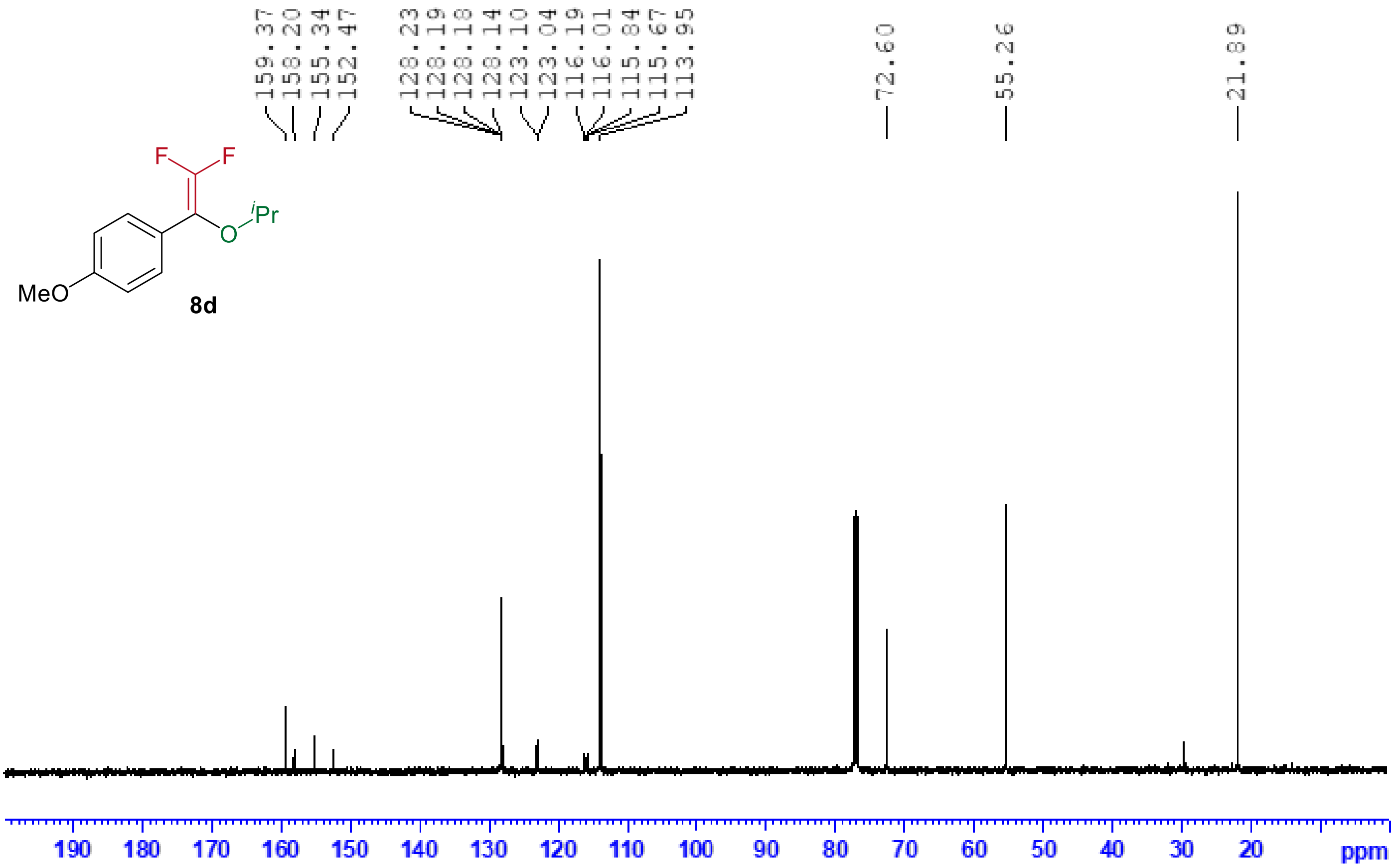

^{19}F NMR of **8d**

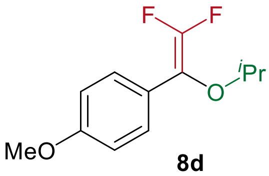

8d

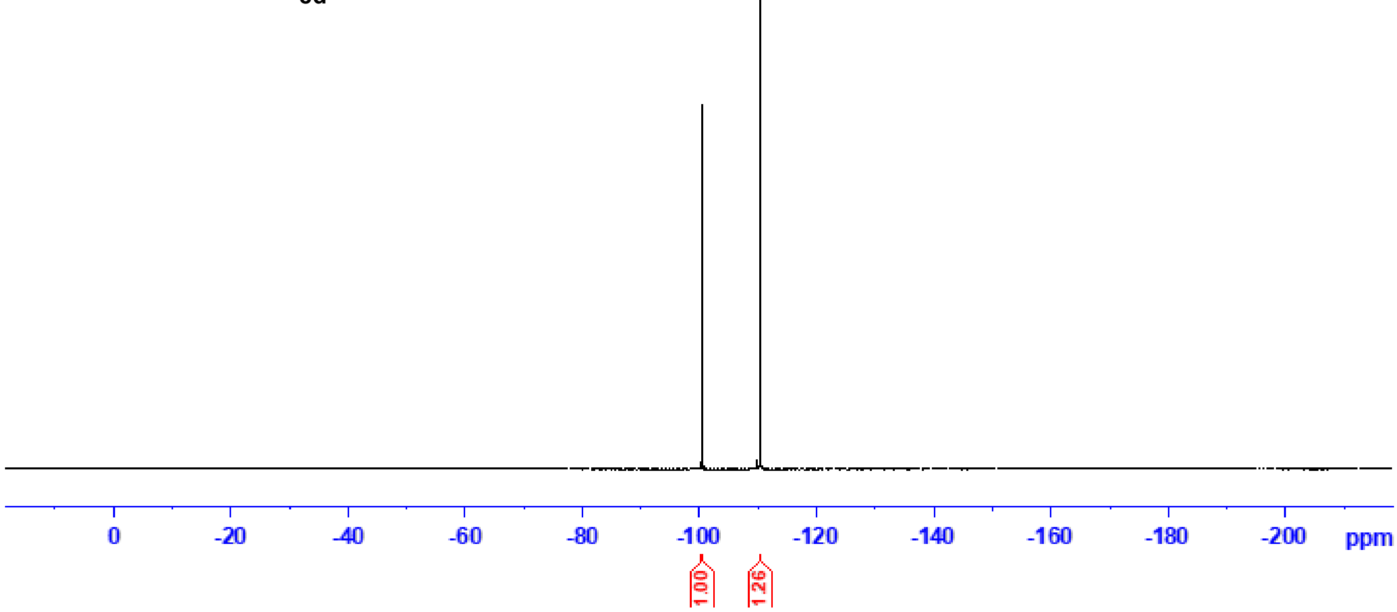

^{1}H NMR of **8e**

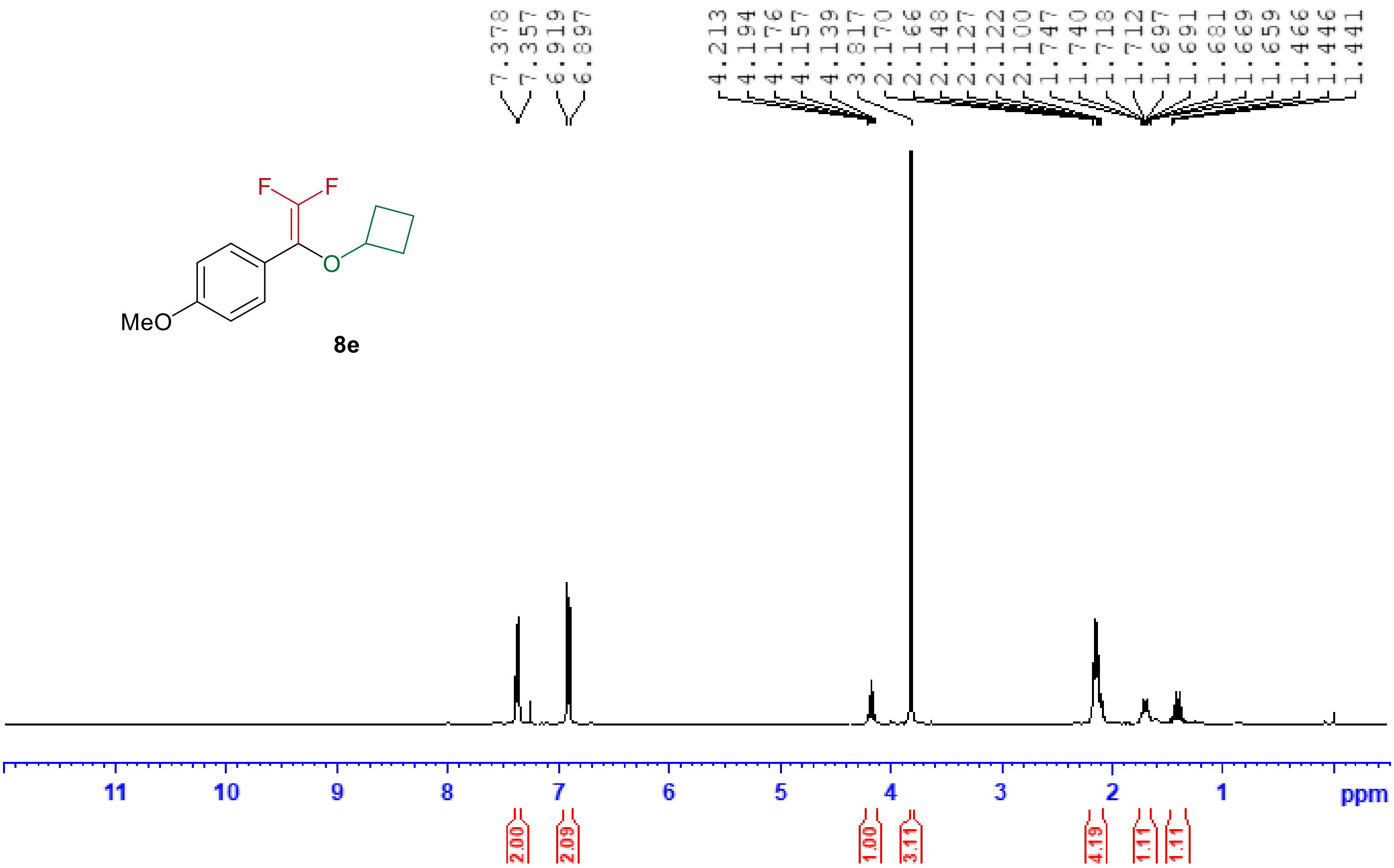

^{13}C NMR of **8e**

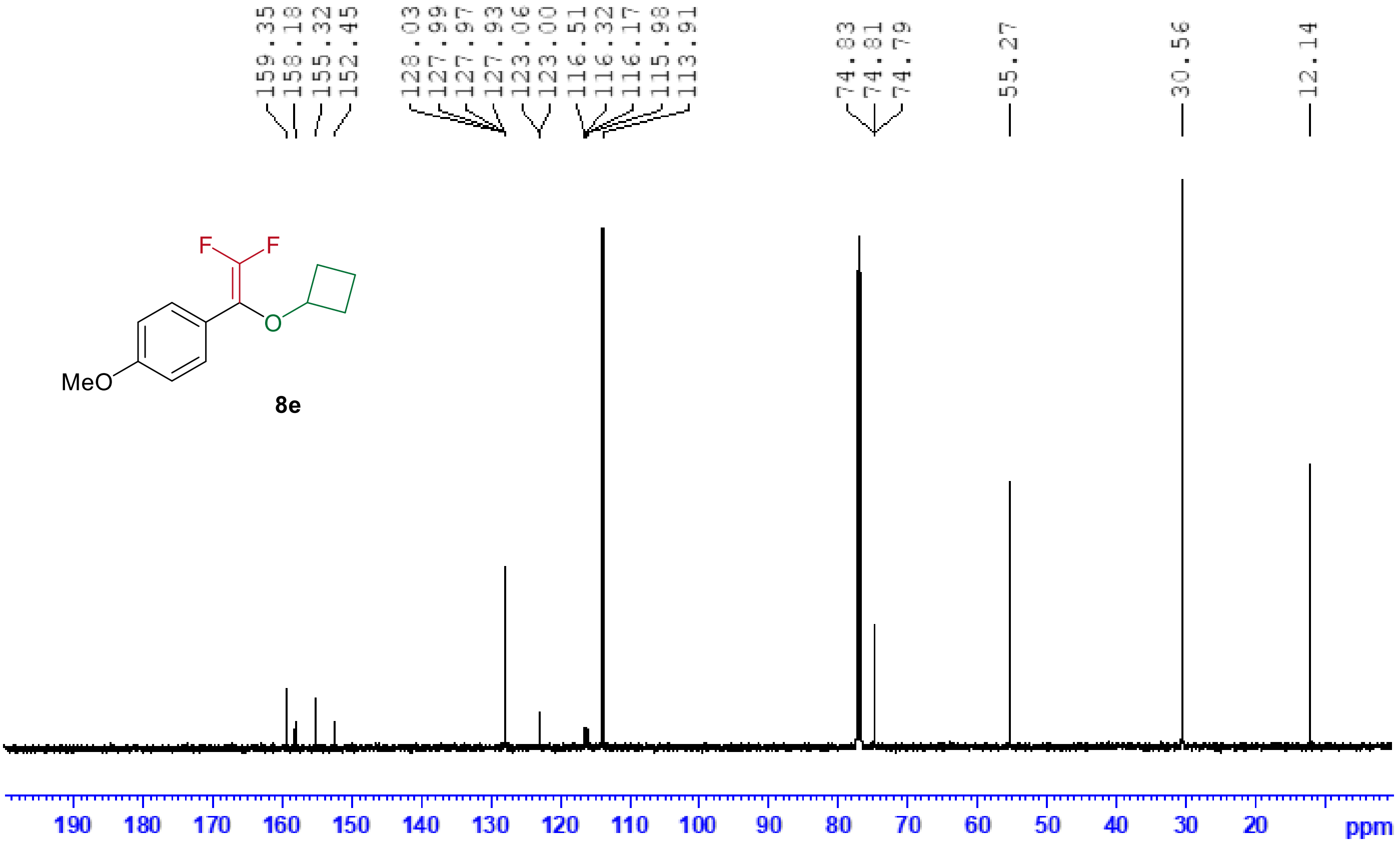

^{19}F NMR of **8e**

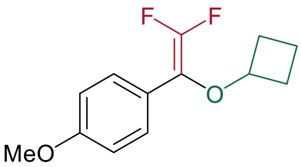

8e

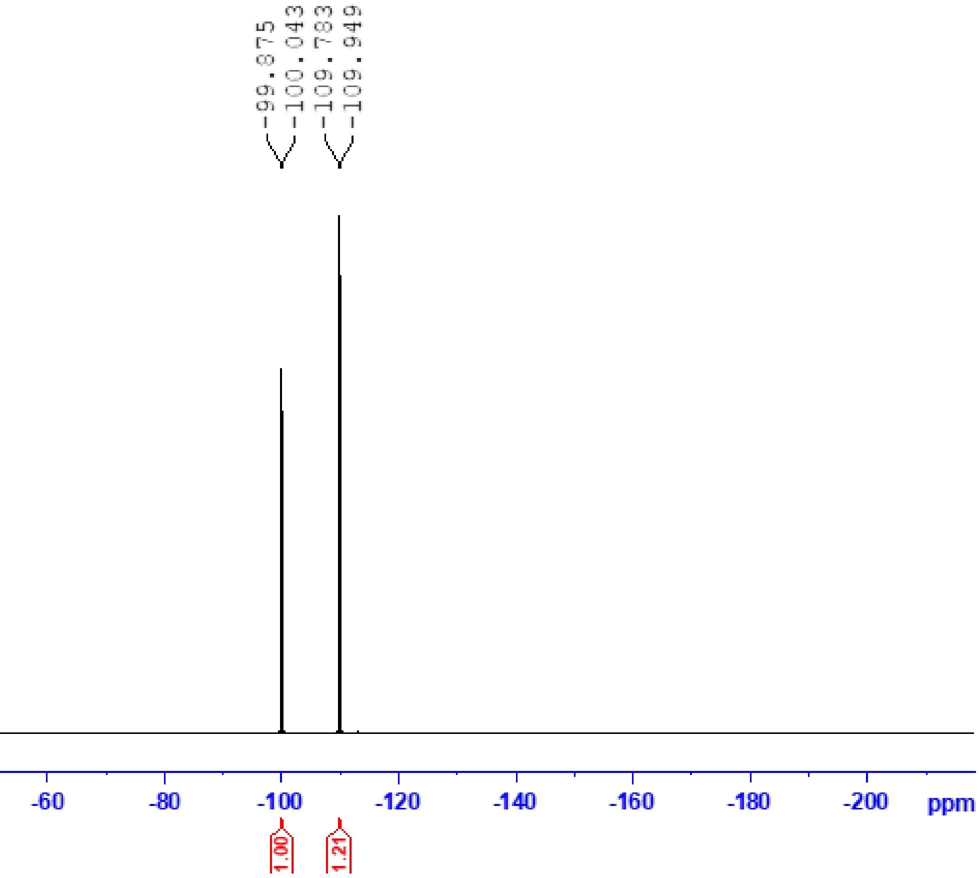

^{1}H NMR of **8f**

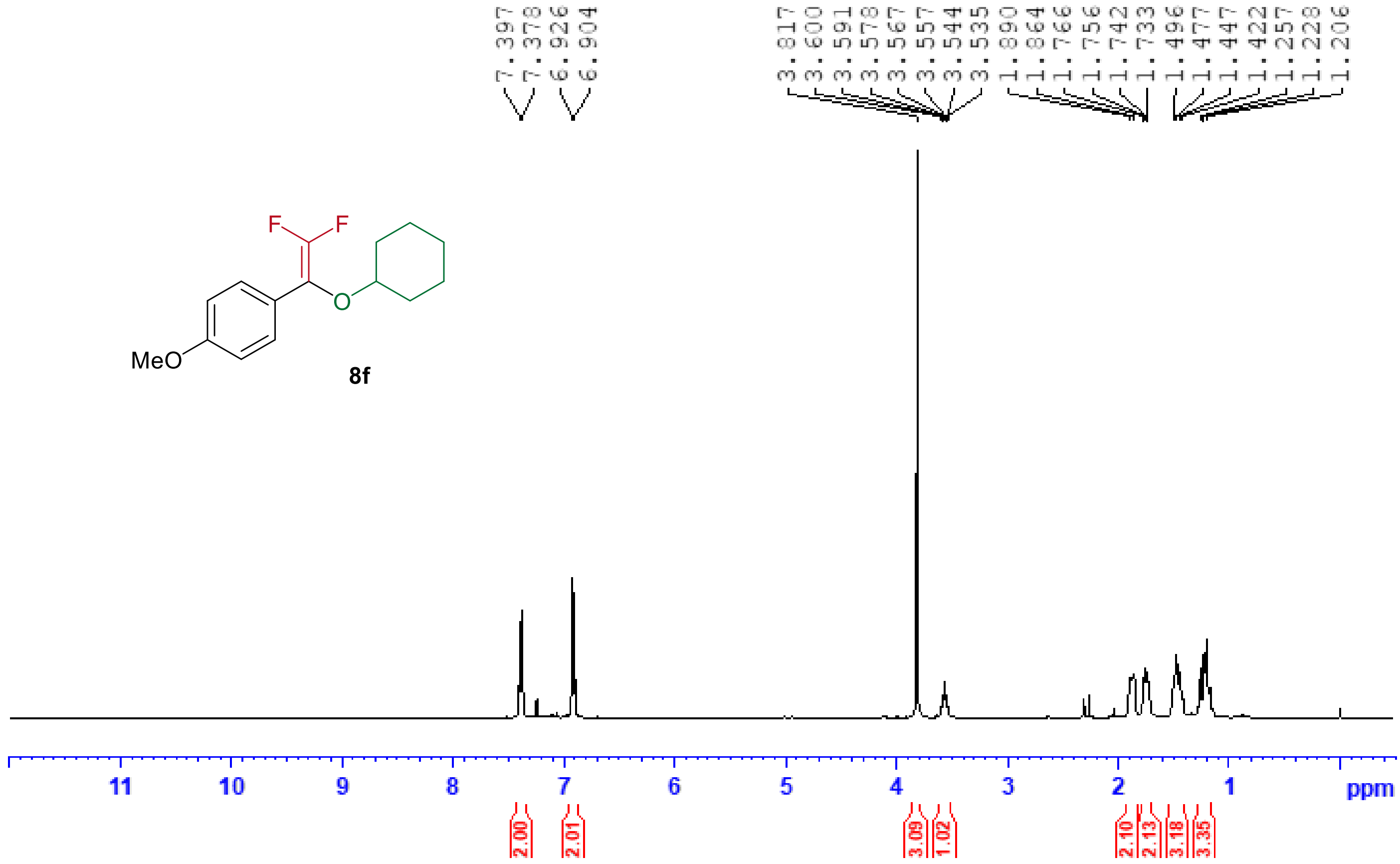

^{13}C NMR of **8f**

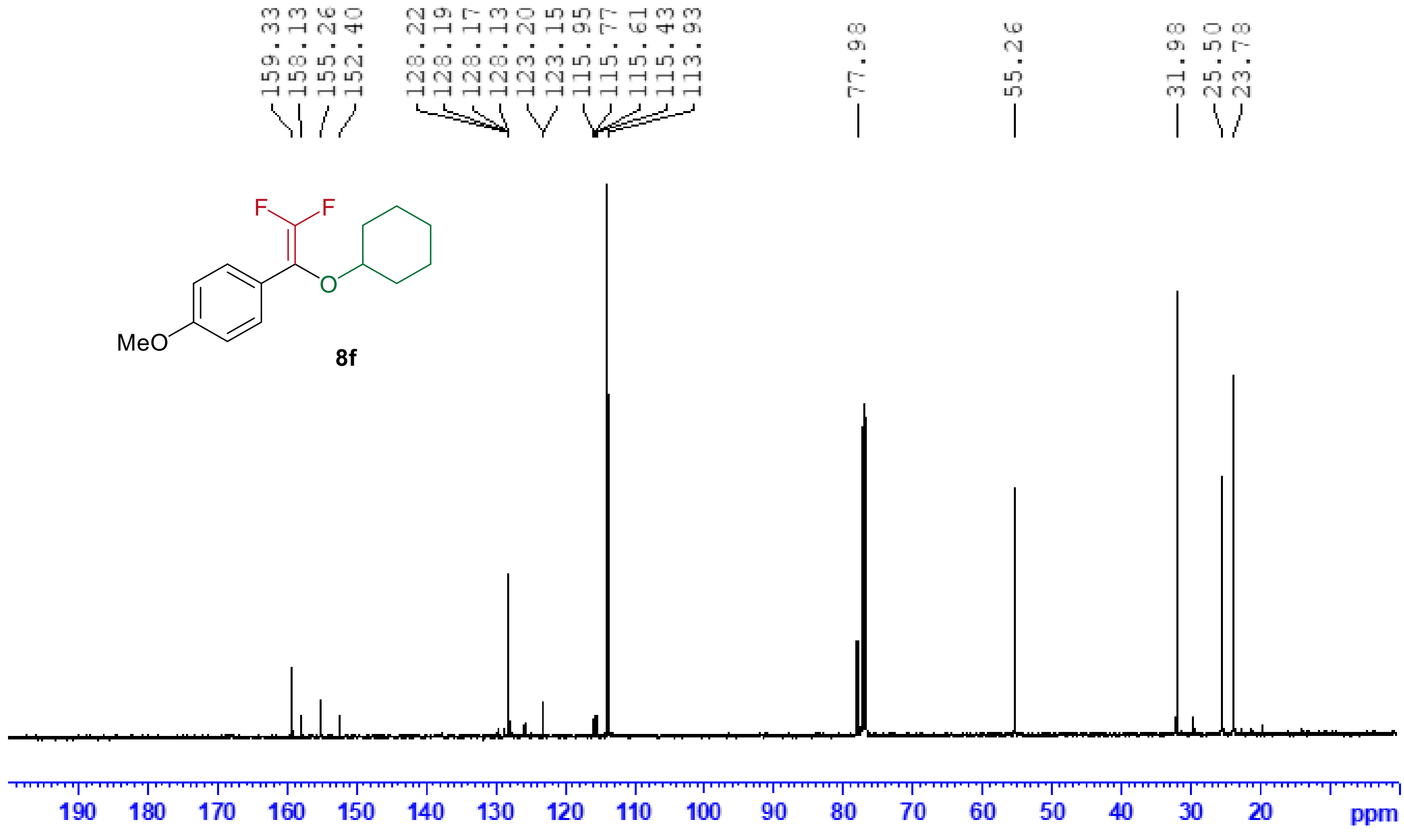

^{19}F NMR of **8f**

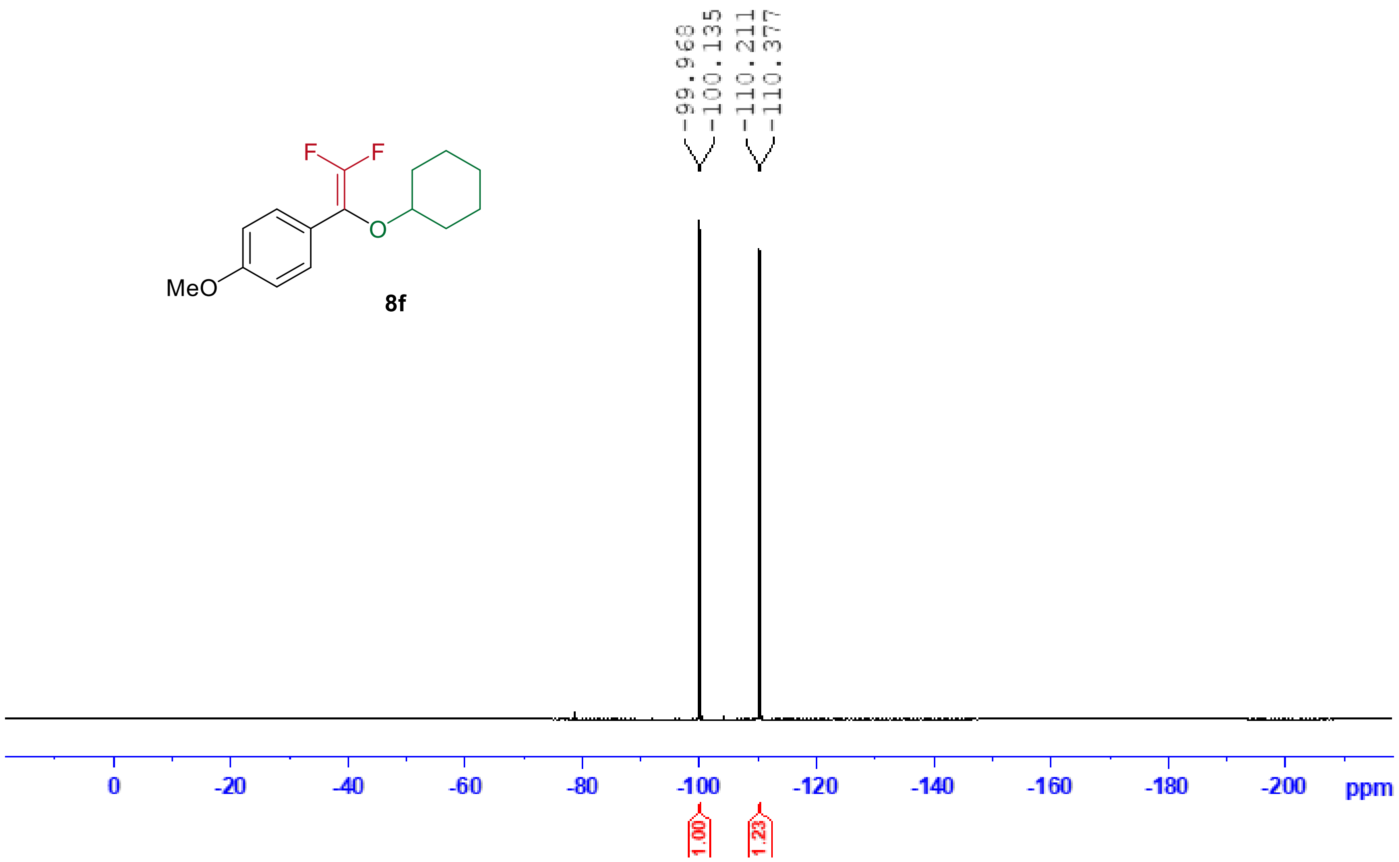

F
F
O
MeO
8f
-99.968
-100.135
-110.211
-110.377
0
-20
-40
-60
-80
-100
-120
-140
-160
-180
-200
ppm
1.00
1.23

^{1}H NMR of **8g**

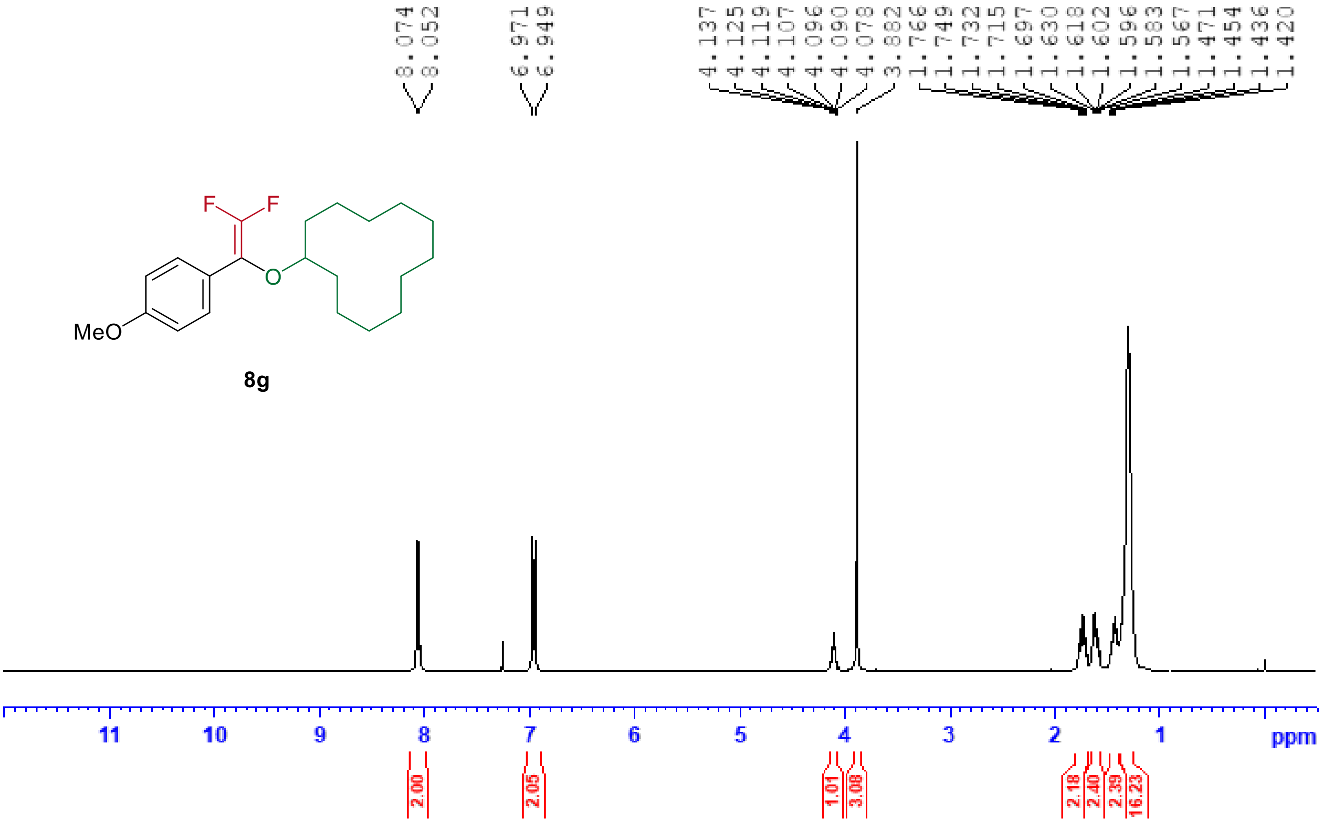

^{13}C NMR of **8g**

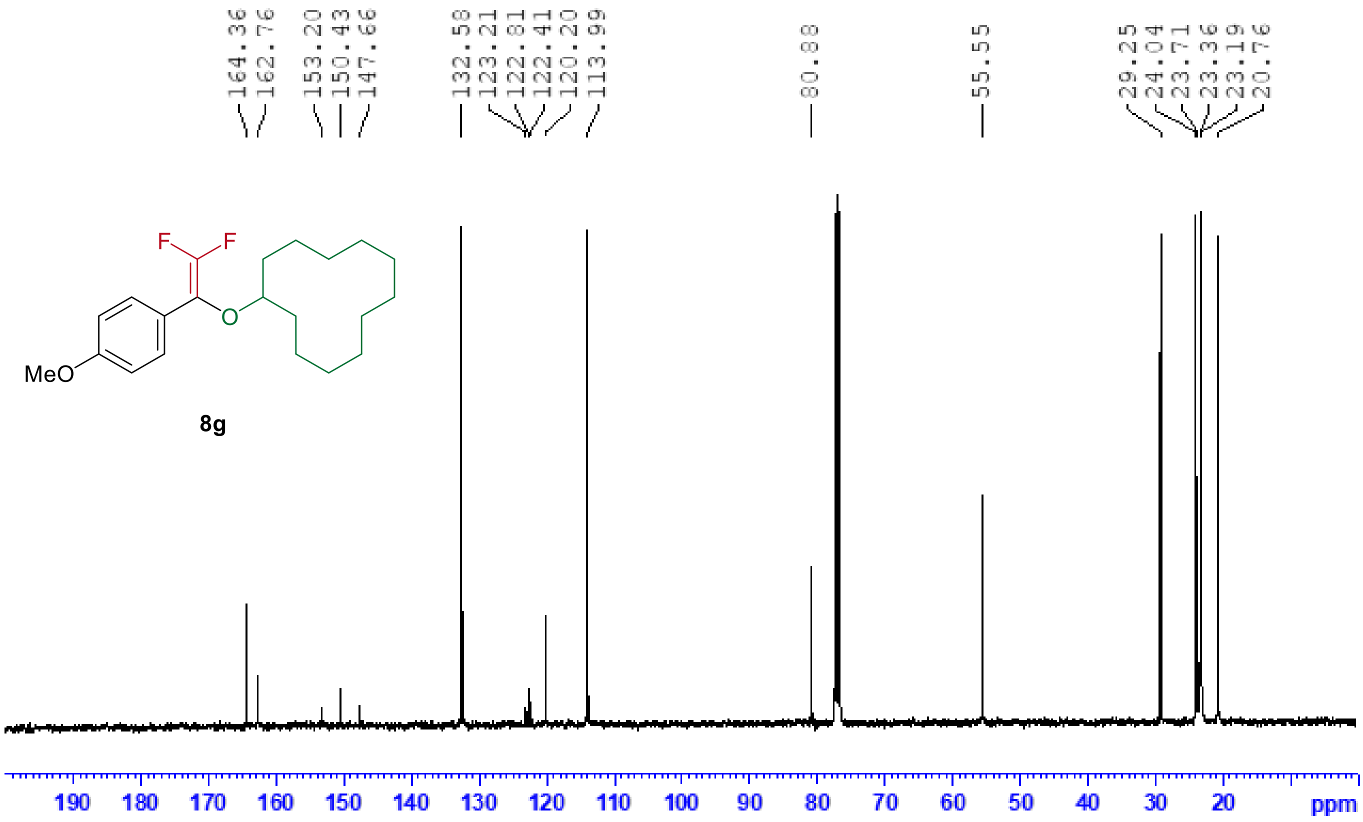

^{19}F NMR of **8g**

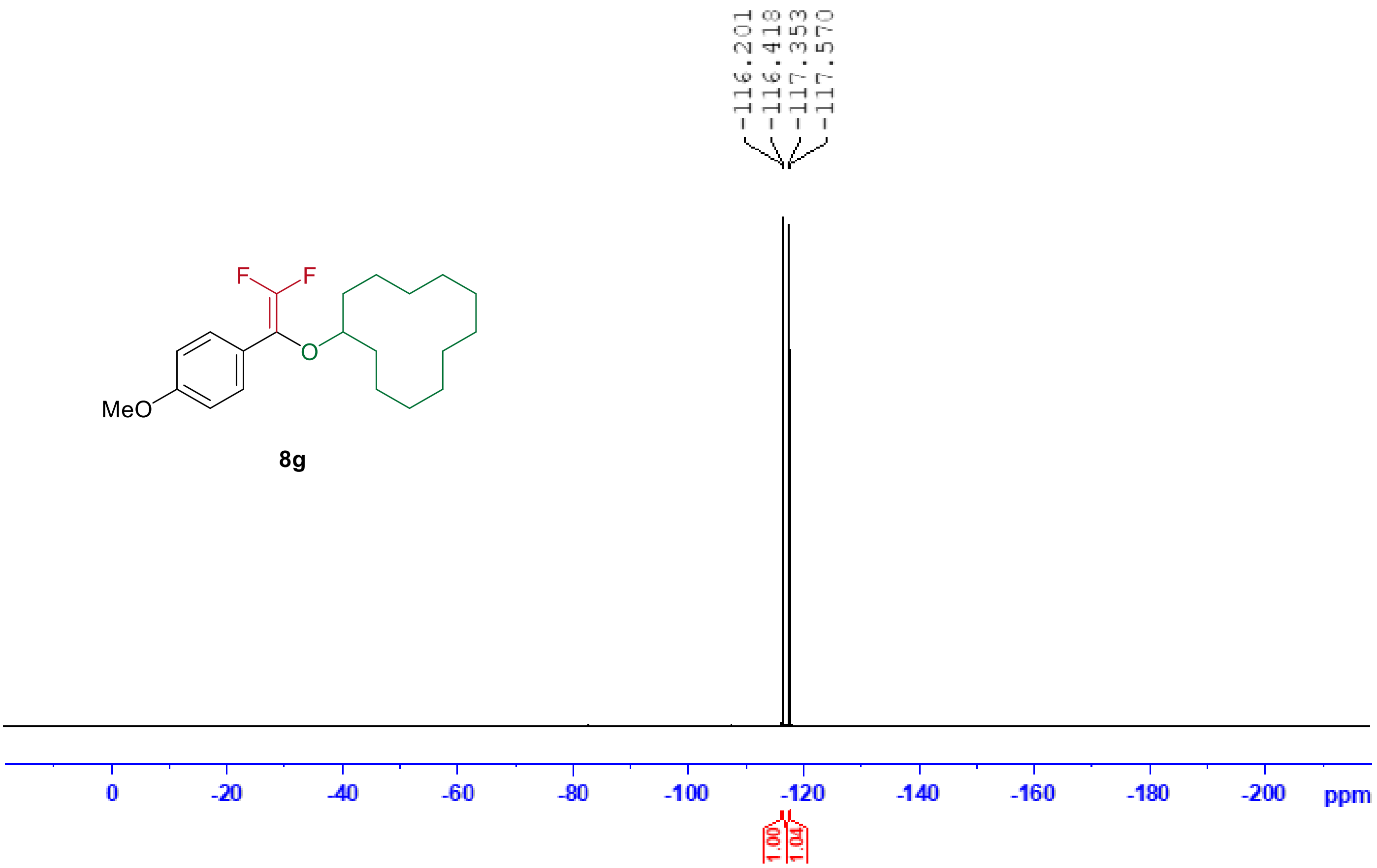

-116.201
-116.418
-117.353
-117.570
F
F
O
MeO
8g
0
-20
-40
-60
-80
-100
-120
-140
-160
-180
-200
ppm
1.00
1.04

^{1}H NMR of **8h**

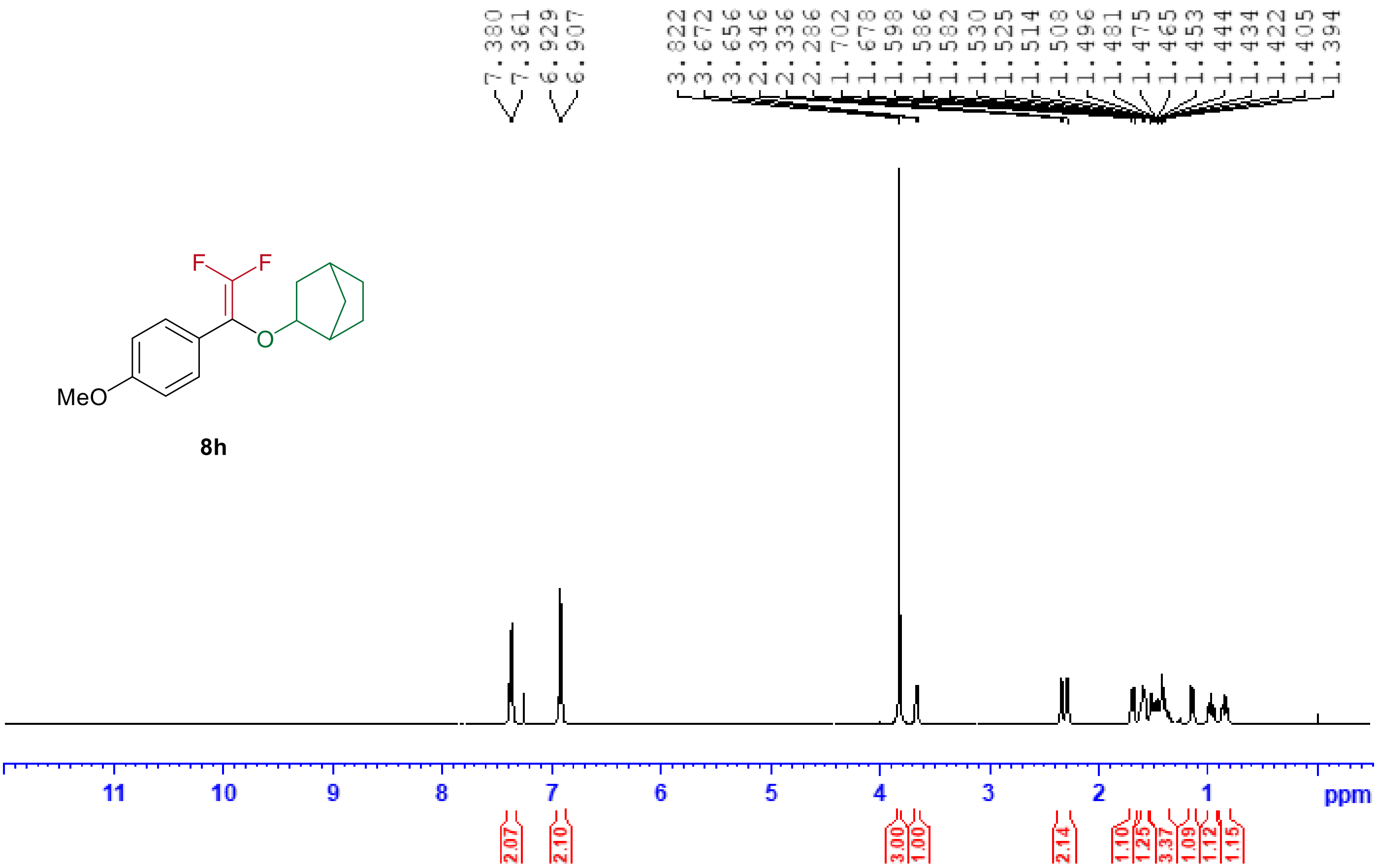

^{13}C NMR of **8h**

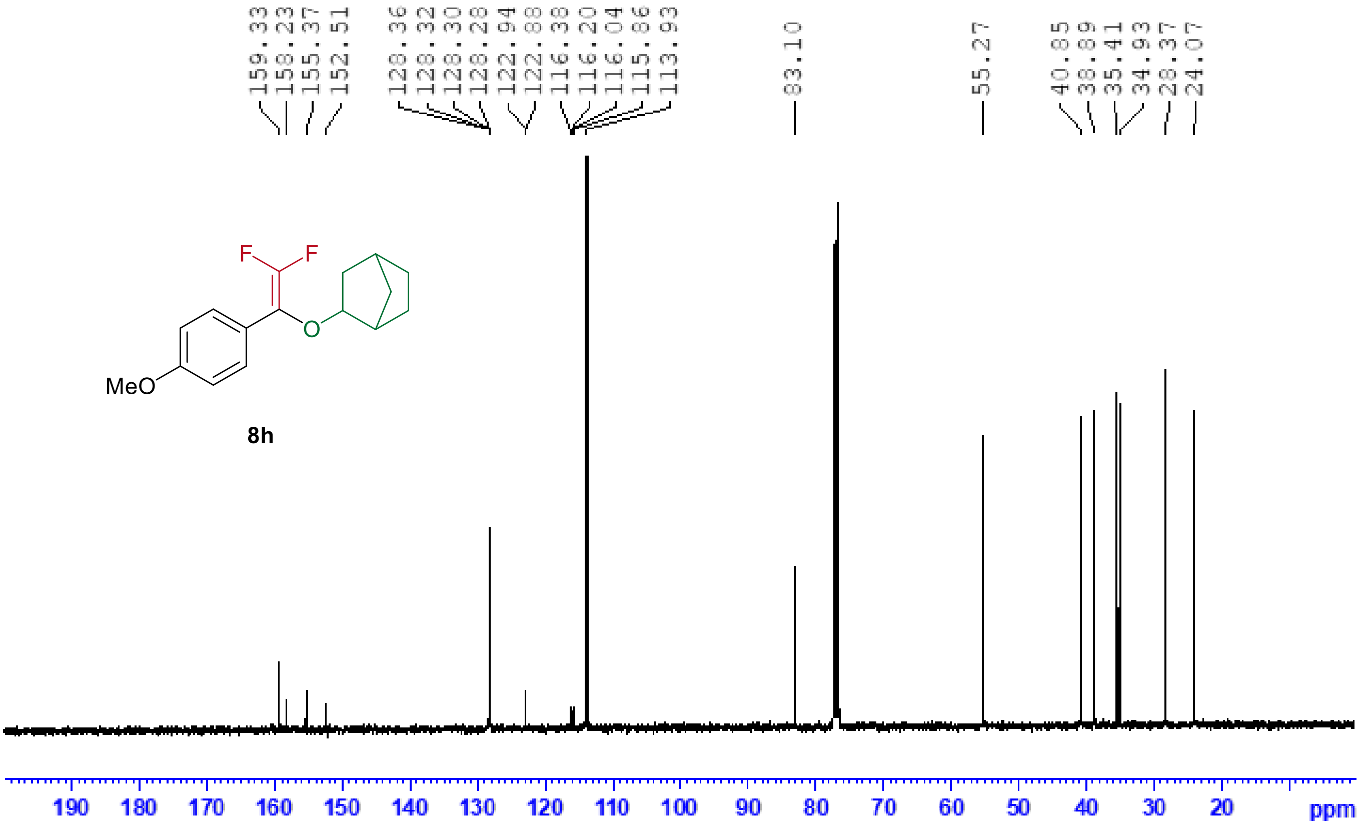

^{19}F NMR of **8h**

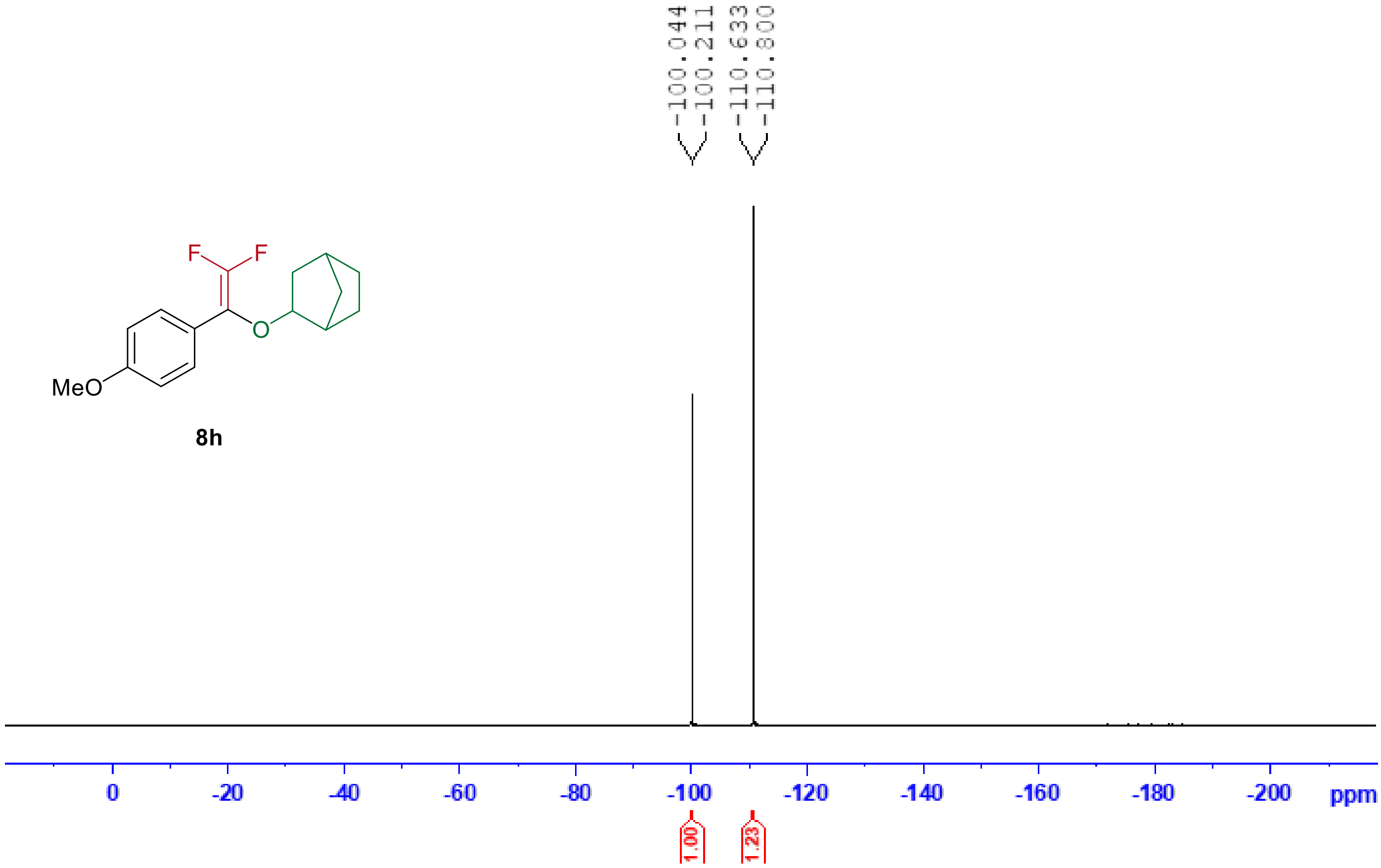

8h

^{1}H NMR of **8i**

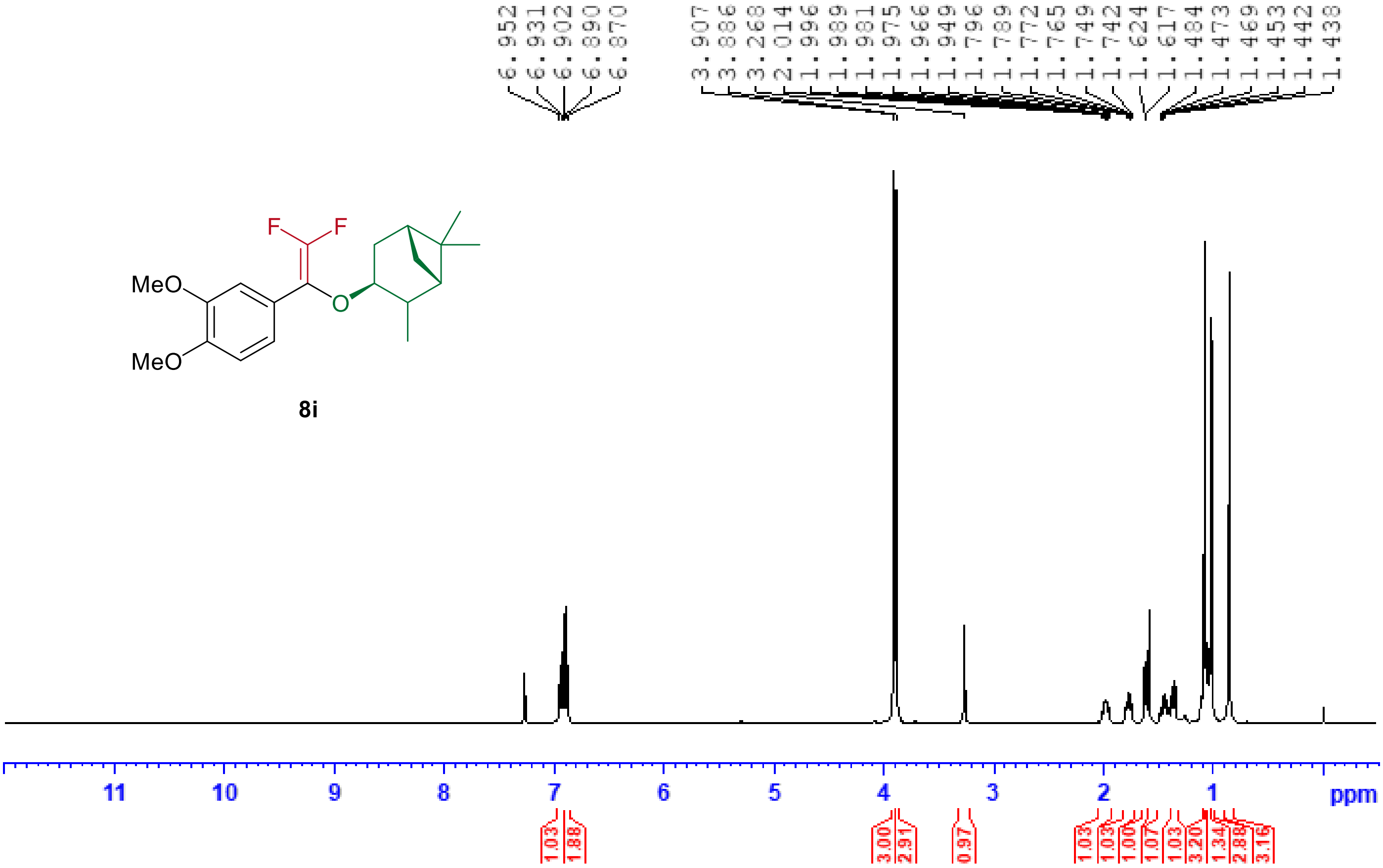

^{13}C NMR of **8i**

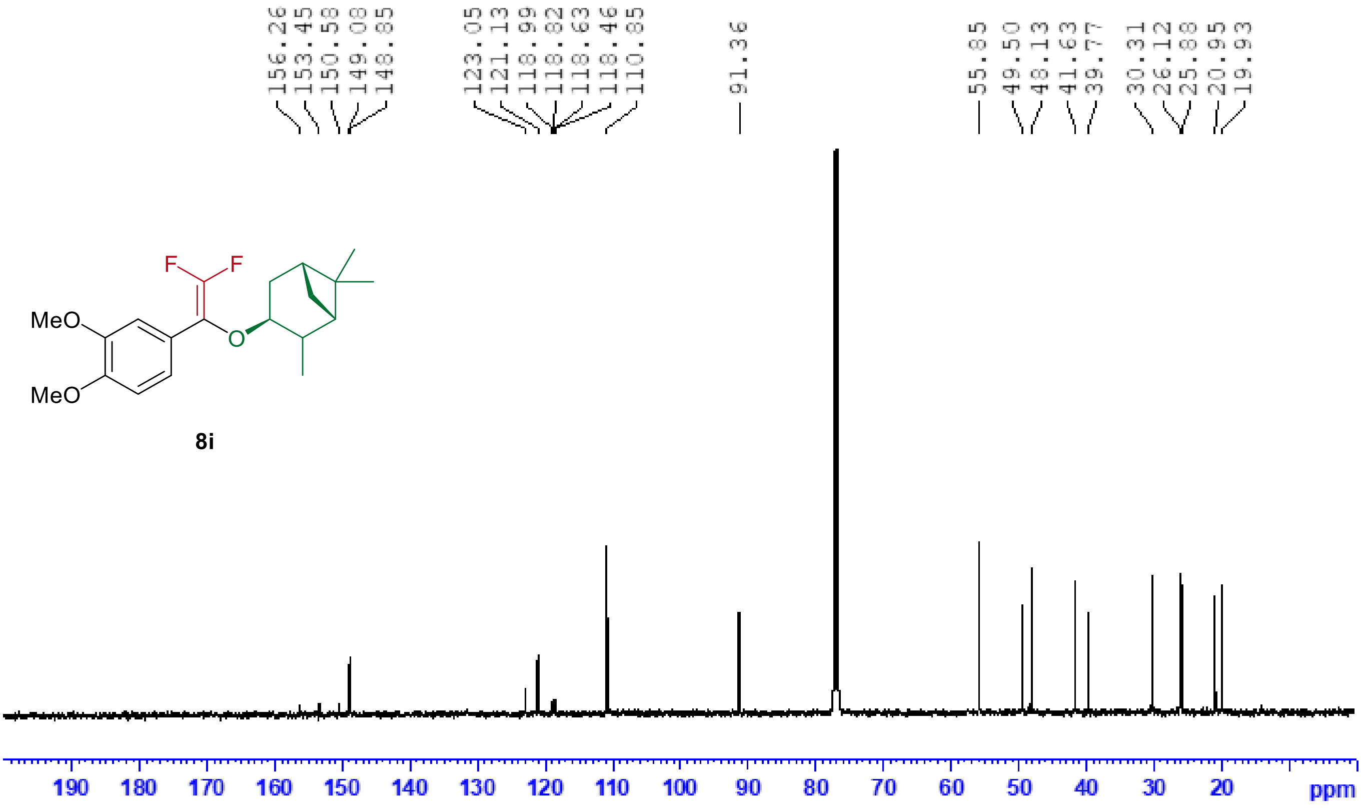

^{19}F NMR of **8i**

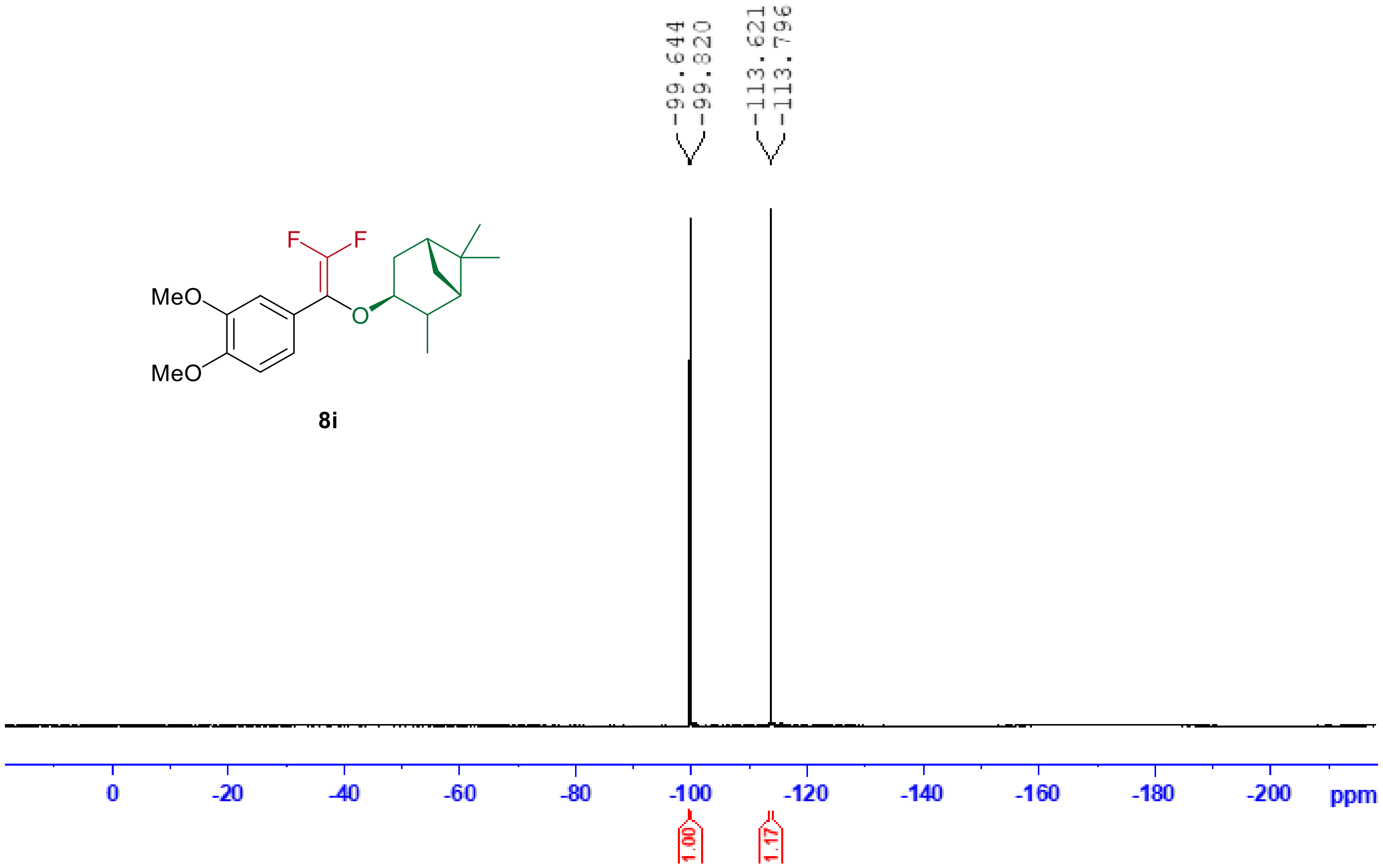

-99.644
-99.820
-113.621
-113.796
MeO
MeO
F
F
O
8i
0
-20
-40
-60
-80
-100
-120
-140
-160
-180
-200
ppm
1.00
1.17

^{1}H NMR of **8j**

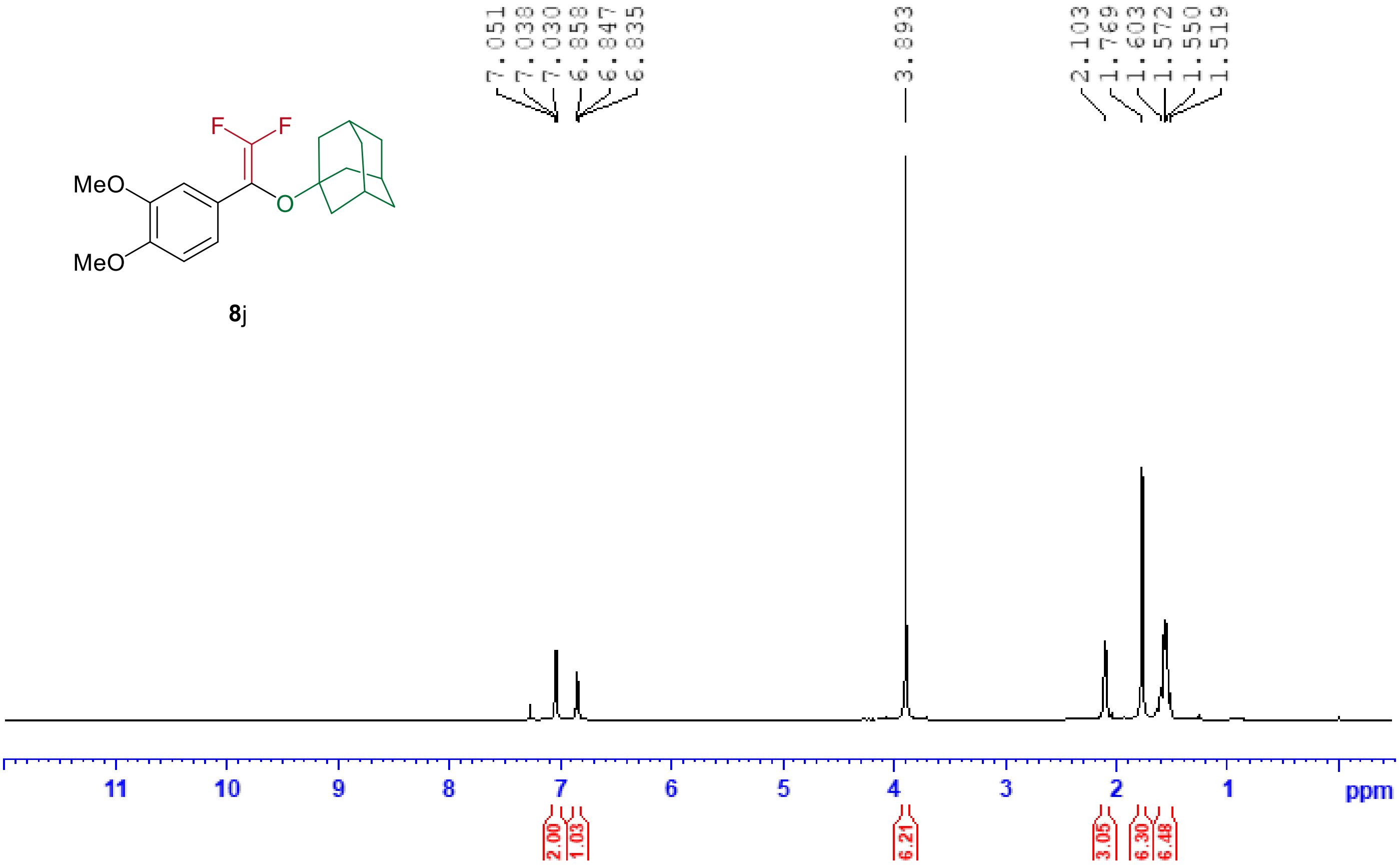

^{13}C NMR of **8j**

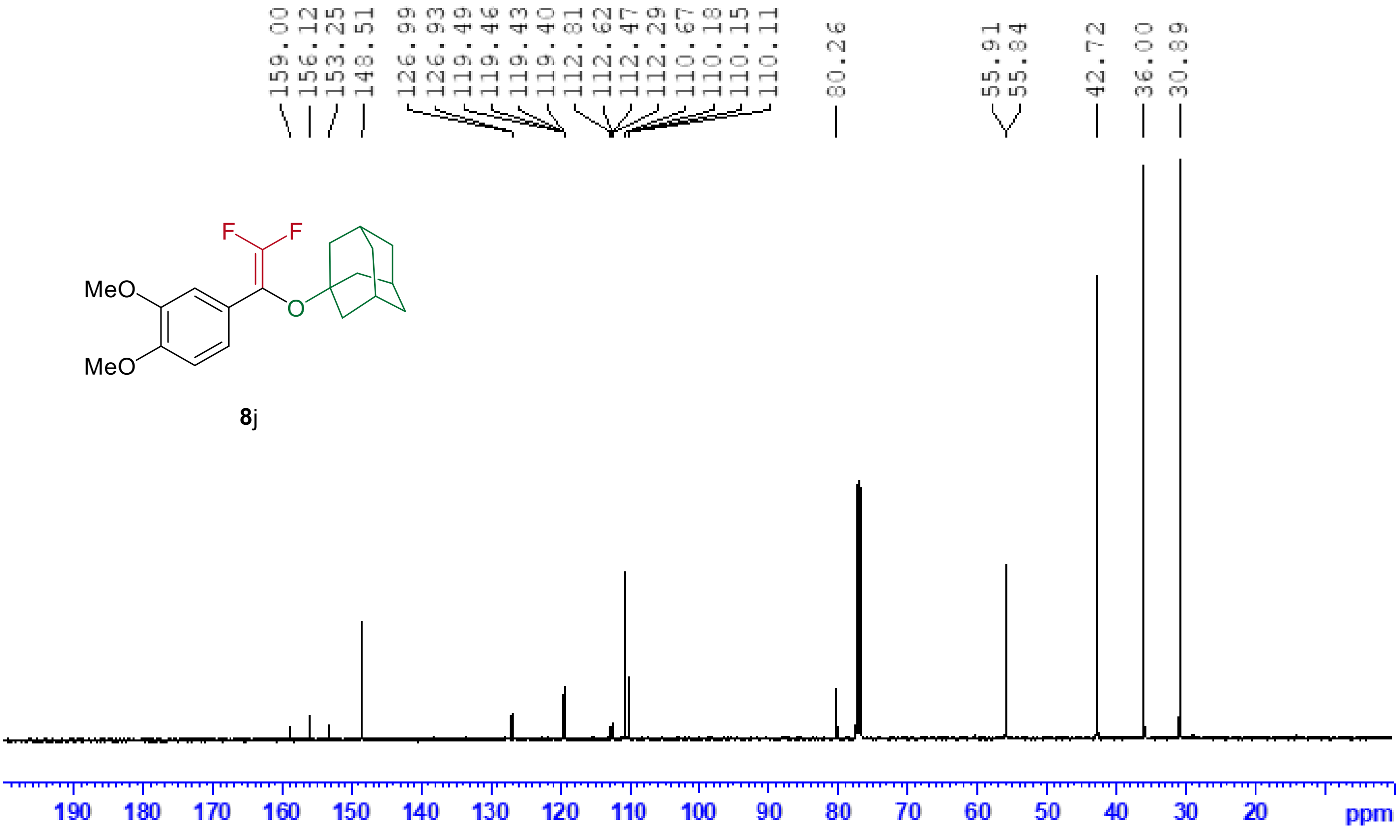

^{19}F NMR of **8j**

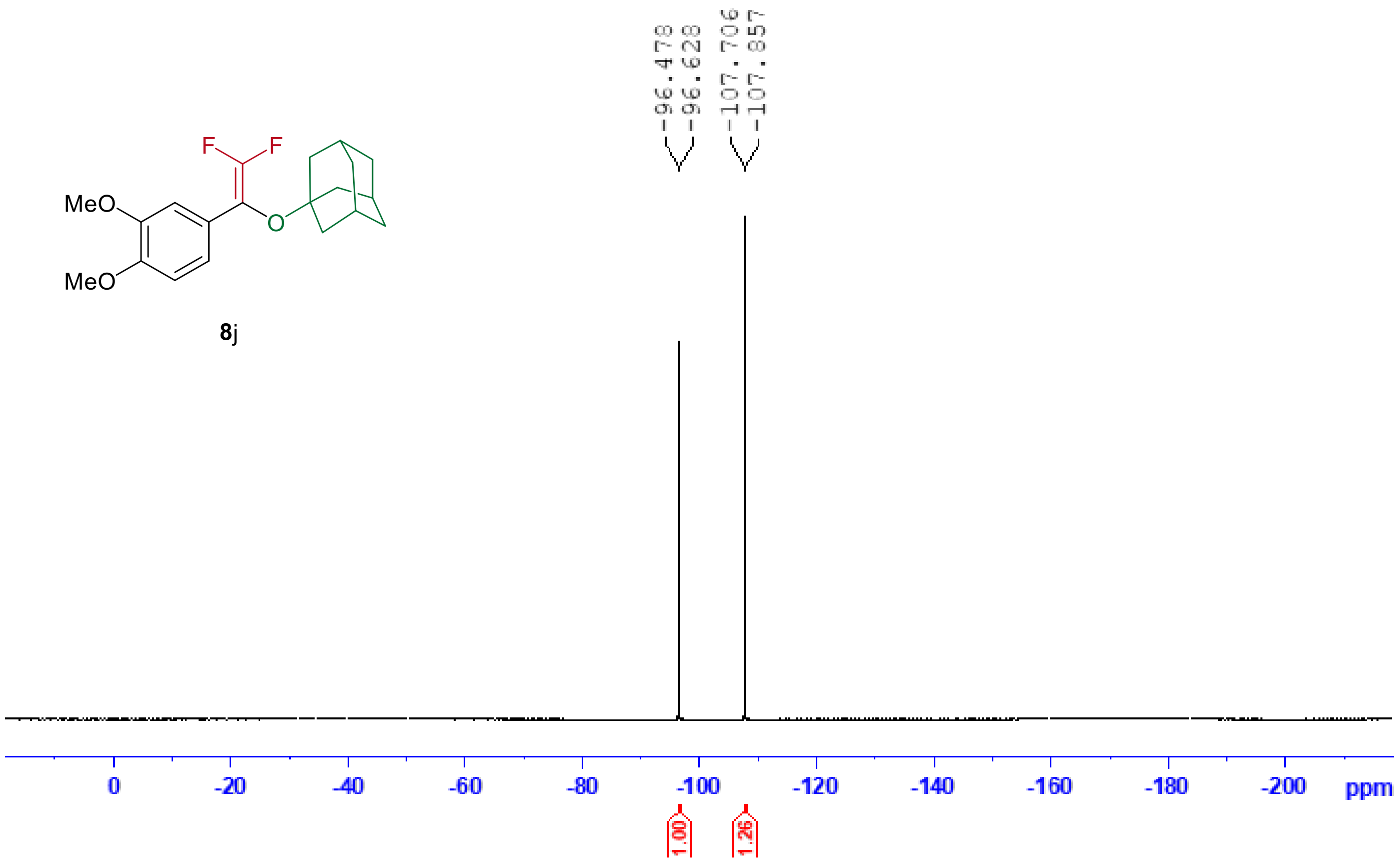

F
F
MeO
MeO
O
8j
-96.478
-96.628
-107.706
-107.857
0
-20
-40
-60
-80
-100
-120
-140
-160
-180
-200
ppm
1.00
1.26

^{1}H NMR of **8k**

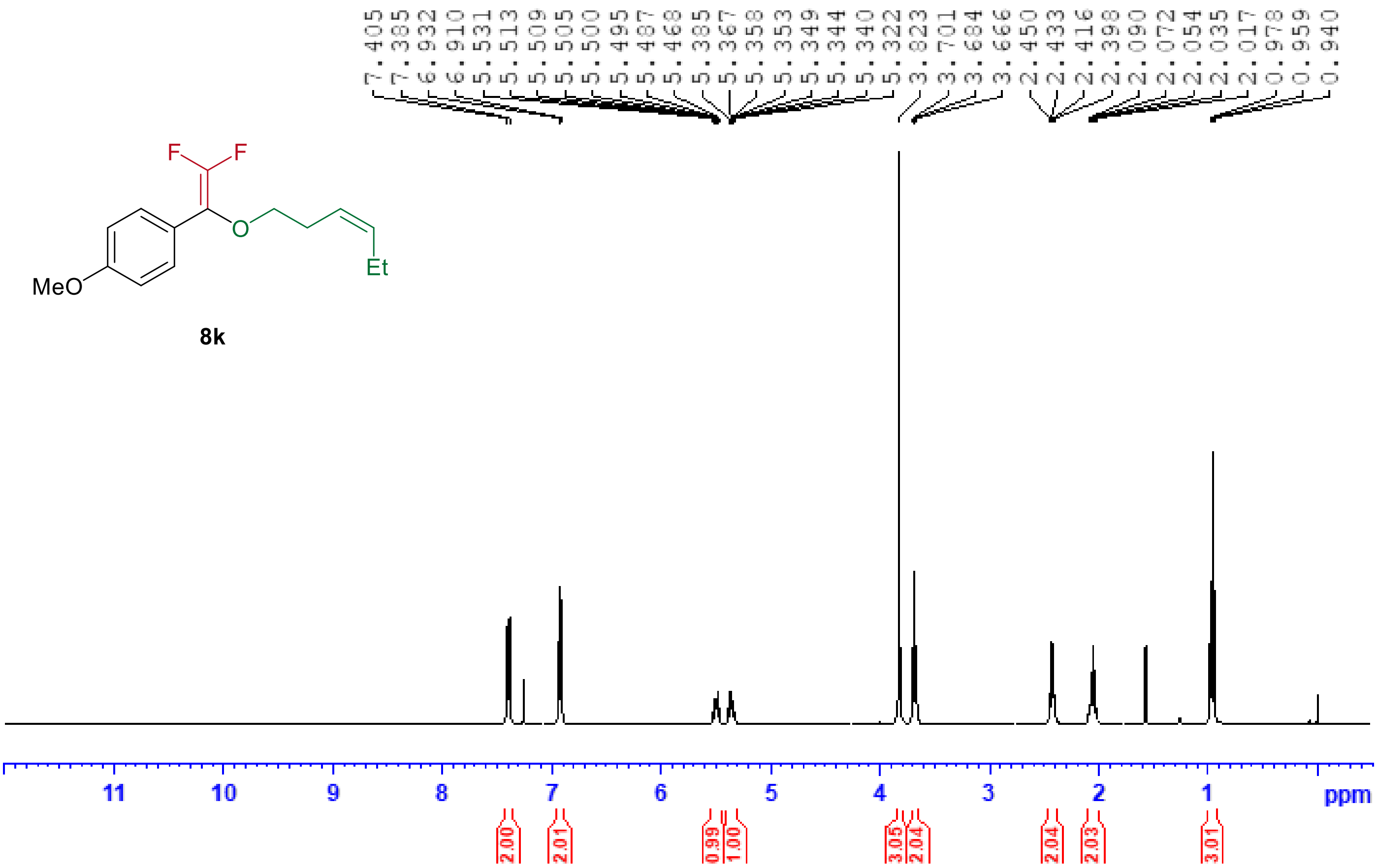

^{13}C NMR of **8k**

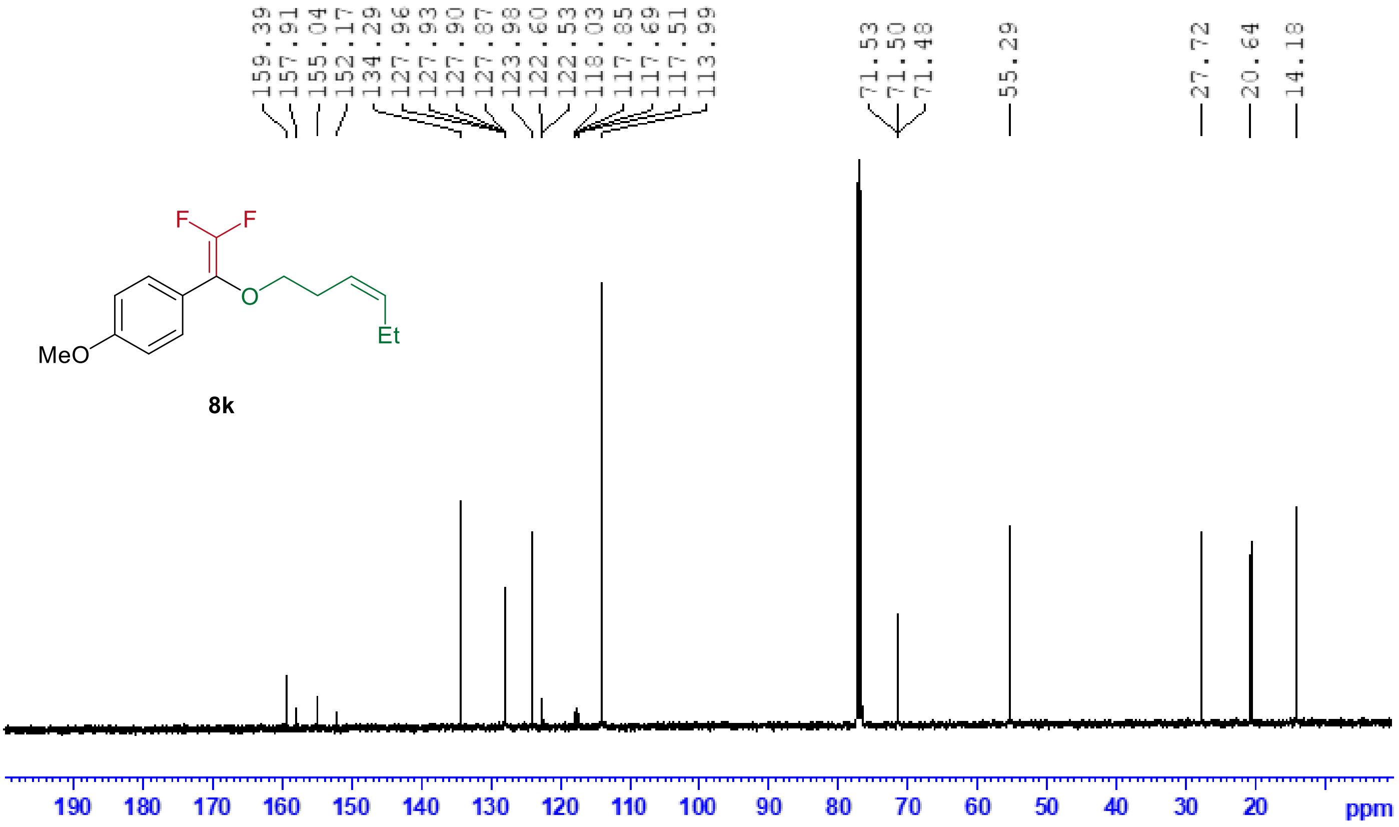

^{19}F NMR of **8k**

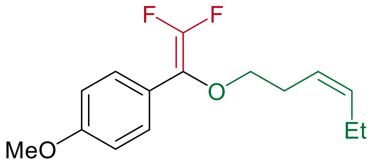

8k

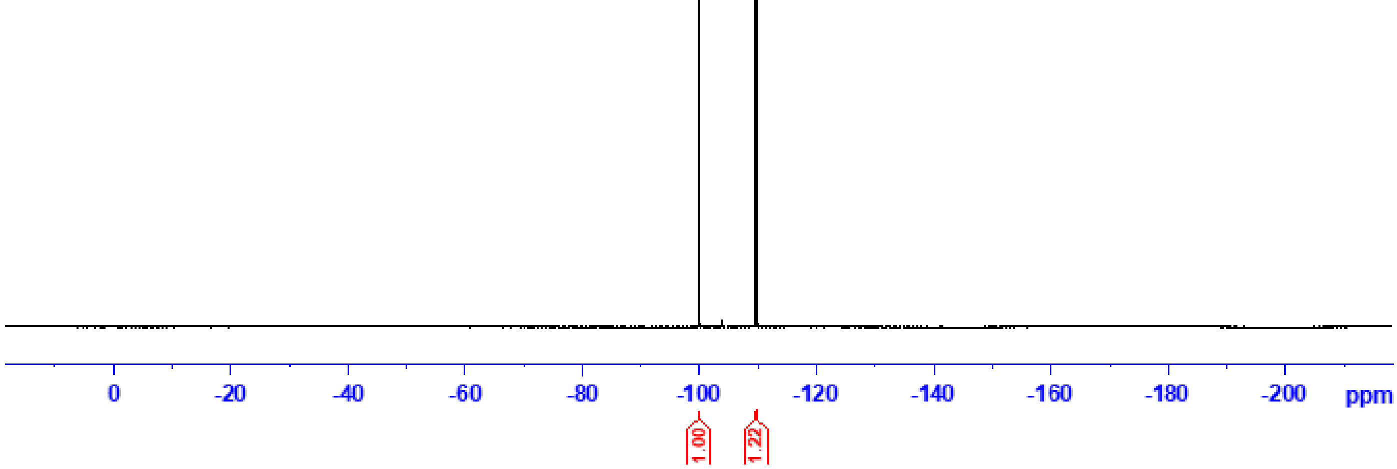

^{1}H NMR of **8l** + **8′**

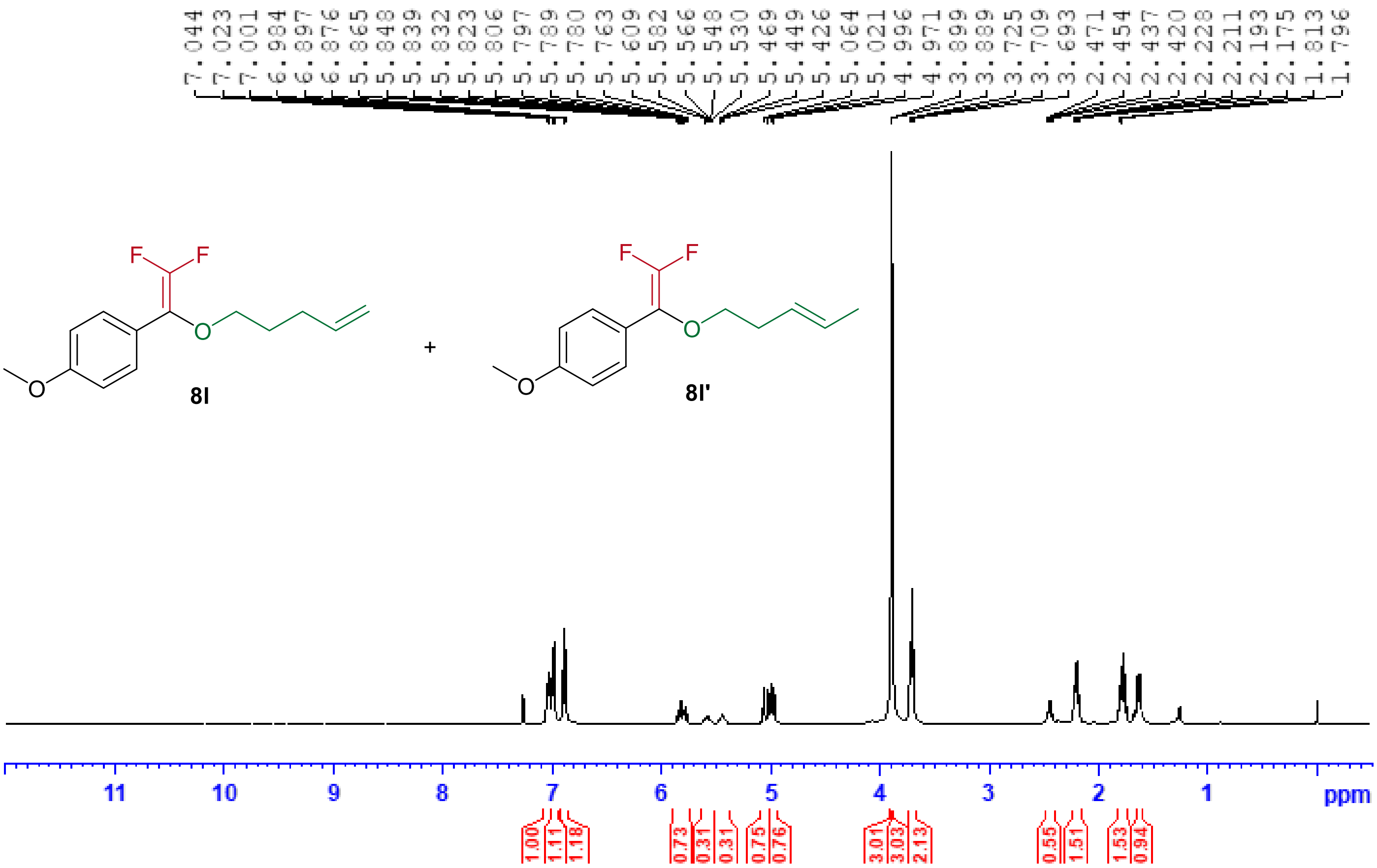

^{13}C NMR of **8l** + **8′**

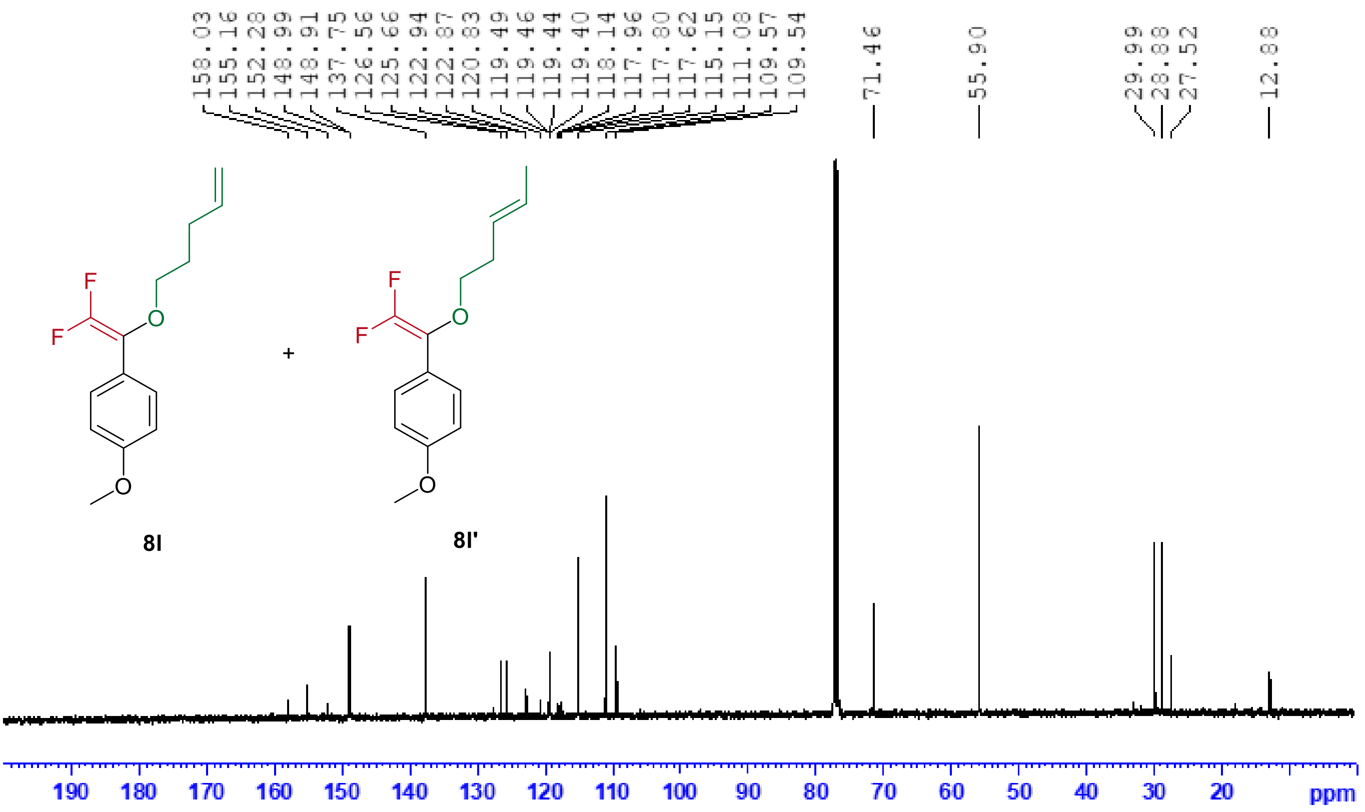

^{19}F NMR of **8l** + **8′**

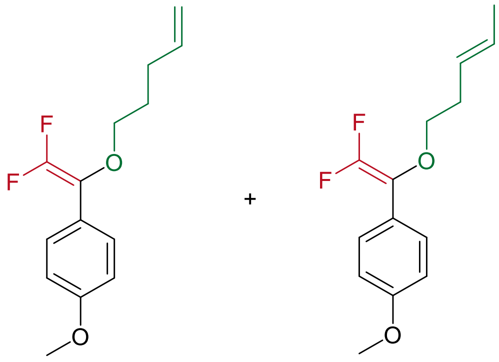

8l

8l'

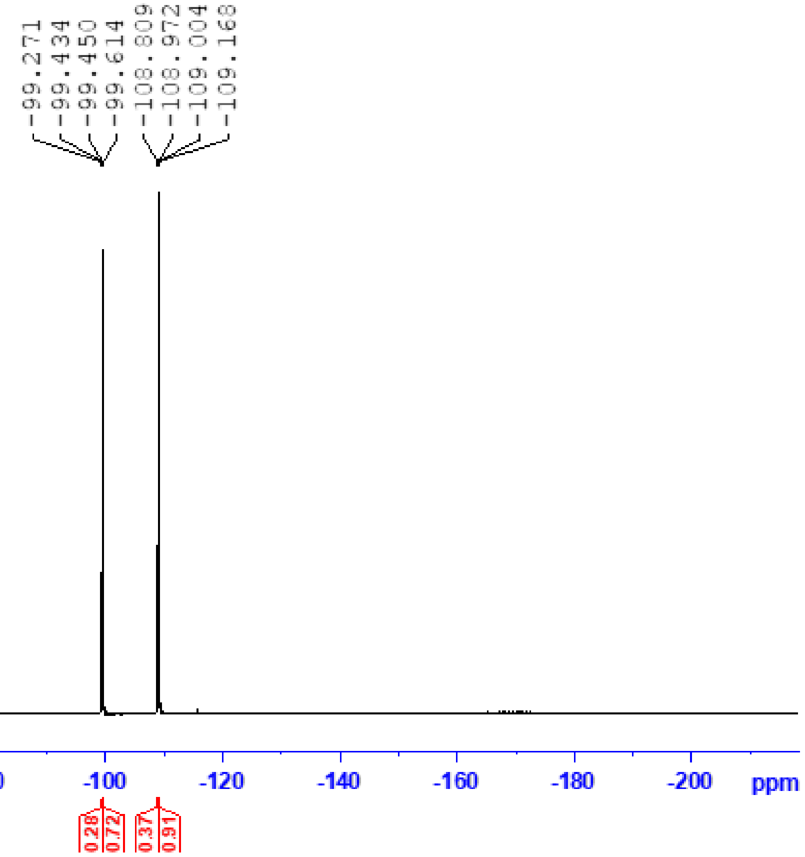

^{1}H NMR of **8m**

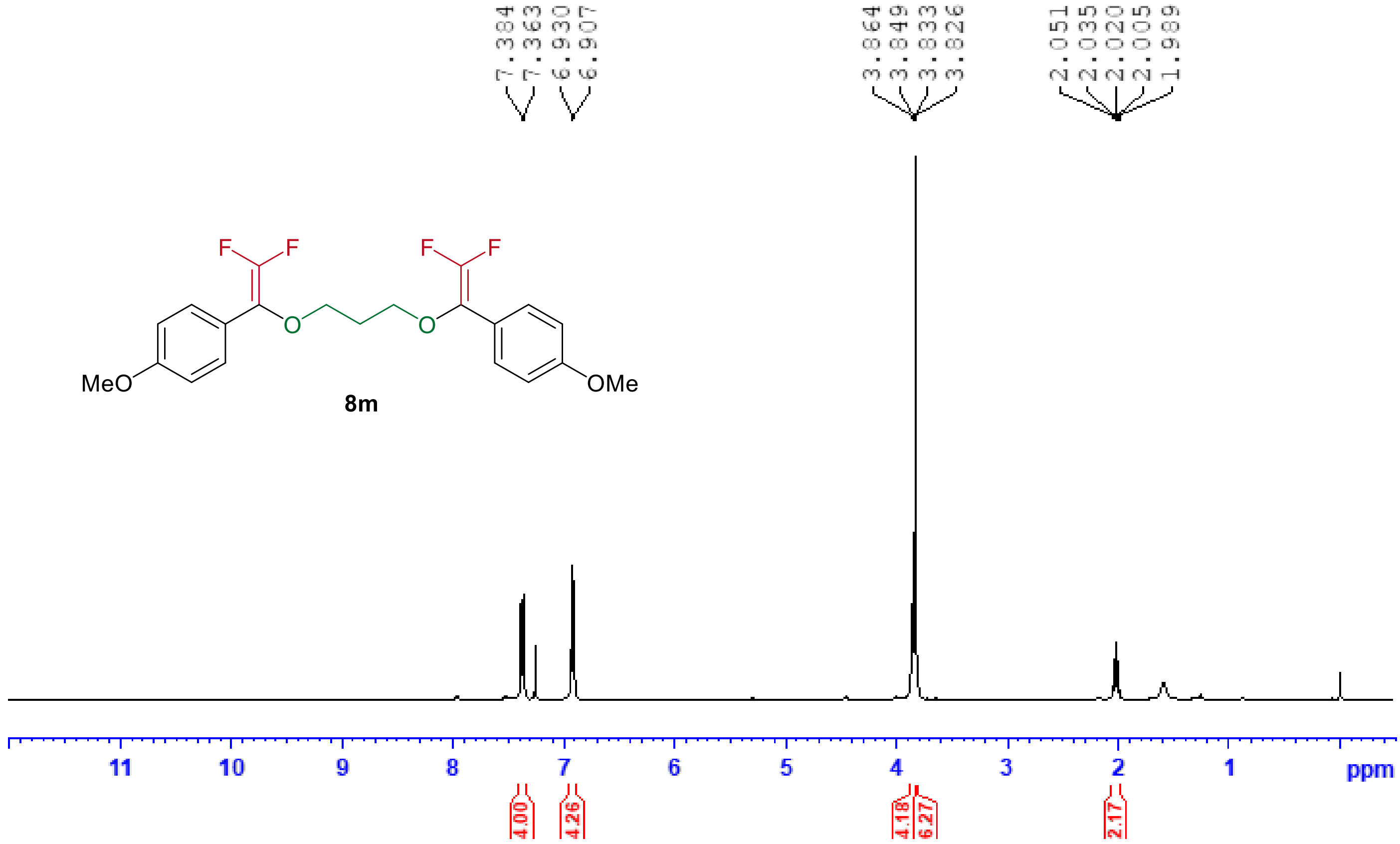

^{13}C NMR of **8m**

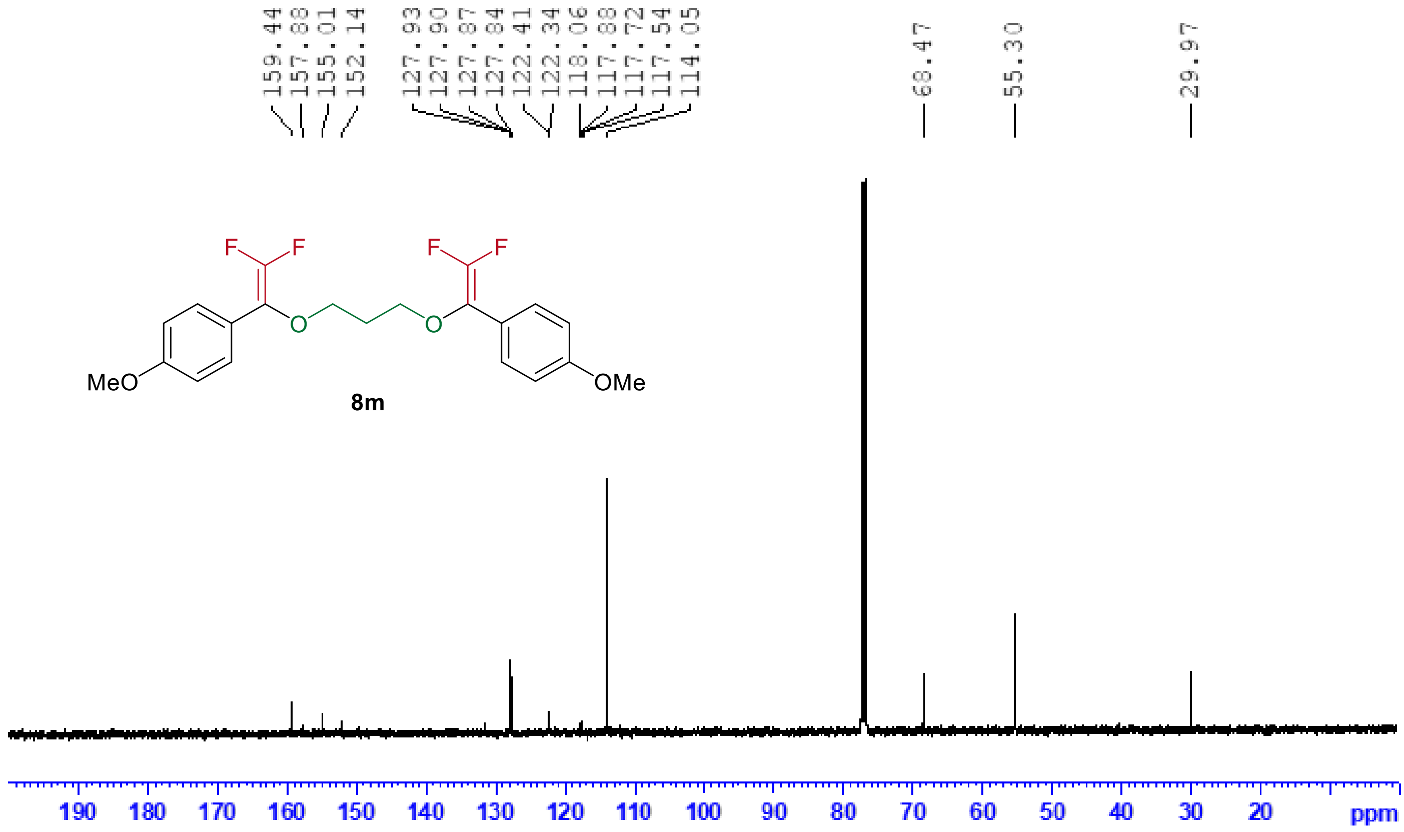

^{19}F NMR of **8m**

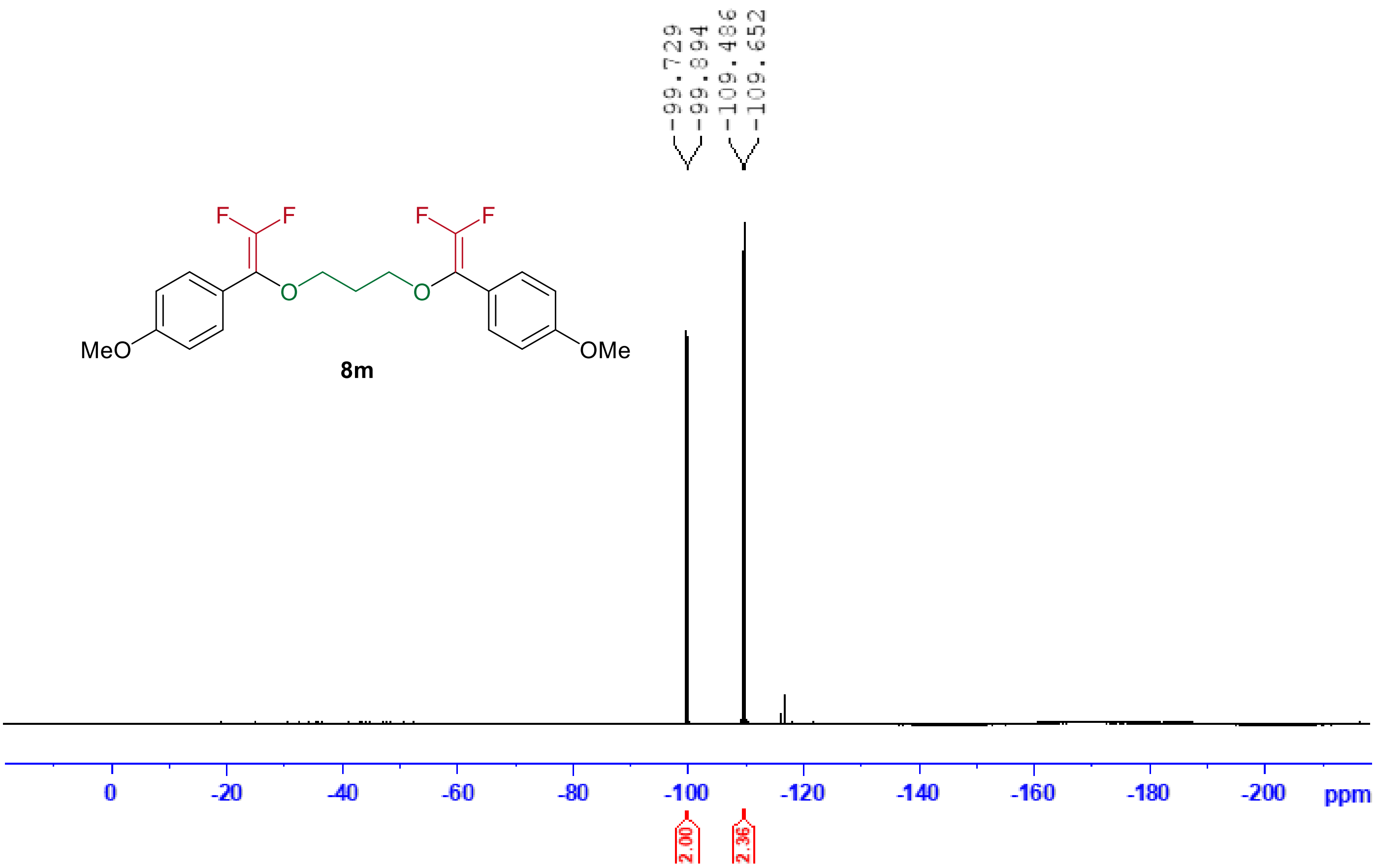

-99.729
-99.894
-109.486
-109.652
MeO
OMe
8m
0
-20
-40
-60
-80
-100
-120
-140
-160
-180
-200
ppm
2.00
2.36

^{1}H NMR of **8n**

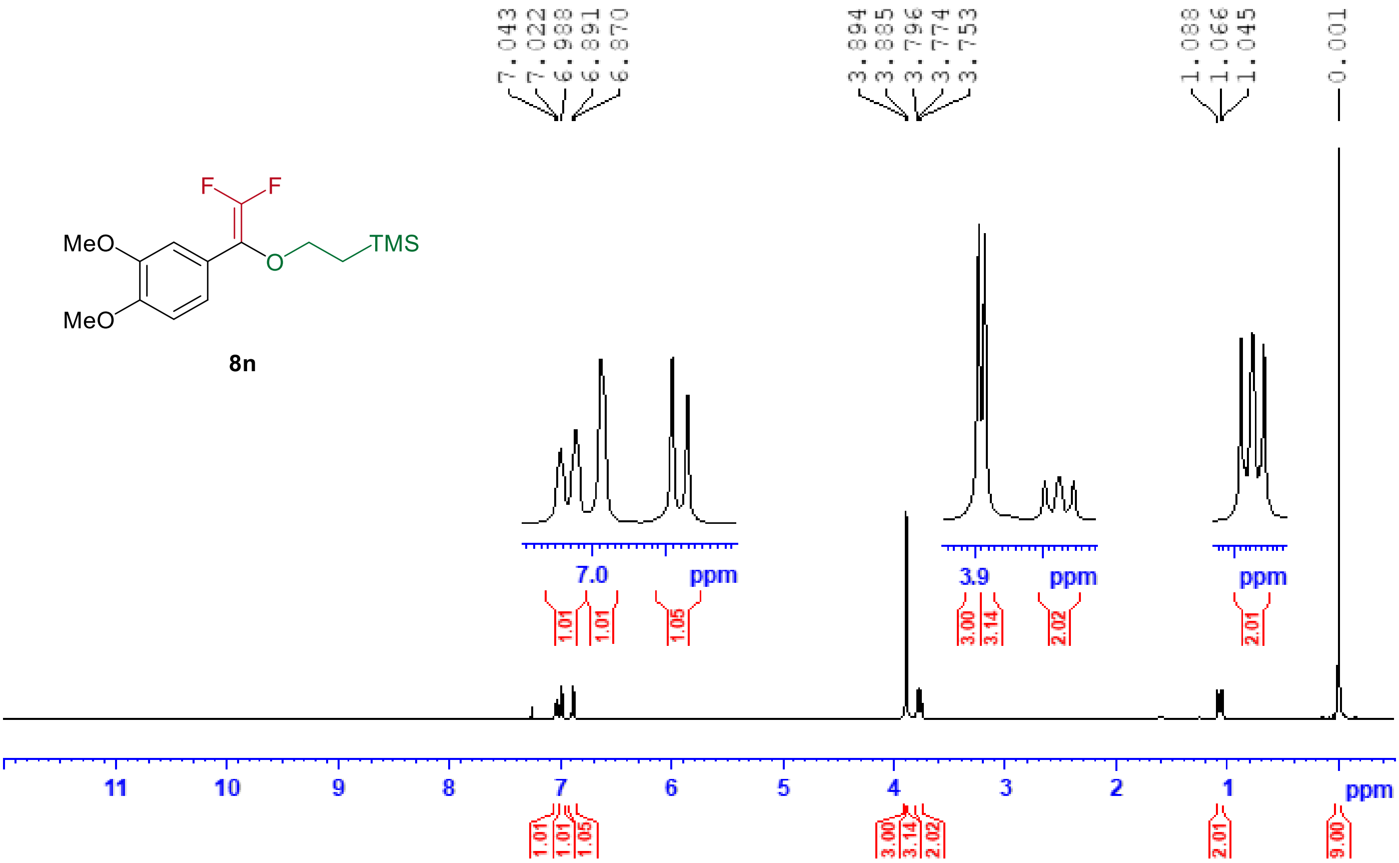

^{13}C NMR of **8n**

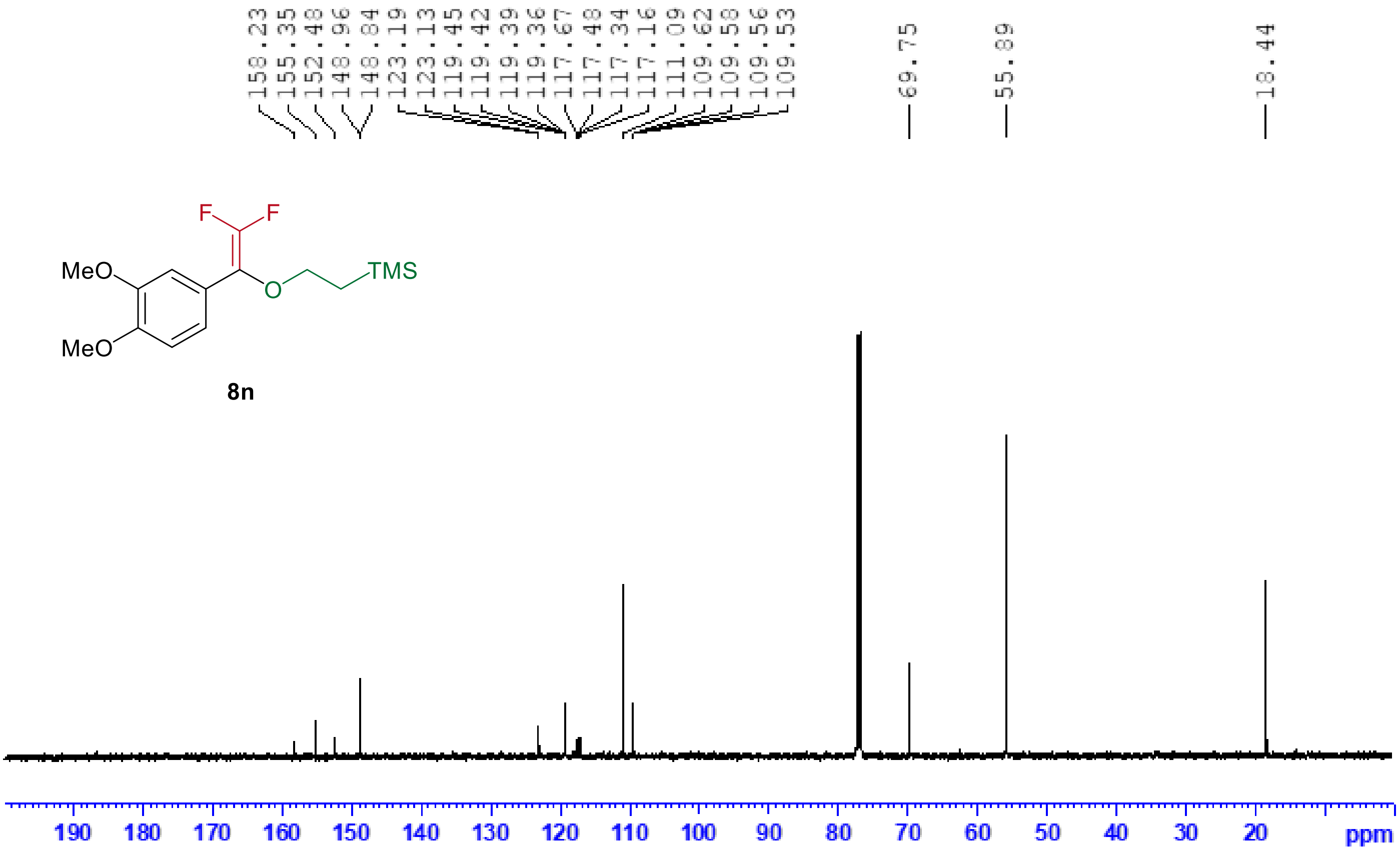

^{19}F NMR of **8n**

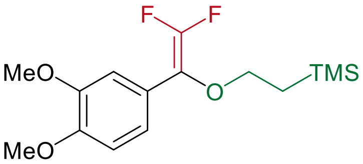

8n

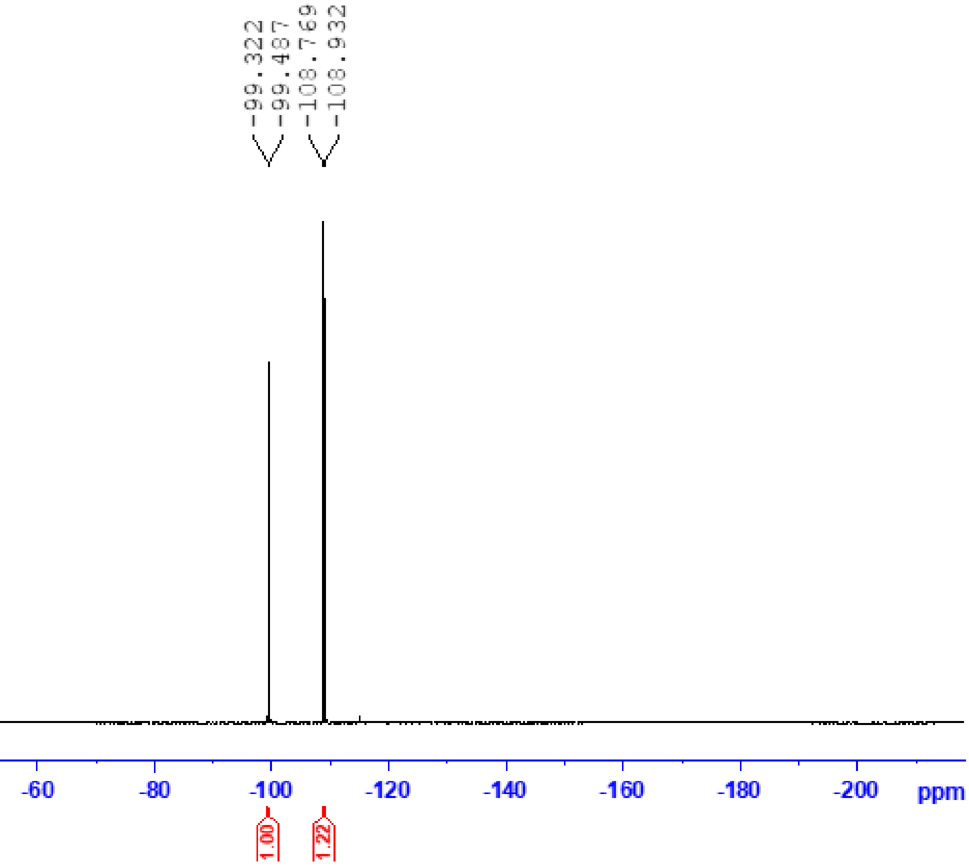

^{1}H NMR of **8o**

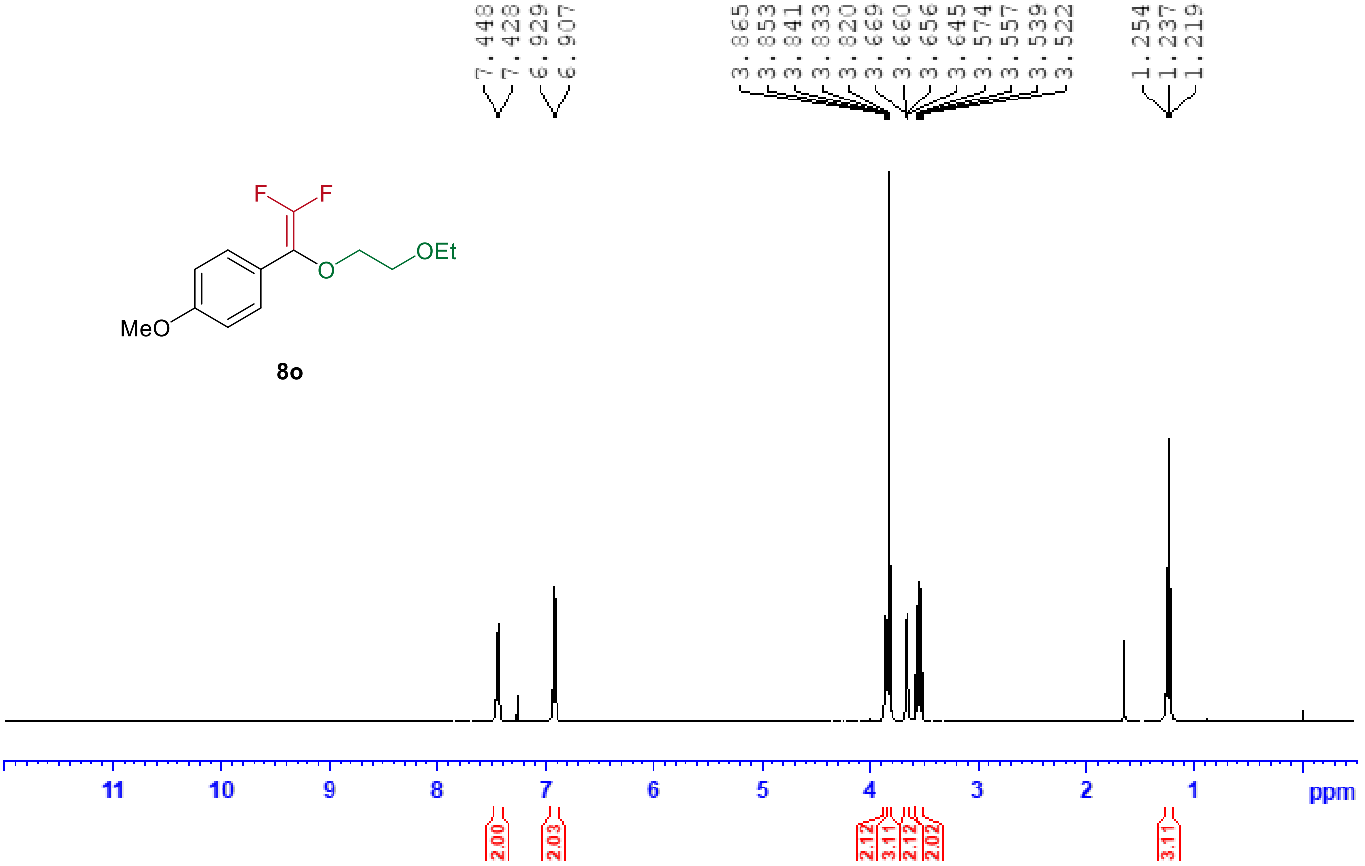

^{13}C NMR of **8o**

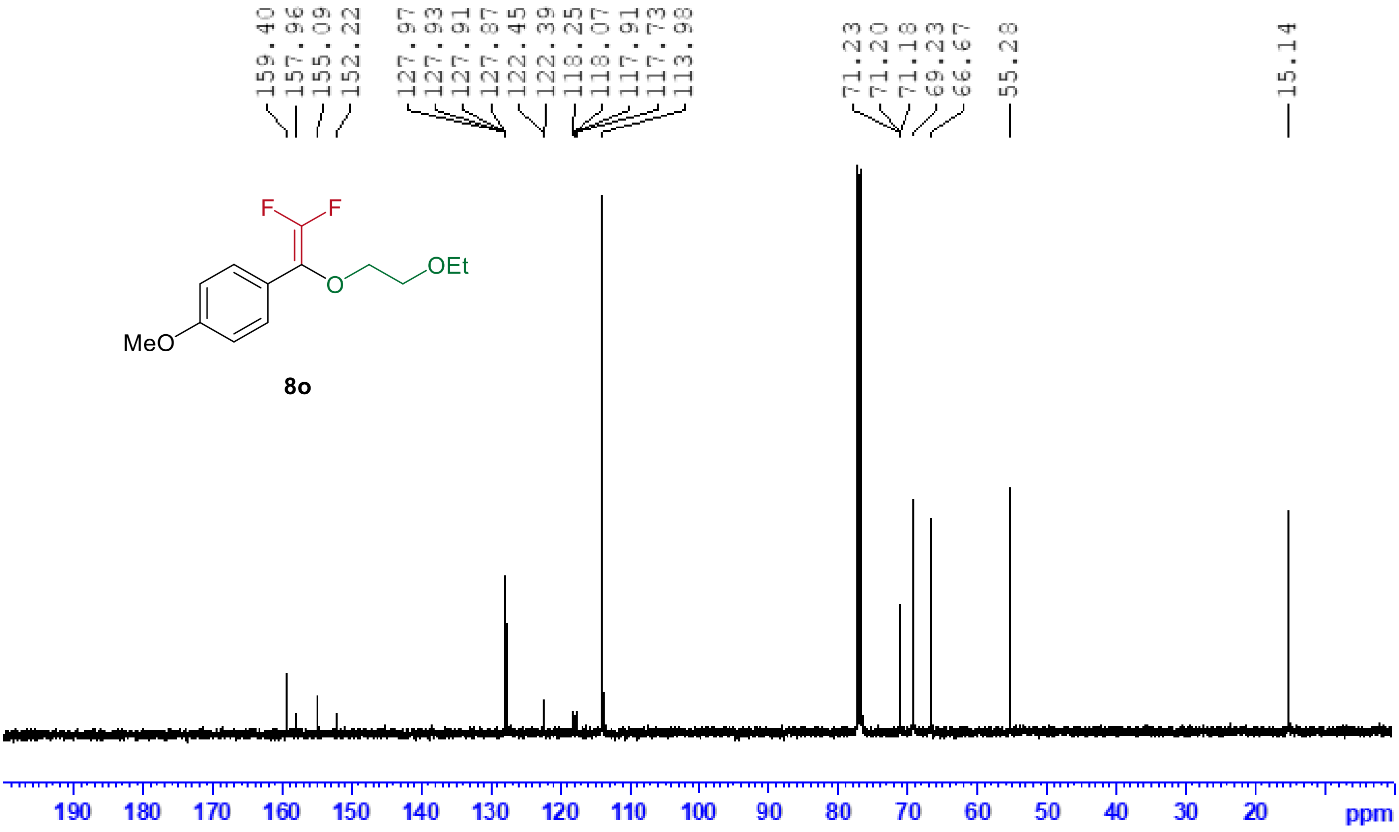

^{19}F NMR of **8o**

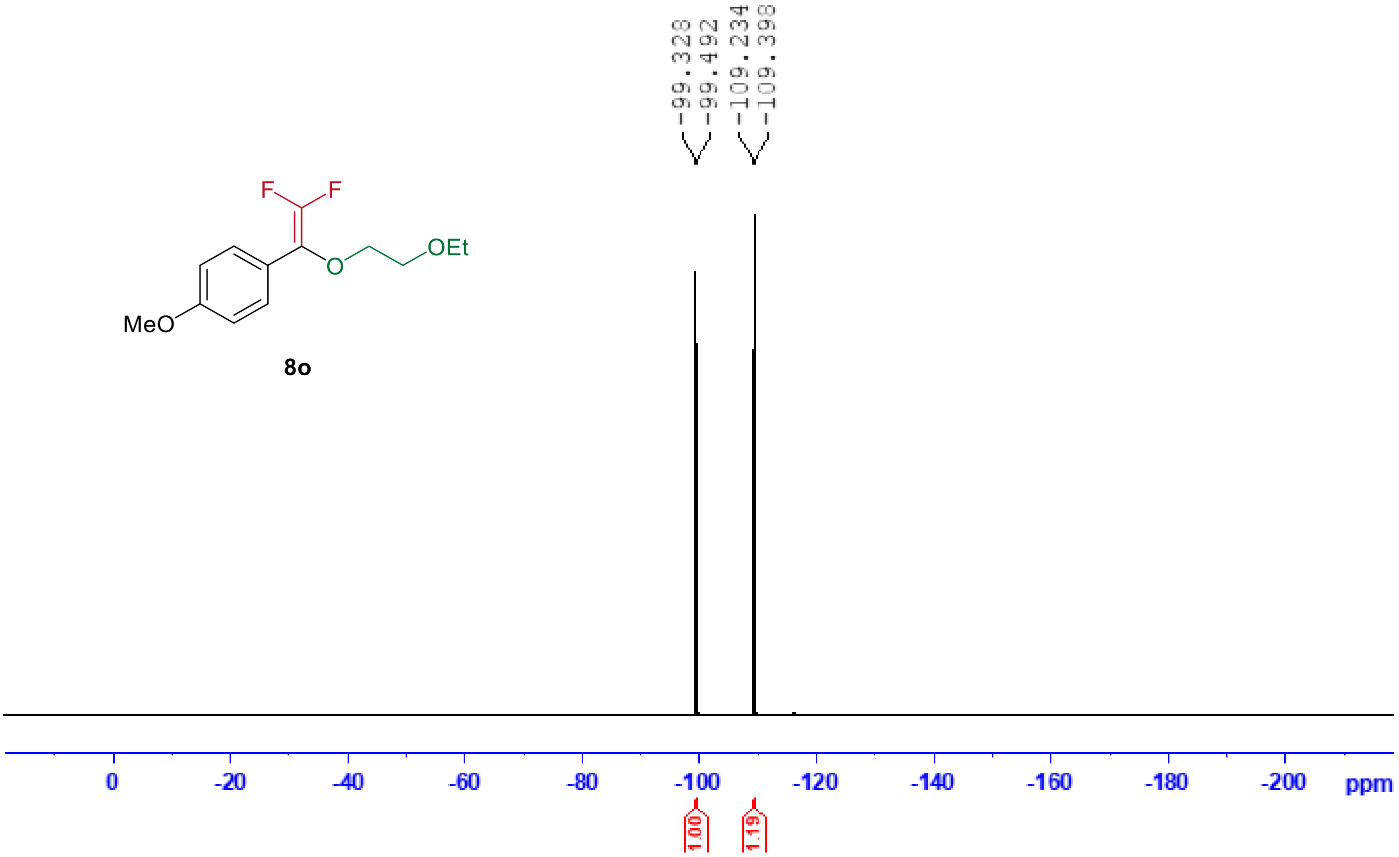
F
F
O
OEt
MeO
8o
-99.328
-99.492
-109.234
-109.398
0
-20
-40
-60
-80
-100
-120
-140
-160
-180
-200
ppm
1.00
1.19

^{1}H NMR of **8p**

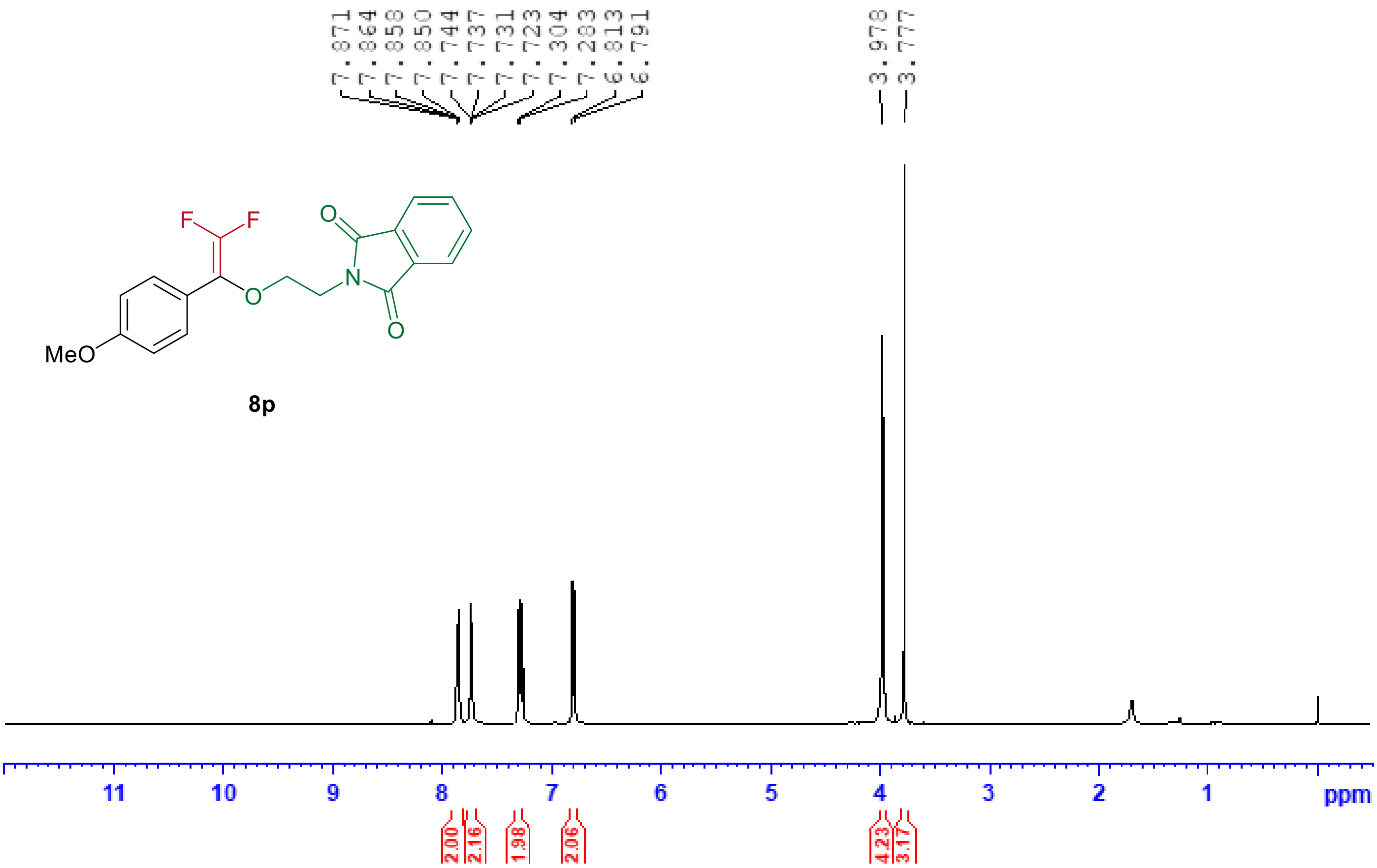

^{13}C NMR of **8p**

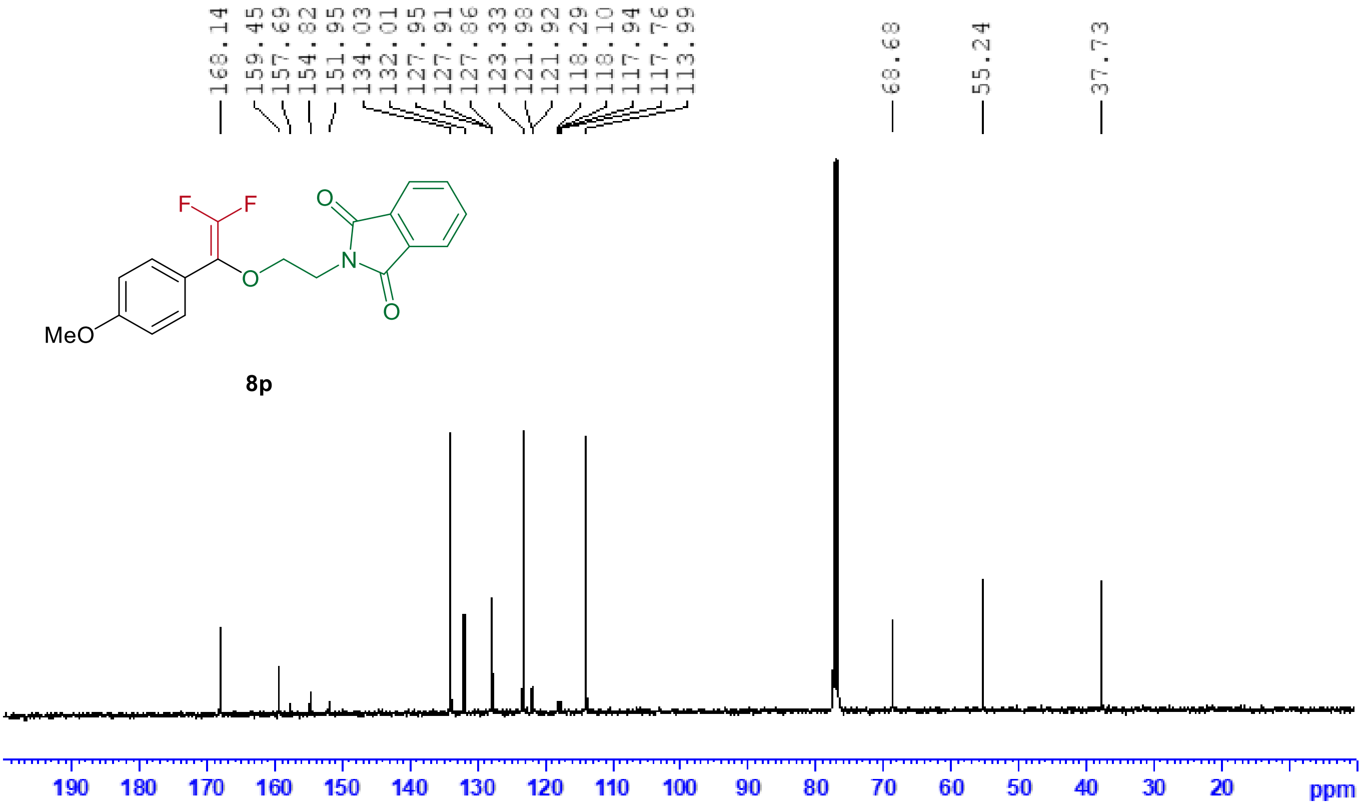

^{19}F NMR of **8p**

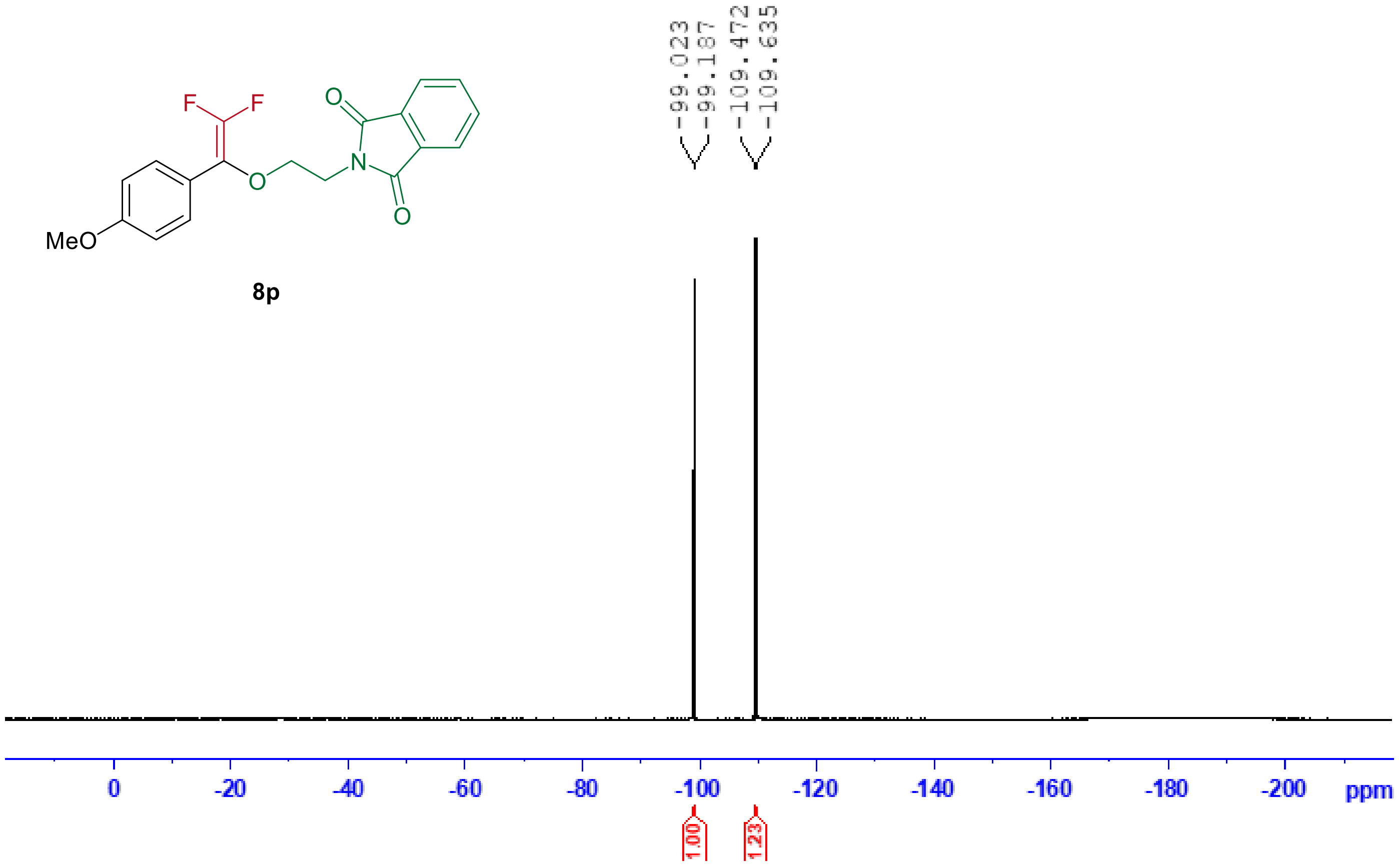

MeO
F
F
O
O
N
O
8p
-99.023
-99.187
-109.472
-109.635
0
-20
-40
-60
-80
-100
-120
-140
-160
-180
-200
ppm
1.00
1.23

^{1}H NMR of **8q**

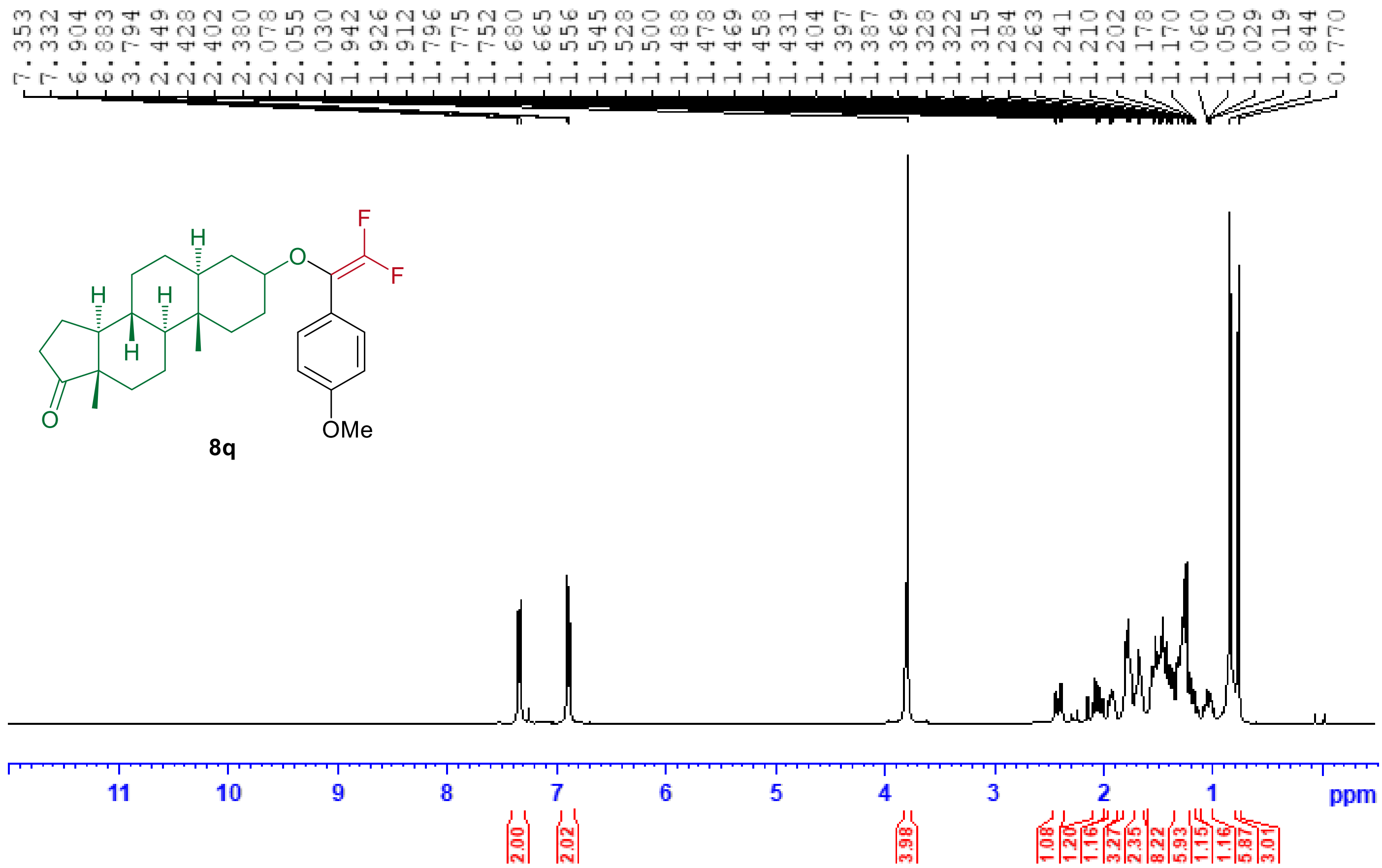

^{13}C NMR of **8q**

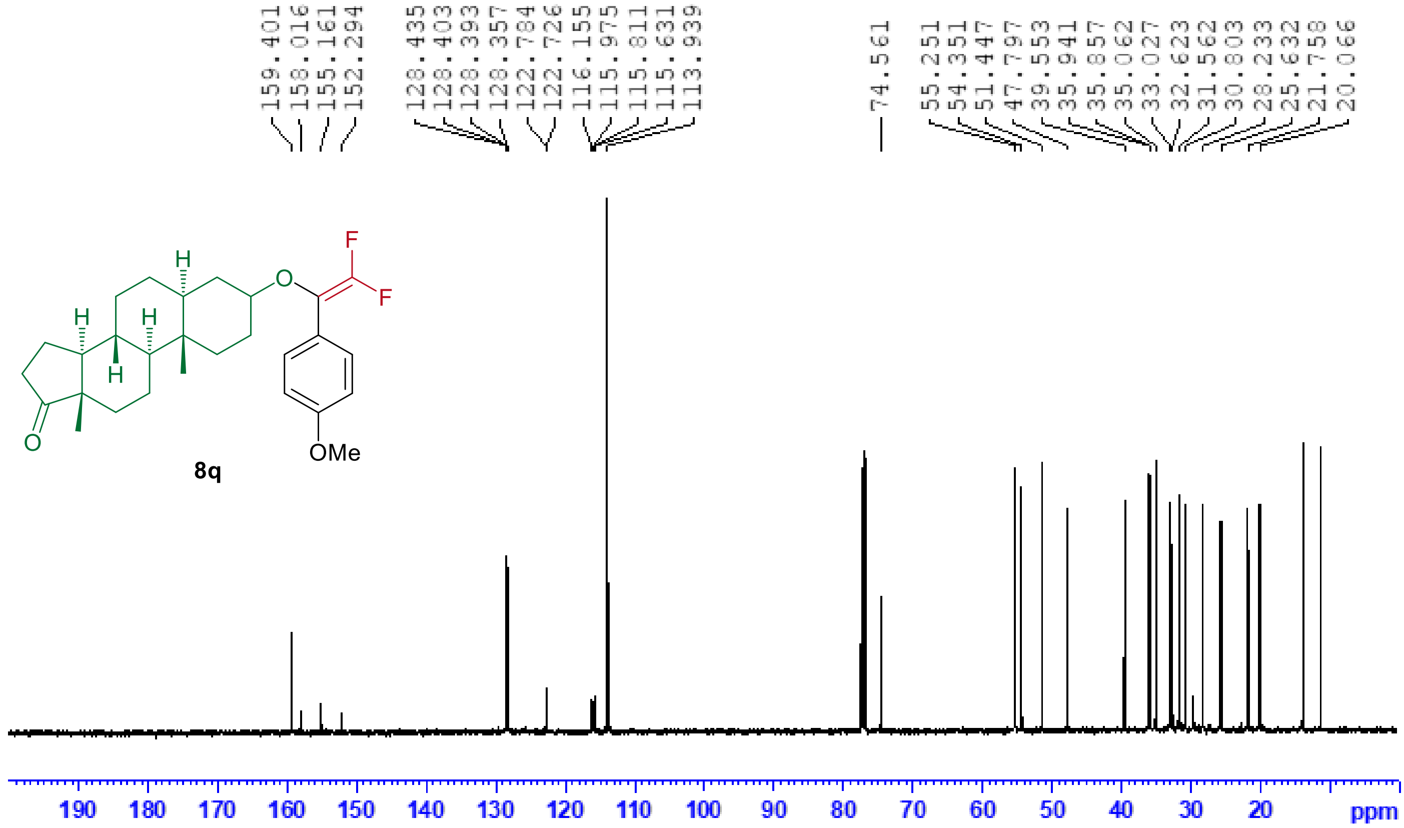

^{19}F NMR of **8q**

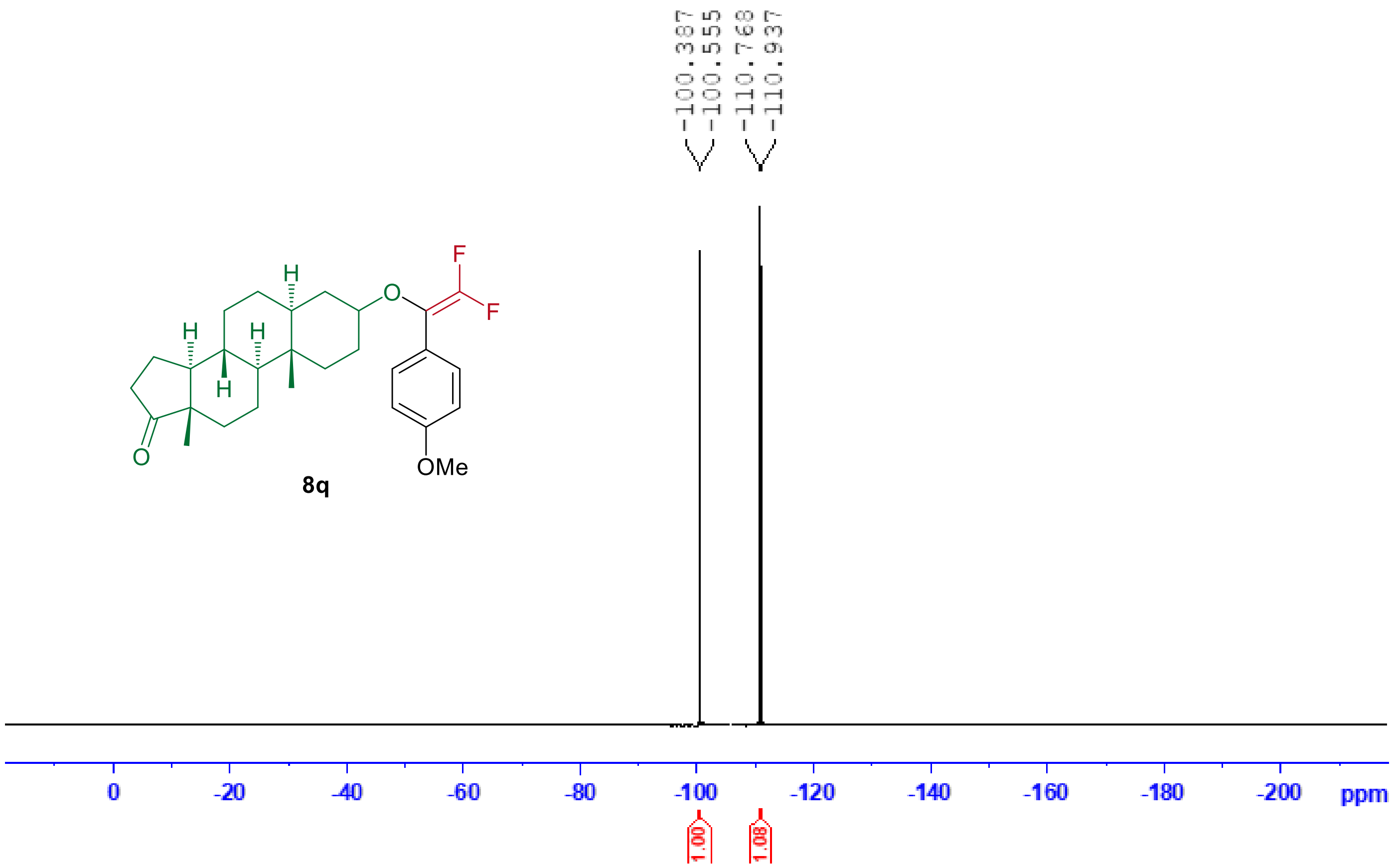
H
H
H
H
O
O
F
F
OMe
8q
-100.387
-100.555
-110.768
-110.937
0
-20
-40
-60
-80
-100
-120
-140
-160
-180
-200
ppm
1.00
1.08

^{1}H NMR of **8r**

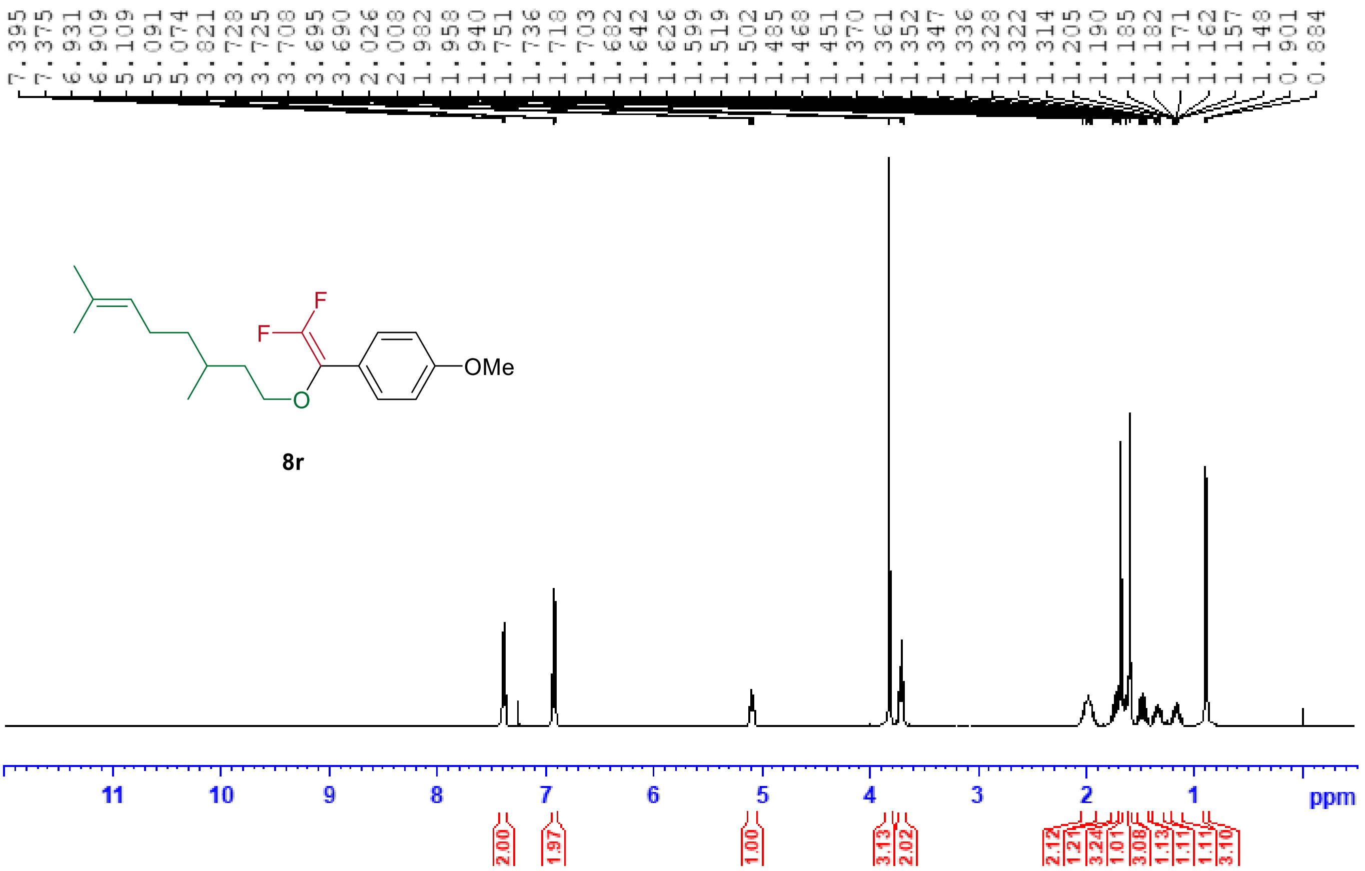

^{13}C NMR of **8r**

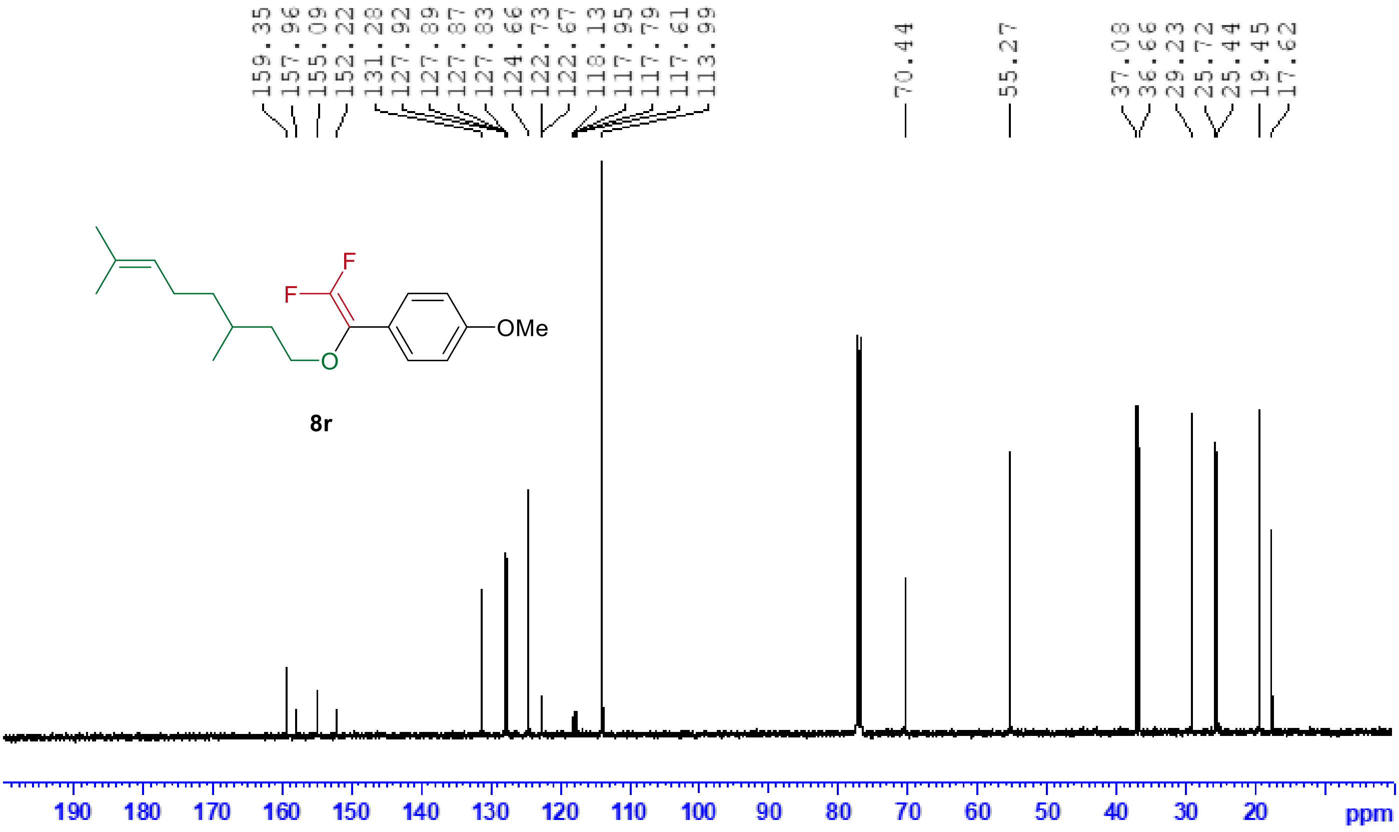

^{19}F NMR of **8r**

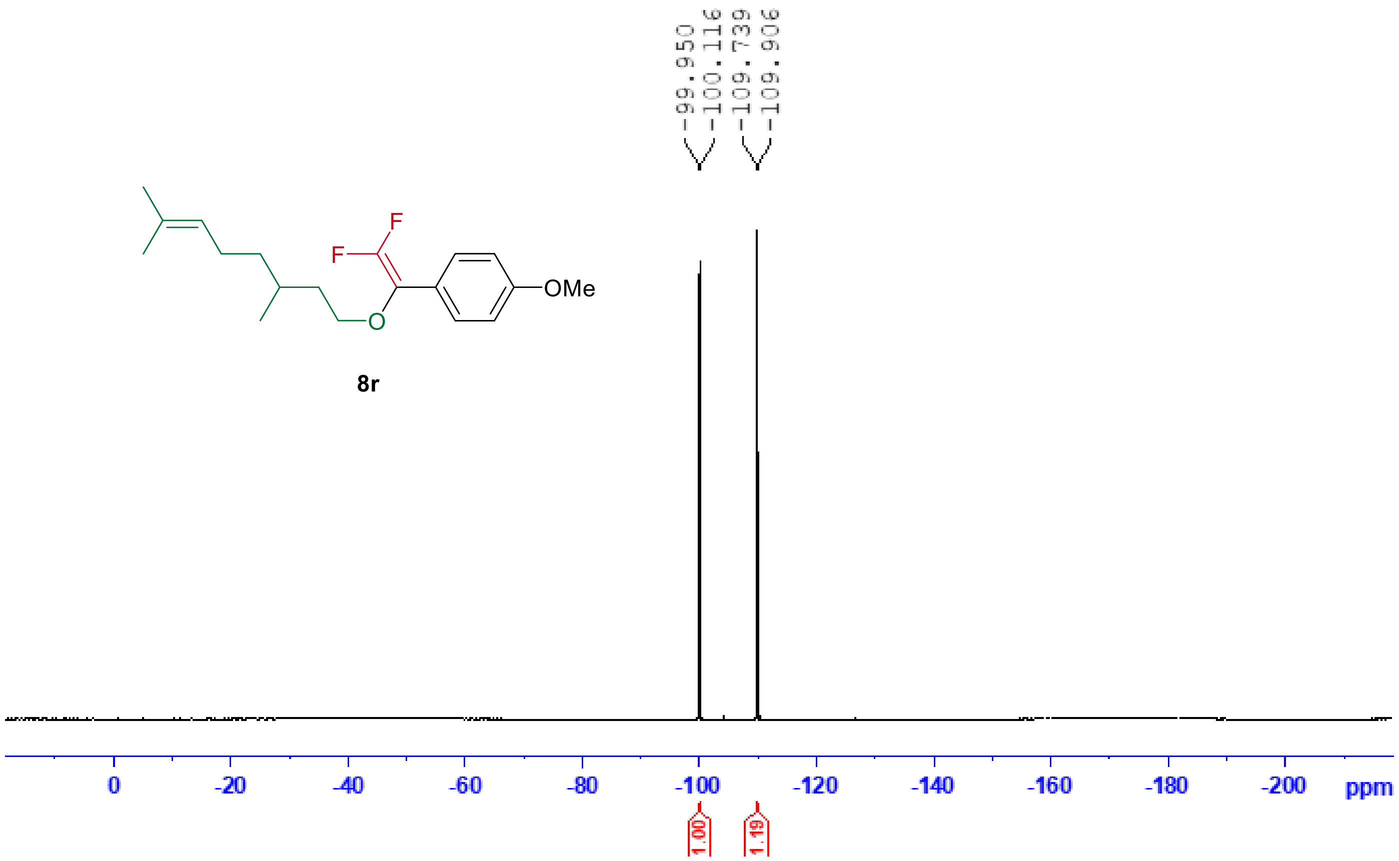
-99.950
-100.116
-109.739
-109.906
F
F
O
OMe
8r
0
-20
-40
-60
-80
-100
-120
-140
-160
-180
-200
ppm
1.00
1.19

^{1}H NMR of **8s**

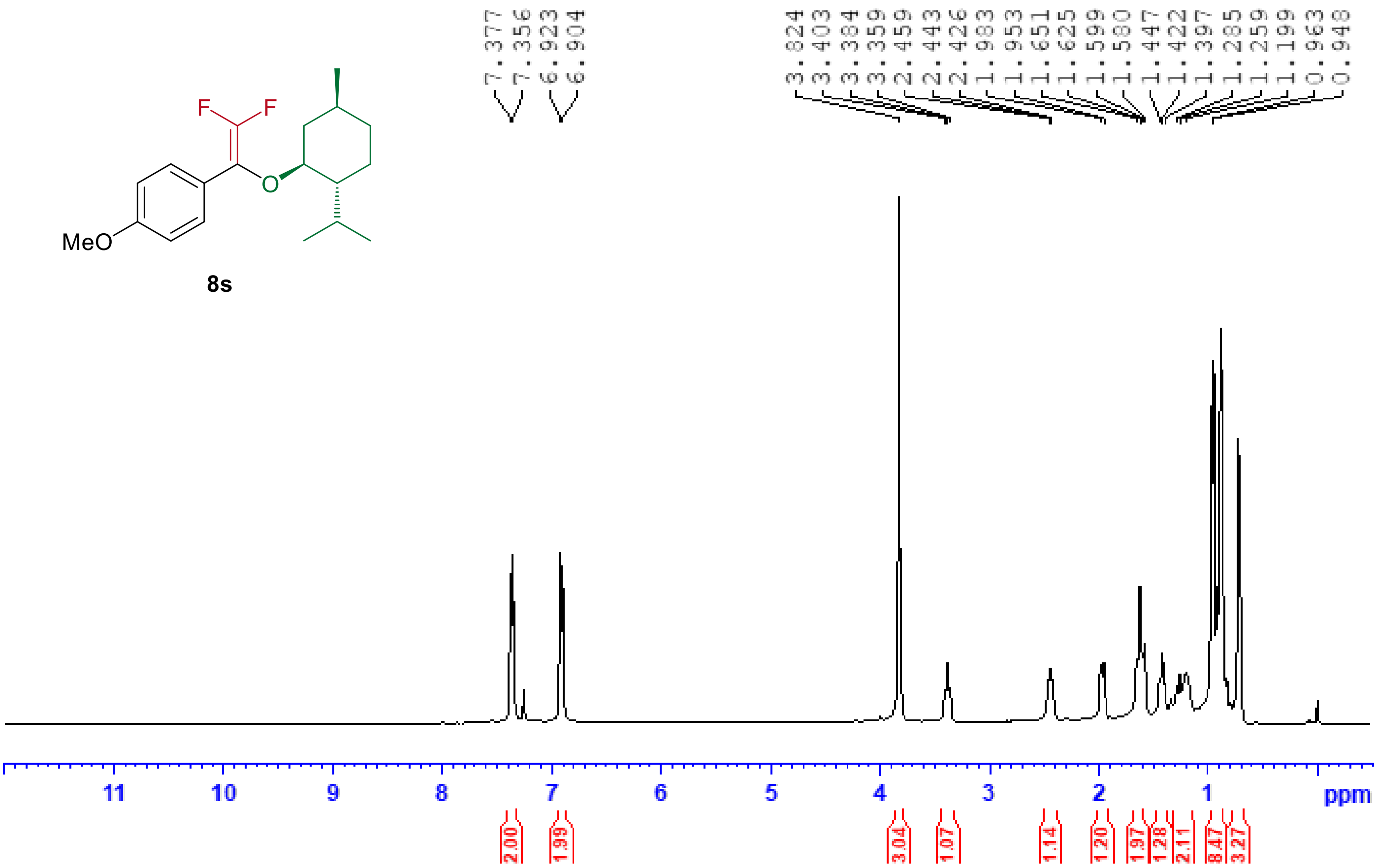

^{13}C NMR of **8s**

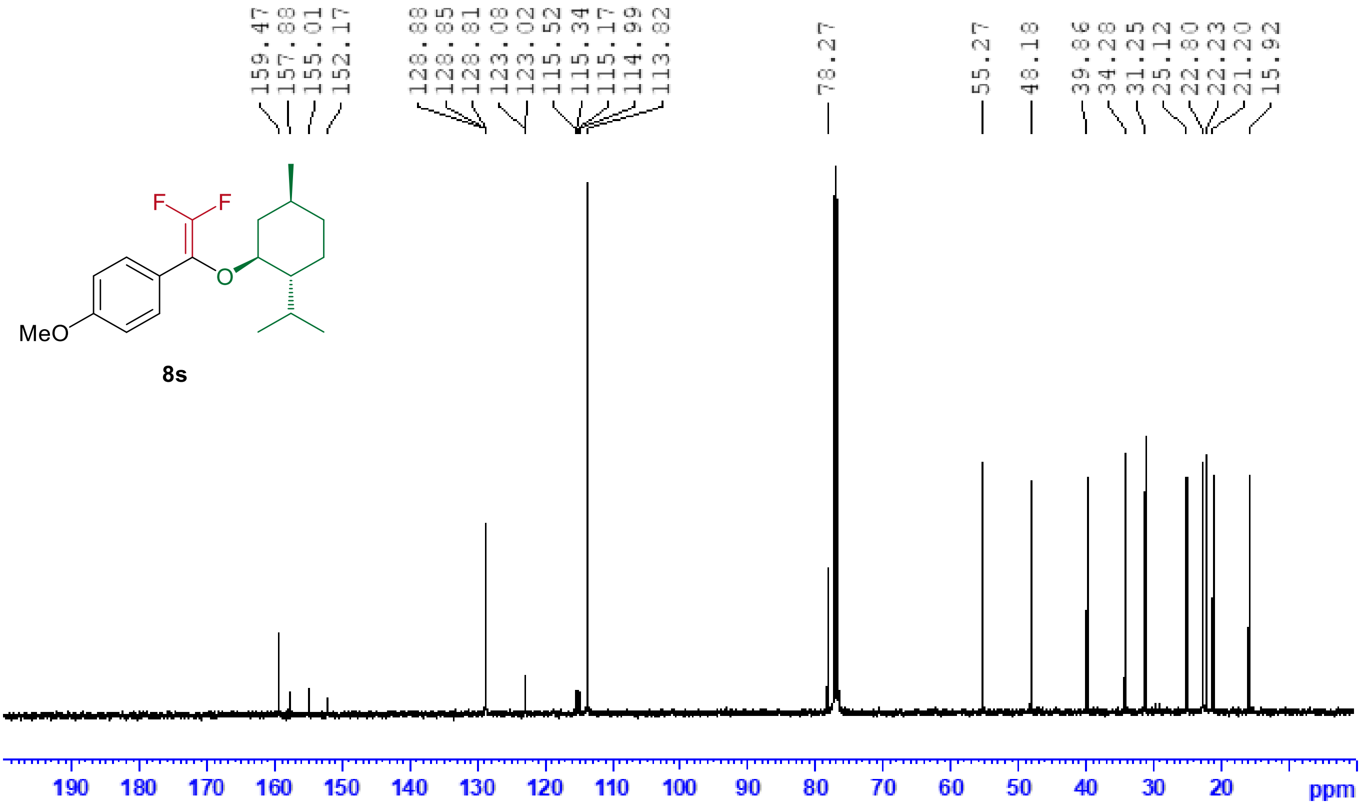

^{19}F NMR of **8s**

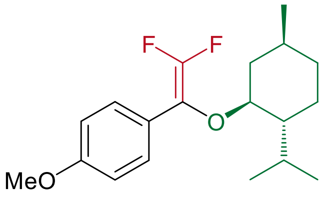

8s

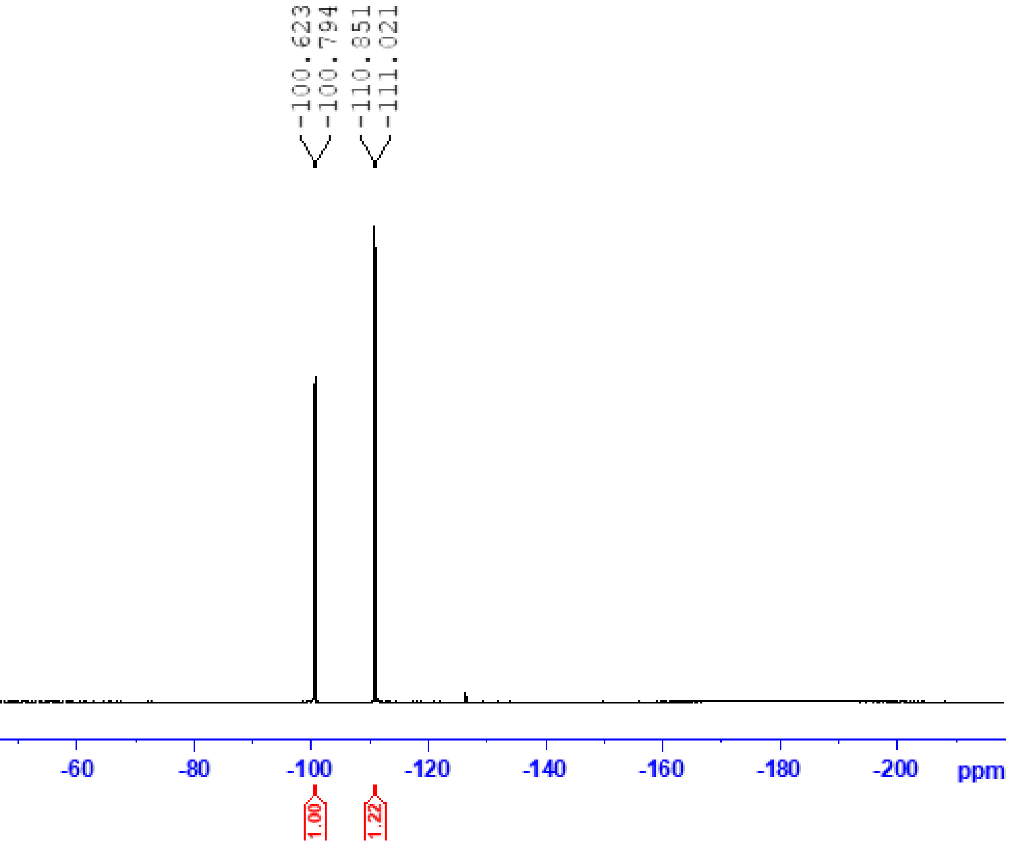

^{1}H NMR of **9a**

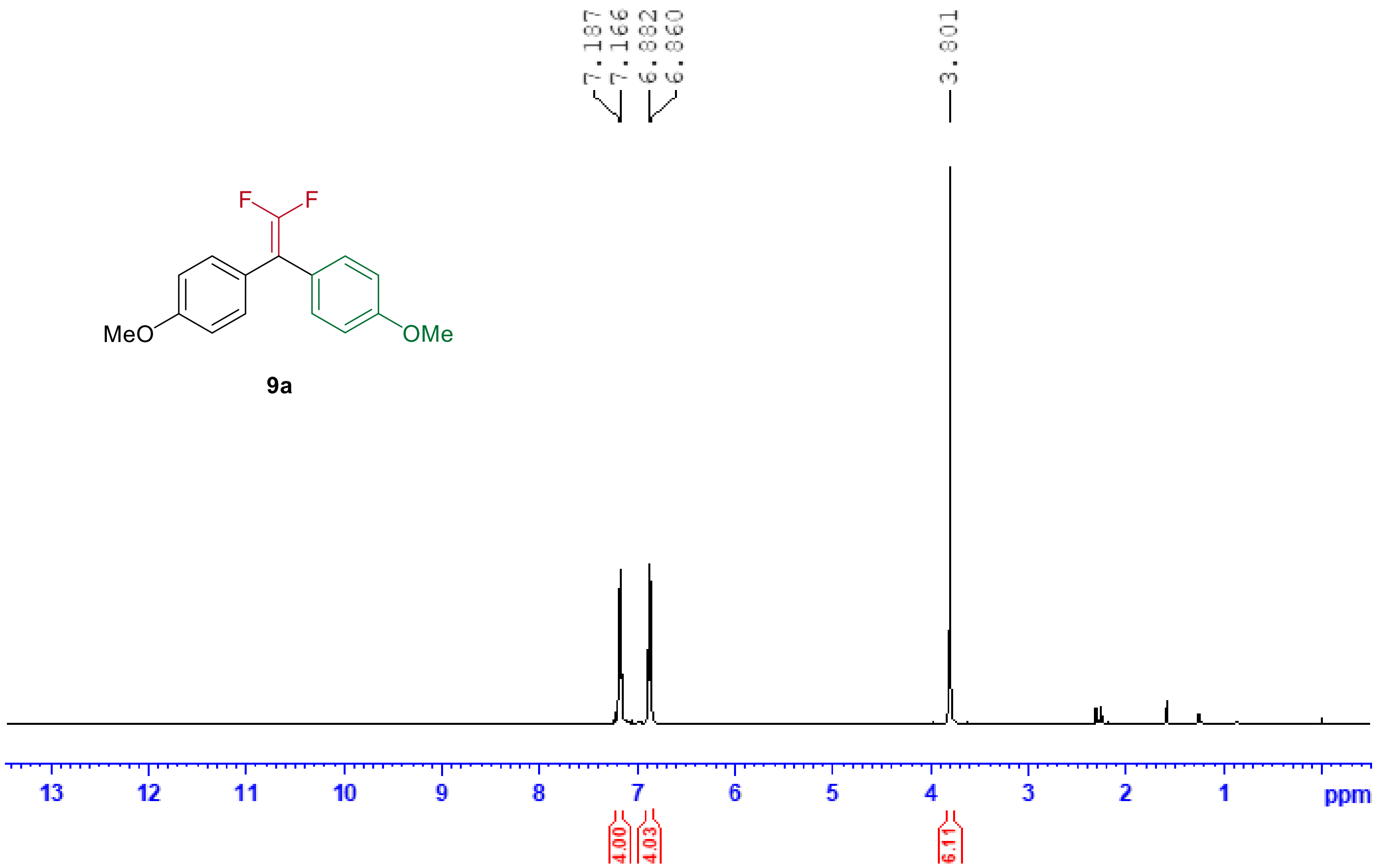

^{13}C NMR of **9a**

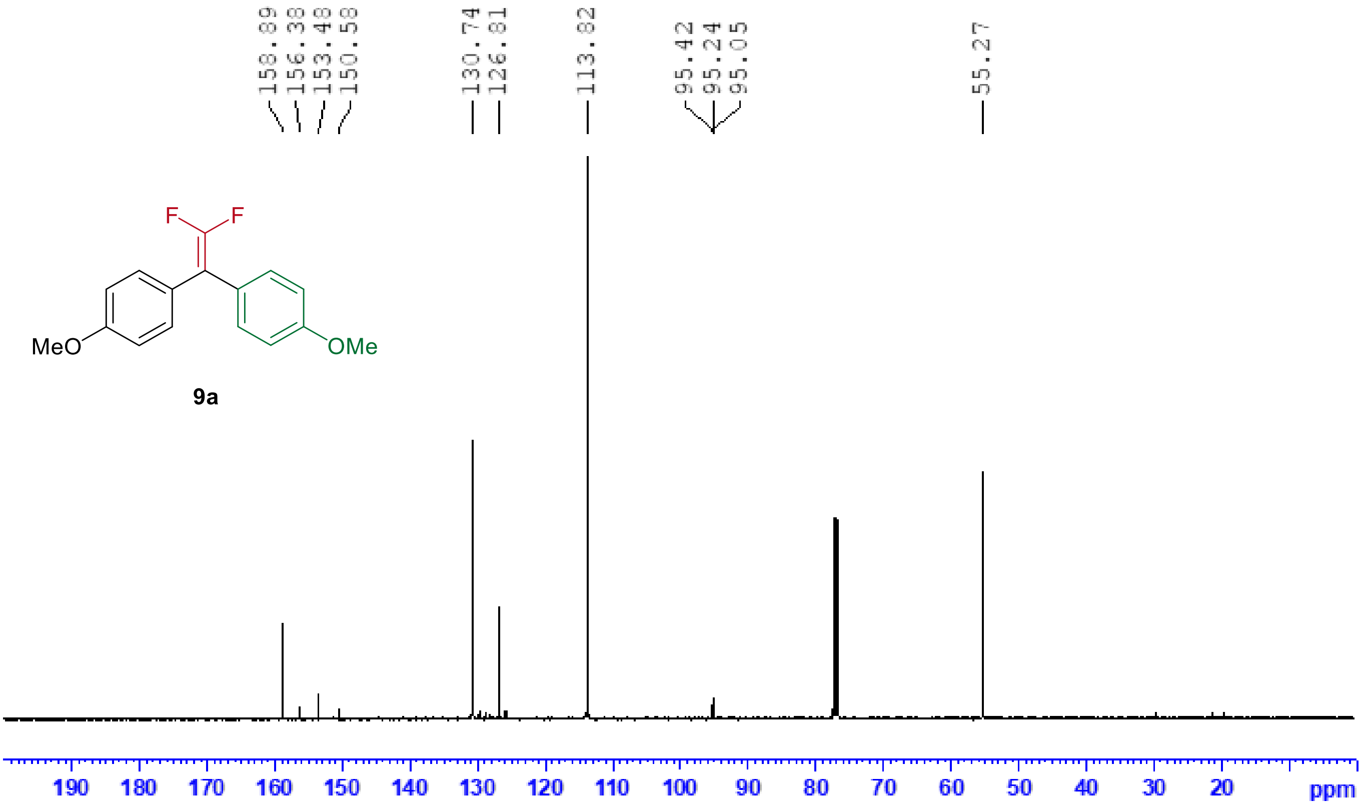

^{19}F NMR of **9a**

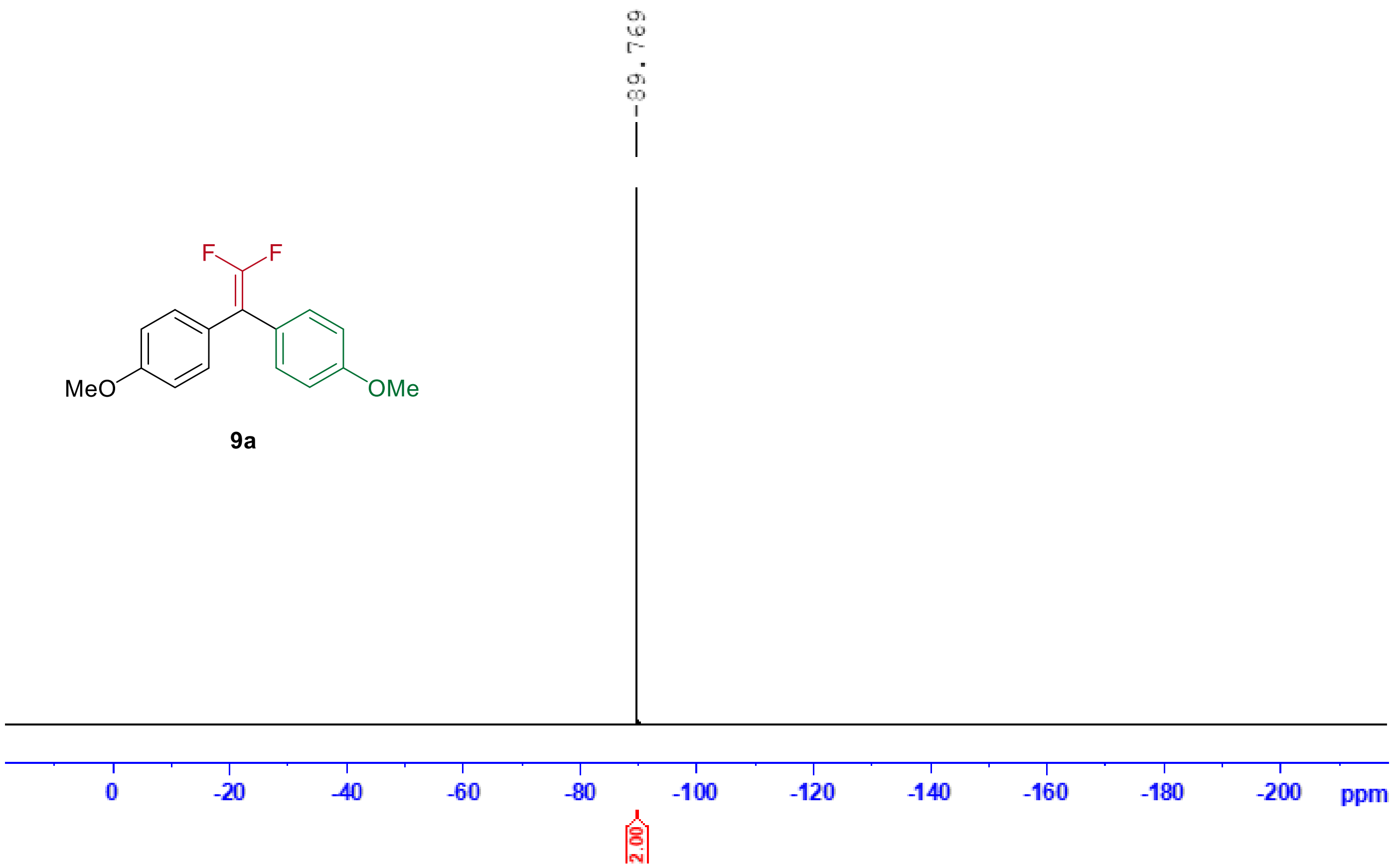

^{1}H NMR of **9b**

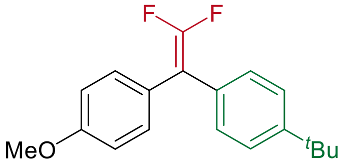

9b

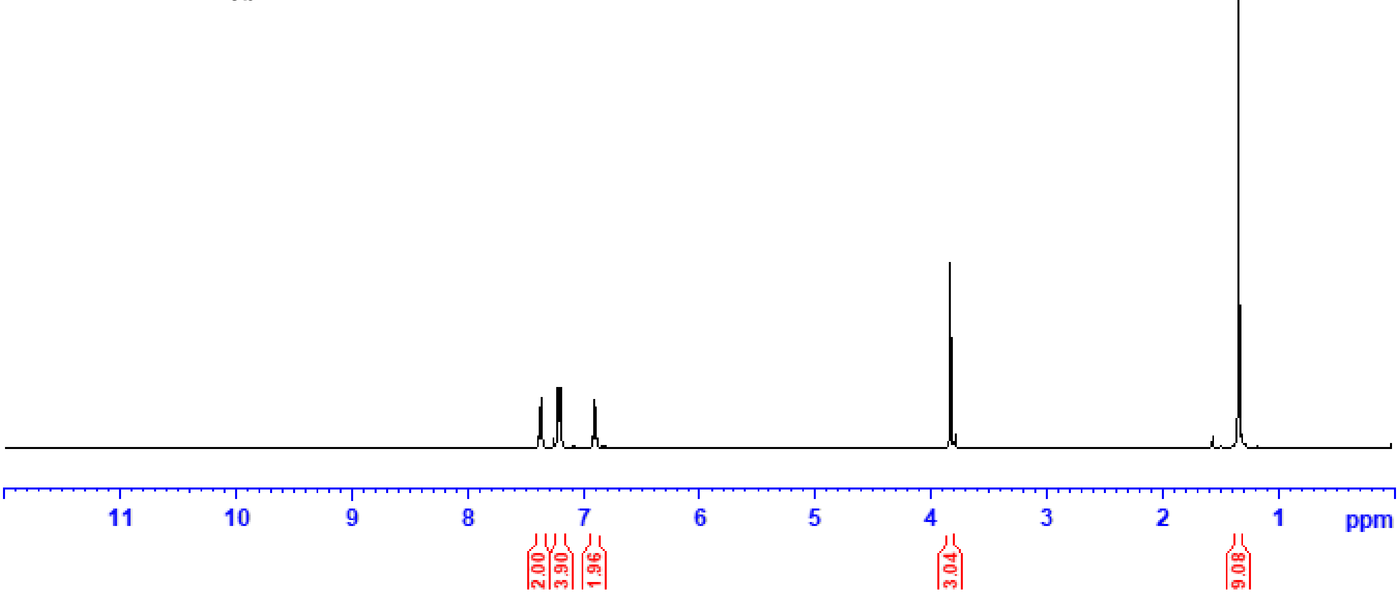

^{13}C NMR of **9b**

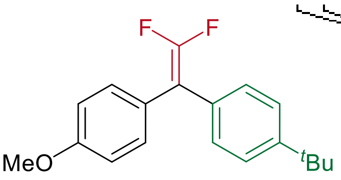

9b

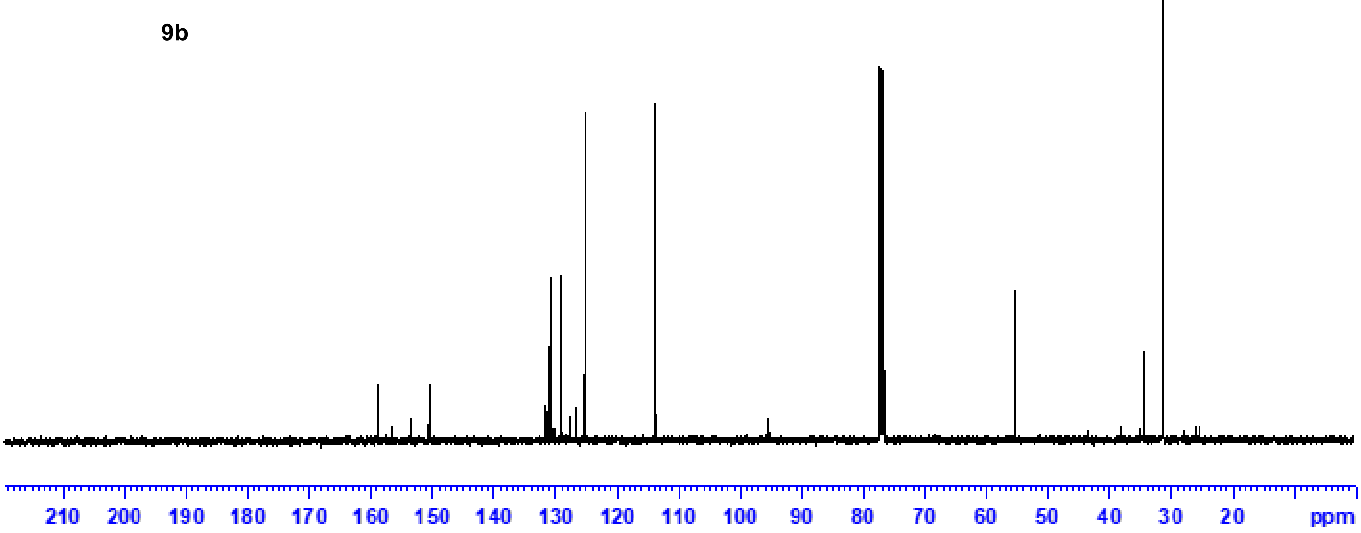

^{19}F NMR of **9b**

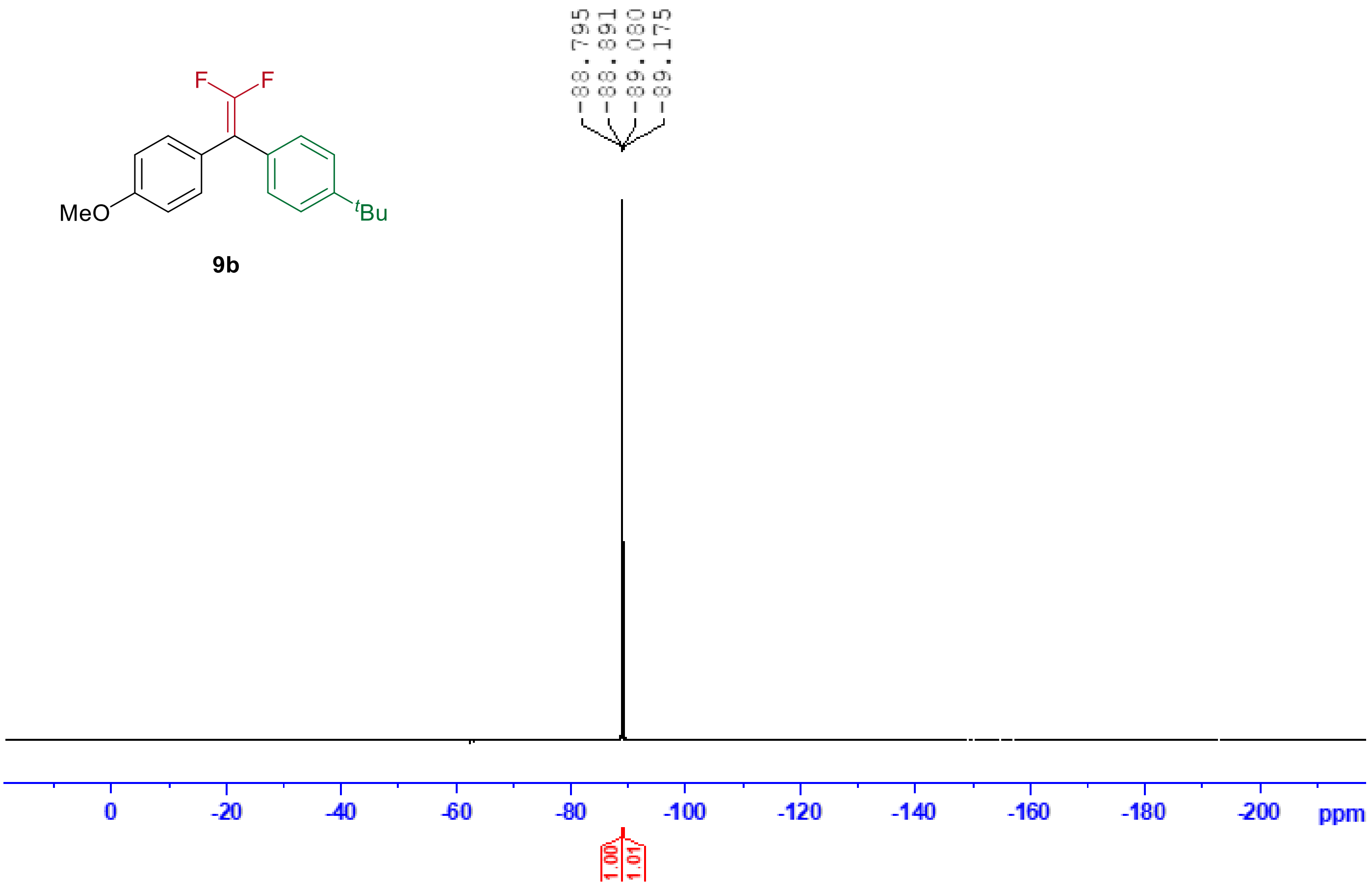
F
F
MeO
tBu
9b
-88.795
-88.891
-89.080
-89.175
0
-20
-40
-60
-80
-100
-120
-140
-160
-180
-200
ppm
1.00
1.01

^{1}H NMR of **3a′**

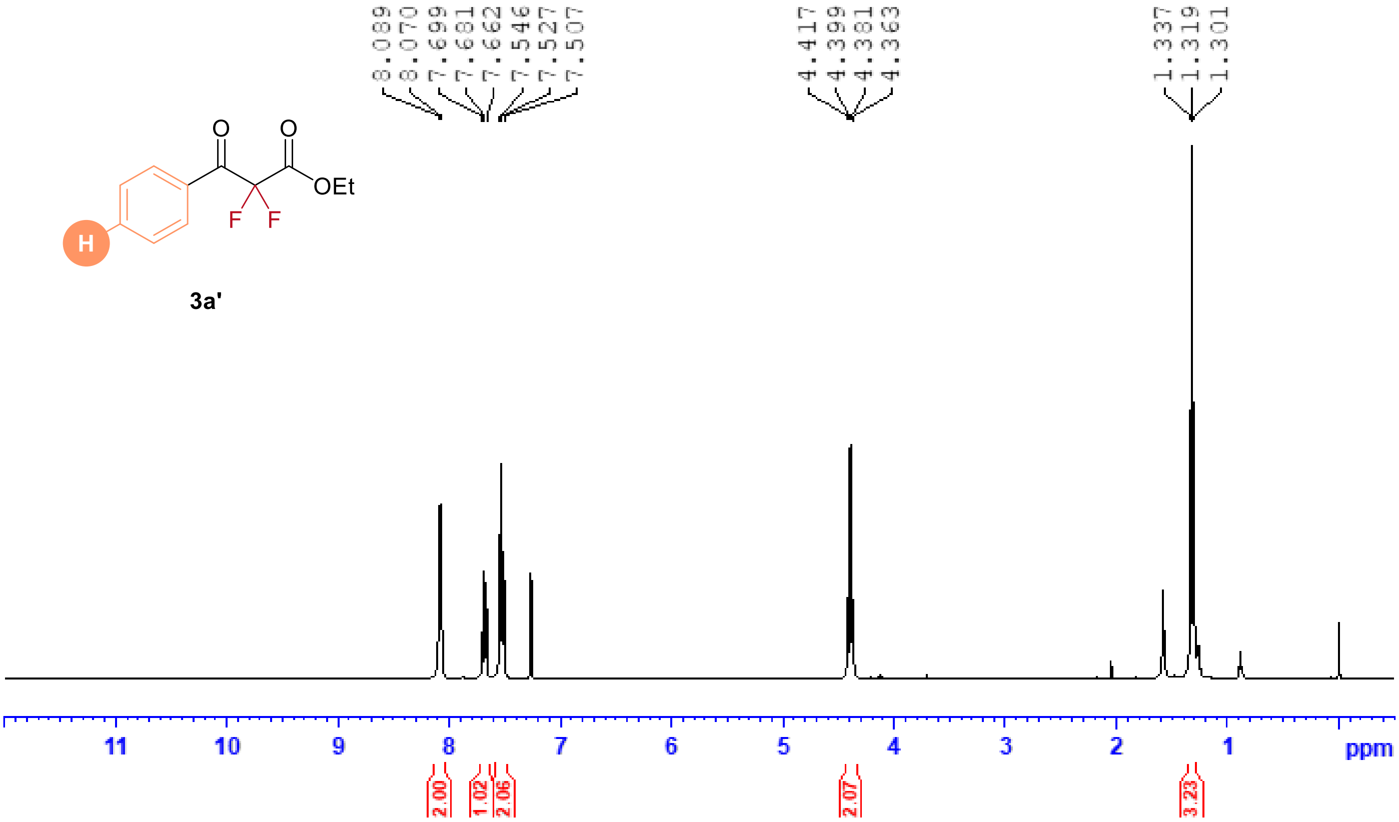

^{13}C NMR of **3a′**

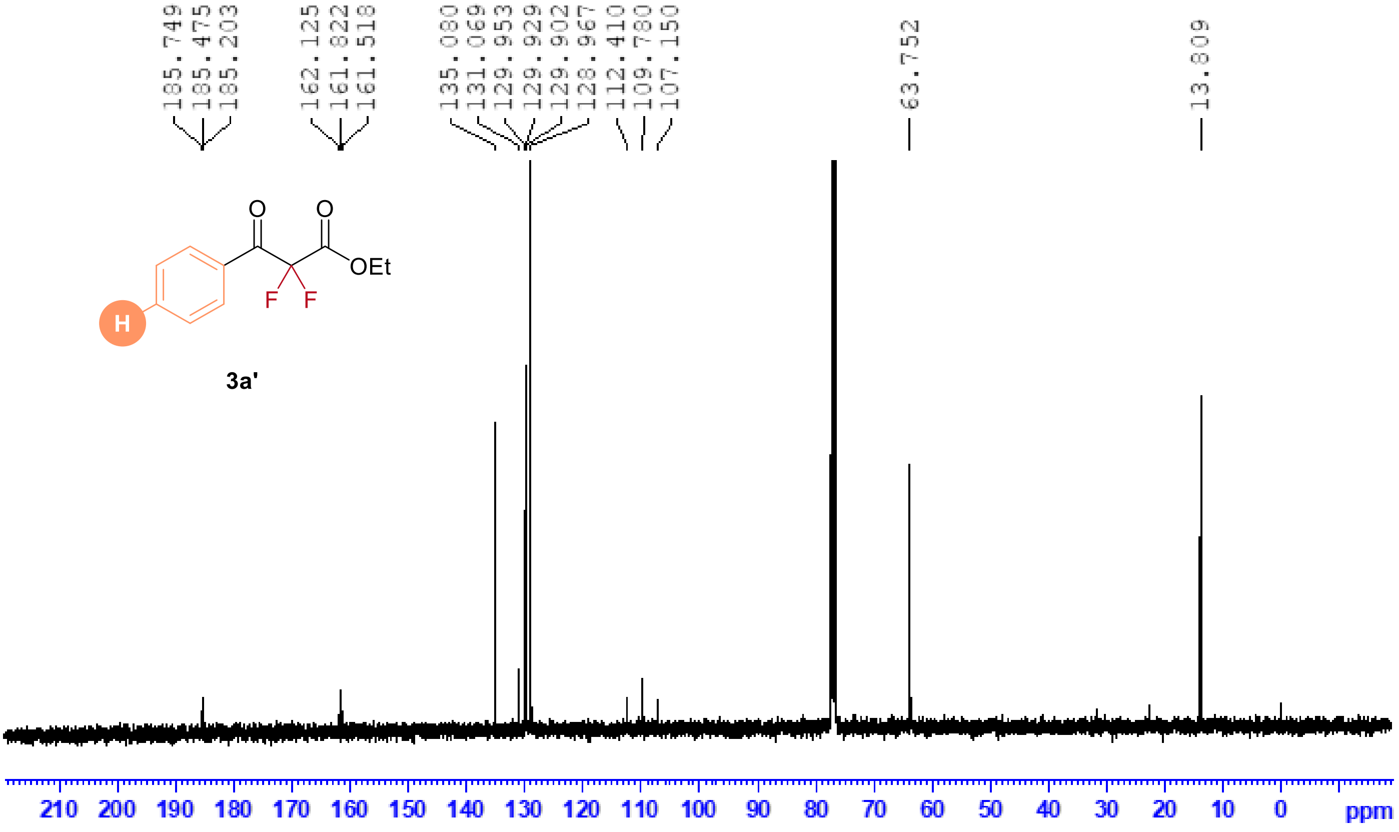

^{19}F NMR of **3a′**

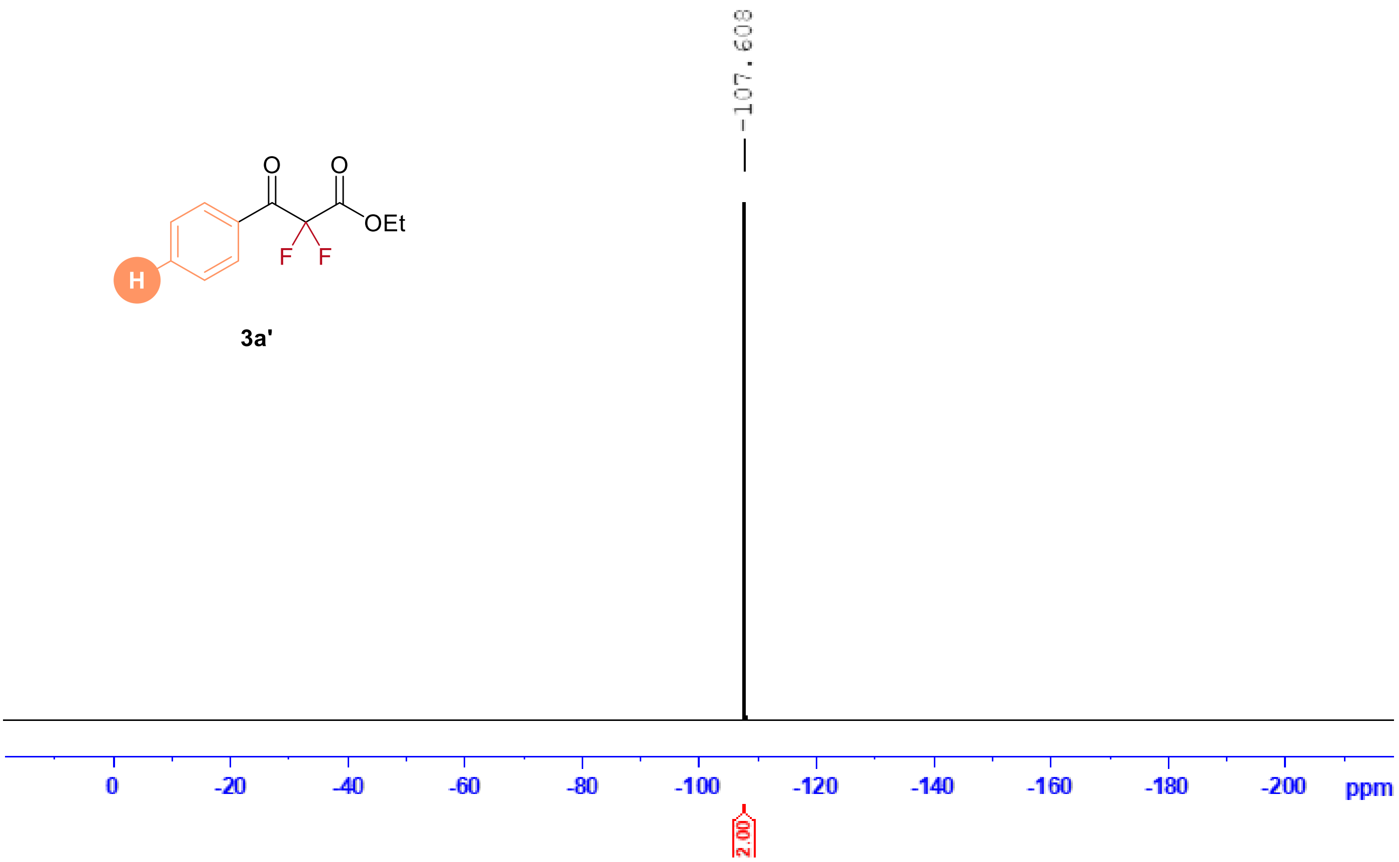

^{1}H NMR of **3a'''**

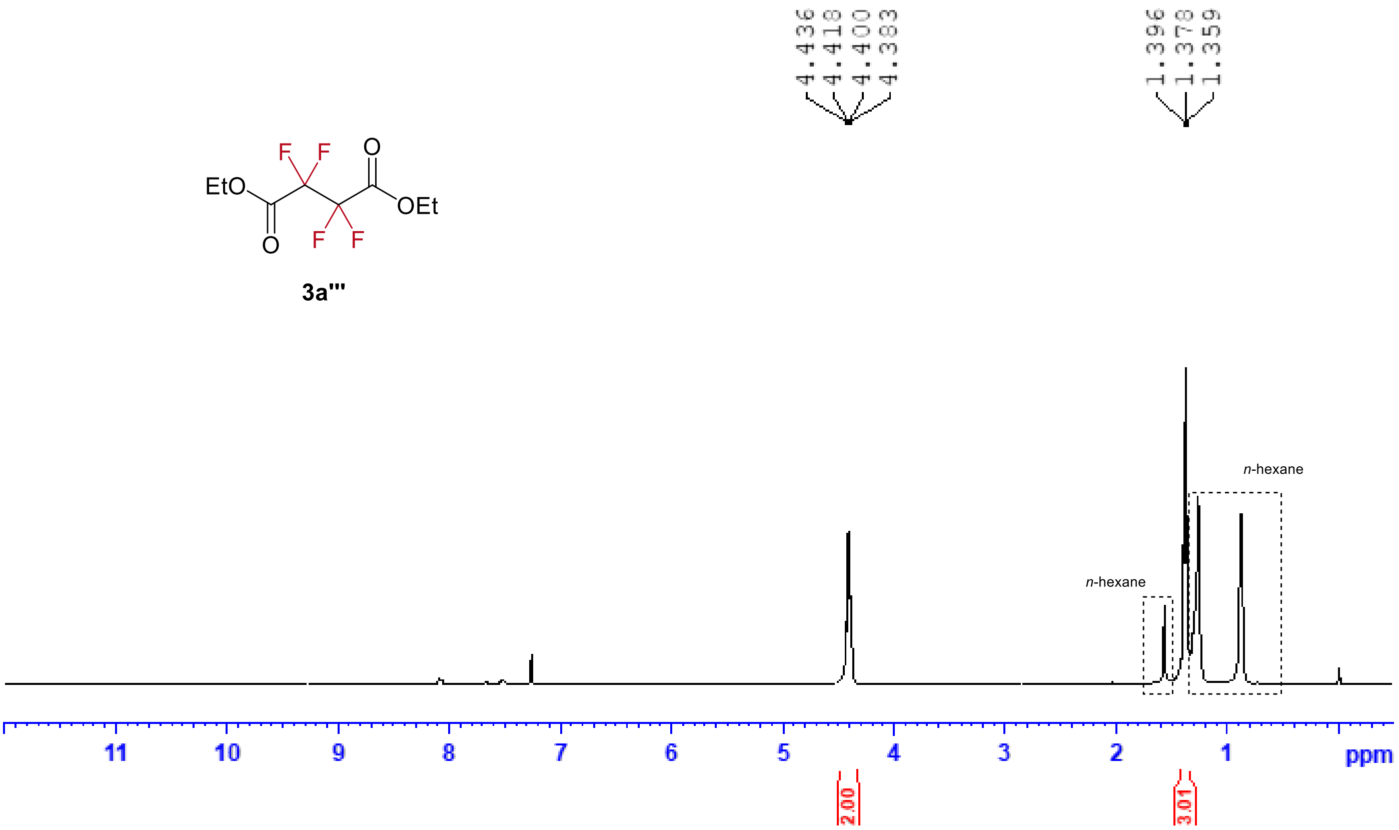

^{13}C NMR of **3a'''**

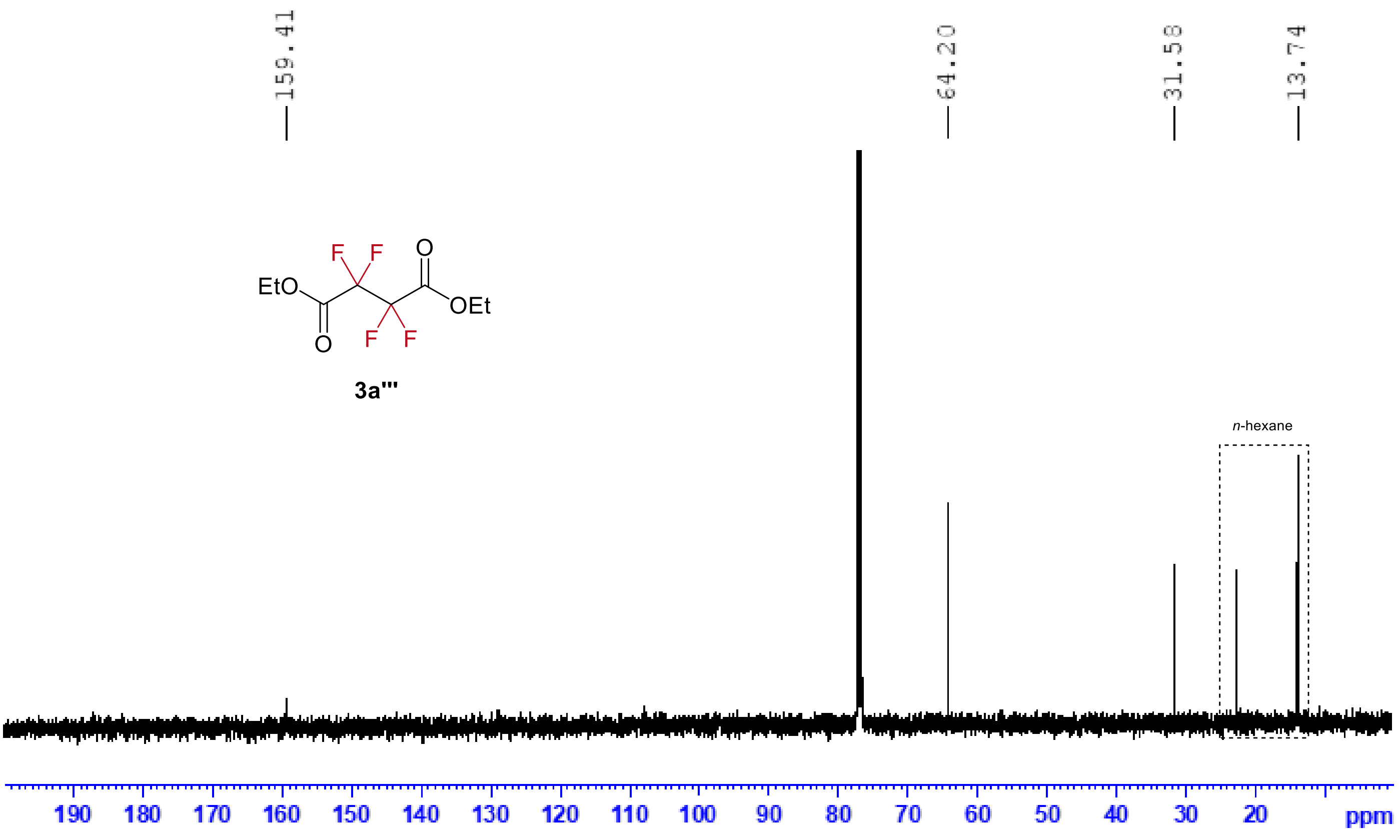

^{19}F NMR of **3a‴**

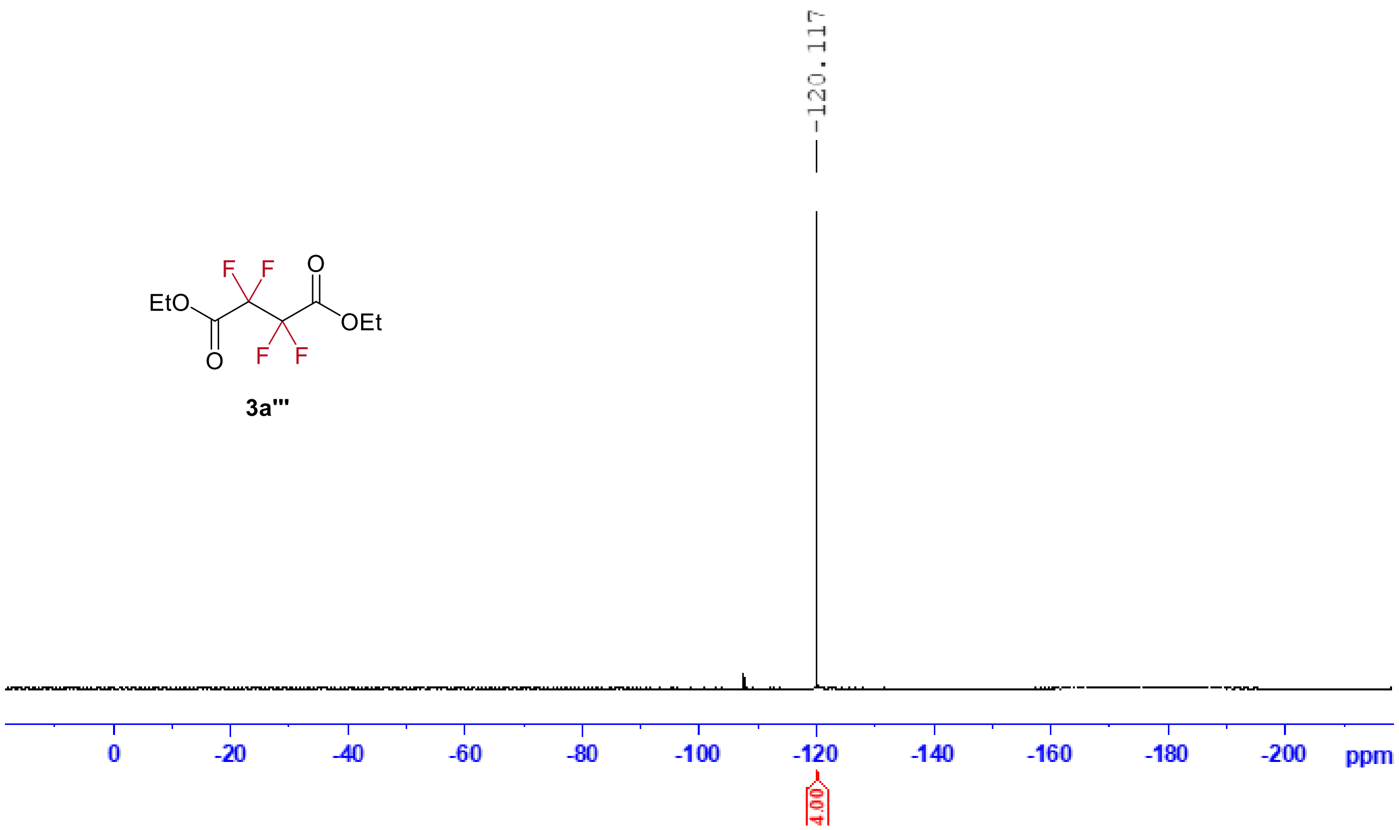

-120.117
F
F
O
EtO
OEt
O
F
F
3a'''
0
-20
-40
-60
-80
-100
-120
-140
-160
-180
-200
ppm
4.00

^{1}H NMR of **3d′**

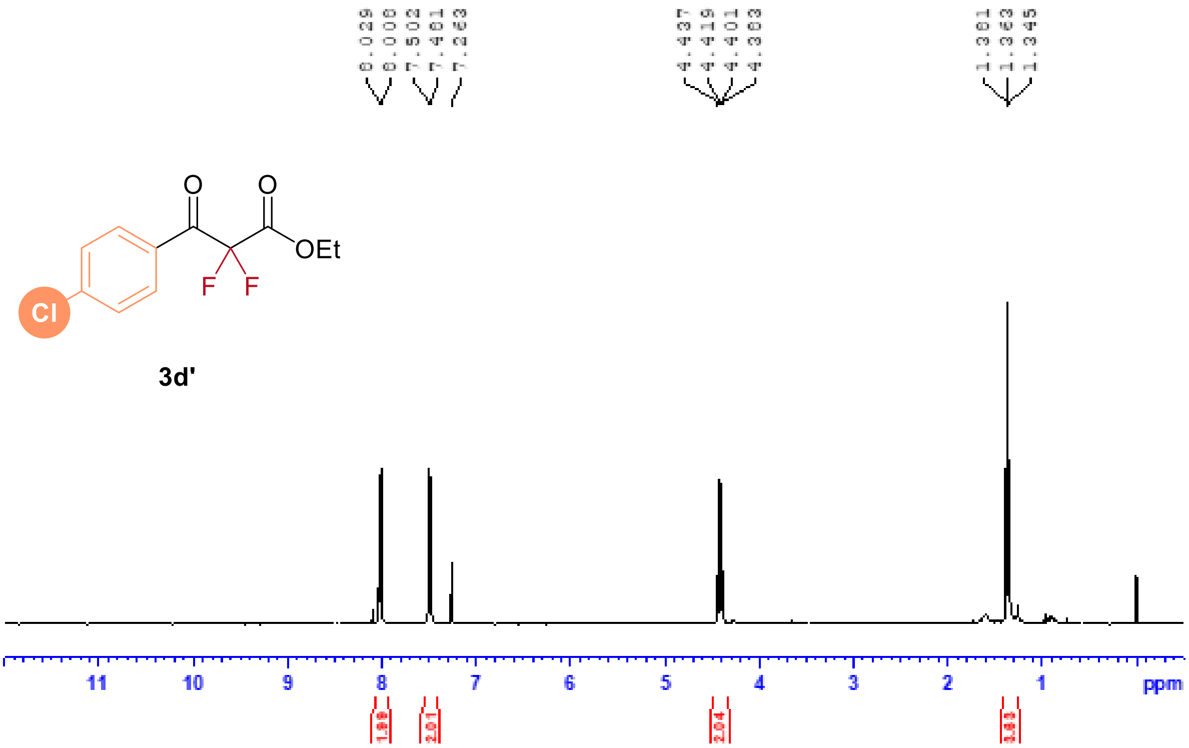

^{13}C NMR of **3d′**

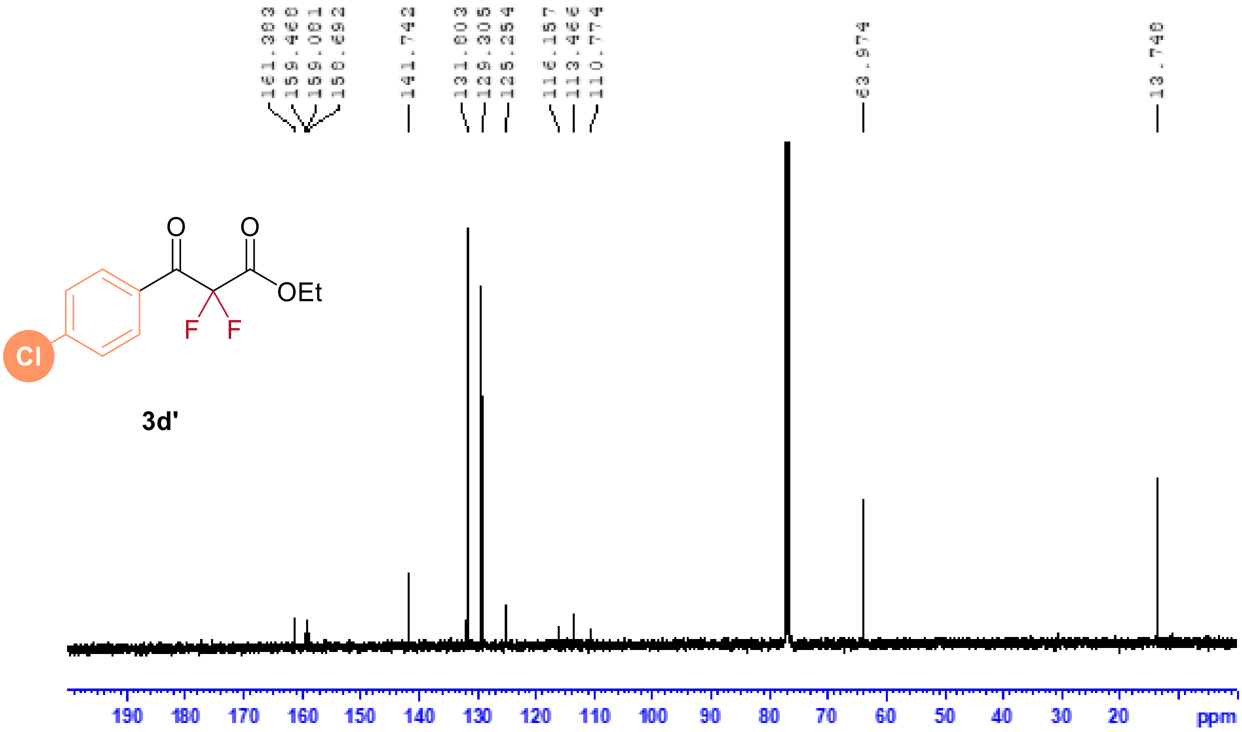

^{19}F NMR of **3d′**

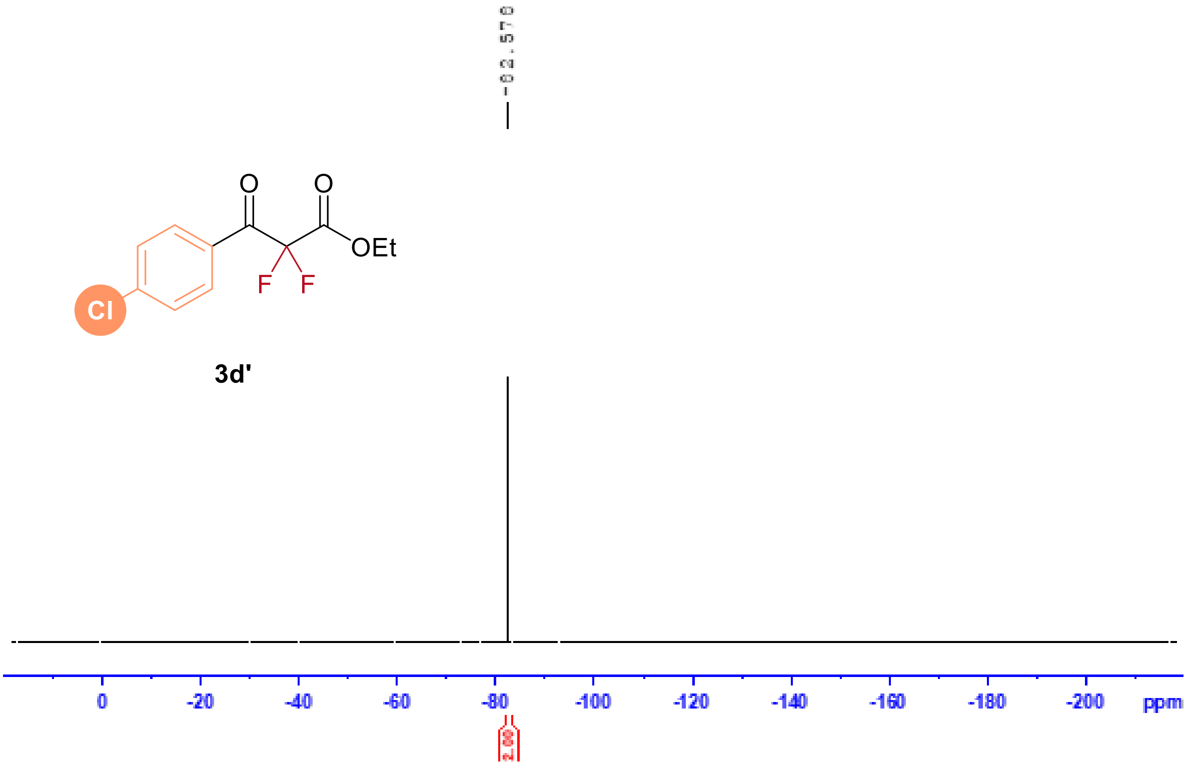

^{1}H NMR of **3d″**

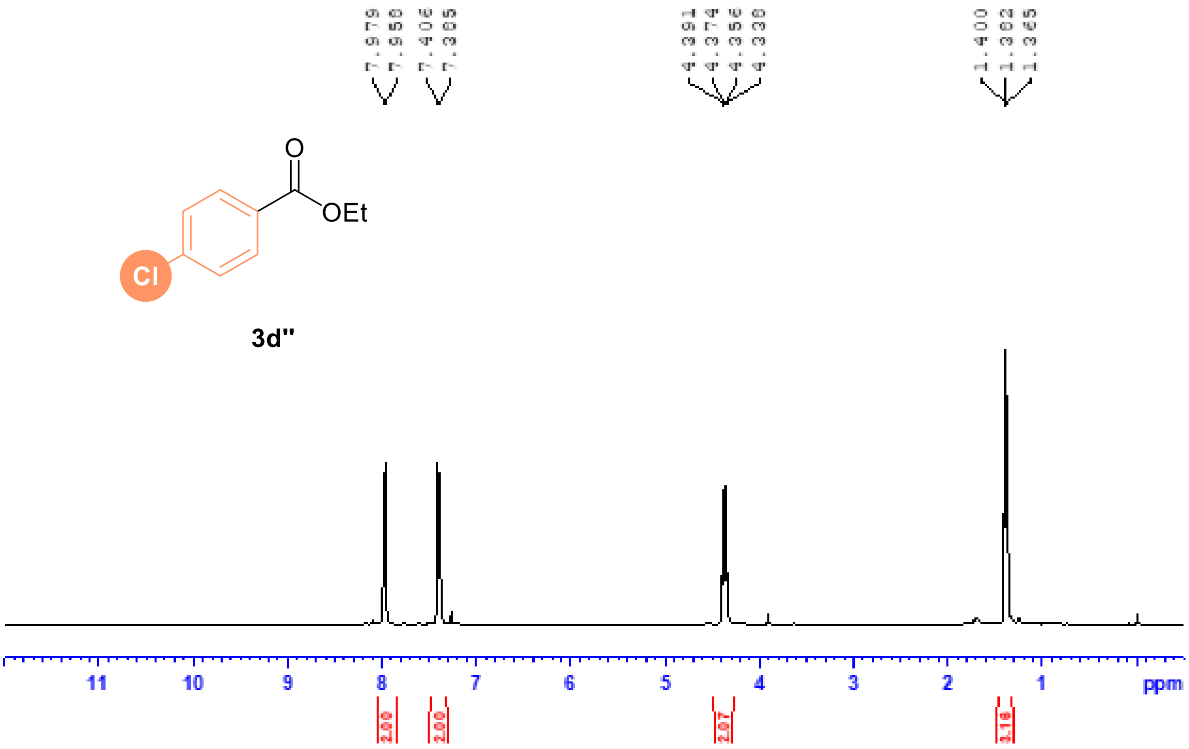

^{13}C NMR of **3d″**

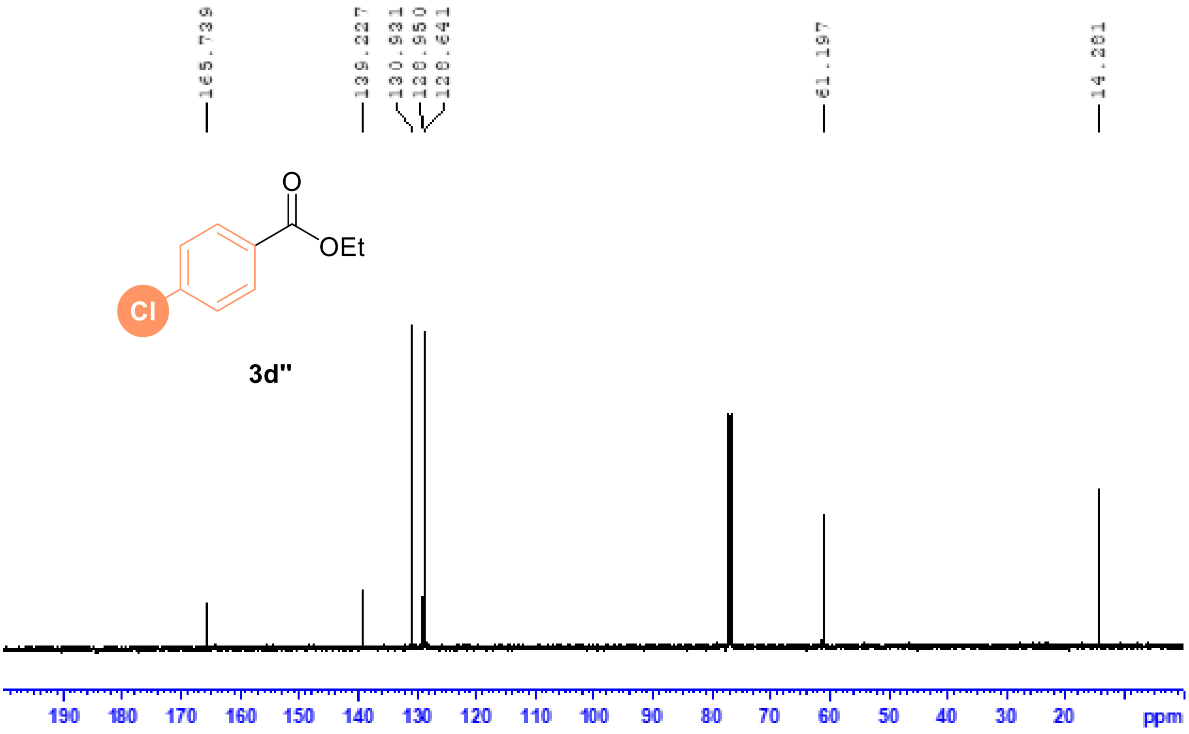

^{1}H NMR of **3e′**

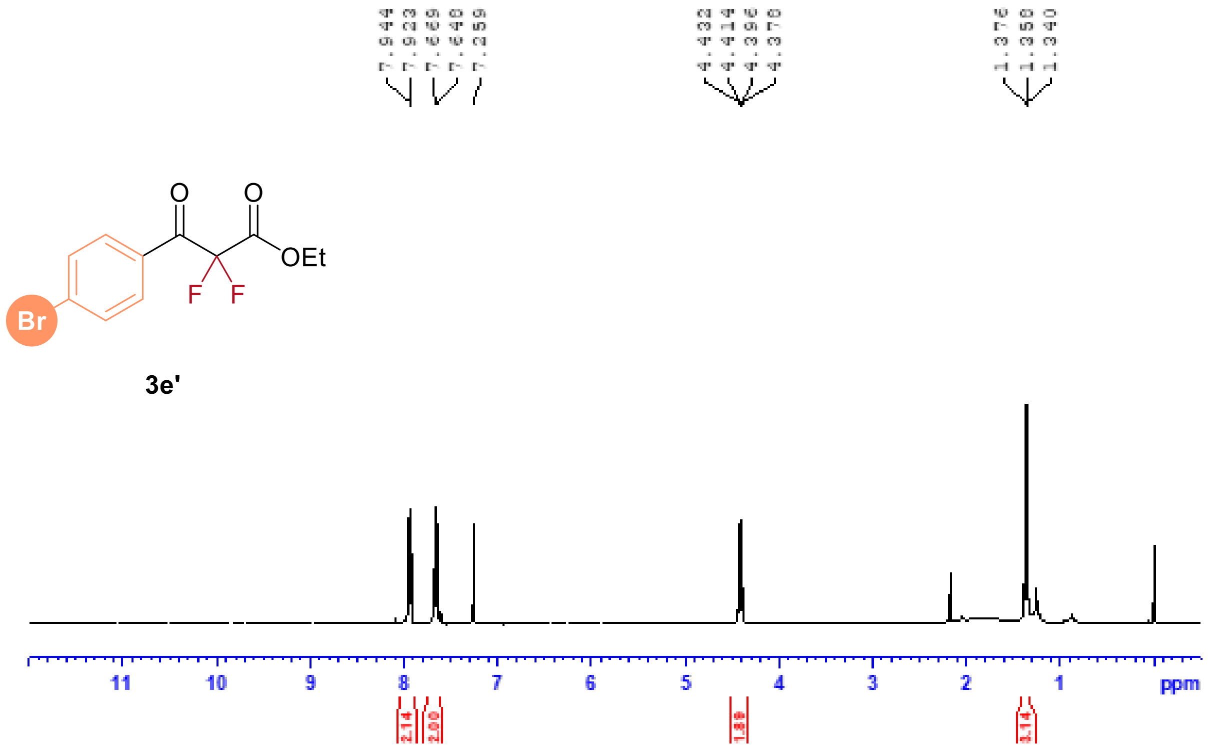

^{13}C NMR of **3e′**

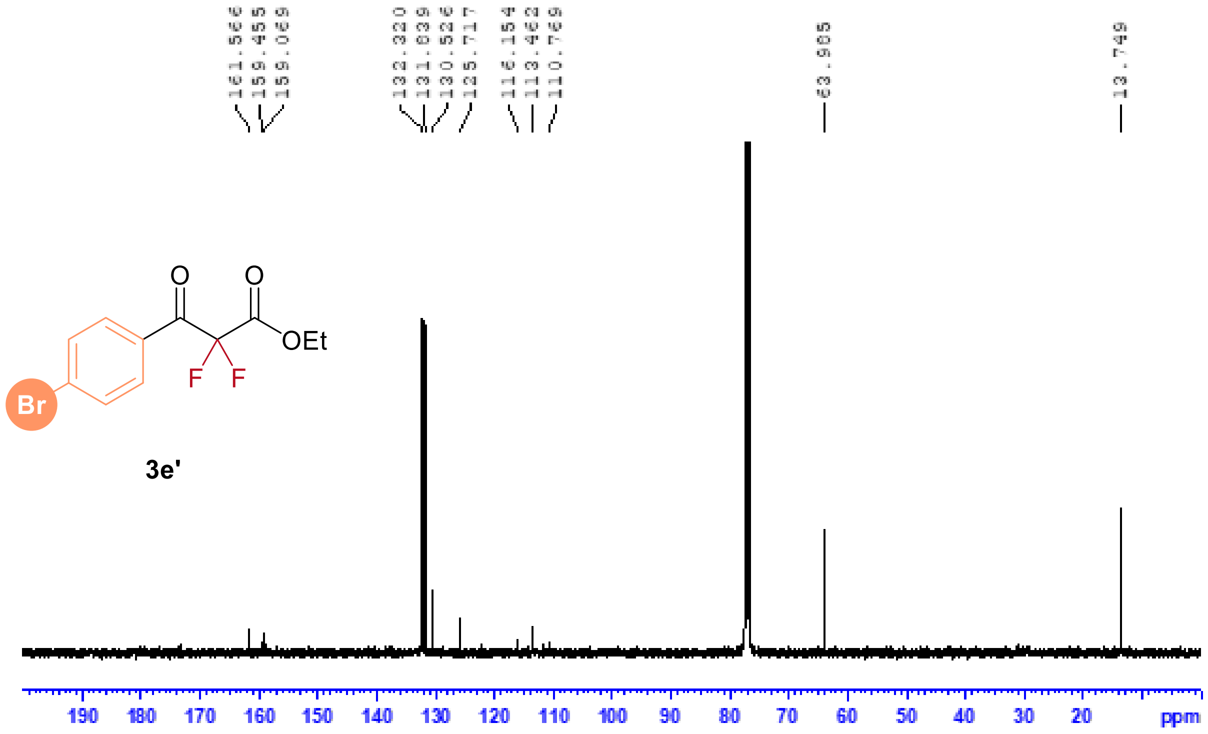

^{19}F NMR of **3e′**

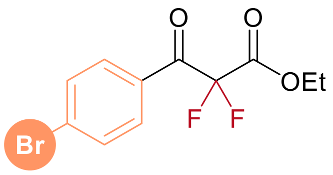

3e'

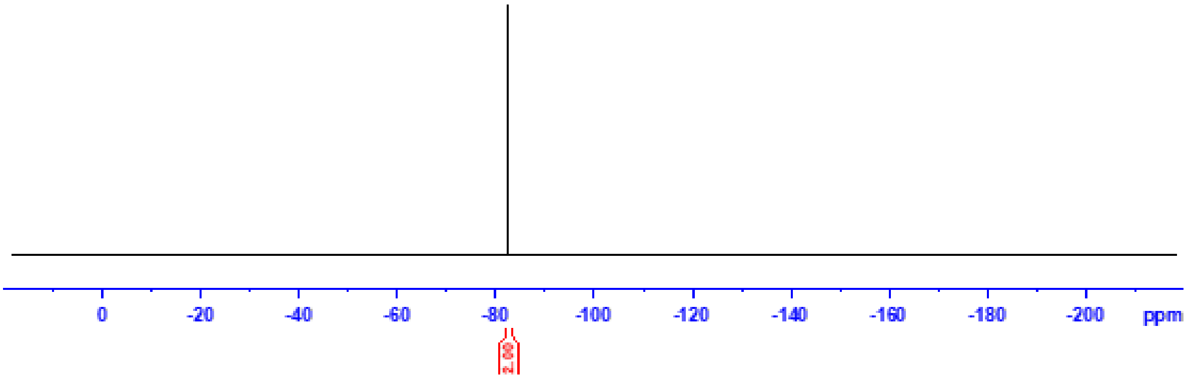

^{1}H NMR of **3e″**

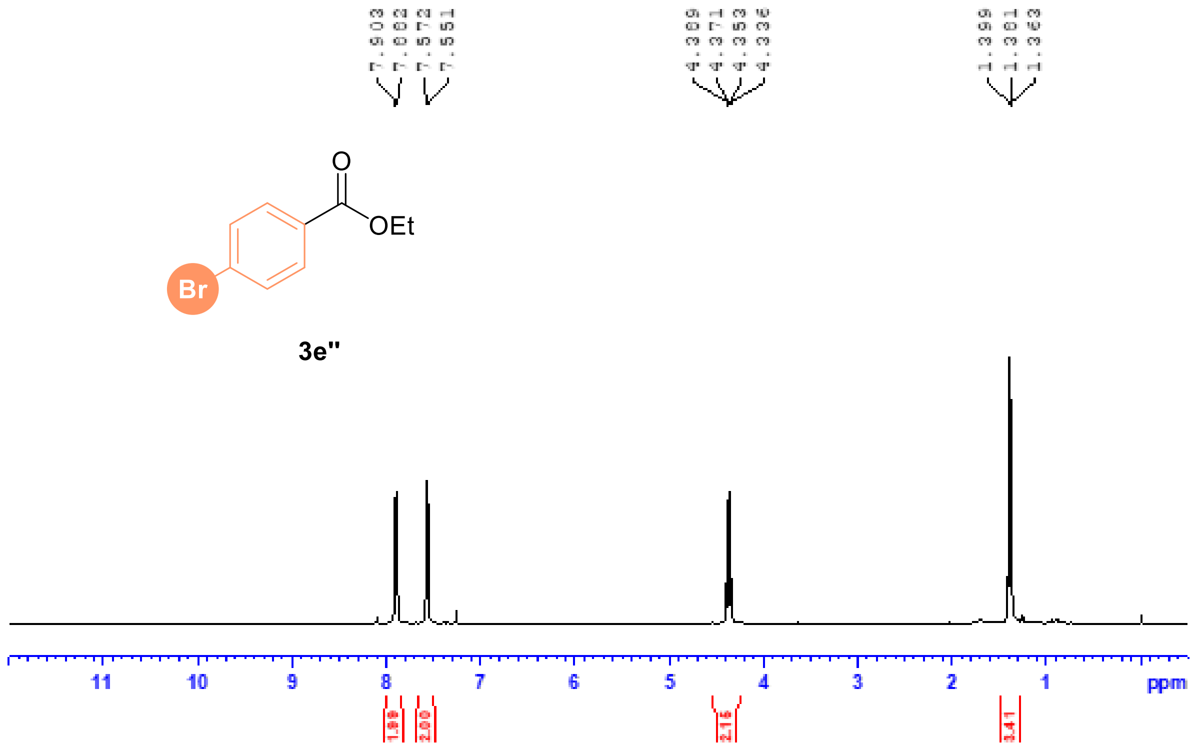

^{13}C NMR of **3e″**

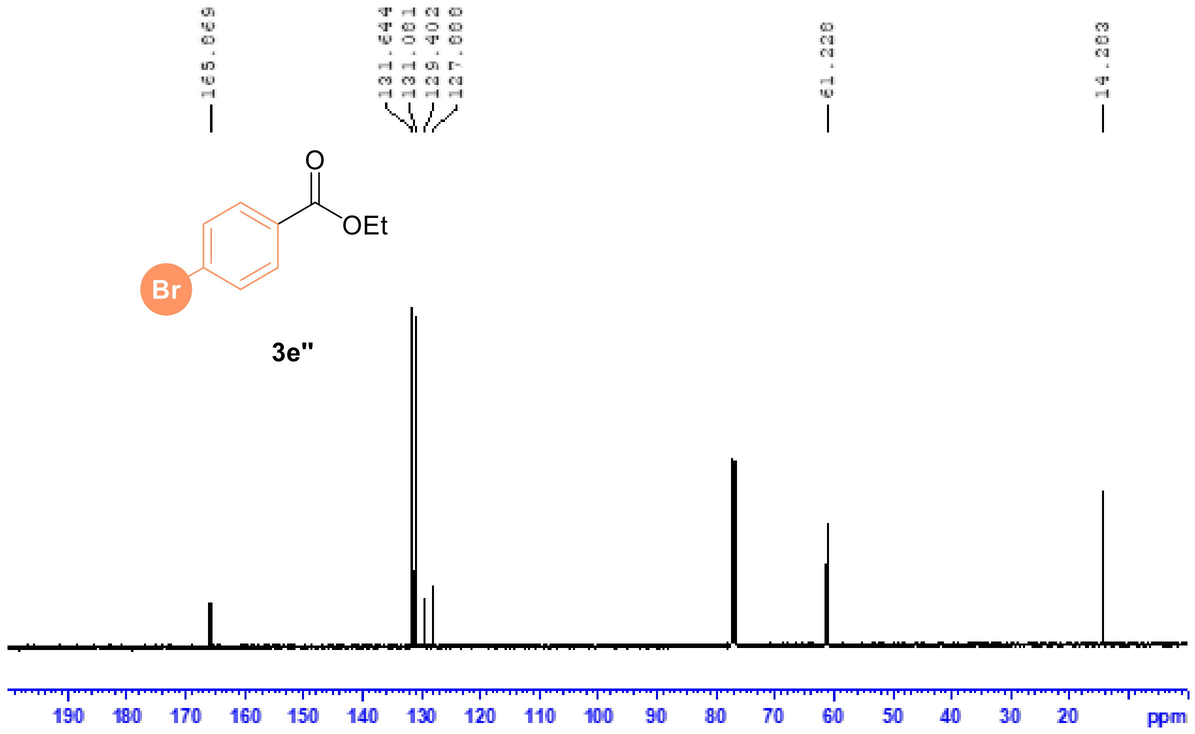

^{1}H NMR of **7j′**

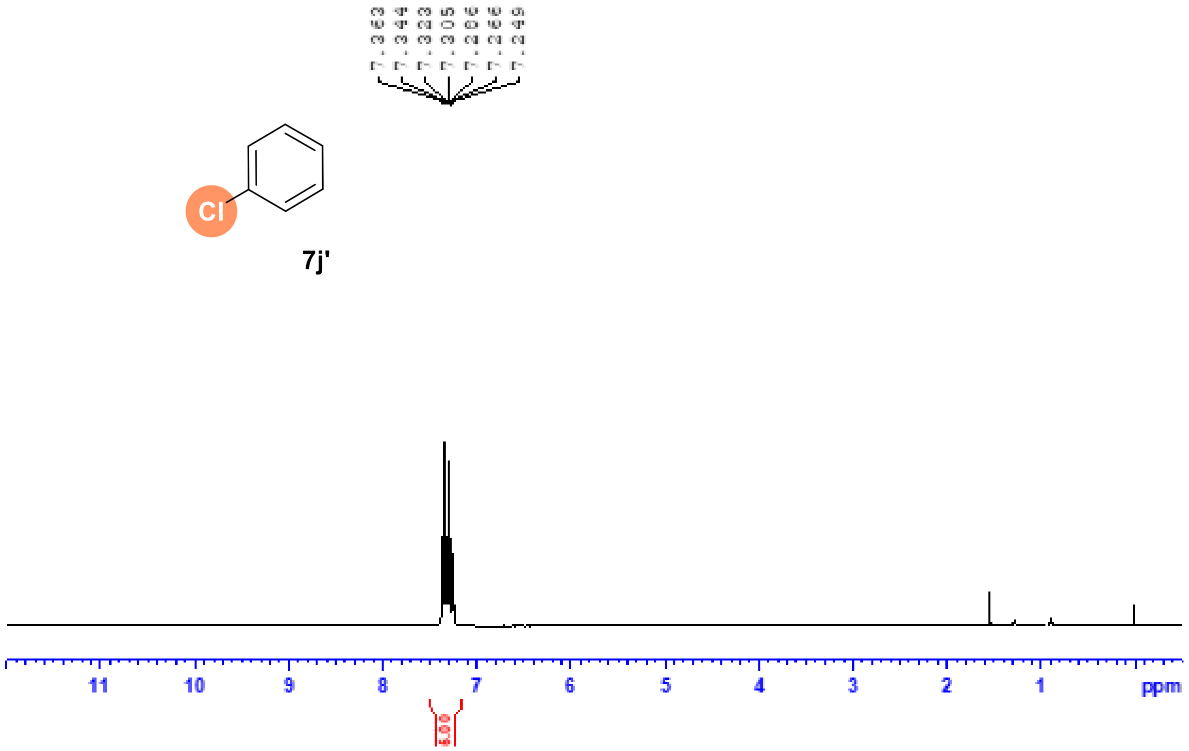

^{13}C NMR of **7j′**

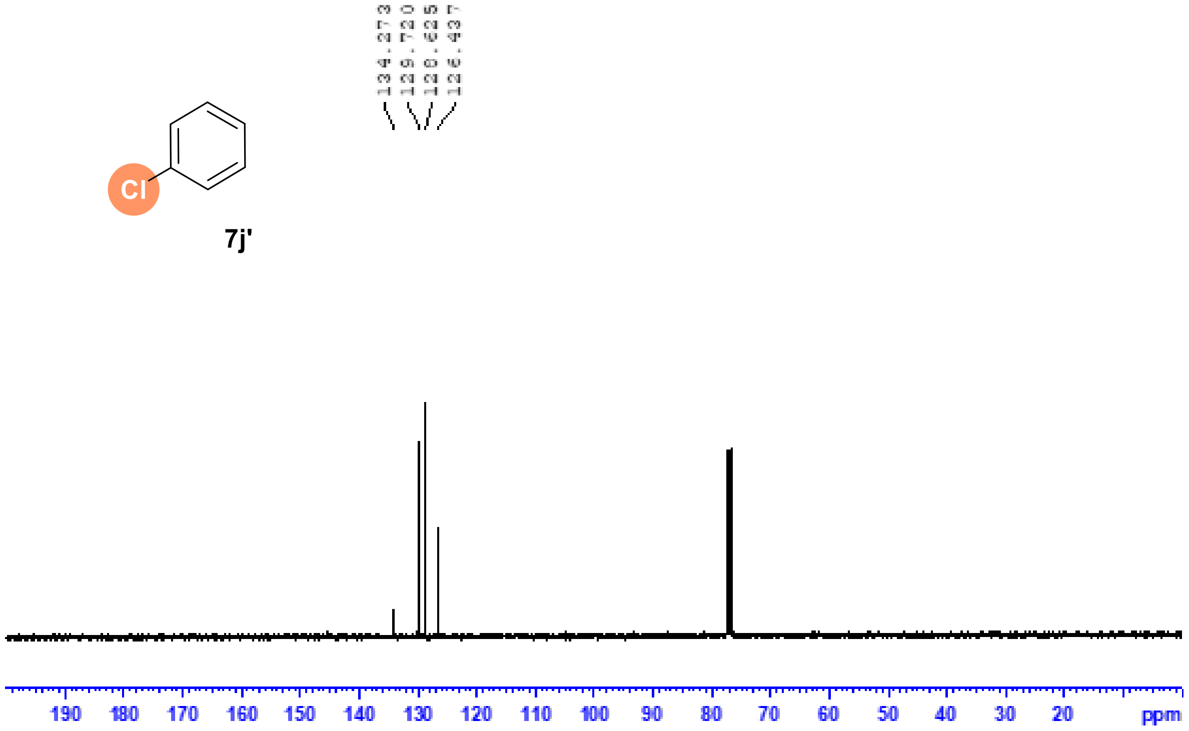

^{1}H NMR of **7h′**

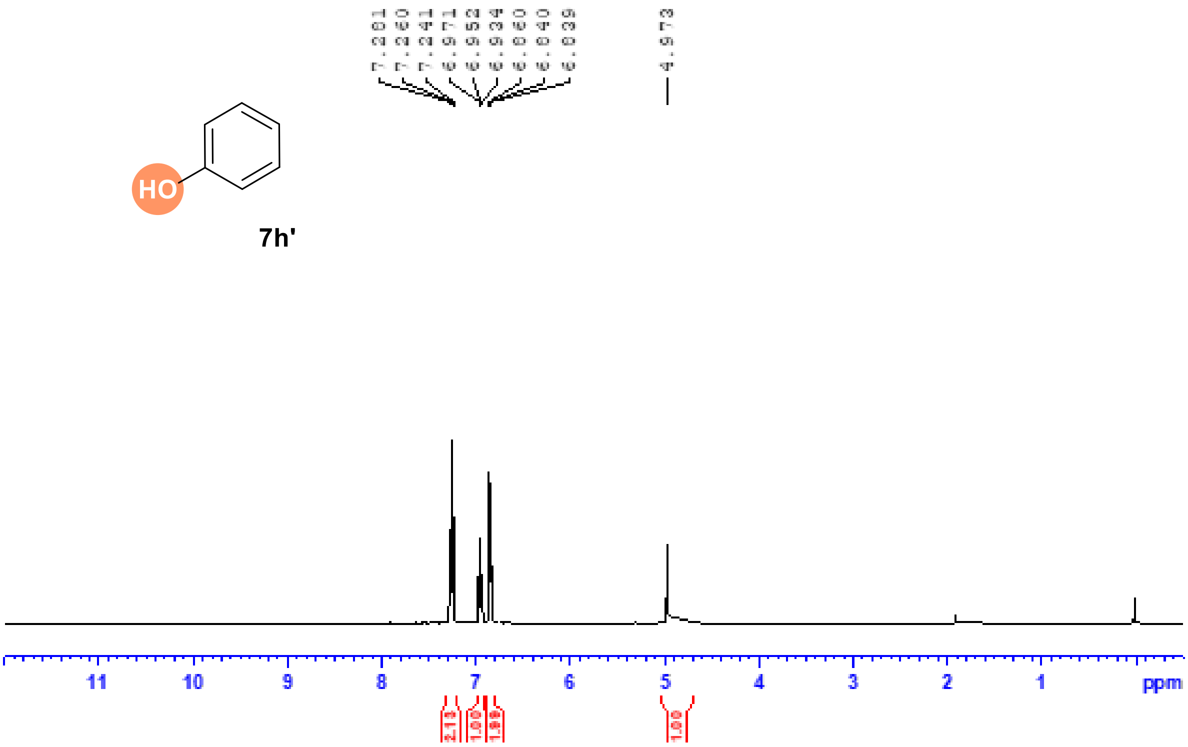

7h'

^{13}C NMR of **7h′**

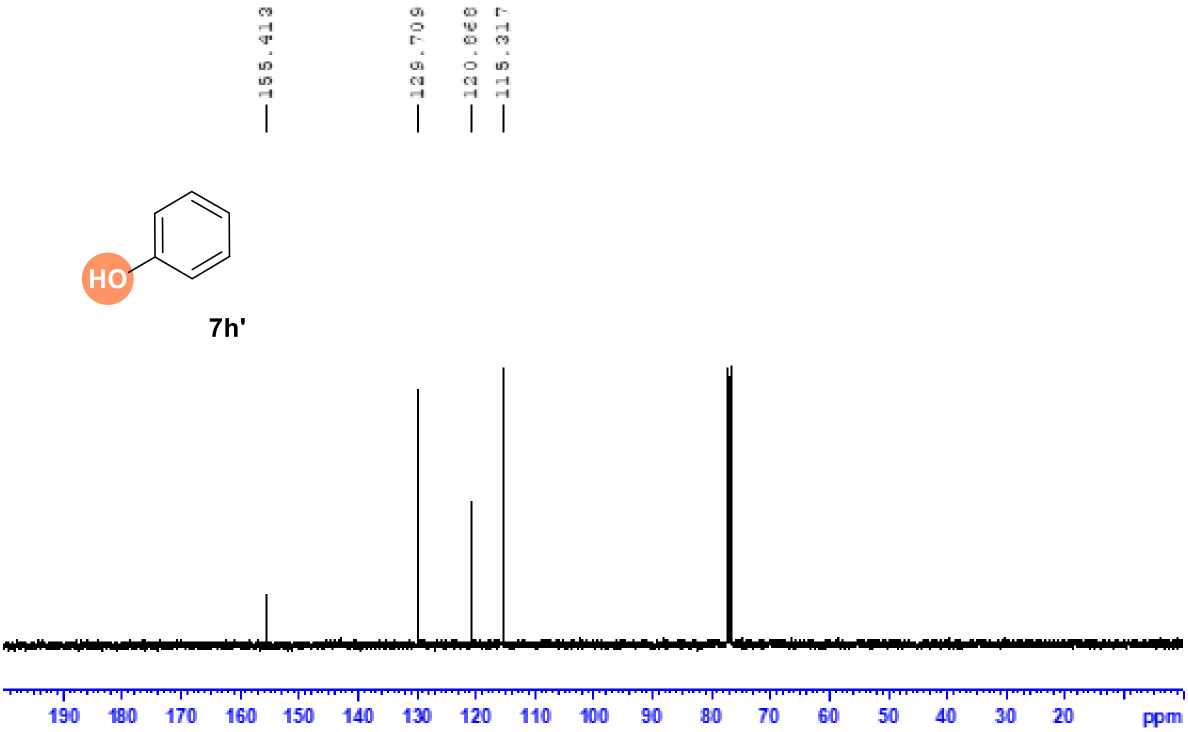

7h'

^{1}H NMR of **7g′**

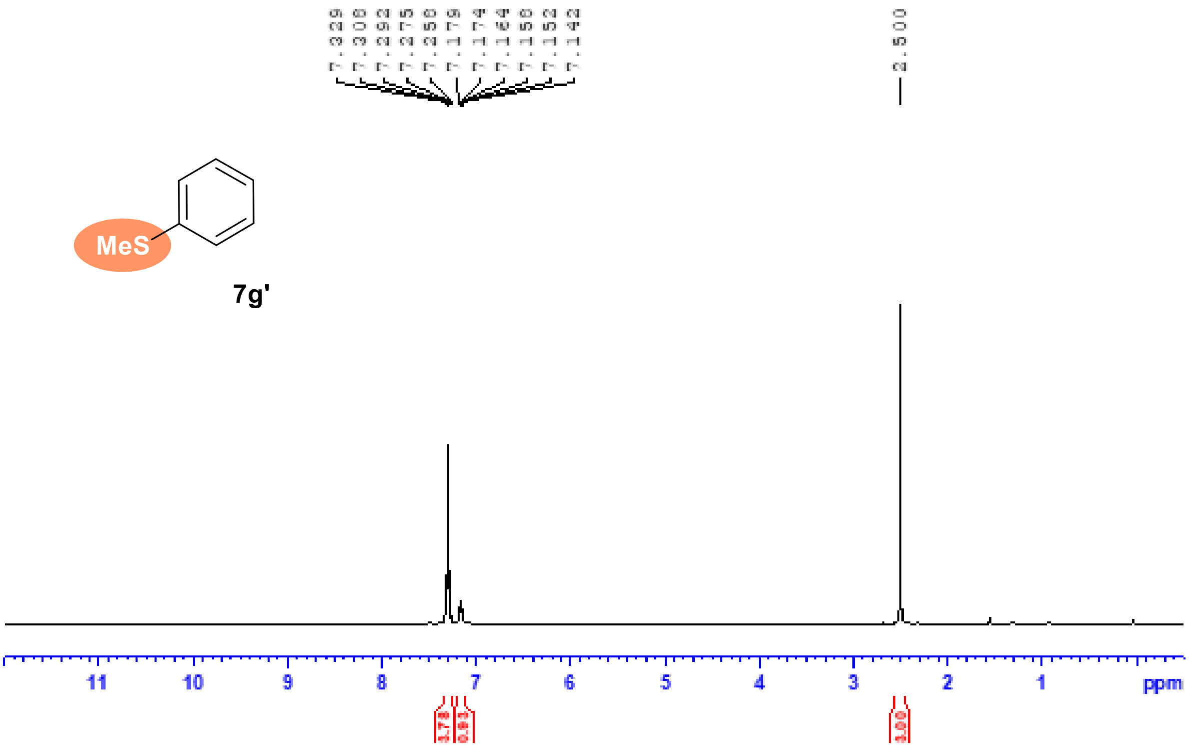

^{13}C NMR of **7g′**

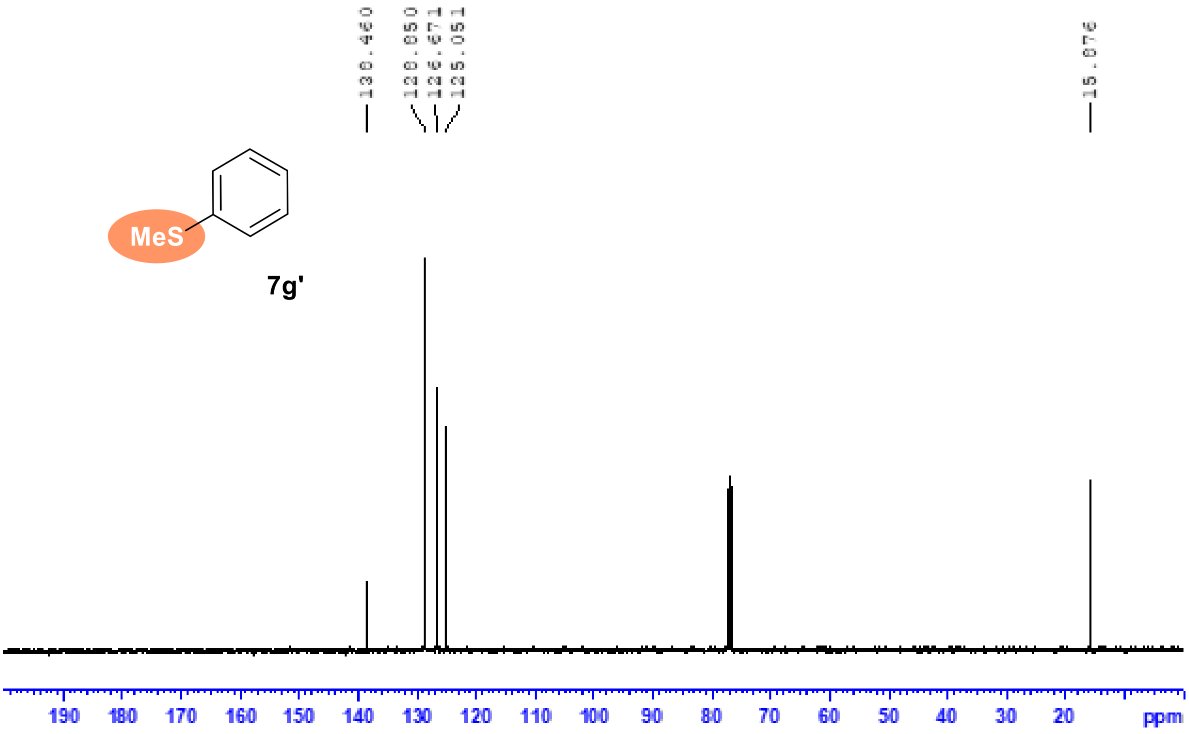

^{1}H NMR of **10**

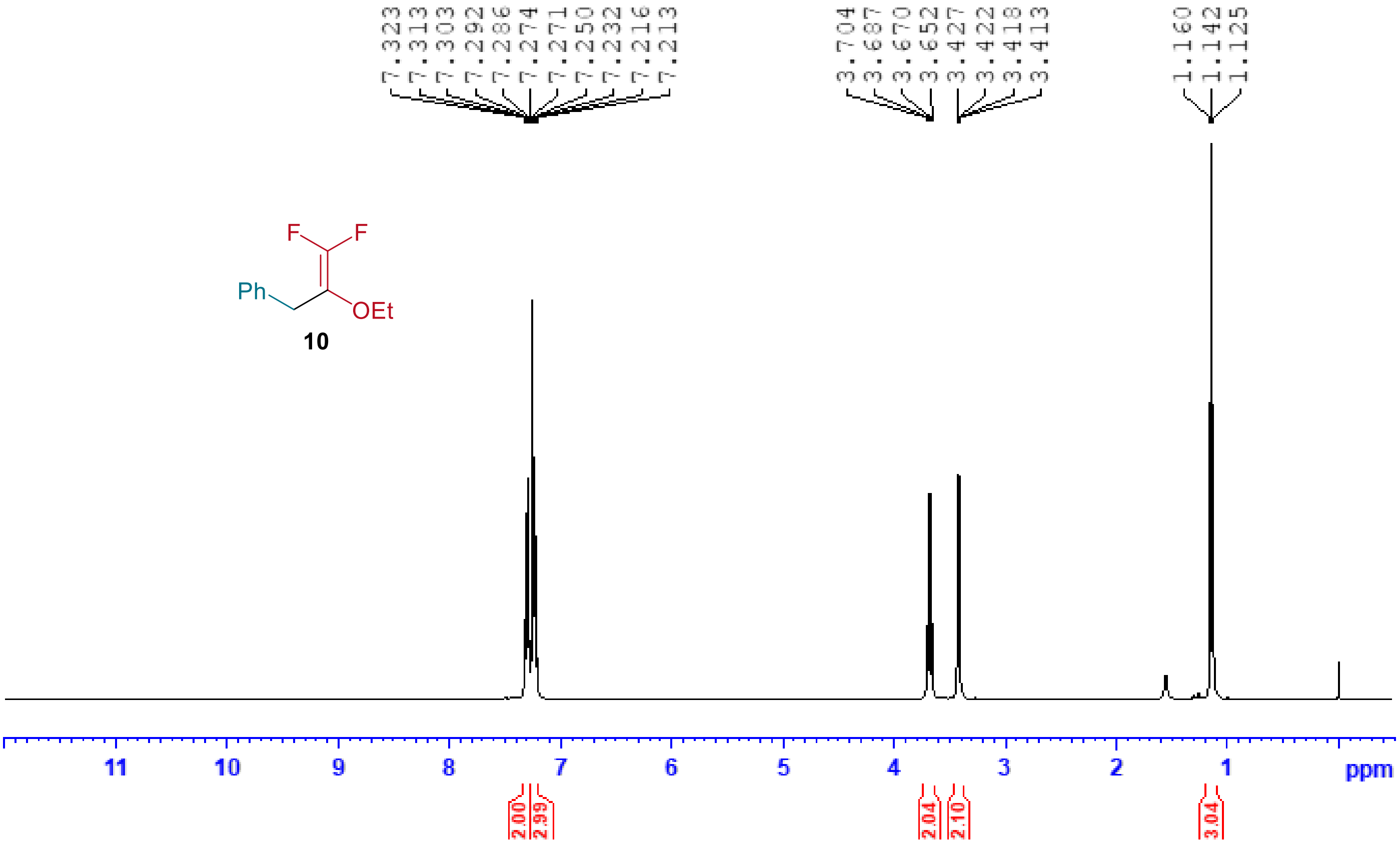

^{13}C NMR of **10**

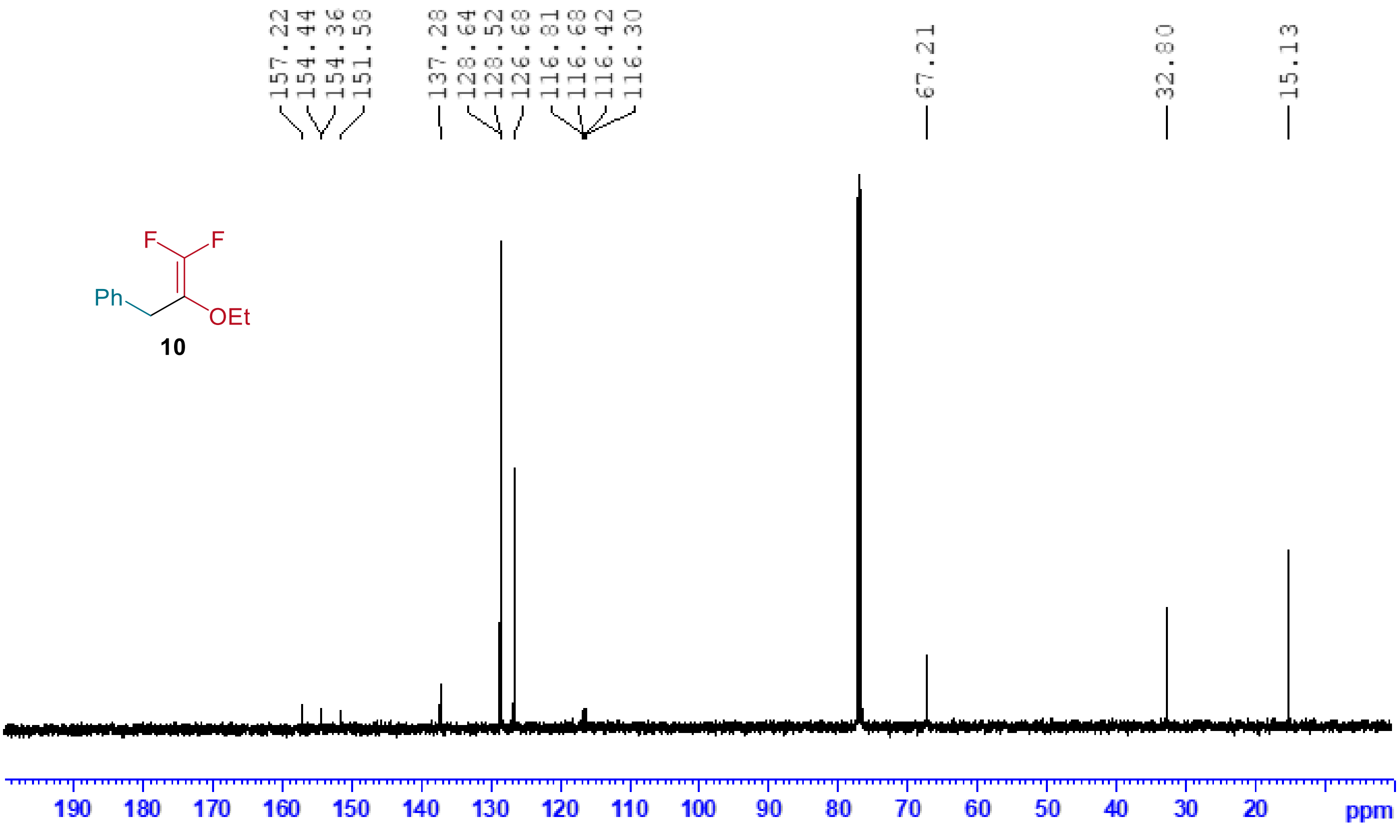

^{19}F NMR of **10**

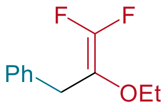

10

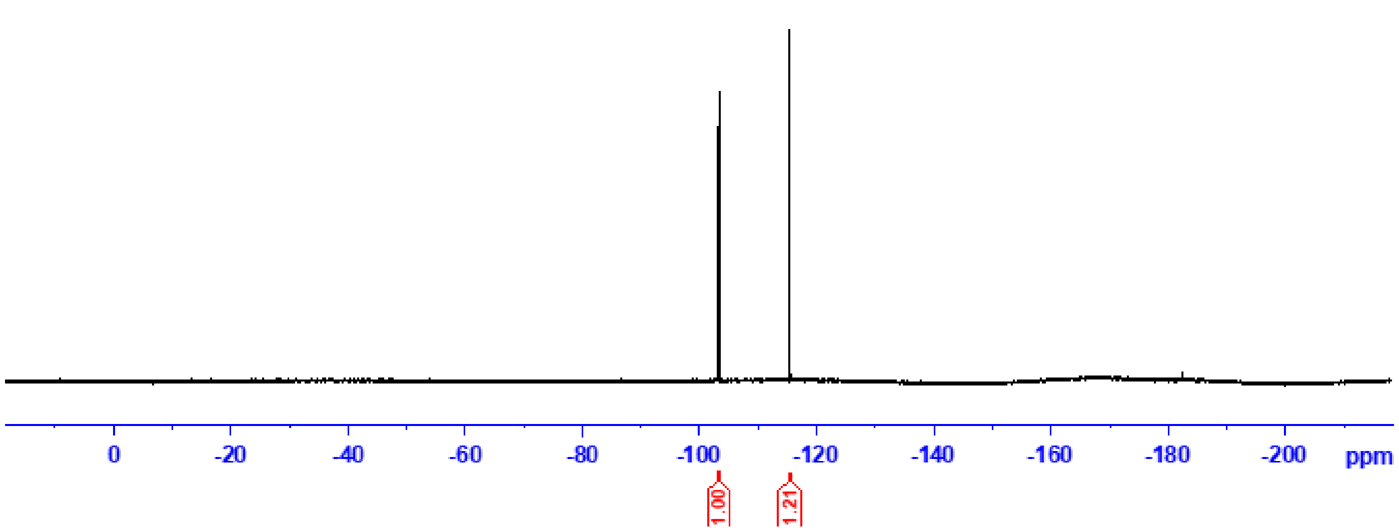

^{1}H NMR of **11**

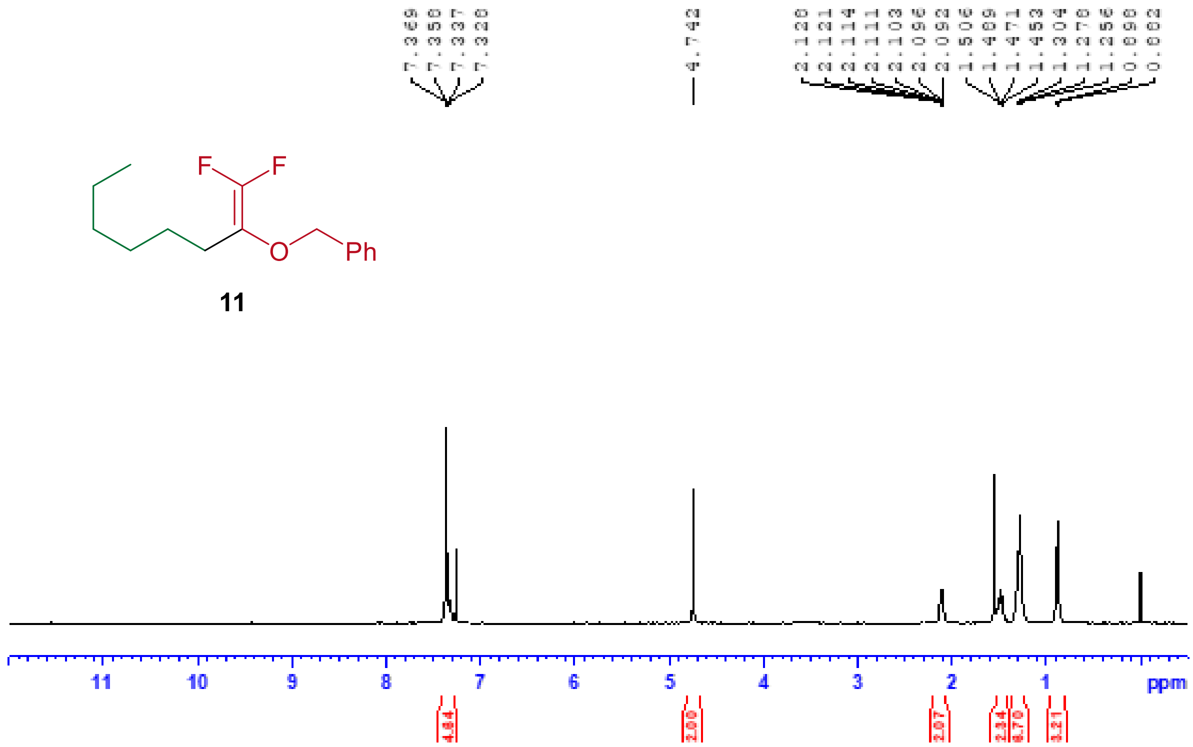

^{13}C NMR of **11**

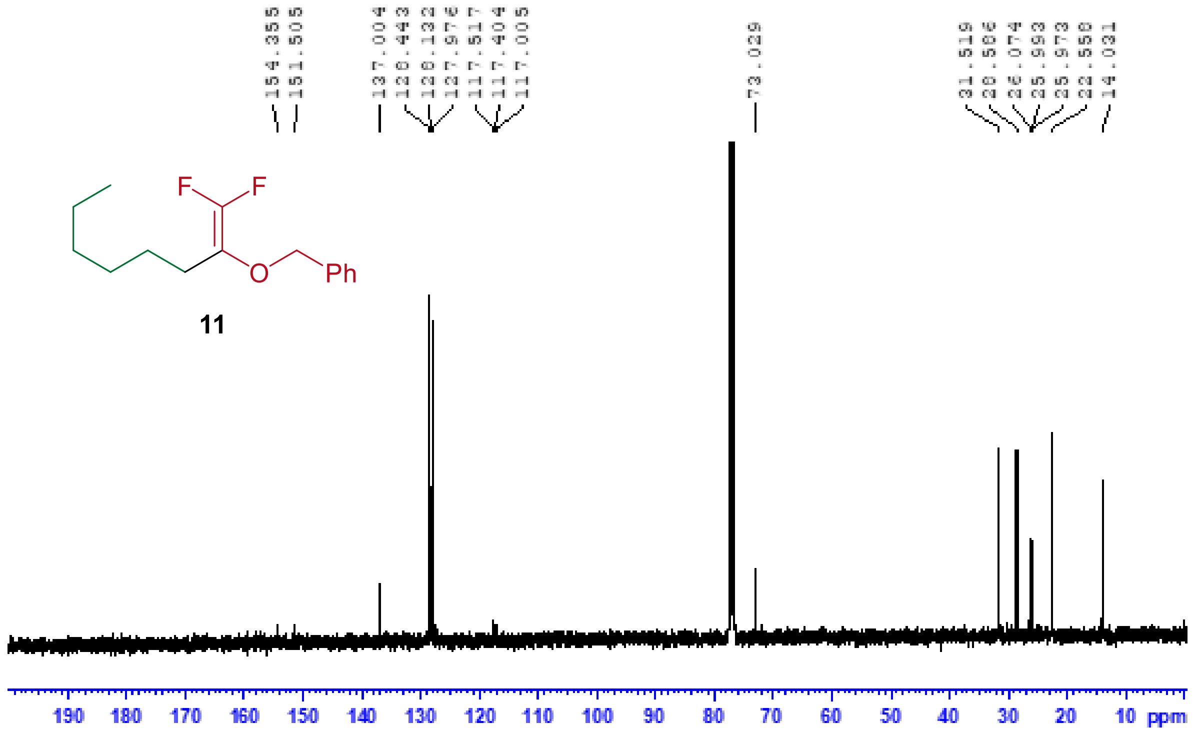

^{19}F NMR of **11**

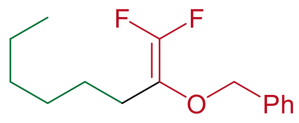

11

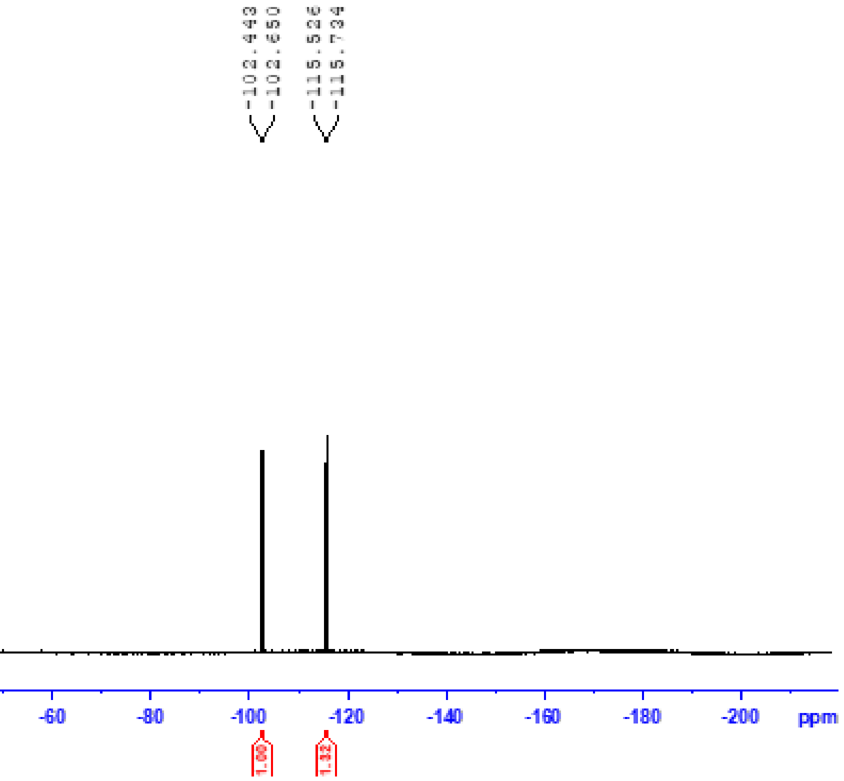

8. Supplementary References

1. Liu, Y.-Y.; Yu, X.-Y.; Chen, J.-R.; Qiao, M.-M.; Qi, X.; Shi, D.-Q.; Xiao, W.-J. Visible-Light-Driven Aza-ortho-quinone Methide Generation for the Synthesis of Indoles in a Multicomponent Reaction. *Angew. Chem. Int. Ed.* **56**, 9527–9531 (2017).
2. Sha, W.; Zhang, W.; Ni, S.; Mei, H.; Han, J.; Pan, Y. Photoredox-Catalyzed Cascade Difluoroalkylation and Intramolecular Cyclization for Construction of Fluorinated γ-Butyrolactones. *J. Org. Chem.* **82**, 9824–9831 (2017).
3. Wu, J.; Zhao, Q.; Wilson, T. C.; Verhoog, S.; Lu, L.; Gouverneur, V.; Shen, Q. Synthesis and Reactivity of α-Cumyl Bromodifluoromethanesulfenate: Application to the Radiosynthesis of [^{18}F]ArylSCF_3. *Angew. Chem. Int. Ed.* **58**, 2413–2417 (2018).
4. Tzschucke, C. C.; Murphy, J. M.; Hartwig, J. F. Arenes to Anilines and Aryl Ethers by Sequential Iridium-Catalyzed Borylation and Copper-Catalyzed Coupling. *Org. Lett.* **9**, 761–764 (2007).
5. Xiao, Y.-L.; Guo, W.-H.; He, G.-Z.; Pan, Q.; Zhang, X. Nickel-Catalyzed Cross-Coupling of Functionalized Difluoromethyl Bromides and Chlorides with Aryl Boronic Acids: A General Method for Difluoroalkylated Arenes. *Angew. Chem. Int. Ed.* **53**, 9909–9913 (2014).
6. Feng, Z.; Min, Q.-Q.; Xiao, Y.-L.; Zhang, B.; Zhang, X. Palladium-Catalyzed Difluoroalkylation of Aryl Boronic Acids: A New Method for the Synthesis of Aryldifluoromethylated Phosphonates and Carboxylic Acid Derivatives. *Angew. Chem. Int. Ed.* **53**, 1669–1673 (2014).
7. Zhao, H.-Y.; Feng, Z.; Luo, Z.; Zhang, X. Carbonylation of Difluoroalkyl Bromides Catalyzed by Palladium. *Angew. Chem. Int. Ed.* **55**, 10401–10405 (2016).

8. Stephens, P. J.; Devlin, F. J.; Chabalowski, C. F.; Frisch, M. J. Ab Initio Calculation of Vibrational Absorption and Circular Dichroism Spectra Using Density Functional Force Fields. *J. Phys. Chem.* **98**, 11623–11627 (1994).

9. Hay, P. J.; Wadt, W. R. Ab Initio Effective Core Potentials for Molecular Calculations. Potentials for the Transition Metal Atoms Sc to Hg. *J. Chem. Phys.* **82**, 270–283 (1985).

10. Wadt, W. R.; Hay, P. J. Ab Initio Effective Core Potentials for Molecular Calculations. Potentials for Main Group Elements Na to Bi. *J. Chem. Phys.* **82**, 284–298 (1985).

11. Ehlers, A. W.; Böhme, M.; Dapprich, S.; Gobbi, A.; Höllwarth, A.; Jonas, V.; Köhler, K. F.; Stegmann, R.; Veldkamp, A.; Frenking, G. A Set of F-Polarization Functions for Pseudo-Potential Basis Sets of the Transition Metals Sc-Cu, Y-Ag and La-Au. *Chem. Phys. Lett.* **208**, 111–114 (1993).

12. Fukui, K. The Path of Chemical Reactions - The IRC Approach. *Acc. Chem. Res.* **14**, 363–368 (1981).

13. Frisch, M. J.; Trucks, G. W.; Schlegel, H. B.; Scuseria, G. E.; Robb, M. A.; Cheeseman, J. R.; Scalmani, G.; Barone, V.; Mennucci, B.; Petersson, G. A.; Nakatsuji, H.; Caricato, M.; Li, X.; Hratchian, H. P.; Izmaylov, A. F.; Bloino, J.; Zheng, G.; Sonnenberg, J. L.; Hada, M.; Ehara, M.; Toyota, K.; Fukuda, R.; Hasegawa, J.; Ishida, M.; Nakajima, T.; Honda, Y.; Kitao, O.; Nakai, H.; Vreven, T.; Montgomery, J. A., Jr.; Peralta, J. E.; Ogliaro, F.; Bearpark, M.; Heyd, J. J.; Brothers, E.; Kudin, K. N.; Staroverov, V. N.; Kobayashi, R.; Normand,

J.; Raghavachari, K.; Rendell, A.; Burant, J. C.; Iyengar, S. S.; Tomasi, J.; Cossi, M.; Rega, N.; Millam, M. J.; Klene, M.; Knox, J. E.; Cross, J. B.; Bakken, V.; Adamo, C.; Jaramillo, J.; Gomperts, R.; Stratmann, R. E.; Yazyev, O.; Austin, A. J.; Cammi, R.; Pomelli, C.; Ochterski, J. W.; Martin, R. L.; Morokuma, K.; Zakrzewski, V. G.; Voth, G. A.; Salvador, P.; Dannenberg, J. J.; Dapprich, S.; Daniels, A. D.; Farkas, Ö.; Foresman, J. B.; Ortiz, J. V.; Cioslowski, J.; Fox, D. J. *Gaussian 09, Revision D.01*; Gaussian, Inc., Wallingford, CT: 2009.
